# Multiple distinct small RNAs originate from the same microRNA precursors

**DOI:** 10.1186/gb-2010-11-8-r81

**Published:** 2010-08-09

**Authors:** Weixiong Zhang, Shang Gao, Xuefeng Zhou, Jing Xia, Padmanabhan Chellappan, Xiang Zhou, Xiaoming Zhang, Hailing Jin

**Affiliations:** 1 Department of Computer Science and Engineering, Washington University in Saint Louis, Campus Box 1045, Saint Louis, MO 63130, USA; 2 Department of Genetics, Washington University School of Medicine, Campus Box 8232, Saint Louis, MO 63110, USA; 3 Department of Plant Pathology and Microbiology, Center for Plant Cell Biology, Institute for Integrative Genome Biology, 900 University Ave, University of California, Riverside, CA 92521, USA

## Abstract

**Background:**

MicroRNAs (miRNAs), which originate from precursor transcripts with stem-loop structures, are essential gene expression regulators in eukaryotes.

**Results:**

We report 19 miRNA precursors in *Arabidopsis *that can yield multiple distinct miRNA-like RNAs in addition to miRNAs and miRNA*s. These miRNA precursor-derived miRNA-like RNAs are often arranged in phase and form duplexes with an approximately two-nucleotide 3'-end overhang. Their production depends on the same biogenesis pathway as their sibling miRNAs and does not require RNA-dependent RNA polymerases or RNA polymerase IV. These miRNA-like RNAs are methylated, and many of them are associated with Argonaute proteins. Some of the miRNA-like RNAs are differentially expressed in response to bacterial challenges, and some are more abundant than the cognate miRNAs. Computational and expression analyses demonstrate that some of these miRNA-like RNAs are potentially functional and they target protein-coding genes for silencing. The function of some of these miRNA-like RNAs was further supported by their target cleavage products from the published small RNA degradome data. Our systematic examination of public small-RNA deep sequencing data from four additional plant species (*Oryza sativa*, *Physcomitrella patens*, *Medicago truncatula *and *Populus trichocarpa*) and four animals (*Homo sapiens*, *Mus musculus*, *Caenorhabditis elegans *and *Drosophila*) shows that such miRNA-like RNAs exist broadly in eukaryotes.

**Conclusions:**

We demonstrate that multiple miRNAs could derive from miRNA precursors by sequential processing of Dicer or Dicer-like proteins. Our results suggest that the pool of miRNAs is larger than was previously recognized, and miRNA-mediated gene regulation may be broader and more complex than previously thought.

## Background

MicroRNAs (miRNAs) are small regulatory RNAs that play a fundamental role in gene expression regulation in eukaryotes through mRNA cleavage, RNA degradation, translation inhibition, or DNA methylation [1-7]. miRNAs belong to a large repertoire of regulatory small RNAs, which also includes small interfering RNAs (siRNAs) [8-11]. Most miRNA genes (*MIR*) are transcribed by RNA polymerase II (Pol II) [12,13]. The resulting single-stranded miRNA precursors fold into stem-loop structures that can be recognized by RNase III-type enzymes, Drosha (as in animals) and Dicer or Dicer-like proteins (DCLs; as in plants), that sequentially cleave the precursors to liberate miRNA-miRNA* duplexes from the hairpins (miRNA* is a small RNA on the opposite arm of the miRNA in the hairpin with partial complementarity to the miRNA) [3,6,14]. The mature miRNAs are subsequently incorporated into Argonaute (AGO) family proteins, and then they target mRNAs through perfect or partially complementary base pairing [15]. miRNAs are normally more abundant than miRNA*s [3,6,14], but there are cases when miRNA* sequences are more abundant and can interact with AGO proteins to exert their function [16]; when the abundances of miRNAs and miRNA*s are comparable, they are called miR-5p and miR-3p, depending on their positions relative to the 5'-end of the sequences [17,18]. *Arabidopsis *contains four Dicer-like proteins, DCL1 to DCL4. The biogenesis of *Arabidopsis *miRNAs depends mainly on DCL1, with that of a few relying on DCL4 [8,19]. *Arabidopsis *miRNAs are stabilized through 3'-end methylation by the RNA methyltransferase HEN1, which protects them from uridylation and subsequent RNA degradation [20,21].

In contrast to miRNAs, siRNAs are derived from double-stranded RNA molecules and have multiple sources of origin [6,8]. Four classes of siRNAs have been found in plants. The first class includes natural antisense transcript (nat)-siRNA which is derived from *cis*-natural antisense transcripts, the so-called nat-siRNAs. They are often induced by abiotic and biotic stresses, are generated by DCL1 and/or DCL2, and are often dependent on RNA-dependent RNA polymerase (RDR) 6 and Pol IV [22-25]. The second class comprises endogenous *trans*-acting siRNAs (tasiRNAs), which are encoded by *TAS *genes [8]. miRNA-mediated cleavage of a *TAS *transcript serves as a template for RDR6 to synthesize a double-stranded RNA, which is subsequently cleaved into approximately 21-nucleotide phased tasiRNAs by DCL4. The third class of siRNAs comprises the heterochromatic siRNAs (hc-siRNAs) [10]. hc-siRNAs normally arise from transposon and repeat regions of the genome, and often silence mobile and repeat elements via DNA methylation and chromatin modification. The formation of hc-siRNAs requires DCL3, RDR2 and Pol IV. The fourth class comprises long siRNAs (lsiRNAs), which are 30 to 40 nucleotides in length [26]. The biogenesis of lsiRNAs requires DCL1 and is also partially dependent on RDR and Pol IV. Therefore, an effective way to distinguish miRNAs from various siRNAs is to examine the major distinctive components of their biogenesis. For example, the biogenesis of miRNAs does not require RDRs or Pol IV.

A structural property of miRNAs is that their precursors form foldback hairpin structures. One miRNA-miRNA* duplex is typically expected to arise from a miRNA precursor [3,14,27]. Nevertheless, some early work also observed additional small RNAs beyond miRNAs and miRNA*s, but such small RNAs were normally considered to be byproducts of Dicer activities and have never been systematically investigated [19,28-32]. Recent studies in animals identified miRNA-offset RNAs (moRNAs) in a chordate [33], human [34], and a herpesvirus [35], but the biogenesis and possible functions of these small RNAs remain to be determined. In a deep-sequencing-based study of small RNAs from bacterial-challenged *Arabidopsis thaliana*, we identified a substantial number of sequencing reads that can map perfectly onto many miRNA precursors even though they do not correspond to the mature miRNA or miRNA* sequences. Most of these small RNAs form pairing partners similar to miRNA-miRNA* duplexes with a two-nucleotide 3'-end overhang and are arranged in phasing. Moreover, we found that they depend on the same biogenesis pathway as the known miRNAs. Furthermore, multiple lines of evidence suggest that some of these miRNA-like RNAs are authentic miRNAs. First, some of them are differentially expressed upon bacterial challenges, and some are more abundant than their sibling miRNAs. Second, many of these miRNA-like RNAs can be associated with AGO proteins. Third, some of them have predicted protein-coding targets with similar functions, and several of their target cleavage products are present when performing parallel analysis of RNA ends (PARE) or in degradome data [36-38]. Fourth, expression analysis using Dicer mutants further supports that some of these miRNA-like RNAs silence their predicted target genes. Moreover, our systematic genome-wide survey of publically available small-RNA deep sequencing data shows that such miRNA-like RNAs broadly exist in plants (*Oryza sativa*, *Physcomitrella patens*, *Medicago truncatula *and *Populus trichocarpa*) and animals (*Homo sapiens*, *Mus musculus*, *Caenorhabditis elegans *and *Drosophila melanogaster*).

## Results

To study the role of small RNAs in response to bacterial challenge, we prepared 13 small-RNA libraries from *Arabidopsis *infected with various *Pseudomonas syringae *pv. *tomato *(*Pst*) DC3000 strains and sequenced them using the Illumina SBS deep-sequencing platform. Sequencing data were collected at 6 and 14 hours post-inoculation (hpi) with 10 mM MgCl_2 _(mock), a type III secretion system mutated strain of *Pst *DC3000 *hrcC*, a virulent strain of *Pst *DC3000 carrying an empty vector (EV), and an avirulent strain of *Pst *DC3000 (*avrRpt2*). *Pst *DC3000 (*avrRpt2*) induces a hypersensitive response (HR) in *Arabidopsis *Col-0 that carries the cognate resistance gene *RPS2 *and leads to cell death symptoms (the hypersensitive response), usually at 15 to 16 hpi. Our samples were collected at 14 hpi, right before the hypersensitive response could be visualized. From a total of more than 24.6 million sequencing reads from all libraries, 13,985,938 reads perfectly matched the *Arabidopsis *genome and cDNAs, among which 2,578,531 were unique. After excluding reads shorter than 17-nucleotide and any that matched tRNAs, rRNAs, small nuclear RNAs (snRNAs), or small nucleolar RNAs (snoRNAs), the remaining reads were kept for further analysis. We detected the expression of 191 of the 207 *Arabidopsis *miRNAs listed in miRBase. The 13 libraries of sequencing reads have been deposited in the NCBI Gene Expression Omnibus (GEO) database [GEO:GSE19694] and a summary of the sequencing data is given in Table S1 in Additional file 1.

### Multiple distinct miRNA-like RNAs arise from a single miRNA precursor

**Figure 1:**
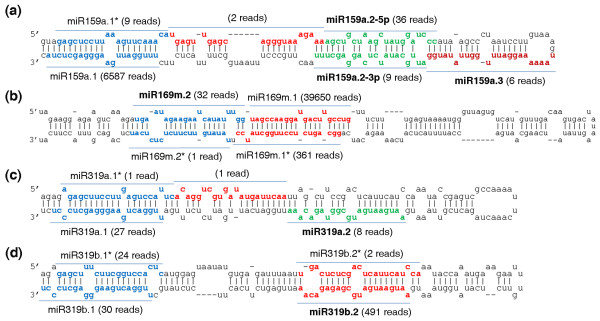
**Four miRNA precursors that can generate multiple miRNA-like RNAs**. **(a-d)**. Three miRNA precursors with miRNA-like RNAs in the upper arms close to the loops of their hairpins (a, c, d), and a miRNA precursor with miRNA-like RNAs in the lower arm of its hairpin (b). Note that miRNA-miRNA* duplexes for miRNA-like RNAs, with approximately two-nucleotide 3'-end overhangs, appear on the miR159, miR169m and miR319b precursors. The previously annotated miRNAs were named as miR*n*.1 (see main text for detail) and those miRNA-like RNAs having less than four reads were not named. For clarity, miR169m.2* and miR319b.2* are also indicated though the numbers of reads mapped to them were below the cutoff threshold of 5.

**Figure 2:**
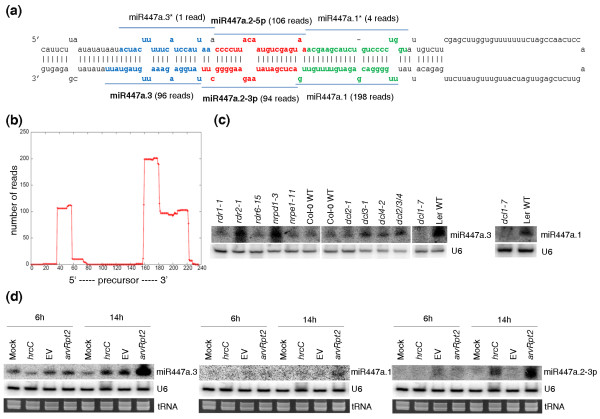
**miRNA-like RNAs from the miR447a precursor**. **(a) **The precursor fold-back structure and sequencing reads mapped to miR447a.1 (that is, miR447a) and the individual miRNA-like RNAs. For clarity, miR447a.3* and miR447a.1* are indicated though the numbers of reads mapped to them were below the cutoff threshold of 5. **(b) **Distribution of sequencing reads along the precursor. **(c) **Expression of miR447a.1 and miR447a.3 in various *Arabidopsis *mutants of small RNA pathways. **(d) **Expression of miR447a.3, miR447a.1, and miR447a.2-3p under the challenge of three *Pst *strains (*hrcC*, *avrRpt2 *and EV) and in a mock control at 6 and 14 hpi.

**Figure 3:**
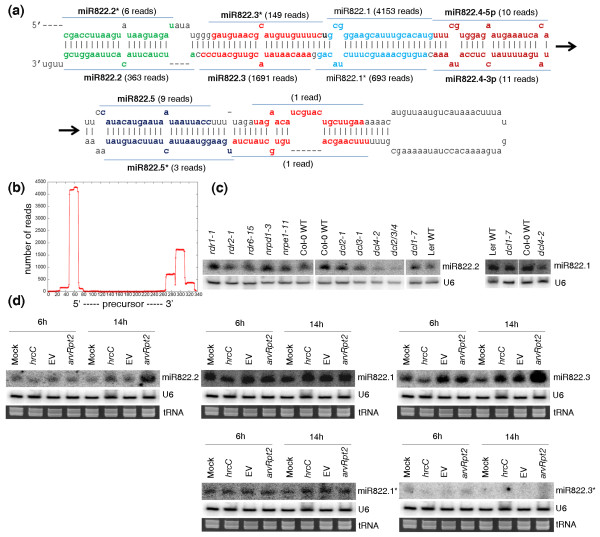
**miRNA-like RNAs from the miR822 precursor**. **(a) **The precursor fold-back structure and sequencing reads mapped to miR822.1 (that is, miR822) and individual miRNA-like RNAs. **(b) **Distribution of sequencing reads along the precursor. For clarity, miR822.5* is indicated though the number of reads mapped to it is below the cutoff threshold of 5. **(c) **Expression of miR822.1 and miR822.2 in various *Arabidopsis *mutants of small RNA pathways. **(d) **Expression of miR822.2, miR822.1, miR822.3, miR822.1* and miR822.3* under the challenge of three *Pst *strains (*hrcC*, *avrRpt2 *and EV) and in a mock control at 6 and 14 hpi.

**Figure 4:**
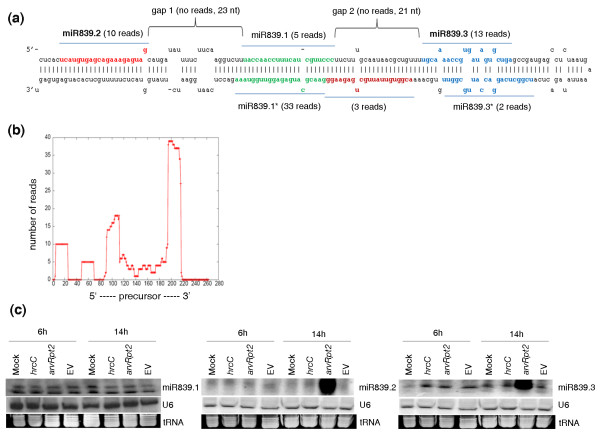
**miRNA-like RNAs from the miR839 precursor**. **(a) **The precursor fold-back structure and sequencing reads corresponding to the individual miRNA-like RNAs miR839.1 (that is, miR839) and miR839.1* (that is, miR839*). Note that miR839.1*, miR839.2 and miR839.3 have more sequencing reads than miR839.1. For clarity, miR839.3* was is though the number of reads mapped to it is below the cutoff threshold of 5. **(b) **Distribution of sequencing reads along the precursor. **(c) **Expression of miR839.1, miR839.2 and miR839.3 under the challenge of three *Pst *strains (hrcC, *avrRpt2 *and EV) and in a mock control at 6 and 14 hpi.

A key observation from our sequencing data is that multiple unique small-RNA reads could be generated from the same miRNA precursor. Specifically, we found a substantial number of reads that originated from the double-stranded stem regions of many miRNA precursors and yet are not themselves the mature miRNA or miRNA* sequences. In some cases, the number of these small-RNA reads is comparable to or even greater than the number of reads mapped to the mature miRNA or miRNA* sequences. Furthermore, using a set of stringent criteria (see Materials and methods), we observed that many sequencing reads that map to a miRNA precursor were arranged in phase, in which unique small-RNA reads followed one another in tandem or sometimes were separated by a gap of 21 to 22 nucleotides along the precursor [39]. Figures 1, 2, 3, and 4 show this type of phasing pattern on the precursors of miR159a, miR169 m, miR319a/b, miR447, miR822 and miR839. It is important to note that more than one such miRNA-like RNA may appear in a fold-back structure. In total, using a minimum of 5 sequencing reads as a cutoff, we identified 35 miRNA-like RNAs from 19 miRNA precursors in 10 *Arabidopsis *miRNA families, including both evolutionarily conserved and young non-conserved miRNAs (Table 1). The sequences of the 35 newly identified small RNAs were also blasted against the *Pst *DC3000 genome, and no homologue with > 30% identity was found for any of them. This result means that at least 9.1% (19 of 207) of the known *Arabidopsis *miRNA precursors can produce this type of small RNA. Table 1 lists these miRNA precursors and the corresponding miRNA-like RNAs identified in our small RNA sequence libraries. As shown in the table, one precursor (that is, pre-miR822) can generate as many as ten distinct miRNA-like RNAs (with seven having more than five reads) from both sides of the stem-loop structure (Figure 3a). Additional file 2 displays the alignment of sequencing reads to these precursors, and Table 1 includes the numbers of sequencing reads of these miRNA-like RNAs.

**Table 1:** Nineteen *Arabidopsis *miRNA precursors from 10 miRNA families generate a total of 35 miRNA-sibling RNAs

*MIR*	Loci	miRNA ID	miRNA sequence	Reads	AGO	PARE
*miR159a*	+	miR159a.1*	GAGCTCCTTAAAGTTCAAACA	9		
		**miR159a.2-5p**	**AGCTGCTAAGCTATGGATCCC**	36	2,7	
		**miR159a.3**	**TAAAAAAGGATTTGGTTATA**	6		
		**miR159a.2-3p**	**ATTGCATATCTCAGGAGCTTT**	9	1,2,7	At5g24620
		miR159a.1*	TTTGGATTGAAGGGAGCTCTA	6,587		
*miR519b*	+	**miR159b.2**	**AGCTGCTAAGCTATGGATCCC**	36	7	
		miR159b.2*	**ATGCCATATCTCAGGAGCTTT**	14	1,2,7	
		miR159b.1	TTTGGATTGAAGGGAGCTC *	2481		
*miR168a*	+	miR168a.1	TCGCTTGGTGCAGGTCGGGAA	266,020		
		**miR168a.2**	**ATTGGTTTGTGAGCAGGGATTGGAT**	10	2	
		miR168a.1*	CCCGCCTTGCATCAACTGAAT	1,497		
*miR169b*	-	**miR169b.2**	**TGAAGTGGAGTAGAGTATAATG**	7		At4g17420
		miR169b.1	CAGCCAAGGATGACTTGCCGG	4,444		
		miR169b.1*	GGCAAGTTGTCCTTCGGCTACA	8		
*miR169f*	-	**miR169f.2**	**TGAAGGAATAACGAATGGAAT**	108	1	
		miR169f.1	TGAGCCAAGGATGACTTGCCG	5,779		
		miR169f.1*	GCAAGTTGACCTTGGCTCTGC	2,505		
*miR169i*	-	**miR169i.2-5p**	**TGAATAGAAGAATCATATTTGG**	32		
		miR169i.1	TAGCCAAGGATGACTTGCCTG	44,477		
		miR169i.1*	GGCAGTCTCCTTGGCTATC	360		
		**miR169i.2-3p**	**TTATATGTTCTTCTCTTTCATC**	9		At5g02710
*miR169j*	-	miR169j.1	TAGCCAAGGATGACTTGCCTG	44,458		
		miR169j.1*	AATCTTGCGGGTTAGGTTTCA	9		
		**miR169j.2**	**GGCAGTCTCCTTGGCTATC**	224	4	At5g48300
*miR169l*	-	miR169l.1	TAGCCAAGGATGACTTGCCTG	44,392		
		miR169l.1*	AATCTTGCGGGTTAGGTTTCA	9		
		**miR169l.2**	**AGGCAGTCTCTTTGGCTATC**	366		
*miR169m*	-	**miR169m.2**	**TGAATAGAAGAATCATATTTGG**	32		
		miR169m.1	TAGCCAAGGATGACTTGCCTG	39,650		
		miR169m.1*	GGCAGTCTCCTTGGCTATC	361		
*miR169n*	-	miR169n.2*	**TGGCGGAAAGCGTCATGTTTAG**	10	4	
		miR169n.1	TAGCCAAGGATGACTTGCCTG	44,458		
		miR169n.1*	AATCTTGCGGGTTAGGTTTCA	9		
		**miR169n.2**	**AGGCAGTCTCTTTGGCTATC**	366		
*miR319a*	+	**miR319a.2**	**AATGAATGATGCGGTAGACAAA**	8	1,2,4,5	
		miR319a.1	TTGGACTGAAGGGAGCTCCCT	27		
*miR319b*	+	miR319b.1*	GAGCTTTCTTCGGTCCACTC	28		
		**miR319b.2**	**AATGAATGATGCGAGAGACAA**	491	1,2	
		miR319b.1	TTGGACTGAAGGGAGCTCCCT	30		
*miR447a*	-	**miR447a.2-5p**	**ACCCCTTACAATGTCGAGTAA**	106	2,4,5	
		miR447a.1	TTGGGGACGAGATGTTTTGTTG	198		
		**miR447a.2-3p**	**ACTCGATATAAGAAGGGGCTT**	94	1,2,4,5,7	
		**miR447a.3**	**TATGGAAGAAATTGTAGTATT**	96	1,2,4,5,7	
*miR447b*	-	miR447b.1*	AGTAAACGAAGCATCTGTCCCC	8		
		miR447b.1	TTGGGGACGAGATGTTTTGTTG	198		
		**miR447b.2**	**ACTCGATATAAGAAGGGGCTT**	94	2,5,7	
		**miR447b.3**	**TATGGAAGAAATTGTAGTATT**	96	2,4,7	
*miR775*	+	miR775.1*	GCACTACGTGACATTGAAAC	8		
		**miR775.2**	**TTTGGTTTGTTCAAAGACATT**	10	5	
		miR775.1	TTCGATGTCTAGCAGTGCCA	3,136		
*miR822*	+/-	miR822.2*	**CGACCTTAAGTATAAGTAGAT**	6		
		miR822.3*	**GATGTAACGCATGTTGTTTTCT**	149	2,4,7	
		miR822.1	TGCGGGAAGCATTTGCACATGT	4,153		
		**miR822.4-5p**	**TTTCGTGGAGAATGAAATCAC**	10	1,4	At1g62030, At2g04680
		**miR822.5**	**CATACATGAATAATAATTACC**	9	1,5	
		**miR822.4-3p**	**TATGATTTTATCCTCCATAAAA**	11	5	
		miR822.1*	ATGTGCAAATGCTTTCTACAG	693		
		**miR822.3**	**AAACAATATACGTTGCATCCC**	1,691	1,2,4,7	
		**miR822.2**	**ATCTACTTACACTTAAGGTCG**	363	1,2,4,5	
*miR839*	+/-	**miR839.2**	**TCATGTGAGCAGAAAGAGTAG**	10	1	
		miR839.1	TACCAACCTTTCATCGTTCCC	5		
		**miR839.3**	**TGCAAAACCGTGATAGTGCTGA**	13	1,2,4,7	At1g65960
		miR839.1*	GAACGCATGAGAGGTTGGTAAA	33		
*miR841*	-	**miR841.2**	**TGTTCTTAAGTTGCTTGTGAA**	8	1	
		miR841.1	TACGAGCCACTTGAAACTGAA	59		
		miR841.1*	ATTTCTAGTGGGTCGTATTCA	3,904		
*miR846*	+	miR846.1*	CATTCAAGGACTTCTATTCAG	59		
		**miR846.2**	**AATTGGATATGATAAATGGTAA**	34		
		miR846.2*	**ACTTTTATCATATCCCATCAG**	18		
		miR846.1	TTGAATTGAAGTGCTTGAATT	37		

Nineteen *Arabidopsis *miRNA precursors (the MIR column) from 10 miRNA families generate a total of 35 miRNA-sibling RNAs (the miRNA ID and miRNA sequence columns). For a particular precursor, the positions of newly identified miRNA-like RNAs relative to the cognate miRNA are indicated in the 'Position' column, where a plus sign (+) means that miRNA-sibling small RNAs (msRNAs) are in the upper arm of the hairpin close to the loop, a minus sign (-) indicates that they reside in the lower portion near the base of the hairpin, and '+/-' means that multiple msRNAs appear on both sides of the sibling miRNA, which is also included here. The cognate (known) miRNAs are named as miR*n*.1 (see the main text). The Argonaute proteins that a miRNA-like RNA can associate with are listed in the 'AGO' column. The 'PARE' column lists the mRNA genes for which a miRNA-like RNA has a corresponding small RNA target product in the three PARE/degradome datasets considered.

In the rest of this section, we provide a slew of genomic and molecular evidence to show that many of these miRNA-like RNAs are authentic and functional miRNAs. Following miRNA nomenclature [17,18], we name these miRNA-like RNAs miR*n*.*k*, where integer *n *specifies a particular miRNA family and precursor (for example, 159a for miR159a) and integer *k *denotes a specific miRNA or miRNA-like RNA. To minimize possible confusion, we reserve miR*n*.1 for the known miRNA, and name the newly identified miRNA-like RNAs as miR*n*.2, miR*n*.3, and so on, starting from the 5'-end of the miRNA precursor. Following the notation for miRNA*, the miRNA-like RNA opposite another miRNA-like RNA (miR*n.k*), but with a lower abundance than the latter, is labeled as miRn.k*. However, if the abundances of miR*n.k *and miRn.k* are comparable, they are named as miR*n.k*-5p and miR*n.k*-3p, depending on their relative positions [17,18]. For example, the three miRNA-like RNAs on the miR159a precursor that passed our selection criteria are labeled as miR159a.2-5p, miR159a.3 and miR159a.2-3p, respectively, starting from the 5'-end of the precursor (Figure 1a).

### The identified miRNA-like RNAs are generated by the miRNA biogenesis pathway

The newly identified miRNA-like RNAs and the known miRNAs share several common characteristics. First, an individual miRNA-like RNA often has a pairing partner on the opposite arm of the precursor fold-back structure, which is analogous to the pairing partnership of miRNA and miRNA*. More critically, such pairing partners typically have an approximately two-nucleotide 3'-end overhang, which reflects RNase III activities [39]. For example, miR159a.2-5p is paired with miR159a.2-3p with a two-nucleotide 3'-end overhang (Figure 1a). Similar examples can be found in the other miRNA precursor structures shown in Figures 1, 2, 3, and 4.

Second and more importantly, these miRNA-like RNAs are generated by the same biogenesis pathway as the cognate miRNAs. We experimentally studied some of the miRNA-like RNAs on miR447a and miR822 precursors using various mutants of small RNA pathway components. As shown in Figure 2c, the accumulation of both miR447a (which was renamed as miR447a.1) and miR447a.3 depended on DCL1. The biogenesis of both mature miR822 (that is, miR822.1) and miR822.2 depended on DCL4 (Figure 3c), which is consistent with previously published results [19]. Therefore, miR447a.3 and miR822.2 were generated by the same Dicer-like proteins as their cognate miRNAs. tasiRNAs are endogenous phased siRNAs generated by RDR6 and DCL4 [40]. miR447a.3 and miR822.2 did not require RDR (Figures 2c and 3c), which ruled out the possibility that these phased miRNA-like RNAs might be tasiRNAs. Furthermore, to determine whether these miRNA-like RNAs could be hc-siRNAs, we examined their accumulation in mutants of RDR2, DCL3 and the largest subunits of Pol IV (NRPD1) and Pol V (NRPE1), which are required for hc-siRNA formation and function [8,10,11,15]. As shown in Figures 2c and 3c, the production of miR447a.3 and miR822.2 did not need any RDR proteins, Pol IV, Pol V or DCL3. Therefore, these small RNAs were generated through the miRNA pathway by sequential DCL cleavages on the long hairpin stem regions; they are surely not siRNAs.

**Figure 5:**
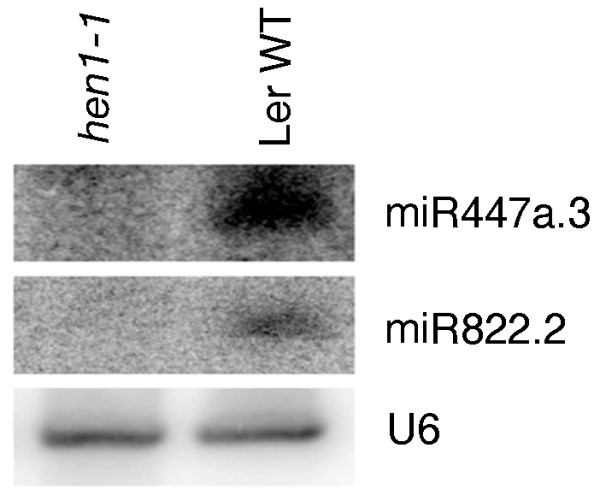
**Accumulation of miR447a.3 and miR822.2 in a mutant of the small-RNA methyltransferase gene *HEN1***. WT, wild type. U6, the control, shows sRNA equal loading.

Third, we examined the effect of HEN1 on these miRNA-like RNAs. In plants, small RNAs, including miRNAs, siRNAs and lsiRNAs, are methylated at their 3'-ends by HEN1 [21,26]. Methylation stabilizes the small RNAs and distinguishes them from RNA degradation products. The accumulation of miR447a.3 and miR822.2 was dependent on HEN1 (Figure 5), indicating that these small RNAs were methylated. Collectively, these results show that these miRNA-like RNAs are produced by the same miRNA pathway as their cognate known miRNAs.

### The identified miRNA-like RNAs are differentially expressed

To investigate the potential functions of the newly identified miRNA-like RNAs in pathogen response, we examined the expression of some of them using Northern blotting. We found that many of the miRNA-like RNAs that we profiled, which have no homologue with identity > 30% in the bacterial genome, were differentially expressed under the challenge of different strains of *Pst*, and exhibited different expression patterns from their cognate miRNAs or miRNA*s (Figures 2d, 3d and 4c). As shown in Figure 2d, for instance, both miR447a.2-3p and miR447a.3 were strongly induced by the avirulent strain *Pst *(*avrRpt2*) and weakly induced by the non-pathogenic strain *Pst *DC3000 *hrcC. *However, the virulent strain *Pst *DC3000 *EV *could induce only miR447a.3 but not miR447a.2-3p. Neither *Pst *DC3000 *EV *nor *Pst *DC3000 *hrcC *induced miR447a (that is, miR447a.1). In addition, miR447a.1 was expressed at a lower level than miR447a.2-3p and miR447a.3. Similarly, miR822.3 was induced by *Pst *DC3000 *EV *and *Pst *(*avrRpt2*) at 6 hpi, and by all three strains tested at 14 hpi, whereas miR822.2 was only induced by *Pst *(*avrRpt2*) at 14 hpi. miR822.3* was barely detected under these conditions (Figure 3d). miR839.2 and miR839.3 were only induced by *Pst *(*avrRpt2*) at 14 hpi and expressed at a very low level under other conditions, whereas miR839.1 was constitutively expressed at a similar level under these conditions (Figure 4c).

The identified miRNA-like RNAs may also be differentially expressed in different tissues. One such example can be seen by comparing the results for the miR839 precursor in Figure 4a with that in Figure 2b of [19]. The peak reads from the deep-sequencing data from [19] also exhibited a phasing pattern, which is in agreement with our deep-sequencing data (Figures 4a,b; Additional file 2). It is important to note that no sequencing read in our small-RNA libraries mapped to gap 2 in Figure 4a, whereas some sequencing reads at gap 2 were shown in Figure 2b in [19]. A major difference between the two deep-sequencing datasets is that total RNA was extracted from whole seedlings, flowers, rosette leaves, and siliques in [19], while we used only matured rosette leaves in our profiling.

As a final note on the expression levels, some of these miRNA-like RNAs can be more abundant than their cognate miRNAs (Table 1). For example, miR319b.2 has 491 reads while miR319b (that is, miR319b.1) has 30 reads (Table 1 and Figure 1d), which is a more than 10-fold difference. Similarly, both miR839.2 and miR839.3 have more reads than miR839 (that is, miR839.1) (Figure 4a). It is possible that some of the miRNA-like RNAs may be induced at certain developmental stages or under specific conditions to regulate gene expression.

### The identified miRNA-like RNAs are potentially functional

We now present three pieces of evidence to show that many of the newly identified miRNA-like RNAs have functional mRNA targets. First, most of these miRNA-like RNAs we identified can be associated with AGO proteins. In general, miRNAs are loaded onto AGO proteins to silence target genes by RNA cleavage, RNA degradation, or translation inhibition. Thus, we searched the *Arabidopsis *datasets of AGO-associated small RNAs [41,42] for the miRNA-like RNAs identified. We found that 25 (71.4%) of the 35 miRNA-like RNAs in 14 (73.7%) of the 19 precursors can be associated with 5 AGO proteins (AGO column in Table 1). In particular, 14, 15, 12, 8 and 11 miRNA-like RNAs can be associated with AGO1, AGO2, AGO4, AGO5 and AGO7, respectively. This result suggests that many of the identified miRNA-like RNAs can potentially function through AGO proteins. Further, the first nucleotide of a small RNA is critical for determining which AGO proteins it may associate with and may consequently dictate its mode of operation [41,42]. The first nucleotides of the unique sequences of the 35 miRNA-like RNAs are preferentially A (45.7% of the total) and U (42.9%), which account for nearly 90% of the total. As a comparison, 75.8% and 12.6% of the known *Arabidopsis *miRNAs start with U and A, respectively. Although the first nucleotides shifted from a preferential U in miRNAs to a nearly equal preference of U and A in the 35 miRNA-like RNAs, U and A are still the two dominant first nucleotides for miRNAs and the miRNA-like RNAs.

**Figure 6:**
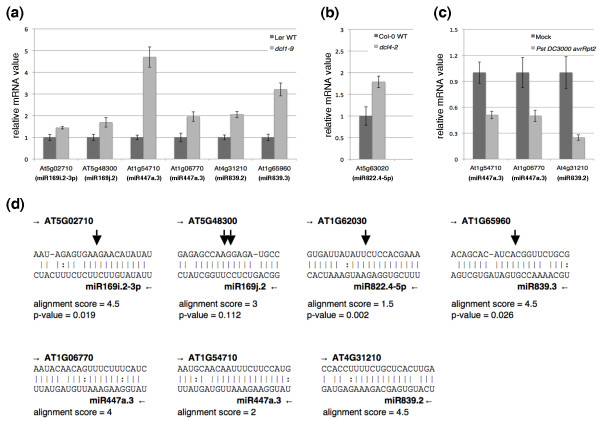
**Negative correlation between the expression of selected miRNA-like RNAs and their targets**. **(a) **The expression of targets of miR169i.2-3p, miR169j.2, miR447a.3, miR839.2 and miR839.3 in a *dcl1-9 *mutant relative to that in the Ler wild type (WT), measured by realtime RT-PCR. **(b) **The expression level of the miR822.4-5p target in a *dcl4-2 *mutant relative to the Col-0 wide type. **(c) **The expression of two miR447a.3 targets and one miR839.2 target under the challenge of *Pst *DC3000 (*avrRpt2*) relative to that in the mock treatment. Actin was used as an internal control for delta Ct calculation. Error bars correspond to standard deviation data from three independent reactions. The experiments were repeated on three sets of biological samples and similar results were obtained. **(d) **Alignments of selected miRNA-like RNAs and some of their mRNA targets whose expression was compared in Dicer mutants and in the wide type (a, b) and under bacterial infection and under mock infection (c). Included are alignment scores and *P*-values of target signatures if miRNA-like RNAs had target degradation products in the three small RNA degradome datasets. The arrows are the target cleavage sites detected in the degradome data.

Second, many of the miRNA-like RNAs identified have putative mRNA targets that have coherent functions. We predicted their putative targets using the target prediction method in version 2 of the CleaveLand software for analyzing small RNA degradomes [43]. With an alignment score cutoff of 4.5, a total of 33 (94.3%) of the 35 miRNA-like RNAs identified have putative targets (Table S2 in Additional file 1). We reasoned that if these miRNA-like RNAs can silence their target genes, de-suppression of the targets might be expected in Dicer mutants, in which the miRNA-like RNAs would no longer be produced. Thus, we examined, using real-time RT-PCR, the expression of some of the predicted targets of miR169i.2-3p (At5g02710), miR169j.2 (At5g48300), miR447a.3 (At1g54710 and At1g06770), miR839.2 (At4g31210), and miR839.3 (At1g65960) in a *dcl1-9 *mutant and in the wild type (Figure 6a), as well as a predicted target of miR822.4-5p (At1g62030) in a *dcl4-2 *mutant and in the wild type (Figure 6b). Indeed, these targets were accumulated to a higher level in the mutants than in the wild type that we studied (Figures 6a,b). Further, because miR447a.3 and miR839.2 were induced by *Pst *(*avrRpt2*), we also examined the expression of their three target genes under the *Pst *(*avrRpt2*) treatment. As shown in Figure 6c, these targets were repressed during *Pst *(*avrRpt2*) challenge, showing a negative correlation with the expression of the corresponding miRNA-like RNAs. Furthermore, similar to most miRNAs, many miRNA-like RNAs identified can target multiple protein-coding genes (Table S2 in Additional file 1). In addition, some of the miRNA-like RNAs may have multiple targets with common or closely related functions. For example, miR775.2 targets two genes in the glycosyl hydrolase family. Different miRNA-like RNAs from the same miRNA precursor may have targets in the same gene family. One pronounced example is the miR822 precursor (Figure 4a). Three miRNA-like RNAs (miR822.3*, miR822.4-5p, and miR822.5), together with their cognate miR822 (miR822.1), can potentially target a total of 60 distinct DC1 domain containing proteins, some of which are targeted by multiple miRNA-like RNAs. Interestingly, miRNA-like RNAs from different miRNA families may also have targets in the same protein family. For example, miR159a.2-3p, miR169j.2, miR319a.2, miR447a.3, miR447b.3, miR822.4-5p, and miR839.2 all have targets in the leucine-rich repeat family. These relationships between the miRNA-like RNAs and their targets are reminiscent of miRNAs and their targets, and also allude to their possible origins of inverted gene duplication [30,44]. In short, our experimental and computational results indicate that the miRNA-like RNAs identified have the potential to silence their target genes, some of which have common or related functions.

Third, some miRNA-like RNAs can mediate target silencing by mRNA cleavage. Since the identified miRNA-like RNAs have the same characteristics as miRNAs and many can be associated with AGO proteins, we hypothesized that they might also directly cleave their mRNA targets. To test this hypothesis, we searched for, using version 2 of the CleaveLand degradome software [43], the small RNA target signatures of mRNA cleavage products in the data from *Arabidopsis *PARE or small RNA degradomes collected by three labs from different tissues and under various conditions [36-38]. With an alignment-score cutoff of 4.5 and a *P*-value threshold of 0.2, we found small RNA cleavage products of seven mRNA genes targeted by six miRNA-like RNAs that we identified (miR159a.2-3p, miR169b.2, miR169i.2-3p, miR169j.2, miR822.4-5p, miR839.3; the PARE column in Table 1). Detailed information on these miRNA-like RNAs and their targets supported by the degradome data is in Table S3 in Additional file 1; the alignments of four of these pairs of miRNA-like RNAs and targets, along with another three pairs tested, are shown in Figure 6d. Furthermore, four of these six miRNA-like RNAs (miR159a.2-3p, miR169j.2, miR822.4-5p, and miR839.3) can also be associated with AGO proteins (Table 1), indicating that, mechanistically, these small RNAs can function through the canonical miRNA pathway. Indeed, the ablation of three of the six miRNA-like RNAs (miR169i.2-3p, miR169j.2, and miR839.3) in the *dcl1-9 *mutant as well as miR822.4-5p in the *dcl4-2 *mutant led to elevated expression of some of their targets (Figures 6a,b). The relatively small number of the miRNA-like RNAs that have mRNA cleavage products may be due to two reasons. First, the miRNA-like RNAs were typically expressed at low abundance; thus, their cleavage products were too low to be detected. Second, different tissues were used in our experiments (mature leaves) and for PARE data collection (floral tissues, including the inflorescence meristem and early stage floral buds, and *EIN5 *mutant). This tissue difference may also explain that no target cleavage product was detected even for four known miRNAs listed in Table 1 (miR447a.1/b.1, miR822.1 and miR839.1) while the expression of miRNAs and miRNA-like RNAs is often tissue-specific. Nevertheless, this degradome analysis provided evidence that some of the miRNA-like RNAs identified in our experiments can function through mRNA target cleavage.

### Distribution of the miRNA-like RNAs on precursor fold-back structures

A remarkable characteristic of the miRNA-like RNAs that we found in *Arabidopsis *is that they can appear on either side of a known miRNA-miRNA* duplex on a precursor hairpin and can be close to either the base or the loop of the hairpin. Two or more miRNA-like RNAs can also reside on both sides of a miRNA-miRNA* duplex. A summary of the location distribution of the miRNA-like RNAs is given in the 'Position' column of Table 1, where a plus sign (+) means that miRNA-like RNAs appear exclusively between miRNA-miRNA* and the loop of the hairpin, a minus sign (-) indicates that miRNA-like RNAs occur exclusively between miRNA-miRNA* and the base of the hairpin, and '+/-' means that there are miRNA-like RNAs on both sides of the miRNA-miRNA* duplex. As shown, among the 19 miRNA precursors identified, 7 harbored miRNA-like RNAs exclusively toward the loops of the hairpins. Examples include *MIR159a*, *MIR319a *and *MIR319b *in Figures 1a,c,d, respectively. This is consistent with the recent discovery that the DCL cleavage that produces mature miR159 and miR319 starts from the loop ends of their fold-back structures [45,46]. Another ten precursors produced miRNA-like RNAs near the hairpin bases - for example, *MIR169m *and *MIR447a *in Figures 1b and 2a. This miRNA-like RNA distribution is well supported by the conventional model of miRNA biogenesis [3,14], in which two subsequent DCL cleavage activities produce a miRNA-miRNA* duplex, first releasing the precursor miRNA and then liberating the duplex. The remaining two precursors - that is, *MIR822 *and *MIR839 *in Figures 3a and 4a - had miRNA-like RNAs on both sides of their cognate miRNA-miRNA* duplexes. These two precursors contain long stems, which are likely to be processed by continuous in-phase dicing activity of DCL1 or DCL4. This process is similar to the biogenesis of tasiRNAs by DCL4.

Another interesting observation is that not every slot in the phasing pattern of a precursor was filled by sequencing reads, as shown in *MIR319b *and *MIR839 *(Figures 1d and 4a). Such a peculiar pattern of miRNA-like RNA expression suggests potential condition- or tissue-specific expression, and variable metabolism rates of these miRNA-like RNAs.

### Conservation of sequential DCL cleavage of long MIR hairpins in eukaryotes

To determine whether the miRNA-like RNAs that we identified in *Arabidopsis *also exist in other plant species, we studied four additional plant organisms. Specifically, we searched for miRNA precursors that are capable of generating multiple miRNA-like RNAs in publicly available small-RNA deep-sequencing datasets from *O. sativa *(rice), *P. patens *(moss), *M. truncatula *and *P. trichocarpa*; see Materials and methods for information on the sources of these datasets. In total, we identified 75, 37, 9 and 11 miRNA precursors, respectively, from these four plant species that harbor miRNA-like RNAs in addition to miRNAs. Table 2 lists these miRNA precursors and Additional files 3, 4, 5 and 6 contain the alignments of the miRNA-like RNAs to their corresponding miRNA precursors. Note that the sequencing data on rice and moss were from Solexa sequencing, while the data on *Medicago *and *Populus *were from a mixture of Solexa and 454 sequencing. Therefore, the sequencing depths for *Medicago *and *Populus *were not as deep as those for rice and moss, resulting in fewer miRNA-like RNA-bearing miRNA precursors identified in the latter two species.

**Table 2:** miRNA precursors of miRNA-like RNA and miRNA families in plants and animals

Species	Number of precursors	Number of families	miRNA precursors
*Arabidopsis*	19	10	**159a/b**, 168a, **169**b/f/i/j/**l**/m/n, **319a/b**, 447a/b, 775, 822, 839, 841, 846
Rice	75	42	156i, **159a/b/c**/d/e/f, 169a, 171i, **319a/b**, 396e/f, 437, 441b, 444b/c/d, 445a/c/d/e/g/h/i, 806h, 807a, 809e/f, 812h/i/j, 820a/b/c, 821a/b/c, 1318, 1319, 1423b, 1427, 1428e/g, 1430, 1432, 1439, 1440, 1441, 1442, 1850, 1851, 1858b, 1862b/d, 1864, 1871, 1874, 1875, 1881, 1883b, 1884a/b, 2121b, 2122, 2123a/b, 2124a/b/c/e/f/g/h/i
Moss	37	27	160i, **319a/b/c**/e, 390a/b, 408b, 533a, 534a, 535d, 536d, 538a/c, 893, 896, 898b, 899, 902k, 904a/b, 1023a, 1030a/f, 1047, 1051, 1063b/d/g, 1068, 1069, 1070, 1072, 1219a/d, 2079, 2083
*Medicago*	9	7	**159a**, **169**d/**l**, **319a/b**, 1510b, 2087, 2088, 2089
*Populus*	11	5	**159a/b/c**, **319c**/d/f/g, 394a/b, 482, 1450
Human	14	14	**7-1**, 15b, 34a, 124-2, 181a-2, 187, 193a, 204, 221, 331, 498, 518c, 769, 1977
Mouse	27	21	**7a-1**, 20a, 24-2, 30b, 96, 101b, 138-1, 328, 329, 331, 337, 341, 434, 466l, 669a-1/-2/-3, 669b/e/m-1/m-2, 674, 685, 708, 1199, 2134-3
*C. elegans*	17	16	51, 60, 61, 63, 64, 70, 75, 78, 80, 232, 244, 257, 258-1/-2, 353, 354, 1817
*Drosophila*	9	9	33, 277, 279, 283, 964, 976, 988, 997, 999

miRNA precursors of miRNA-like RNA and miRNA families in five plant species (*Arabidopsis*, rice, moss, *Medicago *and *Populus*) and four animal organisms (human, mouse, *C. elegans *and *Drosophila*). Entries in bold are miRNA precursors that produce miRNA-like RNAs in more than one species.

As shown in Table 2, several individual miRNA-like RNAs identified are conserved in multiple plants. In the five plant species we studied, miRNA-like RNAs appeared in two well-conserved miRNA families, that is, miR159 and miR319 (Table 2). miRNA-like RNAs appeared in miR319 precursors in all of these five plants, and miRNA-like RNAs occurred in miR159 precursors in all of five bar moss. miR159 and miR319 belong to the same *MIR *family based on their evolutionary origin [30,44], playing important roles in plant development [47]. Importantly, a close inspection showed that many individual miRNA-like RNAs on the miR159 and miR319 precursors are also highly conserved at the sequence level. Figure 6a displays the miR159a precursors in *Arabidopsis*, rice, *Medicago *and *Populus *and Figure 6b shows the miR319b precursors in *Arabidopsis*, rice, moss and *Medicago*. As shown, several miRNA-like RNAs, particularly those expressed at a relatively abundant level, are more conserved than their flanking sequences except their cognate miRNAs or miRNA*s. For example, the sequences of miR319b.2 and miR319b.2* have a comparable level of conservation to miR319b* (that is, miR319b.1*); specifically, miR319a, miR319a*, miR319b.2, and miR319b.2* have 19, 12, 12 and 11 identical nucleotides across the four species, respectively. Furthermore, most of these miRNA-like RNAs, including miR319b.2, miR159a.2-5p, and miR159a.2-3p, can be incorporated into multiple AGO proteins in *Arabidopsis *(Table 1). We also examined the target conservation of the miRNA-like RNAs on miR159a and miR319b precursors across *Arabidopsis*, a dicotyledonous plant, and rice, a monocotyledonous plant. Both ath-miR159a.2-3p and osa-miR159a.2-3p have targets in the pentatricopeptide repeat (PPR) protein family (*At2G40720*, *Os2G05720*, *Os10G40920 *and, *Os5G50690*). Collectively, all these results suggest that these highly conserved miRNA-like RNAs in plants are functional.

**Figure 7:**
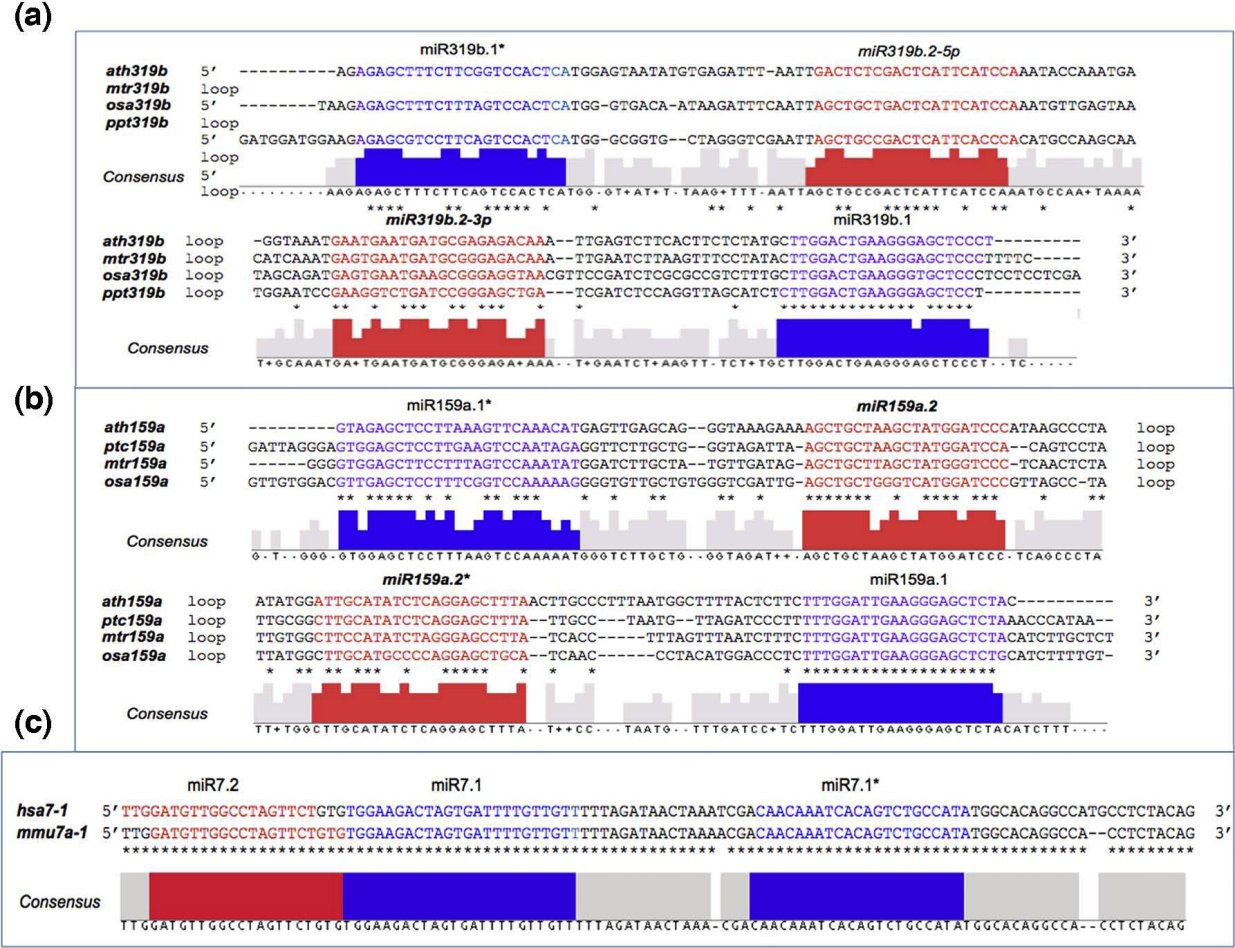
**Conservation of miRNA-like RNAs and miRNAs**. The newly identified miRNA-like RNAs are in red and the annotated miRNAs and miRNA*s are in blue. Also included are the consensus sequences. **(a) **Conservation of miR159a.2-5p/miR159a.2-3p and miR159a.1/miR159a.1* (that is, miR159a/miR159a*) in four plants, *A. thaliana *(ath), *O. sativa *(osa), *P. patens *(ppt) and *M. truncatula *(mtr). **(b) **Conservation of miR319.2/miR319b.2* and miR319.1/miR319b.1* (that is, miR319/miR319b*) in four plants, *Arabidopsis*, rice, *Medicago *and *P. trichocarpa *(ptc). **(c) **Conservation of miR7-1 in human and miR7a-1 in mouse.

To further understand such miRNA-like RNAs, we searched for miRNA-like RNAs in a large collection of small-RNA deep sequencing data from four animal species, *H. sapiens *(human), *M. musculus *(mouse), *C. elegans *and *D. melanogaster *(see Materials and methods). We identified 14, 27, 17 and 9 miRNA precursors in human, mouse, *C. elegans *and *Drosophila*, respectively, that can produce miRNA-like RNAs in addition to the known miRNAs and miRNA*s (Table 2). These miRNA-like RNA-bearing miRNA precursors are distributed in intergenic regions and introns, except has-miR34a and cel-miR354, which reside in 3' untranslated regions. These miRNA-like RNAs are immediately adjacent to the annotated miRNA-miRNA* duplexes, as shown by the alignments to their originating precursor hairpins in the four animals in Additional files 7, 8, 9, and 10, respectively. Different from the results for plants, no more than one small RNA pairing duplex was present beyond the known miRNA-miRNA* duplex in each animal miRNA precursor identified. This difference between plants and animals is mainly due to the relatively short miRNA precursors in animals. The newly identified miRNA-like RNAs in animals are also near the precursor bases. Notice that only miRNA-like RNAs on miR7 precursors in human and mouse are conserved (Table 2; Figure 7c; Additional files 7 and 8). It is also interesting to note that miR7 in *Drosophila *has diverged from that in human and mouse significantly (data not shown), and miR7 has not been reported in *C. elegans*. These results indicate a low conservation of miRNA-like RNAs based on the animal small-RNA sequencing data we examined.

## Discussion

We reported 19 miRNA precursors in *Arabidopsis *that are able to produce multiple distinct miRNA-like RNAs with potential function. Our analysis of the 13 libraries of deep-sequencing data from *Arabidopsis *characterized several important features of these miRNA-like RNAs. First, two miRNA-like RNAs on opposite arms of a miRNA precursor hairpin usually form a duplex with a two-nucleotide 3'-end overhang, which is a key property of miRNA-miRNA* duplexes and reflects the activities of RNase III proteins [39]. Second, such miRNA-like RNAs are arranged in phase, again reflecting sequential dicing activities of some RNase III proteins. Third, the first nucleotide of the discovered miRNA-like RNAs has a strong preference (approximately 90%) for A and U, which is similar to the approximately 88% for all known miRNAs.

Moreover, we obtained several lines of molecular evidence to support the notion that some of the miRNA-like RNAs identified are authentic and potentially functional miRNAs. First, these miRNA-like RNAs are generated through the miRNA pathway, but not the pathways for tasiRNAs or hc-siRNAs (Figures 2 and 3). Second, these miRNA-like RNAs are likely methylated (Figure 5). Third, 25 (71.4%) of the 35 miRNA-like RNAs identified were found in the pools of AGO-associated small RNAs (Table 1) [41,42], suggesting that they may potentially function through the AGO effectors. Fourth, several of these miRNA-like RNAs can induce target mRNA cleavage, and the cleavage products were present in the data from a PARE database or small-RNA degradome (Figure 6) [36-38]. Fifth, most of the miRNA-like RNAs identified have predicted targets, and many of them potentially target genes within the same gene family or with common functions (Table S2 in Additional file 1), which is similar to miRNAs. Sixth, a few of the miRNA-like RNAs are more abundant than cognate miRNAs (Figures 1 and 4), and some of the miRNA-like RNAs are differentially expressed under pathogen infections (Figures 2, 3 and 4), suggesting their potential regulatory functions in response to environmental stresses. The results of gene expression analysis using *dcl1 *and *dcl4 *mutants and wild type *Arabidopsis *plants showed that the loss of miR169i.2-3p, miR169j.2, miR447a.3, miR839.2, miR839.3, and miR822.4-5p can elevate the expression of the predicted target mRNAs that we tested (Figure 6). In addition, miR447a.3 and miR839.2 were induced by *Pst *(*avrRpt2*), and we also detected down-regulation of their targets after *Pst *(*avrRpt2*) challenge (Figure 6).

Moreover, such miRNA-like RNAs exist in five evolutionarily distant plant species as well as four animal organisms we examined - *Arabidopsis*, rice, moss, *Medicago *and *Populus *as well as human, mouse, *C. elegans *and *Drosophila*. This result suggests that the mechanism underlying these miRNA-like RNAs must be conserved within and across plants and animals. The fact that these miRNA-like RNAs appear in moss, an ancient land plant, alludes to their possible evolutionary origin in plants. Some of these miRNA-like RNAs from conserved miRNA families, that is, miR159 and miR319 in plants, are conserved at the sequence level (Figure 7), which adds another layer of evidence that these miRNA-like RNAs are potentially functionally important in plants. In addition, the miRNA-like RNAs we identified appear to occur in evolutionarily 'old' miRNA precursors in both plants and animals based on the publicly available small-RNA sequencing data we examined.

We did not observe a clear correlation between deep sequencing read counts and Northern blot results in *Arabidopsis*, even for a couple of highly expressed known miRNAs. Similar observations were also made in some recent studies [48,49]. The inconsistency between sequencing read counts and Northern blot results is likely due to the differences between the two techniques. Sample preparation for deep sequencing involves two steps of RNA adaptor ligation and one step of PCR amplification, which introduce bias to the final data. Small RNAs with different 5'- and 3'-end structures or modification may have different efficiencies for ligation, and PCR tends to amplify highly abundant sequences more efficiently than less abundant ones. On the other hand, hybridization-based Northern blotting can hybridize any small RNAs with the same sequence regardless of their end modification or structure, although it may have a cross-hybridization side effect depending on the stringency of the hybridization conditions. In light of these observations, we thus relied on conventional Northern blot analysis with high stringency conditions to quantify the expression of the miRNA-like RNAs we studied.

Some early studies, including those using deep sequencing, have also observed small RNAs beyond miRNAs or miRNA*s on some miRNA precursors in plants [19,28-30] and animals [31,32]. For example, sequencing reads of miRNA-like RNAs were found on the ath-miR839 precursor [19]. Interestingly, such miRNA-like RNAs have also been found in a mirtron in mouse [50]. However, none of these small RNAs in plants and animals have been analyzed with regard to their biogenesis or potential functions. Chiang *et al*. [50] argued that their single discovery of a mirtron precursor in mouse was not a coincidence of spliceosome activity releasing the particular mirtron precursor. Indeed, our extensive data on *MIR *genes in four animal and five plant species convincingly show that continuous sequential cleavage activities are a common action for Dicer proteins in animal species or Dicer-like proteins in plant organisms. Another line of related work covers the moRNAs in a chordate, human and a herpesvirus [33-35]. Some of the miRNA-like RNAs that are immediately adjacent to miRNA or miRNA* and located near the base of the miRNA precursors may be classified as moRNAs. However, the moRNAs reported for the chordate *Ciona intestinalis *may be fundamentally different from the miRNA-like RNAs in the four animal genomes that we studied. In particular, the miRNA-like RNAs we identified are within the miRNA precursor sequences within the double-stranded RNA regions, and the ones we tested in *Arabidopsis *are dependent on DCL1 or DCL4. In contrast, many moRNAs identified in *C. intestinalis *- for example, those adjacent to ci-miR124-1 and ci-miR124-2 shown in Figure 2b of [33,34] - can span substantially beyond the double-stranded stem region and even outside the miRNA precursors, which cannot be recognized and processed by Dicer. Thus, although moRNAs are likely to be cut by Drosha at one end, how the other end of moRNAs is generated has yet to be determined [33-35]. More work is required to better understand moRNAs in animals, particularly their biogenesis and functions. In comparison, our study showed that the miRNA-like RNAs in *Arabidopsis *are produced by sequential DCL cleavage on the long hairpin stem regions and can be generated on both sides of the miRNA-miRNA* duplex. Many of them can be incorporated into AGO proteins, and mediate target mRNA cleavage. Some of them are differentially expressed in response to environmental stresses. All of these results suggested their potential biological functions.

## Conclusions

To sum up the results from our in-depth genomic and molecular analysis on *Arabidopsis*, our results from an extensive survey of an additional four plant and four animal species, and some data from early studies on plants [19,28-30] and animals [31,32], it is evident that continuously sequential cleavage by Dicer or Dicer-like proteins is a common theme, rather than an exception, in plants and animals, which gives rise to phased miRNA-like RNAs from *MIR *genes. In *Arabidopsis*, DCL4 has been shown to be capable of such sequential cleavage to generate phased small RNAs such as tasiRNAs [8]. Our results suggest that DCL1, which generates most miRNAs and miRNA-like RNAs, may also act through sequential cleavage as DCL4 does. Further, a limited conservation of miRNA-like RNAs in evolutionarily 'old' *MIR *genes indicates that they are subject to evolutionary selection. The resulting small RNAs may be subject to different rates of degradation. Which miRNAs or miRNA-like RNAs can accumulate in response to developmental and/or environmental cues may be determined by their conservation and through tight regulation by post-biogenesis protection or degradation. For example, these miRNAs or miRNA-like RNAs can be stabilized by binding with AGO proteins or other RNA-binding proteins, or can be degraded by some exoribonucleases [51].

In summary, our results from extensive molecular experiments on *Arabidopsis *and a systematic examination of a large quantity of deep-sequencing datasets from five evolutionarily diverse plant species and four evolutionarily distant animal organisms suggest that the multiple distinct miRNA-like RNAs that we identified broadly exist in eukaryotic species and can be authentic and functional miRNAs. Our results further suggest that the pool of miRNAs is larger than was previously recognized, and miRNA-mediated gene regulation may be broader and more complex than previously thought.

## Materials and methods

### Plant materials, small-RNA library construction, and deep sequencing

Thirteen small-RNA libraries were prepared from 4- to 5-week-old short-day grown *Arabidopsis *Col-0 inoculated with 10 mM MgCl_2 _(mock), type III secretion system mutated strain *Pst *DC3000 *hrcC*, virulent strain *Pst *DC3000, and avirulent strain *Pst *DC3000 (*avrRpt2*). Bacteria infiltration was carried out on the *Arabidopsis *wild-type and mutant plants as described previously [24]. Infiltrated leaves were harvested at 6 and 14 hpi. A large number of *Arabidopsis *mutants (*dcl1-7*, *dcl1-9*, *dcl2-1*, *dcl3-1*, *dcl4-2*, *hen1*, *rdr2-1*, *rdr1-1*, *rdr6-15*, *nrpd1-3*, *nrpe1-11*) and their corresponding wild-type ecotypes were used in this study. Assays were performed on 4-week-old plants grown at 22°C with 12 h light.

Total RNA was isolated using Trizol reagent (Invitrogen, Carlsbad, CA, USA) from infiltrated leaves and fractionated on 15% denaturing polyacrylamide gel. RNA molecules ranging from 18 to 28 nucleotidess were excised and ligated to 5'- and 3'-RNA adaptors using T4 RNA ligase followed by RT-PCR and gel purification as described in the instructions from Illumina [52,53]. The small RNA libraries were sequenced by Illumina Inc. and the University of California, Riverside (UCR) core facility.

### Processing of deep sequencing data

Raw sequence reads were parsed to remove the 3' adaptors. The sequencing reads from each of the small RNA libraries, with adaptors trimmed, were mapped to the *Arabidopsis *nuclear, chloroplast and mitochondrial genome sequences and cDNA sequences, which were all retrieved from the TAIR database [54]. The reads that matched these sequences with no mismatches (the row labeled '*mapped*' in Table S1 in Additional file 1) were retained for further analysis. Sequencing reads were aligned to the precursors of the annotated *Arabidopsis *miRNAs in miRBase [55] with Novealign [56]. Those sequencing reads that could be mapped to a miRNA precursor with zero mismatches were retained for further analysis.

### Small RNA deep-sequencing data on four additional plant and four animal species

We collected small-RNA deep-sequencing data, generated by Illumina/Solexa or 454 sequencing platforms, for four additional plants from the GEO database. In particular, we analyzed a total of 18 small-RNA sequencing datasets on *P. patens *(moss) [57]; six datasets by Solexa sequencing), *O. sativa *(rice [25]; and another two datasets, GEO accession number [GEO:GSE14462]; all by Solexa sequencing), *M. truncatula *(two datasets from Solexa sequencing [58] and three datasets from 454 sequencing [59], and *P. trichocarpa *(four datasets from 454 sequencing [60,61]). Small RNA deep sequencing data were also collected for four animal species: *H. sapiens *(human [62,63]; four datasets); *M. musculus *(mouse [32]; three datasets); *C. elegans *(*C. elegans *[64]; nine datasets) and *D. melanogaster *(*Drosophila *[16]; five datasets). The initial processing of these sequencing libraries followed the same steps as for the 13 *Arabidopsis *datasets. In summary, we collected, processed and analyzed a total of 52 small RNA deep-sequencing datasets in the current study.

### Identifying miRNA-like RNAs and determining their phasing patterns

We first mapped sequence reads, allowing no mismatch, to miRNA precursors. We retained those that have reads arranged in block patterns for further analysis. To this end, we applied the Blockbluster software [33,34] to first identify blocks of sequencing reads on the annotated miRNA precursors. The most abundant sequence read within each detected block was taken as the representative sequence for the block. The total number of sequence reads for the block is the sum of the copy number of the representative read and the copy numbers of other sequence reads that fall within the representative sequence and that overlap with the representative read with no more than three nucleotides beyond the representative sequence on either end. We allowed such overhangs to tolerate imprecise dicing activities of RNase III enzymes. The remaining blocks were further inspected; those spanning across two neighboring blocks were ignored. Those miRNA precursors that have blocks of reads arranged in phase were kept for further analysis. When reporting the results using our *Arabidopsis *deep sequencing data, we ignored such blocks that have less than five total sequencing reads to reduce potential false positive miRNA-like RNAs due to sequencing error.

### Northern blot analysis of small RNAs

Total RNA was isolated using Trizol reagent (Invitrogen) from infiltrated leaves and 80 μg was resolved on 15% denaturing polyacrylamide gel. RNA was cross-linked to membrane using EDC (1-ethyl-3-(3-dimethylaminopropyl) carbodiimide (N-(3-Dimethylaminopropyl)-N'-ethylcarbodiimide hydrochloride (Sigma, St. Louis, MO, USA))) [65]. We used the locked nucleic acid (LNA) probe for small RNA Northern blot analysis to detect miR839, and DNA oligos for detecting miR447 and miR822. Blots were exposed to phosphorscreens, and scanned using PhosphorImager (Molecular Dynamics (Amersham Pharmacia Biotech Inc, Piscataway, NJ, USA)).

The LNA probes used for detection were: mirt839-AT1G67481.2, c+tac+tct+ttc+tgc+tca+cat+ga; mirt839-AT1G67481.1, g+gga+acg+atg+aaa+ggt+tgg+ta; and mirt839-AT1G67481.3, t+cag+cac+tat+cac+ggt+ttt+gca+aa.

We used the following oligo probes for miR447 and miR822: miR447a.1-anti, CAACAAAACATCTCGTCCCCAA; miR447a.2-3p-anti, AAGCCCCTTCTTATATCGAGTC; miR447a.3-anti, AATACTACAATTTCTTCCATA; miR822.1-anti, CATGTGCAAATGCTTCCCGCA; miR822.1*-anti, TGTAGAAAGCATTTGCACATG; miR822.3*-anti, AGAAAACAACATGCGTTACATCC; miR822.3-anti, GGGATGCAACGTATATTGTTTC; miR822.2-anti, ACGACCTTAAGTGTAAGTAGAT.

### miRNA target cleavage product analysis and target prediction

We applied version 2 of the CleaveLand software [43] to the data from PARE analysis or small RNA degradomes [36-38] to characterize the signatures of mRNA cleavage products of the miRNA-like RNAs we studied. We only considered the target cleavage products with an alignment score from CleaveLand of no less than the cutoff threshold of 4.5. Putative targets of miRNA-like RNAs were also predicted by the miRNA target finding method in the CleaveLand software package. This target finding method used a scoring scheme that charges a penalty of one to a mismatch and a penalty of 0.5 to a wobble base pairing, and doubles these penalties in the seed region, which is the two- to seven-nucleotide region near the 5'end of a miRNA.

### Quantitative RT-PCR analysis of small RNA targets

For quantitative RT-PCR analysis, 5 μg of total RNA was used for synthesizing cDNA. DNA contamination was removed by using DNase I (Invitrogen). Amplification of miRNA 169f and miR447f targets (see AT numbers below) was carried out using a realtime PCR machine (iQ5, Bio-Rad, Hercules, CA, USA). The following oligos were used to perform the RT-PCR analysis: At5g02710(miR169i.2-3p)-QRTF, TCGACAACCCTGATGATGTTGAGCTG; At5g02710(miR169i.2-3p)-QRTR, CCTTGTTGAATTTCTTTTCTTCAATCC; At5g48300(miR169j.2)-QRTF, AATGGGAGCTGATTATTACGAGACTGC; At5g48300(miR169j.2)-QRTR, CTTGCACGTTGTCGCTGTTTATGATC; At1g54710(miR447a.3)-QrtF, GGTATATGATAATTTTCACAGTGTGTATACCAG; At1g54710(miR447a.3)-QrtR, ATCTTCGGGGTAAACCATACCATTGAAT; At1g06770(miR447a.3)-QrtF, CGAGAGTGAAAATGAGATAGAGAT; At1g06770(miR447a.3)-QrtR, AGACAAGAACCATCGTAAAATCCT; At4g31210(miR839.2)-QrtF, GTTATTCTAAAGTGTGGACCCTATGGGC; At4g31210(miR839.2)-QrtR, CATCTTCAGGGTGGGTTCCCAGTG; At1g65960(miR839.3)-QRTF, TCATTCACTCTCAATTTCTCCAAGGGA; At1g65960(miR839.3)-QRTR, TTCTCTATACCTTCTTTGAGAACCAC; At1g62030(miR822.4-5p)-QRTF, GCCTGGTTCATCTTGGATAAGCTTCTCC; At1g62030(miR822.4-5p)-QRTR, TTGTAGATATTCGCGATGACCAGC.

## Abbreviations

AGO: Argonaute; DCL: Dicer-like protein; EV: empty vector; GEO: Gene Expression Omnibus; hc-siRNA: heterochromatic small interfering RNA; hpi: hours post-inoculation; lsiRNA: long small interfering RNA; MIR: miRNA gene; miRNA: microRNA; moRNA: miRNA-offset RNA; PARE: parallel analysis of RNA ends; Pol: RNA polymerase; RDR: RNA-dependent RNA polymerase; siRNA: small interfering RNA; tasiRNA: *trans*-acting small interfering RNA.

## Authors' contributions

XuefengZ and HJ initiated the research; HJ and WZ designed the experiments; JX, WZ, XiangZ, and XuefengZ carried out the computational experiments and data analyses; SG and PC performed the biological experiments; XiaomingZ participated in the biological experiments; WZ and HJ analyzed and integrated the results, directed and coordinated the research and wrote the paper.

## Supplementary Material

Additional file 1 **Tables S1, S2, and S3**. Table S1: number of sequencing reads from 13 small RNA libraries of *Arabidopsis *seedlings at 6 and 14 hours after three pathogen infections, along with their corresponding mock infection as control. Shown in the table are the total number of reads (*total*), the number of reads that can map to the *Arabidopsis *genome and cDNA sequences (*mapped*), nuclear genome (*nuclear*), transposons and repeats (*repeats/mobile*), and cDNA sequences (*cDNAs*). No mismatch was allowed for the mapping in the table. The second number for each condition (column) is the percent of reads to the *mapped *reads. The sequencing reads are available in the GEO database under accession number [GEO:GSE19694]. Table S2: putative targets of the identified miRNA-like RNAs identified by the miRNA target finding method, developed by Michael Axtell, in the CleaveLand software for small RNA degradome analysis. Results are those with alignment scores above the cutoff threshold of 4.5. Table S3: six miRNA-like RNAs found in the current study and their mRNA targets identified in small RNA degradome data. In the table, the first two columns list the miRNA-like RNAs and their targets, the third to fifth columns list the three major quantitative measures of the results, that is, the alignment scores, the number of raw reads of target degradation products and *P*-values quantifying the enrichment of degradation products, respectively. The last column indicates if a pair of miRNA-like RNA and target was tested in the current study. Click here for file

Additional file 2 **Supplemental File S1**. This is a file for sequencing reads mapped and aligned to miRNA precursors that can produce miRNA-sibling small RNAs (msRNAs) in *A. thaliana *(ath). The sequencing data were collected in the current study and are available in GEO under accession number [GEO:GSE19694]. Click here for file

Additional file 3 **Supplemental File S2**. This is a file for sequencing reads mapped and aligned to miRNA precursors that can produce miRNA-sibling small RNAs (msRNAs) in rice (osa). The sequencing data were obtained from GEO; see Materials and methods for details. Click here for file

Additional file 4 **Supplemental File S3**. This is a file for sequencing reads mapped and aligned to miRNA precursors that can produce miRNA-sibling small RNAs (msRNAs) in moss (ppt). The sequencing data were obtained from GEO; see Materials and methods for details. Click here for file

Additional file 5 **Supplemental File S4**. This is a file for sequencing reads mapped and aligned to miRNA precursors that can produce miRNA-sibling small RNAs (msRNAs) in *Medicago *(mtr). The sequencing data were obtained from GEO; see Materials and methods for details. Click here for file

Additional file 6 **Supplemental File S5**. This is a file for sequencing reads mapped and aligned to miRNA precursors that can produce miRNA-sibling small RNAs (msRNAs) in *Populus *(ptc). The sequencing data were obtained from GEO; see Materials and methods for details. Click here for file

Additional file 7 **Supplemental File S6**. This is a file for sequencing reads mapped and aligned to miRNA precursors that can produce miRNA-sibling small RNAs (msRNAs) in *H. sapiens *(has). The sequencing data were obtained from GEO; see Materials and methods for details. Click here for file

Additional file 8 **Supplemental File S7**. This is a file for sequencing reads mapped and aligned to miRNA precursors that can produce miRNA-sibling small RNAs (msRNAs) in *M. musculus *(mms). The sequencing data were obtained from GEO; see Materials and methods for details. Click here for file

Additional file 9 **Supplemental File S8**. This is a file for sequencing reads mapped and aligned to miRNA precursors that can produce miRNA-sibling small RNAs (msRNAs) in *C. elegans *(cel). The sequencing data were obtained from GEO; see Materials and methods for details. Click here for file

Additional file 10 **Supplemental File S9**. This is a file for sequencing reads mapped and aligned to miRNA precursors that can produce miRNA-sibling small RNAs (msRNAs) in *D. melanogaster *(dme). The sequencing data were obtained from GEO; see Materials and methods for details. Click here for file
