# Supplementary material for: Multiple distinct small RNAs originate from the same microRNA precursors
Source: Genome Biol. 2010 Aug 9;11(8):R81. doi: 10.1186/gb-2010-11-8-r81 (PMC2945783; doi:10.1186/gb-2010-11-8-r81)
Supplement: Additional file 1 — Tables S1, S2, and S3. Table S1: number of sequencing reads from 13 small RNA libraries of Arabidopsis seedlings at 6 and 14 hours after three pathogen infections, along with their corresponding mock infection as control. Shown in the table are the total number of reads (total), the number of reads that can map to the Arabidopsis genome and cDNA sequences (mapped), nuclear genome (nuclear), transposons and repeats (repeats/mobile), and cDNA sequences (cDNAs). No mismatch was allowed for the mapping in the table. The second number for each condition (column) is the percent of reads to the mapped reads. The sequencing reads are available in the GEO database under accession number [GEO:GSE19694]. Table S2: putative targets of the identified miRNA-like RNAs identified by the miRNA target finding method, developed by Michael Axtell, in the CleaveLand software for small RNA degradome analysis. Results are those with alignment scores above the cutoff threshold of 4.5. Table S3: six miRNA-like RNAs found in the current study and their mRNA targets identified in small RNA degradome data. In the table, the first two columns list the miRNA-like RNAs and their targets, the third to fifth columns list the three major quantitative measures of the results, that is, the alignment scores, the number of raw reads of target degradation products and P-values quantifying the enrichment of degradation products, respectively. The last column indicates if a pair of miRNA-like RNA and target was tested in the current study. [file gb-2010-11-8-r81-S1.DOCX]

**Multiple distinct small RNAs originate from the same microRNA precursors**

Weixiong Zhang^1,2,*,†^, Shang Gao^3,*^, Xuefeng Zhou^1^, Jing Xia^1^, Padmanabhan Chellappan^3^,

Xiang Zhou^1^, Xiaoming Zhang^3^ and Hailing Jin^3,†^

^1^Department of Computer Science and Engineering, Washington University in St. Louis, St. Louis, MO 63130, USA

^2^Department of Genetics, Washington University School of Medicine, St. Louis, MO 63110, USA

^3^Department of Plant Pathology and Microbiology, Center for Plant Cell Biology, Institute for Integrative Genome Biology, University of California, Riverside, CA 92521, USA

*Equal contribution.

†Corresponding to:

Weixiong Zhang, Department of Computer Science and Engineering, Washington University in St. Louis, St. Louis, MO 63130, USA; phone: (314)935-8788; fax: (314)935-7302; email: [weixiong.zhang@wustl.edu](mailto:weixiong.zhang@wustl.edu), or

Hailing Jin, Dept of Plant Pathology and Microbiology, Center for Plant Cell Biology, Institute for Integrative Genome Biology, University of California, Riverside, CA 92521, USA; phone: (951)827-7995; fax: (951)827-4294; email: hailing.jin@ucr.edu.

Running title: Multiple sibling miRN-like RNAs

Keywords: miRNAs, miRNA precursors, miRNA-like RNAs, Dicer-like proteins, Argonaute proteins

**Supplemental Table 1**. Number of sequencing reads from 13 small RNA libraries of *Arabidopsis* seedlings at 6 and 14 hours after three pathogen infections, along with their corresponding mock infection as control. Shown in the table are the total number of reads (*total*), the number of reads that can map to the Arabidopsis genome and cDNA sequences (*mapped*), nuclear genome (*nuclear*), transposons and repeats (*repeats/mobile*), and cDNA sequences (*cDNAs*). *No mismatch was allowed for the mapping in the table.* The second number for each condition (column) is the percent of reads to the *mapped* reads. The sequencing reads are available in NCBI/GEO databases under accession number GSE19694.

| **^total reads^** | ***^m_6h^*** | **^(%)^** | ***^ev_6h^*** | **^(%)^** | ***^hrcc_6h^*** | **^(%)^** | ***^R2_6h^*** | **^(%)^** | ***^m_14h^*** | **^(%)^** | ***^ev_14h^*** | **^(%)^** | ***^hrcc_14h^*** | **^(%)^** | ***^R2_14h^*** | **^(%)^** | ***^total^*** | **^(%)^** |
| --- | --- | --- | --- | --- | --- | --- | --- | --- | --- | --- | --- | --- | --- | --- | --- | --- | --- | --- |
| *^total^* | ^6144892^ |  | ^669129^ |  | ^825545^ |  | ^1388083^ |  | ^4720784^ |  | ^947788^ |  | ^4543376^ |  | ^5409472^ |  | ^24649069^ |  |
| *^mapped^* | ^3659110^ |  | ^421563^ |  | ^517008^ |  | ^960302^ |  | ^2393531^ |  | ^611886^ |  | ^2366759^ |  | ^3055779^ |  | ^13985938^ |  |
| *^nuclear^* | ^3067200^ | ^83.8^ | ^366241^ | ^86.9^ | ^434498^ | ^84.0^ | ^862915^ | ^89.9^ | ^2000906^ | ^83.6^ | ^543407^ | ^88.8^ | ^1979846^ | ^83.7^ | ^2541246^ | ^83.2^ | ^11796259^ | ^84.3^ |
| *^repeats/mobile^* | ^1855440^ | ^50.7^ | ^96934^ | ^23.0^ | ^148873^ | ^28.8^ | ^335049^ | ^34.9^ | ^1058982^ | ^44.2^ | ^147238^ | ^24.1^ | ^1176946^ | ^49.7^ | ^1255222^ | ^41.1^ | ^6074684^ | ^43.4^ |
| *^cDNAs^* | ^832098^ | ^22.7^ | ^38695^ | ^9.2^ | ^31436^ | ^6.1^ | ^122151^ | ^12.7^ | ^522649^ | ^21.8^ | ^53779^ | ^8.8^ | ^529451^ | ^22.4^ | ^584006^ | ^19.1^ | ^2714265^ | ^19.4^ |
| **^unique reads^** | ***^m_6h^*** | **^(%)^** | ***^ev_6h^*** | **^(%)^** | ***^hrcc_6h^*** | ***^(%)^*** | ***^R2_6h^*** | **^(%)^** | ***^m_14h^*** | **^(%)^** | ***^ev_14h^*** | **^(%)^** | ***^hrcc_14h^*** | **^(%)^** | ***^R2_14h^*** | **^(%)^** | ***^total^*** | **^(%)^** |
| *^total^* | ^2585154^ |  | ^216608^ |  | ^258620^ |  | ^448235^ |  | ^2366902^ |  | ^345916^ |  | ^2251203^ |  | ^2538266^ |  | ^11010904^ |  |
| *^mapped^* | ^557372^ |  | ^86217^ |  | ^118376^ |  | ^205075^ |  | ^505409^ |  | ^156350^ |  | ^431560^ |  | ^518172^ |  | ^2578531^ |  |
| *^nuclear^* | ^504607^ | ^90.5^ | ^76940^ | ^89.2^ | ^109273^ | ^92.3^ | ^189518^ | ^92.4^ | ^459912^ | ^91.0^ | ^147663^ | ^94.4^ | ^389679^ | ^90.3^ | ^473384^ | ^91.4^ | ^2350976^ | ^91.2^ |
| *^repeats/mobile^* | ^138207^ | ^24.8^ | ^21394^ | ^24.8^ | ^23147^ | ^19.6^ | ^38773^ | ^18.9^ | ^138537^ | ^27.4^ | ^28270^ | ^18.1^ | ^135850^ | ^31.5^ | ^127413^ | ^24.6^ | ^651591^ | ^25.3^ |
| *^cDNAs^* | ^172594^ | ^31.0^ | ^14674^ | ^17.0^ | ^16177^ | ^13.7^ | ^27999^ | ^13.7^ | ^177542^ | ^35.1^ | ^20387^ | ^13.0^ | ^170020^ | ^39.4^ | ^173175^ | ^33.4^ | ^772568^ | ^30.0^ |

**Supplemental Table 2.** Putative targets of the identified miRNA-like RNAs identified by the miRNA target finding method, developed by Michael Axtell, in the CleaveLand software for small RNA degradome analysis. Results are those with alignment scores above the cutoff threshold of 4.5.

| **miRNAs** | **Score** | **Target and annotation** |
| --- | --- | --- |
| miR159a.2 | 4.5 | AT4G06658.1 \| Symbols: \| transposable element gene \| chr4:3852325-3856095 FORWARD |
| miR159a.2 | 4.5 | AT5G61040.1 \| Symbols: \| unknown protein \| chr5:24562333-24564959 REVERSE |
| miR159a.2* | 2.5 | AT2G10430.1 \| Symbols: \| pseudogene, similar to putative reverse transcriptase, blastp match of 34% identity and 1.6e-24 P-value to GP\|27311287\|gb\|AAO00713.1\|\|AC078894 putative reverse transcriptase {Oryza sativa (japonica cultivar-group)} \| chr2:4008789-4011726 REVERSE |
| miR159a.2* | 3 | AT2G28960.1 \| Symbols: \| leucine-rich repeat protein kinase, putative \| chr2:12437914-12442347 REVERSE |
| miR159a.2* | 3 | AT5G24620.1 \| Symbols: \| thaumatin-like protein, putative \| chr5:8430768-8432414 FORWARD |
| miR159a.2* | 3.5 | AT1G07650.1 \| Symbols: \| leucine-rich repeat transmembrane protein kinase, putative \| chr1:2359549-2366561 REVERSE |
| miR159a.2* | 3.5 | AT1G29730.1 \| Symbols: \| ATP binding / kinase/ protein binding / protein kinase/ protein serine/threonine kinase/ protein tyrosine kinase \| chr1:10400564-10405874 REVERSE |
| miR159a.2* | 3.5 | AT1G29740.1 \| Symbols: \| kinase \| chr1:10407379-10412997 REVERSE |
| miR159a.2* | 3.5 | AT1G43630.1 \| Symbols: \| unknown protein \| chr1:16436371-16437755 REVERSE |
| miR159a.2* | 3.5 | AT1G50140.1 \| Symbols: \| ATP binding / ATPase/ nucleoside-triphosphatase/ nucleotide binding \| chr1:18569735-18578702 REVERSE |
| miR159a.2* | 3.5 | AT1G53430.1 \| Symbols: \| leucine-rich repeat family protein / protein kinase family protein \| chr1:19935167-19941088 FORWARD |
| miR159a.2* | 3.5 | AT1G73920.1 \| Symbols: \| lipase family protein \| chr1:27790875-27794914 FORWARD |
| miR159a.2* | 3.5 | AT4G00960.1 \| Symbols: \| protein kinase family protein \| chr4:414361-416180 FORWARD |
| miR159a.2* | 3.5 | AT4G23230.1 \| Symbols: \| protein kinase family protein \| chr4:12157569-12160270 REVERSE |
| miR159a.2* | 3.5 | AT4G30930.1 \| Symbols: NFD1 \| NFD1 (NUCLEAR FUSION DEFECTIVE 1); RNA binding / structural constituent of ribosome \| chr4:15049824-15051665 REVERSE |
| miR159a.2* | 4 | AT2G11530.1 \| Symbols: \| transposable element gene \| chr2:4624739-4626409 FORWARD |
| miR159a.2* | 4 | AT2G20300.1 \| Symbols: ALE2 \| ALE2 (Abnormal Leaf Shape 2); kinase \| chr2:8755925-8760222 REVERSE |
| miR159a.2* | 4 | AT4G17720.1 \| Symbols: \| RNA recognition motif (RRM)-containing protein \| chr4:9862446-9864663 REVERSE |
| miR159a.2* | 4.5 | AT1G11300.1 \| Symbols: \| ATP binding / carbohydrate binding / kinase/ protein kinase/ protein serine/threonine kinase/ protein tyrosine kinase/ sugar binding \| chr1:3794238-3800719 FORWARD |
| miR159a.2* | 4.5 | AT1G80770.1 \| Symbols: PDE318 \| PDE318 (pigment defective 318); GTP binding \| chr1:30355115-30357964 FORWARD |
| miR159a.2* | 4.5 | AT2G32850.1 \| Symbols: \| protein kinase family protein \| chr2:13935186-13938845 REVERSE |
| miR159a.2* | 4.5 | AT2G34880.1 \| Symbols: MEE27 \| MEE27 (maternal effect embryo arrest 27); transcription factor \| chr2:14711880-14716634 REVERSE |
| miR159a.2* | 4.5 | AT2G40720.1 \| Symbols: \| pentatricopeptide (PPR) repeat-containing protein \| chr2:16987269-16989851 FORWARD |
| miR159a.2* | 4.5 | AT3G15730.1 \| Symbols: PLDALPHA1, PLD \| PLDALPHA1 (PHOSPHOLIPASE D ALPHA 1); phospholipase D \| chr3:5330322-5333745 FORWARD |
| miR159a.2* | 4.5 | AT3G42806.1 \| Symbols: \| transposable element gene \| chr3:14914008-14918667 FORWARD |
| miR159a.2* | 4.5 | AT4G08330.1 \| Symbols: \| unknown protein \| chr4:5255120-5256704 REVERSE |
| miR159a.2* | 4.5 | AT4G23140.1 \| Symbols: CRK6 \| CRK6 (CYSTEINE-RICH RLK 6); kinase \| chr4:12121383-12124205 FORWARD |
| miR159a.2* | 4.5 | AT4G30820.1 \| Symbols: \| cyclin-dependent kinase-activating kinase assembly factor-related / CDK-activating kinase assembly factor-related \| chr4:15006489-15008546 FORWARD |
| miR159a.2* | 4.5 | AT4G37330.1 \| Symbols: CYP81D4 \| CYP81D4; electron carrier/ heme binding / iron ion binding / monooxygenase/ oxygen binding \| chr4:17562345-17564596 REVERSE |
| miR159a.2* | 4.5 | AT5G38285.1 \| Symbols: \| transposable element gene \| chr5:15298033-15300708 REVERSE |
| miR159a.2* | 4.5 | AT5G38560.1 \| Symbols: \| protein kinase family protein \| chr5:15439249-15443164 FORWARD |
| miR159a.2* | 4.5 | AT5G49430.1 \| Symbols: \| transducin family protein / WD-40 repeat family protein \| chr5:20037338-20045454 REVERSE |
| miR159a.3 | 3 | AT1G16020.1 \| Symbols: \| unknown protein \| chr1:5498165-5501358 FORWARD |
| miR159a.3 | 3.5 | AT4G00120.1 \| Symbols: IND1, GT140, IND, EDA33 \| IND (INDEHISCENT); DNA binding / transcription factor \| chr4:41665-43197 REVERSE |
| miR159a.3 | 3.5 | AT4G05590.1 \| Symbols: \| unknown protein \| chr4:2907065-2908557 FORWARD |
| miR159a.3 | 3.5 | AT4G06551.1 \| Symbols: \| transposable element gene \| chr4:3443530-3445107 FORWARD |
| miR159a.3 | 3.5 | AT4G12910.1 \| Symbols: scpl20 \| scpl20 (serine carboxypeptidase-like 20); serine-type carboxypeptidase \| chr4:7550433-7553332 REVERSE |
| miR159a.3 | 3.5 | AT5G29015.1 \| Symbols: \| transposable element gene \| chr5:11031699-11038817 FORWARD |
| miR159a.3 | 4 | AT1G50640.1 \| Symbols: ERF3, ATERF3 \| ERF3 (ETHYLENE RESPONSIVE ELEMENT BINDING FACTOR 3); DNA binding / protein binding / transcription factor/ transcription repressor \| chr1:18757148-18758433 REVERSE |
| miR159a.3 | 4 | AT2G22970.1 \| Symbols: SCPL11 \| serine carboxypeptidase S10 family protein \| chr2:9774853-9778528 FORWARD |
| miR159a.3 | 4 | AT2G37230.1 \| Symbols: \| pentatricopeptide (PPR) repeat-containing protein \| chr2:15637046-15639522 REVERSE |
| miR159a.3 | 4 | AT2G41130.1 \| Symbols: \| basic helix-loop-helix (bHLH) family protein \| chr2:17143294-17144949 FORWARD |
| miR159a.3 | 4 | AT3G05910.1 \| Symbols: \| pectinacetylesterase, putative \| chr3:1764294-1767575 REVERSE |
| miR159a.3 | 4 | AT3G14350.1 \| Symbols: SRF7 \| SRF7 (STRUBBELIG-RECEPTOR FAMILY 7); ATP binding / protein binding / protein kinase/ protein serine/threonine kinase/ protein tyrosine kinase \| chr3:4782757-4787236 REVERSE |
| miR159a.3 | 4 | AT3G32022.1 \| Symbols: \| transposable element gene \| chr3:13003010-13007712 REVERSE |
| miR159a.3 | 4 | AT3G42220.1 \| Symbols: \| transposable element gene \| chr3:14380475-14384179 REVERSE |
| miR159a.3 | 4 | AT3G51580.1 \| Symbols: \| unknown protein \| chr3:19130834-19133342 REVERSE |
| miR159a.3 | 4 | AT3G61550.1 \| Symbols: \| zinc finger (C3HC4-type RING finger) family protein \| chr3:22776401-22777225 FORWARD |
| miR159a.3 | 4 | AT4G17565.1 \| Symbols: \| F-box family protein \| chr4:9782508-9783644 REVERSE |
| miR159a.3 | 4 | AT4G25480.1 \| Symbols: DREB1A, CBF3, ATCBF3 \| DREB1A (DEHYDRATION RESPONSE ELEMENT B1A); DNA binding / transcription activator/ transcription factor \| chr4:13018214-13019121 REVERSE |
| miR159a.3 | 4 | AT5G16310.1 \| Symbols: UCH1 \| UCH1; ubiquitin thiolesterase \| chr5:5342391-5344285 REVERSE |
| miR159a.3 | 4 | AT5G47430.1 \| Symbols: \| zinc ion binding \| chr5:19235631-19241221 REVERSE |
| miR159a.3 | 4.5 | AT1G10920.1 \| Symbols: LOV1 \| LOV1; ATP binding \| chr1:3644420-3647768 REVERSE |
| miR159a.3 | 4.5 | AT1G15210.1 \| Symbols: PDR7, ATPDR7 \| PDR7 (PLEIOTROPIC DRUG RESISTANCE 7); ATPase, coupled to transmembrane movement of substances \| chr1:5231348-5236573 REVERSE |
| miR159a.3 | 4.5 | AT1G18900.1 \| Symbols: \| pentatricopeptide (PPR) repeat-containing protein \| chr1:6528984-6532614 FORWARD |
| miR159a.3 | 4.5 | AT1G21920.1 \| Symbols: \| MORN (Membrane Occupation and Recognition Nexus) repeat-containing protein /phosphatidylinositol-4-phosphate 5-kinase-related \| chr1:7704255-7706140 REVERSE |
| miR159a.3 | 4.5 | AT1G39670.1 \| Symbols: \| transposable element gene \| chr1:14817510-14819595 FORWARD |
| miR159a.3 | 4.5 | AT1G63690.1 \| Symbols: \| protease-associated (PA) domain-containing protein \| chr1:23618378-23622396 FORWARD |
| miR159a.3 | 4.5 | AT1G66173.1 \| Symbols: \| other RNA \| chr1:24642586-24644068 FORWARD |
| miR159a.3 | 4.5 | AT2G04760.1 \| Symbols: \| transposable element gene \| chr2:1665780-1668389 FORWARD |
| miR159a.3 | 4.5 | AT2G11670.1 \| Symbols: \| transposable element gene \| chr2:4685014-4687082 FORWARD |
| miR159a.3 | 4.5 | AT2G26830.1 \| Symbols: emb1187 \| emb1187 (embryo defective 1187); kinase/ phosphotransferase, alcohol group as acceptor \| chr2:11443561-11447171 FORWARD |
| miR159a.3 | 4.5 | AT2G32190.1 \| Symbols: \| unknown protein \| chr2:13674498-13675286 FORWARD |
| miR159a.3 | 4.5 | AT3G04380.1 \| Symbols: SUVR4, SDG31 \| SUVR4; histone-lysine N-methyltransferase \| chr3:1161387-1164929 FORWARD |
| miR159a.3 | 4.5 | AT3G15950.1 \| Symbols: NAI2 \| NAI2 \| chr3:5397569-5402652 REVERSE |
| miR159a.3 | 4.5 | AT3G46790.1 \| Symbols: CRR2 \| CRR2 (CHLORORESPIRATORY REDUCTION 2) \| chr3:17231773-17234002 REVERSE |
| miR159a.3 | 4.5 | AT3G55240.1 \| Symbols: \| Overexpression leads to PEL (Pseudo-Etiolation in Light) phenotype. \| chr3:20473541-20474763 REVERSE |
| miR159a.3 | 4.5 | AT3G55480.1 \| Symbols: \| adaptin family protein \| chr3:20566248-20571248 REVERSE |
| miR159a.3 | 4.5 | AT3G57645.1 \| Symbols: U2.2 \| U2.2; snRNA \| chr3:21346342-21347204 FORWARD |
| miR159a.3 | 4.5 | AT3G61160.1 \| Symbols: \| shaggy-related protein kinase beta / ASK-beta (ASK2) \| chr3:22635881-22638817 FORWARD |
| miR159a.3 | 4.5 | AT4G17800.1 \| Symbols: \| DNA-binding protein-related \| chr4:9895290-9896668 REVERSE |
| miR159a.3 | 4.5 | AT4G20880.1 \| Symbols: \| ethylene-responsive nuclear protein / ethylene-regulated nuclear protein (ERT2) \| chr4:11179115-11180799 REVERSE |
| miR159a.3 | 4.5 | AT4G23770.1 \| Symbols: \| unknown protein \| chr4:12383311-12384496 REVERSE |
| miR159a.3 | 4.5 | AT4G30993.1 \| Symbols: \| unknown protein \| chr4:15098067-15100558 FORWARD |
| miR159a.3 | 4.5 | AT5G15030.1 \| Symbols: \| paired amphipathic helix repeat-containing protein \| chr5:4866692-4869133 REVERSE |
| miR159a.3 | 4.5 | AT5G32254.1 \| Symbols: \| transposable element gene \| chr5:11909710-11913389 REVERSE |
| miR159b.2 | 4.5 | AT4G06658.1 \| Symbols: \| transposable element gene \| chr4:3852325-3856095 FORWARD |
| miR159b.2 | 4.5 | AT5G61040.1 \| Symbols: \| unknown protein \| chr5:24562333-24564959 REVERSE |
| miR159b.2* | 3 | AT5G23575.1 \| Symbols: \| transmembrane protein, putative \| chr5:7946310-7950253 FORWARD |
| miR159b.2* | 3.5 | AT2G32850.1 \| Symbols: \| protein kinase family protein \| chr2:13935186-13938845 REVERSE |
| miR159b.2* | 4 | AT1G76270.1 \| Symbols: \| unknown protein \| chr1:28613207-28616739 REVERSE |
| miR159b.2* | 4 | AT4G17895.1 \| Symbols: UBP20 \| UBP20 (UBIQUITIN-SPECIFIC PROTEASE 20); ubiquitin thiolesterase/ ubiquitin-specific protease \| chr4:9939695-9942772 FORWARD |
| miR159b.2* | 4 | AT5G46740.1 \| Symbols: UBP21 \| UBP21 (UBIQUITIN-SPECIFIC PROTEASE 21); ubiquitin thiolesterase/ ubiquitin-specific protease \| chr5:18965271-18968742 REVERSE |
| miR159b.2* | 4.5 | AT1G04780.1 \| Symbols: \| ankyrin repeat family protein \| chr1:1340669-1343584 REVERSE |
| miR159b.2* | 4.5 | AT1G19140.1 \| Symbols: \| FUNCTIONS IN: molecular_function unknown; INVOLVED IN: ubiquinone biosynthetic process; LOCATED IN: mitochondrion; EXPRESSED IN: 24 plant structures; EXPRESSED DURING: 15 growth stages; CONTAINS InterPro DOMAIN/s: COQ9 (InterPro:IPR013718), Ubiquinone biosynthesis protein COQ9 (InterPro:IPR012762); Has 607 Blast hits to 607 proteins in 177 species: Archae - 0; Bacteria - 151; Metazoa - 114; Fungi - 62; Plants - 22; Viruses - 0; Other Eukaryotes - 258 (source: NCBI BLink). \| chr1:6610721-6612516 REVERSE |
| miR159b.2* | 4.5 | AT1G49960.1 \| Symbols: \| xanthine/uracil permease family protein \| chr1:18498639-18501886 FORWARD |
| miR159b.2* | 4.5 | AT1G71000.1 \| Symbols: \| heat shock protein binding \| chr1:26769336-26770111 REVERSE |
| miR159b.2* | 4.5 | AT1G80660.1 \| Symbols: AHA9 \| AHA9; hydrogen-exporting ATPase, phosphorylative mechanism \| chr1:30316227-30319948 REVERSE |
| miR159b.2* | 4.5 | AT2G40720.1 \| Symbols: \| pentatricopeptide (PPR) repeat-containing protein \| chr2:16987269-16989851 FORWARD |
| miR159b.2* | 4.5 | AT3G09190.1 \| Symbols: \| sugar binding \| chr3:2821479-2822687 REVERSE |
| miR159b.2* | 4.5 | AT3G18180.1 \| Symbols: \| transferase, transferring glycosyl groups \| chr3:6230270-6231878 FORWARD |
| miR159b.2* | 4.5 | AT5G28750.1 \| Symbols: \| thylakoid assembly protein, putative \| chr5:10783983-10785722 REVERSE |
| miR159b.2* | 4.5 | ATCG00170.1 \| Symbols: RPOC2 \| RNA polymerase beta' subunit-2 \| chrC:15938-20068 REVERSE |
| miR168a.2 | 4 | AT3G55130.1 \| Symbols: ATWBC19 \| ATWBC19 (White-Brown Complex homolog 19); ATPase, coupled to transmembrane movement of substances \| chr3:20433878-20436390 REVERSE |
| miR168a.2 | 4.5 | AT1G12064.1 \| Symbols: \| unknown protein \| chr1:4077498-4078095 REVERSE |
| miR169b.2 | 3.5 | AT4G17420.1 \| Symbols: \| FUNCTIONS IN: molecular_function unknown; INVOLVED IN: biological_process unknown; LOCATED IN: cellular_component unknown; EXPRESSED IN: 23 plant structures; EXPRESSED DURING: 13 growth stages; CONTAINS InterPro DOMAIN/s: Protein of unknown function DUF124 (InterPro:IPR002838), Tryptophan RNA-binding attenuator protein-like (InterPro:IPR016031); BEST Arabidopsis thaliana protein match is: unknown protein (TAIR:AT5G47420.1); Has 588 Blast hits to 588 proteins in 254 species: Archae - 60; Bacteria - 418; Metazoa - 0; Fungi - 6; Plants - 37; Viruses - 0; Other Eukaryotes - 67 (source: NCBI BLink). \| chr4:9722356-9724640 REVERSE |
| miR169b.2 | 4.5 | AT2G32300.1 \| Symbols: UCC1 \| UCC1 (UCLACYANIN 1); copper ion binding / electron carrier \| chr2:13722427-13723590 FORWARD |
| miR169b.2 | 4.5 | AT2G38320.1 \| Symbols: \| unknown protein \| chr2:16055289-16057980 FORWARD |
| miR169b.2 | 4.5 | AT4G28620.1 \| Symbols: ATATM2, ATM2 \| ATM2 (ABC TRANSPORTER OF THE MITOCHONDRION 2); ATPase, coupled to transmembrane movement of substances / transporter \| chr4:14135526-14137953 REVERSE |
| miR169b.2 | 4.5 | AT5G27925.1 \| Symbols: \| transposable element gene \| chr5:9949686-9954107 FORWARD |
| miR169f.2 | 3.5 | AT2G11740.1 \| Symbols: \| transposable element gene \| chr2:4706901-4708590 REVERSE |
| miR169f.2 | 4 | AT1G22660.1 \| Symbols: \| tRNA-nucleotidyltransferase, putative / tRNA adenylyltransferase, putative \| chr1:8017397-8021712 FORWARD |
| miR169f.2 | 4 | AT1G60960.1 \| Symbols: IRT3 \| IRT3; cation transmembrane transporter/ metal ion transmembrane transporter \| chr1:22445310-22447214 REVERSE |
| miR169f.2 | 4.5 | AT1G04140.1 \| Symbols: \| transducin family protein / WD-40 repeat family protein \| chr1:1075543-1080630 REVERSE |
| miR169f.2 | 4.5 | AT1G08310.1 \| Symbols: \| esterase/lipase/thioesterase family protein \| chr1:2618647-2620452 FORWARD |
| miR169f.2 | 4.5 | AT1G77180.1 \| Symbols: \| chromatin protein family \| chr1:28999618-29002135 REVERSE |
| miR169f.2 | 4.5 | AT3G03855.1 \| Symbols: \| pseudogene of disease resistance protein \| chr3:986948-990738 FORWARD |
| miR169f.2 | 4.5 | AT3G22760.1 \| Symbols: SOL1 \| SOL1; transcription factor \| chr3:8044455-8047530 FORWARD |
| miR169f.2 | 4.5 | AT3G27350.1 \| Symbols: \| unknown protein \| chr3:10126021-10127955 FORWARD |
| miR169f.2 | 4.5 | AT4G04885.1 \| Symbols: PCFS4 \| PCFS4 (PCF11P-SIMILAR PROTEIN 4); zinc ion binding \| chr4:2471972-2475981 FORWARD |
| miR169f.2 | 4.5 | AT4G12545.1 \| Symbols: \| protease inhibitor/seed storage/lipid transfer protein (LTP) family protein \| chr4:7434199-7434858 FORWARD |
| miR169f.2 | 4.5 | AT5G16920.1 \| Symbols: \| FUNCTIONS IN: molecular_function unknown; LOCATED IN: endomembrane system; EXPRESSED IN: leaf whorl, sepal, flower, seed; EXPRESSED DURING: petal differentiation and expansion stage, E expanded cotyledon stage; CONTAINS InterPro DOMAIN/s: FAS1 domain (InterPro:IPR000782); BEST Arabidopsis thaliana protein match is: unknown protein (TAIR:AT5G26730.1); Has 24 Blast hits to 24 proteins in 6 species: Archae - 0; Bacteria - 0; Metazoa - 0; Fungi - 0; Plants - 18; Viruses - 0; Other Eukaryotes - 6 (source: NCBI BLink). \| chr5:5567011-5568128 FORWARD |
| miR169f.2 | 4.5 | AT5G22180.1 \| Symbols: \| unknown protein \| chr5:7353070-7353706 REVERSE |
| miR169i.2 | 3 | AT3G10160.1 \| Symbols: ATDFC, DFC \| DFC (DHFS-FPGS HOMOLOG C); tetrahydrofolylpolyglutamate synthase \| chr3:3139383-3144523 REVERSE |
| miR169i.2 | 4 | AT1G18400.1 \| Symbols: BEE1 \| BEE1 (BR Enhanced Expression 1); transcription factor \| chr1:6331398-6333743 FORWARD |
| miR169i.2 | 4 | AT1G47270.1 \| Symbols: AtTLP6 \| AtTLP6 (TUBBY LIKE PROTEIN 6); phosphoric diester hydrolase/ transcription factor \| chr1:17326556-17328728 FORWARD |
| miR169i.2 | 4 | AT2G10175.1 \| Symbols: \| transposable element gene \| chr2:3862892-3863553 REVERSE |
| miR169i.2 | 4 | AT2G43750.1 \| Symbols: OASB, ACS1, CPACS1, ATCS-B \| OASB (O-ACETYLSERINE (THIOL) LYASE B); cysteine synthase \| chr2:18129411-18132552 REVERSE |
| miR169i.2 | 4 | AT3G42258.1 \| Symbols: \| transposable element gene \| chr3:14436561-14438817 REVERSE |
| miR169i.2 | 4 | AT4G25225.1 \| Symbols: \| unknown protein \| chr4:12922934-12923307 REVERSE |
| miR169i.2 | 4.5 | AT2G15260.1 \| Symbols: \| zinc finger (C3HC4-type RING finger) family protein \| chr2:6630528-6634008 FORWARD |
| miR169i.2 | 4.5 | AT2G30870.1 \| Symbols: ATGSTF10, ERD13, ATGSTF4, GSTF10 \| GSTF10 (HALIANA GLUTATHIONE S-TRANSFERASE PHI 10); copper ion binding / glutathione binding / glutathione transferase \| chr2:13141357-13142613 FORWARD |
| miR169i.2 | 4.5 | AT3G31403.1 \| Symbols: \| transposable element gene \| chr3:12776573-12778649 FORWARD |
| miR169i.2 | 4.5 | AT3G43500.1 \| Symbols: \| unknown protein \| chr3:15400257-15400718 REVERSE |
| miR169i.2 | 4.5 | AT4G30670.1 \| Symbols: \| unknown protein \| chr4:14957358-14957962 REVERSE |
| miR169i.2 | 4.5 | AT5G67160.1 \| Symbols: EPS1 \| EPS1 (ENHANCED PSEUDOMONAS SUSCEPTIBILTY 1); transferase/ transferase, transferring acyl groups other than amino-acyl groups \| chr5:26797613-26799041 REVERSE |
| miR169i.2* | 3 | AT1G12260.1 \| Symbols: VND4, EMB2749, ANAC007 \| ANAC007 (ARABIDOPSIS NAC 007); transcription factor \| chr1:4162832-4164487 REVERSE |
| miR169i.2* | 3 | AT2G27360.1 \| Symbols: \| lipase, putative \| chr2:11706198-11708065 FORWARD |
| miR169i.2* | 3 | AT3G62600.1 \| Symbols: ATERDJ3B \| ATERDJ3B; heat shock protein binding / unfolded protein binding \| chr3:23150790-23153510 REVERSE |
| miR169i.2* | 4 | AT3G60670.1 \| Symbols: \| zinc-binding protein, putative \| chr3:22424512-22426288 REVERSE |
| miR169i.2* | 4.5 | AT1G08465.1 \| Symbols: YAB2 \| YAB2 (YABBY2); transcription factor \| chr1:2675813-2679781 FORWARD |
| miR169i.2* | 4.5 | AT2G10234.1 \| Symbols: \| transposable element gene \| chr2:3910473-3911711 FORWARD |
| miR169i.2* | 4.5 | AT2G31530.1 \| Symbols: EMB2289, SCY2 \| EMB2289 (EMBRYO DEFECTIVE 2289); P-P-bond-hydrolysis-driven protein transmembrane transporter \| chr2:13427011-13430465 FORWARD |
| miR169i.2* | 4.5 | AT2G37190.1 \| Symbols: \| 60S ribosomal protein L12 (RPL12A) \| chr2:15619407-15620119 REVERSE |
| miR169i.2* | 4.5 | AT3G01440.1 \| Symbols: \| oxygen evolving enhancer 3 (PsbQ) family protein \| chr3:168435-169618 FORWARD |
| miR169i.2* | 4.5 | AT3G27020.1 \| Symbols: YSL6 \| YSL6 (YELLOW STRIPE LIKE 6); oligopeptide transporter \| chr3:9961319-9964597 REVERSE |
| miR169i.2* | 4.5 | AT3G43960.1 \| Symbols: \| cysteine proteinase, putative \| chr3:15774055-15775657 REVERSE |
| miR169i.2* | 4.5 | AT3G49510.1 \| Symbols: \| F-box family protein \| chr3:18353842-18356508 FORWARD |
| miR169i.2* | 4.5 | AT5G02710.1 \| Symbols: \| unknown protein \| chr5:612570-614499 FORWARD |
| miR169i.2* | 4.5 | AT5G05030.1 \| Symbols: \| unknown protein \| chr5:1484177-1486065 REVERSE |
| miR169i.2* | 4.5 | AT5G39610.1 \| Symbols: ATNAC2, ORE1, ANAC092, ATNAC6 \| ATNAC6 (ARABIDOPSIS NAC DOMAIN CONTAINING PROTEIN 6); protein heterodimerization/ protein homodimerization/ transcription factor \| chr5:15858400-15859788 REVERSE |
| miR169i.2* | 4.5 | AT5G62390.1 \| Symbols: ATBAG7 \| ATBAG7 (ARABIDOPSIS THALIANA BCL-2-ASSOCIATED ATHANOGENE 7); calmodulin binding \| chr5:25051978-25054289 REVERSE |
| miR169j.2 | 3 | AT1G54440.1 \| Symbols: \| 3'-5' exonuclease/ nucleic acid binding \| chr1:20323053-20328067 FORWARD |
| miR169j.2 | 3 | AT5G35910.1 \| Symbols: \| 3'-5' exonuclease domain-containing protein / helicase and RNase D C-terminal domain-containing protein / HRDC domain-containing protein \| chr5:14029686-14035405 REVERSE |
| miR169j.2 | 3 | AT5G48300.1 \| Symbols: ADG1, APS1 \| ADG1 (ADP GLUCOSE PYROPHOSPHORYLASE 1); glucose-1-phosphate adenylyltransferase \| chr5:19570240-19572804 FORWARD |
| miR169j.2 | 3 | AT5G48470.1 \| Symbols: \| unknown protein \| chr5:19641744-19644362 FORWARD |
| miR169j.2 | 3.5 | AT1G68020.1 \| Symbols: ATTPS6, TPS6 \| ATTPS6; alpha,alpha-trehalose-phosphate synthase (UDP-forming)/ transferase, transferring glycosyl groups / trehalose-phosphatase \| chr1:25497290-25500374 FORWARD |
| miR169j.2 | 3.5 | AT2G26692.1 \| Symbols: \| other RNA \| chr2:11350719-11351293 FORWARD |
| miR169j.2 | 3.5 | AT3G22440.1 \| Symbols: \| hydroxyproline-rich glycoprotein family protein \| chr3:7959754-7962200 FORWARD |
| miR169j.2 | 3.5 | AT3G48870.1 \| Symbols: ATCLPC, ATHSP93-III, HSP93-III \| HSP93-III; ATP binding / ATPase/ DNA binding / nuclease/ nucleoside-triphosphatase/ nucleotide binding / protein binding \| chr3:18122198-18126638 REVERSE |
| miR169j.2 | 4 | AT1G13170.1 \| Symbols: ORP1D \| ORP1D (OSBP(OXYSTEROL BINDING PROTEIN)-RELATED PROTEIN 1D); oxysterol binding \| chr1:4488625-4492581 REVERSE |
| miR169j.2 | 4 | AT1G60460.1 \| Symbols: \| unknown protein \| chr1:22275484-22279007 FORWARD |
| miR169j.2 | 4 | AT2G10320.1 \| Symbols: \| transposable element gene \| chr2:3973249-3975866 FORWARD |
| miR169j.2 | 4 | AT2G36640.1 \| Symbols: ATECP63 \| ATECP63 (EMBRYONIC CELL PROTEIN 63) \| chr2:15356834-15358612 REVERSE |
| miR169j.2 | 4 | AT2G38040.1 \| Symbols: CAC3 \| CAC3; acetyl-CoA carboxylase \| chr2:15917093-15921170 FORWARD |
| miR169j.2 | 4 | AT3G19000.1 \| Symbols: \| oxidoreductase, 2OG-Fe(II) oxygenase family protein \| chr3:6553529-6555147 REVERSE |
| miR169j.2 | 4 | AT3G19055.1 \| Symbols: \| unknown protein \| chr3:6592316-6592942 FORWARD |
| miR169j.2 | 4 | AT3G19570.1 \| Symbols: \| unknown protein \| chr3:6797569-6801714 FORWARD |
| miR169j.2 | 4 | AT3G57700.1 \| Symbols: \| protein kinase, putative \| chr3:21384917-21385939 FORWARD |
| miR169j.2 | 4 | AT4G29010.1 \| Symbols: AIM1 \| AIM1 (ABNORMAL INFLORESCENCE MERISTEM); enoyl-CoA hydratase \| chr4:14297044-14302147 REVERSE |
| miR169j.2 | 4 | AT5G15840.1 \| Symbols: CO, FG \| CO (CONSTANS); transcription factor/ transcription regulator/ zinc ion binding \| chr5:5171182-5172758 REVERSE |
| miR169j.2 | 4 | AT5G27600.1 \| Symbols: LACS7, ATLACS7 \| LACS7 (LONG-CHAIN ACYL-COA SYNTHETASE 7); long-chain-fatty-acid-CoA ligase/ protein binding \| chr5:9742576-9747005 FORWARD |
| miR169j.2 | 4.5 | AT1G07650.1 \| Symbols: \| leucine-rich repeat transmembrane protein kinase, putative \| chr1:2359549-2366561 REVERSE |
| miR169j.2 | 4.5 | AT1G17110.1 \| Symbols: UBP15 \| UBP15 (UBIQUITIN-SPECIFIC PROTEASE 15); ubiquitin-specific protease \| chr1:5845675-5850889 REVERSE |
| miR169j.2 | 4.5 | AT1G19371.1 \| Symbols: MIR169H \| MIR169H; miRNA \| chr1:6695420-6695609 REVERSE |
| miR169j.2 | 4.5 | AT1G21640.1 \| Symbols: NADK2, ATNADK2 \| NADK2; NAD+ kinase/ calmodulin binding \| chr1:7588597-7592890 FORWARD |
| miR169j.2 | 4.5 | AT1G32070.1 \| Symbols: ATNSI \| ATNSI (NUCLEAR SHUTTLE INTERACTING); N-acetyltransferase \| chr1:11534724-11536305 REVERSE |
| miR169j.2 | 4.5 | AT1G35870.1 \| Symbols: \| transposable element gene \| chr1:13335257-13339587 FORWARD |
| miR169j.2 | 4.5 | AT1G36210.1 \| Symbols: \| transposable element gene \| chr1:13594514-13597863 FORWARD |
| miR169j.2 | 4.5 | AT2G06100.1 \| Symbols: \| transposable element gene \| chr2:2375867-2378098 FORWARD |
| miR169j.2 | 4.5 | AT2G16390.1 \| Symbols: DRD1, CHR35, DMS1 \| DRD1 (DEFECTIVE IN RNA-DIRECTED DNA METHYLATION 1); ATP binding / DNA binding / helicase/ nucleic acid binding \| chr2:7097638-7101096 FORWARD |
| miR169j.2 | 4.5 | AT2G20050.1 \| Symbols: \| ATP binding / cAMP-dependent protein kinase regulator/ catalytic/ protein kinase/ protein serine/threonine phosphatase \| chr2:8649434-8654228 REVERSE |
| miR169j.2 | 4.5 | AT2G32130.1 \| Symbols: \| FUNCTIONS IN: molecular_function unknown; INVOLVED IN: biological_process unknown; LOCATED IN: cellular_component unknown; CONTAINS InterPro DOMAIN/s: Protein of unknown function DUF641, plant (InterPro:IPR006943); BEST Arabidopsis thaliana protein match is: UNE1 (unfertilized embryo sac 1) (TAIR:AT1G29300.1); Has 108 Blast hits to 108 proteins in 15 species: Archae - 0; Bacteria - 14; Metazoa - 0; Fungi - 0; Plants - 92; Viruses - 0; Other Eukaryotes - 2 (source: NCBI BLink). \| chr2:13654886-13655525 REVERSE |
| miR169j.2 | 4.5 | AT3G06980.1 \| Symbols: \| DEAD/DEAH box helicase, putative \| chr3:2201467-2204833 FORWARD |
| miR169j.2 | 4.5 | AT3G12600.1 \| Symbols: atnudt16 \| atnudt16 (Arabidopsis thaliana Nudix hydrolase homolog 16); hydrolase \| chr3:4004512-4006198 FORWARD |
| miR169j.2 | 4.5 | AT3G20780.1 \| Symbols: ATTOP6B, BIN3, HYP6, RHL3 \| ATTOP6B (topoisomerase 6 subunit B); DNA topoisomerase (ATP-hydrolyzing)/ identical protein binding / protein binding \| chr3:7263900-7268572 REVERSE |
| miR169j.2 | 4.5 | AT3G62870.1 \| Symbols: \| 60S ribosomal protein L7A (RPL7aB) \| chr3:23242665-23244353 REVERSE |
| miR169j.2 | 4.5 | AT4G07498.1 \| Symbols: \| transposable element gene \| chr4:4296141-4297736 FORWARD |
| miR169j.2 | 4.5 | AT4G25020.1 \| Symbols: \| KOW domain-containing protein / D111/G-patch domain-containing protein \| chr4:12860634-12861975 REVERSE |
| miR169j.2 | 4.5 | AT4G28610.1 \| Symbols: PHR1, AtPHR1 \| PHR1 (PHOSPHATE STARVATION RESPONSE 1); transcription factor \| chr4:14132856-14135259 REVERSE |
| miR169j.2 | 4.5 | AT5G06670.1 \| Symbols: \| ATP binding / microtubule motor \| chr5:2048243-2055019 REVERSE |
| miR169j.2 | 4.5 | AT5G22010.1 \| Symbols: AtRFC1 \| AtRFC1 (replication factor C 1); ATP binding / DNA binding / DNA clamp loader/ nucleoside-triphosphatase/ nucleotide binding \| chr5:7280389-7287364 REVERSE |
| miR169j.2 | 4.5 | AT5G27550.1 \| Symbols: \| ATP binding / microtubule motor \| chr5:9727634-9731323 REVERSE |
| miR169j.2 | 4.5 | AT5G29436.1 \| Symbols: \| transposable element gene \| chr5:11175677-11176666 REVERSE |
| miR169j.2 | 4.5 | AT5G33990.1 \| Symbols: \| transposable element gene \| chr5:12791432-12794551 REVERSE |
| miR169l.2 | 2.5 | AT1G21640.1 \| Symbols: NADK2, ATNADK2 \| NADK2; NAD+ kinase/ calmodulin binding \| chr1:7588597-7592890 FORWARD |
| miR169l.2 | 3 | AT1G17110.1 \| Symbols: UBP15 \| UBP15 (UBIQUITIN-SPECIFIC PROTEASE 15); ubiquitin-specific protease \| chr1:5845675-5850889 REVERSE |
| miR169l.2 | 3 | AT3G42060.1 \| Symbols: \| myosin heavy chain-related \| chr3:14251600-14254269 REVERSE |
| miR169l.2 | 3.5 | AT5G56340.1 \| Symbols: ATCRT1 \| zinc finger (C3HC4-type RING finger) family protein \| chr5:22817560-22819657 FORWARD |
| miR169l.2 | 4 | AT1G06950.1 \| Symbols: ATTIC110, TIC110 \| TIC110 (TRANSLOCON AT THE INNER ENVELOPE MEMBRANE OF CHLOROPLASTS 110) \| chr1:2130142-2135665 REVERSE |
| miR169l.2 | 4 | AT1G09090.1 \| Symbols: ATRBOHB \| ATRBOHB (respiratory burst oxidase homolog B); FAD binding / calcium ion binding / electron carrier/ iron ion binding / oxidoreductase/ oxidoreductase, acting on NADH or NADPH, with oxygen as acceptor / peroxidase \| chr1:2932739-2936586 FORWARD |
| miR169l.2 | 4 | AT1G27720.1 \| Symbols: TAF4, TAF4B \| TAF4B (TBP-ASSOCIATED FACTOR 4B); transcription initiation factor \| chr1:9643351-9647376 REVERSE |
| miR169l.2 | 4 | AT2G24545.1 \| Symbols: \| other RNA \| chr2:10425818-10427263 REVERSE |
| miR169l.2 | 4 | AT2G25260.1 \| Symbols: \| unknown protein \| chr2:10755524-10757772 REVERSE |
| miR169l.2 | 4 | AT2G43500.1 \| Symbols: \| RWP-RK domain-containing protein \| chr2:18061925-18066639 FORWARD |
| miR169l.2 | 4 | AT4G05390.1 \| Symbols: ATRFNR1 \| ATRFNR1 (ROOT FNR 1); FAD binding / NADP or NADPH binding / electron carrier/ ferredoxin-NADP+ reductase/ oxidoreductase \| chr4:2738715-2740655 REVERSE |
| miR169l.2 | 4 | AT4G11160.1 \| Symbols: \| translation initiation factor IF-2, mitochondrial, putative \| chr4:6803594-6806875 FORWARD |
| miR169l.2 | 4 | AT5G22300.1 \| Symbols: NIT4 \| NIT4 (NITRILASE 4); 3-cyanoalanine hydratase/ cyanoalanine nitrilase/ indole-3-acetonitrile nitrilase/ nitrilase/ nitrile hydratase \| chr5:7379199-7381935 FORWARD |
| miR169l.2 | 4 | AT5G29720.1 \| Symbols: \| transposable element gene \| chr5:11294565-11300009 FORWARD |
| miR169l.2 | 4 | AT5G35910.1 \| Symbols: \| 3'-5' exonuclease domain-containing protein / helicase and RNase D C-terminal domain-containing protein / HRDC domain-containing protein \| chr5:14029686-14035405 REVERSE |
| miR169l.2 | 4 | AT5G40820.1 \| Symbols: ATRAD3, ATR, ATATR \| ATRAD3; binding / inositol or phosphatidylinositol kinase/ phosphotransferase, alcohol group as acceptor / protein serine/threonine kinase \| chr5:16342745-16353898 REVERSE |
| miR169l.2 | 4 | AT5G48300.1 \| Symbols: ADG1, APS1 \| ADG1 (ADP GLUCOSE PYROPHOSPHORYLASE 1); glucose-1-phosphate adenylyltransferase \| chr5:19570240-19572804 FORWARD |
| miR169l.2 | 4 | AT5G48470.1 \| Symbols: \| unknown protein \| chr5:19641744-19644362 FORWARD |
| miR169l.2 | 4 | AT5G49900.1 \| Symbols: \| catalytic/ glucosylceramidase \| chr5:20296518-20302346 REVERSE |
| miR169l.2 | 4.5 | AT1G08060.1 \| Symbols: MOM \| MOM (MORPHEUS MOLECULE) \| chr1:2501742-2511084 REVERSE |
| miR169l.2 | 4.5 | AT1G28680.1 \| Symbols: \| transferase family protein \| chr1:10078189-10080029 FORWARD |
| miR169l.2 | 4.5 | AT1G42816.1 \| Symbols: \| transposable element gene \| chr1:16104714-16105401 REVERSE |
| miR169l.2 | 4.5 | AT1G51740.1 \| Symbols: SYP81, ATUFE1, ATSYP81, UFE1 \| SYP81 (SYNTAXIN OF PLANTS 81); SNAP receptor/ protein binding \| chr1:19188843-19191300 FORWARD |
| miR169l.2 | 4.5 | AT1G56040.1 \| Symbols: \| binding / protein binding / ubiquitin-protein ligase \| chr1:20960356-20962334 REVERSE |
| miR169l.2 | 4.5 | AT1G78410.1 \| Symbols: \| VQ motif-containing protein \| chr1:29502582-29503241 FORWARD |
| miR169l.2 | 4.5 | AT2G21410.1 \| Symbols: VHA-A2 \| VHA-A2 (VACUOLAR PROTON ATPASE A2); ATPase \| chr2:9162620-9168452 FORWARD |
| miR169l.2 | 4.5 | AT2G26692.1 \| Symbols: \| other RNA \| chr2:11350719-11351293 FORWARD |
| miR169l.2 | 4.5 | AT2G42560.1 \| Symbols: \| late embryogenesis abundant domain-containing protein / LEA domain-containing protein \| chr2:17714638-17716912 REVERSE |
| miR169l.2 | 4.5 | AT3G04180.1 \| Symbols: \| germin-like protein, putative \| chr3:1097475-1098422 REVERSE |
| miR169l.2 | 4.5 | AT3G06940.1 \| Symbols: \| transposable element gene \| chr3:2189479-2192622 REVERSE |
| miR169l.2 | 4.5 | AT3G15120.1 \| Symbols: \| AAA-type ATPase family protein \| chr3:5088366-5095482 REVERSE |
| miR169l.2 | 4.5 | AT4G14900.1 \| Symbols: \| hydroxyproline-rich glycoprotein family protein \| chr4:8521522-8523705 REVERSE |
| miR169l.2 | 4.5 | AT4G21540.1 \| Symbols: SPHK1 \| SPHK1 (SPHINGOSINE KINASE 1); D-erythro-sphingosine kinase/ diacylglycerol kinase/ sphinganine kinase \| chr4:11454775-11462606 FORWARD |
| miR169l.2 | 4.5 | AT4G26020.1 \| Symbols: \| FUNCTIONS IN: molecular_function unknown; INVOLVED IN: biological_process unknown; LOCATED IN: cellular_component unknown; EXPRESSED IN: embryo, flower, seed; EXPRESSED DURING: petal differentiation and expansion stage, D bilateral stage, E expanded cotyledon stage; BEST Arabidopsis thaliana protein match is: ATGRIP; protein binding (TAIR:AT5G66030.2); Has 14545 Blast hits to 10066 proteins in 618 species: Archae - 167; Bacteria - 1198; Metazoa - 6354; Fungi - 992; Plants - 404; Viruses - 34; Other Eukaryotes - 5396 (source: NCBI BLink). \| chr4:13202013-13204028 REVERSE |
| miR169l.2 | 4.5 | AT4G26400.1 \| Symbols: \| zinc finger (C3HC4-type RING finger) family protein \| chr4:13344808-13346597 REVERSE |
| miR169l.2 | 4.5 | AT5G27550.1 \| Symbols: \| ATP binding / microtubule motor \| chr5:9727634-9731323 REVERSE |
| miR169l.2 | 4.5 | AT5G38550.1 \| Symbols: \| jacalin lectin family protein \| chr5:15432781-15435779 REVERSE |
| miR169l.2 | 4.5 | AT5G48965.1 \| Symbols: \| transposable element gene \| chr5:19853991-19856859 FORWARD |
| miR169l.2 | 4.5 | AT5G61970.1 \| Symbols: \| signal recognition particle-related / SRP-related \| chr5:24888617-24893328 FORWARD |
| miR169l.2 | 4.5 | AT5G67530.1 \| Symbols: \| peptidyl-prolyl cis-trans isomerase cyclophilin-type family protein \| chr5:26941182-26944146 FORWARD |
| miR169m.2 | 3 | AT3G10160.1 \| Symbols: ATDFC, DFC \| DFC (DHFS-FPGS HOMOLOG C); tetrahydrofolylpolyglutamate synthase \| chr3:3139383-3144523 REVERSE |
| miR169m.2 | 4 | AT1G18400.1 \| Symbols: BEE1 \| BEE1 (BR Enhanced Expression 1); transcription factor \| chr1:6331398-6333743 FORWARD |
| miR169m.2 | 4 | AT1G47270.1 \| Symbols: AtTLP6 \| AtTLP6 (TUBBY LIKE PROTEIN 6); phosphoric diester hydrolase/ transcription factor \| chr1:17326556-17328728 FORWARD |
| miR169m.2 | 4 | AT2G10175.1 \| Symbols: \| transposable element gene \| chr2:3862892-3863553 REVERSE |
| miR169m.2 | 4 | AT2G43750.1 \| Symbols: OASB, ACS1, CPACS1, ATCS-B \| OASB (O-ACETYLSERINE (THIOL) LYASE B); cysteine synthase \| chr2:18129411-18132552 REVERSE |
| miR169m.2 | 4 | AT3G42258.1 \| Symbols: \| transposable element gene \| chr3:14436561-14438817 REVERSE |
| miR169m.2 | 4 | AT4G25225.1 \| Symbols: \| unknown protein \| chr4:12922934-12923307 REVERSE |
| miR169m.2 | 4.5 | AT2G15260.1 \| Symbols: \| zinc finger (C3HC4-type RING finger) family protein \| chr2:6630528-6634008 FORWARD |
| miR169m.2 | 4.5 | AT2G30870.1 \| Symbols: ATGSTF10, ERD13, ATGSTF4, GSTF10 \| GSTF10 (HALIANA GLUTATHIONE S-TRANSFERASE PHI 10); copper ion binding / glutathione binding / glutathione transferase \| chr2:13141357-13142613 FORWARD |
| miR169m.2 | 4.5 | AT3G31403.1 \| Symbols: \| transposable element gene \| chr3:12776573-12778649 FORWARD |
| miR169m.2 | 4.5 | AT3G43500.1 \| Symbols: \| unknown protein \| chr3:15400257-15400718 REVERSE |
| miR169m.2 | 4.5 | AT4G30670.1 \| Symbols: \| unknown protein \| chr4:14957358-14957962 REVERSE |
| miR169m.2 | 4.5 | AT5G67160.1 \| Symbols: EPS1 \| EPS1 (ENHANCED PSEUDOMONAS SUSCEPTIBILTY 1); transferase/ transferase, transferring acyl groups other than amino-acyl groups \| chr5:26797613-26799041 REVERSE |
| miR169n.2 | 2.5 | AT1G21640.1 \| Symbols: NADK2, ATNADK2 \| NADK2; NAD+ kinase/ calmodulin binding \| chr1:7588597-7592890 FORWARD |
| miR169n.2 | 3 | AT1G17110.1 \| Symbols: UBP15 \| UBP15 (UBIQUITIN-SPECIFIC PROTEASE 15); ubiquitin-specific protease \| chr1:5845675-5850889 REVERSE |
| miR169n.2 | 3 | AT3G42060.1 \| Symbols: \| myosin heavy chain-related \| chr3:14251600-14254269 REVERSE |
| miR169n.2 | 3.5 | AT5G56340.1 \| Symbols: ATCRT1 \| zinc finger (C3HC4-type RING finger) family protein \| chr5:22817560-22819657 FORWARD |
| miR169n.2 | 4 | AT1G06950.1 \| Symbols: ATTIC110, TIC110 \| TIC110 (TRANSLOCON AT THE INNER ENVELOPE MEMBRANE OF CHLOROPLASTS 110) \| chr1:2130142-2135665 REVERSE |
| miR169n.2 | 4 | AT1G09090.1 \| Symbols: ATRBOHB \| ATRBOHB (respiratory burst oxidase homolog B); FAD binding / calcium ion binding / electron carrier/ iron ion binding / oxidoreductase/ oxidoreductase, acting on NADH or NADPH, with oxygen as acceptor / peroxidase \| chr1:2932739-2936586 FORWARD |
| miR169n.2 | 4 | AT1G27720.1 \| Symbols: TAF4, TAF4B \| TAF4B (TBP-ASSOCIATED FACTOR 4B); transcription initiation factor \| chr1:9643351-9647376 REVERSE |
| miR169n.2 | 4 | AT2G24545.1 \| Symbols: \| other RNA \| chr2:10425818-10427263 REVERSE |
| miR169n.2 | 4 | AT2G25260.1 \| Symbols: \| unknown protein \| chr2:10755524-10757772 REVERSE |
| miR169n.2 | 4 | AT2G43500.1 \| Symbols: \| RWP-RK domain-containing protein \| chr2:18061925-18066639 FORWARD |
| miR169n.2 | 4 | AT4G05390.1 \| Symbols: ATRFNR1 \| ATRFNR1 (ROOT FNR 1); FAD binding / NADP or NADPH binding / electron carrier/ ferredoxin-NADP+ reductase/ oxidoreductase \| chr4:2738715-2740655 REVERSE |
| miR169n.2 | 4 | AT4G11160.1 \| Symbols: \| translation initiation factor IF-2, mitochondrial, putative \| chr4:6803594-6806875 FORWARD |
| miR169n.2 | 4 | AT5G22300.1 \| Symbols: NIT4 \| NIT4 (NITRILASE 4); 3-cyanoalanine hydratase/ cyanoalanine nitrilase/ indole-3-acetonitrile nitrilase/ nitrilase/ nitrile hydratase \| chr5:7379199-7381935 FORWARD |
| miR169n.2 | 4 | AT5G29720.1 \| Symbols: \| transposable element gene \| chr5:11294565-11300009 FORWARD |
| miR169n.2 | 4 | AT5G35910.1 \| Symbols: \| 3'-5' exonuclease domain-containing protein / helicase and RNase D C-terminal domain-containing protein / HRDC domain-containing protein \| chr5:14029686-14035405 REVERSE |
| miR169n.2 | 4 | AT5G40820.1 \| Symbols: ATRAD3, ATR, ATATR \| ATRAD3; binding / inositol or phosphatidylinositol kinase/ phosphotransferase, alcohol group as acceptor / protein serine/threonine kinase \| chr5:16342745-16353898 REVERSE |
| miR169n.2 | 4 | AT5G48300.1 \| Symbols: ADG1, APS1 \| ADG1 (ADP GLUCOSE PYROPHOSPHORYLASE 1); glucose-1-phosphate adenylyltransferase \| chr5:19570240-19572804 FORWARD |
| miR169n.2 | 4 | AT5G48470.1 \| Symbols: \| unknown protein \| chr5:19641744-19644362 FORWARD |
| miR169n.2 | 4 | AT5G49900.1 \| Symbols: \| catalytic/ glucosylceramidase \| chr5:20296518-20302346 REVERSE |
| miR169n.2 | 4.5 | AT1G08060.1 \| Symbols: MOM \| MOM (MORPHEUS MOLECULE) \| chr1:2501742-2511084 REVERSE |
| miR169n.2 | 4.5 | AT1G28680.1 \| Symbols: \| transferase family protein \| chr1:10078189-10080029 FORWARD |
| miR169n.2 | 4.5 | AT1G42816.1 \| Symbols: \| transposable element gene \| chr1:16104714-16105401 REVERSE |
| miR169n.2 | 4.5 | AT1G51740.1 \| Symbols: SYP81, ATUFE1, ATSYP81, UFE1 \| SYP81 (SYNTAXIN OF PLANTS 81); SNAP receptor/ protein binding \| chr1:19188843-19191300 FORWARD |
| miR169n.2 | 4.5 | AT1G56040.1 \| Symbols: \| binding / protein binding / ubiquitin-protein ligase \| chr1:20960356-20962334 REVERSE |
| miR169n.2 | 4.5 | AT1G78410.1 \| Symbols: \| VQ motif-containing protein \| chr1:29502582-29503241 FORWARD |
| miR169n.2 | 4.5 | AT2G21410.1 \| Symbols: VHA-A2 \| VHA-A2 (VACUOLAR PROTON ATPASE A2); ATPase \| chr2:9162620-9168452 FORWARD |
| miR169n.2 | 4.5 | AT2G26692.1 \| Symbols: \| other RNA \| chr2:11350719-11351293 FORWARD |
| miR169n.2 | 4.5 | AT2G42560.1 \| Symbols: \| late embryogenesis abundant domain-containing protein / LEA domain-containing protein \| chr2:17714638-17716912 REVERSE |
| miR169n.2 | 4.5 | AT3G04180.1 \| Symbols: \| germin-like protein, putative \| chr3:1097475-1098422 REVERSE |
| miR169n.2 | 4.5 | AT3G06940.1 \| Symbols: \| transposable element gene \| chr3:2189479-2192622 REVERSE |
| miR169n.2 | 4.5 | AT3G15120.1 \| Symbols: \| AAA-type ATPase family protein \| chr3:5088366-5095482 REVERSE |
| miR169n.2 | 4.5 | AT4G14900.1 \| Symbols: \| hydroxyproline-rich glycoprotein family protein \| chr4:8521522-8523705 REVERSE |
| miR169n.2 | 4.5 | AT4G21540.1 \| Symbols: SPHK1 \| SPHK1 (SPHINGOSINE KINASE 1); D-erythro-sphingosine kinase/ diacylglycerol kinase/ sphinganine kinase \| chr4:11454775-11462606 FORWARD |
| miR169n.2 | 4.5 | AT4G26020.1 \| Symbols: \| FUNCTIONS IN: molecular_function unknown; INVOLVED IN: biological_process unknown; LOCATED IN: cellular_component unknown; EXPRESSED IN: embryo, flower, seed; EXPRESSED DURING: petal differentiation and expansion stage, D bilateral stage, E expanded cotyledon stage; BEST Arabidopsis thaliana protein match is: ATGRIP; protein binding (TAIR:AT5G66030.2); Has 14545 Blast hits to 10066 proteins in 618 species: Archae - 167; Bacteria - 1198; Metazoa - 6354; Fungi - 992; Plants - 404; Viruses - 34; Other Eukaryotes - 5396 (source: NCBI BLink). \| chr4:13202013-13204028 REVERSE |
| miR169n.2 | 4.5 | AT4G26400.1 \| Symbols: \| zinc finger (C3HC4-type RING finger) family protein \| chr4:13344808-13346597 REVERSE |
| miR169n.2 | 4.5 | AT5G27550.1 \| Symbols: \| ATP binding / microtubule motor \| chr5:9727634-9731323 REVERSE |
| miR169n.2 | 4.5 | AT5G38550.1 \| Symbols: \| jacalin lectin family protein \| chr5:15432781-15435779 REVERSE |
| miR169n.2 | 4.5 | AT5G48965.1 \| Symbols: \| transposable element gene \| chr5:19853991-19856859 FORWARD |
| miR169n.2 | 4.5 | AT5G61970.1 \| Symbols: \| signal recognition particle-related / SRP-related \| chr5:24888617-24893328 FORWARD |
| miR169n.2 | 4.5 | AT5G67530.1 \| Symbols: \| peptidyl-prolyl cis-trans isomerase cyclophilin-type family protein \| chr5:26941182-26944146 FORWARD |
| miR169n.2* | 4.5 | AT1G48970.1 \| Symbols: \| GTP binding / translation initiation factor \| chr1:18113714-18117833 REVERSE |
| miR319a.2 | 4 | AT3G06080.1 \| Symbols: \| unknown protein \| chr3:1834707-1837984 REVERSE |
| miR319a.2 | 4.5 | AT3G20190.1 \| Symbols: \| leucine-rich repeat transmembrane protein kinase, putative \| chr3:7044943-7047390 FORWARD |
| miR319b.2 | 2 | AT3G06080.1 \| Symbols: \| unknown protein \| chr3:1834707-1837984 REVERSE |
| miR319b.2 | 4 | AT1G36927.1 \| Symbols: \| transposable element gene \| chr1:13991779-13993095 REVERSE |
| miR319b.2 | 4 | AT1G72960.1 \| Symbols: \| root hair defective 3 GTP-binding (RHD3) family protein \| chr1:27446614-27450862 FORWARD |
| miR319b.2 | 4 | AT2G11450.1 \| Symbols: \| transposable element gene \| chr2:4573616-4581219 REVERSE |
| miR319b.2 | 4 | AT5G59790.1 \| Symbols: \| unknown protein \| chr5:24090715-24093168 FORWARD |
| miR319b.2 | 4 | AT5G64280.1 \| Symbols: DiT2.2 \| DiT2.2 (dicarboxylate transporter 2.2); oxoglutarate:malate antiporter \| chr5:25711069-25713473 REVERSE |
| miR319b.2 | 4.5 | AT2G07670.1 \| Symbols: \| Pseudogene of AT2G07702 \| chr2:3506865-3507323 FORWARD |
| miR319b.2 | 4.5 | AT2G07702.1 \| Symbols: \| unknown protein \| chr2:3372697-3373155 FORWARD |
| miR319b.2 | 4.5 | AT4G06587.1 \| Symbols: \| transposable element gene \| chr4:3594144-3597062 REVERSE |
| miR319b.2 | 4.5 | ATMG00440.1 \| Symbols: ORF152A \| hypothetical protein \| chrM:116296-116754 FORWARD |
| miR319b.2 | 4.5 | ATMG01140.1 \| Symbols: ORF152B \| hypothetical protein \| chrM:292973-293431 REVERSE |
| miR447a.2-3p | 4.5 | AT1G28310.1 \| Symbols: \| Dof-type zinc finger domain-containing protein \| chr1:9912203-9913837 REVERSE |
| miR447a.2-5p | 4.5 | AT3G13250.1 \| Symbols: \| transposable element gene \| chr3:4276747-4281476 REVERSE |
| miR447a.2-5p | 4.5 | AT5G02170.1 \| Symbols: \| amino acid transporter family protein \| chr5:427833-430695 FORWARD |
| miR447a.3 | 2 | AT1G42630.1 \| Symbols: \| transposable element gene \| chr1:16025298-16025762 FORWARD |
| miR447a.3 | 2 | AT1G54710.1 \| Symbols: ATATG18H \| AtATG18h \| chr1:20416770-20421035 REVERSE |
| miR447a.3 | 3 | AT2G33350.1 \| Symbols: \| FUNCTIONS IN: molecular_function unknown; INVOLVED IN: biological_process unknown; LOCATED IN: cellular_component unknown; EXPRESSED IN: 7 plant structures; EXPRESSED DURING: L mature pollen stage, M germinated pollen stage, 4 anthesis; CONTAINS InterPro DOMAIN/s: CCT domain (InterPro:IPR010402); BEST Arabidopsis thaliana protein match is: zinc finger CONSTANS-related (TAIR:AT1G04500.1); Has 7691 Blast hits to 4729 proteins in 129 species: Archae - 8; Bacteria - 25; Metazoa - 148; Fungi - 114; Plants - 918; Viruses - 0; Other Eukaryotes - 6478 (source: NCBI BLink). \| chr2:14133929-14137154 FORWARD |
| miR447a.3 | 3.5 | AT1G40080.1 \| Symbols: \| transposable element gene \| chr1:14917168-14917632 REVERSE |
| miR447a.3 | 3.5 | AT2G28800.1 \| Symbols: ALB3, ABL3 \| ALB3 (ALBINO 3); P-P-bond-hydrolysis-driven protein transmembrane transporter \| chr2:12356364-12359252 REVERSE |
| miR447a.3 | 3.5 | AT3G03280.1 \| Symbols: \| unknown protein \| chr3:765459-766387 FORWARD |
| miR447a.3 | 3.5 | AT4G05580.1 \| Symbols: \| transposable element gene \| chr4:2841648-2841998 FORWARD |
| miR447a.3 | 4 | AT1G06770.1 \| Symbols: DRIP1 \| DRIP1 (DREB2A-INTERACTING PROTEIN 1); protein binding / ubiquitin-protein ligase/ zinc ion binding \| chr1:2078911-2081927 REVERSE |
| miR447a.3 | 4.5 | AT1G20120.1 \| Symbols: \| family II extracellular lipase, putative \| chr1:6975388-6977264 FORWARD |
| miR447a.3 | 4.5 | AT1G24145.1 \| Symbols: \| unknown protein \| chr1:8541027-8541739 FORWARD |
| miR447a.3 | 4.5 | AT1G71320.1 \| Symbols: \| S locus F-box-related / SLF-related \| chr1:26882485-26883818 FORWARD |
| miR447a.3 | 4.5 | AT2G15300.1 \| Symbols: \| leucine-rich repeat transmembrane protein kinase, putative \| chr2:6649630-6652010 FORWARD |
| miR447a.3 | 4.5 | AT3G27910.1 \| Symbols: \| kelch repeat-containing protein \| chr3:10357232-10358116 REVERSE |
| miR447a.3 | 4.5 | AT5G06380.1 \| Symbols: \| unknown protein \| chr5:1949315-1950282 FORWARD |
| miR447a.3 | 4.5 | AT5G55820.1 \| Symbols: \| unknown protein \| chr5:22586287-22594035 FORWARD |
| miR447a.3 | 4.5 | AT5G61120.1 \| Symbols: \| INVOLVED IN: biological_process unknown; EXPRESSED IN: 8 plant structures; EXPRESSED DURING: 6 growth stages; BEST Arabidopsis thaliana protein match is: nucleic acid binding (TAIR:AT5G61090.1); Has 77 Blast hits to 70 proteins in 8 species: Archae - 0; Bacteria - 0; Metazoa - 20; Fungi - 0; Plants - 56; Viruses - 0; Other Eukaryotes - 1 (source: NCBI BLink). \| chr5:24582303-24585806 REVERSE |
| miR447b.2 | 4.5 | AT1G28310.1 \| Symbols: \| Dof-type zinc finger domain-containing protein \| chr1:9912203-9913837 REVERSE |
| miR447b.3 | 2 | AT1G42630.1 \| Symbols: \| transposable element gene \| chr1:16025298-16025762 FORWARD |
| miR447b.3 | 2 | AT1G54710.1 \| Symbols: ATATG18H \| AtATG18h \| chr1:20416770-20421035 REVERSE |
| miR447b.3 | 3 | AT2G33350.1 \| Symbols: \| FUNCTIONS IN: molecular_function unknown; INVOLVED IN: biological_process unknown; LOCATED IN: cellular_component unknown; EXPRESSED IN: 7 plant structures; EXPRESSED DURING: L mature pollen stage, M germinated pollen stage, 4 anthesis; CONTAINS InterPro DOMAIN/s: CCT domain (InterPro:IPR010402); BEST Arabidopsis thaliana protein match is: zinc finger CONSTANS-related (TAIR:AT1G04500.1); Has 7691 Blast hits to 4729 proteins in 129 species: Archae - 8; Bacteria - 25; Metazoa - 148; Fungi - 114; Plants - 918; Viruses - 0; Other Eukaryotes - 6478 (source: NCBI BLink). \| chr2:14133929-14137154 FORWARD |
| miR447b.3 | 3.5 | AT1G40080.1 \| Symbols: \| transposable element gene \| chr1:14917168-14917632 REVERSE |
| miR447b.3 | 3.5 | AT2G28800.1 \| Symbols: ALB3, ABL3 \| ALB3 (ALBINO 3); P-P-bond-hydrolysis-driven protein transmembrane transporter \| chr2:12356364-12359252 REVERSE |
| miR447b.3 | 3.5 | AT3G03280.1 \| Symbols: \| unknown protein \| chr3:765459-766387 FORWARD |
| miR447b.3 | 3.5 | AT4G05580.1 \| Symbols: \| transposable element gene \| chr4:2841648-2841998 FORWARD |
| miR447b.3 | 4 | AT1G06770.1 \| Symbols: DRIP1 \| DRIP1 (DREB2A-INTERACTING PROTEIN 1); protein binding / ubiquitin-protein ligase/ zinc ion binding \| chr1:2078911-2081927 REVERSE |
| miR447b.3 | 4.5 | AT1G20120.1 \| Symbols: \| family II extracellular lipase, putative \| chr1:6975388-6977264 FORWARD |
| miR447b.3 | 4.5 | AT1G24145.1 \| Symbols: \| unknown protein \| chr1:8541027-8541739 FORWARD |
| miR447b.3 | 4.5 | AT1G71320.1 \| Symbols: \| S locus F-box-related / SLF-related \| chr1:26882485-26883818 FORWARD |
| miR447b.3 | 4.5 | AT2G15300.1 \| Symbols: \| leucine-rich repeat transmembrane protein kinase, putative \| chr2:6649630-6652010 FORWARD |
| miR447b.3 | 4.5 | AT3G27910.1 \| Symbols: \| kelch repeat-containing protein \| chr3:10357232-10358116 REVERSE |
| miR447b.3 | 4.5 | AT5G06380.1 \| Symbols: \| unknown protein \| chr5:1949315-1950282 FORWARD |
| miR447b.3 | 4.5 | AT5G55820.1 \| Symbols: \| unknown protein \| chr5:22586287-22594035 FORWARD |
| miR447b.3 | 4.5 | AT5G61120.1 \| Symbols: \| INVOLVED IN: biological_process unknown; EXPRESSED IN: 8 plant structures; EXPRESSED DURING: 6 growth stages; BEST Arabidopsis thaliana protein match is: nucleic acid binding (TAIR:AT5G61090.1); Has 77 Blast hits to 70 proteins in 8 species: Archae - 0; Bacteria - 0; Metazoa - 20; Fungi - 0; Plants - 56; Viruses - 0; Other Eukaryotes - 1 (source: NCBI BLink). \| chr5:24582303-24585806 REVERSE |
| miR775.2 | 2.5 | AT2G13460.1 \| Symbols: \| transposable element gene \| chr2:5600306-5605593 REVERSE |
| miR775.2 | 3 | AT1G56310.1 \| Symbols: \| 3'-5' exonuclease domain-containing protein \| chr1:21082863-21085300 FORWARD |
| miR775.2 | 3 | AT1G56345.1 \| Symbols: \| pseudouridine synthase family protein \| chr1:21092993-21094461 REVERSE |
| miR775.2 | 3 | AT2G04515.1 \| Symbols: \| unknown protein \| chr2:1573637-1574478 FORWARD |
| miR775.2 | 3 | AT4G31080.1 \| Symbols: \| unknown protein \| chr4:15120888-15123486 FORWARD |
| miR775.2 | 3.5 | AT2G32400.1 \| Symbols: GLR5, GLR3.7, ATGLR3.7 \| GLR5 (GLUTAMATE RECEPTOR 5); intracellular ligand-gated ion channel \| chr2:13752584-13756853 REVERSE |
| miR775.2 | 3.5 | AT4G38545.1 \| Symbols: \| other RNA \| chr4:18023022-18025287 REVERSE |
| miR775.2 | 4 | AT1G01040.1 \| Symbols: DCL1, CAF, SUS1, SIN1, ASU1, EMB76, EMB60, ATDCL1 \| DCL1 (DICER-LIKE 1); ATP-dependent helicase/ double-stranded RNA binding / protein binding / ribonuclease III \| chr1:23146-31227 FORWARD |
| miR775.2 | 4 | AT2G04340.1 \| Symbols: \| unknown protein \| chr2:1513512-1515540 FORWARD |
| miR775.2 | 4 | AT2G35100.1 \| Symbols: ARAD1 \| ARAD1 (ARABINAN DEFICIENT 1); catalytic/ transferase, transferring glycosyl groups \| chr2:14793651-14795629 REVERSE |
| miR775.2 | 4 | AT2G45740.1 \| Symbols: PEX11D \| PEX11D \| chr2:18839716-18841403 FORWARD |
| miR775.2 | 4 | AT4G04655.1 \| Symbols: \| transposable element gene \| chr4:2357418-2364944 FORWARD |
| miR775.2 | 4 | AT5G28670.1 \| Symbols: \| transposable element gene \| chr5:10691737-10695537 REVERSE |
| miR775.2 | 4.5 | AT1G10380.1 \| Symbols: \| unknown protein \| chr1:3400606-3402324 FORWARD |
| miR775.2 | 4.5 | AT1G11800.1 \| Symbols: \| endonuclease/exonuclease/phosphatase family protein \| chr1:3985225-3987290 REVERSE |
| miR775.2 | 4.5 | AT1G63105.1 \| Symbols: \| unknown protein \| chr1:23404141-23404509 FORWARD |
| miR775.2 | 4.5 | AT2G05084.1 \| Symbols: \| transposable element gene \| chr2:1814327-1815487 REVERSE |
| miR775.2 | 4.5 | AT3G05420.1 \| Symbols: ACBP4 \| ACBP4 (ACYL-COA BINDING PROTEIN 4); acyl-CoA binding \| chr3:1561778-1567318 FORWARD |
| miR775.2 | 4.5 | AT3G11980.1 \| Symbols: MS2, FAR2 \| MS2 (MALE STERILITY 2); fatty acyl-CoA reductase (alcohol-forming)/ oxidoreductase, acting on the CH-CH group of donors, NAD or NADP as acceptor \| chr3:3814236-3817117 FORWARD |
| miR775.2 | 4.5 | AT3G62150.1 \| Symbols: PGP21 \| PGP21 (P-GLYCOPROTEIN 21); ATPase, coupled to transmembrane movement of substances \| chr3:23008755-23013579 REVERSE |
| miR775.2 | 4.5 | AT4G06604.1 \| Symbols: \| transposable element gene \| chr4:3685906-3691542 REVERSE |
| miR775.2 | 4.5 | AT4G33810.1 \| Symbols: \| glycosyl hydrolase family 10 protein \| chr4:16213324-16215594 REVERSE |
| miR775.2 | 4.5 | AT5G38690.1 \| Symbols: \| FUNCTIONS IN: molecular_function unknown; INVOLVED IN: biological_process unknown; LOCATED IN: cellular_component unknown; EXPRESSED IN: 21 plant structures; EXPRESSED DURING: 13 growth stages; CONTAINS InterPro DOMAIN/s: DDT superfamily (InterPro:IPR018501), DDT subgroup (InterPro:IPR018500), Cell division cycle-associated protein (InterPro:IPR018866); BEST Arabidopsis thaliana protein match is: unknown protein (TAIR:AT1G67780.1); Has 492 Blast hits to 468 proteins in 97 species: Archae - 9; Bacteria - 41; Metazoa - 179; Fungi - 19; Plants - 99; Viruses - 2; Other Eukaryotes - 143 (source: NCBI BLink). \| chr5:15479177-15483250 REVERSE |
| miR775.2 | 4.5 | AT5G39473.1 \| Symbols: \| pseudogene of DC1 (domain-containing protein) \| chr5:15797758-15799724 REVERSE |
| miR775.2 | 4.5 | AT5G56850.1 \| Symbols: \| unknown protein \| chr5:22985193-22988013 REVERSE |
| miR775.2 | 4.5 | AT5G58120.1 \| Symbols: \| disease resistance protein (TIR-NBS-LRR class), putative \| chr5:23517492-23521067 FORWARD |
| miR775.2 | 4.5 | AT5G67540.1 \| Symbols: \| glycosyl hydrolase family protein 43 \| chr5:26944028-26946365 REVERSE |
| miR822.2 | 4 | AT5G45030.1 \| Symbols: \| catalytic \| chr5:18172471-18176207 FORWARD |
| miR822.2 | 4.5 | AT5G07850.1 \| Symbols: \| transferase family protein \| chr5:2508917-2510503 FORWARD |
| miR822.3 | 4 | AT2G03210.1 \| Symbols: FUT2, ATFUT2 \| FUT2 (FUCOSYLTRANSFERASE 2); fucosyltransferase/ transferase, transferring glycosyl groups \| chr2:968335-970018 REVERSE |
| miR822.3 | 4 | AT5G07070.1 \| Symbols: CIPK2, SnRK3.2 \| CIPK2 (CBL-INTERACTING PROTEIN KINASE 2); ATP binding / kinase/ protein kinase/ protein serine/threonine kinase \| chr5:2196434-2198114 REVERSE |
| miR822.3* | 1 | AT1G61840.1 \| Symbols: \| DC1 domain-containing protein \| chr1:22847389-22849833 FORWARD |
| miR822.3* | 4 | AT1G14850.1 \| Symbols: NUP155 \| NUP155; nucleocytoplasmic transporter \| chr1:5116696-5123259 REVERSE |
| miR822.3* | 4 | AT1G61830.1 \| Symbols: \| pseudogene, CHP-rich zinc finger protein, putative, similar to putative CHP-rich zinc finger protein GI:9293934 from (Arabidopsis thaliana) \| chr1:22839992-22841860 FORWARD |
| miR822.3* | 4 | AT2G40050.1 \| Symbols: \| DC1 domain-containing protein \| chr2:16723881-16725774 REVERSE |
| miR822.3* | 4.5 | AT1G30160.1 \| Symbols: \| unknown protein \| chr1:10606210-10607810 FORWARD |
| miR822.3* | 4.5 | AT2G04450.1 \| Symbols: ATNUDT6 \| ATNUDT6 (Arabidopsis thaliana Nudix hydrolase homolog 6); ADP-ribose diphosphatase/ NAD or NADH binding / hydrolase \| chr2:1543320-1545462 FORWARD |
| miR822.3* | 4.5 | AT3G21210.1 \| Symbols: \| protein binding / zinc ion binding \| chr3:7437964-7440826 REVERSE |
| miR822.3* | 4.5 | AT5G26180.1 \| Symbols: \| NOL1/NOP2/sun family protein \| chr5:9149227-9153502 FORWARD |
| miR822.4-3p | 3.5 | AT5G18230.1 \| Symbols: \| transcription regulator NOT2/NOT3/NOT5 family protein \| chr5:6021444-6027249 REVERSE |
| miR822.4-3p | 4 | AT1G05615.1 \| Symbols: \| unknown protein \| chr1:1677507-1678270 REVERSE |
| miR822.4-3p | 4 | AT2G44980.1 \| Symbols: \| transcription regulatory protein SNF2, putative \| chr2:18552440-18556669 REVERSE |
| miR822.4-3p | 4.5 | AT2G34970.1 \| Symbols: \| eIF4-gamma/eIF5/eIF2-epsilon domain-containing protein \| chr2:14746307-14748781 FORWARD |
| miR822.4-3p | 4.5 | AT3G16760.1 \| Symbols: \| tetratricopeptide repeat (TPR)-containing protein \| chr3:5703013-5705313 FORWARD |
| miR822.4-3p | 4.5 | AT4G20160.1 \| Symbols: \| LOCATED IN: chloroplast; EXPRESSED IN: 6 plant structures; EXPRESSED DURING: L mature pollen stage, M germinated pollen stage, 4 anthesis, petal differentiation and expansion stage; BEST Arabidopsis thaliana protein match is: protein binding / zinc ion binding (TAIR:AT1G30860.1); Has 56595 Blast hits to 33217 proteins in 1313 species: Archae - 191; Bacteria - 4343; Metazoa - 25320; Fungi - 4691; Plants - 1995; Viruses - 268; Other Eukaryotes - 19787 (source: NCBI BLink). \| chr4:10890983-10894971 FORWARD |
| miR822.4-5p | 1.5 | AT1G62030.1 \| Symbols: \| DC1 domain-containing protein \| chr1:22924250-22926598 REVERSE |
| miR822.4-5p | 2 | AT2G28460.1 \| Symbols: \| protein binding / zinc ion binding \| chr2:12166573-12168874 FORWARD |
| miR822.4-5p | 2 | AT4G02540.1 \| Symbols: \| DC1 domain-containing protein \| chr4:1117154-1119817 FORWARD |
| miR822.4-5p | 2 | AT4G10560.1 \| Symbols: MEE53 \| MEE53 (maternal effect embryo arrest 53); protein binding / zinc ion binding \| chr4:6520771-6522882 REVERSE |
| miR822.4-5p | 2 | AT5G26190.1 \| Symbols: \| DC1 domain-containing protein \| chr5:9153628-9155298 REVERSE |
| miR822.4-5p | 2 | AT5G37210.1 \| Symbols: \| DC1 domain-containing protein \| chr5:14729225-14731382 REVERSE |
| miR822.4-5p | 2 | AT5G40320.1 \| Symbols: \| DC1 domain-containing protein \| chr5:16121757-16123541 FORWARD |
| miR822.4-5p | 2 | AT5G59940.1 \| Symbols: \| DC1 domain-containing protein / UV-B light-insensitive protein, putative \| chr5:24137412-24139367 FORWARD |
| miR822.4-5p | 2.5 | AT1G66440.1 \| Symbols: \| DC1 domain-containing protein \| chr1:24782171-24784351 FORWARD |
| miR822.4-5p | 2.5 | AT1G69150.1 \| Symbols: \| DC1 domain-containing protein \| chr1:25993659-25995212 REVERSE |
| miR822.4-5p | 2.5 | AT3G27095.1 \| Symbols: \| pseudogene, hypothetical protein, similar to putative CHP-rich zinc finger protein GB:AAD22655 from (Arabidopsis thaliana) \| chr3:9991972-9994181 REVERSE |
| miR822.4-5p | 2.5 | AT5G03360.1 \| Symbols: \| DC1 domain-containing protein \| chr5:817954-825149 FORWARD |
| miR822.4-5p | 2.5 | AT5G48320.1 \| Symbols: \| DC1 domain-containing protein \| chr5:19581705-19584748 REVERSE |
| miR822.4-5p | 3 | AT1G45243.1 \| Symbols: \| DC1 domain-containing protein \| chr1:17147058-17147849 FORWARD |
| miR822.4-5p | 3 | AT1G66450.1 \| Symbols: \| DC1 domain-containing protein \| chr1:24786200-24788302 FORWARD |
| miR822.4-5p | 3 | AT2G02680.1 \| Symbols: \| DC1 domain-containing protein \| chr2:745775-748573 FORWARD |
| miR822.4-5p | 3 | AT2G13910.1 \| Symbols: \| pseudogene, CHP-rich zinc finger protein, putative \| chr2:5837686-5839920 FORWARD |
| miR822.4-5p | 3 | AT2G19660.1 \| Symbols: \| DC1 domain-containing protein \| chr2:8496720-8498708 REVERSE |
| miR822.4-5p | 3 | AT2G40050.1 \| Symbols: \| DC1 domain-containing protein \| chr2:16723881-16725774 REVERSE |
| miR822.4-5p | 3 | AT3G28650.1 \| Symbols: \| DC1 domain-containing protein \| chr3:10735729-10737726 FORWARD |
| miR822.4-5p | 3 | AT4G01740.1 \| Symbols: \| DC1 domain-containing protein \| chr4:753422-755456 FORWARD |
| miR822.4-5p | 3 | AT4G01910.1 \| Symbols: \| DC1 domain-containing protein \| chr4:824568-826685 REVERSE |
| miR822.4-5p | 3 | AT4G01930.1 \| Symbols: \| DC1 domain-containing protein \| chr4:838802-840760 REVERSE |
| miR822.4-5p | 3 | AT4G11550.1 \| Symbols: \| DC1 domain-containing protein \| chr4:6996823-6998829 REVERSE |
| miR822.4-5p | 3.5 | AT1G53340.1 \| Symbols: \| DC1 domain-containing protein \| chr1:19898928-19900931 REVERSE |
| miR822.4-5p | 3.5 | AT2G13900.1 \| Symbols: \| DC1 domain-containing protein \| chr2:5833044-5835209 FORWARD |
| miR822.4-5p | 3.5 | AT3G26240.1 \| Symbols: \| DC1 domain-containing protein \| chr3:9603889-9607192 REVERSE |
| miR822.4-5p | 3.5 | AT3G26250.1 \| Symbols: \| DC1 domain-containing protein \| chr3:9611696-9613168 REVERSE |
| miR822.4-5p | 3.5 | AT3G27490.1 \| Symbols: \| DC1 domain-containing protein \| chr3:10178100-10180148 REVERSE |
| miR822.4-5p | 3.5 | AT3G27510.1 \| Symbols: \| protein binding / zinc ion binding \| chr3:10188221-10190343 REVERSE |
| miR822.4-5p | 3.5 | AT3G43890.1 \| Symbols: \| DC1 domain-containing protein \| chr3:15741468-15743453 REVERSE |
| miR822.4-5p | 3.5 | AT3G50010.1 \| Symbols: \| DC1 domain-containing protein \| chr3:18537183-18539601 REVERSE |
| miR822.4-5p | 3.5 | AT4G01925.1 \| Symbols: \| DC1 domain-containing protein \| chr4:833241-834634 REVERSE |
| miR822.4-5p | 3.5 | AT4G15070.1 \| Symbols: \| DC1 domain-containing protein \| chr4:8603424-8606141 REVERSE |
| miR822.4-5p | 3.5 | AT4G35520.1 \| Symbols: MLH3, ATMLH3 \| MLH3 (MUTL PROTEIN HOMOLOG 3); ATP binding / mismatched DNA binding \| chr4:16865488-16871764 FORWARD |
| miR822.4-5p | 3.5 | AT5G02340.1 \| Symbols: \| DC1 domain-containing protein \| chr5:492255-494150 REVERSE |
| miR822.4-5p | 3.5 | AT5G22355.1 \| Symbols: \| DC1 domain-containing protein \| chr5:7401707-7403825 FORWARD |
| miR822.4-5p | 3.5 | AT5G39471.1 \| Symbols: \| protein binding / zinc ion binding \| chr5:15793965-15795596 REVERSE |
| miR822.4-5p | 3.5 | AT5G42840.1 \| Symbols: \| DC1 domain-containing protein \| chr5:17179158-17181173 FORWARD |
| miR822.4-5p | 4 | AT1G54370.1 \| Symbols: NHX5, ATNHX5 \| NHX5; sodium ion transmembrane transporter/ sodium:hydrogen antiporter \| chr1:20293151-20297133 REVERSE |
| miR822.4-5p | 4 | AT1G61830.1 \| Symbols: \| pseudogene, CHP-rich zinc finger protein, putative, similar to putative CHP-rich zinc finger protein GI:9293934 from (Arabidopsis thaliana) \| chr1:22839992-22841860 FORWARD |
| miR822.4-5p | 4 | AT2G02610.1 \| Symbols: \| DC1 domain-containing protein \| chr2:710712-712819 FORWARD |
| miR822.4-5p | 4 | AT2G02630.1 \| Symbols: \| DC1 domain-containing protein \| chr2:723565-725620 FORWARD |
| miR822.4-5p | 4 | AT2G02640.1 \| Symbols: \| DC1 domain-containing protein \| chr2:732388-734271 FORWARD |
| miR822.4-5p | 4 | AT2G04680.1 \| Symbols: \| DC1 domain-containing protein \| chr2:1640140-1642209 FORWARD |
| miR822.4-5p | 4 | AT3G26550.1 \| Symbols: \| DC1 domain-containing protein \| chr3:9746895-9749046 REVERSE |
| miR822.4-5p | 4 | AT3G27480.1 \| Symbols: \| DC1 domain-containing protein \| chr3:10173640-10175555 REVERSE |
| miR822.4-5p | 4 | AT4G01350.1 \| Symbols: \| protein binding / zinc ion binding \| chr4:560259-562438 FORWARD |
| miR822.4-5p | 4 | AT4G01920.1 \| Symbols: \| DC1 domain-containing protein \| chr4:828890-831200 REVERSE |
| miR822.4-5p | 4 | AT4G02190.1 \| Symbols: \| DC1 domain-containing protein \| chr4:967372-969351 FORWARD |
| miR822.4-5p | 4 | AT4G11540.1 \| Symbols: \| DC1 domain-containing protein \| chr4:6990978-6992555 REVERSE |
| miR822.4-5p | 4 | AT4G19210.1 \| Symbols: ATRLI2, RLI2 \| ATRLI2; transporter \| chr4:10501200-10505217 FORWARD |
| miR822.4-5p | 4 | AT5G54020.1 \| Symbols: \| zinc ion binding \| chr5:21921864-21923534 REVERSE |
| miR822.4-5p | 4 | AT5G55770.1 \| Symbols: \| DC1 domain-containing protein \| chr5:22571645-22573732 FORWARD |
| miR822.4-5p | 4 | AT5G55780.1 \| Symbols: \| DC1 domain-containing protein \| chr5:22576611-22578668 FORWARD |
| miR822.4-5p | 4 | AT5G59930.1 \| Symbols: \| DC1 domain-containing protein / UV-B light-insensitive protein, putative \| chr5:24134526-24136496 FORWARD |
| miR822.4-5p | 4.5 | AT1G55420.1 \| Symbols: EDA11 \| EDA11 (embryo sac development arrest 11); protein binding / zinc ion binding \| chr1:20691775-20693952 REVERSE |
| miR822.4-5p | 4.5 | AT1G74210.1 \| Symbols: \| glycerophosphoryl diester phosphodiesterase family protein \| chr1:27910314-27912941 FORWARD |
| miR822.4-5p | 4.5 | AT2G02700.1 \| Symbols: \| DC1 domain-containing protein \| chr2:756443-757942 FORWARD |
| miR822.4-5p | 4.5 | AT2G19780.1 \| Symbols: \| leucine-rich repeat family protein / extensin family protein \| chr2:8522678-8525093 REVERSE |
| miR822.4-5p | 4.5 | AT2G22125.1 \| Symbols: \| binding \| chr2:9406263-9414518 FORWARD |
| miR822.4-5p | 4.5 | AT3G27500.1 \| Symbols: \| protein binding / zinc ion binding \| chr3:10182833-10185081 REVERSE |
| miR822.4-5p | 4.5 | AT3G45530.1 \| Symbols: \| DC1 domain-containing protein \| chr3:16701133-16703366 REVERSE |
| miR822.4-5p | 4.5 | AT3G48400.1 \| Symbols: \| DC1 domain-containing protein \| chr3:17923678-17925589 FORWARD |
| miR822.4-5p | 4.5 | AT3G59120.1 \| Symbols: \| DC1 domain-containing protein \| chr3:21857876-21860221 REVERSE |
| miR822.4-5p | 4.5 | AT4G13130.1 \| Symbols: \| DC1 domain-containing protein \| chr4:7647389-7649831 FORWARD |
| miR822.4-5p | 4.5 | AT5G02360.1 \| Symbols: \| DC1 domain-containing protein \| chr5:500626-502329 REVERSE |
| miR822.4-5p | 4.5 | AT5G03880.1 \| Symbols: \| electron carrier \| chr5:1038508-1041480 REVERSE |
| miR822.4-5p | 4.5 | AT5G49650.1 \| Symbols: \| xylulose kinase, putative \| chr5:20152781-20155768 FORWARD |
| miR822.4-5p | 4.5 | AT5G54030.1 \| Symbols: \| DC1 domain-containing protein \| chr5:21925984-21927243 REVERSE |
| miR822.5 | 1 | AT2G13900.1 \| Symbols: \| DC1 domain-containing protein \| chr2:5833044-5835209 FORWARD |
| miR822.5 | 1 | AT5G02350.1 \| Symbols: \| DC1 domain-containing protein \| chr5:495311-497266 REVERSE |
| miR822.5 | 1.5 | AT5G02360.1 \| Symbols: \| DC1 domain-containing protein \| chr5:500626-502329 REVERSE |
| miR822.5 | 2 | AT2G13910.1 \| Symbols: \| pseudogene, CHP-rich zinc finger protein, putative \| chr2:5837686-5839920 FORWARD |
| miR822.5 | 3 | AT5G02330.1 \| Symbols: \| DC1 domain-containing protein \| chr5:487272-489242 REVERSE |
| miR822.5 | 3 | AT5G02340.1 \| Symbols: \| DC1 domain-containing protein \| chr5:492255-494150 REVERSE |
| miR822.5 | 4 | AT1G60440.1 \| Symbols: ATCOAA, ATPANK1 \| ATPANK1 (PANTOTHENATE KINASE 1); pantothenate kinase \| chr1:22266497-22269243 REVERSE |
| miR822.5 | 4 | AT1G62030.1 \| Symbols: \| DC1 domain-containing protein \| chr1:22924250-22926598 REVERSE |
| miR822.5 | 4 | AT3G11410.1 \| Symbols: ATPP2CA, AHG3, PP2CA \| PP2CA (ARABIDOPSIS THALIANA PROTEIN PHOSPHATASE 2CA); protein binding / protein serine/threonine phosphatase \| chr3:3583883-3585790 REVERSE |
| miR822.5 | 4 | AT5G07010.1 \| Symbols: ATST2A, ST2A \| ST2A (SULFOTRANSFERASE 2A); hydroxyjasmonate sulfotransferase/ sulfotransferase \| chr5:2174678-2176106 REVERSE |
| miR822.5 | 4.5 | AT2G22980.1 \| Symbols: SCPL13 \| serine-type carboxypeptidase \| chr2:9778877-9783251 FORWARD |
| miR822.5 | 4.5 | AT2G40530.1 \| Symbols: \| unknown protein \| chr2:16927502-16928208 FORWARD |
| miR839.2 | 3.5 | AT3G27670.1 \| Symbols: RST1 \| RST1 (RESURRECTION1); binding \| chr3:10245134-10253293 FORWARD |
| miR839.2 | 3.5 | AT5G59410.1 \| Symbols: \| FUNCTIONS IN: molecular_function unknown; INVOLVED IN: biological_process unknown; LOCATED IN: cellular_component unknown; EXPRESSED IN: 22 plant structures; EXPRESSED DURING: 13 growth stages; CONTAINS InterPro DOMAIN/s: Rab5-interacting (InterPro:IPR010742); BEST Arabidopsis thaliana protein match is: Rab5-interacting family protein (TAIR:AT2G29020.1); Has 154 Blast hits to 154 proteins in 67 species: Archae - 0; Bacteria - 0; Metazoa - 108; Fungi - 0; Plants - 26; Viruses - 0; Other Eukaryotes - 20 (source: NCBI BLink). \| chr5:23959573-23960878 REVERSE |
| miR839.2 | 4 | AT2G33815.1 \| Symbols: \| other RNA \| chr2:14305008-14306053 REVERSE |
| miR839.2 | 4 | AT3G27600.1 \| Symbols: \| RNA binding \| chr3:10223743-10224948 REVERSE |
| miR839.2 | 4.5 | AT2G37150.1 \| Symbols: \| zinc finger (C3HC4-type RING finger) family protein \| chr2:15603584-15608454 REVERSE |
| miR839.2 | 4.5 | AT3G61690.1 \| Symbols: \| unknown protein \| chr3:22827884-22834048 FORWARD |
| miR839.2 | 4.5 | AT4G31210.1 \| Symbols: \| DNA topoisomerase family protein \| chr4:15165280-15172687 FORWARD |
| miR839.2 | 4.5 | AT5G14420.1 \| Symbols: RGLG2 \| RGLG2 (RING domain Ligase2); ubiquitin-protein ligase \| chr5:4648111-4650698 REVERSE |
| miR839.2 | 4.5 | AT5G55320.1 \| Symbols: \| membrane bound O-acyl transferase (MBOAT) family protein / wax synthase-related \| chr5:22435680-22436699 REVERSE |
| miR839.3 | 3.5 | AT3G47890.1 \| Symbols: \| ubiquitin thiolesterase/ zinc ion binding \| chr3:17666927-17673530 REVERSE |
| miR839.3 | 4.5 | AT1G65960.1 \| Symbols: GAD2 \| GAD2 (GLUTAMATE DECARBOXYLASE 2); calmodulin binding / glutamate decarboxylase \| chr1:24554413-24557431 FORWARD |
| miR839.3 | 4.5 | AT3G47910.1 \| Symbols: \| ubiquitin thiolesterase/ zinc ion binding \| chr3:17674382-17681730 REVERSE |
| miR841.2 | 0 | AT4G13570.1 \| Symbols: HTA4 \| HTA4; DNA binding \| chr4:7884516-7885664 FORWARD |
| miR841.2 | 2 | AT4G04408.1 \| Symbols: \| pseudogene, similar to Putative histone H2A, blastp match of 79% identity and 3.0e-31 P-value to GP\|22773254\|gb\|AAN06860.1\|\|AC105729 Putative histone H2A {Oryza sativa (japonica cultivar-group)} \| chr4:2184059-2184400 REVERSE |
| miR841.2 | 4 | AT1G64355.1 \| Symbols: \| unknown protein \| chr1:23886084-23887212 FORWARD |
| miR841.2 | 4 | AT2G31870.1 \| Symbols: TEJ \| TEJ (Sanskrit for 'bright'); poly(ADP-ribose) glycohydrolase \| chr2:13549999-13553843 REVERSE |
| miR841.2 | 4 | AT3G27950.1 \| Symbols: \| early nodule-specific protein, putative \| chr3:10378048-10379896 FORWARD |
| miR841.2 | 4 | AT5G27390.1 \| Symbols: \| LOCATED IN: chloroplast; CONTAINS InterPro DOMAIN/s: Mog1/PsbP/DUF1795, alpha/beta/alpha sandwich (InterPro:IPR016124); Has 11 Blast hits to 11 proteins in 5 species: Archae - 0; Bacteria - 0; Metazoa - 0; Fungi - 0; Plants - 11; Viruses - 0; Other Eukaryotes - 0 (source: NCBI BLink). \| chr5:9674391-9676189 REVERSE |
| miR841.2 | 4.5 | AT1G65730.1 \| Symbols: YSL7 \| YSL7 (YELLOW STRIPE LIKE 7); oligopeptide transporter \| chr1:24442504-24446291 FORWARD |
| miR841.2 | 4.5 | AT2G15330.1 \| Symbols: \| transposable element gene \| chr2:6672892-6673989 REVERSE |
| miR841.2 | 4.5 | AT4G15475.1 \| Symbols: \| F-box family protein (FBL4) \| chr4:8845875-8848869 FORWARD |
| miR841.2 | 4.5 | AT4G19160.1 \| Symbols: \| unknown protein \| chr4:10477306-10480120 FORWARD |
| miR841.2 | 4.5 | AT4G34570.1 \| Symbols: THY-2 \| THY-2 (thymidylate synthase 2); dihydrofolate reductase/ thymidylate synthase \| chr4:16511001-16514343 REVERSE |
| miR841.2 | 4.5 | AT4G36540.1 \| Symbols: BEE2 \| BEE2 (BR Enhanced Expression 2); DNA binding / transcription factor \| chr4:17243583-17245296 FORWARD |
| miR841.2 | 4.5 | AT5G17890.1 \| Symbols: DAR4 \| DAR4 (DA1-RELATED PROTEIN 4); ATP binding / protein binding / zinc ion binding \| chr5:5916970-5923355 FORWARD |
| miR841.2 | 4.5 | AT5G65200.1 \| Symbols: PUB38, ATPUB38 \| PUB38 (PLANT U-BOX 38); ubiquitin-protein ligase \| chr5:26048065-26049952 REVERSE |
| miR846.2 | 4.5 | AT2G04420.1 \| Symbols: \| nucleic acid binding \| chr2:1535489-1536250 FORWARD |
| miR846.2 | 4.5 | AT3G33004.1 \| Symbols: \| pseudogene, DNA-directed RNA polymerase beta-chain \| chr3:13538953-13541488 REVERSE |
| miR846.2 | 4.5 | AT4G21690.1 \| Symbols: ATGA3OX3, GA3OX3 \| GA3OX3 (GIBBERELLIN 3-OXIDASE 3); iron ion binding / oxidoreductase \| chr4:11527229-11529060 FORWARD |
| miR846.2 | 4.5 | AT5G31032.1 \| Symbols: \| transposable element gene \| chr5:11412727-11415675 FORWARD |
| miR846.2* | 3.5 | AT5G09870.1 \| Symbols: CESA5 \| CESA5 (CELLULOSE SYNTHASE 5); cellulose synthase/ transferase, transferring glycosyl groups \| chr5:3073356-3078262 FORWARD |
| miR846.2* | 4 | AT1G12620.1 \| Symbols: \| pentatricopeptide (PPR) repeat-containing protein \| chr1:4294594-4297084 REVERSE |
| miR846.2* | 4 | AT4G27650.1 \| Symbols: PEL1 \| PEL1 (PELOTA); translation release factor \| chr4:13803297-13807935 REVERSE |
| miR846.2* | 4 | AT5G48830.1 \| Symbols: \| unknown protein \| chr5:19800114-19803015 REVERSE |
| miR846.2* | 4.5 | AT1G15390.1 \| Symbols: PDF1A, ATDEF1 \| PDF1A (PEPTIDE DEFORMYLASE 1A); peptide deformylase \| chr1:5294629-5295797 FORWARD |
| miR846.2* | 4.5 | AT3G29773.1 \| Symbols: \| pseudogene, similar to OSJNBb0043H09.1, blastp match of 34% identity and 6.6e-21 P-value to GP\|21740634\|emb\|CAD40195.1\|\|AL606611 OSJNBb0043H09.1 {Oryza sativa (japonica cultivar-group)} \| chr3:11627225-11630679 FORWARD |
| miR846.2* | 4.5 | AT3G50890.1 \| Symbols: AtHB28 \| AtHB28 (ARABIDOPSIS THALIANA HOMEOBOX PROTEIN 28); DNA binding / transcription factor \| chr3:18916166-18917328 FORWARD |
| miR846.2* | 4.5 | AT4G18090.1 \| Symbols: \| unknown protein \| chr4:10034011-10034847 REVERSE |
| miR846.2* | 4.5 | AT4G22185.1 \| Symbols: \| pseudogene, similar to P0537A05.16, similar to protein; blastp match of 28% identity and 3.1e-07 P-value to GP\|13366010\|dbj\|BAB39287.1\|\|AP002971 P0537A05.16 {Oryza sativa (japonica cultivar-group)} \| chr4:11740053-11741234 FORWARD |

**Supplemental Table 3.** Six miRNA-like RNAs found in the current study and their mRNA targets identified in small RNA degradome data. In the table, the first two columns list the miRNA-like RNAs and their targets, the third to fifth columns list the three major quantitative measures of the results, i.e., the alignment scores, the number of raw reads of target degradation products and p-values quantifying the enrichment of degradation products, respectively. The last column indicates if a pair of miRNA-like RNA and target was tested in the current study.

| **miR-like RNA** | **target gene** | **alignment score** | **# raw reads** | **p-value** | **experiment** |
| --- | --- | --- | --- | --- | --- |
| miR159a.2* | AT5G24620.1 | 3 | 1 | 0.174 |  |
| miR169b.2 | AT4G17420.1 | 3.5 | 1 | 0.198 |  |
| miR169i.2* | AT5G02710.1 | 4.5 | 10 | 0.019 | yes |
| miR169j.2 | AT5G48300.1 | 3 | 2 | 0.112 | yes |
| miR822.4-5p | AT1G62030.1 | 1.5 | 3 | 0.002 | yes |
| miR822.4-5p | AT2G04680.1 | 4 | 3 | 0.006 | yes |
| miR839.3 | AT1G65960.1 | 4.5 | 2 | 0.026 | yes |
