# Supplementary material for: Multiple distinct small RNAs originate from the same microRNA precursors
Source: Genome Biol. 2010 Aug 9;11(8):R81. doi: 10.1186/gb-2010-11-8-r81 (PMC2945783; doi:10.1186/gb-2010-11-8-r81)
Supplement: Additional file 2 — Supplemental File S1. This is a file for sequencing reads mapped and aligned to miRNA precursors that can produce miRNA-sibling small RNAs (msRNAs) in A. thaliana (ath). The sequencing data were collected in the current study and are available in GEO under accession number [GEO:GSE19694]. [file gb-2010-11-8-r81-S2.DOCX]

Zhang, et al., Multiple distinct small RNAs originate from the same microRNA precursors

Supplemental File 1 - Sequencing reads mapped and aligned to miRNA precursors that can produce

miRNA-like RNAs in *Arabidopsis thaliana.*

*Data source – sequencing data collected in the current study, available at NCBI/GEO accession number* GSE19694.

>ath-MIR159a

GUAGAGCUCCUUAAAGUUCAAACAUGAGUUGAGCAGGGUAAAGAAAAGCUGCUAAGCUAUGGAUCCCAUAAGCCCUAAUCCUUGUAAAGUAAAAAAGGAUUUGGUUAUAUGGAUUGCAUAUCUCAGGAGCUUUAACUUGCCCUUUAAUGGCUUUUACUCUUCUUUGGAUUGAAGGGAGCUCUAC

((((((((((((..((((((((...((((.(((((((((((...((((((.((.((.((((.(..(((((((((..(((((((...........))))))).)))).)))))..).)))).)).)).))))))...)))))))......))))..))))...))))))))..)))))))))))) (-75.90)

...GAGCTCCTTAAAGTTCAAACA................................................................................................................................................................ 8

....AGCTCCTTAAAGTTCAAACA................................................................................................................................................................ 1

........................TGAGTTGAGCAGGGTAAAGAAA.......................................................................................................................................... 1

........................TGAGTTGAGCAGGGTAAAGAAAA......................................................................................................................................... 1

............................................AAAGCTGCTAAGCTATGGATCC...................................................................................................................... 2

..............................................AGCTGCTAAGCTATGGATC....................................................................................................................... 4

..............................................AGCTGCTAAGCTATGGATCC...................................................................................................................... 4

..............................................AGCTGCTAAGCTATGGATCCC..................................................................................................................... 24

................................................CTGCTAAGCTATGGATCCC..................................................................................................................... 2

........................................................................................GTAAAAAAGGATTTGGTTATATGG........................................................................ 2

.........................................................................................TAAAAAAGGATTTGGTTATA........................................................................... 2

..........................................................................................AAAAAAGGATTTGGTTATA........................................................................... 1

..........................................................................................................ATATGGATTGCATATCTCAGG......................................................... 1

................................................................................................................ATTGCATATCTCAGGAGCTT.................................................... 1

................................................................................................................ATTGCATATCTCAGGAGCTTT................................................... 4

................................................................................................................ATTGCATATCTCAGGAGCTTTA.................................................. 1

.................................................................................................................TTGCATATCTCAGGAGCTTTA.................................................. 3

.............................................................................................................................................................TCTTCTTTGGATTGAAGGGA....... 2

..............................................................................................................................................................CTTCTTTGGATTGAAGGGAGC..... 8

...............................................................................................................................................................TTCTTTGGATTGAAGGGAGCT.... 2

................................................................................................................................................................TCTTTGGATTGAAGGGAGC..... 4

................................................................................................................................................................TCTTTGGATTGAAGGGAGCT.... 2

................................................................................................................................................................TCTTTGGATTGAAGGGAGCTC... 26

.................................................................................................................................................................CTTTGGATTGAAGGGAGCT.... 4

..................................................................................................................................................................TTTGGATTGAAGGGAGCTC... 1302

..................................................................................................................................................................TTTGGATTGAAGGGAGCTCT.. 446

..................................................................................................................................................................TTTGGATTGAAGGGAGCTCTA. 4429

..................................................................................................................................................................TTTGGATTGAAGGGAGCTCTAC 15

...................................................................................................................................................................TTGGATTGAAGGGAGCTCT.. 20

...................................................................................................................................................................TTGGATTGAAGGGAGCTCTA. 248

....................................................................................................................................................................TGGATTGAAGGGAGCTCTA. 89

>ath-MIR159b

GGAAGAGCUCCUUGAAGUUCAAUGGAGGGUUUAGCAGGGUGAAGUAAAGCUGCUAAGCUAUGGAUCCCAUAAGCCUUAUCAAAUUCAAUAUAAUUGAUGAUAAGGUUUUUUUUAUGGAUGCCAUAUCUCAGGAGCUUUCACUUACCCCUUUAAUGGCUUCACUCUUCUUUGGAUUGAAGGGAGCUCUUCAUCUCUC

.((((((((((((..(((((((.(((((((..(((((((..((((((((((.((.((.(((((...(((((((((((((((...(((((...)))))))))))))).....))))))...))))).)).)).)))))).))))..))))......)))..))))))).)))))))..))))))))))))....... (-85.60)

....GAGCTCCTTGAAGTTCAAT............................................................................................................................................................................. 1

....GAGCTCCTTGAAGTTCAATGG........................................................................................................................................................................... 2

.....AGCTCCTTGAAGTTCAATGGA.......................................................................................................................................................................... 1

.........................AGGGTTTAGCAGGGTGAAGTAA..................................................................................................................................................... 1

.............................................AAAGCTGCTAAGCTATGGATCC................................................................................................................................. 2

...............................................AGCTGCTAAGCTATGGATC.................................................................................................................................. 4

...............................................AGCTGCTAAGCTATGGATCC................................................................................................................................. 4

...............................................AGCTGCTAAGCTATGGATCCC................................................................................................................................ 24

.................................................CTGCTAAGCTATGGATCCC................................................................................................................................ 2

................................................................................................................TATGGATGCCATATCTCAGGA............................................................... 1

.....................................................................................................................ATGCCATATCTCAGGAGCT............................................................ 1

.....................................................................................................................ATGCCATATCTCAGGAGCTT........................................................... 1

.....................................................................................................................ATGCCATATCTCAGGAGCTTT.......................................................... 11

......................................................................................................................TGCCATATCTCAGGAGCTTTC......................................................... 1

..................................................................................................................................................................TCTTCTTTGGATTGAAGGGA.............. 2

...................................................................................................................................................................CTTCTTTGGATTGAAGGGAGC............ 8

....................................................................................................................................................................TTCTTTGGATTGAAGGGAGCT........... 2

.....................................................................................................................................................................TCTTTGGATTGAAGGGAGC............ 4

.....................................................................................................................................................................TCTTTGGATTGAAGGGAGCT........... 2

.....................................................................................................................................................................TCTTTGGATTGAAGGGAGCTC.......... 26

......................................................................................................................................................................CTTTGGATTGAAGGGAGCT........... 4

.......................................................................................................................................................................TTTGGATTGAAGGGAGCTC.......... 1302

.......................................................................................................................................................................TTTGGATTGAAGGGAGCTCT......... 446

.......................................................................................................................................................................TTTGGATTGAAGGGAGCTCTT........ 602

.......................................................................................................................................................................TTTGGATTGAAGGGAGCTCTTC....... 10

........................................................................................................................................................................TTGGATTGAAGGGAGCTCT......... 20

........................................................................................................................................................................TTGGATTGAAGGGAGCTCTT........ 52

.........................................................................................................................................................................TGGATTGAAGGGAGCTCTT........ 10

.........................................................................................................................................................................TGGATTGAAGGGAGCTCTTC....... 1

>ath-MIR168a

CACCAUCGGGCUCGGAUUCGCUUGGUGCAGGUCGGGAACCAAUUCGGCUGACACAGCCUCGUGACUUUUAAACCUUUAUUGGUUUGUGAGCAGGGAUUGGAUCCCGCCUUGCAUCAACUGAAUCGGAUCCUCGAGGUG

((((.(((((.((.((((((.((((((((((.(((((.((((((((((((...)))))...((.(((.((((((......)))))).))))).))))))).))))).)))))))))).)))))).)).)).))))))) (-69.60)

...............ATTCGCTTGGTGCAGGTCGGGAA.................................................................................................... 2

...............ATTCGCTTGGTGCAGGTCGGGAAC................................................................................................... 8

................TTCGCTTGGTGCAGGTCGGGA..................................................................................................... 68

................TTCGCTTGGTGCAGGTCGGGAA.................................................................................................... 40

................TTCGCTTGGTGCAGGTCGGGAAC................................................................................................... 2

................TTCGCTTGGTGCAGGTCGGGAACC.................................................................................................. 2

.................TCGCTTGGTGCAGGTCGGG...................................................................................................... 2916

.................TCGCTTGGTGCAGGTCGGGA..................................................................................................... 2514

.................TCGCTTGGTGCAGGTCGGGAA.................................................................................................... 234030

.................TCGCTTGGTGCAGGTCGGGAAC................................................................................................... 15140

.................TCGCTTGGTGCAGGTCGGGAACC.................................................................................................. 116

.................TCGCTTGGTGCAGGTCGGGAACCA................................................................................................. 56

..................CGCTTGGTGCAGGTCGGGA..................................................................................................... 74

..................CGCTTGGTGCAGGTCGGGAA.................................................................................................... 8866

..................CGCTTGGTGCAGGTCGGGAAC................................................................................................... 1952

..................CGCTTGGTGCAGGTCGGGAACC.................................................................................................. 39

..................CGCTTGGTGCAGGTCGGGAACCA................................................................................................. 23

..................CGCTTGGTGCAGGTCGGGAACCAA................................................................................................ 5

...................GCTTGGTGCAGGTCGGGAA.................................................................................................... 70

...................GCTTGGTGCAGGTCGGGAAC................................................................................................... 24

...................GCTTGGTGCAGGTCGGGAACC.................................................................................................. 1

...................GCTTGGTGCAGGTCGGGAACCA................................................................................................. 1

...................GCTTGGTGCAGGTCGGGAACCAAT............................................................................................... 1

....................CTTGGTGCAGGTCGGGAAC................................................................................................... 40

....................CTTGGTGCAGGTCGGGAACCA................................................................................................. 24

.....................TTGGTGCAGGTCGGGAACC.................................................................................................. 1

......................TGGTGCAGGTCGGGAACCAA................................................................................................ 1

......................TGGTGCAGGTCGGGAACCAAT............................................................................................... 4

.........................TGCAGGTCGGGAACCAATTCGGC.......................................................................................... 1

..........................GCAGGTCGGGAACCAATTCGG........................................................................................... 1

............................AGGTCGGGAACCAATTCGGCT......................................................................................... 2

............................AGGTCGGGAACCAATTCGGCTG........................................................................................ 1

............................AGGTCGGGAACCAATTCGGCTGA....................................................................................... 1

......................................CCAATTCGGCTGACACAGCCTCGTGACTTT...................................................................... 1

...........................................................................TTATTGGTTTGTGAGCAGGGATTGGAT.................................... 1

............................................................................TATTGGTTTGTGAGCAGGGATTGGAT.................................... 1

.............................................................................ATTGGTTTGTGAGCAGGGATTGGAT.................................... 2

..............................................................................TTGGTTTGTGAGCAGGGATTGGA..................................... 1

..............................................................................TTGGTTTGTGAGCAGGGATTGGAT.................................... 1

...............................................................................TGGTTTGTGAGCAGGGATTGG...................................... 1

...............................................................................TGGTTTGTGAGCAGGGATTGGAT.................................... 2

.................................................................................GTTTGTGAGCAGGGATTGGAT.................................... 1

...........................................................................................AGGGATTGGATCCCGCCTTGCATC....................... 1

................................................................................................TTGGATCCCGCCTTGCATCAA..................... 5

.................................................................................................TGGATCCCGCCTTGCATCAAC.................... 4

.................................................................................................TGGATCCCGCCTTGCATCAACT................... 1

...................................................................................................GATCCCGCCTTGCATCAAC.................... 1

...................................................................................................GATCCCGCCTTGCATCAACT................... 1

...................................................................................................GATCCCGCCTTGCATCAACTGA................. 3

...................................................................................................GATCCCGCCTTGCATCAACTGAA................ 10

...................................................................................................GATCCCGCCTTGCATCAACTGAAT............... 38

....................................................................................................ATCCCGCCTTGCATCAACT................... 1

....................................................................................................ATCCCGCCTTGCATCAACTGA................. 7

....................................................................................................ATCCCGCCTTGCATCAACTGAA................ 8

....................................................................................................ATCCCGCCTTGCATCAACTGAAT............... 11

.....................................................................................................TCCCGCCTTGCATCAACTG.................. 4

.....................................................................................................TCCCGCCTTGCATCAACTGA................. 3

.....................................................................................................TCCCGCCTTGCATCAACTGAA................ 19

.....................................................................................................TCCCGCCTTGCATCAACTGAAT............... 28

......................................................................................................CCCGCCTTGCATCAACTGA................. 14

......................................................................................................CCCGCCTTGCATCAACTGAA................ 62

......................................................................................................CCCGCCTTGCATCAACTGAAT............... 1295

......................................................................................................CCCGCCTTGCATCAACTGAATC.............. 4

......................................................................................................CCCGCCTTGCATCAACTGAATCG............. 2

.......................................................................................................CCGCCTTGCATCAACTGAA................ 3

.......................................................................................................CCGCCTTGCATCAACTGAAT............... 17

.......................................................................................................CCGCCTTGCATCAACTGAATC.............. 1

........................................................................................................CGCCTTGCATCAACTGAAT............... 18

........................................................................................................CGCCTTGCATCAACTGAATCGGAT.......... 1

..........................................................................................................CCTTGCATCAACTGAATCGG............ 4

..........................................................................................................CCTTGCATCAACTGAATCGGA........... 37

..........................................................................................................CCTTGCATCAACTGAATCGGAT.......... 1

..........................................................................................................CCTTGCATCAACTGAATCGGATC......... 1

............................................................................................................TTGCATCAACTGAATCGGA........... 1

>ath-MIR169f

GGGUCUUGCAUGAAGGAAUAACGAAUGGAAUUGAGCCAAGGAUGACUUGCCGGUUUAAACCCAACCGGUUUAUGACCAUUGAUUUGGUCUCAUUCACAAUCUGUUGAUUCGUGUCUGGCAAGUUGACCUUGGCUCUGCUUCGUUCUCUAUUCUUCCAUGUUAGAUUC

((((((.(((((.(((((((..(((((((...(((((((((.(((((((((((..((((((.....)))))).(((((......))))).....(((((((....)))).))).))))))))))).)))))))))...)))))))..))))))).))))).)))))) (-74.10)

..........TGAAGGAATAACGAATGGA.......................................................................................................................................... 5

..........TGAAGGAATAACGAATGGAA......................................................................................................................................... 4

..........TGAAGGAATAACGAATGGAAT........................................................................................................................................ 98

...........GAAGGAATAACGAATGGAA......................................................................................................................................... 1

.......................AATGGAATTGAGCCAAGGATG........................................................................................................................... 1

........................ATGGAATTGAGCCAAGGATGA.......................................................................................................................... 1

.............................ATTGAGCCAAGGATGACTTGCC.................................................................................................................... 3

.............................ATTGAGCCAAGGATGACTTGCCG................................................................................................................... 3

..............................TTGAGCCAAGGATGACTTGCC.................................................................................................................... 16

..............................TTGAGCCAAGGATGACTTGCCG................................................................................................................... 16

...............................TGAGCCAAGGATGACTTGC..................................................................................................................... 8

...............................TGAGCCAAGGATGACTTGCC.................................................................................................................... 20

...............................TGAGCCAAGGATGACTTGCCG................................................................................................................... 4940

...............................TGAGCCAAGGATGACTTGCCGG.................................................................................................................. 2

...............................TGAGCCAAGGATGACTTGCCGGT................................................................................................................. 1

...............................TGAGCCAAGGATGACTTGCCGGTT................................................................................................................ 6

................................GAGCCAAGGATGACTTGCCG................................................................................................................... 56

................................GAGCCAAGGATGACTTGCCGG.................................................................................................................. 4

.................................AGCCAAGGATGACTTGCCG................................................................................................................... 203

.................................AGCCAAGGATGACTTGCCGG.................................................................................................................. 416

.................................AGCCAAGGATGACTTGCCGGT................................................................................................................. 64

.................................AGCCAAGGATGACTTGCCGGTT................................................................................................................ 11

..................................GCCAAGGATGACTTGCCGG.................................................................................................................. 16

...............................................................................................ACAATCTGTTGATTCGTGTCTGG................................................. 1

..................................................................................................................CTGGCAAGTTGACCTTGGCTCTGC............................. 3

....................................................................................................................GGCAAGTTGACCTTGGCTCTG.............................. 3

.....................................................................................................................GCAAGTTGACCTTGGCTCT............................... 204

.....................................................................................................................GCAAGTTGACCTTGGCTCTG.............................. 258

.....................................................................................................................GCAAGTTGACCTTGGCTCTGC............................. 1956

.....................................................................................................................GCAAGTTGACCTTGGCTCTGCT............................ 38

.....................................................................................................................GCAAGTTGACCTTGGCTCTGCTT........................... 3

.....................................................................................................................GCAAGTTGACCTTGGCTCTGCTTC.......................... 1

......................................................................................................................CAAGTTGACCTTGGCTCTG.............................. 6

......................................................................................................................CAAGTTGACCTTGGCTCTGC............................. 28

......................................................................................................................CAAGTTGACCTTGGCTCTGCTT........................... 1

.......................................................................................................................AAGTTGACCTTGGCTCTGC............................. 2

.......................................................................................................................AAGTTGACCTTGGCTCTGCT............................ 2

>ath-MIR169i

GAAGGAGAUGUCAAAGAUGAAUAGAAGAAUCAUAUUUGGUAGCCAAGGAUGACUUGCCUGACUCUUUGUGUAAAAUGUUUAGUGUCUUGUUUGAAGUCACUAUAAGUUGUAUCAAGCAAUGACCAUUUUGCUUAUAAAAAAGAUAUCAGGCAGUCUCCUUGGCUAUCCUUAUAUGUUCUUCUCUUUCAUCUCAGACAUUCACCUUC

(((((.((((((..(((((((.(((((((.(((((..((((((((((((.((((.((((((.(((((..((((((((..(((((.(((.....))).)))))...(((((.....)))))...)))))))).......)))))..)))))))))))))))))))).))..))))))))))))..)))))))..))))))..))))) (-88.50)

................ATGAATAGAAGAATCATATTTGG....................................................................................................................................................................... 2

.................TGAATAGAAGAATCATATTTG........................................................................................................................................................................ 2

.................TGAATAGAAGAATCATATTTGG....................................................................................................................................................................... 28

.....................................GGTAGCCAAGGATGACTTGC..................................................................................................................................................... 4

.....................................GGTAGCCAAGGATGACTTGCC.................................................................................................................................................... 4

.....................................GGTAGCCAAGGATGACTTGCCT................................................................................................................................................... 16

.....................................GGTAGCCAAGGATGACTTGCCTG.................................................................................................................................................. 16

......................................GTAGCCAAGGATGACTTGCCT................................................................................................................................................... 12

......................................GTAGCCAAGGATGACTTGCCTG.................................................................................................................................................. 48

......................................GTAGCCAAGGATGACTTGCCTGA................................................................................................................................................. 6

.......................................TAGCCAAGGATGACTTGCC.................................................................................................................................................... 833

.......................................TAGCCAAGGATGACTTGCCT................................................................................................................................................... 350

.......................................TAGCCAAGGATGACTTGCCTG.................................................................................................................................................. 37758

.......................................TAGCCAAGGATGACTTGCCTGA................................................................................................................................................. 4936

.......................................TAGCCAAGGATGACTTGCCTGAC................................................................................................................................................ 10

........................................AGCCAAGGATGACTTGCCT................................................................................................................................................... 42

........................................AGCCAAGGATGACTTGCCTG.................................................................................................................................................. 357

........................................AGCCAAGGATGACTTGCCTGA................................................................................................................................................. 64

........................................AGCCAAGGATGACTTGCCTGAC................................................................................................................................................ 1

.........................................GCCAAGGATGACTTGCCTGA................................................................................................................................................. 4

..........................................CCAAGGATGACTTGCCTGA................................................................................................................................................. 16

................................................................................................................................................ATCAGGCAGTCTCCTTGGCTA......................................... 3

.................................................................................................................................................TCAGGCAGTCTCCTTGGCTA......................................... 10

..................................................................................................................................................CAGGCAGTCTCCTTGGCTAT........................................ 10

..................................................................................................................................................CAGGCAGTCTCCTTGGCTATC....................................... 4

...................................................................................................................................................AGGCAGTCTCCTTGGCTATC....................................... 88

...................................................................................................................................................AGGCAGTCTCCTTGGCTATCC...................................... 12

....................................................................................................................................................GGCAGTCTCCTTGGCTATC....................................... 108

....................................................................................................................................................GGCAGTCTCCTTGGCTATCC...................................... 48

....................................................................................................................................................GGCAGTCTCCTTGGCTATCCT..................................... 3

.....................................................................................................................................................GCAGTCTCCTTGGCTATCC...................................... 9

.....................................................................................................................................................GCAGTCTCCTTGGCTATCCT..................................... 21

.....................................................................................................................................................GCAGTCTCCTTGGCTATCCTT.................................... 3

......................................................................................................................................................CAGTCTCCTTGGCTATCCT..................................... 30

......................................................................................................................................................CAGTCTCCTTGGCTATCCTT.................................... 15

........................................................................................................................................................................TTATATGTTCTTCTCTTTCATC................ 9

>ath-MIR319a

AGAGAGAGCUUCCUUGAGUCCAUUCACAGGUCGUGAUAUGAUUCAAUUAGCUUCCGACUCAUUCAUCCAAAUACCGAGUCGCCAAAAUUCAAACUAGACUCGUUAAAUGAAUGAAUGAUGCGGUAGACAAAUUGGAUCAUUGAUUCUCUUUGAUUGGACUGAAGGGAGCUCCCUCU

((((.((((((((((.((((((.(((.(((..((.(.(((((((((((.(((.(((..((((((((.((..((.((((((................)))))).))..)).))))))))..))).)).).)))))))))))).))..))).))).)))))).)))))))))).)))) (-74.49)

....AGAGCTTCCTTGAGTCCATTC....................................................................................................................................................... 1

.........................ACAGGTCGTGATATGATTCAA.................................................................................................................................. 1

.............................................................................................................AATGAATGATGCGGTAGACAA.............................................. 2

.............................................................................................................AATGAATGATGCGGTAGACAAA............................................. 3

.............................................................................................................AATGAATGATGCGGTAGACAAAT............................................ 3

........................................................................................................................................................ATTGGACTGAAGGGAGCTCCC... 1

.........................................................................................................................................................TTGGACTGAAGGGAGCTCCCT.. 22

..........................................................................................................................................................TGGACTGAAGGGAGCTCCC... 2

..........................................................................................................................................................TGGACTGAAGGGAGCTCCCT.. 2

>ath-MIR319b

AGAGAGCUUUCUUCGGUCCACUCAUGGAGUAAUAUGUGAGAUUUAAUUGACUCUCGACUCAUUCAUCCAAAUACCAAAUGAAAGAAUUUGUUCUCAUAUGGUAAAUGAAUGAAUGAUGCGAGAGACAAAUUGAGUCUUCACUUCUCUAUGCUUGGACUGAAGGGAGCUCCCU

((.(((((..((((((((((..(((((((......(((((((((((((..((((((..((((((((.((..(((((.((((..(((....))))))).)))))..)).))))))))..))))))...))))))))).))))..)))))))..))))))))))..))))).)) (-80.80)

..AGAGCTTTCTTCGGTCCACT...................................................................................................................................................... 1

..AGAGCTTTCTTCGGTCCACTC..................................................................................................................................................... 8

...GAGCTTTCTTCGGTCCACT...................................................................................................................................................... 2

...GAGCTTTCTTCGGTCCACTC..................................................................................................................................................... 13

.............................................ATTGACTCTCGACTCATTCATCC........................................................................................................ 1

...............................................TGACTCTCGACTCATTCATCCA....................................................................................................... 1

...........................................................................................................AATGAATGATGCGAGAGAC.............................................. 5

...........................................................................................................AATGAATGATGCGAGAGACA............................................. 44

...........................................................................................................AATGAATGATGCGAGAGACAA............................................ 380

...........................................................................................................AATGAATGATGCGAGAGACAAA........................................... 35

...........................................................................................................AATGAATGATGCGAGAGACAAAT.......................................... 23

............................................................................................................ATGAATGATGCGAGAGACAA............................................ 2

............................................................................................................ATGAATGATGCGAGAGACAAA........................................... 2

......................................................................................................................................................CTTGGACTGAAGGGAGCTCCC. 4

.......................................................................................................................................................TTGGACTGAAGGGAGCTCCCT 22

........................................................................................................................................................TGGACTGAAGGGAGCTCCC. 2

........................................................................................................................................................TGGACTGAAGGGAGCTCCCT 2

>ath-MIR169j

GAGUAUAAUGAGGAAGAGAGGUCUAACAUGGCGAAAAGAGUCAUGUUUAGUAGCCAAGGAUGACUUGCCUGAUCUUUUUCACCUCCAUGAUUCAAUUUGUAAUUCAUGGGUUUUGGAUUAUUAUACAUUCAAAAGUAUAAUAAUUUGAAAUCAUGUUGAAUCUUGCGGGUUAGGUUUCAGGCAGUCUCCUUGGCUAUCUUGACAUGCUUUUUUCAUUCACG

..((..((((((.(((((..(((.((((((((.......))))))))..(((((((((((.((((.((((((.(((....((((.((.((((((((............(((((..(((((((((((........)))))))))))..)))))..)))))))).)).)))).)))..)))))))))))))))))))))...)))...))))))))))).)). (-84.74)

.....TAATGAGGAAGAGAGGTCTAAC.................................................................................................................................................................................................. 1

................................................AGTAGCCAAGGATGACTTGCCTG...................................................................................................................................................... 2

.................................................GTAGCCAAGGATGACTTGCCT....................................................................................................................................................... 12

.................................................GTAGCCAAGGATGACTTGCCTG...................................................................................................................................................... 48

.................................................GTAGCCAAGGATGACTTGCCTGA..................................................................................................................................................... 6

..................................................TAGCCAAGGATGACTTGCC........................................................................................................................................................ 833

..................................................TAGCCAAGGATGACTTGCCT....................................................................................................................................................... 350

..................................................TAGCCAAGGATGACTTGCCTG...................................................................................................................................................... 37758

..................................................TAGCCAAGGATGACTTGCCTGA..................................................................................................................................................... 4936

..................................................TAGCCAAGGATGACTTGCCTGAT.................................................................................................................................................... 21

...................................................AGCCAAGGATGACTTGCCT....................................................................................................................................................... 42

...................................................AGCCAAGGATGACTTGCCTG...................................................................................................................................................... 357

...................................................AGCCAAGGATGACTTGCCTGA..................................................................................................................................................... 64

...................................................AGCCAAGGATGACTTGCCTGAT.................................................................................................................................................... 3

....................................................GCCAAGGATGACTTGCCTGA..................................................................................................................................................... 4

....................................................GCCAAGGATGACTTGCCTGATC................................................................................................................................................... 3

.....................................................CCAAGGATGACTTGCCTGA..................................................................................................................................................... 16

......................................................CAAGGATGACTTGCCTGATC................................................................................................................................................... 3

..............................................................................................................................................................AATCTTGCGGGTTAGGTTTCA.......................................... 6

...............................................................................................................................................................ATCTTGCGGGTTAGGTTTCA.......................................... 3

..................................................................................................................................................................TTGCGGGTTAGGTTTCAGGCA...................................... 3

................................................................................................................................................................................TCAGGCAGTCTCCTTGGCTA......................... 10

.................................................................................................................................................................................CAGGCAGTCTCCTTGGCTAT........................ 10

.................................................................................................................................................................................CAGGCAGTCTCCTTGGCTATC....................... 4

..................................................................................................................................................................................AGGCAGTCTCCTTGGCTATC....................... 88

..................................................................................................................................................................................AGGCAGTCTCCTTGGCTATCT...................... 2

..................................................................................................................................................................................AGGCAGTCTCCTTGGCTATCTTG.................... 1

...................................................................................................................................................................................GGCAGTCTCCTTGGCTATC....................... 108

....................................................................................................................................................................................GCAGTCTCCTTGGCTATCT...................... 1

.............................................................................................................................................................................................TTGGCTATCTTGACATGCTTT........... 3

......................................................................................................................................................................................................TTGACATGCTTTTTTCATTC... 1

>ath-MIR169l

AUGAAGAAGAGAGGUCUAAUAUGGCGAAAAGAGUCAUGUUUAAUAGCCAAGGAUGACUUGCCUGAUCUUUUUCACCUCCAUGAUUCAAUUUUAAGUUCGUGGAUUUUGGAUUAUUAUGCGUUUAAAAGGUAUAAUAAUUUGAGAUCAUGUUGAAUCUUGCGGGUUAGGUUUCAGGCAGUCUCUUUGGCUAUCUUGACAUGCUUUCUUCAUC

(((((((.(((..(((.((((((((.......))))))))..(((((((((((.((((.((((((.(((....((((.((.((((((((............(((((..(((((((((((.(.....).)))))))))))..)))))..)))))))).)).)))).)))..)))))))))))))))))))))...)))...)))))))))). (-81.24)

.........................................AATAGCCAAGGATGACTTGCCTG................................................................................................................................................... 2

...........................................TAGCCAAGGATGACTTGCC..................................................................................................................................................... 833

...........................................TAGCCAAGGATGACTTGCCT.................................................................................................................................................... 350

...........................................TAGCCAAGGATGACTTGCCTG................................................................................................................................................... 37758

...........................................TAGCCAAGGATGACTTGCCTGA.................................................................................................................................................. 4936

...........................................TAGCCAAGGATGACTTGCCTGAT................................................................................................................................................. 21

............................................AGCCAAGGATGACTTGCCT.................................................................................................................................................... 42

............................................AGCCAAGGATGACTTGCCTG................................................................................................................................................... 357

............................................AGCCAAGGATGACTTGCCTGA.................................................................................................................................................. 64

............................................AGCCAAGGATGACTTGCCTGAT................................................................................................................................................. 3

.............................................GCCAAGGATGACTTGCCTGA.................................................................................................................................................. 4

.............................................GCCAAGGATGACTTGCCTGATC................................................................................................................................................ 3

..............................................CCAAGGATGACTTGCCTGA.................................................................................................................................................. 16

...............................................CAAGGATGACTTGCCTGATC................................................................................................................................................ 3

............................................................................................................................................GAGATCATGTTGAATCTTGCGGGTTAG............................................ 1

........................................................................................................................................................AATCTTGCGGGTTAGGTTTCA...................................... 6

.........................................................................................................................................................ATCTTGCGGGTTAGGTTTCA...................................... 3

............................................................................................................................................................TTGCGGGTTAGGTTTCAGGCA.................................. 3

.........................................................................................................................................................................TTCAGGCAGTCTCTTTGGCTA..................... 2

..........................................................................................................................................................................TCAGGCAGTCTCTTTGGCTA..................... 8

...........................................................................................................................................................................CAGGCAGTCTCTTTGGCTAT.................... 4

............................................................................................................................................................................AGGCAGTCTCTTTGGCTAT.................... 6

............................................................................................................................................................................AGGCAGTCTCTTTGGCTATC................... 204

............................................................................................................................................................................AGGCAGTCTCTTTGGCTATCT.................. 54

............................................................................................................................................................................AGGCAGTCTCTTTGGCTATCTT................. 2

.............................................................................................................................................................................GGCAGTCTCTTTGGCTATC................... 68

.............................................................................................................................................................................GGCAGTCTCTTTGGCTATCT.................. 14

.............................................................................................................................................................................GGCAGTCTCTTTGGCTATCTT................. 2

..............................................................................................................................................................................GCAGTCTCTTTGGCTATCT.................. 2

.......................................................................................................................................................................................TTGGCTATCTTGACATGCTTT....... 3

>ath-MIR169m

UAGAAGGAGAAGUCAAAGAUGAAUAGAAGAAUCAUAUUUGGUAGCCAAGGAUGACUUGCCUGUUUCUUUGAGUAAAAUGGGUUAGUGUCAUGUUUGACAAGUGACUAUAAGUUAUAUCAAGCAAUGACCAUUUUACUCAUCAAAAGACAUCAGGCAGUCUCCUUGGCUAUCCUUAUAUGUUCUUCUCUCUCAUCUCAGACGUUUACCUUCAU

..((((((((.(((..((((((..(((((((.(((((..((((((((((((.((((.(((((..((((((((((((((((.......((((((((((...(((((.....))))).)))))).))))))))))))))))....))))...))))))))))))))))))).))..))))))))))))...))))))..))).))).))))).. (-93.71)

..................ATGAATAGAAGAATCATATTTGG........................................................................................................................................................................... 2

...................TGAATAGAAGAATCATATTTG............................................................................................................................................................................ 2

...................TGAATAGAAGAATCATATTTGG........................................................................................................................................................................... 28

.......................................GGTAGCCAAGGATGACTTGC......................................................................................................................................................... 4

.......................................GGTAGCCAAGGATGACTTGCC........................................................................................................................................................ 4

.......................................GGTAGCCAAGGATGACTTGCCT....................................................................................................................................................... 16

.......................................GGTAGCCAAGGATGACTTGCCTG...................................................................................................................................................... 16

........................................GTAGCCAAGGATGACTTGCCT....................................................................................................................................................... 12

........................................GTAGCCAAGGATGACTTGCCTG...................................................................................................................................................... 48

.........................................TAGCCAAGGATGACTTGCC........................................................................................................................................................ 833

.........................................TAGCCAAGGATGACTTGCCT....................................................................................................................................................... 350

.........................................TAGCCAAGGATGACTTGCCTG...................................................................................................................................................... 37758

.........................................TAGCCAAGGATGACTTGCCTGT..................................................................................................................................................... 205

.........................................TAGCCAAGGATGACTTGCCTGTT.................................................................................................................................................... 3

..........................................AGCCAAGGATGACTTGCCT....................................................................................................................................................... 42

..........................................AGCCAAGGATGACTTGCCTG...................................................................................................................................................... 357

..........................................AGCCAAGGATGACTTGCCTGT..................................................................................................................................................... 2

.........................................................................................ATGTTTGACAAGTGACTATAAGTTAT................................................................................................. 1

....................................................................................................................................................ATCAGGCAGTCTCCTTGGCTA........................................... 3

.....................................................................................................................................................TCAGGCAGTCTCCTTGGCTA........................................... 10

......................................................................................................................................................CAGGCAGTCTCCTTGGCTAT.......................................... 10

......................................................................................................................................................CAGGCAGTCTCCTTGGCTATC......................................... 4

.......................................................................................................................................................AGGCAGTCTCCTTGGCTATC......................................... 88

.......................................................................................................................................................AGGCAGTCTCCTTGGCTATCC........................................ 12

........................................................................................................................................................GGCAGTCTCCTTGGCTATC......................................... 108

........................................................................................................................................................GGCAGTCTCCTTGGCTATCC........................................ 48

........................................................................................................................................................GGCAGTCTCCTTGGCTATCCT....................................... 3

.........................................................................................................................................................GCAGTCTCCTTGGCTATCC........................................ 9

.........................................................................................................................................................GCAGTCTCCTTGGCTATCCT....................................... 21

.........................................................................................................................................................GCAGTCTCCTTGGCTATCCTT...................................... 3

..........................................................................................................................................................CAGTCTCCTTGGCTATCCT....................................... 30

..........................................................................................................................................................CAGTCTCCTTGGCTATCCTT...................................... 15

.............................................................................................................................................................................TATATGTTCTTCTCTCTCAT................... 1

>ath-MIR169n

GAUGAAGAAGAGAGGUCUAACAUGGCGGAAAGCGUCAUGUUUAGUAGCCAAGGAUGACUUGCCUGAUCUUUUUCGCCUCCACGAUUCAAUUUCAAAUUCAUGCAUUUUGGAUUAUUAUACCUUUUAAAGUAUAAUAGGUCAAAUAUCAUGUUGAAUCUUGCGGGUUAGGUUUCAGGCAGUCUCUUUGGCUAUCUUGACAUGCUUUUUCCAUCCAU

((((((((((....(((.(((((((((.....)))))))))..(((((((((((.((((.((((((.(((....((((.((.(((((((.........((((...((((..(((((((((........)))))))))..))))...))))))))))).)).)))).)))..)))))))))))))))))))))...)))...)))))).))))... (-79.30)

.....AGAAGAGAGGTCTAACATGG.............................................................................................................................................................................................. 1

...........GAGGTCTAACATGGCGGAA......................................................................................................................................................................................... 1

............AGGTCTAACATGGCGGAAA........................................................................................................................................................................................ 1

......................TGGCGGAAAGCGTCATGTTT............................................................................................................................................................................. 1

......................TGGCGGAAAGCGTCATGTTTAG........................................................................................................................................................................... 7

......................TGGCGGAAAGCGTCATGTTTAGT.......................................................................................................................................................................... 2

..............................AGCGTCATGTTTAGTAGCCAAGGA................................................................................................................................................................. 1

..........................................AGTAGCCAAGGATGACTTGCCTG...................................................................................................................................................... 2

...........................................GTAGCCAAGGATGACTTGCCT....................................................................................................................................................... 12

...........................................GTAGCCAAGGATGACTTGCCTG...................................................................................................................................................... 48

...........................................GTAGCCAAGGATGACTTGCCTGA..................................................................................................................................................... 6

............................................TAGCCAAGGATGACTTGCC........................................................................................................................................................ 833

............................................TAGCCAAGGATGACTTGCCT....................................................................................................................................................... 350

............................................TAGCCAAGGATGACTTGCCTG...................................................................................................................................................... 37758

............................................TAGCCAAGGATGACTTGCCTGA..................................................................................................................................................... 4936

............................................TAGCCAAGGATGACTTGCCTGAT.................................................................................................................................................... 21

.............................................AGCCAAGGATGACTTGCCT....................................................................................................................................................... 42

.............................................AGCCAAGGATGACTTGCCTG...................................................................................................................................................... 357

.............................................AGCCAAGGATGACTTGCCTGA..................................................................................................................................................... 64

.............................................AGCCAAGGATGACTTGCCTGAT.................................................................................................................................................... 3

..............................................GCCAAGGATGACTTGCCTGA..................................................................................................................................................... 4

..............................................GCCAAGGATGACTTGCCTGATC................................................................................................................................................... 3

...............................................CCAAGGATGACTTGCCTGA..................................................................................................................................................... 16

................................................CAAGGATGACTTGCCTGATC................................................................................................................................................... 3

.........................................................................................................................................................AATCTTGCGGGTTAGGTTTCA......................................... 6

..........................................................................................................................................................ATCTTGCGGGTTAGGTTTCA......................................... 3

.............................................................................................................................................................TTGCGGGTTAGGTTTCAGGCA..................................... 3

..........................................................................................................................................................................TTCAGGCAGTCTCTTTGGCTA........................ 2

...........................................................................................................................................................................TCAGGCAGTCTCTTTGGCTA........................ 8

............................................................................................................................................................................CAGGCAGTCTCTTTGGCTAT....................... 4

.............................................................................................................................................................................AGGCAGTCTCTTTGGCTAT....................... 6

.............................................................................................................................................................................AGGCAGTCTCTTTGGCTATC...................... 204

.............................................................................................................................................................................AGGCAGTCTCTTTGGCTATCT..................... 54

.............................................................................................................................................................................AGGCAGTCTCTTTGGCTATCTT.................... 2

..............................................................................................................................................................................GGCAGTCTCTTTGGCTATC...................... 68

..............................................................................................................................................................................GGCAGTCTCTTTGGCTATCT..................... 14

..............................................................................................................................................................................GGCAGTCTCTTTGGCTATCTT.................... 2

...............................................................................................................................................................................GCAGTCTCTTTGGCTATCT..................... 2

........................................................................................................................................................................................TTGGCTATCTTGACATGCTTT.......... 3

............................................................................................................................................................................................CTATCTTGACATGCTTTTTC....... 1

>ath-MIR447a

CAUUCUUAAUAUAUAAUACUACUUUUUCAUCCAUUAAACCCCUUACAAUGUCGAGUAAACGAAGCAUCUGUCCCCUGGUAUUGUCUUCGAGCUUGGUGUUUUUUUCUAGCCAACUCCAAGUUCUCGAGUUGAUCAUUGUUUGUAUUCUUGAGACAUUAUUUGGGGACGAGAUGUUUUGUUGACUCGAUAUAAGAAGGGGCUUUAUGGAAGAAAUUGUAGUAUUAUAUAUCGAGAGUG

((((((..((((((((((((((..((((.(((((.((.((((((...(((((((((.((((((((((((((((((..(((.((((((.((((((((.(((...........))).))))))))..((((....((.....)).))))..)))))).)))..)))))).)))))))))))).)))))))))...)))))).)).))))).))))..))))))))))))))..)))))) (-111.20)

.................ACTACTTTTTCATCCATTAAA....................................................................................................................................................................................................... 1

.....................................ACCCCTTACAATGTCGAGTA.................................................................................................................................................................................... 1

.....................................ACCCCTTACAATGTCGAGTAA................................................................................................................................................................................... 104

.....................................ACCCCTTACAATGTCGAGTAAA.................................................................................................................................................................................. 1

...................................................CGAGTAAACGAAGCATCTGTCCC................................................................................................................................................................... 4

.....................................................AGTAAACGAAGCATCTGTCCCC.................................................................................................................................................................. 2

..........................................................ACGAAGCATCTGTCCCCTGG............................................................................................................................................................... 2

..........................................................ACGAAGCATCTGTCCCCTGGT.............................................................................................................................................................. 2

....................................................................................................................................................TGAGACATTATTTGGGGACGAG................................................................... 1

..............................................................................................................................................................TTTGGGGACGAGATGTTTTGTT......................................................... 10

...............................................................................................................................................................TTGGGGACGAGATGTTTTGT.......................................................... 6

...............................................................................................................................................................TTGGGGACGAGATGTTTTGTT......................................................... 6

...............................................................................................................................................................TTGGGGACGAGATGTTTTGTTG........................................................ 84

...............................................................................................................................................................TTGGGGACGAGATGTTTTGTTGA....................................................... 6

................................................................................................................................................................TGGGGACGAGATGTTTTGT.......................................................... 4

................................................................................................................................................................TGGGGACGAGATGTTTTGTT......................................................... 4

................................................................................................................................................................TGGGGACGAGATGTTTTGTTG........................................................ 66

................................................................................................................................................................TGGGGACGAGATGTTTTGTTGA....................................................... 8

................................................................................................................................................................TGGGGACGAGATGTTTTGTTGAC...................................................... 4

.............................................................................................................................................................................TTTTGTTGACTCGATATAAGA........................................... 3

....................................................................................................................................................................................GACTCGATATAAGAAGGGGCT.................................... 16

....................................................................................................................................................................................GACTCGATATAAGAAGGGGCTT................................... 2

.....................................................................................................................................................................................ACTCGATATAAGAAGGGGC..................................... 2

.....................................................................................................................................................................................ACTCGATATAAGAAGGGGCT.................................... 6

.....................................................................................................................................................................................ACTCGATATAAGAAGGGGCTT................................... 66

.....................................................................................................................................................................................ACTCGATATAAGAAGGGGCTTTA................................. 2

.........................................................................................................................................................................................................TTATGGAAGAAATTGTAGTA................ 2

.........................................................................................................................................................................................................TTATGGAAGAAATTGTAGTAT............... 22

..........................................................................................................................................................................................................TATGGAAGAAATTGTAGTAT............... 2

..........................................................................................................................................................................................................TATGGAAGAAATTGTAGTATT.............. 66

..........................................................................................................................................................................................................TATGGAAGAAATTGTAGTATTA............. 2

...........................................................................................................................................................................................................ATGGAAGAAATTGTAGTATT.............. 2

...............................................................................................................................................................................................................AAGAAATTGTAGTATTATATA......... 6

...................................................................................................................................................................................................................AATTGTAGTATTATATATCGA..... 1

>ath-MIR447b

CAUUCUUAAUAUACAAUACUACUUUUUCAUCCAUUAAUCCCCUUACAAUGUCGAGUAAACGAAGCAUCUGUCCCCUGGUAUUGUCUUCGAGCUUGGUGUGUUUUUCUAGCCAGCCCCAAGUUCUCGAGUUGAUCAUUGUUUGUAUUCUGACACAUUAUUUGGGGACGAGAUGUUUUGUUGACUCGAUAUAAGAAGGGGCUUUAUGGAAGAAAUUGUAGUAUUAUAUAUUGAGAAUG

(((((((((((((.((((((((..((((.(((((.((.((((((...(((((((((.((((((((((((((((((..(((.((((...((((((((.(((((.....)))..)).))))))))..((((....((.....)).)))).))))...)))..)))))).)))))))))))).)))))))))...)))))).)).))))).))))..)))))))).))))))))))))) (-105.80)

...................................................CGAGTAAACGAAGCATCTGTCCC.................................................................................................................................................................. 4

.....................................................AGTAAACGAAGCATCTGTCCCC................................................................................................................................................................. 2

..........................................................ACGAAGCATCTGTCCCCTGG.............................................................................................................................................................. 2

..........................................................ACGAAGCATCTGTCCCCTGGT............................................................................................................................................................. 2

.............................................................................................................................................................TTTGGGGACGAGATGTTTTGTT......................................................... 10

..............................................................................................................................................................TTGGGGACGAGATGTTTTGT.......................................................... 6

..............................................................................................................................................................TTGGGGACGAGATGTTTTGTT......................................................... 6

..............................................................................................................................................................TTGGGGACGAGATGTTTTGTTG........................................................ 84

..............................................................................................................................................................TTGGGGACGAGATGTTTTGTTGA....................................................... 6

...............................................................................................................................................................TGGGGACGAGATGTTTTGT.......................................................... 4

...............................................................................................................................................................TGGGGACGAGATGTTTTGTT......................................................... 4

...............................................................................................................................................................TGGGGACGAGATGTTTTGTTG........................................................ 66

...............................................................................................................................................................TGGGGACGAGATGTTTTGTTGA....................................................... 8

...............................................................................................................................................................TGGGGACGAGATGTTTTGTTGAC...................................................... 4

............................................................................................................................................................................TTTTGTTGACTCGATATAAGA........................................... 3

...................................................................................................................................................................................GACTCGATATAAGAAGGGGCT.................................... 16

...................................................................................................................................................................................GACTCGATATAAGAAGGGGCTT................................... 2

....................................................................................................................................................................................ACTCGATATAAGAAGGGGC..................................... 2

....................................................................................................................................................................................ACTCGATATAAGAAGGGGCT.................................... 6

....................................................................................................................................................................................ACTCGATATAAGAAGGGGCTT................................... 66

....................................................................................................................................................................................ACTCGATATAAGAAGGGGCTTTA................................. 2

........................................................................................................................................................................................................TTATGGAAGAAATTGTAGTA................ 2

........................................................................................................................................................................................................TTATGGAAGAAATTGTAGTAT............... 22

.........................................................................................................................................................................................................TATGGAAGAAATTGTAGTAT............... 2

.........................................................................................................................................................................................................TATGGAAGAAATTGTAGTATT.............. 66

.........................................................................................................................................................................................................TATGGAAGAAATTGTAGTATTA............. 2

..........................................................................................................................................................................................................ATGGAAGAAATTGTAGTATT.............. 2

..............................................................................................................................................................................................................AAGAAATTGTAGTATTATATA......... 6

>ath-MIR775

UUUAAACGUUGCACUACGUGACAUUGAAACUGUCUUUCAACAUUCCAAUAUUUCAACUUUCGAAUACCCAAUAUUUGGUUUGUUCAAAGACAUUUUCGAUGUCUAGCAGUGCCAAUGUUUAAA

.((((((((((((((....((((((((((.(((((((.((((..((((((((.................)))).))))..)))).))))))).))))))))))....)))).)))))))))). (-43.23)

....AACGTTGCACTACGTGACATTGAA............................................................................................... 1

........TTGCACTACGTGACATTGAA............................................................................................... 1

..........GCACTACGTGACATTGAAAC............................................................................................. 5

..........GCACTACGTGACATTGAAACTG........................................................................................... 2

...........CACTACGTGACATTGAAACTGTC......................................................................................... 1

...............ACGTGACATTGAAACTGTCTT....................................................................................... 1

..................TGACATTGAAACTGTCTTTCA.................................................................................... 1

......................................................................ATATTTGGTTTGTTCAAAGACATT............................. 5

.........................................................................TTTGGTTTGTTCAAAGACATT............................. 2

.........................................................................TTTGGTTTGTTCAAAGACATTTTC.......................... 3

............................................................................................TTTTCGATGTCTAGCAGTG............ 6

............................................................................................TTTTCGATGTCTAGCAGTGC........... 5

............................................................................................TTTTCGATGTCTAGCAGTGCC.......... 45

............................................................................................TTTTCGATGTCTAGCAGTGCCA......... 122

............................................................................................TTTTCGATGTCTAGCAGTGCCAA........ 504

............................................................................................TTTTCGATGTCTAGCAGTGCCAAT....... 6

.............................................................................................TTTCGATGTCTAGCAGTGCC.......... 6

.............................................................................................TTTCGATGTCTAGCAGTGCCA......... 34

.............................................................................................TTTCGATGTCTAGCAGTGCCAA........ 74

..............................................................................................TTCGATGTCTAGCAGTGCC.......... 17

..............................................................................................TTCGATGTCTAGCAGTGCCA......... 1673

..............................................................................................TTCGATGTCTAGCAGTGCCAA........ 474

..............................................................................................TTCGATGTCTAGCAGTGCCAAT....... 82

...............................................................................................TCGATGTCTAGCAGTGCCA......... 63

...............................................................................................TCGATGTCTAGCAGTGCCAA........ 11

...............................................................................................TCGATGTCTAGCAGTGCCAAT....... 6

................................................................................................CGATGTCTAGCAGTGCCAA........ 6

................................................................................................CGATGTCTAGCAGTGCCAAT....... 1

.................................................................................................GATGTCTAGCAGTGCCAAT....... 1

.....................................................................................................TCTAGCAGTGCCAATGTTTAAA 1

>ath-MIR822

CGACCUUAAGUAUAAGUAGAUAUAUGGGGAUGUAACGCAUGUUGUUUUCUGCGGGAAGCAUUUGCACAUGUUUCGUGGAGAAUGAAAUCACAUUCCAUACAUGAAUAAUAAUUACCUUUUAGAUAGAACAUCGUACUGCUUGAAAAAACAUGUUAAUGUCAUAAACUUUAUGAUGAAAACACCUAUAAAAAGCGUUUUUUCAAGCAUGUGCUAUCUAUGAAGGUAAUUACUAUUCAUGUAUAAAAUAUGAUUUUAUCCUCCAUAAAACAUGUGCAAAUGCUUUCUACAGGAAACAAUAUACGUUGCAUCCCCAUCUACUUACACUUAAGGUCGUUGU

(((((((((((.((((((((....(((((((((((((.(((((((((((((..((((((((((((((((((((..(((((.(((((((((.(((..(((((((((((.((((((((((((((((((.(((......(((((((((((((.((((..(((((((.....)))))))..)))).((......)).)))))))))))))))).))))))).))))))))))).)))))))))))..))).))))))))).)))))..))))))))))))))))))))..))))))))))))).))))))))))))))))))))).))))))))))).... (-175.60)

CGACCTTAAGTATAAGTAGAT............................................................................................................................................................................................................................................................................................................................ 5

..ACCTTAAGTATAAGTAGATAT.......................................................................................................................................................................................................................................................................................................................... 1

..ACCTTAAGTATAAGTAGATATATGG...................................................................................................................................................................................................................................................................................................................... 2

..ACCTTAAGTATAAGTAGATATATGGG..................................................................................................................................................................................................................................................................................................................... 1

.....TTAAGTATAAGTAGATATATGGGGA................................................................................................................................................................................................................................................................................................................... 1

............TAAGTAGATATATGGGGATGT................................................................................................................................................................................................................................................................................................................ 1

.............AAGTAGATATATGGGGATGTAACG............................................................................................................................................................................................................................................................................................................ 1

...................ATATATGGGGATGTAACGCAT......................................................................................................................................................................................................................................................................................................... 1

....................TATATGGGGATGTAACGCATG........................................................................................................................................................................................................................................................................................................ 3

....................TATATGGGGATGTAACGCATGTT...................................................................................................................................................................................................................................................................................................... 1

....................TATATGGGGATGTAACGCATGTTGT.................................................................................................................................................................................................................................................................................................... 1

.....................ATATGGGGATGTAACGCATGTTGT.................................................................................................................................................................................................................................................................................................... 5

......................TATGGGGATGTAACGCATGTTGTT................................................................................................................................................................................................................................................................................................... 1

........................TGGGGATGTAACGCATGTTGT.................................................................................................................................................................................................................................................................................................... 12

...........................GGATGTAACGCATGTTGTTT.................................................................................................................................................................................................................................................................................................. 1

...........................GGATGTAACGCATGTTGTTTT................................................................................................................................................................................................................................................................................................. 5

...........................GGATGTAACGCATGTTGTTTTC................................................................................................................................................................................................................................................................................................ 4

............................GATGTAACGCATGTTGTTT.................................................................................................................................................................................................................................................................................................. 6

............................GATGTAACGCATGTTGTTTT................................................................................................................................................................................................................................................................................................. 4

............................GATGTAACGCATGTTGTTTTC................................................................................................................................................................................................................................................................................................ 16

............................GATGTAACGCATGTTGTTTTCT............................................................................................................................................................................................................................................................................................... 89

............................GATGTAACGCATGTTGTTTTCTG.............................................................................................................................................................................................................................................................................................. 2

.............................ATGTAACGCATGTTGTTTTC................................................................................................................................................................................................................................................................................................ 1

.............................ATGTAACGCATGTTGTTTTCT............................................................................................................................................................................................................................................................................................... 4

..............................TGTAACGCATGTTGTTTTCT............................................................................................................................................................................................................................................................................................... 1

..............................TGTAACGCATGTTGTTTTCTG.............................................................................................................................................................................................................................................................................................. 3

..............................TGTAACGCATGTTGTTTTCTGCGG........................................................................................................................................................................................................................................................................................... 1

......................................ATGTTGTTTTCTGCGGGAAGCA..................................................................................................................................................................................................................................................................................... 1

............................................TTTTCTGCGGGAAGCATTTGC................................................................................................................................................................................................................................................................................ 2

.............................................TTTCTGCGGGAAGCATTTGCA............................................................................................................................................................................................................................................................................... 19

.............................................TTTCTGCGGGAAGCATTTGCAC.............................................................................................................................................................................................................................................................................. 1

.............................................TTTCTGCGGGAAGCATTTGCACAT............................................................................................................................................................................................................................................................................ 1

..............................................TTCTGCGGGAAGCATTTGCAC.............................................................................................................................................................................................................................................................................. 3

..............................................TTCTGCGGGAAGCATTTGCACA............................................................................................................................................................................................................................................................................. 1

...............................................TCTGCGGGAAGCATTTGCACA............................................................................................................................................................................................................................................................................. 38

...............................................TCTGCGGGAAGCATTTGCACAT............................................................................................................................................................................................................................................................................ 1

................................................CTGCGGGAAGCATTTGCACA............................................................................................................................................................................................................................................................................. 2

................................................CTGCGGGAAGCATTTGCACAT............................................................................................................................................................................................................................................................................ 13

................................................CTGCGGGAAGCATTTGCACATG........................................................................................................................................................................................................................................................................... 70

................................................CTGCGGGAAGCATTTGCACATGT.......................................................................................................................................................................................................................................................................... 7

.................................................TGCGGGAAGCATTTGCACA............................................................................................................................................................................................................................................................................. 109

.................................................TGCGGGAAGCATTTGCACAT............................................................................................................................................................................................................................................................................ 10

.................................................TGCGGGAAGCATTTGCACATG........................................................................................................................................................................................................................................................................... 3488

.................................................TGCGGGAAGCATTTGCACATGT.......................................................................................................................................................................................................................................................................... 127

.................................................TGCGGGAAGCATTTGCACATGTT......................................................................................................................................................................................................................................................................... 1

.................................................TGCGGGAAGCATTTGCACATGTTT........................................................................................................................................................................................................................................................................ 1

..................................................GCGGGAAGCATTTGCACAT............................................................................................................................................................................................................................................................................ 2

..................................................GCGGGAAGCATTTGCACATG........................................................................................................................................................................................................................................................................... 166

..................................................GCGGGAAGCATTTGCACATGT.......................................................................................................................................................................................................................................................................... 67

..................................................GCGGGAAGCATTTGCACATGTT......................................................................................................................................................................................................................................................................... 24

..................................................GCGGGAAGCATTTGCACATGTTT........................................................................................................................................................................................................................................................................ 2

...................................................CGGGAAGCATTTGCACATG........................................................................................................................................................................................................................................................................... 4

...................................................CGGGAAGCATTTGCACATGT.......................................................................................................................................................................................................................................................................... 2

...................................................CGGGAAGCATTTGCACATGTT......................................................................................................................................................................................................................................................................... 14

...................................................CGGGAAGCATTTGCACATGTTTCG...................................................................................................................................................................................................................................................................... 1

......................................................GAAGCATTTGCACATGTTT........................................................................................................................................................................................................................................................................ 1

......................................................GAAGCATTTGCACATGTTTCGTGG................................................................................................................................................................................................................................................................... 1

........................................................AGCATTTGCACATGTTTCGTGGAG................................................................................................................................................................................................................................................................. 2

.........................................................GCATTTGCACATGTTTCGTGGAGA................................................................................................................................................................................................................................................................ 1

..........................................................CATTTGCACATGTTTCGTGGA.................................................................................................................................................................................................................................................................. 1

...........................................................ATTTGCACATGTTTCGTGGA.................................................................................................................................................................................................................................................................. 1

...........................................................ATTTGCACATGTTTCGTGGAG................................................................................................................................................................................................................................................................. 25

...........................................................ATTTGCACATGTTTCGTGGAGA................................................................................................................................................................................................................................................................ 1

...........................................................ATTTGCACATGTTTCGTGGAGAAT.............................................................................................................................................................................................................................................................. 2

............................................................TTTGCACATGTTTCGTGGA.................................................................................................................................................................................................................................................................. 1

............................................................TTTGCACATGTTTCGTGGAG................................................................................................................................................................................................................................................................. 3

............................................................TTTGCACATGTTTCGTGGAGA................................................................................................................................................................................................................................................................ 46

............................................................TTTGCACATGTTTCGTGGAGAA............................................................................................................................................................................................................................................................... 14

............................................................TTTGCACATGTTTCGTGGAGAAT.............................................................................................................................................................................................................................................................. 1

............................................................TTTGCACATGTTTCGTGGAGAATG............................................................................................................................................................................................................................................................. 1

.............................................................TTGCACATGTTTCGTGGAGA................................................................................................................................................................................................................................................................ 2

.............................................................TTGCACATGTTTCGTGGAGAA............................................................................................................................................................................................................................................................... 4

.............................................................TTGCACATGTTTCGTGGAGAAT.............................................................................................................................................................................................................................................................. 1

..............................................................TGCACATGTTTCGTGGAGA................................................................................................................................................................................................................................................................ 2

..............................................................TGCACATGTTTCGTGGAGAAT.............................................................................................................................................................................................................................................................. 3

....................................................................TGTTTCGTGGAGAATGAAATCA....................................................................................................................................................................................................................................................... 1

......................................................................TTTCGTGGAGAATGAAATC........................................................................................................................................................................................................................................................ 1

......................................................................TTTCGTGGAGAATGAAATCAC...................................................................................................................................................................................................................................................... 1

......................................................................TTTCGTGGAGAATGAAATCACA..................................................................................................................................................................................................................................................... 5

.......................................................................TTCGTGGAGAATGAAATCACA..................................................................................................................................................................................................................................................... 2

......................................................................................ATCACATTCCATACATGAATA...................................................................................................................................................................................................................................... 2

..............................................................................................CCATACATGAATAATAATTAC.............................................................................................................................................................................................................................. 1

..............................................................................................CCATACATGAATAATAATTACC............................................................................................................................................................................................................................. 4

...............................................................................................CATACATGAATAATAATTACC............................................................................................................................................................................................................................. 4

.................................................................................................TACATGAATAATAATTACCTTTT......................................................................................................................................................................................................................... 1

..................................................................................................ACATGAATAATAATTACCTTTTA........................................................................................................................................................................................................................ 1

...........................................................................................................................TAGAACATCGTACTGCTTGAA................................................................................................................................................................................................. 1

.....................................................................................................................................................................................................TTTCAAGCATGTGCTATCTAT....................................................................................................................... 1

.........................................................................................................................................................................................................................TGAAGGTAATTACTATTCATGTAT................................................................................................ 1

..........................................................................................................................................................................................................................GAAGGTAATTACTATTCATGT.................................................................................................. 1

...........................................................................................................................................................................................................................AAGGTAATTACTATTCATGT.................................................................................................. 1

...............................................................................................................................................................................................................................TAATTACTATTCATGTATAA.............................................................................................. 1

..................................................................................................................................................................................................................................................AAATATGATTTTATCCTCCAT.......................................................................... 1

..................................................................................................................................................................................................................................................AAATATGATTTTATCCTCCATA......................................................................... 1

....................................................................................................................................................................................................................................................ATATGATTTTATCCTCCATAA........................................................................ 1

....................................................................................................................................................................................................................................................ATATGATTTTATCCTCCATAAA....................................................................... 2

.....................................................................................................................................................................................................................................................TATGATTTTATCCTCCATAAAA...................................................................... 4

.......................................................................................................................................................................................................................................................TGATTTTATCCTCCATAAAACA.................................................................... 1

.........................................................................................................................................................................................................................................................ATTTTATCCTCCATAAAAC..................................................................... 1

.........................................................................................................................................................................................................................................................ATTTTATCCTCCATAAAACATG.................................................................. 1

..............................................................................................................................................................................................................................................................ATCCTCCATAAAACATGTGCA.............................................................. 8

...............................................................................................................................................................................................................................................................TCCTCCATAAAACATGTGCAAA............................................................ 1

................................................................................................................................................................................................................................................................CCTCCATAAAACATGTGCAAA............................................................ 1

..................................................................................................................................................................................................................................................................TCCATAAAACATGTGCAAATG.......................................................... 1

........................................................................................................................................................................................................................................................................AAACATGTGCAAATGCTTTCT.................................................... 1

.........................................................................................................................................................................................................................................................................AACATGTGCAAATGCTTTCTA................................................... 1

..........................................................................................................................................................................................................................................................................ACATGTGCAAATGCTTTCTAC.................................................. 7

..........................................................................................................................................................................................................................................................................ACATGTGCAAATGCTTTCTACA................................................. 16

...........................................................................................................................................................................................................................................................................CATGTGCAAATGCTTTCTAC.................................................. 2

...........................................................................................................................................................................................................................................................................CATGTGCAAATGCTTTCTACA................................................. 11

...........................................................................................................................................................................................................................................................................CATGTGCAAATGCTTTCTACAG................................................ 22

...........................................................................................................................................................................................................................................................................CATGTGCAAATGCTTTCTACAGG............................................... 2

............................................................................................................................................................................................................................................................................ATGTGCAAATGCTTTCTACA................................................. 6

............................................................................................................................................................................................................................................................................ATGTGCAAATGCTTTCTACAG................................................ 474

............................................................................................................................................................................................................................................................................ATGTGCAAATGCTTTCTACAGG............................................... 43

............................................................................................................................................................................................................................................................................ATGTGCAAATGCTTTCTACAGGA.............................................. 2

............................................................................................................................................................................................................................................................................ATGTGCAAATGCTTTCTACAGGAA............................................. 2

.............................................................................................................................................................................................................................................................................TGTGCAAATGCTTTCTACA................................................. 1

.............................................................................................................................................................................................................................................................................TGTGCAAATGCTTTCTACAG................................................ 1

.............................................................................................................................................................................................................................................................................TGTGCAAATGCTTTCTACAGG............................................... 52

.............................................................................................................................................................................................................................................................................TGTGCAAATGCTTTCTACAGGA.............................................. 34

..............................................................................................................................................................................................................................................................................GTGCAAATGCTTTCTACAG................................................ 6

..............................................................................................................................................................................................................................................................................GTGCAAATGCTTTCTACAGGA.............................................. 4

...............................................................................................................................................................................................................................................................................TGCAAATGCTTTCTACAGGA.............................................. 1

...............................................................................................................................................................................................................................................................................TGCAAATGCTTTCTACAGGAA............................................. 6

................................................................................................................................................................................................................................................................................GCAAATGCTTTCTACAGGA.............................................. 1

................................................................................................................................................................................................................................................................................GCAAATGCTTTCTACAGGAAA............................................ 6

...................................................................................................................................................................................................................................................................................AATGCTTTCTACAGGAAACAA......................................... 4

....................................................................................................................................................................................................................................................................................ATGCTTTCTACAGGAAACAATA....................................... 1

.............................................................................................................................................................................................................................................................................................ACAGGAAACAATATACGTTGCATC............................ 31

...............................................................................................................................................................................................................................................................................................AGGAAACAATATACGTTGCAT............................. 1

...............................................................................................................................................................................................................................................................................................AGGAAACAATATACGTTGCATC............................ 2

...............................................................................................................................................................................................................................................................................................AGGAAACAATATACGTTGCATCC........................... 1

................................................................................................................................................................................................................................................................................................GGAAACAATATACGTTGCA.............................. 2

................................................................................................................................................................................................................................................................................................GGAAACAATATACGTTGCATC............................ 13

.................................................................................................................................................................................................................................................................................................GAAACAATATACGTTGCATC............................ 2

.................................................................................................................................................................................................................................................................................................GAAACAATATACGTTGCATCCC.......................... 62

.................................................................................................................................................................................................................................................................................................GAAACAATATACGTTGCATCCCC......................... 3

..................................................................................................................................................................................................................................................................................................AAACAATATACGTTGCATC............................ 12

..................................................................................................................................................................................................................................................................................................AAACAATATACGTTGCATCC........................... 14

..................................................................................................................................................................................................................................................................................................AAACAATATACGTTGCATCCC.......................... 1094

..................................................................................................................................................................................................................................................................................................AAACAATATACGTTGCATCCCC......................... 82

..................................................................................................................................................................................................................................................................................................AAACAATATACGTTGCATCCCCA........................ 1

...................................................................................................................................................................................................................................................................................................AACAATATACGTTGCATCC........................... 1

...................................................................................................................................................................................................................................................................................................AACAATATACGTTGCATCCC.......................... 8

...................................................................................................................................................................................................................................................................................................AACAATATACGTTGCATCCCC......................... 339

...................................................................................................................................................................................................................................................................................................AACAATATACGTTGCATCCCCA........................ 15

....................................................................................................................................................................................................................................................................................................ACAATATACGTTGCATCCC.......................... 1

....................................................................................................................................................................................................................................................................................................ACAATATACGTTGCATCCCCA........................ 38

.............................................................................................................................................................................................................................................................................................................GTTGCATCCCCATCTACTTACACT............ 1

.....................................................................................................................................................................................................................................................................................................................CCCATCTACTTACACTTAAGGTC..... 3

.....................................................................................................................................................................................................................................................................................................................CCCATCTACTTACACTTAAGGTCG.... 2

.....................................................................................................................................................................................................................................................................................................................CCCATCTACTTACACTTAAGGTCGT... 2

.......................................................................................................................................................................................................................................................................................................................CATCTACTTACACTTAAGG....... 6

.......................................................................................................................................................................................................................................................................................................................CATCTACTTACACTTAAGGTC..... 7

.......................................................................................................................................................................................................................................................................................................................CATCTACTTACACTTAAGGTCG.... 58

.......................................................................................................................................................................................................................................................................................................................CATCTACTTACACTTAAGGTCGT... 9

........................................................................................................................................................................................................................................................................................................................ATCTACTTACACTTAAGGT...... 7

........................................................................................................................................................................................................................................................................................................................ATCTACTTACACTTAAGGTC..... 3

........................................................................................................................................................................................................................................................................................................................ATCTACTTACACTTAAGGTCG.... 178

........................................................................................................................................................................................................................................................................................................................ATCTACTTACACTTAAGGTCGT... 16

........................................................................................................................................................................................................................................................................................................................ATCTACTTACACTTAAGGTCGTT.. 1

.........................................................................................................................................................................................................................................................................................................................TCTACTTACACTTAAGGTCG.... 7

.........................................................................................................................................................................................................................................................................................................................TCTACTTACACTTAAGGTCGT... 6

.........................................................................................................................................................................................................................................................................................................................TCTACTTACACTTAAGGTCGTTGT 2

..........................................................................................................................................................................................................................................................................................................................CTACTTACACTTAAGGTCG.... 6

..........................................................................................................................................................................................................................................................................................................................CTACTTACACTTAAGGTCGTT.. 1

...........................................................................................................................................................................................................................................................................................................................TACTTACACTTAAGGTCGT... 1

...........................................................................................................................................................................................................................................................................................................................TACTTACACTTAAGGTCGTT.. 2

...........................................................................................................................................................................................................................................................................................................................TACTTACACTTAAGGTCGTTG. 16

...........................................................................................................................................................................................................................................................................................................................TACTTACACTTAAGGTCGTTGT 5

............................................................................................................................................................................................................................................................................................................................ACTTACACTTAAGGTCGTTGT 22

.............................................................................................................................................................................................................................................................................................................................CTTACACTTAAGGTCGTTG. 3

.............................................................................................................................................................................................................................................................................................................................CTTACACTTAAGGTCGTTGT 2

>ath-MIR839

CUCACUCAUGUGAGCAGAAAGAGUAGCAUGAUAUUUUCUUCAAGGUCUUUACCAACCUUUCAUCGUUCCCUUCUUUGCAAUAACGCUGUUUUGCAAAACCGUGAUAGUGCUGAGCCGAUGAGCCUCUAAUGAAAUUAUAGACUCAUCGGCUCAGGACCAUUGCGGUUGUGCAAAACGGUGUUAUUGCUGAGAAGGGAACGCAUGAGAGGUUGGUAAAGACCUCAAUGGAAUCUUAUGGUACUCUUUUUGCUCACAUGAGUGAGU

(((((((((((((((((((((((((.(((((...((((....(((((((((((((((((((((((((((((((((.(((((((((((((((((((.(((((..((.((.(((((((((((((.((.((((...)))).)).))))))))))))).)).))..))))).))))))))))))))))))).)))))))))))).)))))))))))))))))))))....))))..))))).))))))))))))))))))))))))). (-182.80)

....CTCATGTGAGCAGAAAGAGTAG.............................................................................................................................................................................................................................................. 1

.....TCATGTGAGCAGAAAGAGTAG.............................................................................................................................................................................................................................................. 9

.................................................TACCAACCTTTCATCGTTCCC.................................................................................................................................................................................................. 5

.......................................................................................GTTTTGCAAAACCGTGATAGTGCT......................................................................................................................................................... 1

.........................................................................................TTTGCAAAACCGTGATAGTGCTGA....................................................................................................................................................... 1

...........................................................................................TGCAAAACCGTGATAGTGCTGA....................................................................................................................................................... 10

............................................................................................GCAAAACCGTGATAGTGCTGA....................................................................................................................................................... 2

.................................................................................................ACCGTGATAGTGCTGAGCCGATGA............................................................................................................................................... 1

....................................................................................................GTGATAGTGCTGAGCCGATGAGCC............................................................................................................................................ 1

........................................................................................................TAGTGCTGAGCCGATGAGCCT........................................................................................................................................... 1

.........................................................................................................AGTGCTGAGCCGATGAGCCTCTA........................................................................................................................................ 1

................................................................................................................AGCCGATGAGCCTCTAATGAAATT................................................................................................................................ 1

..................................................................................................................CCGATGAGCCTCTAATGAAATTAT.............................................................................................................................. 1

......................................................................................................................TGAGCCTCTAATGAAATTAT.............................................................................................................................. 1

.....................................................................................................................................ATTATAGACTCATCGGCTCAGGAC........................................................................................................... 1

.................................................................................................................................................TCGGCTCAGGACCATTGCGGT.................................................................................................. 2

........................................................................................................................................................AGGACCATTGCGGTTGTGCAA........................................................................................... 1

.................................................................................................................................................................GCGGTTGTGCAAAACGGTGTTATTG.............................................................................. 1

.............................................................................................................................................................................AACGGTGTTATTGCTGAGAAG...................................................................... 1

..............................................................................................................................................................................ACGGTGTTATTGCTGAGAAGG..................................................................... 2

.......................................................................................................................................................................................TTGCTGAGAAGGGAACGCAT............................................................. 1

........................................................................................................................................................................................TGCTGAGAAGGGAACGCATGAG.......................................................... 1

................................................................................................................................................................................................AGGGAACGCATGAGAGGTTGGT.................................................. 3

................................................................................................................................................................................................AGGGAACGCATGAGAGGTTGGTAA................................................ 1

..................................................................................................................................................................................................GGAACGCATGAGAGGTTGGTAA................................................ 2

...................................................................................................................................................................................................GAACGCATGAGAGGTTGGTAA................................................ 4

...................................................................................................................................................................................................GAACGCATGAGAGGTTGGTAAA............................................... 25

...................................................................................................................................................................................................GAACGCATGAGAGGTTGGTAAAGA............................................. 1

....................................................................................................................................................................................................AACGCATGAGAGGTTGGTAAAG.............................................. 1

>ath-MIR841

GCACCAACACUACUAUGUGCAGAAACUCUGUUCUUAAGUUGCUUGUGAAUACGAGCCACUUGAAACUGAAAGAAACAAAGAAACAAUAUCAGUAUAAAAUUUUAUCAAAUUAUACUACAAAAAAUGAAAUUUCUUUGUUUAUUUCAAUUUCUAGUGGGUCGUAUUCACAUGCAACUCAAGACUAGAGUAUCUACAACAUGGUCGACUUGGUG

.((((((..(.(((((((..(((.((((((.((((.((((((.(((((((((((.(((((.((((.(((((.(((((((((((.......(((((((............))))))).((.....))...))))))))))).))))).)))).))))).))))))))))).)))))).)))).)))))).)))...))))))).)..)))))) (-86.20)

............................TGTTCTTAAGTTGCTTGTGAA................................................................................................................................................................... 7

............................TGTTCTTAAGTTGCTTGTGAAT.................................................................................................................................................................. 1

.............................GTTCTTAAGTTGCTTGTGAATACG............................................................................................................................................................... 1

...............................TCTTAAGTTGCTTGTGAATACG............................................................................................................................................................... 1

......................................TTGCTTGTGAATACGAGCCACTT....................................................................................................................................................... 1

.......................................TGCTTGTGAATACGAGCCACT........................................................................................................................................................ 2

...............................................AATACGAGCCACTTGAAACTG................................................................................................................................................ 5

.................................................TACGAGCCACTTGAAACTGA............................................................................................................................................... 1

.................................................TACGAGCCACTTGAAACTGAA.............................................................................................................................................. 51

..................................................ACGAGCCACTTGAAACTGAA.............................................................................................................................................. 1

..................................................ACGAGCCACTTGAAACTGAAA............................................................................................................................................. 1

............................................................TGAAACTGAAAGAAACAAAGAAAC................................................................................................................................ 1

...........................................................................................................................................TATTTCAATTTCTAGTGGGTCGT.................................................. 1

............................................................................................................................................ATTTCAATTTCTAGTGGGTCGTA................................................. 2

............................................................................................................................................ATTTCAATTTCTAGTGGGTCGTAT................................................ 1

.............................................................................................................................................TTTCAATTTCTAGTGGGTCGTATT............................................... 7

..............................................................................................................................................TTCAATTTCTAGTGGGTCGTA................................................. 1

..............................................................................................................................................TTCAATTTCTAGTGGGTCGTAT................................................ 3

..............................................................................................................................................TTCAATTTCTAGTGGGTCGTATT............................................... 1

..............................................................................................................................................TTCAATTTCTAGTGGGTCGTATTC.............................................. 48

...............................................................................................................................................TCAATTTCTAGTGGGTCGTA................................................. 4

...............................................................................................................................................TCAATTTCTAGTGGGTCGTAT................................................ 81

...............................................................................................................................................TCAATTTCTAGTGGGTCGTATT............................................... 7

...............................................................................................................................................TCAATTTCTAGTGGGTCGTATTC.............................................. 3

................................................................................................................................................CAATTTCTAGTGGGTCGTAT................................................ 2

................................................................................................................................................CAATTTCTAGTGGGTCGTATT............................................... 42

................................................................................................................................................CAATTTCTAGTGGGTCGTATTC.............................................. 2

.................................................................................................................................................AATTTCTAGTGGGTCGTATTC.............................................. 10

.................................................................................................................................................AATTTCTAGTGGGTCGTATTCACA........................................... 18

..................................................................................................................................................ATTTCTAGTGGGTCGTATT............................................... 6

..................................................................................................................................................ATTTCTAGTGGGTCGTATTC.............................................. 17

..................................................................................................................................................ATTTCTAGTGGGTCGTATTCA............................................. 2230

..................................................................................................................................................ATTTCTAGTGGGTCGTATTCAC............................................ 2

..................................................................................................................................................ATTTCTAGTGGGTCGTATTCACA........................................... 1

..................................................................................................................................................ATTTCTAGTGGGTCGTATTCACAT.......................................... 5

...................................................................................................................................................TTTCTAGTGGGTCGTATTC.............................................. 2

...................................................................................................................................................TTTCTAGTGGGTCGTATTCA............................................. 53

...................................................................................................................................................TTTCTAGTGGGTCGTATTCAC............................................ 947

...................................................................................................................................................TTTCTAGTGGGTCGTATTCACA........................................... 27

...................................................................................................................................................TTTCTAGTGGGTCGTATTCACAT.......................................... 2

....................................................................................................................................................TTCTAGTGGGTCGTATTCA............................................. 29

....................................................................................................................................................TTCTAGTGGGTCGTATTCAC............................................ 72

....................................................................................................................................................TTCTAGTGGGTCGTATTCACA........................................... 80

....................................................................................................................................................TTCTAGTGGGTCGTATTCACATGC........................................ 12

.....................................................................................................................................................TCTAGTGGGTCGTATTCAC............................................ 34

.....................................................................................................................................................TCTAGTGGGTCGTATTCACA........................................... 31

.....................................................................................................................................................TCTAGTGGGTCGTATTCACAT.......................................... 192

.....................................................................................................................................................TCTAGTGGGTCGTATTCACATG......................................... 7

.....................................................................................................................................................TCTAGTGGGTCGTATTCACATGC........................................ 9

......................................................................................................................................................CTAGTGGGTCGTATTCACA........................................... 1

......................................................................................................................................................CTAGTGGGTCGTATTCACAT.......................................... 4

......................................................................................................................................................CTAGTGGGTCGTATTCACATG......................................... 1

.......................................................................................................................................................TAGTGGGTCGTATTCACAT.......................................... 1

.......................................................................................................................................................TAGTGGGTCGTATTCACATGC........................................ 3

.......................................................................................................................................................TAGTGGGTCGTATTCACATGCA....................................... 3

.......................................................................................................................................................TAGTGGGTCGTATTCACATGCAAC..................................... 6

........................................................................................................................................................AGTGGGTCGTATTCACATGC........................................ 1

..........................................................................................................................................................TGGGTCGTATTCACATGCAAC..................................... 43

............................................................................................................................................................GGTCGTATTCACATGCAACT.................................... 1

.................................................................................................................................................................TATTCACATGCAACTCAAGA............................... 1

.................................................................................................................................................................TATTCACATGCAACTCAAGAC.............................. 2

....................................................................................................................................................................TCACATGCAACTCAAGACTAG........................... 1

......................................................................................................................................................................ACATGCAACTCAAGACTAGAGTAT...................... 1

.......................................................................................................................................................................CATGCAACTCAAGACTAGAGTATC..................... 1

........................................................................................................................................................................ATGCAACTCAAGACTAGAGTATC..................... 1

>ath-MIR846

CAAACAUCUUGAAUCCGUUGAGGUUGAUCACGAUGAUGCCACAUCCGGUUUCAUUCAAGGACUUCUAUUCAGAACAAACUUCAUGAUUUCUGAACUAAUUGGAUAUGAUAAAUGGUAACAAGUAUUCACUUGCAUUCAAGGGACAAAAAAUCAUUGGGAUAUAUGAUUAUGACAAACACGAUUGGAAGCUGAAUGGUUGCGGGAGGCAAGCAGUGGGAUAAUGAUCUGCAAGUGGAAACUUCUUACUUUUAUCAUAUCCCAUCAGCUCGAAAGUCUUGAUGUUAGUUUUGAAUUGAAGUGCUUGAAUUACACCAGAUUUAUUGUGCUCGUGUAUCCCGGUAAAUUCGACUAAUGACCAA

....(((.(((((((((..((...(((.((((((((......(((.(((.(.(((((((.(((((.(((((((((.(((.(((.(((((.(((.((.((.((((((((((((.(((((.((((.(((((((((...............((((((........))))))...........(((((....((.(.((.((((.....)))).)).).))....))))).))))))))).)))).))))))))))))))))).)).)).))).))))).))).))).))))))))).))))).))))))).).))).))))))))))).)))....)).)))...))))))...)))..... (-110.60)

..................................................TCATTCAAGGACTTCTATTC................................................................................................................................................................................................................................................................................................. 1

..................................................TCATTCAAGGACTTCTATTCAGAA............................................................................................................................................................................................................................................................................................. 1

...................................................CATTCAAGGACTTCTATTC................................................................................................................................................................................................................................................................................................. 1

...................................................CATTCAAGGACTTCTATTCAG............................................................................................................................................................................................................................................................................................... 19

....................................................ATTCAAGGACTTCTATTCA................................................................................................................................................................................................................................................................................................ 1

....................................................ATTCAAGGACTTCTATTCAGA.............................................................................................................................................................................................................................................................................................. 1

.....................................................TTCAAGGACTTCTATTCAG............................................................................................................................................................................................................................................................................................... 2

.....................................................TTCAAGGACTTCTATTCAGA.............................................................................................................................................................................................................................................................................................. 4

.....................................................TTCAAGGACTTCTATTCAGAA............................................................................................................................................................................................................................................................................................. 17

......................................................TCAAGGACTTCTATTCAGAA............................................................................................................................................................................................................................................................................................. 1

......................................................TCAAGGACTTCTATTCAGAAC............................................................................................................................................................................................................................................................................................ 10

........................................................AAGGACTTCTATTCAGAACA........................................................................................................................................................................................................................................................................................... 1

.............................................................................ACTTCATGATTTCTGAACTAA..................................................................................................................................................................................................................................................................... 1

.............................................................................................ACTAATTGGATATGATAAATGGTAA................................................................................................................................................................................................................................................. 2

...............................................................................................TAATTGGATATGATAAATGGTAA................................................................................................................................................................................................................................................. 4

................................................................................................AATTGGATATGATAAATGGTA.................................................................................................................................................................................................................................................. 3

................................................................................................AATTGGATATGATAAATGGTAA................................................................................................................................................................................................................................................. 22

.................................................................................................ATTGGATATGATAAATGGTAA................................................................................................................................................................................................................................................. 1

..................................................................................................TTGGATATGATAAATGGTAAC................................................................................................................................................................................................................................................ 1

...................................................................................................TGGATATGATAAATGGTAA................................................................................................................................................................................................................................................. 1

....................................................................................................................................................................................................................................................ACTTTTATCATATCCCATCA............................................................................................... 2

....................................................................................................................................................................................................................................................ACTTTTATCATATCCCATCAG.............................................................................................. 15

....................................................................................................................................................................................................................................................ACTTTTATCATATCCCATCAGCTC........................................................................................... 1

.............................................................................................................................................................................................................................................................................................TTTTGAATTGAAGTGCTTGAA..................................................... 1

..............................................................................................................................................................................................................................................................................................TTTGAATTGAAGTGCTTGAA..................................................... 1

..............................................................................................................................................................................................................................................................................................TTTGAATTGAAGTGCTTGAAT.................................................... 6

...............................................................................................................................................................................................................................................................................................TTGAATTGAAGTGCTTGAAT.................................................... 5

...............................................................................................................................................................................................................................................................................................TTGAATTGAAGTGCTTGAATT................................................... 22

................................................................................................................................................................................................................................................................................................TGAATTGAAGTGCTTGAAT.................................................... 1

................................................................................................................................................................................................................................................................................................TGAATTGAAGTGCTTGAATT................................................... 1
