# Supplementary material for: Multiple distinct small RNAs originate from the same microRNA precursors
Source: Genome Biol. 2010 Aug 9;11(8):R81. doi: 10.1186/gb-2010-11-8-r81 (PMC2945783; doi:10.1186/gb-2010-11-8-r81)
Supplement: Additional file 3 — Supplemental File S2. This is a file for sequencing reads mapped and aligned to miRNA precursors that can produce miRNA-sibling small RNAs (msRNAs) in rice (osa). The sequencing data were obtained from GEO; see Materials and methods for details. [file gb-2010-11-8-r81-S3.DOCX]

Zhang, et al., Multiple distinct small RNAs originate from the same microRNA precursors

Supplemental File 2 - Sequencing reads mapped and aligned to miRNA precursors that can produce

miRNA-like RNAs in *Oryza sativa.*

>osa-MIR169a_MI0000679_Oryza_sativa_miR169a_stem-loop GSM361264

CGCCGGCGGCCUGACAUUGGGAUCGGAGGCCAUGGUGCAGCCAAGGAUGACUUGCCGAUCGAUCGAUCUAUCUAUGAAGCUAAGCUAGCUGGCCAUGGAUCCAUCCAUCAAUUGGCAAGUUGUUCUUGGCUACAUCUUGGCCCCUGCUCCUCAUGUAAGGCCGGCCUGUGGCG

((((((((((((.((((.((((.(((.(((((.((((.(((((((((..((((((((((.(((.(((..(((((((.(((((...)))))...)))))))..))).))).))))))))))..))))))))).)))).))))).))).)))).)))).))))).)))...)))) (-99.60)

................TTGGGATCGGAGGCCATG........................................................................................................................................... 1

................TTGGGATCGGAGGCCATGGTG........................................................................................................................................ 7

................TTGGGATCGGAGGCCATGGTGC....................................................................................................................................... 3

.....................ATCGGAGGCCATGGTGCAGCC................................................................................................................................... 34

....................................GCAGCCAAGGATGACTTGCCG.................................................................................................................... 3

.....................................CAGCCAAGGATGACTTGC...................................................................................................................... 1

.....................................CAGCCAAGGATGACTTGCC..................................................................................................................... 1

.....................................CAGCCAAGGATGACTTGCCGA................................................................................................................... 200

.....................................CAGCCAAGGATGACTTGCCGATCG................................................................................................................ 6

......................................AGCCAAGGATGACTTGCCGA................................................................................................................... 5

..........................................AAGGATGACTTGCCGATCG................................................................................................................ 1

..........................................AAGGATGACTTGCCGATCGA............................................................................................................... 1

..........................................AAGGATGACTTGCCGATCGAT.............................................................................................................. 49

..........................................AAGGATGACTTGCCGATCGATCG............................................................................................................ 77

..........................................AAGGATGACTTGCCGATCGATCGA........................................................................................................... 120

..........................................AAGGATGACTTGCCGATCGATCGAT.......................................................................................................... 7

...........................................AGGATGACTTGCCGATCGATCGA........................................................................................................... 1

.............................................................................................................AATTGGCAAGTTGTTCTTGGCTAC........................................ 1

................................................................................................................TGGCAAGTTGTTCTTGGCTAC........................................ 1

.......................................................................................................................TTGTTCTTGGCTACATCTTGG................................. 1

.............................................................................................................................TTGGCTACATCTTGGCCCCTGCTC........................ 1

.................................................................................................................................CTACATCTTGGCCCCTGCT......................... 1

.................................................................................................................................CTACATCTTGGCCCCTGCTCC....................... 5

>osa-MIR159a_MI0001092_Oryza_sativa_miR159a_stem-loop GSM361264

GUUGUGGACGUUGAGCUCCUUUCGGUCCAAAAAGGGGUGUUGCUGUGGGUCGAUUGAGCUGCUGGGUCAUGGAUCCCGUUAGCCUACUCCAUGUUCAUCAUUCAGCUCGAGAUCUGAAAGAAACUACUCCAAUUUAUACUAAUAGUAUGUGUGUAGAUAGGAAAAUGAUGGAGUACUCGUUGUUGGGAUAGGCUUAUGGCUUGCAUGCCCCAGGAGCUGCAUCAACCCUACAUGGACCCUCUUUGGAUUGAAGGGAGCUCUGCAUCUUUUGU

.....(((.((.((((((((((((((((((..(((((.(((..(((((((.(((..((((.(((((.((((.(..((((.((((((..(((..((((((((((((........))))..........(((.((((((((...........)))))))).)))..))))))))..((.....)))))..)))))).))))..).)))).))))).))))..))).)))).)))..)))))))))))))))))))))))))).)).)))..... (-114.70)

..........TTGAGCTCCTTTCGGTCCAAA................................................................................................................................................................................................................................................. 16

..........TTGAGCTCCTTTCGGTCCAAAA................................................................................................................................................................................................................................................ 2

...........TGAGCTCCTTTCGGTCCAA.................................................................................................................................................................................................................................................. 1

...........TGAGCTCCTTTCGGTCCAAA................................................................................................................................................................................................................................................. 5

...........TGAGCTCCTTTCGGTCCAAAA................................................................................................................................................................................................................................................ 70

...........TGAGCTCCTTTCGGTCCAAAAA............................................................................................................................................................................................................................................... 11

............GAGCTCCTTTCGGTCCAAA................................................................................................................................................................................................................................................. 1

............GAGCTCCTTTCGGTCCAAAA................................................................................................................................................................................................................................................ 2

............GAGCTCCTTTCGGTCCAAAAA............................................................................................................................................................................................................................................... 11

.............AGCTCCTTTCGGTCCAAAAAG.............................................................................................................................................................................................................................................. 1

..............GCTCCTTTCGGTCCAAAAA............................................................................................................................................................................................................................................... 1

......................................................TGAGCTGCTGGGTCATGGATCC.................................................................................................................................................................................................... 2

.......................................................GAGCTGCTGGGTCATGGATCC.................................................................................................................................................................................................... 1

........................................................AGCTGCTGGGTCATGGAT...................................................................................................................................................................................................... 27

........................................................AGCTGCTGGGTCATGGATC..................................................................................................................................................................................................... 67

........................................................AGCTGCTGGGTCATGGATCC.................................................................................................................................................................................................... 10

........................................................AGCTGCTGGGTCATGGATCCC................................................................................................................................................................................................... 7

.........................................................GCTGCTGGGTCATGGATC..................................................................................................................................................................................................... 2

.........................................................GCTGCTGGGTCATGGATCC.................................................................................................................................................................................................... 1

.....................................................................................................................................................................................................GGCTTGCATGCCCCAGGAGCTGC.................................................... 1

.......................................................................................................................................................................................................CTTGCATGCCCCAGGAGCTG..................................................... 1

.......................................................................................................................................................................................................CTTGCATGCCCCAGGAGCTGC.................................................... 57

.......................................................................................................................................................................................................CTTGCATGCCCCAGGAGCTGCA................................................... 2

........................................................................................................................................................................................................TTGCATGCCCCAGGAGCT...................................................... 2

........................................................................................................................................................................................................TTGCATGCCCCAGGAGCTG..................................................... 3

........................................................................................................................................................................................................TTGCATGCCCCAGGAGCTGC.................................................... 2769

........................................................................................................................................................................................................TTGCATGCCCCAGGAGCTGCA................................................... 925

........................................................................................................................................................................................................TTGCATGCCCCAGGAGCTGCAT.................................................. 39

.........................................................................................................................................................................................................TGCATGCCCCAGGAGCTGC.................................................... 127

.........................................................................................................................................................................................................TGCATGCCCCAGGAGCTGCA................................................... 37

.........................................................................................................................................................................................................TGCATGCCCCAGGAGCTGCAT.................................................. 4

..........................................................................................................................................................................................................GCATGCCCCAGGAGCTGC.................................................... 15

..........................................................................................................................................................................................................GCATGCCCCAGGAGCTGCA................................................... 5

...................................................................................................................................................................................................................AGGAGCTGCATCAACCCTACA........................................ 1

....................................................................................................................................................................................................................GGAGCTGCATCAACCCTACA........................................ 1

............................................................................................................................................................................................................................ATCAACCCTACATGGACCC................................. 1

............................................................................................................................................................................................................................ATCAACCCTACATGGACCCT................................ 17

............................................................................................................................................................................................................................ATCAACCCTACATGGACCCTC............................... 14

............................................................................................................................................................................................................................ATCAACCCTACATGGACCCTCT.............................. 4

...............................................................................................................................................................................................................................AACCCTACATGGACCCTC............................... 1

.............................................................................................................................................................................................................................................CCTCTTTGGATTGAAGGGAGC.............. 1

..............................................................................................................................................................................................................................................CTCTTTGGATTGAAGGGAGC.............. 1

..............................................................................................................................................................................................................................................CTCTTTGGATTGAAGGGAGCT............. 1

...............................................................................................................................................................................................................................................TCTTTGGATTGAAGGGAGCTC............ 3

................................................................................................................................................................................................................................................CTTTGGATTGAAGGGAGC.............. 4

................................................................................................................................................................................................................................................CTTTGGATTGAAGGGAGCTCT........... 1

................................................................................................................................................................................................................................................CTTTGGATTGAAGGGAGCTCTG.......... 1

.................................................................................................................................................................................................................................................TTTGGATTGAAGGGAGCT............. 94

.................................................................................................................................................................................................................................................TTTGGATTGAAGGGAGCTC............ 23

.................................................................................................................................................................................................................................................TTTGGATTGAAGGGAGCTCT........... 8

.................................................................................................................................................................................................................................................TTTGGATTGAAGGGAGCTCTG.......... 344

.................................................................................................................................................................................................................................................TTTGGATTGAAGGGAGCTCTGC......... 7

..................................................................................................................................................................................................................................................TTGGATTGAAGGGAGCTC............ 2

..................................................................................................................................................................................................................................................TTGGATTGAAGGGAGCTCTG.......... 11

..................................................................................................................................................................................................................................................TTGGATTGAAGGGAGCTCTGC......... 10

..................................................................................................................................................................................................................................................TTGGATTGAAGGGAGCTCTGCA........ 1

...................................................................................................................................................................................................................................................TGGATTGAAGGGAGCTCTG.......... 5

>osa-MIR159b_MI0001093_Oryza_sativa_miR159b_stem-loop GSM361264

GGUUAUGAAGUGGAGCUCCUUUCGUUCCAAUGAAAGGUUUAUCUGAAGGGUGAUACAGCUGCUUGUUCAUGGUUCCCACUAUUCUAUCUCAUAGGAAAAGAGAUAGGCUUGUGGUUUGCAUGACCAAGGAGCCGAAUCAACUCCUUGCUGACCACUCUUUGGAUUGAAGGGAGCUCUGCAUCUUGAUC

(((((.((.(..((((((((((((.(((((.((..((((......(((((((((.(.(((.((((.(((((....((((...((((((((.........))))))))...))))....))))).)))).))).).))).)).))))...))))..)).))))).))))))))))))..).)).))))) (-79.30)

........................................................AGCTGCTTGTTCATGGTTCCC............................................................................................................... 1

..........................................................................................ATAGGAAAAGAGATAGGCT............................................................................... 1

..........................................................................................ATAGGAAAAGAGATAGGCTTGTGG.......................................................................... 1

..................................................................................................................TTTGCATGACCAAGGAGC........................................................ 1

..................................................................................................................TTTGCATGACCAAGGAGCCG...................................................... 3

..................................................................................................................TTTGCATGACCAAGGAGCCGA..................................................... 64

..................................................................................................................TTTGCATGACCAAGGAGCCGAA.................................................... 6

..................................................................................................................TTTGCATGACCAAGGAGCCGAAT................................................... 1

..................................................................................................................TTTGCATGACCAAGGAGCCGAATC.................................................. 1

...................................................................................................................TTGCATGACCAAGGAGCCGA..................................................... 2

..........................................................................................................................................................CTCTTTGGATTGAAGGGAGC.............. 1

..........................................................................................................................................................CTCTTTGGATTGAAGGGAGCT............. 1

...........................................................................................................................................................TCTTTGGATTGAAGGGAGCTC............ 3

............................................................................................................................................................CTTTGGATTGAAGGGAGC.............. 4

............................................................................................................................................................CTTTGGATTGAAGGGAGCTCT........... 1

............................................................................................................................................................CTTTGGATTGAAGGGAGCTCTG.......... 1

.............................................................................................................................................................TTTGGATTGAAGGGAGCT............. 94

.............................................................................................................................................................TTTGGATTGAAGGGAGCTC............ 23

.............................................................................................................................................................TTTGGATTGAAGGGAGCTCT........... 8

.............................................................................................................................................................TTTGGATTGAAGGGAGCTCTG.......... 344

.............................................................................................................................................................TTTGGATTGAAGGGAGCTCTGC......... 7

..............................................................................................................................................................TTGGATTGAAGGGAGCTC............ 2

..............................................................................................................................................................TTGGATTGAAGGGAGCTCTG.......... 11

..............................................................................................................................................................TTGGATTGAAGGGAGCTCTGC......... 10

..............................................................................................................................................................TTGGATTGAAGGGAGCTCTGCA........ 1

...............................................................................................................................................................TGGATTGAAGGGAGCTCTG.......... 5

>osa-MIR159c_MI0001094_Oryza_sativa_miR159c_stem-loop GSM361264

GAGGAGGAAGAGGAGCUCCUUUCGAUCCAAUUCAGGAGAGGAAGUGGUAGGAUGCAGCUGCCGAUUCAUGGAUACCUCUGGAGUGCAUGGCAGCAAUGCUGUAGGCCUGCACUUGCAUGGGUUUGCAUGACCCGGGAGAUGAACCCACCAUUGUCUUCCUCUAUUGAUUGGAUUGAAGGGAGCUCCACAUCUCUCUC

(((..(((.(.((((((((((((((((((((.(((.((((((((((((.((...((.((.(((..(((((.(.((((.(((((((((.(((.(((....)))..)))))))))).)).)))).).)))))..))).)).))..)).))))....)))))))).))))))))))))))))))))))).).)))..))) (-103.10)

............GAGCTCCTTTCGATCCAATTCA................................................................................................................................................................... 1

..................................GGAGAGGAAGTGGTAGGATGC.............................................................................................................................................. 3

....................................AGAGGAAGTGGTAGGATGC.............................................................................................................................................. 3

.........................................................................................................................TTTGCATGACCCGGGAGA.......................................................... 2

.........................................................................................................................TTTGCATGACCCGGGAGATG........................................................ 3

.........................................................................................................................TTTGCATGACCCGGGAGATGA....................................................... 246

.........................................................................................................................TTTGCATGACCCGGGAGATGAA...................................................... 3

..........................................................................................................................TTGCATGACCCGGGAGATGA....................................................... 8

..........................................................................................................................TTGCATGACCCGGGAGATGAA...................................................... 2

...........................................................................................................................TGCATGACCCGGGAGATGA....................................................... 2

.......................................................................................................................................................................TTGGATTGAAGGGAGCTC............ 2

>osa-MIR159d_MI0001095_Oryza_sativa_miR159d_stem-loop GSM361264

UGAUGUGAGGAGGAGCUCCUUUCGAUCCAAUUCAGGAGAGGAAGUGGUGGGAUGCAGCUGCCGGUUCAUGGAUACCUCUGCAGUUCAUGCCGGUAGGCCUGCACUUGCAUGGGUUUGCAUGACCUGGGAGAUGAACCUGCCAUUGUGUUCCUCUAUUGAUUGGAUUGAAGGGAGCUCCGGCUACACCUA

....(((((..((((((((((((((((((((.(((.(((((((((((..((...((.((.((((.(((((.(.((((.((((((.((.(((....))).)).)).)))).)))).).))))).)))).)).))..))..))))....))))))).)))))))))))))))))))))))..)).)))... (-100.10)

............GAGCTCCTTTCGATCCAATTCA........................................................................................................................................................... 1

.......................................................AGCTGCCGGTTCATGGATACC................................................................................................................. 1

.................................................................................................................TTTGCATGACCTGGGAGATGA....................................................... 2

...............................................................................................................................................................TTGGATTGAAGGGAGCTC............ 2

>osa-MIR159e_MI0001096_Oryza_sativa_miR159e_stem-loop GSM361264

GAUGAAGAAGAAGAGCUCCCUUUCGAUCCAAUUCAGGAGAGGAAGUGGUAGGAUGCAGCUGCCGGUUCAUGGAUACCUCUGGAGUGCAGGGCAAAUAGUCCUACCCUUUCAUGGGUUUGCAUGACUCGGGAGAUGAACCCGCCAUUGUCUUCCUCUAUUGAUUGGAUUGAAGGGAGCUCCUCUAGCUACAU

.(((.((.(((.((((((((((.(((((((((.(((.(((((((((((..((...((.((.(((..(((((.(.((((.(((((.(.(((((.....))))).).).)))).)))).).)))))..))).)).))..))..)))....)))))))).)))))))))))))))))))))).)))..)).))) (-89.20)

...................................GGAGAGGAAGTGGTAGGATGC....................................................................................................................................... 3

.....................................AGAGGAAGTGGTAGGATGC....................................................................................................................................... 3

........................................................AGCTGCCGGTTCATGGATACC.................................................................................................................. 1

.................................................................................................................................................................TTGGATTGAAGGGAGCTC............ 2

>osa-MIR159f_MI0001097_Oryza_sativa_miR159f_stem-loop GSM361264

GAAGAAGAAGACGAGCUCCCUUCGAUCCAAUCCAGGAGAGGAAGUGGUAGGAUGCAGCUGCCGGUUCAUGGAUACCUCUGCAGUGCAUGUCGUAGGCUUGCACUUGCAUGGGUUUGCAUGACCCGGGAGAUGAACCCACCAUUGUCUUCCUCUUAUGCUUGGAUUGAAGGGAGCUCUACACCUCUCUC

............((((((((((((((((((..((.(((((((((((((.((...((.((.((((.(((((.(.((((.(((((((((.((.....)).))))).)))).)))).).))))).)))).)).))..)).))))....))))))))).)).))))))))))))))))))............ (-95.50)

.............AGCTCCCTTCGATCCAATC............................................................................................................................................................ 1

.............AGCTCCCTTCGATCCAATCC........................................................................................................................................................... 1

.............AGCTCCCTTCGATCCAATCCA.......................................................................................................................................................... 1

..................................GGAGAGGAAGTGGTAGGATGC..................................................................................................................................... 3

....................................AGAGGAAGTGGTAGGATGC..................................................................................................................................... 3

.......................................................AGCTGCCGGTTCATGGATACC................................................................................................................ 1

................................................................................................................TTTGCATGACCCGGGAGA.......................................................... 2

................................................................................................................TTTGCATGACCCGGGAGATG........................................................ 3

................................................................................................................TTTGCATGACCCGGGAGATGA....................................................... 246

................................................................................................................TTTGCATGACCCGGGAGATGAA...................................................... 3

.................................................................................................................TTGCATGACCCGGGAGATGA....................................................... 8

.................................................................................................................TTGCATGACCCGGGAGATGAA...................................................... 2

..................................................................................................................TGCATGACCCGGGAGATGA....................................................... 2

.............................................................................................................................................................CTTGGATTGAAGGGAGCTCT........... 1

..............................................................................................................................................................TTGGATTGAAGGGAGCTC............ 2

>osa-MIR319b_MI0001099_Oryza_sativa_miR319b_stem-loop GSM361264

GAUGGAUGGAAGAGAGCGUCCUUCAGUCCACUCAUGGGCGGUGCUAGGGUCGAAUUAGCUGCCGACUCAUUCACCCACAUGCCAAGCAAGAAACGCUUGAGAUAGCGAAGCUUAGCAGAUGAGUGAAUGAAGCGGGAGGUAACGUUCCGAUCUCGCGCCGUCUUUGCUUGGACUGAAGGGUGCUCCCUCCUCCUCGA

((.(((.(((.(.((((..(((((((((((..((.(((((((((..(((((((((((.((.(((..((((((((.((..(((.((((......((((......))))..)))).)))..)).))))))))..))).)).))..))).)))))).))))))))).))..)))))))))))..))))).)))))))).. (-99.20)

........................................................AGCTGCCGACTCATTCACCCA........................................................................................................................ 2

.........................................................................................................................AGTGAATGAAGCGGGAGGTA........................................................ 2

.........................................................................................................................AGTGAATGAAGCGGGAGGTAA....................................................... 24

.......................................................................................................................................................................TTGGACTGAAGGGTGCTCCCT......... 1

>osa-MIR171i_MI0001155_Oryza_sativa_miR171i_stem-loop GSM361264

UAAAAAGAGGUAUUGGCGUGCCUCAAUCCGAAGGCAUGGCUGAUUACAGGCACCUCGACCGAUCUAGCGCAUGCAGCCAUGUUUCUUGGAUUGAGCCGCGUCAAUAUCUCUCCUUGCUUC

.....(((((((((((((((.(((((((((((((((((((((.......((..((.((....)).)).))...))))))))))).)))))))))).)))))))))))))))......... (-55.40)

..........TATTGGCGTGCCTCAATCCGA......................................................................................... 2

......................TCAATCCGAAGGCATGGCTGA............................................................................. 137

.......................CAATCCGAAGGCATGGCTGA............................................................................. 11

..........................................................................................TTGAGCCGCGTCAATATC............ 2

..........................................................................................TTGAGCCGCGTCAATATCT........... 1

..........................................................................................TTGAGCCGCGTCAATATCTC.......... 2

..........................................................................................TTGAGCCGCGTCAATATCTCT......... 56

...........................................................................................TGAGCCGCGTCAATATCTCT......... 1

>osa-MIR437_MI0001688_Oryza_sativa_miR437_stem-loop GSM361264

CCCUCUGUUUCAUAUUGUAUUGUGUUUUAGUUUUGUUGUAAGUCAAAAUUCUUUUACUUUGACCAAGUUUGUAGUAAAAUAAUUAACAUCUAAAAUACCAAAUAAAUACACUAUUGAGACAUAUUUCUUGGUUGAUUUUUCUAUAAACUUGGUCAAAGUUAGAGAAGUUUGACUUAGGACAAAACUAAACACCUUAUGAUAUGGAACAGAGGG

(((((((((((((((..((..((((((.(((((((((.(((((((((.((((((.(((((((((((((((((((.(((((................(((((..(((((..((....))...))))).)))))..))))).))))))))))))))))))).)))))).))))))))).)))))))))))))))..))..))))))))))))))) (-96.07)

.........................TTTAGTTTTGTTGTAAGTCAA....................................................................................................................................................................... 1

.....................................................TTACTTTGACCAAGTTTGTAG........................................................................................................................................... 1

......................................................TACTTTGACCAAGTTTGTAGTA......................................................................................................................................... 18

......................................................TACTTTGACCAAGTTTGTAGTAA........................................................................................................................................ 5

......................................................TACTTTGACCAAGTTTGTAGTAAA....................................................................................................................................... 34

......................................................TACTTTGACCAAGTTTGTAGTAAAA...................................................................................................................................... 3

.......................................................ACTTTGACCAAGTTTGTAGTA......................................................................................................................................... 1

.......................................................ACTTTGACCAAGTTTGTAGTAA........................................................................................................................................ 1

.......................................................ACTTTGACCAAGTTTGTAGTAAA....................................................................................................................................... 2

.......................................................ACTTTGACCAAGTTTGTAGTAAAA...................................................................................................................................... 7

.........................................................TTTGACCAAGTTTGTAGTAAA....................................................................................................................................... 11

.........................................................TTTGACCAAGTTTGTAGTAAAA...................................................................................................................................... 16

........................................................................................................................................................TCAAAGTTAGAGAAGTTTGAC........................................ 2

..........................................................................................................................................................AAAGTTAGAGAAGTTTGACTTAGG................................... 6

..................................................................................................................................................................AGAAGTTTGACTTAGGACAAAACT........................... 1

>osa-MIR441b_MI0001705_Oryza_sativa_miR441b_stem-loop GSM361264

UUAUCACCACUGACAUACUUCCUCCGUUUCACAAUGUAAGUCAUUUUAGUAAUUUUCAUAUUCAUAUUGAUGUUAAUGAAUCUAGACUCAUGUACCAUCAAUAUAAAUGUGGGAAAUGGUAGAAUGACUUACAUUAUGAAACAUAGGGAGCA

.................((((((..((((((.((((((((((((((((.((.((..((((((.(((((((((...((((........))))....))))))))).))))))..)).)).)))))))))))))))).))))))..)))))).. (-57.60)

.............CATACTTCCTCCGTTTCACAATGT................................................................................................................... 1

..............ATACTTCCTCCGTTTCACAA...................................................................................................................... 2

...............TACTTCCTCCGTTTCACAATGT................................................................................................................... 3

...............TACTTCCTCCGTTTCACAATG.................................................................................................................... 1

...............TACTTCCTCCGTTTCACAATGTA.................................................................................................................. 2

...............TACTTCCTCCGTTTCACAATGTAAG................................................................................................................ 1

...............TACTTCCTCCGTTTCACAATGTAA................................................................................................................. 1

................ACTTCCTCCGTTTCACAATGT................................................................................................................... 7

................ACTTCCTCCGTTTCACAATG.................................................................................................................... 1

................ACTTCCTCCGTTTCACAATGTA.................................................................................................................. 1

................ACTTCCTCCGTTTCACAATGTAA................................................................................................................. 3

.................CTTCCTCCGTTTCACAATGT................................................................................................................... 1

.................CTTCCTCCGTTTCACAATGTAAGT............................................................................................................... 7

.................CTTCCTCCGTTTCACAATGTA.................................................................................................................. 11

.................CTTCCTCCGTTTCACAATGTAA................................................................................................................. 2

..................TTCCTCCGTTTCACAATGT................................................................................................................... 1

..................TTCCTCCGTTTCACAATGTAAGT............................................................................................................... 2

..................TTCCTCCGTTTCACAATGTAAG................................................................................................................ 2

..................TTCCTCCGTTTCACAATGTAAGTC.............................................................................................................. 91

..................TTCCTCCGTTTCACAATGTA.................................................................................................................. 4

..................TTCCTCCGTTTCACAATGTAAGTCA............................................................................................................. 1

..................TTCCTCCGTTTCACAATGTAA................................................................................................................. 12

...................TCCTCCGTTTCACAATGT................................................................................................................... 1

...................TCCTCCGTTTCACAATGTAAGT............................................................................................................... 8

...................TCCTCCGTTTCACAATGTAAG................................................................................................................ 1

...................TCCTCCGTTTCACAATGTAAGTC.............................................................................................................. 33

...................TCCTCCGTTTCACAATGTA.................................................................................................................. 2

...................TCCTCCGTTTCACAATGTAAGTCA............................................................................................................. 54

...................TCCTCCGTTTCACAATGTAA................................................................................................................. 4

....................CCTCCGTTTCACAATGTAAGT............................................................................................................... 18

....................CCTCCGTTTCACAATGTAAGTCAT............................................................................................................ 16

....................CCTCCGTTTCACAATGTAAGTCAT............................................................................................................ 1

....................CCTCCGTTTCACAATGTAAGTCA............................................................................................................. 18

....................CCTCCGTTTCACAATGTAA................................................................................................................. 1

.....................CTCCGTTTCACAATGTAAGTCATT........................................................................................................... 35

.....................CTCCGTTTCACAATGTAAGT............................................................................................................... 4

.....................CTCCGTTTCACAATGTAAGTCAT............................................................................................................ 34

.....................CTCCGTTTCACAATGTAAGTC.............................................................................................................. 2

.....................CTCCGTTTCACAATGTAAGTCA............................................................................................................. 9

......................TCCGTTTCACAATGTAAGTCATTT.......................................................................................................... 17

......................TCCGTTTCACAATGTAAGTCATT........................................................................................................... 30

......................TCCGTTTCACAATGTAAGTCAT............................................................................................................ 42

......................TCCGTTTCACAATGTAAGTC.............................................................................................................. 6

......................TCCGTTTCACAATGTAAGTCA............................................................................................................. 119

.......................CCGTTTCACAATGTAAGTCATTTT......................................................................................................... 11

.......................CCGTTTCACAATGTAAGTCATTT.......................................................................................................... 1

.......................CCGTTTCACAATGTAAGTCATT........................................................................................................... 8

.......................CCGTTTCACAATGTAAGTCAT............................................................................................................ 35

.......................CCGTTTCACAATGTAAGTCAT............................................................................................................ 1

.......................CCGTTTCACAATGTAAGTC.............................................................................................................. 1

.......................CCGTTTCACAATGTAAGTCA............................................................................................................. 7

........................CGTTTCACAATGTAAGTCATT........................................................................................................... 2

..........................TTTCACAATGTAAGTCATT........................................................................................................... 1

..........................TTTCACAATGTAAGTCATTTTA........................................................................................................ 1

...........................TTCACAATGTAAGTCATTTTA........................................................................................................ 1

............................TCACAATGTAAGTCATTTTA........................................................................................................ 2

.........................................................ATATTCATATTGATGTTAATGAAT....................................................................... 5

..........................................................TATTCATATTGATGTTAA............................................................................ 1

...........................................................ATTCATATTGATGTTAATGAATCT..................................................................... 7

............................................................TTCATATTGATGTTAATGAAT....................................................................... 2

............................................................TTCATATTGATGTTAATGAATCTA.................................................................... 1

...............................................................ATATTGATGTTAATGAATCTA.................................................................... 4

...............................................................ATATTGATGTTAATGAATCTAGA.................................................................. 7

...............................................................ATATTGATGTTAATGAATCTAGAC................................................................. 7

..................................................................TTGATGTTAATGAATCTAGAC................................................................. 1

...............................................................................................CATCAATATAAATGTGGGAAAT................................... 1

................................................................................................ATCAATATAAATGTGGGAAA.................................... 1

................................................................................................ATCAATATAAATGTGGGAAATGGT................................ 1

........................................................................................................AAATGTGGGAAATGGTAGAATGAC........................ 1

.......................................................................................................................TAGAATGACTTACATTATGAA............ 10

.......................................................................................................................TAGAATGACTTACATTATGA............. 1

........................................................................................................................AGAATGACTTACATTATGAAACA......... 1

>osa-MIR445d_MI0001712_Oryza_sativa_miR445d_stem-loop GSM361264

UGGCCAAAAUUUAGUCCCUGUCACAUCAGAUGUUAUGACACUAAUUAAAAGUAUUAAACAUAGACUAAUGACAAACCCCAUUCCAUAACCCUGGAUUAAUUCGCGAGAUGAAUCUAUUGAGUCUAAUUAAUCAAUGAUUAGCCUAUGUGAUGCUACACUAAACAUGUGUUAAUUAUGGAUUAAUUAGGCUUAAAAAUUUUGUCUCACGAAUUAGCUCUCAUUUAUGCAAUUAUUAGUUUUGUAAGUAGUUUAUGUUUAAUAUCUAAAUUAGUGUAUAAACAUCCGAUGUGAUAGGGACUAAAGUUGGAUCCA

.((((((..((((((((((((((((((.((((((...(((((((((....(((((((((((((((((...(((((.(..(((.(((((....(..((((((((.((((..((....((((((((((((((((.(((((((((.(((((.............))))).))))))))).))))))))))))))))....))..)))).))))))))..)....))))).))).....).)))))...)))))))))))))))))...)))))))))...)))))).)))))))))))))))))).))))..)). (-126.72)

.........TTTAGTCCCTGTCACATCAGATGT....................................................................................................................................................................................................................................................................................... 1

.........TTTAGTCCCTGTCACATCAGA.......................................................................................................................................................................................................................................................................................... 1

............AGTCCCTGTCACATCAGA.......................................................................................................................................................................................................................................................................................... 1

.............GTCCCTGTCACATCAGATGTT...................................................................................................................................................................................................................................................................................... 1

.............GTCCCTGTCACATCAGATGT....................................................................................................................................................................................................................................................................................... 1

...................................................TATTAAACATAGACTAATGACA............................................................................................................................................................................................................................................... 1

.................................................................................................AATTCGCGAGATGAATCTATTGAG............................................................................................................................................................................................... 1

.....................................................................................................................................ATGATTAGCCTATGTGATGCTACA........................................................................................................................................................... 1

.........................................................................................................................................................................TAATTATGGATTAATTAGGCTT......................................................................................................................... 1

..........................................................................................................................................................................AATTATGGATTAATTAGGCT.......................................................................................................................... 1

..........................................................................................................................................................................AATTATGGATTAATTAGGCTTAA....................................................................................................................... 1

..........................................................................................................................................................................AATTATGGATTAATTAGGCTTAAA...................................................................................................................... 17

...........................................................................................................................................................................ATTATGGATTAATTAGGCTT......................................................................................................................... 1

...........................................................................................................................................................................ATTATGGATTAATTAGGCTTAAA...................................................................................................................... 1

...........................................................................................................................................................................ATTATGGATTAATTAGGCTTAAAA..................................................................................................................... 10

............................................................................................................................................................................TTATGGATTAATTAGGCTTAA....................................................................................................................... 1

............................................................................................................................................................................TTATGGATTAATTAGGCTTAAAA..................................................................................................................... 1

............................................................................................................................................................................TTATGGATTAATTAGGCTTAAAAA.................................................................................................................... 2

.............................................................................................................................................................................TATGGATTAATTAGGCTTAAAAAT................................................................................................................... 1

..............................................................................................................................................................................ATGGATTAATTAGGCTTAAAA..................................................................................................................... 3

...............................................................................................................................................................................TGGATTAATTAGGCTTAAA...................................................................................................................... 1

...............................................................................................................................................................................TGGATTAATTAGGCTTAAAA..................................................................................................................... 36

...............................................................................................................................................................................TGGATTAATTAGGCTTAAAAA.................................................................................................................... 7

...............................................................................................................................................................................TGGATTAATTAGGCTTAAAAAT................................................................................................................... 2

................................................................................................................................................................................GGATTAATTAGGCTTAAAA..................................................................................................................... 4

................................................................................................................................................................................GGATTAATTAGGCTTAAAAA.................................................................................................................... 1

.................................................................................................................................................................................GATTAATTAGGCTTAAAA..................................................................................................................... 1

.........................................................................................................................................................................................................TCTCACGAATTAGCTCTCATTTAT....................................................................................... 1

.................................................................................................................................................................................................................ATTAGCTCTCATTTATGCAA................................................................................... 1

...................................................................................................................................................................................................................................................................................TAAACATCCGATGTGATAGGGACT............. 4

...................................................................................................................................................................................................................................................................................TAAACATCCGATGTGATAGGGA............... 1

....................................................................................................................................................................................................................................................................................AAACATCCGATGTGATAGGGACTA............ 1

.....................................................................................................................................................................................................................................................................................AACATCCGATGTGATAGGGA............... 1

.....................................................................................................................................................................................................................................................................................AACATCCGATGTGATAGGGACT............. 1

.....................................................................................................................................................................................................................................................................................AACATCCGATGTGATAGGGACTA............ 1

.....................................................................................................................................................................................................................................................................................AACATCCGATGTGATAGGGACTAA........... 6

.....................................................................................................................................................................................................................................................................................AACATCCGATGTGATAGGGACT............. 1

.....................................................................................................................................................................................................................................................................................AACATCCGATGTGATAGGGACTA............ 1

.....................................................................................................................................................................................................................................................................................AACATCCGATGTGATAGGGA............... 1

......................................................................................................................................................................................................................................................................................ACATCCGATGTGATAGGGAC.............. 1

......................................................................................................................................................................................................................................................................................ACATCCGATGTGATAGGGACTAA........... 1

......................................................................................................................................................................................................................................................................................ACATCCGATGTGATAGGGACTAAA.......... 3

........................................................................................................................................................................................................................................................................................ATCCGATGTGATAGGGACT............. 1

........................................................................................................................................................................................................................................................................................ATCCGATGTGATAGGGACTA............ 1

..........................................................................................................................................................................................................................................................................................CCGATGTGATAGGGACTA............ 1

>osa-MIR807a_MI0005209_Oryza_sativa_miR807a_stem-loop GSM361264

AACCACACUAUCAAUAAAUAGAUUCACCCGUGAGAUGACUUGGUUGUUCCACAUAGGACAAUGACAUGCAUCCUAUUACCAGCCAUCUCAUGAAAUUAAUAGGAUGCCACGUCGACUUGGUUGUUCCACAUAGGACAAUGACAUGCAUCCUAUUACCAGCCAUCUCAUGAAAUUAAUAGGAUGCCACGUCCUCAUCCUAGGUAAAAUAACCACGUCAUCUCACAGGUGAAUCCAUUCAUUGAUAUUGUGGUU

(((((((.(((((((.(((.((((((((.((((((((((.((((((((..((.(((((....(((..(((((((((((......((.((((((.(((((((((((((...(((......(((((((......))))))))))..)))))))))))......)).)))))).)))))))))))))...)))....))))).))..)))))))).)))))))))).)))))))).))).))))))).))))))) (-108.10)

AACCACACTATCAATAAAT......................................................................................................................................................................................................................................... 1

.............ATAAATAGATTCACCCGTGAGATG....................................................................................................................................................................................................................... 1

...............................................TCCACATAGGACAATGACATGCAT..................................................................................................................................................................................... 1

...........................................................AATGACATGCATCCTATTACCAGC......................................................................................................................................................................... 1

.............................................................TGACATGCATCCTATTACCAGCCAT...................................................................................................................................................................... 1

.................................................................ATGCATCCTATTACCAGCCATCTC................................................................................................................................................................... 2

............................................................................................................................TCCACATAGGACAATGACATGCAT........................................................................................................ 1

........................................................................................................................................AATGACATGCATCCTATTACCAGC............................................................................................ 1

..........................................................................................................................................TGACATGCATCCTATTACCAGCCAT......................................................................................... 1

..............................................................................................................................................ATGCATCCTATTACCAGCCATCTC...................................................................................... 2

.............................................................................................................................................................................TAATAGGATGCCACGTCCTCA.......................................................... 1

..............................................................................................................................................................................AATAGGATGCCACGTCCTCAT......................................................... 1

..............................................................................................................................................................................AATAGGATGCCACGTCCTCATC........................................................ 1

...............................................................................................................................................................................ATAGGATGCCACGTCCTCAT......................................................... 1

................................................................................................................................................................................TAGGATGCCACGTCCTCATC........................................................ 2

...................................................................................................................................................................................GATGCCACGTCCTCATCCTA..................................................... 13

....................................................................................................................................................................................ATGCCACGTCCTCATCCTAG.................................................... 2

....................................................................................................................................................................................ATGCCACGTCCTCATCCTA..................................................... 2

.....................................................................................................................................................................................TGCCACGTCCTCATCCTA..................................................... 2

.....................................................................................................................................................................................TGCCACGTCCTCATCCTAGGTAAA............................................... 1

...............................................................................................................................................................................................................AACCACGTCATCTCACAGGTGAAT..................... 1

................................................................................................................................................................................................................ACCACGTCATCTCACAGGTGAATC.................... 2

..................................................................................................................................................................................................................CACGTCATCTCACAGGTGAATCCAT................. 1

..................................................................................................................................................................................................................CACGTCATCTCACAGGTGAAT..................... 2

..................................................................................................................................................................................................................CACGTCATCTCACAGGTG........................ 1

...................................................................................................................................................................................................................ACGTCATCTCACAGGTGAATCCAT................. 24

....................................................................................................................................................................................................................CGTCATCTCACAGGTGAATCCATT................ 1

>osa-MIR809e_MI0005225_Oryza_sativa_miR809e_stem-loop GSM361264

UACUCCCUCCGUUUCACAAUGUAAGUUAUUCUAACAUUUCCUACAUUUAUAUUAAUGUUAAUGAAUCUAGACAUCAUUAACAUCAAUAUGUCUAGAUUUAUUAACAUCAAUAUGAAUGUGAGAAAUGUUAGAAUAACUUACAUUGUGAAACGGAUGAAGUA

((((.(.(((((((((((((((((((((((((((((((((.((((((((((((.((((((((((((((((((((.(((......))))))))))))))))))))))).)))))))))))).))))))))))))))))))))))))))))))))).).)))) (-92.30)

TACTCCCTCCGTTTCACAATGTA.......................................................................................................................................... 2

TACTCCCTCCGTTTCACA............................................................................................................................................... 1

.ACTCCCTCCGTTTCACAATGT........................................................................................................................................... 3

.ACTCCCTCCGTTTCACAATGTAAG........................................................................................................................................ 1

.ACTCCCTCCGTTTCACAATGTAA......................................................................................................................................... 1

.ACTCCCTCCGTTTCACAA.............................................................................................................................................. 1

..CTCCCTCCGTTTCACAATGTAAGT....................................................................................................................................... 4

..CTCCCTCCGTTTCACAATGTAAG........................................................................................................................................ 1

..CTCCCTCCGTTTCACAATGTA.......................................................................................................................................... 2

..CTCCCTCCGTTTCACAATGTAA......................................................................................................................................... 1

...TCCCTCCGTTTCACAATGTAAGT....................................................................................................................................... 1

...TCCCTCCGTTTCACAATGTAA......................................................................................................................................... 1

....CCCTCCGTTTCACAATGT........................................................................................................................................... 1

....CCCTCCGTTTCACAATGTAAGT....................................................................................................................................... 11

....CCCTCCGTTTCACAATGTAAGTTA..................................................................................................................................... 1

....CCCTCCGTTTCACAATGTAA......................................................................................................................................... 3

.....CCTCCGTTTCACAATGTAAGT....................................................................................................................................... 18

.....CCTCCGTTTCACAATGTAAGTTAT.................................................................................................................................... 1

.....CCTCCGTTTCACAATGTAA......................................................................................................................................... 1

......CTCCGTTTCACAATGTAAGTT...................................................................................................................................... 1

......CTCCGTTTCACAATGTAAGTTATT................................................................................................................................... 2

......CTCCGTTTCACAATGTAAGT....................................................................................................................................... 4

......CTCCGTTTCACAATGTAAGTTA..................................................................................................................................... 1

.......TCCGTTTCACAATGTAAGTTATT................................................................................................................................... 3

.......TCCGTTTCACAATGTAAGTTATTC.................................................................................................................................. 19

........CCGTTTCACAATGTAAGTTATTCT................................................................................................................................. 23

........CCGTTTCACAATGTAAGTTATTC.................................................................................................................................. 2

...........TTTCACAATGTAAGTTATTCT................................................................................................................................. 1

............TTCACAATGTAAGTTATTCTA................................................................................................................................ 1

............................TTCTAACATTTCCTACATTTATAT............................................................................................................. 1

.............................TCTAACATTTCCTACATTTATATT............................................................................................................ 1

................................AACATTTCCTACATTTATATTAAT......................................................................................................... 1

................................................ATATTAATGTTAATGAATCTAGAC......................................................................................... 2

..................................................ATTAATGTTAATGAATCTAGACAT....................................................................................... 1

.....................................................AATGTTAATGAATCTAGACA........................................................................................ 1

......................................................ATGTTAATGAATCTAGACAT....................................................................................... 1

.....................................................................................ATATGTCTAGATTTATTAACATCA.................................................... 4

.........................................................................................GTCTAGATTTATTAACATCAATAT................................................ 1

.........................................................................................................ATCAATATGAATGTGAGAAATGTT................................ 1

.........................................................................................................ATCAATATGAATGTGAGAAAT................................... 1

..........................................................................................................TCAATATGAATGTGAGAA..................................... 1

.............................................................................................................ATATGAATGTGAGAAATGTTAGAA............................ 1

................................................................................................................................TAGAATAACTTACATTGTGAA............ 1

.................................................................................................................................AGAATAACTTACATTGTGAAACGG........ 23

.................................................................................................................................AGAATAACTTACATTGTGAAA........... 1

..................................................................................................................................GAATAACTTACATTGTGAAACGG........ 2

..................................................................................................................................GAATAACTTACATTGTGAAACGGA....... 19

...................................................................................................................................AATAACTTACATTGTGAAACGGA....... 3

...................................................................................................................................AATAACTTACATTGTGAAACGGAT...... 2

....................................................................................................................................ATAACTTACATTGTGAAACGGAT...... 1

......................................................................................................................................AACTTACATTGTGAAACGGAT...... 1

......................................................................................................................................AACTTACATTGTGAAACGGATGAA... 1

........................................................................................................................................CTTACATTGTGAAACGGATGAAGT. 1

........................................................................................................................................CTTACATTGTGAAACGGATGAAGT. 1

.........................................................................................................................................TTACATTGTGAAACGGATGAA... 2

.........................................................................................................................................TTACATTGTGAAACGGATGAAG.. 1

..........................................................................................................................................TACATTGTGAAACGGATGAAGT. 1

...........................................................................................................................................ACATTGTGAAACGGATGAAGTA 1

>osa-MIR809f_MI0005226_Oryza_sativa_miR809f_stem-loop GSM361264

UACUCCCUCCGUUUCACAAUUUAAGUCAUUCUAUCAUUUCUCACAUUCAUAUUAAUGUUAAUGAAUCUAGACAUAUAUAUCUAUCUAAAUUCAUUACCAUCAAUAUGAAUGUGAGAAAUGUUAGAAUGAUUUAAAUUGUGAAACGGAGGAAGUA

((((.((((((((((((((((((((((((((((.(((((((((((((((((((.(((.((((((((.((((............)))).)))))))).))).))))))))))))))))))).)))))))))))))))))))))))))))).)))) (-86.50)

TACTCCCTCCGTTTCACA........................................................................................................................................ 1

.ACTCCCTCCGTTTCACAA....................................................................................................................................... 1

.....CCTCCGTTTCACAATTTAAGT................................................................................................................................ 2

.....CCTCCGTTTCACAATTTAAGTCA.............................................................................................................................. 1

......CTCCGTTTCACAATTTAAGTCATT............................................................................................................................ 8

.......TCCGTTTCACAATTTAAGTCATT............................................................................................................................ 2

.......TCCGTTTCACAATTTAAGTCATTCT.......................................................................................................................... 1

.......TCCGTTTCACAATTTAAGTCAT............................................................................................................................. 9

.......TCCGTTTCACAATTTAAGTCATTC........................................................................................................................... 3

.......TCCGTTTCACAATTTAAGTCA.............................................................................................................................. 3

........CCGTTTCACAATTTAAGTCATTCT.......................................................................................................................... 20

........CCGTTTCACAATTTAAGTCATTC........................................................................................................................... 4

...........TTTCACAATTTAAGTCATTCT.......................................................................................................................... 2

...........TTTCACAATTTAAGTCATTCTA......................................................................................................................... 1

.......................AGTCATTCTATCATTTCTCA............................................................................................................... 1

........................GTCATTCTATCATTTCTCACAT............................................................................................................ 1

.............................TCTATCATTTCTCACATTCATAT...................................................................................................... 1

................................................ATATTAATGTTAATGAATCTAGAC.................................................................................. 2

..................................................ATTAATGTTAATGAATCTAGACAT................................................................................ 1

....................................................TAATGTTAATGAATCTAGACATAT.............................................................................. 1

.....................................................AATGTTAATGAATCTAGACA................................................................................. 1

.....................................................AATGTTAATGAATCTAGACATA............................................................................... 2

.....................................................AATGTTAATGAATCTAGACATATA............................................................................. 4

......................................................ATGTTAATGAATCTAGACATATAT............................................................................ 2

......................................................ATGTTAATGAATCTAGACAT................................................................................ 1

......................................................ATGTTAATGAATCTAGACATA............................................................................... 2

......................................................ATGTTAATGAATCTAGACATAT.............................................................................. 2

......................................................ATGTTAATGAATCTAGACATATA............................................................................. 11

......................................................ATGTTAATGAATCTAGACATATAT............................................................................ 2

.......................................................TGTTAATGAATCTAGACATATATAT.......................................................................... 1

.......................................................TGTTAATGAATCTAGACATA............................................................................... 1

.......................................................TGTTAATGAATCTAGACATA............................................................................... 1

........................................................GTTAATGAATCTAGACATATATAT.......................................................................... 6

........................................................GTTAATGAATCTAGACATATAT............................................................................ 1

.........................................................TTAATGAATCTAGACATATATAT.......................................................................... 2

.........................................................TTAATGAATCTAGACATATAT............................................................................ 1

...........................................................AATGAATCTAGACATATATAT.......................................................................... 2

............................................................ATGAATCTAGACATATATATCTATC..................................................................... 1

..............................................................GAATCTAGACATATATATCTATCT.................................................................... 1

........................................................................................ATTCATTACCATCAATATGAATGT.......................................... 2

........................................................................................ATTCATTACCATCAATAT................................................ 1

..................................................................................................ATCAATATGAATGTGAGAAATGTT................................ 1

..................................................................................................ATCAATATGAATGTGAGAAAT................................... 1

...................................................................................................TCAATATGAATGTGAGAA..................................... 1

......................................................................................................ATATGAATGTGAGAAATGTTAGAA............................ 1

..................................................................................................................................TTAAATTGTGAAACGGAGGAAG.. 1

>osa-MIR820a_MI0005263_Oryza_sativa_miR820a_stem-loop GSM361264

UGCGUCGGCCUCGUGGAUGGACCAGGAGCUCAACAUUCCUUAAGGUUGUUCUUUCAAACCCAUACAAGGUUCCACCGCCUGCAUUGUUCCAAGAGUGUCUUGGAUGAAAGUAGGAAGUGGAACCUUGUUAGGGUUGGAACGAACUGCCUUAAGGAAGUCGAUGCUCCAGGUCCGUCCAGGAGGACGACUCA

.(.((((.((((.((((((((((.(((((((.((.(((((((((((.((((.(((.(((((..((((((((((((..(((((.((..((((((.....))))))..)).)))))..))))))))))))..))))).))).)))).))))))))))))).)).))))).)))))))))).)))).)))).). (-118.80)

....TCGGCCTCGTGGATGGACCAGG..................................................................................................................................................................... 31

....TCGGCCTCGTGGATGGACCAGGA.................................................................................................................................................................... 52

....TCGGCCTCGTGGATGGACCAGGAG................................................................................................................................................................... 3574

.....CGGCCTCGTGGATGGACCAGG..................................................................................................................................................................... 1

.....CGGCCTCGTGGATGGACCAGGAG................................................................................................................................................................... 20

.....CGGCCTCGTGGATGGACCAGGAGC.................................................................................................................................................................. 1

......GGCCTCGTGGATGGACCAGGAG................................................................................................................................................................... 17

........CCTCGTGGATGGACCAGGAGC.................................................................................................................................................................. 1

............................................................................GCCTGCATTGTTCCAAGAGTGTCT........................................................................................... 6

.............................................................................CCTGCATTGTTCCAAGAGTGTCTT.......................................................................................... 1

..............................................................................CTGCATTGTTCCAAGAGTGTCTTG......................................................................................... 3

....................................................................................................................GTGGAACCTTGTTAGGGTTGGAA.................................................... 1

............................................................................................................................................GAACTGCCTTAAGGAAGTCGATGCT.......................... 3

.............................................................................................................................................AACTGCCTTAAGGAAGTCGATGCT.......................... 8

.............................................................................................................................................AACTGCCTTAAGGAAGTCGATGCTC......................... 19

..............................................................................................................................................ACTGCCTTAAGGAAGTCGATGCTCC........................ 1

................................................................................................................................................TGCCTTAAGGAAGTCGATGCTC......................... 3

................................................................................................................................................TGCCTTAAGGAAGTCGATGCTCCA....................... 1

.........................................................................................................................................................GAAGTCGATGCTCCAGGTCCGTCCA............. 3

>osa-MIR820b_MI0005264_Oryza_sativa_miR820b_stem-loop GSM361264

GUACAUGCGUCGGCCUCGUGGAUGGACCAGGAGCUCGACGUUCCUUAAGGCCGUUCUUUCCGACCCAUACAAGGUUCCGUCGCCUGCAUUGUUCCAAGAGUGUCUUGGACGAAAGCAGGAAGUGGAACCUUGUUAAGGUCGGAACGAACUGCCUUAAGGAAGUCGAUGCUUUAGGUCCGUCCACGAGGACGACGCAGCUAG

.....(((((((.(((((((((((((((..(((((((((.(((((((((((.((((.((((((((...((((((((((((..(((((.((((.(((((.....)))))))))..)))))..))))))))))))...)))))))).)))).)))))))))))))))).))))..))))))))))))))).)))))))..... (-136.70)

.........TCGGCCTCGTGGATGGACCAGG.......................................................................................................................................................................... 31

.........TCGGCCTCGTGGATGGACCAGGA......................................................................................................................................................................... 52

.........TCGGCCTCGTGGATGGACCAGGAG........................................................................................................................................................................ 3574

..........CGGCCTCGTGGATGGACCAGG.......................................................................................................................................................................... 1

..........CGGCCTCGTGGATGGACCAGGAG........................................................................................................................................................................ 20

..........CGGCCTCGTGGATGGACCAGGAGC....................................................................................................................................................................... 1

...........GGCCTCGTGGATGGACCAGGAG........................................................................................................................................................................ 17

.............CCTCGTGGATGGACCAGGAGC....................................................................................................................................................................... 1

.................................................................................GCCTGCATTGTTCCAAGAGTGTCT................................................................................................ 6

..................................................................................CCTGCATTGTTCCAAGAGTGTCTT............................................................................................... 1

...................................................................................CTGCATTGTTCCAAGAGTGTCTTG.............................................................................................. 3

.......................................................................................................................AAGTGGAACCTTGTTAAGGTCGGA.......................................................... 1

.........................................................................................................................GTGGAACCTTGTTAAGGTCGGAA......................................................... 1

.........................................................................................................................GTGGAACCTTGTTAAGGTCGGAAC........................................................ 3

...........................................................................................................................GGAACCTTGTTAAGGTCGGAAC........................................................ 1

.................................................................................................................................................GAACTGCCTTAAGGAAGTCGATGCT............................... 3

..................................................................................................................................................AACTGCCTTAAGGAAGTCGATGCT............................... 8

..................................................................................................................................................AACTGCCTTAAGGAAGTCGATGCTTT............................. 1

>osa-MIR820c_MI0005265_Oryza_sativa_miR820c_stem-loop GSM361264

UGCGUCGGCCUCGUGGAUGGACCAGGAGCUCGACAUUCCUUAAGGUCGUUCUUUCCGACCCAUACAAGGUUCCGCUGCCUGCAUUGUUCCAAGAGUGUCUUGGACAAAAGCAGGAAGUGGAACCUUGUUAAGGUCGGAACGAACUGCCUUAAGGAAGUCGAUGCUCCAGGUCCGUCCACGAGGACGACGCA

.((((((.(((((((((((((((.((((((((((.(((((((((((.((((.((((((((...(((((((((((((.(((((.((((.(((((.....)))))))))..))))).)))))))))))))...)))))))).)))).)))))))))))))))).))))).))))))))))))))).)))))). (-143.00)

....TCGGCCTCGTGGATGGACCAGG..................................................................................................................................................................... 31

....TCGGCCTCGTGGATGGACCAGGA.................................................................................................................................................................... 52

....TCGGCCTCGTGGATGGACCAGGAG................................................................................................................................................................... 3574

.....CGGCCTCGTGGATGGACCAGG..................................................................................................................................................................... 1

.....CGGCCTCGTGGATGGACCAGGAG................................................................................................................................................................... 20

.....CGGCCTCGTGGATGGACCAGGAGC.................................................................................................................................................................. 1

......GGCCTCGTGGATGGACCAGGAG................................................................................................................................................................... 17

........CCTCGTGGATGGACCAGGAGC.................................................................................................................................................................. 1

....................ACCAGGAGCTCGACATTCCTTA..................................................................................................................................................... 1

.......................................TTAAGGTCGTTCTTTCCGA..................................................................................................................................... 1

.......................................TTAAGGTCGTTCTTTCCGACCC.................................................................................................................................. 1

.......................................TTAAGGTCGTTCTTTCCGACCCA................................................................................................................................. 4

.....................................................TCCGACCCATACAAGGTTCCGC.................................................................................................................... 1

........................................................................CGCTGCCTGCATTGTTCCAAGAGT............................................................................................... 1

...........................................................................TGCCTGCATTGTTCCAAGAGTGTC............................................................................................ 1

............................................................................GCCTGCATTGTTCCAAGAGTGTCT........................................................................................... 6

.............................................................................CCTGCATTGTTCCAAGAGTGTCTT.......................................................................................... 1

..............................................................................CTGCATTGTTCCAAGAGTGTCTTG......................................................................................... 3

..................................................................................................................AAGTGGAACCTTGTTAAGGTCGGA..................................................... 1

....................................................................................................................GTGGAACCTTGTTAAGGTCGGAA.................................................... 1

....................................................................................................................GTGGAACCTTGTTAAGGTCGGAAC................................................... 3

......................................................................................................................GGAACCTTGTTAAGGTCGGAAC................................................... 1

............................................................................................................................................GAACTGCCTTAAGGAAGTCGATGCT.......................... 3

.............................................................................................................................................AACTGCCTTAAGGAAGTCGATGCT.......................... 8

.............................................................................................................................................AACTGCCTTAAGGAAGTCGATGCTC......................... 19

..............................................................................................................................................ACTGCCTTAAGGAAGTCGATGCTCC........................ 1

................................................................................................................................................TGCCTTAAGGAAGTCGATGCTC......................... 3

................................................................................................................................................TGCCTTAAGGAAGTCGATGCTCCA....................... 1

.........................................................................................................................................................GAAGTCGATGCTCCAGGTCCGTCCA............. 3

.............................................................................................................................................................TCGATGCTCCAGGTCCGTCCAC............ 1

.....................................................................................................................................................................CCAGGTCCGTCCACGAGGACG..... 1

.....................................................................................................................................................................CCAGGTCCGTCCACGAGGACGACG.. 1

>osa-MIR821a_MI0005266_Oryza_sativa_miR821a_stem-loop GSM361264

GAUAUCAGCUGAAAAAGUCAUCAACAAAAAAGUUGAAUAACUCAUCAAGAUCUAAAACUUUUAUUUUGGUCAUUUCUUCAUACGACAAAGUGAUAGUAACAUUGUUCACAGAAUUGACAUAUCUCUUAUCUGGUUUUAUAAACUAUAACACAUAUAUGUAAAAUUUGUGAAUAAUAUUACUAACAUUUCGUCAGAUGAAGAAAUAAAAAAAUAAAAGUUAUAGAUCUUGAUGAGUUAUUCAACUUUGUUGUUGAUGACUUUUUCAAUUGAAAUC

(((.((((.((((((((((((((((((.((((((((((((((((((((((((((.((((((((((((..(.((((((((((..(((.(((((.((((((.((((((((((((.((.((((((........(((((.....)))))........)))))).)).)))))))))))).)))))).))))).)))..)))))))))))..)))))))))))).)))))))))))))))))))))))))).)))))))))))))))))).)))).))) (-126.69)

....................................ATAACTCATCAAGATCTAAAACTT...................................................................................................................................................................................................................... 3

......................................AACTCATCAAGATCTAAAACTTTT.................................................................................................................................................................................................................... 1

........................................CTCATCAAGATCTAAAACTTTTAT.................................................................................................................................................................................................................. 1

.........................................TCATCAAGATCTAAAACTTTTATT................................................................................................................................................................................................................. 1

..........................................CATCAAGATCTAAAACTTTTAT.................................................................................................................................................................................................................. 1

..........................................CATCAAGATCTAAAACTTTTA................................................................................................................................................................................................................... 7

...........................................ATCAAGATCTAAAACTTTTATTTT............................................................................................................................................................................................................... 2

................................................................................TACGACAAAGTGATAGTAACATT........................................................................................................................................................................... 1

....................................................................................ACAAAGTGATAGTAACATT........................................................................................................................................................................... 1

.....................................................................................CAAAGTGATAGTAACATT........................................................................................................................................................................... 1

......................................................................................AAAGTGATAGTAACATTGTTCACA.................................................................................................................................................................... 1

.................................................................................................................TTGACATATCTCTTATCTGGTTTT......................................................................................................................................... 1

.......................................................................................................................................................................TGAATAATATTACTAACATTTCGT................................................................................... 1

.........................................................................................................................................................................AATAATATTACTAACATTTCGTC.................................................................................. 1

.........................................................................................................................................................................AATAATATTACTAACATTTCGTCA................................................................................. 1

..........................................................................................................................................................................ATAATATTACTAACATTTCG.................................................................................... 1

............................................................................................................................................................................AATATTACTAACATTTCGTCAGAT.............................................................................. 8

.............................................................................................................................................................................ATATTACTAACATTTCGTCAGAT.............................................................................. 3

...............................................................................................................................................................................ATTACTAACATTTCGTCAGATGA............................................................................ 1

...............................................................................................................................................................................ATTACTAACATTTCGTCAGATGAA........................................................................... 5

.................................................................................................................................................................................TACTAACATTTCGTCAGATGAAG.......................................................................... 1

..................................................................................................................................................................................ACTAACATTTCGTCAGATGAAGA......................................................................... 1

..................................................................................................................................................................................ACTAACATTTCGTCAGATGAAGAA........................................................................ 2

................................................................................................................................................................................................................AAATAAAAGTTATAGATCTTGA............................................ 1

..................................................................................................................................................................................................................ATAAAAGTTATAGATCTTGAT........................................... 1

...................................................................................................................................................................................................................TAAAAGTTATAGATCTTGATG.......................................... 2

.....................................................................................................................................................................................................................AAAGTTATAGATCTTGATGAGTTA..................................... 1

..........................................................................................................................................................................................................................TATAGATCTTGATGAGTTATT................................... 1

.................................................................................................................................................................................................................................CTTGATGAGTTATTCAACTTTGTT......................... 1

....................................................................................................................................................................................................................................GATGAGTTATTCAACTTTGTT......................... 1

.................................................................................................................................................................................................................................................ACTTTGTTGTTGATGACTTTTT........... 1

.................................................................................................................................................................................................................................................ACTTTGTTGTTGATGACTTTT............ 1

>osa-MIR821b_MI0005267_Oryza_sativa_miR821b_stem-loop GSM361264

CUAAAUGAUUUCAAUUGAAAAAGUCAUCAACAAAAAAGUUGAAUAACUUAUCAAGAUCUAAAACUUUUAUUUUUGUUAUUUCUUCAUCCGACGAAAUGUUAGUAAUAUUAUUCACAAAUUUUACAUAUAUGUGUUAAAGUUUAUAAAACCAGAUAAGAGAUAUGACAAUUUUAUAAAUAAUGUUACCAUCACUUUGUCGUAUGAAGAAAUAACCACAAUAAAAGUUUUAGAUCUUGAAGAGUUAUUCAACUUUGUUGUUGAUGACUUUUUCAGUUGAUAUCAUUUAG

(((((((((.(((((((((((((((((((((((.(((((((((((((((.(((((((((((((((((((((...(((((((((((((.(((((((.((...(((((((((((...(((((((((((....))).))))))))..........((((((..........)))))).)))))))))))...)).))))))).)))))))))))))...))))))))))))))))))))).))))))))))))))).))))))))))))))))))))))).))))))))) (-130.00)

....ATGATTTCAATTGAAAAAGTCATC................................................................................................................................................................................................................................................................... 1

.................................................ATCAAGATCTAAAACTTTTATTTT...................................................................................................................................................................................................................... 2

..................................................TCAAGATCTAAAACTTTTATTTTT..................................................................................................................................................................................................................... 2

...................................................................TATTTTTGTTATTTCTTCATC....................................................................................................................................................................................................... 1

..............................................................................TTTCTTCATCCGACGAAATGTTAG......................................................................................................................................................................................... 1

...............................................................................TTCTTCATCCGACGAAATGTTAGT........................................................................................................................................................................................ 1

................................................................................TCTTCATCCGACGAAATGTTAGT........................................................................................................................................................................................ 1

.................................................................................CTTCATCCGACGAAATGTTAGTA....................................................................................................................................................................................... 1

..................................................................................TTCATCCGACGAAATGTTAGTAAT..................................................................................................................................................................................... 9

..................................................................................TTCATCCGACGAAATGTTAGTA....................................................................................................................................................................................... 1

...................................................................................TCATCCGACGAAATGTTAGTAAT..................................................................................................................................................................................... 2

...................................................................................TCATCCGACGAAATGTTAGTAATA.................................................................................................................................................................................... 1

.....................................................................................ATCCGACGAAATGTTAGTAATATT.................................................................................................................................................................................. 24

.....................................................................................ATCCGACGAAATGTTAGTAATAT................................................................................................................................................................................... 5

.....................................................................................ATCCGACGAAATGTTAGTAATATTA................................................................................................................................................................................. 1

......................................................................................TCCGACGAAATGTTAGTAATATT.................................................................................................................................................................................. 2

......................................................................................TCCGACGAAATGTTAGTAAT..................................................................................................................................................................................... 1

......................................................................................TCCGACGAAATGTTAGTAATATTA................................................................................................................................................................................. 1

......................................................................................TCCGACGAAATGTTAGTAATA.................................................................................................................................................................................... 1

.......................................................................................CCGACGAAATGTTAGTAATATTAT................................................................................................................................................................................ 9

.......................................................................................CCGACGAAATGTTAGTAATATTA................................................................................................................................................................................. 1

.........................................................................................GACGAAATGTTAGTAATATTATT............................................................................................................................................................................... 1

..........................................................................................ACGAAATGTTAGTAATATTATTCA............................................................................................................................................................................. 1

...........................................................................................CGAAATGTTAGTAATATTAT................................................................................................................................................................................ 1

........................................................................................................................................................................................................................................................ACTTTGTTGTTGATGACTTTTT................. 1

........................................................................................................................................................................................................................................................ACTTTGTTGTTGATGACTTTT.................. 1

>osa-MIR821c_MI0005268_Oryza_sativa_miR821c_stem-loop GSM361264

UCAACUGAAAAAGUCAUCAACAAAAAAGUUGAAUAACUCAUCAACAUCUAAAACUUUUAUUUUGGUUAUUUCUUCAUACGACAAAGUGAUAGUAACAUUAUUCACAAAAUUGACAUAACUCUUAUCUGGUUUUAUAAACUAUAACACAUAUAUGUAAAAUUUGUGAAUAAUUUUCUAACAUUUUGUCAGAUGAAGAAACAACAAAAAUAAAAGUUUUAGAUCUUGAUGAGUUAUUCAACUUUGUUGUUGAUGACUUUUUCAAUUGA

((((.((((((((((((((((((.((((((((((((((((((((.((((((((((((((((((.(((.(((((((((..(((((((((.(((.((.((((((((((((.((.(((((.........(((((.....))))).........))))).)).)))))))))))).))))).)))))))))..))))))))).))).)))))))))))))))))).)))))))))))))))))))).)))))))))))))))))).)))) (-122.07)

............................................................................TACGACAAAGTGATAGTAACATT....................................................................................................................................................................... 1

..............................................................................CGACAAAGTGATAGTAACATTATT.................................................................................................................................................................... 1

................................................................................ACAAAGTGATAGTAACATT....................................................................................................................................................................... 1

.................................................................................CAAAGTGATAGTAACATT....................................................................................................................................................................... 1

..........................................................................................................................................................................................................AAAAATAAAAGTTTTAGATCTTGA........................................ 2

...........................................................................................................................................................................................................AAAATAAAAGTTTTAGATCTTGAT....................................... 2

.............................................................................................................................................................................................................AATAAAAGTTTTAGATCTTGATGA..................................... 1

..............................................................................................................................................................................................................ATAAAAGTTTTAGATCTTGATG...................................... 1

..............................................................................................................................................................................................................ATAAAAGTTTTAGATCTTGATGAG.................................... 1

...............................................................................................................................................................................................................TAAAAGTTTTAGATCTTGATG...................................... 7

................................................................................................................................................................................................................AAAAGTTTTAGATCTTGATGAGTT.................................. 1

..................................................................................................................................................................................................................AAGTTTTAGATCTTGATGAGTTAT................................ 3

.............................................................................................................................................................................................................................CTTGATGAGTTATTCAACTTTGTT..................... 1

................................................................................................................................................................................................................................GATGAGTTATTCAACTTTGTT..................... 1

.............................................................................................................................................................................................................................................ACTTTGTTGTTGATGACTTTTT....... 1

.............................................................................................................................................................................................................................................ACTTTGTTGTTGATGACTTTT........ 1

>osa-MIR1427_MI0006967_Oryza_sativa_miR1427_stem-loop GSM361264

UGGCUGCGCGCGACCGCGCCGUCCCGCAGGCCAGCGCCGCGCCACCGCGCGGUUCCGCAGCAGGGACAUGCGCCACCCGUUACAGCCAACUGAAAUCUGCACUGAUUUGAUUACAUGCGCACGUAUAUAUGCAGUGCAUGCAGCGAGUGUGCGCUGUUCCUGGAUUCGGUCGCGCGUGGCCGUGCUGCGGAACCGUGCGGUGGCGCGGCGCCGGCCUGCGGGACCGCGCAGUCGCGCGCAGCCAAGCCACGGGACCG

((((((((((((((.((((.((((((((((((.(((((((((((((((((((((((((((((.((.(((((((.(((.....(((.......(((((......)))))....(((.(((((((......((((.....)))).....))))))))))..))).....))).))))))).)).))))))))))))))))))))))))))))).)))))))))))).)))).)))))))))))))).....((....)) (-190.20)

....................................................TTCCGCAGCAGGGACATGCGCC....................................................................................................................................................................................... 1

.....................................................TCCGCAGCAGGGACATGCGCC....................................................................................................................................................................................... 1

...................................................................................................................................................................................CCGTGCTGCGGAACCGTGCGGTGG...................................................... 6

....................................................................................................................................................................................CGTGCTGCGGAACCGTGCGGTGGC..................................................... 2

.....................................................................................................................................................................................GTGCTGCGGAACCGTGCGGT........................................................ 1

.....................................................................................................................................................................................GTGCTGCGGAACCGTGCGGTGG...................................................... 2

.....................................................................................................................................................................................GTGCTGCGGAACCGTGCGGTGGC..................................................... 4

.....................................................................................................................................................................................GTGCTGCGGAACCGTGCGGTGGCG.................................................... 1

......................................................................................................................................................................................TGCTGCGGAACCGTGCGGTGGC..................................................... 2

........................................................................................................................................................................................CTGCGGAACCGTGCGGTGGC..................................................... 2

........................................................................................................................................................................................CTGCGGAACCGTGCGGTGGCG.................................................... 1

........................................................................................................................................................................................CTGCGGAACCGTGCGGTGGCGC................................................... 1

.........................................................................................................................................................................................TGCGGAACCGTGCGGTGGCG.................................................... 4

.........................................................................................................................................................................................TGCGGAACCGTGCGGTGGCGC................................................... 58

..........................................................................................................................................................................................GCGGAACCGTGCGGTGGCGC................................................... 1

..........................................................................................................................................................................................GCGGAACCGTGCGGTGGCGCGGCG............................................... 1

........................................................................................................................................................................................................................TGCGGGACCGCGCAGTCGCGCGCA................. 1

.........................................................................................................................................................................................................................GCGGGACCGCGCAGTCGCGCGCA................. 1

>osa-MIR1430_MI0006970_Oryza_sativa_miR1430_stem-loop GSM361264

AUGAGGAGAUUGCCUCUGUUAGCCAAGAAUGGCUUGCCUAUCUCCACUAUUUGGUUCAUCACUGGAACACACUUGGGGUUCUCAGAUGGUGGAUGAAAUAUGGAAGAUGGUGAGCCUUCCUGGCUAAGAGAGUGAUUCUCAU

(((((((.....(((((.(((((((.(((.((((..((.(((((((.(((((.(((((((((((((((.(....)..)))).))).)))))))).)))))))).))))))..))))))).))))))).)))).).))))))) (-68.10)

.TGAGGAGATTGCCTCTGTTAGCC...................................................................................................................... 4

...AGGAGATTGCCTCTGTTAGCCAAGAA................................................................................................................. 1

...................TAGCCAAGAATGGCTTGCCTAT..................................................................................................... 1

...................TAGCCAAGAATGGCTTGCCTATC.................................................................................................... 6

...................TAGCCAAGAATGGCTTGCCTATCT................................................................................................... 1

....................AGCCAAGAATGGCTTGCCTATC.................................................................................................... 1

........................................................................TTGGGGTTCTCAGATGGTGGATGAA............................................. 1

.....................................................................................ATGGTGGATGAAATATGGAAG.................................... 1

......................................................................................TGGTGGATGAAATATGGAAGA................................... 43

.......................................................................................GGTGGATGAAATATGGAAGA................................... 1

.................................................................................................ATATGGAAGATGGTGAGCCTT........................ 1

>osa-MIR1432_MI0006972_Oryza_sativa_miR1432_stem-loop GSM361264

CCUGUGAUCAGGAGAGAUGACACCGACAUCGCCGGAAUUCGUUCUUGGUCUUGUGCCAUGAUGAAUUGAUGGUCCGUUUGAUGCAGGUGUCAUCUCCCCUGAACAUAGG

((((((.(((((.(((((((((((..(((((.(((((((((((..((((.....)))).)))))))......))))..)))))..))))))))))).))))).)))))) (-55.80)

.CTGTGATCAGGAGAGATGACA....................................................................................... 2

..TGTGATCAGGAGAGATGACAC...................................................................................... 3

.....GATCAGGAGAGATGACACCGA................................................................................... 32

.....GATCAGGAGAGATGACACCGAC.................................................................................. 6

.....GATCAGGAGAGATGACACCGACA................................................................................. 33

......ATCAGGAGAGATGACACC..................................................................................... 16

......ATCAGGAGAGATGACACCG.................................................................................... 14

......ATCAGGAGAGATGACACCGA................................................................................... 99

......ATCAGGAGAGATGACACCGAC.................................................................................. 9315

......ATCAGGAGAGATGACACCGACA................................................................................. 39380

......ATCAGGAGAGATGACACCGACAT................................................................................ 201

......ATCAGGAGAGATGACACCGACATC............................................................................... 16

.......TCAGGAGAGATGACACCG.................................................................................... 9

.......TCAGGAGAGATGACACCGA................................................................................... 236

.......TCAGGAGAGATGACACCGAC.................................................................................. 7005

.......TCAGGAGAGATGACACCGACA................................................................................. 3681

.......TCAGGAGAGATGACACCGACAT................................................................................ 13

.......TCAGGAGAGATGACACCGACATCG.............................................................................. 1

........CAGGAGAGATGACACCGA................................................................................... 2

........CAGGAGAGATGACACCGAC.................................................................................. 102

........CAGGAGAGATGACACCGACA................................................................................. 56

........CAGGAGAGATGACACCGACAT................................................................................ 1

.........AGGAGAGATGACACCGAC.................................................................................. 206

.........AGGAGAGATGACACCGACA................................................................................. 274

.........AGGAGAGATGACACCGACAT................................................................................ 1

.............GAGATGACACCGACATCGCCG........................................................................... 4

.............GAGATGACACCGACATCGCCGGAA........................................................................ 1

..............AGATGACACCGACATCGCCGG.......................................................................... 1

...............GATGACACCGACATCGCCGG.......................................................................... 1

..............................................................GAATTGATGGTCCGTTTGATG.......................... 1

.............................................................................TTGATGCAGGTGTCATCTCCC........... 1

..................................................................................GCAGGTGTCATCTCCCCTGAAC..... 1

...................................................................................CAGGTGTCATCTCCCCTGAA...... 4

...................................................................................CAGGTGTCATCTCCCCTGAAC..... 66

...................................................................................CAGGTGTCATCTCCCCTGAACA.... 16

....................................................................................AGGTGTCATCTCCCCTGAAC..... 3

....................................................................................AGGTGTCATCTCCCCTGAACA.... 1

.....................................................................................GGTGTCATCTCCCCTGAAC..... 1

.....................................................................................GGTGTCATCTCCCCTGAACA.... 2

.....................................................................................GGTGTCATCTCCCCTGAACAT... 4

>osa-MIR444b_MI0006974_Oryza_sativa_miR444b_stem-loop GSM361264

AUGCAAGGGGAUGGUGACAAGCUUGUGGCAGCAACUGCACAUCUUGCAAGAAAAUCUUUAGGGUUUUUCAGACCAUACCGAUGAUUUUCUUGCAAGUUGUGCAGUUGUUGUCUCAAGCUUGCUGCCUCCCUUUGCCAA

..(((((((((.(((..((((((((.(((((((((((((((.((((((((((((((.....((((.....))))........)))))))))))))).))))))))))))))).))))))))..))))))))))))... (-85.52)

.......GGGATGGTGACAAGCTTGTGGCA............................................................................................................ 1

............................CAGCAACTGCACATCTTGCAA......................................................................................... 15

............................CAGCAACTGCACATCTTGCAAGAA...................................................................................... 2

.............................AGCAACTGCACATCTTGCAA......................................................................................... 3

.............................AGCAACTGCACATCTTGCAAG........................................................................................ 7

..............................GCAACTGCACATCTTGCAAG........................................................................................ 1

...................................................................................ATTTTCTTGCAAGTTGTGCAG.................................. 1

.....................................................................................TTTCTTGCAAGTTGTGCAGTT................................ 18

......................................................................................TTCTTGCAAGTTGTGCAGTT................................ 1

......................................................................................TTCTTGCAAGTTGTGCAGTTG............................... 8

.......................................................................................TCTTGCAAGTTGTGCAGTTG............................... 1

........................................................................................CTTGCAAGTTGTGCAGTTG............................... 1

..........................................................................................TGCAAGTTGTGCAGTTGTTGT........................... 33

...........................................................................................GCAAGTTGTGCAGTTGTTGT........................... 3

...........................................................................................GCAAGTTGTGCAGTTGTTGTC.......................... 12

............................................................................................CAAGTTGTGCAGTTGTTGTC.......................... 1

..............................................................................................AGTTGTGCAGTTGTTGTCTC........................ 1

..............................................................................................AGTTGTGCAGTTGTTGTCTCA....................... 3

................................................................................................TTGTGCAGTTGTTGTCTCAAG..................... 19

................................................................................................TTGTGCAGTTGTTGTCTCAAGCTT.................. 1

.................................................................................................TGTGCAGTTGTTGTCTCAAGC.................... 1

...................................................................................................TGCAGTTGTTGTCTCAAG..................... 96

...................................................................................................TGCAGTTGTTGTCTCAAGC.................... 193

...................................................................................................TGCAGTTGTTGTCTCAAGCT................... 41

...................................................................................................TGCAGTTGTTGTCTCAAGCTT.................. 6351

...................................................................................................TGCAGTTGTTGTCTCAAGCTTG................. 1

....................................................................................................GCAGTTGTTGTCTCAAGC.................... 3

....................................................................................................GCAGTTGTTGTCTCAAGCT................... 1

....................................................................................................GCAGTTGTTGTCTCAAGCTT.................. 142

......................................................................................................AGTTGTTGTCTCAAGCTT.................. 3

......................................................................................................AGTTGTTGTCTCAAGCTTGCTGCC............ 1

........................................................................................................TTGTTGTCTCAAGCTTGCTGCC............ 6

.........................................................................................................TGTTGTCTCAAGCTTGCTGC............. 1

.........................................................................................................TGTTGTCTCAAGCTTGCTGCC............ 366

..........................................................................................................GTTGTCTCAAGCTTGCTGCC............ 5

...........................................................................................................TTGTCTCAAGCTTGCTGCC............ 2

...........................................................................................................TTGTCTCAAGCTTGCTGCCT........... 1

>osa-MIR1440_MI0007028_Oryza_sativa_miR1440_stem-loop GSM361264

AAAUGCCAAUGCUCAAAUACCACUCUCCUAAAUUUCCAUUCCCAAAUACCACCCGGGCCCACAUGUCAGCCUCAUCCAGCACAGGGUCCCACAUCACUUUUGUUUGAAUGGAAAGUGAGGCUGACAUGUGGGCCCGGAUGGUAUUUGGUAAUGAAAAUUUGGGAGAGUGGUAUUUGAGCACUGGCAUUU

((((((((.((((((((((((((((((((((((((.((((.((((((((((.(((((((((((((((((((((((((((((.(((((........)))))))).....))))...)))))))))))))))))))))).)))))))))).)))).)))))))))))))))))))))))))).)))))))) (-130.00)

..........GCTCAAATACCACTCTCCTAAATT........................................................................................................................................................... 1

...........CTCAAATACCACTCTCCTAAA............................................................................................................................................................. 4

............TCAAATACCACTCTCCTAAATTT.......................................................................................................................................................... 1

.....................................ATTCCCAAATACCACCCGGGC................................................................................................................................... 1

.....................................ATTCCCAAATACCACCCGGGCCCA................................................................................................................................ 1

......................................TTCCCAAATACCACCCGGGCCCAC............................................................................................................................... 2

.......................................TCCCAAATACCACCCGGGCCCA................................................................................................................................ 1

............................................AATACCACCCGGGCCCACATGT........................................................................................................................... 1

.............................................ATACCACCCGGGCCCACATGTCAG........................................................................................................................ 2

.............................................ATACCACCCGGGCCCACATGTCA......................................................................................................................... 1

..............................................TACCACCCGGGCCCACATGTCAG........................................................................................................................ 2

..............................................TACCACCCGGGCCCACATGTCAGC....................................................................................................................... 2

..............................................TACCACCCGGGCCCACATGTCA......................................................................................................................... 1

...............................................ACCACCCGGGCCCACATGTCAGCC...................................................................................................................... 5

................................................CCACCCGGGCCCACATGTCAGCCT..................................................................................................................... 1

................................................CCACCCGGGCCCACATGTCAG........................................................................................................................ 1

................................................CCACCCGGGCCCACATGTCA......................................................................................................................... 1

...................................................CCCGGGCCCACATGTCAGCCTC.................................................................................................................... 1

......................................................................................................TTTGAATGGAAAGTGAGGCTGA................................................................. 1

......................................................................................................TTTGAATGGAAAGTGAGGCTGAC................................................................ 1

......................................................................................................TTTGAATGGAAAGTGAGGCTGACA............................................................... 1

.................................................................................................................AGTGAGGCTGACATGTGGGCC....................................................... 2

.........................................................................................................................................................AAAATTTGGGAGAGTGGTATTTGA............ 1

.............................................................................................................................................................TTTGGGAGAGTGGTATTTGAG........... 2

........................................................................................................................................................................GGTATTTGAGCACTGGCAT.. 1

>osa-MIR1441_MI0007029_Oryza_sativa_miR1441_stem-loop GSM361264

GUUUUUUCAUUCGUGUCCGAAAACUCCUUUUGAUAUCUGGUCAAACAUUCGAUGUGACAUCUAAAAAUUUUCUUUUCGCGAACUAAGGGCCUGUUUAGUUCGCGAAAAGAAAAUUUUUGGGUGUCACAUCGGACGUUUAACCGGAUGUCGGAAAAGGUUUUCAGACACGAAUAAAAAAAC

(((((((.(((((((((.(((((((..((((((((((((((.((((.((((((((((((((((((((((((((((((((((((((((......)))))))))))))))))))))))))))))))))))))))).)))).))))))))))))))..))))))).))))))))).))))))) (-108.40)

...TTTTCATTCGTGTCCGAAAACTCCT........................................................................................................................................................ 1

...TTTTCATTCGTGTCCGAAAACTCC......................................................................................................................................................... 1

....TTTCATTCGTGTCCGAAAACTCCT........................................................................................................................................................ 6

.....TTCATTCGTGTCCGAAAACTCCTT....................................................................................................................................................... 2

........ATTCGTGTCCGAAAACTCCTTTT..................................................................................................................................................... 1

........ATTCGTGTCCGAAAACTCCTT....................................................................................................................................................... 1

..............................................................AAAAATTTTCTTTTCGCGAACTAA.............................................................................................. 7

......................................................................................GGGCCTGTTTAGTTCGCGAAAAG....................................................................... 1

......................................................................................GGGCCTGTTTAGTTCGCGAAAAGA...................................................................... 1

.......................................................................................GGCCTGTTTAGTTCGCGA........................................................................... 1

.......................................................................................GGCCTGTTTAGTTCGCGAAAAGA...................................................................... 1

.......................................................................................GGCCTGTTTAGTTCGCGAA.......................................................................... 1

.......................................................................................GGCCTGTTTAGTTCGCGAAAAGAA..................................................................... 1

.......................................................................................GGCCTGTTTAGTTCGCGAAAA........................................................................ 1

........................................................................................GCCTGTTTAGTTCGCGAAAAGAA..................................................................... 1

........................................................................................GCCTGTTTAGTTCGCGAAAAGAAA.................................................................... 3

...........................................................................................TGTTTAGTTCGCGAAAAGAAAATT................................................................. 1

..............................................................................................TTAGTTCGCGAAAAGAAAATTTTT.............................................................. 7

..................................................................................................TTCGCGAAAAGAAAATTTTTGGG........................................................... 1

.......................................................................................................GAAAAGAAAATTTTTGGGTGTCAC..................................................... 2

........................................................................................................AAAAGAAAATTTTTGGGTGTCACA.................................................... 1

...........................................................................................................AGAAAATTTTTGGGTGTCACATC.................................................. 2

.............................................................................................................AAAATTTTTGGGTGTCACA.................................................... 1

.............................................................................................................AAAATTTTTGGGTGTCACATCGGA............................................... 3

..............................................................................................................AAATTTTTGGGTGTCACATC.................................................. 1

..............................................................................................................AAATTTTTGGGTGTCACATCGGAC.............................................. 24

...............................................................................................................AATTTTTGGGTGTCACATCGGAC.............................................. 3

...............................................................................................................AATTTTTGGGTGTCACATCGGACG............................................. 2

................................................................................................................ATTTTTGGGTGTCACATCGGA............................................... 1

................................................................................................................ATTTTTGGGTGTCACATCGGACG............................................. 2

................................................................................................................ATTTTTGGGTGTCACATCGGACGT............................................ 2

................................................................................................................ATTTTTGGGTGTCACATCGGACGTT........................................... 1

.................................................................................................................TTTTTGGGTGTCACATCGGAC.............................................. 1

.................................................................................................................TTTTTGGGTGTCACATCGGACGTT........................................... 1

..................................................................................................................TTTTGGGTGTCACATCGGACGTTT.......................................... 1

..................................................................................................................TTTTGGGTGTCACATCGGAC.............................................. 1

..................................................................................................................TTTTGGGTGTCACATCGGACGTT........................................... 3

..................................................................................................................TTTTGGGTGTCACATCGGACGTTT.......................................... 9

...................................................................................................................TTTGGGTGTCACATCGGAC.............................................. 2

...................................................................................................................TTTGGGTGTCACATCGGACGTT........................................... 2

...................................................................................................................TTTGGGTGTCACATCGGACGTTT.......................................... 3

...................................................................................................................TTTGGGTGTCACATCGGACGTTTA......................................... 1

....................................................................................................................TTGGGTGTCACATCGGACGTT........................................... 1

.....................................................................................................................TGGGTGTCACATCGGACGTT........................................... 1

..........................................................................................................................GTCACATCGGACGTTTAACCGGAT.................................. 3

...............................................................................................................................ATCGGACGTTTAACCGGATGTCGG............................. 2

................................................................................................................................TCGGACGTTTAACCGGATGTCGG............................. 1

.................................................................................................................................CGGACGTTTAACCGGATGTCGGA............................ 6

.................................................................................................................................CGGACGTTTAACCGGATGTCGGAA........................... 6

..................................................................................................................................GGACGTTTAACCGGATGTCGGAA........................... 1

...................................................................................................................................GACGTTTAACCGGATGTCGGAAA.......................... 1

....................................................................................................................................ACGTTTAACCGGATGTCGGA............................ 1

....................................................................................................................................ACGTTTAACCGGATGTCGGAA........................... 10

....................................................................................................................................ACGTTTAACCGGATGTCGGAAA.......................... 1

.....................................................................................................................................CGTTTAACCGGATGTCGGAA........................... 1

.................................................................................................................................................................CAGACACGAATAAAAAAA. 1

>osa-MIR1442_MI0007030_Oryza_sativa_miR1442_stem-loop GSM361264

UCUAUAUUCAUAGUACUAGAUGUGUCACAUCCAGUACUAGGUUGGUGUUUUAUGGGACGAAGGGAGUAGAUCUUUUGCUAAGUGGAGGCUAAAUUCUCAUCAUCAGAGGAGUAUACUACUCCAUCCGUCCCAUAAUAUAAGGGAUAAUGGGUGGAUGCGACACAUCCUAGUAAAAUGAAUCUGGA

((((.((((((..(((((((((((((.((((((.(.....(((..(((.((((((((((...(((((((.(((((((.......((((......)))).....))))))).....)))))))...)))))))))).)))...)))....).)))))).))))))).))))))..)))))).)))) (-75.00)

.....ATTCATAGTACTAGATGTGTCACA............................................................................................................................................................ 2

......TTCATAGTACTAGATGTGTCAC............................................................................................................................................................. 1

...................ATGTGTCACATCCAGTACTAGGTT.............................................................................................................................................. 1

....................TGTGTCACATCCAGTACTAGGTTG............................................................................................................................................. 2

...............................................TTTTATGGGACGAAGGGAG....................................................................................................................... 1

...............................................TTTTATGGGACGAAGGGAGT...................................................................................................................... 1

..................................................................................................................ACTACTCCATCCGTCCCATAATAT............................................... 3

...................................................................................................................CTACTCCATCCGTCCCATAATAT............................................... 1

....................................................................................................................TACTCCATCCGTCCCATAATAT............................................... 2

....................................................................................................................TACTCCATCCGTCCCATAATATAA............................................. 1

.....................................................................................................................ACTCCATCCGTCCCATAATAT............................................... 2

.....................................................................................................................ACTCCATCCGTCCCATAATATAAG............................................ 2

.....................................................................................................................ACTCCATCCGTCCCATAATA................................................ 2

......................................................................................................................CTCCATCCGTCCCATAATAT............................................... 1

......................................................................................................................CTCCATCCGTCCCATAATATAAGG........................................... 12

......................................................................................................................CTCCATCCGTCCCATAATATA.............................................. 7

......................................................................................................................CTCCATCCGTCCCATAATATAA............................................. 3

.......................................................................................................................TCCATCCGTCCCATAATATAAGGG.......................................... 2

.......................................................................................................................TCCATCCGTCCCATAATATAA............................................. 2

........................................................................................................................CCATCCGTCCCATAATATAA............................................. 1

.........................................................................................................................CATCCGTCCCATAATATAAGGGAT........................................ 1

.....................................................................................................................................AATATAAGGGATAATGGGTGGA.............................. 1

.....................................................................................................................................AATATAAGGGATAATGGGTGGATG............................ 1

......................................................................................................................................ATATAAGGGATAATGGGTGGATG............................ 1

......................................................................................................................................ATATAAGGGATAATGGGTGGATGC........................... 1

.........................................................................................................................................TAAGGGATAATGGGTGGATGCGAC........................ 1

..........................................................................................................................................AAGGGATAATGGGTGGATGCGACAC...................... 1

.........................................................................................................................................................GATGCGACACATCCTAGTAAAATG........ 1

............................................................................................................................................................GCGACACATCCTAGTAAAATGA....... 1

>osa-MIR1439_MI0007031_Oryza_sativa_miR1439_stem-loop GSM361264

UAUUGUUUUGUUAUAAAUGUAAUACUACCUCCGUCCCAAAAUAUAGCCAUUUUUAGCUAUGAAUCUAGACAUUGUUUUUAGCUAUGAAUCUAGACAUACAAUGUCCAAAUUCAUAACUAAAAAUGCUUAUAUUUUGGAACGGAGUGAGUAUUAAAUUUGUAGUUUUGUGAUA

(((..(....((((((((.(((((((..((((((.(((((((((((.(((((((((.(((((((...((((((((.(((((........)))))...))))))))...))))))).))))))))).))))))))))).))))))..))))))).))))))))....)..))) (-63.70)

.....................ATACTACCTCCGTCCCAAAAT.................................................................................................................................. 1

.....................ATACTACCTCCGTCCCAAAAT.................................................................................................................................. 2

......................TACTACCTCCGTCCCAAAATA................................................................................................................................. 3

......................TACTACCTCCGTCCCAAAA................................................................................................................................... 1

........................CTACCTCCGTCCCAAAATAT................................................................................................................................ 1

........................CTACCTCCGTCCCAAAATATAGC............................................................................................................................. 1

.........................TACCTCCGTCCCAAAATATA............................................................................................................................... 1

..........................ACCTCCGTCCCAAAATATAGC............................................................................................................................. 1

............................CTCCGTCCCAAAATATAGC............................................................................................................................. 2

.............................TCCGTCCCAAAATATAGC............................................................................................................................. 1

.................................................TTTTTAGCTATGAATCTAGACAT.................................................................................................... 1

..........................................................................TTTTTAGCTATGAATCTAGACAT........................................................................... 1

.....................................................................................................TGTCCAAATTCATAACTAAAAAT................................................ 4

......................................................................................................GTCCAAATTCATAACTAAAAAT................................................ 2

......................................................................................................GTCCAAATTCATAACTAAAAATGC.............................................. 1

......................................................................................................................AAAAATGCTTATATTTTGGAACGG.............................. 2

.......................................................................................................................AAAATGCTTATATTTTGGAACGGA............................. 2

........................................................................................................................AAATGCTTATATTTTGGAACGGA............................. 1

........................................................................................................................AAATGCTTATATTTTGGAACGGAG............................ 2

>osa-MIR1851_MI0008228_Oryza_sativa_miR1851_stem-loop GSM361264

CAGAGUGUCUUCGCCAAAAUGCCAUCCCGAACAGAAAUGCCACUUCCUCUUCUUCCUCCAUCCUGCGCGUGAAGAGCUCGCCUGCGGCCAUGGCUAGCUCCCUCCCUGCCAUCGCCGCCGCACGGGCAGCGGAGCCCGCGGCGCAGUGGCGACUCUUCGGUUGACGGCGGCUGGCUACGCGGUGGCAACGAGGAGGCGGCGGCGGCCAGGAGCUGCUCCACCGGCCCCACGCGCACGCGCGCCGGCGGGAUCCGCGCGUCGUCGCCGGAGUCCUUCCGCGACGACCUCAGCUGCACAGGCGUUGGCGGGGAUGGCAGGGAGGGCAGCGCCCCGAUCGCCGUCGGCAAGGCGGCAUCGGCGCGGCGGUGGAGAUGGCCAUGGCCGCCGGCGAGCUCUUCACGCGCGGGACGGAGGAAGAAGAAGAGCCAGGGGCAUUUCCGUCUGGGAUGGCAUUUUGGCGAAGACACCUGG

(((.(((((((((((((((((((((((((.((.((((((((.((((.((((((((((((.((((((((((((((((((((((.(((((((((((((...((((((((((((((.((((((.(((.((((((((.((((((((((.((((((.....(((((.((((((..((((((.(((.((..(......)..)).)))..))))))..)))).)).))))).....))).)))..))))).))))).)))....((((((((.((((...))))))))))))....))))).....))))))))).))))))))))))))((.(((((((((.((((((.....)))))))))))...)))).))....))))))))))))).)))))))))))))))))))))).)))))))))))).))...)).)))))))).)).)))))))))))))))))))))))))))). (-340.50)

..GAGTGTCTTCGCCAAAATGCCATC............................................................................................................................................................................................................................................................................................................................................................................................................................................................. 2

...............................................TCTTCTTCCTCCATCCTGCG.................................................................................................................................................................................................................................................................................................................................................................................................................... 1

................................................CTTCTTCCTCCATCCTGCGCG.................................................................................................................................................................................................................................................................................................................................................................................................................. 1

..................................................TCTTCCTCCATCCTGCGCGT................................................................................................................................................................................................................................................................................................................................................................................................................. 1

...................................................CTTCCTCCATCCTGCGCGT................................................................................................................................................................................................................................................................................................................................................................................................................. 1

.....................................................TCCTCCATCCTGCGCGTGAA.............................................................................................................................................................................................................................................................................................................................................................................................................. 8

......................................................CCTCCATCCTGCGCGTGAAGAGCT......................................................................................................................................................................................................................................................................................................................................................................................................... 1

......................................................CCTCCATCCTGCGCGTGAAGA............................................................................................................................................................................................................................................................................................................................................................................................................ 1

.......................................................CTCCATCCTGCGCGTGAAGA............................................................................................................................................................................................................................................................................................................................................................................................................ 2

...................................................................................................................................................................................................................................................................................................................................................................................ATGGCCATGGCCGCCGGCGAG............................................................................... 1

....................................................................................................................................................................................................................................................................................................................................................................................TGGCCATGGCCGCCGGCGAGCT............................................................................. 1

.....................................................................................................................................................................................................................................................................................................................................................................................GGCCATGGCCGCCGGCGAGC.............................................................................. 1

.......................................................................................................................................................................................................................................................................................................................................................................................CCATGGCCGCCGGCGAGCTCT........................................................................... 15

.......................................................................................................................................................................................................................................................................................................................................................................................CCATGGCCGCCGGCGAGCTC............................................................................ 2

.......................................................................................................................................................................................................................................................................................................................................................................................CCATGGCCGCCGGCGAGCTCTTCA........................................................................ 1

........................................................................................................................................................................................................................................................................................................................................................................................CATGGCCGCCGGCGAGCTCTT.......................................................................... 5

.........................................................................................................................................................................................................................................................................................................................................................................................ATGGCCGCCGGCGAGCTCTTCACG...................................................................... 10

.........................................................................................................................................................................................................................................................................................................................................................................................ATGGCCGCCGGCGAGCTCTTCA........................................................................ 1

..........................................................................................................................................................................................................................................................................................................................................................................................TGGCCGCCGGCGAGCTCTT.......................................................................... 1

..........................................................................................................................................................................................................................................................................................................................................................................................TGGCCGCCGGCGAGCTCTTC......................................................................... 2

..........................................................................................................................................................................................................................................................................................................................................................................................TGGCCGCCGGCGAGCTCTTCACGC..................................................................... 8

..........................................................................................................................................................................................................................................................................................................................................................................................TGGCCGCCGGCGAGCTCTTCA........................................................................ 19

...........................................................................................................................................................................................................................................................................................................................................................................................GGCCGCCGGCGAGCTCTT.......................................................................... 1

...........................................................................................................................................................................................................................................................................................................................................................................................GGCCGCCGGCGAGCTCTTCACGCG.................................................................... 3

...........................................................................................................................................................................................................................................................................................................................................................................................GGCCGCCGGCGAGCTCTTCACG...................................................................... 3

...........................................................................................................................................................................................................................................................................................................................................................................................GGCCGCCGGCGAGCTCTTCACGC..................................................................... 1

...........................................................................................................................................................................................................................................................................................................................................................................................GGCCGCCGGCGAGCTCTTCAC....................................................................... 7

...........................................................................................................................................................................................................................................................................................................................................................................................GGCCGCCGGCGAGCTCTTCA........................................................................ 2

............................................................................................................................................................................................................................................................................................................................................................................................GCCGCCGGCGAGCTCTTCACG...................................................................... 197

............................................................................................................................................................................................................................................................................................................................................................................................GCCGCCGGCGAGCTCTTCACGC..................................................................... 1

............................................................................................................................................................................................................................................................................................................................................................................................GCCGCCGGCGAGCTCTTCAC....................................................................... 9

............................................................................................................................................................................................................................................................................................................................................................................................GCCGCCGGCGAGCTCTTCA........................................................................ 2

.............................................................................................................................................................................................................................................................................................................................................................................................CCGCCGGCGAGCTCTTCACGC..................................................................... 1

..............................................................................................................................................................................................................................................................................................................................................................................................CGCCGGCGAGCTCTTCACGCG.................................................................... 13

..............................................................................................................................................................................................................................................................................................................................................................................................CGCCGGCGAGCTCTTCACGC..................................................................... 35

..............................................................................................................................................................................................................................................................................................................................................................................................CGCCGGCGAGCTCTTCAC....................................................................... 1

...............................................................................................................................................................................................................................................................................................................................................................................................GCCGGCGAGCTCTTCACGCGCG.................................................................. 2

...............................................................................................................................................................................................................................................................................................................................................................................................GCCGGCGAGCTCTTCACGCG.................................................................... 8

...............................................................................................................................................................................................................................................................................................................................................................................................GCCGGCGAGCTCTTCACG...................................................................... 7

...............................................................................................................................................................................................................................................................................................................................................................................................GCCGGCGAGCTCTTCACGC..................................................................... 5

................................................................................................................................................................................................................................................................................................................................................................................................CCGGCGAGCTCTTCACGCGCG.................................................................. 3

................................................................................................................................................................................................................................................................................................................................................................................................CCGGCGAGCTCTTCACGCGC................................................................... 1

................................................................................................................................................................................................................................................................................................................................................................................................CCGGCGAGCTCTTCACGC..................................................................... 2

.................................................................................................................................................................................................................................................................................................................................................................................................CGGCGAGCTCTTCACGCGCG.................................................................. 1

....................................................................................................................................................................................................................................................................................................................................................................................................CGAGCTCTTCACGCGCGGG................................................................ 1

...........................................................................................................................................................................................................................................................................................................................................................................................................................AGGAAGAAGAAGAGCCAGGGGCA..................................... 1

............................................................................................................................................................................................................................................................................................................................................................................................................................GGAAGAAGAAGAGCCAGGGG....................................... 1

....................................................................................................................................................................................................................................................................................................................................................................................................................................AAGAGCCAGGGGCATTTCCGTC............................. 1

......................................................................................................................................................................................................................................................................................................................................................................................................................................GAGCCAGGGGCATTTCCGTC............................. 1

>osa-MIR1884a_MI0008246_Oryza_sativa_miR1884a_stem-loop GSM361264

UCAAAAUGUGUGACGCCGUUGACUUUUCAUACAACGUUUGACCAUUCGUCUUAUUCAAAAAUUUAACAUAAAUAUGUAAAAGUAUAAGUUAAGAUUACAUUUUAUUUGAUGAUAAAACAAGUUACAAUAAAAUAAAUGAUAUUUAUAUAACUUUUUUGAAUAAGAUGAAUGGUCAAACGUUAUGCAAAAAGUCAACGGUGUCAUACAUUUUGAA

(((((((((((((((((((((((((((..((.(((((((((((((((((((((((((((((....((((....))))....((((((((.........((((((((((((..........)))).)))))))).......))))))))....))))))))))))))))))))))))))))).))..))))))))))))))))))))))))))). (-91.49)

....AATGTGTGACGCCGTTGACTTTT........................................................................................................................................................................................... 1

.....ATGTGTGACGCCGTTGACTTTTCA......................................................................................................................................................................................... 2

........TGTGACGCCGTTGACTTTTC.......................................................................................................................................................................................... 1

........TGTGACGCCGTTGACTTTTCA......................................................................................................................................................................................... 5

........TGTGACGCCGTTGACTTTTCAT........................................................................................................................................................................................ 16

........TGTGACGCCGTTGACTTTTCATA....................................................................................................................................................................................... 2

..........TGACGCCGTTGACTTTTC.......................................................................................................................................................................................... 1

..............................ACAACGTTTGACCATTCGTCTTAT................................................................................................................................................................ 1

................................AACGTTTGACCATTCGTCT................................................................................................................................................................... 1

................................AACGTTTGACCATTCGTCTTAT................................................................................................................................................................ 1

................................AACGTTTGACCATTCGTCTTATT............................................................................................................................................................... 1

................................AACGTTTGACCATTCGTCTTATTC.............................................................................................................................................................. 1

.................................ACGTTTGACCATTCGTCTTAT................................................................................................................................................................ 1

.................................ACGTTTGACCATTCGTCTTATTCA............................................................................................................................................................. 1

..................................CGTTTGACCATTCGTCTTATTCA............................................................................................................................................................. 2

...................................GTTTGACCATTCGTCTTATTCAAA........................................................................................................................................................... 1

...................................GTTTGACCATTCGTCTTATTCAAA........................................................................................................................................................... 4

....................................TTTGACCATTCGTCTTATT............................................................................................................................................................... 1

....................................TTTGACCATTCGTCTTAT................................................................................................................................................................ 1

....................................TTTGACCATTCGTCTTATTCA............................................................................................................................................................. 1

....................................TTTGACCATTCGTCTTATTCAA............................................................................................................................................................ 2

....................................TTTGACCATTCGTCTTATTCAAA........................................................................................................................................................... 1

....................................TTTGACCATTCGTCTTATTCAAAA.......................................................................................................................................................... 2

.....................................TTGACCATTCGTCTTATTCAA............................................................................................................................................................ 10

.....................................TTGACCATTCGTCTTATTCAA............................................................................................................................................................ 2

.....................................TTGACCATTCGTCTTATTCAAAAA......................................................................................................................................................... 1

.....................................TTGACCATTCGTCTTATTCAAA........................................................................................................................................................... 4

.....................................TTGACCATTCGTCTTATTCAAAA.......................................................................................................................................................... 2

.....................................TTGACCATTCGTCTTATTCAAAAA......................................................................................................................................................... 5

......................................TGACCATTCGTCTTATTCAAA........................................................................................................................................................... 1

......................................TGACCATTCGTCTTATTCAAAA.......................................................................................................................................................... 1

......................................TGACCATTCGTCTTATTCAA............................................................................................................................................................ 1

......................................TGACCATTCGTCTTATTCAAA........................................................................................................................................................... 48

.......................................GACCATTCGTCTTATTCAAAAATTT...................................................................................................................................................... 1

.......................................GACCATTCGTCTTATTCAAA........................................................................................................................................................... 1

........................................ACCATTCGTCTTATTCAAAAATTT...................................................................................................................................................... 9

........................................ACCATTCGTCTTATTCAAAAATT....................................................................................................................................................... 1

........................................ACCATTCGTCTTATTCAAAAA......................................................................................................................................................... 1

........................................ACCATTCGTCTTATTCAA............................................................................................................................................................ 1

........................................ACCATTCGTCTTATTCAAAA.......................................................................................................................................................... 1

........................................ACCATTCGTCTTATTCAAAAA......................................................................................................................................................... 13

.........................................CCATTCGTCTTATTCAAAAATTT...................................................................................................................................................... 9

.........................................CCATTCGTCTTATTCAAAAA......................................................................................................................................................... 2

.........................................CCATTCGTCTTATTCAAAA.......................................................................................................................................................... 1

.........................................CCATTCGTCTTATTCAAAAA......................................................................................................................................................... 3

..........................................CATTCGTCTTATTCAAAAATTT...................................................................................................................................................... 1

..........................................CATTCGTCTTATTCAAAAATT....................................................................................................................................................... 1

..........................................CATTCGTCTTATTCAAAAA......................................................................................................................................................... 2

...........................................ATTCGTCTTATTCAAAAA......................................................................................................................................................... 1

............................................TTCGTCTTATTCAAAAATTT...................................................................................................................................................... 1

.......................................................................................................................................................TTTTTTGAATAAGATGAATGGT......................................... 1

.........................................................................................................................................................TTTTGAATAAGATGAATGGT......................................... 1

..........................................................................................................................................................TTTGAATAAGATGAATGGT......................................... 1

..........................................................................................................................................................TTTGAATAAGATGAATGGTCA....................................... 1

..........................................................................................................................................................TTTGAATAAGATGAATGGTCAAAC.................................... 2

............................................................................................................................................................TGAATAAGATGAATGGTCAAACG................................... 1

..............................................................................................................................................................AATAAGATGAATGGTCAAACG................................... 1

...............................................................................................................................................................ATAAGATGAATGGTCAAA..................................... 1

...............................................................................................................................................................ATAAGATGAATGGTCAAACGT.................................. 1

...............................................................................................................................................................ATAAGATGAATGGTCAAACGTTAT............................... 1

......................................................................................................................................................................GAATGGTCAAACGTTATGCAAAA......................... 1

.......................................................................................................................................................................................GCAAAAAGTCAACGGTGTCATACA....... 5

........................................................................................................................................................................................CAAAAAGTCAACGGTGTCATACATT..... 1

........................................................................................................................................................................................CAAAAAGTCAACGGTGTCATACAT...... 1

.........................................................................................................................................................................................AAAAAGTCAACGGTGTCATACATT..... 1

.........................................................................................................................................................................................AAAAAGTCAACGGTGTCATACATT..... 4

.........................................................................................................................................................................................AAAAAGTCAACGGTGTCATA......... 4

..........................................................................................................................................................................................AAAAGTCAACGGTGTCATA......... 1

..........................................................................................................................................................................................AAAAGTCAACGGTGTCATACATT..... 1

..........................................................................................................................................................................................AAAAGTCAACGGTGTCATACATT..... 1

..........................................................................................................................................................................................AAAAGTCAACGGTGTCATA......... 2

...........................................................................................................................................................................................AAAGTCAACGGTGTCATA......... 1

...........................................................................................................................................................................................AAAGTCAACGGTGTCATACATTTT... 1

...........................................................................................................................................................................................AAAGTCAACGGTGTCATACATT..... 1

...........................................................................................................................................................................................AAAGTCAACGGTGTCATACAT...... 1

.............................................................................................................................................................................................AGTCAACGGTGTCATACATT..... 1

...............................................................................................................................................................................................TCAACGGTGTCATACATTTTG.. 1

>osa-MIR1875_MI0008278_Oryza_sativa_miR1875_stem-loop GSM361264

GCUGUCCAUUUUCAUUGCACUUUGCUCCAUUGUGGUAUUGACAUUCUGCAAGAACGAGUCCCAGUAUCAAGUCACUGUUACGGUGUUGUUGGUACCAUUGAUUAACAUAACACUUGACGGCCAUACUUGGUCAUGUUGUAUGUUAUCAGCUUCACAGAGGUACAUGUGGCACUUUAACAGUUAUUUGAUACAGUGACUAGGCUGCAGUUGAGCGAAACCACAAUGGAGUGAAGUGCAACAGAAAUGAACAUU

..(((.((((((..((((((((..((((((((((((.(((.....(((((.(....((((.(.(((((((((.(((((((.(((((..(..(((((..(((.....((((((..(((((((((....)))))..))))..)))))).....)))....)))))..)..))))).))))))).))))))))).).))))...)))))).....))).))))))))))))..))))))))..)))))).))).. (-108.10)

.............ATTGCACTTTGCTCCATTGTG.......................................................................................................................................................................................................................... 1

......................................TGACATTCTGCAAGAACGAGTCCC.............................................................................................................................................................................................. 1

......................................................................................................................................................................................ATTTGATACAGTGACTAGGC.................................................. 2

.......................................................................................................................................................................................TTTGATACAGTGACTAGGCT................................................. 1

...............................................................................................................................................................................................AGTGACTAGGCTGCAGTTGAG........................................ 1

..........................................................................................................................................................................................................................CACAATGGAGTGAAGTGCAACA............ 1

...........................................................................................................................................................................................................................ACAATGGAGTGAAGTGCAACAG........... 6

...........................................................................................................................................................................................................................ACAATGGAGTGAAGTGCAACAGA.......... 8

...........................................................................................................................................................................................................................ACAATGGAGTGAAGTGCAACAGAA......... 14

...........................................................................................................................................................................................................................ACAATGGAGTGAAGTGCAACAGAAA........ 3

............................................................................................................................................................................................................................CAATGGAGTGAAGTGCAACAG........... 1

.............................................................................................................................................................................................................................AATGGAGTGAAGTGCAACAG........... 1

.............................................................................................................................................................................................................................AATGGAGTGAAGTGCAACAGA.......... 1

.............................................................................................................................................................................................................................AATGGAGTGAAGTGCAACAGAA......... 6

.............................................................................................................................................................................................................................AATGGAGTGAAGTGCAACAGAAA........ 7

..............................................................................................................................................................................................................................ATGGAGTGAAGTGCAACAG........... 3

..............................................................................................................................................................................................................................ATGGAGTGAAGTGCAACAGA.......... 4

..............................................................................................................................................................................................................................ATGGAGTGAAGTGCAACAGAA......... 10

..............................................................................................................................................................................................................................ATGGAGTGAAGTGCAACAGAAA........ 25

..............................................................................................................................................................................................................................ATGGAGTGAAGTGCAACAGAAAT....... 1

..............................................................................................................................................................................................................................ATGGAGTGAAGTGCAACAGAAATG...... 1

................................................................................................................................................................................................................................GGAGTGAAGTGCAACAGAAAT....... 1

....................................................................................................................................................................................................................................TGAAGTGCAACAGAAATGAAC... 1

>osa-MIR1884b_MI0008281_Oryza_sativa_miR1884b_stem-loop GSM361264

UCUGUUUUUAAAUGUAUGACGCUGUUGACUUUUAGAUAUAUGUUGACAAUUCGUCUUAUUCAAAAAAUCGAGCAAAUAUAAAAAUAUAAGUGAGGCUUAAGGUAUAUUUGAUGAUAAAACAAGUCAUGGCAAAAUUAAUGAUACCUGUAUAACUUUUUUGAGUAAGACGAGUGGUCAAACGUAGAUUUAAAAGUCAACGGUGUCAUAUAUUUAUAAAUGGA

(((((((.(((((((((((((((((((((((((((((.(((((((((.(((((((((((((((((((.....((((((((....(.((((.....)))).).))))))))........(((.(((((..........)))))...))).....))))))))))))))))))).))).)))))).))))))))))))))))))))))))))))).))))))) (-86.30)

.......TTAAATGTATGACGCTGTTGACTT.............................................................................................................................................................................................. 1

..........AATGTATGACGCTGTTGACT............................................................................................................................................................................................... 3

..........AATGTATGACGCTGTTGACTTTTA........................................................................................................................................................................................... 17

...........ATGTATGACGCTGTTGACT............................................................................................................................................................................................... 1

........................TTGACTTTTAGATATATGTTGA............................................................................................................................................................................... 1

.........................TGACTTTTAGATATATGTTGAC.............................................................................................................................................................................. 1

..........................................TTGACAATTCGTCTTATTCAA.............................................................................................................................................................. 1

...........................................TGACAATTCGTCTTATTCAAAA............................................................................................................................................................ 1

..............................................CAATTCGTCTTATTCAAAAAA.......................................................................................................................................................... 2

...............................................AATTCGTCTTATTCAAAA............................................................................................................................................................ 1

...............................................AATTCGTCTTATTCAAAAA........................................................................................................................................................... 1

................................................ATTCGTCTTATTCAAAAA........................................................................................................................................................... 1

.................................................TTCGTCTTATTCAAAAAA.......................................................................................................................................................... 1

.............................................................................................................................................................TTGAGTAAGACGAGTGGTCAA........................................... 3

.............................................................................................................................................................TTGAGTAAGACGAGTGGTCAAA.......................................... 2

..............................................................................................................................................................TGAGTAAGACGAGTGGTCAA........................................... 1

..............................................................................................................................................................TGAGTAAGACGAGTGGTCAAA.......................................... 1

..............................................................................................................................................................TGAGTAAGACGAGTGGTCAAACG........................................ 5

...............................................................................................................................................................GAGTAAGACGAGTGGTCAAACG........................................ 1

................................................................................................................................................................AGTAAGACGAGTGGTCAAACGTAGA.................................... 20

.................................................................................................................................................................GTAAGACGAGTGGTCAAACGTAGA.................................... 2

..................................................................................................................................................................TAAGACGAGTGGTCAAACG........................................ 1

..................................................................................................................................................................TAAGACGAGTGGTCAAACGTA...................................... 2

.......................................................................................................................................................................................GATTTAAAAGTCAACGGTGTCATA.............. 3

........................................................................................................................................................................................ATTTAAAAGTCAACGGTGTCATAT............. 1

.........................................................................................................................................................................................TTTAAAAGTCAACGGTGTCATA.............. 2

.........................................................................................................................................................................................TTTAAAAGTCAACGGTGTCATATA............ 5

..........................................................................................................................................................................................TTAAAAGTCAACGGTGTCATA.............. 3

..........................................................................................................................................................................................TTAAAAGTCAACGGTGTCATAT............. 1

...........................................................................................................................................................................................TAAAAGTCAACGGTGTCATA.............. 1

............................................................................................................................................................................................AAAAGTCAACGGTGTCATA.............. 1

............................................................................................................................................................................................AAAAGTCAACGGTGTCATATA............ 2

............................................................................................................................................................................................AAAAGTCAACGGTGTCATATAT........... 1

............................................................................................................................................................................................AAAAGTCAACGGTGTCATATATT.......... 7

............................................................................................................................................................................................AAAAGTCAACGGTGTCATATATTT......... 3

............................................................................................................................................................................................AAAAGTCAACGGTGTCATA.............. 2

.............................................................................................................................................................................................AAAGTCAACGGTGTCATA.............. 1

.............................................................................................................................................................................................AAAGTCAACGGTGTCATATAT........... 1

.............................................................................................................................................................................................AAAGTCAACGGTGTCATATATT.......... 1

.............................................................................................................................................................................................AAAGTCAACGGTGTCATATATTT......... 3

.............................................................................................................................................................................................AAAGTCAACGGTGTCATATATTTA........ 179

..............................................................................................................................................................................................AAGTCAACGGTGTCATATATTTA........ 8

...............................................................................................................................................................................................AGTCAACGGTGTCATATATTTA........ 1

>osa-MIR812h_MI0008297_Oryza_sativa_miR812h_stem-loop GSM361264

UUGUACUCCCUCCGUCCCAUUUUAAGUGCAAUCAUGAGUUUCGCCCAACUUUAAUCAUCUGUUUUAUUUGAACUUUUUAUAAAUAGUAUUUUUAUUGUUAUGAGAUUAUAAAAUUUAAAUAAGACGGAUGAUUAAAGUUGGACACGAAAACUCAUGGCUACACUUACAAUGAAAUGGAGGGGUACUA

..((((.((((((((..((((.((((((.(..(((((((((((.((((((((((((((((((((((((((((..((((((((((((((....)))))).......))))))))))))))))))))))))))))))))))))...)).)))))))))..).)))))).))))..)))))))))))).. (-83.71)

....ACTCCCTCCGTCCCATTTTAAGT................................................................................................................................................................ 1

....ACTCCCTCCGTCCCATTTTAAGTG............................................................................................................................................................... 1

....ACTCCCTCCGTCCCATTTTAA.................................................................................................................................................................. 1

.....CTCCCTCCGTCCCATTTTAAGT................................................................................................................................................................ 1

.....CTCCCTCCGTCCCATTTTAAGTGC.............................................................................................................................................................. 1

......TCCCTCCGTCCCATTTTAAGT................................................................................................................................................................ 1

......TCCCTCCGTCCCATTTTAAGTGC.............................................................................................................................................................. 1

.......CCCTCCGTCCCATTTTAAGT................................................................................................................................................................ 1

.......CCCTCCGTCCCATTTTAAGTG............................................................................................................................................................... 2

.......CCCTCCGTCCCATTTTAAGTGCA............................................................................................................................................................. 1

........CCTCCGTCCCATTTTAAGTGCAA............................................................................................................................................................ 1

.........CTCCGTCCCATTTTAAGTGCAAT........................................................................................................................................................... 1

.........CTCCGTCCCATTTTAAGTGCAATC.......................................................................................................................................................... 4

.........CTCCGTCCCATTTTAAGTGC.............................................................................................................................................................. 1

.........CTCCGTCCCATTTTAAGTGCA............................................................................................................................................................. 1

..........TCCGTCCCATTTTAAGTGCAATCA......................................................................................................................................................... 3

..........TCCGTCCCATTTTAAGTGCAA............................................................................................................................................................ 2

...........CCGTCCCATTTTAAGTGCAATCAT........................................................................................................................................................ 34

.........................................CGCCCAACTTTAATCATCTGTTTT.......................................................................................................................... 8

...........................................CCCAACTTTAATCATCTGTTT........................................................................................................................... 1

............................................CCAACTTTAATCATCTGTTTTATT....................................................................................................................... 1

...........................................................................................................ATAAAATTTAAATAAGACGGATG......................................................... 1

...........................................................................................................ATAAAATTTAAATAAGACGGATGA........................................................ 7

.............................................................................................................AAAATTTAAATAAGACGGATGA........................................................ 1

..............................................................................................................AAATTTAAATAAGACGGATGATTA..................................................... 1

...............................................................................................................AATTTAAATAAGACGGATGATTAA.................................................... 1

................................................................................................................ATTTAAATAAGACGGATGATTAA.................................................... 1

................................................................................................................ATTTAAATAAGACGGATGATTAAA................................................... 3

.................................................................................................................TTTAAATAAGACGGATGATTA..................................................... 1

..................................................................................................................TTAAATAAGACGGATGATTAAAGT................................................. 1

..................................................................................................................TTAAATAAGACGGATGATTAAA................................................... 1

....................................................................................................................AAATAAGACGGATGATTAAAG.................................................. 2

....................................................................................................................AAATAAGACGGATGATTAAAGTT................................................ 1

....................................................................................................................AAATAAGACGGATGATTAAAGTTG............................................... 1

....................................................................................................................AAATAAGACGGATGATTAAAGTTGG.............................................. 1

.....................................................................................................................AATAAGACGGATGATTAAA................................................... 1

.....................................................................................................................AATAAGACGGATGATTAAAG.................................................. 1

.....................................................................................................................AATAAGACGGATGATTAAAGT................................................. 2

.....................................................................................................................AATAAGACGGATGATTAAAGTT................................................ 1

.....................................................................................................................AATAAGACGGATGATTAAAGTTGG.............................................. 48

......................................................................................................................ATAAGACGGATGATTAAAGTTGG.............................................. 2

......................................................................................................................ATAAGACGGATGATTAAAGTTGGA............................................. 15

.......................................................................................................................TAAGACGGATGATTAAAGTT................................................ 1

.......................................................................................................................TAAGACGGATGATTAAAGTTG............................................... 3

.......................................................................................................................TAAGACGGATGATTAAAGTTGG.............................................. 1

.......................................................................................................................TAAGACGGATGATTAAAGTTGGA............................................. 2

.......................................................................................................................TAAGACGGATGATTAAAGTTGGAC............................................ 6

........................................................................................................................AAGACGGATGATTAAAGTTGGACA........................................... 39

.........................................................................................................................AGACGGATGATTAAAGTTGGACA........................................... 1

..........................................................................................................................GACGGATGATTAAAGTTGGACACG......................................... 4

...........................................................................................................................ACGGATGATTAAAGTTGGACACG......................................... 1

...............................................................................................................................ATGATTAAAGTTGGACACG......................................... 1

...............................................................................................................................ATGATTAAAGTTGGACACGAA....................................... 1

...............................................................................................................................ATGATTAAAGTTGGACACGAAAAC.................................... 25

................................................................................................................................TGATTAAAGTTGGACACGAAA...................................... 1

................................................................................................................................TGATTAAAGTTGGACACGAAAA..................................... 1

................................................................................................................................TGATTAAAGTTGGACACGAAAAC.................................... 1

.................................................................................................................................GATTAAAGTTGGACACGAA....................................... 1

...................................................................................................................................TTAAAGTTGGACACGAAAAC.................................... 2

>osa-MIR1883b_MI0008301_Oryza_sativa_miR1883b_stem-loop GSM361264

CGGUUGCAGCCCGUCACAGGUAUCUAGUCACCUGUGACGGGCCGAGAAUGGAAUCCGUCAAAGGAACACACCUUUGUCAGAUUCUACUUCCUGCCCAUCACAGGUGACUAGAUACCUGUGACGGGCCGCAACUUAC

.((((((.(((((((((((((((((((((((((((((.((((.(.((((((((((.(.((((((......)))))).).))))))).)))).)))).))))))))))))))))))))))))))))).))))))... (-98.40)

........GCCCGTCACAGGTATCTAGTCACC........................................................................................................ 3

.........CCCGTCACAGGTATCTAGTCACCT....................................................................................................... 2

...............................CTGTGACGGGCCGAGAATGGA.................................................................................... 1

.....................................................................................................AGGTGACTAGATACCTGTGACGGG........... 2

......................................................................................................GGTGACTAGATACCTGTGACGGGC.......... 3

>osa-MIR1318_MI0009715_Oryza_sativa_miR1318_stem-loop GSM361264

GGAUGUGCGUUCUUGUGGGGGGUUUGGGCCCUGUGAUCAGGAGAGAUGACACCGACAUCGCCGGAAUUCGUUCUUGGUCUUGUGCCAUGAUGAAUUGAUGGUCCGUUUGAUGCAGGUGUCAUCUCCCCUGAACAUAGGACUGACUGACCCUGAAUGGAU

....((.(((((....(((.((((..((.((((((.(((((.(((((((((((..(((((.(((((((((((..((((.....)))).)))))))......))))..)))))..))))))))))).))))).)))))).))))))..))).))))).)) (-70.60)

..............................CTGTGATCAGGAGAGATGACA............................................................................................................ 2

...............................TGTGATCAGGAGAGATGACAC........................................................................................................... 3

..................................GATCAGGAGAGATGACACCGA........................................................................................................ 32

..................................GATCAGGAGAGATGACACCGAC....................................................................................................... 6

..................................GATCAGGAGAGATGACACCGACA...................................................................................................... 33

...................................ATCAGGAGAGATGACACC.......................................................................................................... 16

...................................ATCAGGAGAGATGACACCG......................................................................................................... 14

...................................ATCAGGAGAGATGACACCGA........................................................................................................ 99

...................................ATCAGGAGAGATGACACCGAC....................................................................................................... 9315

...................................ATCAGGAGAGATGACACCGACA...................................................................................................... 39380

...................................ATCAGGAGAGATGACACCGACAT..................................................................................................... 201

...................................ATCAGGAGAGATGACACCGACATC.................................................................................................... 16

....................................TCAGGAGAGATGACACCG......................................................................................................... 9

....................................TCAGGAGAGATGACACCGA........................................................................................................ 236

....................................TCAGGAGAGATGACACCGAC....................................................................................................... 7005

....................................TCAGGAGAGATGACACCGACA...................................................................................................... 3681

....................................TCAGGAGAGATGACACCGACAT..................................................................................................... 13

....................................TCAGGAGAGATGACACCGACATCG................................................................................................... 1

.....................................CAGGAGAGATGACACCGA........................................................................................................ 2

.....................................CAGGAGAGATGACACCGAC....................................................................................................... 102

.....................................CAGGAGAGATGACACCGACA...................................................................................................... 56

.....................................CAGGAGAGATGACACCGACAT..................................................................................................... 1

......................................AGGAGAGATGACACCGAC....................................................................................................... 206

......................................AGGAGAGATGACACCGACA...................................................................................................... 274

......................................AGGAGAGATGACACCGACAT..................................................................................................... 1

..........................................GAGATGACACCGACATCGCCG................................................................................................ 4

..........................................GAGATGACACCGACATCGCCGGAA............................................................................................. 1

...........................................AGATGACACCGACATCGCCGG............................................................................................... 1

............................................GATGACACCGACATCGCCGG............................................................................................... 1

...........................................................................................GAATTGATGGTCCGTTTGATG............................................... 1

..........................................................................................................TTGATGCAGGTGTCATCTCCC................................ 1

...............................................................................................................GCAGGTGTCATCTCCCCTGAAC.......................... 1

................................................................................................................CAGGTGTCATCTCCCCTGAA........................... 4

................................................................................................................CAGGTGTCATCTCCCCTGAAC.......................... 66

................................................................................................................CAGGTGTCATCTCCCCTGAACA......................... 16

.................................................................................................................AGGTGTCATCTCCCCTGAAC.......................... 3

.................................................................................................................AGGTGTCATCTCCCCTGAACA......................... 1

..................................................................................................................GGTGTCATCTCCCCTGAAC.......................... 1

..................................................................................................................GGTGTCATCTCCCCTGAACA......................... 2

..................................................................................................................GGTGTCATCTCCCCTGAACAT........................ 4

...............................................................................................................................CTGAACATAGGACTGACTG............. 8

...............................................................................................................................CTGAACATAGGACTGACTGA............ 2

>osa-MIR1319_MI0009716_Oryza_sativa_miR1319_stem-loop GSM361264

UAUGGGUGUCGGUUUUUAAGAAAAAACCGGCAUCUGUAAUAUAUUAUAGGUGUCGGUUAAUUAAAAACCGAGACCUAUAAUAUAUUAUAAGUGCCGGUUUUUUUAAAACCGACACCUAUCU

.(((((((((((((((...(((((((((((((..(((((((((((((((((.((((((.......)))))).)))))))))))))))))..)))))))))))))))))))))))))))).. (-73.80)

.................AAGAAAAAACCGGCATCTGTAATA................................................................................ 1

....................AAAAAACCGGCATCTGTAAT................................................................................. 1

.................................CTGTAATATATTATAGGTGTCGGT................................................................ 1

....................................TAATATATTATAGGTGTCGGTTAA............................................................. 2

.....................................AATATATTATAGGTGTCGGTT............................................................... 1

.....................................AATATATTATAGGTGTCGGTTAAT............................................................ 1

........................................ATATTATAGGTGTCGGTTAAT............................................................ 1

........................................ATATTATAGGTGTCGGTTAATTAA......................................................... 3

..........................................ATTATAGGTGTCGGTTAATTAAAA....................................................... 3

...........................................TTATAGGTGTCGGTTAATTAA......................................................... 1

...........................................................ATTAAAAACCGAGACCTATAATAT...................................... 1

.................................................................AACCGAGACCTATAATATATTAT................................. 1

..................................................................ACCGAGACCTATAATATATTAT................................. 1

......................................................................................ATAAGTGCCGGTTTTTTTAAAACC........... 1

.......................................................................................TAAGTGCCGGTTTTTTTAA............... 1

................................................................................................GTTTTTTTAAAACCGACACCTATC. 1

.................................................................................................TTTTTTTAAAACCGACACCTATCT 1

...................................................................................................TTTTTAAAACCGACACCTAT.. 1

>osa-MIR396f_MI0010563_Oryza_sativa_miR396f_stem-loop GSM361264

GCCAUGCUCUCCACAGGCUUUCUUGAACUGUGAACUCGUGUGUGCAUGCUCCUCAUAUAUUGUUCUAGAUCCCAUGCAUGAUGCAUAUCGAUCGAUCUGAUCUGAAUUAGGUCAUCGAUGCGCAUCUGGAUCCCCAUCUUGUUGAUAGUUCAAGAAAGUCCUUGGAAAACAUGGUG

((((((...((((..((((((((((((((((.(((..(((((((.........))))))).......(((((.......(((((.(((((((.((((((((....))))))))))))))).))))).)))))........))).))))))))))))))))..))))...)))))). (-70.20)

........CTCCACAGGCTTTCTTGAACTG.................................................................................................................................................. 9

.........TCCACAGGCTTTCTTGAA..................................................................................................................................................... 66

.........TCCACAGGCTTTCTTGAAC.................................................................................................................................................... 250

.........TCCACAGGCTTTCTTGAACT................................................................................................................................................... 13

.........TCCACAGGCTTTCTTGAACTG.................................................................................................................................................. 2629

.........TCCACAGGCTTTCTTGAACTGT................................................................................................................................................. 3

..........CCACAGGCTTTCTTGAACTG.................................................................................................................................................. 13

..........CCACAGGCTTTCTTGAACTGT................................................................................................................................................. 4

...........CACAGGCTTTCTTGAACT................................................................................................................................................... 5

...........CACAGGCTTTCTTGAACTG.................................................................................................................................................. 423

...........CACAGGCTTTCTTGAACTGT................................................................................................................................................. 2

...........CACAGGCTTTCTTGAACTGTG................................................................................................................................................ 1

...........CACAGGCTTTCTTGAACTGTGA............................................................................................................................................... 1

............ACAGGCTTTCTTGAACTG.................................................................................................................................................. 1

............ACAGGCTTTCTTGAACTGTG................................................................................................................................................ 1

............ACAGGCTTTCTTGAACTGTGA............................................................................................................................................... 331

............ACAGGCTTTCTTGAACTGTGAA.............................................................................................................................................. 1

..........................................................................................................TTAGGTCATCGATGCGCATC.................................................. 1

................................................................................................................................................ATAGTTCAAGAAAGTCCTTG............ 2

................................................................................................................................................ATAGTTCAAGAAAGTCCTTGG........... 7

................................................................................................................................................ATAGTTCAAGAAAGTCCTTGGAAA........ 13

....................................................................................................................................................TTCAAGAAAGTCCTTGGAAA........ 5

.....................................................................................................................................................TCAAGAAAGTCCTTGGAAA........ 1

>osa-MIR2122_MI0010711_Oryza_sativa_miR2122_stem-loop GSM361264

UUUCAAAAAUAACCUUUUGUUCAGCGCAAAAUCGUAUAGCGCUGAACUUAGACACCUCAGCGCCACGUCAACUGGCGCUGAAUGUCGUGCCACCGUGAAUAGGAGGCUGAGUCAGCGUGCCAAUGUACAUUCAGCGUCGUGCUAUUUAGCGCUGAGGUGUCUAAGUUUAGUGCCAUAUGAUUUGGCGCUGAACAAAAUGGUAAUUUUUUAAA

....((((((.((((((((((((((((.(((((((((.(((((((((((((((((((((((((.......((.((((((((((((((.(((.((.......)).))))))....((((....)))).)))))))))))))........))))))))))))))))))))))))).))))))))).))))))))))))).))).)))))).... (-113.56)

.............CTTTTGTTCAGCGCAAAATCGTAT............................................................................................................................................................................... 1

.................................GTATAGCGCTGAACTTAGACACCT........................................................................................................................................................... 1

......................................GCGCTGAACTTAGACACCTCAGC....................................................................................................................................................... 1

.........................................CTGAACTTAGACACCTCAGCG...................................................................................................................................................... 1

..........................................TGAACTTAGACACCTCAGCGCCAC.................................................................................................................................................. 2

............................................AACTTAGACACCTCAGCGCCAC.................................................................................................................................................. 1

..............................................CTTAGACACCTCAGCGCCACGTCA.............................................................................................................................................. 1

...............................................TTAGACACCTCAGCGCCACGTCAA............................................................................................................................................. 1

.................................................AGACACCTCAGCGCCACGTCAACT........................................................................................................................................... 4

..................................................GACACCTCAGCGCCACGTCAACT........................................................................................................................................... 1

..................................................GACACCTCAGCGCCACGTCAACTG.......................................................................................................................................... 3

...................................................ACACCTCAGCGCCACGTCAACTGG......................................................................................................................................... 1

....................................................CACCTCAGCGCCACGTCAACT........................................................................................................................................... 1

....................................................CACCTCAGCGCCACGTCAACTGGC........................................................................................................................................ 5

..............................................................................................................................................................................ATATGATTTGGCGCTGAACAAA................ 1

..............................................................................................................................................................................ATATGATTTGGCGCTGAACAAAA............... 1

................................................................................................................................................................................ATGATTTGGCGCTGAACAAAATGG............ 1

.....................................................................................................................................................................................TTGGCGCTGAACAAAATGGTAATT....... 1

>osa-MIR2123a_MI0010712_Oryza_sativa_miR2123a_stem-loop GSM361264

UUUGACACCGUUGACUUUUUAGGACGUGUUUGACCAUUCGUCUUAUUCAAAAAUUUUAAGUAAUUAUUUAUUCUUUUCAUAUCAUUUGAUUCCUUGUUAAAUGUAUUUUCAUGUACACAUCUAGUUUUACAUAUUUCACAAGACGAACGGUCAGACAUGUACUAAAAAGUCAACGGUGUCAAACA

((((((((((((((((((((((.((((((((((((.((((((((......................................((((((((.....)))))))).......(((((.((.....))..))))).......)))))))).)))))))))))).)))))))))))))))))))))).. (-71.80)

TTTGACACCGTTGACTTTTT..................................................................................................................................................................... 1

TTTGACACCGTTGACTTT....................................................................................................................................................................... 1

TTTGACACCGTTGACTTTTTAG................................................................................................................................................................... 2

TTTGACACCGTTGACTTTTTA.................................................................................................................................................................... 7

TTTGACACCGTTGACTTT....................................................................................................................................................................... 4

TTTGACACCGTTGACTTTT...................................................................................................................................................................... 12

TTTGACACCGTTGACTTTTT..................................................................................................................................................................... 23

TTTGACACCGTTGACTTTTTA.................................................................................................................................................................... 6

TTTGACACCGTTGACTTTTTAG................................................................................................................................................................... 7

.TTGACACCGTTGACTTTTTAG................................................................................................................................................................... 5

.TTGACACCGTTGACTTTT...................................................................................................................................................................... 4

.TTGACACCGTTGACTTTTT..................................................................................................................................................................... 36

.TTGACACCGTTGACTTTTTA.................................................................................................................................................................... 3

.TTGACACCGTTGACTTTTTAG................................................................................................................................................................... 6

.....CACCGTTGACTTTTTAGGACGTGT............................................................................................................................................................ 1

......ACCGTTGACTTTTTAGGACGTGTT........................................................................................................................................................... 1

.......CCGTTGACTTTTTAGGACGTGTTT.......................................................................................................................................................... 2

.........GTTGACTTTTTAGGACGTGTTTGA........................................................................................................................................................ 1

..............CTTTTTAGGACGTGTTTGACCA..................................................................................................................................................... 1

.................TTTAGGACGTGTTTGACCAT.................................................................................................................................................... 1

.......................ACGTGTTTGACCATTCGTCTTATT.......................................................................................................................................... 1

........................CGTGTTTGACCATTCGTCTTAT........................................................................................................................................... 1

........................CGTGTTTGACCATTCGTCTTATT.......................................................................................................................................... 1

.........................GTGTTTGACCATTCGTCTTAT........................................................................................................................................... 1

.........................GTGTTTGACCATTCGTCTTATTCA........................................................................................................................................ 8

..........................TGTTTGACCATTCGTCTTATT.......................................................................................................................................... 28

..........................TGTTTGACCATTCGTCTTAT........................................................................................................................................... 13

..........................TGTTTGACCATTCGTCTTA............................................................................................................................................ 1

..........................TGTTTGACCATTCGTCTTATTCA........................................................................................................................................ 9

..........................TGTTTGACCATTCGTCTTATT.......................................................................................................................................... 2

..........................TGTTTGACCATTCGTCTTATTCAA....................................................................................................................................... 8

...........................GTTTGACCATTCGTCTTATTCAAA...................................................................................................................................... 1

...........................GTTTGACCATTCGTCTTATTCAAA...................................................................................................................................... 4

............................TTTGACCATTCGTCTTATT.......................................................................................................................................... 1

............................TTTGACCATTCGTCTTAT........................................................................................................................................... 1

............................TTTGACCATTCGTCTTATTCA........................................................................................................................................ 1

............................TTTGACCATTCGTCTTATTCAA....................................................................................................................................... 2

............................TTTGACCATTCGTCTTATTCAAA...................................................................................................................................... 1

............................TTTGACCATTCGTCTTATTCAAAA..................................................................................................................................... 2

.............................TTGACCATTCGTCTTATTCAA....................................................................................................................................... 10

.............................TTGACCATTCGTCTTATTCAA....................................................................................................................................... 2

.............................TTGACCATTCGTCTTATTCAAAAA.................................................................................................................................... 1

.............................TTGACCATTCGTCTTATTCAAA...................................................................................................................................... 4

.............................TTGACCATTCGTCTTATTCAAAA..................................................................................................................................... 2

.............................TTGACCATTCGTCTTATTCAAAAA.................................................................................................................................... 5

..............................TGACCATTCGTCTTATTCAAA...................................................................................................................................... 1

..............................TGACCATTCGTCTTATTCAAAA..................................................................................................................................... 1

..............................TGACCATTCGTCTTATTCAA....................................................................................................................................... 1

..............................TGACCATTCGTCTTATTCAAA...................................................................................................................................... 48

...............................GACCATTCGTCTTATTCAAAAATTT................................................................................................................................. 1

...............................GACCATTCGTCTTATTCAAA...................................................................................................................................... 1

................................ACCATTCGTCTTATTCAAAAATTTT................................................................................................................................ 2

................................ACCATTCGTCTTATTCAAAAATTT................................................................................................................................. 9

................................ACCATTCGTCTTATTCAAAAATT.................................................................................................................................. 1

................................ACCATTCGTCTTATTCAAAAA.................................................................................................................................... 1

................................ACCATTCGTCTTATTCAA....................................................................................................................................... 1

................................ACCATTCGTCTTATTCAAAA..................................................................................................................................... 1

................................ACCATTCGTCTTATTCAAAAA.................................................................................................................................... 13

.................................CCATTCGTCTTATTCAAAAATTTT................................................................................................................................ 103

.................................CCATTCGTCTTATTCAAAAATTT................................................................................................................................. 9

.................................CCATTCGTCTTATTCAAAAA.................................................................................................................................... 2

.................................CCATTCGTCTTATTCAAAA..................................................................................................................................... 1

.................................CCATTCGTCTTATTCAAAAA.................................................................................................................................... 3

..................................CATTCGTCTTATTCAAAAATTTT................................................................................................................................ 23

..................................CATTCGTCTTATTCAAAAATTT................................................................................................................................. 1

..................................CATTCGTCTTATTCAAAAATT.................................................................................................................................. 1

..................................CATTCGTCTTATTCAAAAATTTTA............................................................................................................................... 3

..................................CATTCGTCTTATTCAAAAA.................................................................................................................................... 2

...................................ATTCGTCTTATTCAAAAATTTT................................................................................................................................ 1

...................................ATTCGTCTTATTCAAAAATTTTA............................................................................................................................... 17

...................................ATTCGTCTTATTCAAAAATTTTAA.............................................................................................................................. 1

...................................ATTCGTCTTATTCAAAAA.................................................................................................................................... 1

....................................TTCGTCTTATTCAAAAATTTT................................................................................................................................ 3

....................................TTCGTCTTATTCAAAAATTT................................................................................................................................. 1

....................................TTCGTCTTATTCAAAAATTTTA............................................................................................................................... 5

....................................TTCGTCTTATTCAAAAATTTTAA.............................................................................................................................. 1

.....................................TCGTCTTATTCAAAAATTTT................................................................................................................................ 1

.....................................TCGTCTTATTCAAAAATTTTAAGT............................................................................................................................ 9

.....................................TCGTCTTATTCAAAAATTTTAAG............................................................................................................................. 1

.....................................TCGTCTTATTCAAAAATTTTA............................................................................................................................... 9

......................................CGTCTTATTCAAAAATTTTAAGTA........................................................................................................................... 1

........................................TCTTATTCAAAAATTTTAAGTA........................................................................................................................... 1

...........................................TATTCAAAAATTTTAAGTAATTAT...................................................................................................................... 1

........................................................................................................................................................AGACATGTACTAAAAAGTCAACGG......... 2

..........................................................................................................................................................ACATGTACTAAAAAGTCAACGGTG....... 1

.............................................................................................................................................................TGTACTAAAAAGTCAACG.......... 1

.............................................................................................................................................................TGTACTAAAAAGTCAACGGTGTC..... 1

..............................................................................................................................................................GTACTAAAAAGTCAACGGTGT...... 1

..............................................................................................................................................................GTACTAAAAAGTCAACGGTGTCAA... 1

...............................................................................................................................................................TACTAAAAAGTCAACGGTGT...... 1

...............................................................................................................................................................TACTAAAAAGTCAACGGTGTC..... 4

...............................................................................................................................................................TACTAAAAAGTCAACGGTGTCA.... 1

................................................................................................................................................................ACTAAAAAGTCAACGGTGTC..... 1

................................................................................................................................................................ACTAAAAAGTCAACGGTGTCA.... 3

................................................................................................................................................................ACTAAAAAGTCAACGGTGTCAA... 5

................................................................................................................................................................ACTAAAAAGTCAACGGTGTCAAAC. 30

................................................................................................................................................................ACTAAAAAGTCAACGGTGTCAA... 1

.................................................................................................................................................................CTAAAAAGTCAACGGTGTCAA... 5

.................................................................................................................................................................CTAAAAAGTCAACGGTGTCAAA.. 2

.................................................................................................................................................................CTAAAAAGTCAACGGTGTCAA... 6

.................................................................................................................................................................CTAAAAAGTCAACGGTGTCAAA.. 7

..................................................................................................................................................................TAAAAAGTCAACGGTGTCAAA.. 7

..................................................................................................................................................................TAAAAAGTCAACGGTGTCAA... 3

..................................................................................................................................................................TAAAAAGTCAACGGTGTCAAA.. 6

...................................................................................................................................................................AAAAAGTCAACGGTGTCAAA.. 1

...................................................................................................................................................................AAAAAGTCAACGGTGTCAAAC. 3

...................................................................................................................................................................AAAAAGTCAACGGTGTCAAACA 5

...................................................................................................................................................................AAAAAGTCAACGGTGTCAA... 36

...................................................................................................................................................................AAAAAGTCAACGGTGTCAAA.. 23

....................................................................................................................................................................AAAAGTCAACGGTGTCAAAC. 1

....................................................................................................................................................................AAAAGTCAACGGTGTCAAACA 4

....................................................................................................................................................................AAAAGTCAACGGTGTCAAACA 1

....................................................................................................................................................................AAAAGTCAACGGTGTCAA... 4

....................................................................................................................................................................AAAAGTCAACGGTGTCAAA.. 12

.....................................................................................................................................................................AAAGTCAACGGTGTCAAA.. 1

.....................................................................................................................................................................AAAGTCAACGGTGTCAAAC. 2

.....................................................................................................................................................................AAAGTCAACGGTGTCAAACA 2

.....................................................................................................................................................................AAAGTCAACGGTGTCAAA.. 4

>osa-MIR2123b_MI0010713_Oryza_sativa_miR2123b_stem-loop GSM361264

UUUGACACCGUUGACUUUUUAGUACAUGUCUGACCGUUCGUCUUGUGAAAUAUGUAAAACUAGAUGUGUACAUGAAAAUACAUUUAACAAGGAAUCAAAUGAUAUGAAAAGAAUAAAUAAUUACUUAAAAUUUUUGAAUAAGACGAAUGGUCAAACACGUCCUAAAAAGUCAACGGUGUCAAACA

((((((((((((((((((((((.((.(((.(((((((((((((((((((((..((((...(((((((((........))))))))).......(((....))).................)))).....)))))...)))))))))))))))).))).)).)))))))))))))))))))))).. (-71.70)

TTTGACACCGTTGACTTTTT..................................................................................................................................................................... 1

TTTGACACCGTTGACTTT....................................................................................................................................................................... 1

TTTGACACCGTTGACTTTTTAG................................................................................................................................................................... 2

TTTGACACCGTTGACTTTTTA.................................................................................................................................................................... 7

TTTGACACCGTTGACTTT....................................................................................................................................................................... 4

TTTGACACCGTTGACTTTT...................................................................................................................................................................... 12

TTTGACACCGTTGACTTTTT..................................................................................................................................................................... 23

TTTGACACCGTTGACTTTTTA.................................................................................................................................................................... 6

TTTGACACCGTTGACTTTTTAG................................................................................................................................................................... 7

.TTGACACCGTTGACTTTTTAGT.................................................................................................................................................................. 5

.TTGACACCGTTGACTTTTTAG................................................................................................................................................................... 5

.TTGACACCGTTGACTTTT...................................................................................................................................................................... 4

.TTGACACCGTTGACTTTTT..................................................................................................................................................................... 36

.TTGACACCGTTGACTTTTTA.................................................................................................................................................................... 3

.TTGACACCGTTGACTTTTTAG................................................................................................................................................................... 6

.TTGACACCGTTGACTTTTTAGT.................................................................................................................................................................. 1

.TTGACACCGTTGACTTTTTAGTAC................................................................................................................................................................ 1

..TGACACCGTTGACTTTTTAGT.................................................................................................................................................................. 3

..TGACACCGTTGACTTTTTAGTA................................................................................................................................................................. 1

...GACACCGTTGACTTTTTAGT.................................................................................................................................................................. 1

...GACACCGTTGACTTTTTAGTA................................................................................................................................................................. 4

...GACACCGTTGACTTTTTAGTACA............................................................................................................................................................... 1

....ACACCGTTGACTTTTTAGTAC................................................................................................................................................................ 1

....ACACCGTTGACTTTTTAGTA................................................................................................................................................................. 1

.....CACCGTTGACTTTTTAGTACATGT............................................................................................................................................................ 1

.......CCGTTGACTTTTTAGTACATGTCT.......................................................................................................................................................... 2

........CGTTGACTTTTTAGTACA............................................................................................................................................................... 1

....................................................................................................................ATAATTACTTAAAATTTTTGAATA............................................. 1

.........................................................................................................................TACTTAAAATTTTTGAATAAGA.......................................... 1

.........................................................................................................................TACTTAAAATTTTTGAATAAGACG........................................ 1

..........................................................................................................................ACTTAAAATTTTTGAATAAGACGA....................................... 9

...........................................................................................................................CTTAAAATTTTTGAATAAGACGA....................................... 1

............................................................................................................................TTAAAATTTTTGAATAAGACGAA...................................... 1

............................................................................................................................TTAAAATTTTTGAATAAGACGAAT..................................... 1

.............................................................................................................................TAAAATTTTTGAATAAGACGA....................................... 9

.............................................................................................................................TAAAATTTTTGAATAAGACGAA...................................... 5

.............................................................................................................................TAAAATTTTTGAATAAGACGAAT..................................... 17

.............................................................................................................................TAAAATTTTTGAATAAGACGAATG.................................... 3

..............................................................................................................................AAAATTTTTGAATAAGACGA....................................... 1

..............................................................................................................................AAAATTTTTGAATAAGACGAA...................................... 3

..............................................................................................................................AAAATTTTTGAATAAGACGAAT..................................... 1

..............................................................................................................................AAAATTTTTGAATAAGACGAATG.................................... 23

..............................................................................................................................AAAATTTTTGAATAAGACGAATGG................................... 103

..............................................................................................................................AAAATTTTTGAATAAGACGAATGGT.................................. 2

...............................................................................................................................AAATTTTTGAATAAGACGAA...................................... 1

...............................................................................................................................AAATTTTTGAATAAGACGAATG.................................... 1

...............................................................................................................................AAATTTTTGAATAAGACGAATGG................................... 9

...............................................................................................................................AAATTTTTGAATAAGACGAATGGT.................................. 9

...............................................................................................................................AAATTTTTGAATAAGACGAATGGTC................................. 1

................................................................................................................................AATTTTTGAATAAGACGAATG.................................... 1

................................................................................................................................AATTTTTGAATAAGACGAATGGT.................................. 1

..................................................................................................................................TTTTTGAATAAGACGAATGGT.................................. 1

..................................................................................................................................TTTTTGAATAAGACGAATGG................................... 2

..................................................................................................................................TTTTTGAATAAGACGAATGGTCAA............................... 1

..................................................................................................................................TTTTTGAATAAGACGAAT..................................... 1

..................................................................................................................................TTTTTGAATAAGACGAATG.................................... 2

..................................................................................................................................TTTTTGAATAAGACGAATGG................................... 3

..................................................................................................................................TTTTTGAATAAGACGAATGGT.................................. 13

..................................................................................................................................TTTTTGAATAAGACGAATGGTCAA............................... 5

...................................................................................................................................TTTTGAATAAGACGAATGGTCA................................ 1

...................................................................................................................................TTTTGAATAAGACGAATGG................................... 1

...................................................................................................................................TTTTGAATAAGACGAATGGT.................................. 1

...................................................................................................................................TTTTGAATAAGACGAATGGTCAA............................... 2

...................................................................................................................................TTTTGAATAAGACGAATGGTCAAA.............................. 2

....................................................................................................................................TTTGAATAAGACGAATGGTCAAAC............................. 1

....................................................................................................................................TTTGAATAAGACGAATGGTCA................................ 1

....................................................................................................................................TTTGAATAAGACGAATGGTC................................. 1

....................................................................................................................................TTTGAATAAGACGAATGGTCA................................ 48

....................................................................................................................................TTTGAATAAGACGAATGGTCAA............................... 4

....................................................................................................................................TTTGAATAAGACGAATGGTCAAA.............................. 1

....................................................................................................................................TTTGAATAAGACGAATGGTCAAAC............................. 4

.....................................................................................................................................TTGAATAAGACGAATGGT.................................. 1

.....................................................................................................................................TTGAATAAGACGAATGGTCA................................ 1

.....................................................................................................................................TTGAATAAGACGAATGGTCAA............................... 10

.....................................................................................................................................TTGAATAAGACGAATGGTCAAA.............................. 2

.....................................................................................................................................TTGAATAAGACGAATGGTCAAACA............................ 8

.....................................................................................................................................TTGAATAAGACGAATGGTCAA............................... 2

......................................................................................................................................TGAATAAGACGAATGGTCAAA.............................. 1

......................................................................................................................................TGAATAAGACGAATGGTCAAACA............................ 9

......................................................................................................................................TGAATAAGACGAATGGTCAAACAC........................... 8

........................................................................................................................................AATAAGACGAATGGTCAAA.............................. 1

........................................................................................................................................AATAAGACGAATGGTCAAACA............................ 28

........................................................................................................................................AATAAGACGAATGGTCAAACACGT......................... 1

........................................................................................................................................AATAAGACGAATGGTCAAACACG.......................... 1

........................................................................................................................................AATAAGACGAATGGTCAAACA............................ 2

.........................................................................................................................................ATAAGACGAATGGTCAAA.............................. 1

.........................................................................................................................................ATAAGACGAATGGTCAAACA............................ 13

.........................................................................................................................................ATAAGACGAATGGTCAAACAC........................... 1

.........................................................................................................................................ATAAGACGAATGGTCAAACACG.......................... 1

..........................................................................................................................................TAAGACGAATGGTCAAACA............................ 1

..................................................................................................................................................ATGGTCAAACACGTCCTAAA................... 1

...................................................................................................................................................TGGTCAAACACGTCCTAAAAAG................ 1

......................................................................................................................................................TCAAACACGTCCTAAAAAGTCAAC........... 1

........................................................................................................................................................AAACACGTCCTAAAAAGTCAACGG......... 2

.........................................................................................................................................................AACACGTCCTAAAAAGTCAACGGT........ 1

..........................................................................................................................................................ACACGTCCTAAAAAGTCAACGGTG....... 1

.................................................................................................................................................................CTAAAAAGTCAACGGTGTCAA... 5

.................................................................................................................................................................CTAAAAAGTCAACGGTGTCAAA.. 2

.................................................................................................................................................................CTAAAAAGTCAACGGTGTCAA... 6

.................................................................................................................................................................CTAAAAAGTCAACGGTGTCAAA.. 7

..................................................................................................................................................................TAAAAAGTCAACGGTGTCAAA.. 7

..................................................................................................................................................................TAAAAAGTCAACGGTGTCAA... 3

..................................................................................................................................................................TAAAAAGTCAACGGTGTCAAA.. 6

...................................................................................................................................................................AAAAAGTCAACGGTGTCAAA.. 1

...................................................................................................................................................................AAAAAGTCAACGGTGTCAAAC. 3

...................................................................................................................................................................AAAAAGTCAACGGTGTCAAACA 5

...................................................................................................................................................................AAAAAGTCAACGGTGTCAA... 36

...................................................................................................................................................................AAAAAGTCAACGGTGTCAAA.. 23

....................................................................................................................................................................AAAAGTCAACGGTGTCAAAC. 1

....................................................................................................................................................................AAAAGTCAACGGTGTCAAACA 4

....................................................................................................................................................................AAAAGTCAACGGTGTCAAACA 1

....................................................................................................................................................................AAAAGTCAACGGTGTCAA... 4

....................................................................................................................................................................AAAAGTCAACGGTGTCAAA.. 12

.....................................................................................................................................................................AAAGTCAACGGTGTCAAA.. 1

.....................................................................................................................................................................AAAGTCAACGGTGTCAAAC. 2

.....................................................................................................................................................................AAAGTCAACGGTGTCAAACA 2

.....................................................................................................................................................................AAAGTCAACGGTGTCAAA.. 4

>osa-MIR2124f_MI0010720_Oryza_sativa_miR2124f_stem-loop GSM361264

ACGUUUGAACACAUGCAUGGAGUAUUAAAUGUUGACGAAAAAAUAACUACUUAAACAGUUUGCAUGUAAAUUGCGAGAUGAAUCUUUUAACUCUAAUUGCGCCAUGAUUUGAUAAUAUGGUGUUACAGUAAACUUUUGCUAAUGAUAGAUUAAUUAGCCUUAACAAAUUCGUCUCUUGGUUUACAGGCGGAUCCUGCAAUUUGUUUUGUUAUUAGACUACGUUUAAUACUUUAAAUGUGUGUCCGUAUAUUC

(((.....((((((...(((((((((((((((.(.(.....((((((.....((((((.((((((((((((((.(((((((((.(.((((..((((((((((((((.((....))))))))))...(((((....)))))...........))))))..)))).).))))))))).))))))))).........))))).)))))).)))))).).).))))))))))))))).))))))...)))...... (-62.80)

....TTGAACACATGCATGGAGTATTA................................................................................................................................................................................................................................. 1

...............CATGGAGTATTAAATGTTGACGA...................................................................................................................................................................................................................... 1

.................TGGAGTATTAAATGTTGACGA...................................................................................................................................................................................................................... 1

...........................................................TTGCATGTAAATTGCGAGATG............................................................................................................................................................................ 1

...........................................................................................................TTTGATAATATGGTGTTACAG............................................................................................................................ 1

.......................................................................................................................................................................TTCGTCTCTTGGTTTACAGGCGGAT............................................................ 1

.......................................................................................................................................................................TTCGTCTCTTGGTTTACAGGC................................................................ 1

.......................................................................................................................................................................TTCGTCTCTTGGTTTACAGGCGGA............................................................. 1

........................................................................................................................................................................TCGTCTCTTGGTTTACAGGCGGAT............................................................ 3

........................................................................................................................................................................TCGTCTCTTGGTTTACAGGCG............................................................... 1

........................................................................................................................................................................TCGTCTCTTGGTTTACAGGCGGA............................................................. 1

......................................................................................................................................................................................................ATTTGTTTTGTTATTAGACTACG............................... 1

.......................................................................................................................................................................................................TTTGTTTTGTTATTAGACTACGTT............................. 1

........................................................................................................................................................................................................TTGTTTTGTTATTAGACTACGTTT............................ 3

.........................................................................................................................................................................................................TGTTTTGTTATTAGACTACGTTT............................ 1

..........................................................................................................................................................................................................GTTTTGTTATTAGACTACGTTT............................ 1

...........................................................................................................................................................................................................TTTTGTTATTAGACTACGTTTAA.......................... 1

............................................................................................................................................................................................................TTTGTTATTAGACTACGTTTA........................... 1

..............................................................................................................................................................................................................TGTTATTAGACTACGTTTAATAC....................... 1

................................................................................................................................................................................................................TTATTAGACTACGTTTAATACTT..................... 1

..................................................................................................................................................................................................................ATTAGACTACGTTTAATACTTTAA.................. 3

...................................................................................................................................................................................................................TTAGACTACGTTTAATACTT..................... 1

.....................................................................................................................................................................................................................AGACTACGTTTAATACTTTAAATG............... 1

.......................................................................................................................................................................................................................ACTACGTTTAATACTTTAAATGTG............. 2

........................................................................................................................................................................................................................CTACGTTTAATACTTTAAATGTGT............ 1

..........................................................................................................................................................................................................................ACGTTTAATACTTTAAATGTGTGT.......... 1

..............................................................................................................................................................................................................................TTAATACTTTAAATGTGTGTCCGT...... 3

................................................................................................................................................................................................................................AATACTTTAAATGTGTGTCCGTAT.... 1

..................................................................................................................................................................................................................................TACTTTAAATGTGTGTCCGTAT.... 1

..................................................................................................................................................................................................................................TACTTTAAATGTGTGTCCGTATAT.. 9

..................................................................................................................................................................................................................................TACTTTAAATGTGTGTCCGTAT.... 1

..................................................................................................................................................................................................................................TACTTTAAATGTGTGTCCGTATAT.. 1

...................................................................................................................................................................................................................................ACTTTAAATGTGTGTCCGTATATT. 14

...................................................................................................................................................................................................................................ACTTTAAATGTGTGTCCGTATAT.. 30

....................................................................................................................................................................................................................................CTTTAAATGTGTGTCCGTATATT. 5

....................................................................................................................................................................................................................................CTTTAAATGTGTGTCCGTATAT.. 2

....................................................................................................................................................................................................................................CTTTAAATGTGTGTCCGTATATTC 2

....................................................................................................................................................................................................................................CTTTAAATGTGTGTCCGTATA... 1

.....................................................................................................................................................................................................................................TTTAAATGTGTGTCCGTAT.... 1

.....................................................................................................................................................................................................................................TTTAAATGTGTGTCCGTATAT.. 5

.....................................................................................................................................................................................................................................TTTAAATGTGTGTCCGTATATTC 2

.......................................................................................................................................................................................................................................TAAATGTGTGTCCGTATAT.. 1

.......................................................................................................................................................................................................................................TAAATGTGTGTCCGTATA... 1

>osa-MIR2124g_MI0010721_Oryza_sativa_miR2124g_stem-loop GSM361264

AUGCUUGGACAUAACAUGGAGUAUUAAAUAUAAUCGAGAAAAUAACUAAUUACACAGAUUGCGUGUAAAUUGCGACACGAAUCUUUUAAGCAUAAUUGCUCCAUGAUCUGACAAUGUGAUGCUACAGUAAACAUUUGCUAAUAACGAAUUAAUUAGUCUUAAUAAAUUCGUCUCGCGGUUUACAGGCGGAUUCUCUAAUUUGUUUUGUUAUUAGUCUACAUUUAAUACUUUAAAUGUGUGUCCGUAUAUUC

((((..(((((((.(((.(((((((((((.......(((.((((((.(((.....(((((((.((((((((((((.((((((...(((((..((((((.......((..(..(((((..(((....))).)))))..)..)).......))))))..)))))...)))))).)))))))))))).))))....))).....)))..))))))..)))..)))))))))))...)))))))))))))).... (-63.14)

......................................AAAATAACTAATTACACAGATTGC............................................................................................................................................................................................. 9

........................................AATAACTAATTACACAGATTGCG............................................................................................................................................................................................ 1

...................................................ACACAGATTGCGTGTAAATTGCG................................................................................................................................................................................. 1

............................................................................................TAATTGCTCCATGATCTGACAATG....................................................................................................................................... 2

.............................................................................................AATTGCTCCATGATCTGACAATGT...................................................................................................................................... 3

..............................................................................................ATTGCTCCATGATCTGACAATGTG..................................................................................................................................... 1

..................................................................................................CTCCATGATCTGACAATGTGATGC................................................................................................................................. 1

...................................................................................................TCCATGATCTGACAATGTGAT................................................................................................................................... 1

............................................................................................................TGACAATGTGATGCTACAGTAAAC....................................................................................................................... 6

............................................................................................................TGACAATGTGATGCTACAGTAAA........................................................................................................................ 2

.............................................................................................................GACAATGTGATGCTACAGTAAAC....................................................................................................................... 1

..............................................................................................................ACAATGTGATGCTACAGTAAACAT..................................................................................................................... 2

..............................................................................................................ACAATGTGATGCTACAGTAAA........................................................................................................................ 1

................................................................................................................AATGTGATGCTACAGTAAACATT.................................................................................................................... 1

..................................................................................................................TGTGATGCTACAGTAAACATTTGCT................................................................................................................ 1

...................................................................................................................GTGATGCTACAGTAAACATTTGCT................................................................................................................ 2

...................................................................................................................GTGATGCTACAGTAAACATTTGC................................................................................................................. 1

......................................................................................................................ATGCTACAGTAAACATTTGCTAAT............................................................................................................. 1

......................................................................................................................................................AATTAGTCTTAATAAATTCGTCTC............................................................................. 1

.............................................................................................................................................................CTTAATAAATTCGTCTCGCGGTTT...................................................................... 5

..............................................................................................................................................................TTAATAAATTCGTCTCGCGGTT....................................................................... 1

..............................................................................................................................................................TTAATAAATTCGTCTCGCGGTTTA..................................................................... 1

..............................................................................................................................................................TTAATAAATTCGTCTCGCGGTT....................................................................... 2

...............................................................................................................................................................TAATAAATTCGTCTCGCGGTTTAC.................................................................... 1

................................................................................................................................................................AATAAATTCGTCTCGCGGTTT...................................................................... 1

................................................................................................................................................................AATAAATTCGTCTCGCGGTTTACA................................................................... 1

......................................................................................................................................................................TTCGTCTCGCGGTTTACAGGCGG.............................................................. 1

......................................................................................................................................................................TTCGTCTCGCGGTTTACAGGCGGA............................................................. 3

.......................................................................................................................................................................TCGTCTCGCGGTTTACAGGCGGAT............................................................ 5

.......................................................................................................................................................................TCGTCTCGCGGTTTACAGG................................................................. 1

.......................................................................................................................................................................TCGTCTCGCGGTTTACAGGCG............................................................... 2

.......................................................................................................................................................................TCGTCTCGCGGTTTACAGGCGGA............................................................. 1

..........................................................................................................................................................................TCTCGCGGTTTACAGGCGGATTC.......................................................... 1

.....................................................................................................................................................................................ACAGGCGGATTCTCTAATTTGTTT.............................................. 1

......................................................................................................................................................................................................TTTGTTTTGTTATTAGTCTACATT............................. 1

.......................................................................................................................................................................................................TTGTTTTGTTATTAGTCT.................................. 1

........................................................................................................................................................................................................TGTTTTGTTATTAGTCTACATTT............................ 1

............................................................................................................................................................................................................TTGTTATTAGTCTACATTTAATAC....................... 1

...............................................................................................................................................................................................................TTATTAGTCTACATTTAATACTTT.................... 1

.............................................................................................................................................................................................................................TTAATACTTTAAATGTGTGTCCGT...... 3

...............................................................................................................................................................................................................................AATACTTTAAATGTGTGTCCGTAT.... 1

.................................................................................................................................................................................................................................TACTTTAAATGTGTGTCCGTAT.... 1

.................................................................................................................................................................................................................................TACTTTAAATGTGTGTCCGTATAT.. 9

.................................................................................................................................................................................................................................TACTTTAAATGTGTGTCCGTAT.... 1

.................................................................................................................................................................................................................................TACTTTAAATGTGTGTCCGTATAT.. 1

..................................................................................................................................................................................................................................ACTTTAAATGTGTGTCCGTATATT. 14

..................................................................................................................................................................................................................................ACTTTAAATGTGTGTCCGTATAT.. 30

...................................................................................................................................................................................................................................CTTTAAATGTGTGTCCGTATATT. 5

...................................................................................................................................................................................................................................CTTTAAATGTGTGTCCGTATAT.. 2

...................................................................................................................................................................................................................................CTTTAAATGTGTGTCCGTATATTC 2

...................................................................................................................................................................................................................................CTTTAAATGTGTGTCCGTATA... 1

....................................................................................................................................................................................................................................TTTAAATGTGTGTCCGTAT.... 1

....................................................................................................................................................................................................................................TTTAAATGTGTGTCCGTATAT.. 5

....................................................................................................................................................................................................................................TTTAAATGTGTGTCCGTATATTC 2

......................................................................................................................................................................................................................................TAAATGTGTGTCCGTATAT.. 1

......................................................................................................................................................................................................................................TAAATGTGTGTCCGTATA... 1

>osa-MIR2124h_MI0010724_Oryza_sativa_miR2124h_stem-loop GSM361264

GUCAAAUCAAAUGUUUGGACACAUGCAUGGAGUAUUAAAUAUAAUAAAAAUAACUAAUUACACAGAUUGUGUGUAAAUUACGAGAUGAAUCUUUUAAGCCUAAUUGCUUCUUGAUUUGACAAUGUGGUGCUACAAUGUGGUGCUACAGUAAACAUAUGCUAACGAGGGAUUAAUUAGGCUUAAUAAAUUUGUCUCGCGGUUUACAAGUGGAAUCUGUAAUUUGUUUUAUUACUAGUCUAUGUUUAAUACUUUAAAUGUGUGUCCGUAUAUUCGAU

.....(((.(((((.((((((((((..((((((((((((((((.((..(((((........(((((((.(.(((((((..(((((..(((...(((((((((((((.((((((.........(((((..((((...))))..)))))(((......)))...))))))..)))))))))))))...)))..)))))..)))))))...).)))))))........)))))..))...))))))))))))))))..)))))))))).))))).))) (-92.59)

.....ATCAAATGTTTGGACACATG.......................................................................................................................................................................................................................................................... 1

.....ATCAAATGTTTGGACACATGCAT....................................................................................................................................................................................................................................................... 1

.....ATCAAATGTTTGGACACATGCATG...................................................................................................................................................................................................................................................... 95

.....ATCAAATGTTTGGACACATGCATGG..................................................................................................................................................................................................................................................... 1

......TCAAATGTTTGGACACATGCATGG..................................................................................................................................................................................................................................................... 1

.......CAAATGTTTGGACACATGCATG...................................................................................................................................................................................................................................................... 2

.......CAAATGTTTGGACACATGCATGG..................................................................................................................................................................................................................................................... 4

........AAATGTTTGGACACATGCA........................................................................................................................................................................................................................................................ 1

........AAATGTTTGGACACATGCATG...................................................................................................................................................................................................................................................... 27

........AAATGTTTGGACACATGCATGG..................................................................................................................................................................................................................................................... 1

........AAATGTTTGGACACATGCATGGAG................................................................................................................................................................................................................................................... 9

.........AATGTTTGGACACATGCAT....................................................................................................................................................................................................................................................... 4

.........AATGTTTGGACACATGCATG...................................................................................................................................................................................................................................................... 3

.........AATGTTTGGACACATGCATGG..................................................................................................................................................................................................................................................... 1

.........AATGTTTGGACACATGCATGGA.................................................................................................................................................................................................................................................... 2

.........AATGTTTGGACACATGCATGGAG................................................................................................................................................................................................................................................... 12

.........AATGTTTGGACACATGCATGGAGT.................................................................................................................................................................................................................................................. 5

..........ATGTTTGGACACATGCAT....................................................................................................................................................................................................................................................... 1

..........ATGTTTGGACACATGCATGGA.................................................................................................................................................................................................................................................... 1

..........ATGTTTGGACACATGCATGGAG................................................................................................................................................................................................................................................... 2

..........ATGTTTGGACACATGCATGGAGT.................................................................................................................................................................................................................................................. 4

..........ATGTTTGGACACATGCATGGAGTA................................................................................................................................................................................................................................................. 1

...........TGTTTGGACACATGCATGGAGTATT............................................................................................................................................................................................................................................... 1

...........TGTTTGGACACATGCATGGAG................................................................................................................................................................................................................................................... 6

...........TGTTTGGACACATGCATGGAGT.................................................................................................................................................................................................................................................. 1

...........TGTTTGGACACATGCATGGAGTA................................................................................................................................................................................................................................................. 1

...........TGTTTGGACACATGCATGGAGTAT................................................................................................................................................................................................................................................ 7

............GTTTGGACACATGCATGGAG................................................................................................................................................................................................................................................... 1

............GTTTGGACACATGCATGGAGTA................................................................................................................................................................................................................................................. 1

............GTTTGGACACATGCATGGAGTATT............................................................................................................................................................................................................................................... 1

.............TTTGGACACATGCATGGAGTATT............................................................................................................................................................................................................................................... 1

.............TTTGGACACATGCATGGAGT.................................................................................................................................................................................................................................................. 1

.............TTTGGACACATGCATGGAGTAT................................................................................................................................................................................................................................................ 1

.............TTTGGACACATGCATGGAGTATT............................................................................................................................................................................................................................................... 1

.............TTTGGACACATGCATGGAGTATTA.............................................................................................................................................................................................................................................. 1

..............TTGGACACATGCATGGAGTA................................................................................................................................................................................................................................................. 1

..............TTGGACACATGCATGGAGTAT................................................................................................................................................................................................................................................ 5

..............TTGGACACATGCATGGAGTATT............................................................................................................................................................................................................................................... 1

..............TTGGACACATGCATGGAGTATTA.............................................................................................................................................................................................................................................. 11

..............TTGGACACATGCATGGAGTATTAA............................................................................................................................................................................................................................................. 3

...............TGGACACATGCATGGAGTATT............................................................................................................................................................................................................................................... 1

...............TGGACACATGCATGGAGTATT............................................................................................................................................................................................................................................... 1

...............TGGACACATGCATGGAGTATTAA............................................................................................................................................................................................................................................. 1

................GGACACATGCATGGAGTATTAAA............................................................................................................................................................................................................................................ 1

..................ACACATGCATGGAGTATTAAATA.......................................................................................................................................................................................................................................... 1

..................ACACATGCATGGAGTATTAAATAT......................................................................................................................................................................................................................................... 1

...................CACATGCATGGAGTATTAAATAT......................................................................................................................................................................................................................................... 1

.................................................ATAACTAATTACACAGATTGT............................................................................................................................................................................................................. 1

.................................................................................GAGATGAATCTTTTAAGCCTAAT........................................................................................................................................................................... 1

...............................................................................................................TGATTTGACAATGTGGTGCTACA............................................................................................................................................. 1

................................................................................................................GATTTGACAATGTGGTGCTACAAT........................................................................................................................................... 1

...................................................................................................................................ACAATGTGGTGCTACAGTAAACA......................................................................................................................... 1

...................................................................................................................................ACAATGTGGTGCTACAGTAAACAT........................................................................................................................ 1

......................................................................................................................................ATGTGGTGCTACAGTAAAC.......................................................................................................................... 1

.......................................................................................................................................................................GATTAATTAGGCTTAATAAATTTG.................................................................................... 1

..................................................................................................................................................................................CTTAATAAATTTGTCTCGCGGTTT......................................................................... 3

..................................................................................................................................................................................CTTAATAAATTTGTCTCGCGG............................................................................ 1

............................................................................................................................................................................................................................................CTATGTTTAATACTTTAAATGTGT............... 1

..................................................................................................................................................................................................................................................TTAATACTTTAAATGTGTGTCCGT......... 3

....................................................................................................................................................................................................................................................AATACTTTAAATGTGTGTCCGTAT....... 1

......................................................................................................................................................................................................................................................TACTTTAAATGTGTGTCCGTAT....... 1

......................................................................................................................................................................................................................................................TACTTTAAATGTGTGTCCGTATAT..... 9

......................................................................................................................................................................................................................................................TACTTTAAATGTGTGTCCGTAT....... 1

......................................................................................................................................................................................................................................................TACTTTAAATGTGTGTCCGTATAT..... 1

.......................................................................................................................................................................................................................................................ACTTTAAATGTGTGTCCGTATATT.... 14

.......................................................................................................................................................................................................................................................ACTTTAAATGTGTGTCCGTATAT..... 30

........................................................................................................................................................................................................................................................CTTTAAATGTGTGTCCGTATATT.... 5

........................................................................................................................................................................................................................................................CTTTAAATGTGTGTCCGTATAT..... 2

........................................................................................................................................................................................................................................................CTTTAAATGTGTGTCCGTATATTC... 2

........................................................................................................................................................................................................................................................CTTTAAATGTGTGTCCGTATA...... 1

.........................................................................................................................................................................................................................................................TTTAAATGTGTGTCCGTAT....... 1

.........................................................................................................................................................................................................................................................TTTAAATGTGTGTCCGTATAT..... 5

.........................................................................................................................................................................................................................................................TTTAAATGTGTGTCCGTATATTC... 2

..........................................................................................................................................................................................................................................................TTAAATGTGTGTCCGTATATTCGA. 1

...........................................................................................................................................................................................................................................................TAAATGTGTGTCCGTATAT..... 1

...........................................................................................................................................................................................................................................................TAAATGTGTGTCCGTATA...... 1

...........................................................................................................................................................................................................................................................TAAATGTGTGTCCGTATATTCGA. 1

.............................................................................................................................................................................................................................................................AATGTGTGTCCGTATATTCGA. 1

>osa-MIR156i_MI0000661_Oryza_sativa_miR156i_stem-loop GSM278571

GGUGACAGAAGAGAGUGAGCACACGGCCGGGCGGAACGGCACCGGCGGAUGUGCCGUCGCGGCCGCGUGCUCACUGCUCUGUCUGUCAUC

((((((((((((((((((((((.((((((.(....(((((((........)))))))).)))))).))))))))).)))).))))))))) (-60.70)

..TGACAGAAGAGAGTGAGCA..................................................................... 67

..TGACAGAAGAGAGTGAGCACA................................................................... 69

..TGACAGAAGAGAGTGAGCAC.................................................................... 29513

..TGACAGAAGAGAGTGAGC...................................................................... 35

...GACAGAAGAGAGTGAGCA..................................................................... 1

...GACAGAAGAGAGTGAGCAC.................................................................... 163

...GACAGAAGAGAGTGAGCACA................................................................... 5

....ACAGAAGAGAGTGAGCACAC.................................................................. 1

....ACAGAAGAGAGTGAGCACA................................................................... 1

....ACAGAAGAGAGTGAGCAC.................................................................... 13

.............................................CGGATGTGCCGTCGCGGCCGCG....................... 1

...................................................................TGCTCACTGCTCTGTCTGTCATC 1

....................................................................GCTCACTGCTCTGTCTGTCATC 3

>osa-MIR169a_MI0000679_Oryza_sativa_miR169a_stem-loop GSM278571

CGCCGGCGGCCUGACAUUGGGAUCGGAGGCCAUGGUGCAGCCAAGGAUGACUUGCCGAUCGAUCGAUCUAUCUAUGAAGCUAAGCUAGCUGGCCAUGGAUCCAUCCAUCAAUUGGCAAGUUGUUCUUGGCUACAUCUUGGCCCCUGCUCCUCAUGUAAGGCCGGCCUGUGGCG

((((((((((((.((((.((((.(((.(((((.((((.(((((((((..((((((((((.(((.(((..(((((((.(((((...)))))...)))))))..))).))).))))))))))..))))))))).)))).))))).))).)))).)))).))))).)))...)))) (-99.60)

................TTGGGATCGGAGGCCATGGTG........................................................................................................................................ 5

.....................ATCGGAGGCCATGGTGCAGCC................................................................................................................................... 6

....................................GCAGCCAAGGATGACTTGCCGA................................................................................................................... 1

.....................................CAGCCAAGGATGACTTGCCGA................................................................................................................... 21

.....................................CAGCCAAGGATGACTTGCCGATCG................................................................................................................ 1

..........................................AAGGATGACTTGCCGATCGATCGA........................................................................................................... 115

..........................................AAGGATGACTTGCCGATCGAT.............................................................................................................. 1

..........................................AAGGATGACTTGCCGATCGATCGAT.......................................................................................................... 3

..........................................AAGGATGACTTGCCGATCGATCG............................................................................................................ 15

...........................................AGGATGACTTGCCGATCGATCGA........................................................................................................... 1

......................................................................................................................................TCTTGGCCCCTGCTCCTCATG.................. 1

>osa-MIR159a_MI0001092_Oryza_sativa_miR159a_stem-loop GSM278571

GUUGUGGACGUUGAGCUCCUUUCGGUCCAAAAAGGGGUGUUGCUGUGGGUCGAUUGAGCUGCUGGGUCAUGGAUCCCGUUAGCCUACUCCAUGUUCAUCAUUCAGCUCGAGAUCUGAAAGAAACUACUCCAAUUUAUACUAAUAGUAUGUGUGUAGAUAGGAAAAUGAUGGAGUACUCGUUGUUGGGAUAGGCUUAUGGCUUGCAUGCCCCAGGAGCUGCAUCAACCCUACAUGGACCCUCUUUGGAUUGAAGGGAGCUCUGCAUCUUUUGU

.....(((.((.((((((((((((((((((..(((((.(((..(((((((.(((..((((.(((((.((((.(..((((.((((((..(((..((((((((((((........))))..........(((.((((((((...........)))))))).)))..))))))))..((.....)))))..)))))).))))..).)))).))))).))))..))).)))).)))..)))))))))))))))))))))))))).)).)))..... (-114.70)

..........TTGAGCTCCTTTCGGTCCAAA................................................................................................................................................................................................................................................. 7

...........TGAGCTCCTTTCGGTCCAAAAA............................................................................................................................................................................................................................................... 1

............GAGCTCCTTTCGGTCCAAAAA............................................................................................................................................................................................................................................... 1

.......................................................GAGCTGCTGGGTCATGGATC..................................................................................................................................................................................................... 1

........................................................AGCTGCTGGGTCATGGATCC.................................................................................................................................................................................................... 2

........................................................AGCTGCTGGGTCATGGATCCC................................................................................................................................................................................................... 5

........................................................AGCTGCTGGGTCATGGATC..................................................................................................................................................................................................... 3

.......................................................................................................................................................................................................CTTGCATGCCCCAGGAGCTGC.................................................... 1

........................................................................................................................................................................................................TTGCATGCCCCAGGAGCTGC.................................................... 12

........................................................................................................................................................................................................TTGCATGCCCCAGGAGCTGCA................................................... 2

............................................................................................................................................................................................................................ATCAACCCTACATGGACCCT................................ 1

............................................................................................................................................................................................................................ATCAACCCTACATGGACCCTCT.............................. 2

............................................................................................................................................................................................................................ATCAACCCTACATGGACCCTC............................... 3

.................................................................................................................................................................................................................................................TTTGGATTGAAGGGAGCT............. 19

.................................................................................................................................................................................................................................................TTTGGATTGAAGGGAGCTCTGCA........ 1

.................................................................................................................................................................................................................................................TTTGGATTGAAGGGAGCTCTG.......... 25

.................................................................................................................................................................................................................................................TTTGGATTGAAGGGAGCTC............ 1

..................................................................................................................................................................................................................................................TTGGATTGAAGGGAGCTCTG.......... 2

>osa-MIR159b_MI0001093_Oryza_sativa_miR159b_stem-loop GSM278571

GGUUAUGAAGUGGAGCUCCUUUCGUUCCAAUGAAAGGUUUAUCUGAAGGGUGAUACAGCUGCUUGUUCAUGGUUCCCACUAUUCUAUCUCAUAGGAAAAGAGAUAGGCUUGUGGUUUGCAUGACCAAGGAGCCGAAUCAACUCCUUGCUGACCACUCUUUGGAUUGAAGGGAGCUCUGCAUCUUGAUC

(((((.((.(..((((((((((((.(((((.((..((((......(((((((((.(.(((.((((.(((((....((((...((((((((.........))))))))...))))....))))).)))).))).).))).)).))))...))))..)).))))).))))))))))))..).)).))))) (-79.30)

............GAGCTCCTTTCGTTCCAATGA........................................................................................................................................................... 1

........................................................AGCTGCTTGTTCATGGTTCCC............................................................................................................... 1

..................................................................................................................TTTGCATGACCAAGGAGCCGA..................................................... 21

..................................................................................................................TTTGCATGACCAAGGAGCCGAATC.................................................. 1

.......................................................................................................................................ATCAACTCCTTGCTGACCACTC............................... 1

.............................................................................................................................................................TTTGGATTGAAGGGAGCT............. 19

.............................................................................................................................................................TTTGGATTGAAGGGAGCTCTGCA........ 1

.............................................................................................................................................................TTTGGATTGAAGGGAGCTCTG.......... 25

.............................................................................................................................................................TTTGGATTGAAGGGAGCTC............ 1

..............................................................................................................................................................TTGGATTGAAGGGAGCTCTG.......... 2

>osa-MIR319a_MI0001098_Oryza_sativa_miR319a_stem-loop GSM278571

UGUGUAAGAAGAGAGCUCUCUUCAGUCCACUCUCAGAUGGCUGUAGGGUUUUAUUAGCUGCCGAAUCAUCCAUUCACCUACCAAGAAAGUUGCAGGAGUGUAUCUCUUGGUAGCGGACUGGAUGACGCGGGAGCUAAAAUUUAGCUCUGCGCCGUUUGUGGUUGGACUGAAGGGUGCUCCCUUGCUCAAGC

((.(((((..(.((((.((((((((((((..(.((((((((.((((((((...((((((.(((..(((((((.((..(((((((((....((((....))))..)))))))))..)).)))))))..))).)))))).....)))))))))))))))).)..)))))))))))).)))))))))).))... (-101.50)

.......................................................AGCTGCCGAATCATCCATTCA................................................................................................................... 1

....................................................................................................................ACTGGATGACGCGGGAGCTAA...................................................... 17

....................................................................................................................ACTGGATGACGCGGGAGCTAAA..................................................... 3

.................................................................................................................................................................TTGGACTGAAGGGTGCTCCC.......... 1

.................................................................................................................................................................TTGGACTGAAGGGTGCTCCCT......... 15

..................................................................................................................................................................TGGACTGAAGGGTGCTCCCT......... 1

>osa-MIR319b_MI0001099_Oryza_sativa_miR319b_stem-loop GSM278571

GAUGGAUGGAAGAGAGCGUCCUUCAGUCCACUCAUGGGCGGUGCUAGGGUCGAAUUAGCUGCCGACUCAUUCACCCACAUGCCAAGCAAGAAACGCUUGAGAUAGCGAAGCUUAGCAGAUGAGUGAAUGAAGCGGGAGGUAACGUUCCGAUCUCGCGCCGUCUUUGCUUGGACUGAAGGGUGCUCCCUCCUCCUCGA

((.(((.(((.(.((((..(((((((((((..((.(((((((((..(((((((((((.((.(((..((((((((.((..(((.((((......((((......))))..)))).)))..)).))))))))..))).)).))..))).)))))).))))))))).))..)))))))))))..))))).)))))))).. (-99.20)

............AGAGCGTCCTTCAGTCCACTCA................................................................................................................................................................... 8

............AGAGCGTCCTTCAGTCCACTC.................................................................................................................................................................... 5

........................................................AGCTGCCGACTCATTCACCCA........................................................................................................................ 2

.........................................................................................................................AGTGAATGAAGCGGGAGGTAA....................................................... 51

..........................................................................................................................GTGAATGAAGCGGGAGGTAA....................................................... 2

......................................................................................................................................................................CTTGGACTGAAGGGTGCTCCC.......... 1

......................................................................................................................................................................CTTGGACTGAAGGGTGCTCCCT......... 5

......................................................................................................................................................................CTTGGACTGAAGGGTGCT............. 1

.......................................................................................................................................................................TTGGACTGAAGGGTGCTCCC.......... 1

.......................................................................................................................................................................TTGGACTGAAGGGTGCTCCCT......... 15

........................................................................................................................................................................TGGACTGAAGGGTGCTCCCT......... 1

>osa-MIR396e_MI0001703_Oryza_sativa_miR396e_stem-loop GSM278571

GCGGGCAUGCUUUCCACAGGCUUUCUUGAACUGUGAACUCGUGGGGGUGUAUGUGCUCAUGUUGGGAUUGUGGUCGGUGGCCUCCAAUUCUCUGAAAAGAAAGCUGAAUUGUCGAGCUCCCCGUUCUGUCUUUGGUCGUCUCUACCUGUUGAUGGUUCAAGAAAGCCCAUGGAAACCAUGCCGC

((((.((((.((((((..((((((((((((((((.(((..((((((..(((((....))))..(((((...(..(((.((.((((((((((((....))).....))))))..)))..)))))..).)))))....)..))))))..))).))))))))))))))))..)))))).)))))))) (-74.90)

............TCCACAGGCTTTCTTGAA.......................................................................................................................................................... 6

............TCCACAGGCTTTCTTGAAC......................................................................................................................................................... 3

............TCCACAGGCTTTCTTGAACTG....................................................................................................................................................... 178

............TCCACAGGCTTTCTTGAACT........................................................................................................................................................ 1

..............CACAGGCTTTCTTGAACTG....................................................................................................................................................... 1

...............ACAGGCTTTCTTGAACTGTGA.................................................................................................................................................... 3

.........................................................CATGTTGGGATTGTGGTCGGTG......................................................................................................... 1

.......................................................................................................................................................ATGGTTCAAGAAAGCCCATGGAAA......... 1

..........................................................................................................................................................GTTCAAGAAAGCCCATGGAAA......... 2

>osa-MIR445a_MI0001709_Oryza_sativa_miR445a_stem-loop GSM278571

UCACAUUGAAUGUUUGACACUAAUUUGGAGUAUUAAACAUAGACUAAUAAAAAAACUAAUUUCAUAAAUGAAAGCUAAUCUGCGAGACGAAUUUUUUAAGCCUAAUUAAUCCAUAAUUAUGAAAAGUUUACUGUAGCAUCACAUUGUCAAAUCAUGGUGUAAUUAGACUCAAAAGAUUCGUCUCGCGAAUUAGUCCAAGGUUAUAGAAUAGGUUUUAUAAUUAGUGUAUGUUUAAUACUCUAAAUUAGUGUAUAAACAUCCGAUGUGA

((((((((.((((((((((((((((((((((((((((((((.((((((.(.((((((.(((..((((......((((((.(((((((((((((((((.((.(((((((..((((.(((......((....))...(((......)))..))).))))..))))))).)).))))))))))))))))).))))))......))))..))).)))))).).)))))).))))))))))))))))))))))))).))))))).)))))))) (-107.10)

....................TAATTTGGAGTATTAAACATAGA................................................................................................................................................................................................................................. 1

.................................................................................GCGAGACGAATTTTTTAAGCCTAA................................................................................................................................................................... 1

............................................................................................TTTTTAAGCCTAATTAATCCATAA........................................................................................................................................................ 1

..............................................................................................TTTAAGCCTAATTAATCCATAATTA..................................................................................................................................................... 1

..............................................................................................TTTAAGCCTAATTAATCCATAA........................................................................................................................................................ 1

.............................................................................................................................GTTTACTGTAGCATCACATTGTCA....................................................................................................................... 1

..............................................................................................................................TTTACTGTAGCATCACATTGTCA....................................................................................................................... 2

..............................................................................................................................TTTACTGTAGCATCACATTGT......................................................................................................................... 1

...............................................................................................................................TTACTGTAGCATCACATTGTCAAA..................................................................................................................... 1

..............................................................................................................................................................GTAATTAGACTCAAAAGATTCGTC...................................................................................... 1

..................................................................................................................................................................TTAGACTCAAAAGATTCGTCTCGC.................................................................................. 1

......................................................................................................................................................................ACTCAAAAGATTCGTCTCGCG................................................................................. 1

.........................................................................................................................................................................CAAAAGATTCGTCTCGCGA................................................................................ 1

..........................................................................................................................................................................AAAAGATTCGTCTCGCGA................................................................................ 1

...............................................................................................................................................................................ATTCGTCTCGCGAATTAGTCCAAGG.................................................................... 1

...............................................................................................................................................................................ATTCGTCTCGCGAATTAGTCCAAG..................................................................... 5

...............................................................................................................................................................................ATTCGTCTCGCGAATTAGTCCAA...................................................................... 1

................................................................................................................................................................................TTCGTCTCGCGAATTAGTCCAA...................................................................... 1

................................................................................................................................................................................TTCGTCTCGCGAATTAGTCCAAGG.................................................................... 3

.................................................................................................................................................................................TCGTCTCGCGAATTAGTCCAAG..................................................................... 1

.................................................................................................................................................................................TCGTCTCGCGAATTAGTCCAAGGT................................................................... 7

..................................................................................................................................................................................CGTCTCGCGAATTAGTCCAAGGTT.................................................................. 15

..................................................................................................................................................................................CGTCTCGCGAATTAGTCCAAGGT................................................................... 1

...................................................................................................................................................................................GTCTCGCGAATTAGTCCAAGGTTA................................................................. 2

............................................................................................................................................................................................ATTAGTCCAAGGTTATAGAATAGGT....................................................... 1

.............................................................................................................................................................................................TTAGTCCAAGGTTATAGAATAGGT....................................................... 1

..............................................................................................................................................................................................TAGTCCAAGGTTATAGAATAGGTT...................................................... 1

...............................................................................................................................................................................................AGTCCAAGGTTATAGAATAGGTTT..................................................... 1

>osa-MIR445c_MI0001711_Oryza_sativa_miR445c_stem-loop GSM278571

CCGUGUCACAUCGAAUGUUUGACGCUAAUUUGGAGUAUUAAACAUAGACUAAUAAAAAAACUAAUUUCAUAAAUGAAAGCUAAUCUGCGAGACGAAUUUUUUAAGCCUAAUUAAUCCAUAAUUAUGAAAAGUUUACUAAUCAUGGUGUAAUUAGGCUUAAAAGAUUCGUCUCGUGAAUUAGUCCAAGGUUAUAGAAUAGGUUUUAUAAUUAGUGUAUAUUUAAUACUCUAAAUUAGUGUAUAAACAUCCGAUGUGACAUA

..(((((((((((.((((((((((((((((((((((((((((.(((.((((((.(.((((((.(((..((((......((((((..(((((((((((((((((((((((((((..((((.(((((((.....))).)))).))))..)))))))))))))))))))))))))))..))))))......))))..))).)))))).).)))))).))).))))))))))))))))))))).))))))).))))))))))). (-119.70)

.........................TAATTTGGAGTATTAAACATAGA.................................................................................................................................................................................................................... 1

......................................................................................GCGAGACGAATTTTTTAAGCCTAA...................................................................................................................................................... 1

.................................................................................................TTTTTAAGCCTAATTAATCCATAA........................................................................................................................................... 1

...................................................................................................TTTAAGCCTAATTAATCCATAATTA........................................................................................................................................ 1

...................................................................................................TTTAAGCCTAATTAATCCATAA........................................................................................................................................... 1

.................................................................................................................................................TGTAATTAGGCTTAAAAGATTCGT........................................................................................... 1

..................................................................................................................................................GTAATTAGGCTTAAAAGATTCGTC.......................................................................................... 1

......................................................................................................................................................TTAGGCTTAAAAGATTCGTCTCG....................................................................................... 1

......................................................................................................................................................TTAGGCTTAAAAGATTCGTCTC........................................................................................ 3

.......................................................................................................................................................TAGGCTTAAAAGATTCGTCTCG....................................................................................... 2

.......................................................................................................................................................TAGGCTTAAAAGATTCGTCTCGTG..................................................................................... 1

.......................................................................................................................................................TAGGCTTAAAAGATTCGTCTC........................................................................................ 5

.........................................................................................................................................................GGCTTAAAAGATTCGTCTCGTGA.................................................................................... 1

...................................................................................................................................................................ATTCGTCTCGTGAATTAGTCCAA.......................................................................... 1

.......................................................................................................................................................................GTCTCGTGAATTAGTCCAAGGTTA..................................................................... 1

................................................................................................................................................................................ATTAGTCCAAGGTTATAGAATAGGT........................................................... 1

.................................................................................................................................................................................TTAGTCCAAGGTTATAGAATAGGT........................................................... 1

..................................................................................................................................................................................TAGTCCAAGGTTATAGAATAGGTT.......................................................... 1

...................................................................................................................................................................................AGTCCAAGGTTATAGAATAGGTTT......................................................... 1

>osa-MIR445e_MI0001713_Oryza_sativa_miR445e_stem-loop GSM278571

GUCCGUGUCACAUCGAAUGUUUGACGCUAAUUUGGAGUAUUAAACAUAGACUAAUAAAAAAACUAAUUUCAUAAAUGAAAGCUAAUCUGCGAGACGAAUUUUUUAAGCCUAAUUAAUCCAUAAUUAUGAAAAGUUUACUAAUCAUGGUGUAAUUAGACUCAAAAGAUUCGUCUCGCGAAUUAGUCCAAGGUUAUAGAAUAGGUUUUAUAAUUAGUGUAUGUUUAAUACUCUAAAUUAGUGUAUAAACAUCCGAUGUGACAGGGAC

((((.((((((((((.((((((((((((((((((((((((((((((((.((((((.(.((((((.(((..((((......((((((.(((((((((((((((((.((.(((((((..((((.(((((((.....))).)))).))))..))))))).)).))))))))))))))))).))))))......))))..))).)))))).).)))))).))))))))))))))))))))))))).))))))).)))))))))).)))) (-123.10)

...........................TAATTTGGAGTATTAAACATAGA....................................................................................................................................................................................................................... 1

........................................................................................GCGAGACGAATTTTTTAAGCCTAA......................................................................................................................................................... 1

...................................................................................................TTTTTAAGCCTAATTAATCCATAA.............................................................................................................................................. 1

.....................................................................................................TTTAAGCCTAATTAATCCATAATTA........................................................................................................................................... 1

.....................................................................................................TTTAAGCCTAATTAATCCATAA.............................................................................................................................................. 1

....................................................................................................................................................GTAATTAGACTCAAAAGATTCGTC............................................................................................. 1

........................................................................................................................................................TTAGACTCAAAAGATTCGTCTCGC......................................................................................... 1

............................................................................................................................................................ACTCAAAAGATTCGTCTCGCG........................................................................................ 1

...............................................................................................................................................................CAAAAGATTCGTCTCGCGA....................................................................................... 1

................................................................................................................................................................AAAAGATTCGTCTCGCGA....................................................................................... 1

.....................................................................................................................................................................ATTCGTCTCGCGAATTAGTCCAAGG........................................................................... 1

.....................................................................................................................................................................ATTCGTCTCGCGAATTAGTCCAAG............................................................................ 5

.....................................................................................................................................................................ATTCGTCTCGCGAATTAGTCCAA............................................................................. 1

......................................................................................................................................................................TTCGTCTCGCGAATTAGTCCAA............................................................................. 1

......................................................................................................................................................................TTCGTCTCGCGAATTAGTCCAAGG........................................................................... 3

.......................................................................................................................................................................TCGTCTCGCGAATTAGTCCAAG............................................................................ 1

.......................................................................................................................................................................TCGTCTCGCGAATTAGTCCAAGGT.......................................................................... 7

........................................................................................................................................................................CGTCTCGCGAATTAGTCCAAGGTT......................................................................... 15

........................................................................................................................................................................CGTCTCGCGAATTAGTCCAAGGT.......................................................................... 1

.........................................................................................................................................................................GTCTCGCGAATTAGTCCAAGGTTA........................................................................ 2

..................................................................................................................................................................................ATTAGTCCAAGGTTATAGAATAGGT.............................................................. 1

...................................................................................................................................................................................TTAGTCCAAGGTTATAGAATAGGT.............................................................. 1

....................................................................................................................................................................................TAGTCCAAGGTTATAGAATAGGTT............................................................. 1

.....................................................................................................................................................................................AGTCCAAGGTTATAGAATAGGTTT............................................................ 1

>osa-MIR445g_MI0001715_Oryza_sativa_miR445g_stem-loop GSM278571

UUUUAGUCCGUGUCACAUCGAAUGUUUGACGCUAAUUUGGAGUAUUAAACAUAGACUAAUAAAAAAACUAAUUUCAUAAAUGAAAGCUAAUCUGCGAGACGAAUUUUUUAAGCCUAAUUAAUCCAUAAUUAUGAAAAGUUUACUAAUCAUGGUGUAAUUAAACUCAAAAGAUUCGUCUUGCGAAUUAGUCCAAGGUUAUAGAAUAUGUUUUAUAAUUAGUGUAUGUUUAAUAUUCUAAAUUAGUGUAUAAACAUCCGAUGUGACAGGGACUUAAA

....(((((.((((((((((.((((((((((((((((((((((((((((((((.((((((.(.(((((..(((..((((......((((((.(((((((((((((((((.((..((((((..((((.(((((((.....))).)))).))))..))))))..)).))))))))))))))))).))))))......))))..)))..))))).).)))))).))))))))))))))))))))))))).))))))).)))))))))).))))).... (-114.60)

..TTAGTCCGTGTCACATCGAATGTT......................................................................................................................................................................................................................................................... 2

...TAGTCCGTGTCACATCGAATGTTT........................................................................................................................................................................................................................................................ 1

................................TAATTTGGAGTATTAAACATAGA............................................................................................................................................................................................................................ 1

.............................................................................................GCGAGACGAATTTTTTAAGCCTAA.............................................................................................................................................................. 1

........................................................................................................TTTTTAAGCCTAATTAATCCATAA................................................................................................................................................... 1

..........................................................................................................TTTAAGCCTAATTAATCCATAATTA................................................................................................................................................ 1
[truncated: 176,168 more chars]
