# Supplementary material for: Multiple distinct small RNAs originate from the same microRNA precursors
Source: Genome Biol. 2010 Aug 9;11(8):R81. doi: 10.1186/gb-2010-11-8-r81 (PMC2945783; doi:10.1186/gb-2010-11-8-r81)
Supplement: Additional file 4 — Supplemental File S3. This is a file for sequencing reads mapped and aligned to miRNA precursors that can produce miRNA-sibling small RNAs (msRNAs) in moss (ppt). The sequencing data were obtained from GEO; see Materials and methods for details. [file gb-2010-11-8-r81-S4.DOCX]

Zhang, et al., Multiple distinct small RNAs originate from the same microRNA precursors

Supplemental File 3 - Sequencing reads mapped and aligned to miRNA precursors that can produce

miRNA-like RNAs in *Physcomitralla patens.*

>ppt-MIR319a_MI0003496_Physcomitrella_patens_miR319a_stem-loop GSM313212

GUGGAGCUCCGUUUCGGUCCAAUAGUGGCUGCGACGGAAGGUGGUCCCGCUGCCGAAUCACACGUCCGGGUUGCUUAUCGGGGCAGGGCCCCGAUACGGUAUCCGAACGUUUGUCCCGGGAACUGGUCGACCUUCCGCCCGGCGUCUCUUGGACUGAAGGGAGCUCCAC

((((((((((.(((((((((((.((..((((.(.((((((((.(.((.(.(.(((...((.((((.(((((.((((((((((((...))))))))).)))))))).)))).))...))).).).)).).)))))))).)))))..)).))))))))))))))))))))) (-98.80)

.TGGAGCTCCGTTTCGGTCCAAT.................................................................................................................................................. 2

.TGGAGCTCCGTTTCGGTCCAA................................................................................................................................................... 1

.TGGAGCTCCGTTTCGGTCCAATA................................................................................................................................................. 1

..GGAGCTCCGTTTCGGTCCA.................................................................................................................................................... 1

..GGAGCTCCGTTTCGGTCCAA................................................................................................................................................... 1

..GGAGCTCCGTTTCGGTCCAAT.................................................................................................................................................. 4

..GGAGCTCCGTTTCGGTCCAATA................................................................................................................................................. 18

...GAGCTCCGTTTCGGTCCAATAG................................................................................................................................................ 557

...GAGCTCCGTTTCGGTCCAATAGT............................................................................................................................................... 34

...GAGCTCCGTTTCGGTCCAAT.................................................................................................................................................. 65

...GAGCTCCGTTTCGGTCCAA................................................................................................................................................... 17

...GAGCTCCGTTTCGGTCCAATA................................................................................................................................................. 544

....AGCTCCGTTTCGGTCCAATAG................................................................................................................................................ 999

....AGCTCCGTTTCGGTCCAAT.................................................................................................................................................. 213

....AGCTCCGTTTCGGTCCAATA................................................................................................................................................. 834

....AGCTCCGTTTCGGTCCAATAGT............................................................................................................................................... 94

.....GCTCCGTTTCGGTCCAATAG................................................................................................................................................ 10

.....GCTCCGTTTCGGTCCAATAGT............................................................................................................................................... 1

.....GCTCCGTTTCGGTCCAATA................................................................................................................................................. 5

.............TCGGTCCAATAGTGGCTGCGA....................................................................................................................................... 3

........................GTGGCTGCGACGGAAGGTGGTC........................................................................................................................... 1

.........................TGGCTGCGACGGAAGGTGGTC........................................................................................................................... 94

.........................TGGCTGCGACGGAAGGTGGTCC.......................................................................................................................... 413

.........................TGGCTGCGACGGAAGGTGGT............................................................................................................................ 6

.........................TGGCTGCGACGGAAGGTGGTCCC......................................................................................................................... 20

..........................GGCTGCGACGGAAGGTGGTCC.......................................................................................................................... 1

............................CTGCGACGGAAGGTGGTCC.......................................................................................................................... 1

..................................CGGAAGGTGGTCCCGCTGCCG.................................................................................................................. 2

.........................................................................................................AACGTTTGTCCCGGGAACTGG........................................... 1

..............................................................................................................................TCGACCTTCCGCCCGGCGT........................ 13

..............................................................................................................................TCGACCTTCCGCCCGGCGTC....................... 33

..............................................................................................................................TCGACCTTCCGCCCGGCGTCT...................... 64

...............................................................................................................................CGACCTTCCGCCCGGCGTCT...................... 2

................................................................................................................................GACCTTCCGCCCGGCGTCT...................... 2

.............................................................................................................................................GCGTCTCTTGGACTGAAGGGAGCT.... 1

................................................................................................................................................TCTCTTGGACTGAAGGGAGCTCC.. 1

................................................................................................................................................TCTCTTGGACTGAAGGGAGCT.... 13

................................................................................................................................................TCTCTTGGACTGAAGGGAGCTC... 1

..................................................................................................................................................TCTTGGACTGAAGGGAGCTCC.. 7

..................................................................................................................................................TCTTGGACTGAAGGGAGCTC... 1

...................................................................................................................................................CTTGGACTGAAGGGAGCTC... 24

...................................................................................................................................................CTTGGACTGAAGGGAGCTCC.. 442

...................................................................................................................................................CTTGGACTGAAGGGAGCTCCAC 10

...................................................................................................................................................CTTGGACTGAAGGGAGCTCCA. 403

....................................................................................................................................................TTGGACTGAAGGGAGCTCC.. 10

....................................................................................................................................................TTGGACTGAAGGGAGCTCCA. 24

....................................................................................................................................................TTGGACTGAAGGGAGCTCCAC 23

.....................................................................................................................................................TGGACTGAAGGGAGCTCCA. 3

.....................................................................................................................................................TGGACTGAAGGGAGCTCCAC 3

>ppt-MIR896_MI0005680_Physcomitrella_patens_miR896_stem-loop GSM313212

CCCACCCUGAGCGGGGGGGUCUGAUUGUGGUCGGUCGAGGGGCGCUGACCGUGUGGCCCAAUGGAUAAGGCGCUUGCCUACGGAGCAAGAGAUUCUGGGUUCGAUCCCCAGCAUGGUCGUGGGUCGCCGGAAUUGUAGAUUGUGGCGCGUGUCAAUUUGGCCGAGUGGUUAAGGCGGCAGACUCGAAAUCUGCUGGGGUUUCCCCGCGCAGGUUCAAAUCCUGCA

.....((((.((((((((..((.(..(..(....(((((.(.(((((((((..(((((.(((.((((..(((((((((....).)))...((((((((...((((((((.....))....)))))))))))))).........))))).)))).))).)))))..)))))..)))).)...)))))...)..)).))..)))))))).))))............. (-94.10)

............GGGGGGGTCTGATTGTGGTCGGT.............................................................................................................................................................................................. 1

.........................TGTGGTCGGTCGAGGGGCGCT................................................................................................................................................................................... 1

..............................................GACCGTGTGGCCCAATGGA................................................................................................................................................................ 36

..............................................GACCGTGTGGCCCAATGGATA.............................................................................................................................................................. 25

..............................................GACCGTGTGGCCCAATGGATAA............................................................................................................................................................. 42

..............................................GACCGTGTGGCCCAATGGAT............................................................................................................................................................... 24

..............................................GACCGTGTGGCCCAATGGATAAGGC.......................................................................................................................................................... 19

..............................................GACCGTGTGGCCCAATGGATAAGG........................................................................................................................................................... 14

..............................................GACCGTGTGGCCCAATGGATAAG............................................................................................................................................................ 10

...............................................ACCGTGTGGCCCAATGGATAAGG........................................................................................................................................................... 1

.....................................................TGGCCCAATGGATAAGGCGCT....................................................................................................................................................... 1

..........................................................CAATGGATAAGGCGCTTGCCTA................................................................................................................................................. 1

...........................................................AATGGATAAGGCGCTTGCCT.................................................................................................................................................. 1

..................................................................................GAGCAAGAGATTCTGGGTTCG.......................................................................................................................... 1

...................................................................................AGCAAGAGATTCTGGGTTC........................................................................................................................... 1

....................................................................................GCAAGAGATTCTGGGTTCGAT........................................................................................................................ 1

....................................................................................GCAAGAGATTCTGGGTTCG.......................................................................................................................... 1

.....................................................................................CAAGAGATTCTGGGTTCGATCCCCA................................................................................................................... 1

.....................................................................................CAAGAGATTCTGGGTTCGAT........................................................................................................................ 1

.........................................................................................AGATTCTGGGTTCGATCCC..................................................................................................................... 1

...........................................................................................ATTCTGGGTTCGATCCCCAGCA................................................................................................................ 1

............................................................................................TTCTGGGTTCGATCCCCAGCA................................................................................................................ 1

.............................................................................................TCTGGGTTCGATCCCCAGCA................................................................................................................ 1

...................................................................................................TTCGATCCCCAGCATGGTCGTG........................................................................................................ 1

.......................................................................................................................TGGGTCGCCGGAATTGTAGATTG................................................................................... 1

............................................................................................................................CGCCGGAATTGTAGATTGTGGCGC............................................................................. 2

.............................................................................................................................GCCGGAATTGTAGATTGTGGCGCG............................................................................ 1

.............................................................................................................................GCCGGAATTGTAGATTGTGGC............................................................................... 2

.............................................................................................................................GCCGGAATTGTAGATTGTGGCGC............................................................................. 9

.............................................................................................................................GCCGGAATTGTAGATTGTGGCG.............................................................................. 23

...................................................................................................................................ATTGTAGATTGTGGCGCGT........................................................................... 1

......................................................................................................................................................GTCAATTTGGCCGAGTGGTTAAGGC.................................................. 12

......................................................................................................................................................GTCAATTTGGCCGAGTGGTT....................................................... 80

......................................................................................................................................................GTCAATTTGGCCGAGTGGT........................................................ 105

......................................................................................................................................................GTCAATTTGGCCGAGTGGTTAAG.................................................... 144

......................................................................................................................................................GTCAATTTGGCCGAGTGGTTAAGG................................................... 64

......................................................................................................................................................GTCAATTTGGCCGAGTGGTTA...................................................... 123

......................................................................................................................................................GTCAATTTGGCCGAGTGGTTAA..................................................... 187

.......................................................................................................................................................TCAATTTGGCCGAGTGGTTAA..................................................... 1

.........................................................................................................................................................AATTTGGCCGAGTGGTTAAG.................................................... 4

.........................................................................................................................................................AATTTGGCCGAGTGGTTAA..................................................... 7

.........................................................................................................................................................AATTTGGCCGAGTGGTTAAGG................................................... 1

...........................................................................................................................................................TTTGGCCGAGTGGTTAAGG................................................... 2

...........................................................................................................................................................TTTGGCCGAGTGGTTAAGGC.................................................. 1

............................................................................................................................................................TTGGCCGAGTGGTTAAGGCG................................................. 1

......................................................................................................................................................................................TCGAAATCTGCTGGGGTTTC....................... 1

..........................................................................................................................................................................................AATCTGCTGGGGTTTCCCCGCGCA............... 1

..........................................................................................................................................................................................AATCTGCTGGGGTTTCCCCGCG................. 1

...........................................................................................................................................................................................ATCTGCTGGGGTTTCCCCGCG................. 1

...........................................................................................................................................................................................ATCTGCTGGGGTTTCCCCGCGCA............... 1

...........................................................................................................................................................................................ATCTGCTGGGGTTTCCCCGCGC................ 1

............................................................................................................................................................................................TCTGCTGGGGTTTCCCCGCGCAG.............. 1

............................................................................................................................................................................................TCTGCTGGGGTTTCCCCGCGCAGGT............ 1

............................................................................................................................................................................................TCTGCTGGGGTTTCCCCGCGCA............... 1

.............................................................................................................................................................................................CTGCTGGGGTTTCCCCGCGCAGGT............ 2

.............................................................................................................................................................................................CTGCTGGGGTTTCCCCGCGCAGG............. 1

..............................................................................................................................................................................................TGCTGGGGTTTCCCCGCGCAGGTT........... 1

..............................................................................................................................................................................................TGCTGGGGTTTCCCCGCGCAGGTTCA......... 1

..............................................................................................................................................................................................TGCTGGGGTTTCCCCGCGCAGG............. 1

................................................................................................................................................................................................CTGGGGTTTCCCCGCGCAGG............. 1

................................................................................................................................................................................................CTGGGGTTTCCCCGCGCAGGTTCAAA....... 1

.................................................................................................................................................................................................TGGGGTTTCCCCGCGCAGGTTC.......... 1

.............................................................................................................................................................................................................GCGCAGGTTCAAATCCTGCA 1

>ppt-MIR319b_MI0003497_Physcomitrella_patens_miR319b_stem-loop GSM313212

GAGCUCUUUUCAGUCCAGUAGCAGCUAAUGUCGAAGGUUGUACCGCUGCCGACUCAAACUUCCGGCUUCCAUAUCACGACGCGUGAUAUGGAAUCCGAAGGUCUGAUCCGGGAGCUGAUCGAUCUCCAGGUUAGCAUCUCUUGGACUGAAGGGAGCUCCU

((((((((((((((((((.((..((((((.(.((.(((((....(((.(((..(((.((((.(((.((((((((((((...)))))))))))).))).)))).)))..))).)))....))))))).).))))))..)).)))))))))))))))))).. (-83.70)

GAGCTCTTTTCAGTCCAGT............................................................................................................................................. 1

GAGCTCTTTTCAGTCCAGTAG........................................................................................................................................... 1

GAGCTCTTTTCAGTCCAGTA............................................................................................................................................ 7

.AGCTCTTTTCAGTCCAGTA............................................................................................................................................ 27

.AGCTCTTTTCAGTCCAGTAG........................................................................................................................................... 9

.....................CAGCTAATGTCGAAGGTTGTAC..................................................................................................................... 1

...........................................CGCTGCCGACTCAAACTTC.................................................................................................. 1

...........................................CGCTGCCGACTCAAACTTCCG................................................................................................ 2

.....................................................................CATATCACGACGCGTGATATGGA.................................................................... 1

.................................................................................................AAGGTCTGATCCGGGAGCTGA.......................................... 10

..................................................................................................................................TTAGCATCTCTTGGACTGAA.......... 6

..................................................................................................................................TTAGCATCTCTTGGACTGAAG......... 46

.......................................................................................................................................ATCTCTTGGACTGAAGGGAG..... 1

.......................................................................................................................................ATCTCTTGGACTGAAGGGA...... 1

........................................................................................................................................TCTCTTGGACTGAAGGGAGCTCC. 1

........................................................................................................................................TCTCTTGGACTGAAGGGAGCT... 13

........................................................................................................................................TCTCTTGGACTGAAGGGAGCTC.. 1

..........................................................................................................................................TCTTGGACTGAAGGGAGCTCC. 7

..........................................................................................................................................TCTTGGACTGAAGGGAGCTC.. 1

...........................................................................................................................................CTTGGACTGAAGGGAGCTCCT 19

...........................................................................................................................................CTTGGACTGAAGGGAGCTC.. 24

...........................................................................................................................................CTTGGACTGAAGGGAGCTCC. 442

............................................................................................................................................TTGGACTGAAGGGAGCTCC. 10

............................................................................................................................................TTGGACTGAAGGGAGCTCCT 1

>ppt-MIR319c_MI0003498_Physcomitrella_patens_miR319c_stem-loop GSM313212

GGAGATTCTTTCAGTCCAGTC TGTGAATGATGCGGGAGATAT TTTGGACTGAAGGGAGCTC

UAACCUCACUGGCUGUGGGAGCUUCCUUCGGUUCAAUAGUGGCUGAUAUGAGGUUGCACUGCUGCCGACUCAAACUUCCGGCUUCCCUCUCUUAGAAUGGCAGGGAAUCCGAAUGUCUGAUGCGGGAGCUGAGCGGUCUUCAACUCAGCUUCUCUUGGACUGAAGGGAGCUCCCAUGUCUUUGUGGUUA

(((((.((..(((.((((((((((((((((((((((.((.((((((..(((((((((.(.(((.(((..(((.((...(((.((((((.((.......)).)))))).)))...)).)))..))).))).).)))).)))))..)))))).)).)))))))))))))))))))))))))..)).))))) (-99.10)

.................GGAGCTTCCTTCGGTTCAAT........................................................................................................................................................ 1

.................GGAGCTTCCTTCGGTTCAATA....................................................................................................................................................... 1

..................GAGCTTCCTTCGGTTCAATAG...................................................................................................................................................... 755

..................GAGCTTCCTTCGGTTCAATA....................................................................................................................................................... 875

..................GAGCTTCCTTCGGTTCAATAGT..................................................................................................................................................... 65

..................GAGCTTCCTTCGGTTCAAT........................................................................................................................................................ 385

...................AGCTTCCTTCGGTTCAATA....................................................................................................................................................... 48

...................AGCTTCCTTCGGTTCAATAG...................................................................................................................................................... 101

...................AGCTTCCTTCGGTTCAATAGT..................................................................................................................................................... 6

....................GCTTCCTTCGGTTCAATAG...................................................................................................................................................... 1

.........................CTTCGGTTCAATAGTGGCTGA............................................................................................................................................... 5

.......................................TGGCTGATATGAGGTTGCA................................................................................................................................... 9

.......................................TGGCTGATATGAGGTTGCAC.................................................................................................................................. 196

...........................................................TGCTGCCGACTCAAACTTCCG............................................................................................................. 2

...........................................................TGCTGCCGACTCAAACTTCC.............................................................................................................. 3

................................................................................GCTTCCCTCTCTTAGAATGGCAGG..................................................................................... 1

................................................................................GCTTCCCTCTCTTAGAATGGCA....................................................................................... 5

................................................................................GCTTCCCTCTCTTAGAATGGCAGGGA................................................................................... 7

.................................................................................CTTCCCTCTCTTAGAATGGCAGGG.................................................................................... 1

.................................................................................CTTCCCTCTCTTAGAATGGCAGGGA................................................................................... 1

..................................................................................TTCCCTCTCTTAGAATGGCAGGGAAT................................................................................. 1

..................................................................................TTCCCTCTCTTAGAATGGCAGGGA................................................................................... 2

...................................................................................TCCCTCTCTTAGAATGGCAGGGA................................................................................... 1

....................................................................................CCCTCTCTTAGAATGGCAGGGA................................................................................... 1

.....................................................................................CCTCTCTTAGAATGGCAGGGAATCC............................................................................... 1

.......................................................................................TCTCTTAGAATGGCAGGGAATCC............................................................................... 1

...............................................................................................................AATGTCTGATGCGGGAGCTGA......................................................... 13

....................................................................................................................CTGATGCGGGAGCTGAGCGGT.................................................... 1

....................................................................................................................................GCGGTCTTCAACTCAGCTT...................................... 5

....................................................................................................................................GCGGTCTTCAACTCAGCTTCTCT.................................. 1

....................................................................................................................................GCGGTCTTCAACTCAGCTTC..................................... 11

....................................................................................................................................GCGGTCTTCAACTCAGCTTCT.................................... 58

...................................................................................................................................................GCTTCTCTTGGACTGAAGGGAGCTC................. 1

...................................................................................................................................................GCTTCTCTTGGACTGAAGGGAGCTCC................ 1

......................................................................................................................................................TCTCTTGGACTGAAGGGAGCTCC................ 1

......................................................................................................................................................TCTCTTGGACTGAAGGGAGCT.................. 13

......................................................................................................................................................TCTCTTGGACTGAAGGGAGCTC................. 1

........................................................................................................................................................TCTTGGACTGAAGGGAGCTCC................ 7

........................................................................................................................................................TCTTGGACTGAAGGGAGCTC................. 1

.........................................................................................................................................................CTTGGACTGAAGGGAGCTCCC............... 1171

.........................................................................................................................................................CTTGGACTGAAGGGAGCTCCCA.............. 10

.........................................................................................................................................................CTTGGACTGAAGGGAGCTC................. 24

.........................................................................................................................................................CTTGGACTGAAGGGAGCTCC................ 442

.........................................................................................................................................................CTTGGACTGAAGGGAGCTCCCAT............. 1

..........................................................................................................................................................TTGGACTGAAGGGAGCTCC................ 10

..........................................................................................................................................................TTGGACTGAAGGGAGCTCCCA.............. 1

..........................................................................................................................................................TTGGACTGAAGGGAGCTCCC............... 31

...........................................................................................................................................................TGGACTGAAGGGAGCTCCC............... 6

...........................................................................................................................................................TGGACTGAAGGGAGCTCCCA.............. 2

>ppt-MIR2083_MI0010537_Physcomitrella_patens_miR2083_stem-loop GSM313212

AAAAGUGUGUGUAUGCGGAAGUUGGAGAAAACCCAGACUUCAUUGCUCGAUGAAGCAUGUAGCUUCGCAGUCGUACUUCUUGCACUCCUCCAUCUCUCGCAUCUCUUGCGCAUUGUGGUUCUCCAACACCUGCACAGCGGCGGUCUUGUCCGGAUUAGGGAUCGUCUACACCAGAUAAGCCACGGCGUUUUUCUCGGAGAAAAAUACCAUCCUUUGGUUCAACAACGGUUGCUGCGUUUUCCACGUUCGGAUACAACCCUGCCCAGUUGCUGUGAGAGAUUGACGAGUGCAAGAAGACCCUCUUUCCAUGUUCCAGACGCA

....((((.((((((.(((((..((.(((....((..(((((((....)))))))..))....))).........((((((((((((.((.((((((((((.(..(((.(((.(((((....)).)))..)))...((((.(((..(((((((((...(((.((.......(((...((((..((..((((((.....))))))..)).....(((......))).)))).)))))...)))..)))))))))..))))))).)))..).)))))))))).)).)))))))))))).))..)))))))))...)).)))). (-109.51)

............................................................................TTCTTGCACTCCTCCATCTCTC............................................................................................................................................................................................................................... 2

............................................................................TTCTTGCACTCCTCCATCT.................................................................................................................................................................................................................................. 2

............................................................................TTCTTGCACTCCTCCATCTC................................................................................................................................................................................................................................. 2

............................................................................TTCTTGCACTCCTCCATCTCT................................................................................................................................................................................................................................ 97

.............................................................................TCTTGCACTCCTCCATCTCT................................................................................................................................................................................................................................ 2

..............................................................................CTTGCACTCCTCCATCTCT................................................................................................................................................................................................................................ 1

................................................................................................TCGCATCTCTTGCGCATTGTG............................................................................................................................................................................................................ 1

........................................................................................................................CTCCAACACCTGCACAGCGGC.................................................................................................................................................................................... 1

...................................................................................................................................................................................................................................GTTGCTGCGTTTTCCACGTTCGGA...................................................................... 1

..............................................................................................................................................................................................................................................................................TGTGAGAGATTGACGAGTGCA.............................. 11

...................................................................................................................................................................................................................................................................................GAGATTGACGAGTGCAAGAAG......................... 3

....................................................................................................................................................................................................................................................................................AGATTGACGAGTGCAAGAAG......................... 5

....................................................................................................................................................................................................................................................................................AGATTGACGAGTGCAAGAAGA........................ 148

....................................................................................................................................................................................................................................................................................AGATTGACGAGTGCAAGAA.......................... 1

>ppt-MIR538c_MI0003512_Physcomitrella_patens_miR538c_stem-loop GSM313212

UUACAAAGAGUUCUUGAGUCUCCAUGCUUCUCUGACGUUGCAUGGAGUCUAUGUCUGGACCUUCUCCAUUUCCUCUUUCAAGAGAAUGUGAGCAUUGAAGGAUUCGGACAUAGCCUUCAUGCAUGCUAGAGUAUCAUGGAGGCCCGUGAACUACUUGUAA

.(((((..(((((.((.(((((((((...(((((.(((.((((((((.((((((((((((((((.((((...((((....))))...))).)....))))).))))))))))).))))))))))).)))))...))))))))).)).)))))..))))). (-73.90)

................AGTCTCCATGCTTCTCTGACG........................................................................................................................... 10

................AGTCTCCATGCTTCTCTGAC............................................................................................................................ 1

.....................................TTGCATGGAGTCTATGTCTGG...................................................................................................... 24

.....................................TTGCATGGAGTCTATGTCTG....................................................................................................... 2

.....................................TTGCATGGAGTCTATGTCTGGACC................................................................................................... 1

.....................................TTGCATGGAGTCTATGTCT........................................................................................................ 2

.....................................TTGCATGGAGTCTATGTCTGGAC.................................................................................................... 12

.....................................TTGCATGGAGTCTATGTCTGGA..................................................................................................... 865

......................................TGCATGGAGTCTATGTCTGGA..................................................................................................... 95

......................................TGCATGGAGTCTATGTCTGGAC.................................................................................................... 7

......................................TGCATGGAGTCTATGTCTGG...................................................................................................... 11

.......................................GCATGGAGTCTATGTCTGGA..................................................................................................... 1

........................................CATGGAGTCTATGTCTGGA..................................................................................................... 1

.......................................................................................................TCGGACATAGCCTTCATGCATG................................... 1

........................................................................................................CGGACATAGCCTTCATGCATGC.................................. 2

........................................................................................................CGGACATAGCCTTCATGCAT.................................... 10

........................................................................................................CGGACATAGCCTTCATGCATG................................... 48

........................................................................................................CGGACATAGCCTTCATGCA..................................... 2

.........................................................................................................GGACATAGCCTTCATGCATG................................... 1

.............................................................................................................................CTAGAGTATCATGGAGGCCC............... 4

.............................................................................................................................CTAGAGTATCATGGAGGCCCGT............. 4

.............................................................................................................................CTAGAGTATCATGGAGGCCCG.............. 122

.............................................................................................................................CTAGAGTATCATGGAGGCC................ 1

..............................................................................................................................TAGAGTATCATGGAGGCCCG.............. 4

>ppt-MIR1219a_MI0004718_Physcomitrella_patens_miR1219a_stem-loop GSM313212

UGAAGUGUGGACGAUGGAGAGUCAGCCUCUUCCUGCCUCUCACUAGCUUCAUCCCUUCCUCCCUAAAUUUUAGUCUGGGAGGGAAGGAGCUAUUGGUGGUCAGGAAUAGCGCACCCUUCAUUUAUCCACACUUCA

.((((((((((.(((((((.((..((((.((((((((.(.((.(((((((.((((((((...(((.....)))...)))))))).))))))).))).)).)))))).)).)))).)))))))..)))))))))). (-67.20)

.....TGTGGACGATGGAGAGTCAGC............................................................................................................. 2

.....TGTGGACGATGGAGAGTCA............................................................................................................... 7

.....TGTGGACGATGGAGAGTCAGCCT........................................................................................................... 48

.....TGTGGACGATGGAGAGTCAG.............................................................................................................. 3

.....TGTGGACGATGGAGAGTCAGCC............................................................................................................ 38

......GTGGACGATGGAGAGTCAGCC............................................................................................................ 1

.......TGGACGATGGAGAGTCAGCCT........................................................................................................... 1

.......TGGACGATGGAGAGTCAGCC............................................................................................................ 1

...........................TCTTCCTGCCTCTCACTAGCT....................................................................................... 1

............................CTTCCTGCCTCTCACTAGCTTC..................................................................................... 1

............................CTTCCTGCCTCTCACTAGCTT...................................................................................... 47

............................CTTCCTGCCTCTCACTAGCT....................................................................................... 3

.............................TTCCTGCCTCTCACTAGCTT...................................................................................... 2

..........................................................CTCCCTAAATTTTAGTCTGGGA....................................................... 1

........................................................................................GCTATTGGTGGTCAGGAATAG.......................... 7

........................................................................................GCTATTGGTGGTCAGGAATA........................... 6

.............................................................................................................CGCACCCTTCATTTATCCACAC.... 2

.............................................................................................................CGCACCCTTCATTTATCCACACT... 5

>ppt-MIR319e_MI0005665_Physcomitrella_patens_miR319e_stem-loop GSM313212

CUAGCCGUGGGAGCUCCUUCCGGUUCAAUAGUGGCUGAUGUGAGGUUGCACUGCUGCCGAUUCAAACUUCCGGCUUCCCUUUCUUAACACGACAGGGAAUCCGAAUGUCUGAUGCGGGAGCUGUGCGGUCUUCAACUUUAGCGCCUCUUGGACUGAAGGGAGCUCCCAAGUCUU

.......((((((((((((.((((((((.((..(((((..(((((((((((.(((.(((..(((.((...(((.((((((.((.......)).)))))).)))...)).)))..))).))).)))))).)))))...)))))..)).)))))))).))))))))))))...... (-86.10)

.........GGAGCTCCTTCCGGTTCAATA................................................................................................................................................ 1

..........GAGCTCCTTCCGGTTCAAT................................................................................................................................................. 12

..........GAGCTCCTTCCGGTTCAATA................................................................................................................................................ 39

..........GAGCTCCTTCCGGTTCAATAGT.............................................................................................................................................. 4

..........GAGCTCCTTCCGGTTCAATAG............................................................................................................................................... 12

...........AGCTCCTTCCGGTTCAATAG............................................................................................................................................... 1

...............................TGGCTGATGTGAGGTTGCACT.......................................................................................................................... 1

........................................................................GCTTCCCTTTCTTAACACGACAGGG............................................................................. 1

.............................................................................CCTTTCTTAACACGACAGGGAAT.......................................................................... 1

...........................................................................................................................TGCGGTCTTCAACTTTAGCGCC............................. 1

............................................................................................................................GCGGTCTTCAACTTTAGCGCCT............................ 1

............................................................................................................................GCGGTCTTCAACTTTAGCGCC............................. 1

.................................................................................................................................................TCTTGGACTGAAGGGAGCTCC........ 7

.................................................................................................................................................TCTTGGACTGAAGGGAGCTC......... 1

..................................................................................................................................................CTTGGACTGAAGGGAGCTCCC....... 1171

..................................................................................................................................................CTTGGACTGAAGGGAGCTCCCA...... 10

..................................................................................................................................................CTTGGACTGAAGGGAGCTC......... 24

..................................................................................................................................................CTTGGACTGAAGGGAGCTCC........ 442

...................................................................................................................................................TTGGACTGAAGGGAGCTCC........ 10

...................................................................................................................................................TTGGACTGAAGGGAGCTCCCA...... 1

...................................................................................................................................................TTGGACTGAAGGGAGCTCCC....... 31

...................................................................................................................................................TTGGACTGAAGGGAGCTCCCAA..... 1

....................................................................................................................................................TGGACTGAAGGGAGCTCCC....... 6

....................................................................................................................................................TGGACTGAAGGGAGCTCCCA...... 2

>ppt-MIR904b_MI0005693_Physcomitrella_patens_miR904b_stem-loop GSM313212

CUUUGGAGCGAUGUGUGUCAUGAUGGCGGUAUUUUGUGAUGCUAAUACGGUCUUGUCAAUGUUUAGGGGCAAAGGUGCAACUAUGCAACAUCCCUGGUGCGAGCUCUUGAUCGCGUAAGUGUUGCUAGUGCCCUCCACCCCUAAAUGUUGGCAAGACCUAAUUGGCGCACAACAUUGGGACCGACGCUGGAUCAGCAGAAGUCAAAAGACACAAUUGUGA

.......(((((.((((((.((((..((((...(((((.(((((((..(((((((((((((((((((((...(((.(((.(((.((((((...((..(((((........)))))..)))))))))))))))))...)))))))))))))))))))))..)))))))))))).......))))..((((...))))....))))...))))))))))).. (-93.30)

................................................GGTCTTGTCAATGTTTAGGGG....................................................................................................................................................... 1

..................................................TCTTGTCAATGTTTAGGGGCAA.................................................................................................................................................... 442

..................................................TCTTGTCAATGTTTAGGGGCAAAG.................................................................................................................................................. 9

..................................................TCTTGTCAATGTTTAGGGG....................................................................................................................................................... 7

..................................................TCTTGTCAATGTTTAGGGGCAAA................................................................................................................................................... 9

..................................................TCTTGTCAATGTTTAGGGGC...................................................................................................................................................... 10

..................................................TCTTGTCAATGTTTAGGGGCA..................................................................................................................................................... 6743

...................................................CTTGTCAATGTTTAGGGGCAA.................................................................................................................................................... 121

...................................................CTTGTCAATGTTTAGGGGC...................................................................................................................................................... 6

...................................................CTTGTCAATGTTTAGGGGCAAAG.................................................................................................................................................. 4

...................................................CTTGTCAATGTTTAGGGGCA..................................................................................................................................................... 2602

...................................................CTTGTCAATGTTTAGGGGCAAA................................................................................................................................................... 1

....................................................TTGTCAATGTTTAGGGGCA..................................................................................................................................................... 75

....................................................TTGTCAATGTTTAGGGGCAA.................................................................................................................................................... 28

.....................................................TGTCAATGTTTAGGGGCAA.................................................................................................................................................... 2

.........................................................................GGTGCAACTATGCAACATCCCTGG........................................................................................................................... 1

......................................................................................................................................CCACCCCTAAATGTTGGCAAG................................................................. 1

........................................................................................................................................ACCCCTAAATGTTGGCAAGA................................................................ 2

........................................................................................................................................ACCCCTAAATGTTGGCAAGAC............................................................... 9

........................................................................................................................................ACCCCTAAATGTTGGCAAGACC.............................................................. 1

.........................................................................................................................................CCCCTAAATGTTGGCAAGAC............................................................... 11

.........................................................................................................................................CCCCTAAATGTTGGCAAGACC.............................................................. 163

.........................................................................................................................................CCCCTAAATGTTGGCAAGA................................................................ 3

..........................................................................................................................................CCCTAAATGTTGGCAAGACC.............................................................. 9

..........................................................................................................................................CCCTAAATGTTGGCAAGAC............................................................... 1

...........................................................................................................................................CCTAAATGTTGGCAAGACC.............................................................. 1

...........................................................................................................................................CCTAAATGTTGGCAAGACCTA............................................................ 1

............................................................................................................................................CTAAATGTTGGCAAGACCTAA........................................................... 1

>ppt-MIR1072_MI0006038_Physcomitrella_patens_miR1072_stem-loop GSM313212

AAUUUGUUCUAAUCUUAUGAGAUGGAUAUGUUCAAGCUUCUAGUUUCACAAUGUGAUAAUUUUAAAUAAUUGUUUUUGUAAUCAAAGUAUGAAUUUUAUGAUUUUAUAUUUCAUUCUAAUUUCUUAAAAAUAAUGUUGGUUGUUUUUGUAGAAUUAUUUUAUAAAUACAUAUUGUAAAAUCUCUAUGUUAUAUGAAAAAAUAUCUUUUAAAAUUUAAAUCCCAAUAAAAAUAUGACAAUACAAUUAUUUUGAUAUUGCAUUGUGUUAUUUGAAGCUUGAAAAUAGGCCAUCUUUAAAAUUAGAACUAAU

.....((((((((.(((.(((((((.(((.((((((((((.(((..(((((((..(((.....((((((((((..((((........(((....((((.(((((((..........((((((..((((((((((....))))))))))..))))))(((((((...(((((..(......)..)))))..)))))))..........))))))))))).....)))........)))).))))))))))...)))..)))))))..))).)))))))))).)))..)))))))))).)))))))).... (-78.99)

.................................AAGCTTCTAGTTTCACAATGTGAT............................................................................................................................................................................................................................................................ 2

.................................AAGCTTCTAGTTTCACAATGTGA............................................................................................................................................................................................................................................................. 1

..................................AGCTTCTAGTTTCACAATGTGA............................................................................................................................................................................................................................................................. 1

...............................................................................................................................................................................................................................................................TGCATTGTGTTATTTGAAGCTTGA.............................. 1

................................................................................................................................................................................................................................................................GCATTGTGTTATTTGAAGCTTGA.............................. 1

.......................................................................................................................................................................................................................................................................................AAATAGGCCATCTTTAAAATTAGAAC.... 1

>ppt-MIR319e_MI0005665_Physcomitrella_patens_miR319e_stem-loop GSM313213

CUAGCCGUGGGAGCUCCUUCCGGUUCAAUAGUGGCUGAUGUGAGGUUGCACUGCUGCCGAUUCAAACUUCCGGCUUCCCUUUCUUAACACGACAGGGAAUCCGAAUGUCUGAUGCGGGAGCUGUGCGGUCUUCAACUUUAGCGCCUCUUGGACUGAAGGGAGCUCCCAAGUCUU

.......((((((((((((.((((((((.((..(((((..(((((((((((.(((.(((..(((.((...(((.((((((.((.......)).)))))).)))...)).)))..))).))).)))))).)))))...)))))..)).)))))))).))))))))))))...... (-86.10)

.........GGAGCTCCTTCCGGTTCAAT................................................................................................................................................. 1

.........GGAGCTCCTTCCGGTTCAATA................................................................................................................................................ 1

..........GAGCTCCTTCCGGTTCAAT................................................................................................................................................. 9

..........GAGCTCCTTCCGGTTCAATA................................................................................................................................................ 41

..........GAGCTCCTTCCGGTTCAATAG............................................................................................................................................... 10

...........AGCTCCTTCCGGTTCAATA................................................................................................................................................ 3

......................................................TGCCGATTCAAACTTCCGGCT................................................................................................... 1

..........................................................................TTCCCTTTCTTAACACGACAGGGA............................................................................ 1

.............................................................................CCTTTCTTAACACGACAGGGAAT.......................................................................... 1

.......................................................................................................AATGTCTGATGCGGGAGCT.................................................... 1

...............................................................................................................................................CCTCTTGGACTGAAGGGAGCT.......... 1

................................................................................................................................................CTCTTGGACTGAAGGGAGCT.......... 1

................................................................................................................................................CTCTTGGACTGAAGGGAGCTC......... 1

.................................................................................................................................................TCTTGGACTGAAGGGAGCTCCC....... 1

.................................................................................................................................................TCTTGGACTGAAGGGAGCTCC........ 6

.................................................................................................................................................TCTTGGACTGAAGGGAGCTC......... 1

..................................................................................................................................................CTTGGACTGAAGGGAGCTC......... 21

..................................................................................................................................................CTTGGACTGAAGGGAGCTCCC....... 1254

..................................................................................................................................................CTTGGACTGAAGGGAGCTCCCA...... 24

..................................................................................................................................................CTTGGACTGAAGGGAGCTCC........ 367

..................................................................................................................................................CTTGGACTGAAGGGAGCTCCCAA..... 2

...................................................................................................................................................TTGGACTGAAGGGAGCTCC........ 13

...................................................................................................................................................TTGGACTGAAGGGAGCTCCCA...... 2

...................................................................................................................................................TTGGACTGAAGGGAGCTCCC....... 34

....................................................................................................................................................TGGACTGAAGGGAGCTCCC....... 12

....................................................................................................................................................TGGACTGAAGGGAGCTCCCA...... 2

.......................................................................................................................................................ACTGAAGGGAGCTCCCAAGT... 1

>ppt-MIR896_MI0005680_Physcomitrella_patens_miR896_stem-loop GSM313213

CCCACCCUGAGCGGGGGGGUCUGAUUGUGGUCGGUCGAGGGGCGCUGACCGUGUGGCCCAAUGGAUAAGGCGCUUGCCUACGGAGCAAGAGAUUCUGGGUUCGAUCCCCAGCAUGGUCGUGGGUCGCCGGAAUUGUAGAUUGUGGCGCGUGUCAAUUUGGCCGAGUGGUUAAGGCGGCAGACUCGAAAUCUGCUGGGGUUUCCCCGCGCAGGUUCAAAUCCUGCA

.....((((.((((((((..((.(..(..(....(((((.(.(((((((((..(((((.(((.((((..(((((((((....).)))...((((((((...((((((((.....))....)))))))))))))).........))))).)))).))).)))))..)))))..)))).)...)))))...)..)).))..)))))))).))))............. (-94.10)

........................TTGTGGTCGGTCGAGGGGCGCT................................................................................................................................................................................... 1

..........................GTGGTCGGTCGAGGGGCGCT................................................................................................................................................................................... 1

..............................................GACCGTGTGGCCCAATGGA................................................................................................................................................................ 29

..............................................GACCGTGTGGCCCAATGGATAA............................................................................................................................................................. 36

..............................................GACCGTGTGGCCCAATGGAT............................................................................................................................................................... 28

..............................................GACCGTGTGGCCCAATGGATA.............................................................................................................................................................. 27

..............................................GACCGTGTGGCCCAATGGATAAGGC.......................................................................................................................................................... 14

..............................................GACCGTGTGGCCCAATGGATAAGG........................................................................................................................................................... 9

..............................................GACCGTGTGGCCCAATGGATAAG............................................................................................................................................................ 6

...............................................ACCGTGTGGCCCAATGGAT............................................................................................................................................................... 1

...............................................ACCGTGTGGCCCAATGGATA.............................................................................................................................................................. 1

.........................................................CCAATGGATAAGGCGCTTGCCT.................................................................................................................................................. 1

.........................................................................TTGCCTACGGAGCAAGAGAT.................................................................................................................................... 1

..................................................................................GAGCAAGAGATTCTGGGTTCG.......................................................................................................................... 1

.....................................................................................CAAGAGATTCTGGGTTCGATC....................................................................................................................... 1

........................................................................................GAGATTCTGGGTTCGATCC...................................................................................................................... 1

...................................................................................................TTCGATCCCCAGCATGGTCGT......................................................................................................... 2

....................................................................................................TCGATCCCCAGCATGGTCG.......................................................................................................... 2

.......................................................................................................................TGGGTCGCCGGAATTGTAGAT..................................................................................... 1

...........................................................................................................................TCGCCGGAATTGTAGATTGTGGCGCG............................................................................ 1

............................................................................................................................CGCCGGAATTGTAGATTGTGGCGC............................................................................. 1

.............................................................................................................................GCCGGAATTGTAGATTGTGGCGCG............................................................................ 2

.............................................................................................................................GCCGGAATTGTAGATTGTGGC............................................................................... 3

.............................................................................................................................GCCGGAATTGTAGATTGTGGCGC............................................................................. 10

.............................................................................................................................GCCGGAATTGTAGATTGTGGCG.............................................................................. 14

.............................................................................................................................GCCGGAATTGTAGATTGTGGCGCGT........................................................................... 3

...............................................................................................................................CGGAATTGTAGATTGTGGCGCG............................................................................ 1

......................................................................................................................................................GTCAATTTGGCCGAGTGGTTAAGGC.................................................. 18

......................................................................................................................................................GTCAATTTGGCCGAGTGGTT....................................................... 101

......................................................................................................................................................GTCAATTTGGCCGAGTGGT........................................................ 109

......................................................................................................................................................GTCAATTTGGCCGAGTGGTTAAG.................................................... 130

......................................................................................................................................................GTCAATTTGGCCGAGTGGTTAAGG................................................... 67

......................................................................................................................................................GTCAATTTGGCCGAGTGGTTA...................................................... 117

......................................................................................................................................................GTCAATTTGGCCGAGTGGTTAA..................................................... 204

.......................................................................................................................................................TCAATTTGGCCGAGTGGTTAA..................................................... 1

.........................................................................................................................................................AATTTGGCCGAGTGGTTAAG.................................................... 5

.........................................................................................................................................................AATTTGGCCGAGTGGTTAAGG................................................... 2

..........................................................................................................................................................ATTTGGCCGAGTGGTTAAG.................................................... 2

..........................................................................................................................................................ATTTGGCCGAGTGGTTAAGG................................................... 1

...........................................................................................................................................................TTTGGCCGAGTGGTTAAGG................................................... 1

...........................................................................................................................................................TTTGGCCGAGTGGTTAAGGC.................................................. 1

..............................................................................................................................................................................CGGCAGACTCGAAATCTGCTGG............................. 2

......................................................................................................................................................................................TCGAAATCTGCTGGGGTTTCCC..................... 2

.......................................................................................................................................................................................CGAAATCTGCTGGGGTTTC....................... 1

.......................................................................................................................................................................................CGAAATCTGCTGGGGTTTCC...................... 1

.......................................................................................................................................................................................CGAAATCTGCTGGGGTTTCCCC.................... 1

..........................................................................................................................................................................................AATCTGCTGGGGTTTCCCCGCGCA............... 1

..........................................................................................................................................................................................AATCTGCTGGGGTTTCCCCGCG................. 1

...........................................................................................................................................................................................ATCTGCTGGGGTTTCCCCGCGCAGG............. 1

...........................................................................................................................................................................................ATCTGCTGGGGTTTCCCCGCGCA............... 1

............................................................................................................................................................................................TCTGCTGGGGTTTCCCCGCGCAGGT............ 4

............................................................................................................................................................................................TCTGCTGGGGTTTCCCCGCGCAGGTT........... 1

.............................................................................................................................................................................................CTGCTGGGGTTTCCCCGCGCAGGT............ 5

.............................................................................................................................................................................................CTGCTGGGGTTTCCCCGCGCAGGTT........... 1

.............................................................................................................................................................................................CTGCTGGGGTTTCCCCGCGCAG.............. 1

..............................................................................................................................................................................................TGCTGGGGTTTCCCCGCGCAGG............. 2

...............................................................................................................................................................................................GCTGGGGTTTCCCCGCGCAGGT............ 1

................................................................................................................................................................................................CTGGGGTTTCCCCGCGCAGGTTC.......... 1

.................................................................................................................................................................................................TGGGGTTTCCCCGCGCAGGTTCA......... 1

.....................................................................................................................................................................................................GTTTCCCCGCGCAGGTTCA......... 1

............................................................................................................................................................................................................CGCGCAGGTTCAAATCCTGCA 1

>ppt-MIR899_MI0005682_Physcomitrella_patens_miR899_stem-loop GSM313213

ACGGCGUUUUUCUCGGAGAAAAAUACCAUCCUUUGGUUCAACAACGGUUGCUGCGUUUUCCACGUUCGGAUACAACCCUGCCCAGUUGCUGUGAGAGAUUGACGAGUGCAAGAAGACCCUCUUUCCAUGUUCCAGACGCAGGCCUUCGCCGAACUGAGAUACAUCGCAAUCGACACACCGGCCGCUUCAGAAUCAGUGACUAGCAUACCCAGCUGCUAUUGG

..((..((((((.....))))))..)).....(((((((.....((((((.((.(((......((((((.......(((((...(..((((.((((((.....................)))))))).))..)....)))))......)))))).((......))......))).)).)))))).....)))))))....(((((........))))).... (-51.42)

.............CGGAGAAAAATACCATCCTTT............................................................................................................................................................................................ 1

......................................................GTTTTCCACGTTCGGATACAACCCT............................................................................................................................................... 1

.........................................................................................TGTGAGAGATTGACGAGTGCA................................................................................................................ 12

..............................................................................................GAGATTGACGAGTGCAAGAAG........................................................................................................... 2

...............................................................................................AGATTGACGAGTGCAAGAAG........................................................................................................... 3

...............................................................................................AGATTGACGAGTGCAAGAAGAC......................................................................................................... 1

...............................................................................................AGATTGACGAGTGCAAGAAGA.......................................................................................................... 152

...............................................................................................AGATTGACGAGTGCAAGAA............................................................................................................ 1

................................................................................................GATTGACGAGTGCAAGAAGA.......................................................................................................... 1

.......................................................................................................................................ACGCAGGCCTTCGCCGAACTGAGA............................................................... 1

.....................................................................................................................................................CGAACTGAGATACATCGCAAT.................................................... 1

..................................................................................................................................................................ATCGCAATCGACACACCGGCCG...................................... 1

.....................................................................................................................................................................GCAATCGACACACCGGCCGCT.................................... 1

>ppt-MIR1063b_MI0006023_Physcomitrella_patens_miR1063b_stem-loop GSM313213

AGAGUUACUUGUGCUUCGUAGUUCACUUACAUCUUGGAGUACUGCAUCUUCAGAUCUGUGAGUCUUCGCCGCCUAUUGCCUUCGCCACCAUGCUUGACGAAUGGUUUGAAACUGAUCACAUCCAACUUGAACGGAUAUCCUGCCCCUUCAUAGUGCUGUGAUUCCGUUCAAGCAGUUCAGUUGUCCGACAACCAUGGUGUGGUAGGACAGUAGUACUGCUAAGGCUUUGACAUUUUGAAGAUGCAGCACUACAAGAAGUGUGUGAGCAACGGGCUGGAUUCAAGAU

.(((((......(((.(((.((((((.(((.(((((.(((.(((((((((((((..((((((((((.((...(((((((((((((.((((((......((((.(((((((...(((((((....(((((((.((.........)).)))).)))..)))))))...))))))).)))).((((.....)))))))))))))).))).))))))....)).)))))))..))).))))))))))))).))).))))).))).)))))).))))))..)))))..... (-107.90)

.............................CATCTTGGAGTACTGCATCTT............................................................................................................................................................................................................................................ 21

.............................CATCTTGGAGTACTGCATCTTC........................................................................................................................................................................................................................................... 1

...............................TCTTGGAGTACTGCATCTTC........................................................................................................................................................................................................................................... 1

......................................................................GCCTATTGCCTTCGCCACCA.................................................................................................................................................................................................... 1

.........................................................................................................................................................................................................................................TTTGAAGATGCAGCACTACAA................................ 1

............................................................................................................................................................................................................................................GAAGATGCAGCACTACAAGAA............................. 1

...............................................................................................................................................................................................................................................GATGCAGCACTACAAGAAGT........................... 1

>ppt-MIR2083_MI0010537_Physcomitrella_patens_miR2083_stem-loop GSM313213

AAAAGUGUGUGUAUGCGGAAGUUGGAGAAAACCCAGACUUCAUUGCUCGAUGAAGCAUGUAGCUUCGCAGUCGUACUUCUUGCACUCCUCCAUCUCUCGCAUCUCUUGCGCAUUGUGGUUCUCCAACACCUGCACAGCGGCGGUCUUGUCCGGAUUAGGGAUCGUCUACACCAGAUAAGCCACGGCGUUUUUCUCGGAGAAAAAUACCAUCCUUUGGUUCAACAACGGUUGCUGCGUUUUCCACGUUCGGAUACAACCCUGCCCAGUUGCUGUGAGAGAUUGACGAGUGCAAGAAGACCCUCUUUCCAUGUUCCAGACGCA

....((((.((((((.(((((..((.(((....((..(((((((....)))))))..))....))).........((((((((((((.((.((((((((((.(..(((.(((.(((((....)).)))..)))...((((.(((..(((((((((...(((.((.......(((...((((..((..((((((.....))))))..)).....(((......))).)))).)))))...)))..)))))))))..))))))).)))..).)))))))))).)).)))))))))))).))..)))))))))...)).)))). (-109.51)

............................................................................TTCTTGCACTCCTCCATCT.................................................................................................................................................................................................................................. 2

............................................................................TTCTTGCACTCCTCCATCTCT................................................................................................................................................................................................................................ 91

............................................................................TTCTTGCACTCCTCCATCTC................................................................................................................................................................................................................................. 8

.............................................................................TCTTGCACTCCTCCATCTCTC............................................................................................................................................................................................................................... 1

................................................................................................TCGCATCTCTTGCGCATTGT............................................................................................................................................................................................................. 1

...........................................................................................................................CAACACCTGCACAGCGGCGG.................................................................................................................................................................................. 1

....................................................................................................................................................................................CACGGCGTTTTTCTCGGAGAAA....................................................................................................................... 1

..................................................................................................................................................................................................CGGAGAAAAATACCATCCTTT.......................................................................................................... 1

...........................................................................................................................................................................................................................................GTTTTCCACGTTCGGATACAACCCT............................................................. 1

..............................................................................................................................................................................................................................................................................TGTGAGAGATTGACGAGTGCA.............................. 12

...................................................................................................................................................................................................................................................................................GAGATTGACGAGTGCAAGAAG......................... 2

....................................................................................................................................................................................................................................................................................AGATTGACGAGTGCAAGAAG......................... 3

....................................................................................................................................................................................................................................................................................AGATTGACGAGTGCAAGAAGAC....................... 1

....................................................................................................................................................................................................................................................................................AGATTGACGAGTGCAAGAAGA........................ 152

....................................................................................................................................................................................................................................................................................AGATTGACGAGTGCAAGAA.......................... 1

.....................................................................................................................................................................................................................................................................................GATTGACGAGTGCAAGAAGA........................ 1

>ppt-MIR408b_MI0005917_Physcomitrella_patens_miR408b_stem-loop GSM313213

GUGGAAGAGAGAGAGUGGUGGAAGGGAGGGAAGCCAGCGUGAGGCAAUGCAUGACAACAGCAUGCCCAGGAGGUCCUGAGGGUGUUGUCCUCAUGCACUGCCUCUUCCCUGGCUUCCCUACAUAGCUCGCCAUUCUUGUGCUCU

.....((((.(((((((((((....((((((((((((.(.((((((.(((((((..(((((((.(.((((....)))).).)))))))..))))))).))))))..).))))))))))).)....).))))))))))...)))) (-82.00)

........GAGAGAGTGGTGGAAGGGAGGG.................................................................................................................. 1

..........GAGAGTGGTGGAAGGGAGGGAAG............................................................................................................... 1

..........GAGAGTGGTGGAAGGGAGGGAAGC.............................................................................................................. 1

.................................CCAGCGTGAGGCAATGCATGA.......................................................................................... 13

.................................CCAGCGTGAGGCAATGCATG........................................................................................... 2

.................................CCAGCGTGAGGCAATGCAT............................................................................................ 1

..................................CAGCGTGAGGCAATGCATGA.......................................................................................... 36

..................................CAGCGTGAGGCAATGCATGAC......................................................................................... 1

...................................AGCGTGAGGCAATGCATGA.......................................................................................... 2

............................................................................................ATGCACTGCCTCTTCCCTGGCT.............................. 1

............................................................................................ATGCACTGCCTCTTCCCTGGC............................... 1

.............................................................................................TGCACTGCCTCTTCCCTGGCT.............................. 9

.............................................................................................TGCACTGCCTCTTCCCTGG................................ 2

.............................................................................................TGCACTGCCTCTTCCCTGGC............................... 1

>ppt-MIR1068_MI0006034_Physcomitrella_patens_miR1068_stem-loop GSM313213

CUUUUCAACACCUUGGCGCUGGCCUUCGUAGCCAUUUGCUUGAAGGUCAACUCAGACGGGUGGAAGAGGCAAUGUAUUGACGUCUUCCUCCAUUUUCCGACCAUUCUCUAGGCACCACAGAGGCAUCAAUGCUCUGCCUGUUUCUGCCGUGUGGGUUGGUCCUCAAGCAACGGGCUACGAGGACCAAUGUCAAUAAGCAUCAAC

............((((((.(((.((((((((((..((((((((.(..(((((((.((((..((((.(((((..(((((((.(((((..........((............)).......))))).)))))))..))))).))))..)))).)))))))..).))))))))..)))))))))).))).))))))........... (-88.73)

............................TAGCCATTTGCTTGAAGGTCAA.......................................................................................................................................................... 3

............................TAGCCATTTGCTTGAAGGTCA........................................................................................................................................................... 11

.............................AGCCATTTGCTTGAAGGTCAA.......................................................................................................................................................... 7

.................................................ACTCAGACGGGTGGAAGAGGCA..................................................................................................................................... 1

.............................................................................................................................................................GGTCCTCAAGCAACGGGCTAC.......................... 1

..............................................................................................................................................................GTCCTCAAGCAACGGGCTACG......................... 1

...............................................................................................................................................................TCCTCAAGCAACGGGCTACG......................... 1

>ppt-MIR1069_MI0006035_Physcomitrella_patens_miR1069_stem-loop GSM313213

UGUUCUUCAAGCACAUGUCCACUUCAUGUUCUCAAGUCUUAUCAUUGGAUUGAGCACCUUGGUCUUUCAAAUAUUCUAUUAUGAUAUGUUGAUAUAUAAUUGGUCUUUCAAGUAUUUUCUCAUUAAUAUGAAAACAUAUUAUUACAAAAUAUUUUUGCAUAUUAUUGUAGUAUACUUGAUAAAUCAAAGUGCUCACUGUAAUGAUAAAGCUUGAGAACAUGAAGUCUACUUGACUUCUACUUUG

.............((.((..((((((((((((((((..(((((((((.(.(((((((.(((((...((((.(((.((((.(((((((((.(((((....((((....))))............(((((((....))))))).......)))))...))))))))).)))).))).))))...))))).))))))).).)))))))))..))))))))))))))))..)).))............ (-79.20)

............................TTCTCAAGTCTTATCATTGGAT.................................................................................................................................................................................................. 1

.............................TCTCAAGTCTTATCATTGGAT.................................................................................................................................................................................................. 9

...............................TCAAGTCTTATCATTGGATTG................................................................................................................................................................................................ 1

.................................................TTGAGCACCTTGGTCTTTCAA.............................................................................................................................................................................. 1

..................................................TGAGCACCTTGGTCTTTCAAA............................................................................................................................................................................. 1

...............................................................................................................................................................................TTGATAAATCAAAGTGCTCACT............................................... 2

>ppt-MIR319e_MI0005665_Physcomitrella_patens_miR319e_stem-loop GSM313214

CUAGCCGUGGGAGCUCCUUCCGGUUCAAUAGUGGCUGAUGUGAGGUUGCACUGCUGCCGAUUCAAACUUCCGGCUUCCCUUUCUUAACACGACAGGGAAUCCGAAUGUCUGAUGCGGGAGCUGUGCGGUCUUCAACUUUAGCGCCUCUUGGACUGAAGGGAGCUCCCAAGUCUU

.......((((((((((((.((((((((.((..(((((..(((((((((((.(((.(((..(((.((...(((.((((((.((.......)).)))))).)))...)).)))..))).))).)))))).)))))...)))))..)).)))))))).))))))))))))...... (-86.10)

..........GAGCTCCTTCCGGTTCAATA................................................................................................................................................ 5

..........GAGCTCCTTCCGGTTCAAT................................................................................................................................................. 3

..........GAGCTCCTTCCGGTTCAATAG............................................................................................................................................... 16

..........GAGCTCCTTCCGGTTCAATAGT.............................................................................................................................................. 1

...........AGCTCCTTCCGGTTCAATAG............................................................................................................................................... 1

...............................TGGCTGATGTGAGGTTGCAC........................................................................................................................... 6

.......................................................................................................AATGTCTGATGCGGGAGCTGT.................................................. 1

.......................................................................................................AATGTCTGATGCGGGAGCTG................................................... 1

............................................................................................................................GCGGTCTTCAACTTTAGCGCC............................. 2

............................................................................................................................GCGGTCTTCAACTTTAGCGCCT............................ 2

............................................................................................................................GCGGTCTTCAACTTTAGCGC.............................. 1

...............................................................................................................................................CCTCTTGGACTGAAGGGAGCT.......... 1

................................................................................................................................................CTCTTGGACTGAAGGGAGCTC......... 1

................................................................................................................................................CTCTTGGACTGAAGGGAGCT.......... 2

.................................................................................................................................................TCTTGGACTGAAGGGAGCTCCC....... 26

.................................................................................................................................................TCTTGGACTGAAGGGAGCT.......... 1

.................................................................................................................................................TCTTGGACTGAAGGGAGCTCC........ 43

.................................................................................................................................................TCTTGGACTGAAGGGAGCTC......... 2

..................................................................................................................................................CTTGGACTGAAGGGAGCTC......... 88

..................................................................................................................................................CTTGGACTGAAGGGAGCTCC........ 4487

..................................................................................................................................................CTTGGACTGAAGGGAGCTCCC....... 18658

..................................................................................................................................................CTTGGACTGAAGGGAGCTCCCA...... 16

...................................................................................................................................................TTGGACTGAAGGGAGCTCCCA...... 3

...................................................................................................................................................TTGGACTGAAGGGAGCTCCC....... 394

...................................................................................................................................................TTGGACTGAAGGGAGCTCC........ 130

....................................................................................................................................................TGGACTGAAGGGAGCTCCC....... 22

....................................................................................................................................................TGGACTGAAGGGAGCTCCCA...... 2

......................................................................................................................................................GACTGAAGGGAGCTCCCAAGT... 1

>ppt-MIR896_MI0005680_Physcomitrella_patens_miR896_stem-loop GSM313214

CCCACCCUGAGCGGGGGGGUCUGAUUGUGGUCGGUCGAGGGGCGCUGACCGUGUGGCCCAAUGGAUAAGGCGCUUGCCUACGGAGCAAGAGAUUCUGGGUUCGAUCCCCAGCAUGGUCGUGGGUCGCCGGAAUUGUAGAUUGUGGCGCGUGUCAAUUUGGCCGAGUGGUUAAGGCGGCAGACUCGAAAUCUGCUGGGGUUUCCCCGCGCAGGUUCAAAUCCUGCA

.....((((.((((((((..((.(..(..(....(((((.(.(((((((((..(((((.(((.((((..(((((((((....).)))...((((((((...((((((((.....))....)))))))))))))).........))))).)))).))).)))))..)))))..)))).)...)))))...)..)).))..)))))))).))))............. (-94.10)

..............................................GACCGTGTGGCCCAATGGA................................................................................................................................................................ 93

..............................................GACCGTGTGGCCCAATGGATAAGGC.......................................................................................................................................................... 2

..............................................GACCGTGTGGCCCAATGGAT............................................................................................................................................................... 20

..............................................GACCGTGTGGCCCAATGGATAA............................................................................................................................................................. 12

..............................................GACCGTGTGGCCCAATGGATAAGG........................................................................................................................................................... 8

..............................................GACCGTGTGGCCCAATGGATAAG............................................................................................................................................................ 18

..............................................GACCGTGTGGCCCAATGGATA.............................................................................................................................................................. 20

...............................................ACCGTGTGGCCCAATGGATA.............................................................................................................................................................. 1

..................................................GTGTGGCCCAATGGATAAG............................................................................................................................................................ 1

....................................................GTGGCCCAATGGATAAGGCGCT....................................................................................................................................................... 1

..........................................................CAATGGATAAGGCGCTTGCCT.................................................................................................................................................. 1

..............................................................GGATAAGGCGCTTGCCTACGGA............................................................................................................................................. 1

.................................................................................GGAGCAAGAGATTCTGGGTTCG.......................................................................................................................... 1

..................................................................................GAGCAAGAGATTCTGGGTT............................................................................................................................ 1

..................................................................................GAGCAAGAGATTCTGGGTTC........................................................................................................................... 12

......................................................................................AAGAGATTCTGGGTTCGATCCC..................................................................................................................... 1

......................................................................................AAGAGATTCTGGGTTCGATCC...................................................................................................................... 1

......................................................................................AAGAGATTCTGGGTTCGATCCCCA................................................................................................................... 1

..........................................................................................GATTCTGGGTTCGATCCCCAGCA................................................................................................................ 2

...........................................................................................................................TCGCCGGAATTGTAGATTGTGGCGCG............................................................................ 1

............................................................................................................................CGCCGGAATTGTAGATTGTGGCGC............................................................................. 1

.............................................................................................................................GCCGGAATTGTAGATTGTGGCGC............................................................................. 49

.............................................................................................................................GCCGGAATTGTAGATTGTGGCGCGT........................................................................... 6

.............................................................................................................................GCCGGAATTGTAGATTGTGG................................................................................ 2

.............................................................................................................................GCCGGAATTGTAGATTGTGGCG.............................................................................. 21

.............................................................................................................................GCCGGAATTGTAGATTGTGGCGCG............................................................................ 2

.............................................................................................................................GCCGGAATTGTAGATTGTG................................................................................. 3

..............................................................................................................................CCGGAATTGTAGATTGTGGCGCGT........................................................................... 1

................................................................................................................................GGAATTGTAGATTGTGGCG.............................................................................. 1

...................................................................................................................................ATTGTAGATTGTGGCGCGT........................................................................... 1

......................................................................................................................................................GTCAATTTGGCCGAGTGGTTAAGG................................................... 19

......................................................................................................................................................GTCAATTTGGCCGAGTGGTTA...................................................... 43

......................................................................................................................................................GTCAATTTGGCCGAGTGGTT....................................................... 98

......................................................................................................................................................GTCAATTTGGCCGAGTGGTTAA..................................................... 23

......................................................................................................................................................GTCAATTTGGCCGAGTGGTTAAG.................................................... 38

......................................................................................................................................................GTCAATTTGGCCGAGTGGT........................................................ 47

........................................................................................................................................................CAATTTGGCCGAGTGGTTA...................................................... 1

.........................................................................................................................................................AATTTGGCCGAGTGGTTAAGGC.................................................. 1

.........................................................................................................................................................AATTTGGCCGAGTGGTTAAG.................................................... 1

...........................................................................................................................................................TTTGGCCGAGTGGTTAAGG................................................... 1

..........................................................................................................................................................................AAGGCGGCAGACTCGAAATC................................... 1

..............................................................................................................................................................................CGGCAGACTCGAAATCTGCTGG............................. 1

..................................................................................................................................................................................AGACTCGAAATCTGCTGGGGT.......................... 1

...................................................................................................................................................................................GACTCGAAATCTGCTGGGGTTTC....................... 1

......................................................................................................................................................................................TCGAAATCTGCTGGGGTTTCCCCGC.................. 1

.......................................................................................................................................................................................CGAAATCTGCTGGGGTTTC....................... 1

.......................................................................................................................................................................................CGAAATCTGCTGGGGTTTCC...................... 4

........................................................................................................................................................................................GAAATCTGCTGGGGTTTCCCC.................... 1

........................................................................................................................................................................................GAAATCTGCTGGGGTTTCCC..................... 2

..........................................................................................................................................................................................AATCTGCTGGGGTTTCCCCGCG................. 1

...........................................................................................................................................................................................ATCTGCTGGGGTTTCCCCGCGC................ 1

..............................................................................................................................................................................................TGCTGGGGTTTCCCCGCGCAGGT............ 1

.....................................................................................................................................................................................................GTTTCCCCGCGCAGGTTCA......... 1

>ppt-MIR2083_MI0010537_Physcomitrella_patens_miR2083_stem-loop GSM313214

AAAAGUGUGUGUAUGCGGAAGUUGGAGAAAACCCAGACUUCAUUGCUCGAUGAAGCAUGUAGCUUCGCAGUCGUACUUCUUGCACUCCUCCAUCUCUCGCAUCUCUUGCGCAUUGUGGUUCUCCAACACCUGCACAGCGGCGGUCUUGUCCGGAUUAGGGAUCGUCUACACCAGAUAAGCCACGGCGUUUUUCUCGGAGAAAAAUACCAUCCUUUGGUUCAACAACGGUUGCUGCGUUUUCCACGUUCGGAUACAACCCUGCCCAGUUGCUGUGAGAGAUUGACGAGUGCAAGAAGACCCUCUUUCCAUGUUCCAGACGCA

....((((.((((((.(((((..((.(((....((..(((((((....)))))))..))....))).........((((((((((((.((.((((((((((.(..(((.(((.(((((....)).)))..)))...((((.(((..(((((((((...(((.((.......(((...((((..((..((((((.....))))))..)).....(((......))).)))).)))))...)))..)))))))))..))))))).)))..).)))))))))).)).)))))))))))).))..)))))))))...)).)))). (-109.51)

................GGAAGTTGGAGAAAACCCA.............................................................................................................................................................................................................................................................................................. 1

.....................................................AGCATGTAGCTTCGCAGTCGTA...................................................................................................................................................................................................................................................... 1

.....................................................................GTCGTACTTCTTGCACTCCT........................................................................................................................................................................................................................................ 1

..........................................................................ACTTCTTGCACTCCTCCATCTCT................................................................................................................................................................................................................................ 1

...........................................................................CTTCTTGCACTCCTCCATCTCT................................................................................................................................................................................................................................ 1

............................................................................TTCTTGCACTCCTCCATCTCT................................................................................................................................................................................................................................ 398

............................................................................TTCTTGCACTCCTCCATCT.................................................................................................................................................................................................................................. 3

............................................................................TTCTTGCACTCCTCCATCTC................................................................................................................................................................................................................................. 3

............................................................................TTCTTGCACTCCTCCATCTCTC............................................................................................................................................................................................................................... 1

.............................................................................TCTTGCACTCCTCCATCTCTC............................................................................................................................................................................................................................... 7

.............................................................................TCTTGCACTCCTCCATCTCT................................................................................................................................................................................................................................ 2

..............................................................................CTTGCACTCCTCCATCTCT................................................................................................................................................................................................................................ 2

..................................................................................CACTCCTCCATCTCTCGCATC.......................................................................................................................................................................................................................... 1

.........................................................................................................................................................................ACCAGATAAGCCACGGCGTTT................................................................................................................................... 1

.............................................................................................................................................................................................................................ACAACGGTTGCTGCGTTTTCC............................................................................... 1

..................................................................................................................................................................................................................................................ACGTTCGGATACAACCCTGCC.......................................................... 1

....................................................................................................................................................................................................................................................................GCCCAGTTGCTGTGAGAGATT........................................ 1

..............................................................................................................................................................................................................................................................................TGTGAGAGATTGACGAGTGCA.............................. 32

...................................................................................................................................................................................................................................................................................GAGATTGACGAGTGCAAGAA.......................... 3

...................................................................................................................................................................................................................................................................................GAGATTGACGAGTGCAAGAAG......................... 2

....................................................................................................................................................................................................................................................................................AGATTGACGAGTGCAAGAAG......................... 3

....................................................................................................................................................................................................................................................................................AGATTGACGAGTGCAAGAAGAC....................... 1

....................................................................................................................................................................................................................................................................................AGATTGACGAGTGCAAGAA.......................... 2

....................................................................................................................................................................................................................................................................................AGATTGACGAGTGCAAGAAGA........................ 171

.....................................................................................................................................................................................................................................................................................GATTGACGAGTGCAAGAAGA........................ 1

.........................................................................................................................................................................................................................................................................................GACGAGTGCAAGAAGACCCTC................... 1

>ppt-MIR534a_MI0003501_Physcomitrella_patens_miR534a_stem-loop GSM313214

AUAUGCAUGCAACUUGUGUGGACAGACUGACUAGUCUAGUGGUGUAGUGGAAAGUAUUAGUUGGUUGAUGCACGAUGUUGUUUUGCAACAAGUGACUCGUCGCUGCAAUACUGAACCCACAAGUUUUGGAAGUUGUUUUGAUUGGGGCCACCCACAUUGCUCGACUAGAUUCAGUAUGUCCAUUGCAGUUGCAUACAUAU

(((((.((((((((...(((((((.(((((((((((.((..((((.((((..((((((.((.(((.(((((((..(((((.....))))).)))...)))))))))))))))...((((((((.............))))..)))).))))..))))..)).))))))..))))).)))))))...)))))))).))))) (-75.32)

.....CATGCAACTTGTGTGGACAGAC............................................................................................................................................................................. 20

.....CATGCAACTTGTGTGGACAGACT............................................................................................................................................................................ 2

.....CATGCAACTTGTGTGGACAGA.............................................................................................................................................................................. 5

......ATGCAACTTGTGTGGACAGA.............................................................................................................................................................................. 3507

......ATGCAACTTGTGTGGACAG............................................................................................................................................................................... 34

......ATGCAACTTGTGTGGACAGAC............................................................................................................................................................................. 5384

......ATGCAACTTGTGTGGACAGACT............................................................................................................................................................................ 5363

......ATGCAACTTGTGTGGACAGACTG........................................................................................................................................................................... 2

.......TGCAACTTGTGTGGACAGACT............................................................................................................................................................................ 214

.......TGCAACTTGTGTGGACAGAC............................................................................................................................................................................. 79

.......TGCAACTTGTGTGGACAGA.............................................................................................................................................................................. 49

.......TGCAACTTGTGTGGACAGACTG........................................................................................................................................................................... 4

........GCAACTTGTGTGGACAGACT............................................................................................................................................................................ 2

........GCAACTTGTGTGGACAGAC............................................................................................................................................................................. 4

.........CAACTTGTGTGGACAGACT............................................................................................................................................................................ 1

...........................TGACTAGTCTAGTGGTGTAGT........................................................................................................................................................ 2

...........................TGACTAGTCTAGTGGTGTAG......................................................................................................................................................... 1

........................................................................................................................................................CACATTGCTCGACTAGATTCAGT......................... 1

..............................................................................................................................................................................TATGTCCATTGCAGTTGCATA..... 24

..............................................................................................................................................................................TATGTCCATTGCAGTTGCATAC.... 492

..............................................................................................................................................................................TATGTCCATTGCAGTTGCAT...... 3

..............................................................................................................................................................................TATGTCCATTGCAGTTGCA....... 10

...............................................................................................................................................................................ATGTCCATTGCAGTTGCATAC.... 167

...............................................................................................................................................................................ATGTCCATTGCAGTTGCATA..... 2

...............................................................................................................................................................................ATGTCCATTGCAGTTGCATACA... 1

................................................................................................................................................................................TGTCCATTGCAGTTGCATAC.... 2

>ppt-MIR538c_MI0003512_Physcomitrella_patens_miR538c_stem-loop GSM313214

UUACAAAGAGUUCUUGAGUCUCCAUGCUUCUCUGACGUUGCAUGGAGUCUAUGUCUGGACCUUCUCCAUUUCCUCUUUCAAGAGAAUGUGAGCAUUGAAGGAUUCGGACAUAGCCUUCAUGCAUGCUAGAGUAUCAUGGAGGCCCGUGAACUACUUGUAA

.(((((..(((((.((.(((((((((...(((((.(((.((((((((.((((((((((((((((.((((...((((....))))...))).)....))))).))))))))))).))))))))))).)))))...))))))))).)).)))))..))))). (-73.90)

................AGTCTCCATGCTTCTCTGACGT.......................................................................................................................... 4

................AGTCTCCATGCTTCTCTGACG........................................................................................................................... 27

................AGTCTCCATGCTTCTCTGAC............................................................................................................................ 6

...................................CGTTGCATGGAGTCTATGTCT........................................................................................................ 2

.....................................TTGCATGGAGTCTATGTCTGG...................................................................................................... 27

.....................................TTGCATGGAGTCTATGTCTG....................................................................................................... 1

.....................................TTGCATGGAGTCTATGTCTGGAC.................................................................................................... 10

.....................................TTGCATGGAGTCTATGTCT........................................................................................................ 1

.....................................TTGCATGGAGTCTATGTCTGGA..................................................................................................... 3507

......................................TGCATGGAGTCTATGTCTGGAC.................................................................................................... 6

......................................TGCATGGAGTCTATGTCTGG...................................................................................................... 50

......................................TGCATGGAGTCTATGTCTGGA..................................................................................................... 719

......................................TGCATGGAGTCTATGTCTG....................................................................................................... 3

.......................................GCATGGAGTCTATGTCTGG...................................................................................................... 1

.......................................GCATGGAGTCTATGTCTGGA..................................................................................................... 28

........................................CATGGAGTCTATGTCTGGA..................................................................................................... 2

........................................................................................................CGGACATAGCCTTCATGCAT.................................... 26

........................................................................................................CGGACATAGCCTTCATGCATG................................... 214

........................................................................................................CGGACATAGCCTTCATGCATGC.................................. 1

........................................................................................................CGGACATAGCCTTCATGCA..................................... 1

.........................................................................................................GGACATAGCCTTCATGCATG................................... 4

.............................................................................................................................CTAGAGTATCATGGAGGCC................ 41

.............................................................................................................................CTAGAGTATCATGGAGGCCC............... 6

.............................................................................................................................CTAGAGTATCATGGAGGCCCG.............. 3703

.............................................................................................................................CTAGAGTATCATGGAGGCCCGT............. 43

..............................................................................................................................TAGAGTATCATGGAGGCCCG.............. 118

..............................................................................................................................TAGAGTATCATGGAGGCCCGT............. 1

...............................................................................................................................AGAGTATCATGGAGGCCCG.............. 7

...................................................................................................................................TATCATGGAGGCCCGTGAACT........ 1

.....................................................................................................................................TCATGGAGGCCCGTGAACTA....... 1

.....................................................................................................................................TCATGGAGGCCCGTGAACTAC...... 15

......................................................................................................................................CATGGAGGCCCGTGAACTAC...... 1

>ppt-MIR1219a_MI0004718_Physcomitrella_patens_miR1219a_stem-loop GSM313214

UGAAGUGUGGACGAUGGAGAGUCAGCCUCUUCCUGCCUCUCACUAGCUUCAUCCCUUCCUCCCUAAAUUUUAGUCUGGGAGGGAAGGAGCUAUUGGUGGUCAGGAAUAGCGCACCCUUCAUUUAUCCACACUUCA

.((((((((((.(((((((.((..((((.((((((((.(.((.(((((((.((((((((...(((.....)))...)))))))).))))))).))).)).)))))).)).)))).)))))))..)))))))))). (-67.20)

...AGTGTGGACGATGGAGAGTCAGCCT........................................................................................................... 1

.....TGTGGACGATGGAGAGTCAGC............................................................................................................. 13

.....TGTGGACGATGGAGAGTCA............................................................................................................... 26

.....TGTGGACGATGGAGAGTCAG.............................................................................................................. 56

.....TGTGGACGATGGAGAGTCAGCCT........................................................................................................... 179

.....TGTGGACGATGGAGAGTCAGCC............................................................................................................ 328

......GTGGACGATGGAGAGTCAGC............................................................................................................. 2

......GTGGACGATGGAGAGTCAGCC............................................................................................................ 10

......GTGGACGATGGAGAGTCAG.............................................................................................................. 3

......GTGGACGATGGAGAGTCAGCCT........................................................................................................... 3

.......TGGACGATGGAGAGTCAGCC............................................................................................................ 3

............................CTTCCTGCCTCTCACTAGC........................................................................................ 4

............................CTTCCTGCCTCTCACTAGCTT...................................................................................... 213

............................CTTCCTGCCTCTCACTAGCT....................................................................................... 1

.............................TTCCTGCCTCTCACTAGCTT...................................................................................... 3

.............................TTCCTGCCTCTCACTAGCTTC..................................................................................... 1

...............................................................TAAATTTTAGTCTGGGAGGGAAGGA............................................... 1

...................................................................................AAGGAGCTATTGGTGGTCAGGA.............................. 2

....................................................................................AGGAGCTATTGGTGGTCAGGA.............................. 1

........................................................................................GCTATTGGTGGTCAGGAATAGC......................... 1

........................................................................................GCTATTGGTGGTCAGGAAT............................ 1

........................................................................................GCTATTGGTGGTCAGGAATAG.......................... 92

........................................................................................GCTATTGGTGGTCAGGAATA........................... 8

.............................................................................................................CGCACCCTTCATTTATCCA....... 5

.............................................................................................................CGCACCCTTCATTTATCCACACT... 1

>ppt-MIR904a_MI0005692_Physcomitrella_patens_miR904a_stem-loop GSM313214

UGUUGGAGUUCCUUCGGUGUGGUGUUGCAAUAUUUGUGAUGCUAAUAUGGUCUUGUCAAUGUUUAGGGGCAAAGGCCUGAUUAUGCAGAUGCUUGGUGUAAGCUUAUGCUUGUACAACUUUUGCUAUUGGCCUCCACCCCUAAAUCUUGGCAAGACCUAAUUAGCCCACAACAUUGAAUUGACCGGGACCGGCGACUAAUCAGUACGAGCUCAAGCAGGA

((((.((((((..(((((.(.(.(((.((((..(((((..((((((..(((((((((((.(((((((((...(((((.(((...(((((......((((((((....))))))))....))))).))))))))...))))))))).)))))))))))..)))))).))))).))))....)))).).)))))..(((....)))..)))))).))))... (-86.60)

...............................................TGGTCTTGTCAATGTTTAGGG........................................................................................................................................................ 5

................................................GGTCTTGTCAATGTTTAGGGG....................................................................................................................................................... 2

..................................................TCTTGTCAATGTTTAGGGG....................................................................................................................................................... 180

..................................................TCTTGTCAATGTTTAGGGGCAAA................................................................................................................................................... 4

..................................................TCTTGTCAATGTTTAGGGGCAA.................................................................................................................................................... 187

..................................................TCTTGTCAATGTTTAGGGGC...................................................................................................................................................... 58

..................................................TCTTGTCAATGTTTAGGGGCA..................................................................................................................................................... 3938

...................................................CTTGTCAATGTTTAGGGGCAA.................................................................................................................................................... 124

...................................................CTTGTCAATGTTTAGGGGC...................................................................................................................................................... 9

...................................................CTTGTCAATGTTTAGGGGCA..................................................................................................................................................... 1930

....................................................TTGTCAATGTTTAGGGGCA..................................................................................................................................................... 37

....................................................TTGTCAATGTTTAGGGGCAA.................................................................................................................................................... 11

.......................................................................AAGGCCTGATTATGCAGAT.................................................................................................................................. 1

.......................................................................AAGGCCTGATTATGCAGATGCTTGGT........................................................................................................................... 1

.......................................................................................................................................ACCCCTAAATCTTGGCAAGACC............................................................... 2

.......................................................................................................................................ACCCCTAAATCTTGGCAAGAC................................................................ 3

.......................................................................................................................................ACCCCTAAATCTTGGCAAGA................................................................. 1

........................................................................................................................................CCCCTAAATCTTGGCAAGA................................................................. 7

........................................................................................................................................CCCCTAAATCTTGGCAAGACC............................................................... 797

........................................................................................................................................CCCCTAAATCTTGGCAAGAC................................................................ 20

.........................................................................................................................................CCCTAAATCTTGGCAAGACC............................................................... 75

.........................................................................................................................................CCCTAAATCTTGGCAAGAC................................................................ 1

..........................................................................................................................................CCTAAATCTTGGCAAGACC............................................................... 2

...........................................................................................................................................CTAAATCTTGGCAAGACCTA............................................................. 1

...........................................................................................................................................CTAAATCTTGGCAAGACCTAA............................................................ 36

............................................................................................................................................TAAATCTTGGCAAGACCTAAT........................................................... 2

............................................................................................................................................TAAATCTTGGCAAGACCTAA............................................................ 10

................................................................................................................................................TCTTGGCAAGACCTAATTAG........................................................ 1

CUUUUCAACACCUUGGCGCUGGCCUUCGUAGCCAUUUGCUUGAAGGUCAACUCAGACGGGUGGAAGAGGCAAUGUAUUGACGUCUUCCUCCAUUUUCCGACCAUUCUCUAGGCACCACAGAGGCAUCAAUGCUCUGCCUGUUUCUGCCGUGUGGGUUGGUCCUCAAGCAACGGGCUACGAGGACCAAUGUCAAUAAGCAUCAAC

............((((((.(((.((((((((((..((((((((.(..(((((((.((((..((((.(((((..(((((((.(((((..........((............)).......))))).)))))))..))))).))))..)))).)))))))..).))))))))..)))))))))).))).))))))........... (-88.73)

............................TAGCCATTTGCTTGAAGGT............................................................................................................................................................. 1

.............................AGCCATTTGCTTGAAGGTCAA.......................................................................................................................................................... 4

........................................TGAAGGTCAACTCAGACGGGT............................................................................................................................................... 3

.................................................ACTCAGACGGGTGGAAGAGG....................................................................................................................................... 1

.................................................ACTCAGACGGGTGGAAGAGGC...................................................................................................................................... 1

.............................................................................................................................................................GGTCCTCAAGCAACGGGCTAC.......................... 3

..............................................................................................................................................................GTCCTCAAGCAACGGGCTACG......................... 6

...............................................................................................................................................................TCCTCAAGCAACGGGCTACG......................... 1

>ppt-MIR1069_MI0006035_Physcomitrella_patens_miR1069_stem-loop GSM313214

UGUUCUUCAAGCACAUGUCCACUUCAUGUUCUCAAGUCUUAUCAUUGGAUUGAGCACCUUGGUCUUUCAAAUAUUCUAUUAUGAUAUGUUGAUAUAUAAUUGGUCUUUCAAGUAUUUUCUCAUUAAUAUGAAAACAUAUUAUUACAAAAUAUUUUUGCAUAUUAUUGUAGUAUACUUGAUAAAUCAAAGUGCUCACUGUAAUGAUAAAGCUUGAGAACAUGAAGUCUACUUGACUUCUACUUUG

.............((.((..((((((((((((((((..(((((((((.(.(((((((.(((((...((((.(((.((((.(((((((((.(((((....((((....))))............(((((((....))))))).......)))))...))))))))).)))).))).))))...))))).))))))).).)))))))))..))))))))))))))))..)).))............ (-79.20)

............................TTCTCAAGTCTTATCATTGGA................................................................................................................................................................................................... 2

...............................TCAAGTCTTATCATTGGATTGA............................................................................................................................................................................................... 1

.................................................TTGAGCACCTTGGTCTTTCAA.............................................................................................................................................................................. 1

.................................................TTGAGCACCTTGGTCTTTCAAATAT.......................................................................................................................................................................... 1

>ppt-MIR319b_MI0003497_Physcomitrella_patens_miR319b_stem-loop GSM313215

GAGCUCUUUUCAGUCCAGUAGCAGCUAAUGUCGAAGGUUGUACCGCUGCCGACUCAAACUUCCGGCUUCCAUAUCACGACGCGUGAUAUGGAAUCCGAAGGUCUGAUCCGGGAGCUGAUCGAUCUCCAGGUUAGCAUCUCUUGGACUGAAGGGAGCUCCU

((((((((((((((((((.((..((((((.(.((.(((((....(((.(((..(((.((((.(((.((((((((((((...)))))))))))).))).)))).)))..))).)))....))))))).).))))))..)).)))))))))))))))))).. (-83.70)

GAGCTCTTTTCAGTCCAGTAG........................................................................................................................................... 2

GAGCTCTTTTCAGTCCAGTA............................................................................................................................................ 6

GAGCTCTTTTCAGTCCAGT............................................................................................................................................. 3

.AGCTCTTTTCAGTCCAGTA............................................................................................................................................ 5

.AGCTCTTTTCAGTCCAGTAG........................................................................................................................................... 23

..GCTCTTTTCAGTCCAGTAG........................................................................................................................................... 1

.....................CAGCTAATGTCGAAGGTTGTAC..................................................................................................................... 1

......................AGCTAATGTCGAAGGTTGTA...................................................................................................................... 1

......................AGCTAATGTCGAAGGTTGTAC..................................................................................................................... 1

...........................................CGCTGCCGACTCAAACTTCCG................................................................................................ 2

.......................................................................TATCACGACGCGTGATATGGAAT.................................................................. 1

........................................................................ATCACGACGCGTGATATGGAA................................................................... 2

.................................................................................................AAGGTCTGATCCGGGAGCTG........................................... 1

.................................................................................................AAGGTCTGATCCGGGAGCTGA.......................................... 28

.................................................................................................AAGGTCTGATCCGGGAGCTGAT......................................... 1

.................................................................................................................................GTTAGCATCTCTTGGACTGAA.......... 1

..................................................................................................................................TTAGCATCTCTTGGACTGAA.......... 7

..................................................................................................................................TTAGCATCTCTTGGACTGAAG......... 388

...................................................................................................................................TAGCATCTCTTGGACTGAAG......... 4

.....................................................................................................................................GCATCTCTTGGACTGAAGGGAGCTCC. 1

.......................................................................................................................................ATCTCTTGGACTGAAGGGAGC.... 3

........................................................................................................................................TCTCTTGGACTGAAGGGAGC.... 2

........................................................................................................................................TCTCTTGGACTGAAGGGAGCT... 19

.........................................................................................................................................CTCTTGGACTGAAGGGAGCTCC. 2

.........................................................................................................................................CTCTTGGACTGAAGGGAGCT... 1

..........................................................................................................................................TCTTGGACTGAAGGGAGCTCC. 31

..........................................................................................................................................TCTTGGACTGAAGGGAGCTC.. 1

..........................................................................................................................................TCTTGGACTGAAGGGAGCT... 1

...........................................................................................................................................CTTGGACTGAAGGGAGCTCC. 2273

...........................................................................................................................................CTTGGACTGAAGGGAGCTC.. 56

...........................................................................................................................................CTTGGACTGAAGGGAGCTCCT 108

............................................................................................................................................TTGGACTGAAGGGAGCTCCT 4

............................................................................................................................................TTGGACTGAAGGGAGCTCC. 36

>ppt-MIR319c_MI0003498_Physcomitrella_patens_miR319c_stem-loop GSM313215

UAACCUCACUGGCUGUGGGAGCUUCCUUCGGUUCAAUAGUGGCUGAUAUGAGGUUGCACUGCUGCCGACUCAAACUUCCGGCUUCCCUCUCUUAGAAUGGCAGGGAAUCCGAAUGUCUGAUGCGGGAGCUGAGCGGUCUUCAACUCAGCUUCUCUUGGACUGAAGGGAGCUCCCAUGUCUUUGUGGUUA

(((((.((..(((.((((((((((((((((((((((.((.((((((..(((((((((.(.(((.(((..(((.((...(((.((((((.((.......)).)))))).)))...)).)))..))).))).).)))).)))))..)))))).)).)))))))))))))))))))))))))..)).))))) (-99.10)

...............TGGGAGCTTCCTTCGGTTCAA......................................................................................................................................................... 1

................GGGAGCTTCCTTCGGTTCAAT........................................................................................................................................................ 1

..................GAGCTTCCTTCGGTTCAATA....................................................................................................................................................... 191

..................GAGCTTCCTTCGGTTCAATAGT..................................................................................................................................................... 277

..................GAGCTTCCTTCGGTTCAATAG...................................................................................................................................................... 2551

..................GAGCTTCCTTCGGTTCAAT........................................................................................................................................................ 18

...................AGCTTCCTTCGGTTCAATAG...................................................................................................................................................... 214

...................AGCTTCCTTCGGTTCAATAGT..................................................................................................................................................... 53

...................AGCTTCCTTCGGTTCAATA....................................................................................................................................................... 19

.........................CTTCGGTTCAATAGTGGCTGA............................................................................................................................................... 1

..........................TTCGGTTCAATAGTGGCTGA............................................................................................................................................... 2

......................................GTGGCTGATATGAGGTTGCAC.................................................................................................................................. 5

.......................................TGGCTGATATGAGGTTGCACTG................................................................................................................................ 1

.......................................TGGCTGATATGAGGTTGCAC.................................................................................................................................. 1606

.......................................TGGCTGATATGAGGTTGCA................................................................................................................................... 23

.......................................TGGCTGATATGAGGTTGCACT................................................................................................................................. 13

........................................GGCTGATATGAGGTTGCACT................................................................................................................................. 1

........................................GGCTGATATGAGGTTGCAC.................................................................................................................................. 10

..........................................CTGATATGAGGTTGCACTGC............................................................................................................................... 1

...........................................................TGCTGCCGACTCAAACTTCCG............................................................................................................. 5

...........................................................TGCTGCCGACTCAAACTTCC.............................................................................................................. 4

................................................................................GCTTCCCTCTCTTAGAATGGCA....................................................................................... 3

...............................................................................................................AATGTCTGATGCGGGAGCTGA......................................................... 43

...............................................................................................................AATGTCTGATGCGGGAGCTG.......................................................... 1

.................................................................................................................................TGAGCGGTCTTCAACTCAGCT....................................... 2

.................................................................................................................................TGAGCGGTCTTCAACTCAGC........................................ 1

..................................................................................................................................GAGCGGTCTTCAACTCAGCTTC..................................... 1

....................................................................................................................................GCGGTCTTCAACTCAGCTTC..................................... 26

....................................................................................................................................GCGGTCTTCAACTCAGCTTCT.................................... 193

....................................................................................................................................GCGGTCTTCAACTCAGCTT...................................... 4

...................................................................................................................................................GCTTCTCTTGGACTGAAGGGAGCTC................. 1

...................................................................................................................................................GCTTCTCTTGGACTGAAGGGAGCTCC................ 1

......................................................................................................................................................TCTCTTGGACTGAAGGGAGC................... 2

......................................................................................................................................................TCTCTTGGACTGAAGGGAGCT.................. 19

.......................................................................................................................................................CTCTTGGACTGAAGGGAGCTCC................ 2

.......................................................................................................................................................CTCTTGGACTGAAGGGAGCT.................. 1

........................................................................................................................................................TCTTGGACTGAAGGGAGCTCCC............... 14

........................................................................................................................................................TCTTGGACTGAAGGGAGCTCC................ 31

........................................................................................................................................................TCTTGGACTGAAGGGAGCTC................. 1

........................................................................................................................................................TCTTGGACTGAAGGGAGCT.................. 1

.........................................................................................................................................................CTTGGACTGAAGGGAGCTCC................ 2273

.........................................................................................................................................................CTTGGACTGAAGGGAGCTC................. 56

.........................................................................................................................................................CTTGGACTGAAGGGAGCTCCCA.............. 13

.........................................................................................................................................................CTTGGACTGAAGGGAGCTCCC............... 11233

..........................................................................................................................................................TTGGACTGAAGGGAGCTCCCA.............. 2

..........................................................................................................................................................TTGGACTGAAGGGAGCTCCC............... 179

..........................................................................................................................................................TTGGACTGAAGGGAGCTCC................ 36

...........................................................................................................................................................TGGACTGAAGGGAGCTCCC............... 9

...........................................................................................................................................................TGGACTGAAGGGAGCTCCCA.............. 1

>ppt-MIR538c_MI0003512_Physcomitrella_patens_miR538c_stem-loop GSM313215

UUACAAAGAGUUCUUGAGUCUCCAUGCUUCUCUGACGUUGCAUGGAGUCUAUGUCUGGACCUUCUCCAUUUCCUCUUUCAAGAGAAUGUGAGCAUUGAAGGAUUCGGACAUAGCCUUCAUGCAUGCUAGAGUAUCAUGGAGGCCCGUGAACUACUUGUAA

.(((((..(((((.((.(((((((((...(((((.(((.((((((((.((((((((((((((((.((((...((((....))))...))).)....))))).))))))))))).))))))))))).)))))...))))))))).)).)))))..))))). (-73.90)

.......GAGTTCTTGAGTCTCCATGCT.................................................................................................................................... 1

........AGTTCTTGAGTCTCCATGCT.................................................................................................................................... 1

................AGTCTCCATGCTTCTCTGACGT.......................................................................................................................... 2

................AGTCTCCATGCTTCTCTGACG........................................................................................................................... 22

................AGTCTCCATGCTTCTCTGAC............................................................................................................................ 2

.............................CTCTGACGTTGCATGGAGTCT.............................................................................................................. 1

...................................CGTTGCATGGAGTCTATGTC......................................................................................................... 1

...................................CGTTGCATGGAGTCTATGTCT........................................................................................................ 1

.....................................TTGCATGGAGTCTATGTCTGG...................................................................................................... 16

.....................................TTGCATGGAGTCTATGTCTG....................................................................................................... 2

.....................................TTGCATGGAGTCTATGTCT........................................................................................................ 1

.....................................TTGCATGGAGTCTATGTCTGGAC.................................................................................................... 5

.....................................TTGCATGGAGTCTATGTCTGGA..................................................................................................... 1749

......................................TGCATGGAGTCTATGTCTGGAC.................................................................................................... 2

......................................TGCATGGAGTCTATGTCTGG...................................................................................................... 29

......................................TGCATGGAGTCTATGTCTGGA..................................................................................................... 413

.......................................GCATGGAGTCTATGTCTGG...................................................................................................... 1

.......................................GCATGGAGTCTATGTCTGGA..................................................................................................... 12

........................................CATGGAGTCTATGTCTGGA..................................................................................................... 1

...................................................................ATTTCCTCTTTCAAGAGAATGTGAGC................................................................... 1

.....................................................................................................ATTCGGACATAGCCTTCATGC...................................... 1

........................................................................................................CGGACATAGCCTTCATGCATGC.................................. 2

........................................................................................................CGGACATAGCCTTCATGCAT.................................... 14

........................................................................................................CGGACATAGCCTTCATGCATG................................... 182

.........................................................................................................GGACATAGCCTTCATGCATG................................... 1

.............................................................................................................................CTAGAGTATCATGGAGGCC................ 39

.............................................................................................................................CTAGAGTATCATGGAGGCCC............... 6

.............................................................................................................................CTAGAGTATCATGGAGGCCCG.............. 5596

.............................................................................................................................CTAGAGTATCATGGAGGCCCGT............. 53

..............................................................................................................................TAGAGTATCATGGAGGCCCG.............. 140

..............................................................................................................................TAGAGTATCATGGAGGCCCGT............. 1

..............................................................................................................................TAGAGTATCATGGAGGCCC............... 1

...............................................................................................................................AGAGTATCATGGAGGCCCG.............. 5

.....................................................................................................................................TCATGGAGGCCCGTGAACTAC...... 5

......................................................................................................................................CATGGAGGCCCGTGAACTACT..... 1

>ppt-MIR319e_MI0005665_Physcomitrella_patens_miR319e_stem-loop GSM313215

CUAGCCGUGGGAGCUCCUUCCGGUUCAAUAGUGGCUGAUGUGAGGUUGCACUGCUGCCGAUUCAAACUUCCGGCUUCCCUUUCUUAACACGACAGGGAAUCCGAAUGUCUGAUGCGGGAGCUGUGCGGUCUUCAACUUUAGCGCCUCUUGGACUGAAGGGAGCUCCCAAGUCUU

.......((((((((((((.((((((((.((..(((((..(((((((((((.(((.(((..(((.((...(((.((((((.((.......)).)))))).)))...)).)))..))).))).)))))).)))))...)))))..)).)))))))).))))))))))))...... (-86.10)

........GGGAGCTCCTTCCGGTTCAAT................................................................................................................................................. 1

..........GAGCTCCTTCCGGTTCAATA................................................................................................................................................ 3

..........GAGCTCCTTCCGGTTCAATAGT.............................................................................................................................................. 1

..........GAGCTCCTTCCGGTTCAAT................................................................................................................................................. 2

..........GAGCTCCTTCCGGTTCAATAG............................................................................................................................................... 9

...........AGCTCCTTCCGGTTCAATAG............................................................................................................................................... 2

.......................................................................................................AATGTCTGATGCGGGAGCTGT.................................................. 2

.......................................................................................................AATGTCTGATGCGGGAGCTG................................................... 1

................................................................................................................................................CTCTTGGACTGAAGGGAGCTCC........ 2

................................................................................................................................................CTCTTGGACTGAAGGGAGCT.......... 1

.................................................................................................................................................TCTTGGACTGAAGGGAGCTCCC....... 14

.................................................................................................................................................TCTTGGACTGAAGGGAGCTCC........ 31

.................................................................................................................................................TCTTGGACTGAAGGGAGCTC......... 1

.................................................................................................................................................TCTTGGACTGAAGGGAGCT.......... 1

..................................................................................................................................................CTTGGACTGAAGGGAGCTCC........ 2273

..................................................................................................................................................CTTGGACTGAAGGGAGCTC......... 56

..................................................................................................................................................CTTGGACTGAAGGGAGCTCCCA...... 13

..................................................................................................................................................CTTGGACTGAAGGGAGCTCCC....... 11233

...................................................................................................................................................TTGGACTGAAGGGAGCTCCCA...... 2

...................................................................................................................................................TTGGACTGAAGGGAGCTCCC....... 179

...................................................................................................................................................TTGGACTGAAGGGAGCTCC........ 36

....................................................................................................................................................TGGACTGAAGGGAGCTCCC....... 9

....................................................................................................................................................TGGACTGAAGGGAGCTCCCA...... 1

.......................................................................................................................................................ACTGAAGGGAGCTCCCAAGT... 1

>ppt-MIR893_MI0005676_Physcomitrella_patens_miR893_stem-loop GSM313215

CUCUGCAUUGCUCUCAAACCUCCUCCCUCUUCCUUGUCCUAGCAUAGAUCCCAGUCCCUUCUUCCUUGUCUUGAUGCACGACCCAAUCCUCUCCUCUUCUUCUUUAUCCCGUCAUACCCAUACUAAGACAAGAAAGAGUUGGAAGAGAGAAUUGGGACUUGUGCUGGGACAAGGAAGAAAGGAACUACUACUUGCACCAAGACAAGGAAAAGGAGGAGGA

..................((((((((...(((((((((...(((.((.......(((.((((((((((((((...((((((((((((.(((((.(((..((((((.....(((.............)))...))))))..))).))))).))))))..)))))).)))))))))))))).))).......))))).....)))))))))..)))))))). (-89.76)

..........CTCTCAAACCTCCTCCCTCTTCCTTG........................................................................................................................................................................................ 2

....................................TCCTAGCATAGATCCCAGTCC................................................................................................................................................................... 5

.............................................................................ACGACCCAATCCTCTCCTCTT.......................................................................................................................... 1

.....................................................................................................................................................AATTGGGACTTGTGCTGGGACA................................................. 1

......................................................................................................................................................ATTGGGACTTGTGCTGGGAC.................................................. 1

......................................................................................................................................................ATTGGGACTTGTGCTGGGACA................................................. 8

.........................................................................................................................................................GGGACTTGTGCTGGGACAAGGAA............................................ 1

0)

>ppt-MIR896_MI0005680_Physcomitrella_patens_miR896_stem-loop GSM313215

CCCACCCUGAGCGGGGGGGUCUGAUUGUGGUCGGUCGAGGGGCGCUGACCGUGUGGCCCAAUGGAUAAGGCGCUUGCCUACGGAGCAAGAGAUUCUGGGUUCGAUCCCCAGCAUGGUCGUGGGUCGCCGGAAUUGUAGAUUGUGGCGCGUGUCAAUUUGGCCGAGUGGUUAAGGCGGCAGACUCGAAAUCUGCUGGGGUUUCCCCGCGCAGGUUCAAAUCCUGCA

.....((((.((((((((..((.(..(..(....(((((.(.(((((((((..(((((.(((.((((..(((((((((....).)))...((((((((...((((((((.....))....)))))))))))))).........))))).)))).))).)))))..)))))..)))).)...)))))...)..)).))..)))))))).))))............. (-94.10)

...............GGGGTCTGATTGTGGTCGGTC............................................................................................................................................................................................. 1

.............................................TGACCGTGTGGCCCAATGGATAA............................................................................................................................................................. 1

..............................................GACCGTGTGGCCCAATGGA................................................................................................................................................................ 70

..............................................GACCGTGTGGCCCAATGGATAAGGC.......................................................................................................................................................... 3

..............................................GACCGTGTGGCCCAATGGATAAG............................................................................................................................................................ 4

..............................................GACCGTGTGGCCCAATGGATAAGG........................................................................................................................................................... 8

..............................................GACCGTGTGGCCCAATGGAT............................................................................................................................................................... 19

..............................................GACCGTGTGGCCCAATGGATAA............................................................................................................................................................. 16

..............................................GACCGTGTGGCCCAATGGATA.............................................................................................................................................................. 22

...............................................ACCGTGTGGCCCAATGGATAA............................................................................................................................................................. 1

.................................................CGTGTGGCCCAATGGATAAG............................................................................................................................................................ 1

...................................................TGTGGCCCAATGGATAAGG........................................................................................................................................................... 1

.....................................................TGGCCCAATGGATAAGGCGCTTGCCT.................................................................................................................................................. 1

.........................................................CCAATGGATAAGGCGCTTGCCT.................................................................................................................................................. 2

..........................................................CAATGGATAAGGCGCTTGC.................................................................................................................................................... 1

..........................................................CAATGGATAAGGCGCTTGCCT.................................................................................................................................................. 5

.................................................................................GGAGCAAGAGATTCTGGGTTC........................................................................................................................... 2

..................................................................................GAGCAAGAGATTCTGGGTTC........................................................................................................................... 2

...................................................................................AGCAAGAGATTCTGGGTTC........................................................................................................................... 2

....................................................................................GCAAGAGATTCTGGGTTCG.......................................................................................................................... 2

...........................................................................................ATTCTGGGTTCGATCCCCA................................................................................................................... 1

............................................................................................................................CGCCGGAATTGTAGATTGTGGCGC............................................................................. 1

............................................................................................................................CGCCGGAATTGTAGATTGTGGCGCGT........................................................................... 1

.............................................................................................................................GCCGGAATTGTAGATTGTGGC............................................................................... 1

.............................................................................................................................GCCGGAATTGTAGATTGTGGCGC............................................................................. 18

.............................................................................................................................GCCGGAATTGTAGATTGTGGCG.............................................................................. 7

.............................................................................................................................GCCGGAATTGTAGATTGTGG................................................................................ 1

.............................................................................................................................GCCGGAATTGTAGATTGTGGCGCGT........................................................................... 3

.............................................................................................................................GCCGGAATTGTAGATTGTGGCGCG............................................................................ 1

.............................................................................................................................GCCGGAATTGTAGATTGTG................................................................................. 1

...............................................................................................................................CGGAATTGTAGATTGTGGCGCGT........................................................................... 1

......................................................................................................................................................GTCAATTTGGCCGAGTGGTTAAGG................................................... 20

......................................................................................................................................................GTCAATTTGGCCGAGTGGTTA...................................................... 70

......................................................................................................................................................GTCAATTTGGCCGAGTGGTTAAGGC.................................................. 8

......................................................................................................................................................GTCAATTTGGCCGAGTGGTT....................................................... 91

......................................................................................................................................................GTCAATTTGGCCGAGTGGTTAA..................................................... 16

......................................................................................................................................................GTCAATTTGGCCGAGTGGTTAAG.................................................... 29

......................................................................................................................................................GTCAATTTGGCCGAGTGGT........................................................ 11

.........................................................................................................................................................AATTTGGCCGAGTGGTTAAG.................................................... 1

.........................................................................................................................................................AATTTGGCCGAGTGGTTAA..................................................... 2

..........................................................................................................................................................ATTTGGCCGAGTGGTTAAG.................................................... 1

..........................................................................................................................................................ATTTGGCCGAGTGGTTAAGG................................................... 1

..................................................................................................................................................................GAGTGGTTAAGGCGGCAGA............................................ 1

...................................................................................................................................................................AGTGGTTAAGGCGGCAGACTCG........................................ 1

......................................................................................................................................................................................TCGAAATCTGCTGGGGTTTC....................... 1

......................................................................................................................................................................................TCGAAATCTGCTGGGGTTTCC...................... 1

.......................................................................................................................................................................................CGAAATCTGCTGGGGTTTC....................... 1

.......................................................................................................................................................................................CGAAATCTGCTGGGGTTTCCCCG................... 1

.......................................................................................................................................................................................CGAAATCTGCTGGGGTTTCCCCGC.................. 1

.......................................................................................................................................................................................CGAAATCTGCTGGGGTTTCC...................... 2

........................................................................................................................................................................................GAAATCTGCTGGGGTTTCCC..................... 1

>ppt-MIR2083_MI0010537_Physcomitrella_patens_miR2083_stem-loop GSM313215

AAAAGUGUGUGUAUGCGGAAGUUGGAGAAAACCCAGACUUCAUUGCUCGAUGAAGCAUGUAGCUUCGCAGUCGUACUUCUUGCACUCCUCCAUCUCUCGCAUCUCUUGCGCAUUGUGGUUCUCCAACACCUGCACAGCGGCGGUCUUGUCCGGAUUAGGGAUCGUCUACACCAGAUAAGCCACGGCGUUUUUCUCGGAGAAAAAUACCAUCCUUUGGUUCAACAACGGUUGCUGCGUUUUCCACGUUCGGAUACAACCCUGCCCAGUUGCUGUGAGAGAUUGACGAGUGCAAGAAGACCCUCUUUCCAUGUUCCAGACGCA

....((((.((((((.(((((..((.(((....((..(((((((....)))))))..))....))).........((((((((((((.((.((((((((((.(..(((.(((.(((((....)).)))..)))...((((.(((..(((((((((...(((.((.......(((...((((..((..((((((.....))))))..)).....(((......))).)))).)))))...)))..)))))))))..))))))).)))..).)))))))))).)).)))))))))))).))..)))))))))...)).)))). (-109.51)

.....................................................................GTCGTACTTCTTGCACTCCT........................................................................................................................................................................................................................................ 1

............................................................................TTCTTGCACTCCTCCATCTCT................................................................................................................................................................................................................................ 146

............................................................................TTCTTGCACTCCTCCATCTC................................................................................................................................................................................................................................. 2

.............................................................................TCTTGCACTCCTCCATCTCTC............................................................................................................................................................................................................................... 3

..............................................................................CTTGCACTCCTCCATCTCT................................................................................................................................................................................................................................ 1

..........................................................................................................................CCAACACCTGCACAGCGGCGG.................................................................................................................................................................................. 1

.......................................................................................................................................................................ACACCAGATAAGCCACGGCGT..................................................................................................................................... 1

..................................................................................................................................................................................................................................................ACGTTCGGATACAACCCTGCC.......................................................... 1

.............................................................................................................................................................................................................................................................................CTGTGAGAGATTGACGAGTGC............................... 2

..............................................................................................................................................................................................................................................................................TGTGAGAGATTGACGAGTGCA.............................. 23

..............................................................................................................................................................................................................................................................................TGTGAGAGATTGACGAGTGC............................... 1

....................................................................................................................................................................................................................................................................................AGATTGACGAGTGCAAGAAGAC....................... 1

....................................................................................................................................................................................................................................................................................AGATTGACGAGTGCAAGAA.......................... 2

....................................................................................................................................................................................................................................................................................AGATTGACGAGTGCAAGAAGA........................ 151

.......................................................................................................................................................................................................................................................................................TTGACGAGTGCAAGAAGACCC..................... 1

>ppt-MIR390a_MI0003494_Physcomitrella_patens_miR390a_stem-loop GSM313215

CAUGAUACAAUUACGAAGCUCAGGAGGGAUAGCGCCAUUCUCUGUUCUUCUCUAUCGACACUUGGUAAACCAGCUUGUGCUAGCUAUGGGCAGAGAUAUGGCGUUAUCCAUUCUGAGCUUUGCAAGUGUCUCGUGCU

(((((.(((.((.((((((((((((.(((((((((((((((((((((....(((.(.(((.((((....))))..)))).)))....))))))))).)))))))))))).)))))))))))).)).))).))))).. (-66.00)

...............AAGCTCAGGAGGGATAGCGC...................................................................................................... 5

...............AAGCTCAGGAGGGATAGCGCC..................................................................................................... 583

................AGCTCAGGAGGGATAGCGCC..................................................................................................... 26

................AGCTCAGGAGGGATAGCGCCA.................................................................................................... 7

.................GCTCAGGAGGGATAGCGCC..................................................................................................... 1

....................................ATTCTCTGTTCTTCTCTATCGA............................................................................... 1

.....................................................................................................CGTTATCCATTCTGAGCTTT................ 6

.....................................................................................................CGTTATCCATTCTGAGCTTTG............... 12

.......................................................................................................TTATCCATTCTGAGCTTTGCA............. 4

........................................................................................................TATCCATTCTGAGCTTTGCAA............ 2

........................................................................................................TATCCATTCTGAGCTTTGCA............. 1

>ppt-MIR534a_MI0003501_Physcomitrella_patens_miR534a_stem-loop GSM313215

AUAUGCAUGCAACUUGUGUGGACAGACUGACUAGUCUAGUGGUGUAGUGGAAAGUAUUAGUUGGUUGAUGCACGAUGUUGUUUUGCAACAAGUGACUCGUCGCUGCAAUACUGAACCCACAAGUUUUGGAAGUUGUUUUGAUUGGGGCCACCCACAUUGCUCGACUAGAUUCAGUAUGUCCAUUGCAGUUGCAUACAUAU

(((((.((((((((...(((((((.(((((((((((.((..((((.((((..((((((.((.(((.(((((((..(((((.....))))).)))...)))))))))))))))...((((((((.............))))..)))).))))..))))..)).))))))..))))).)))))))...)))))))).))))) (-75.32)

.....CATGCAACTTGTGTGGACAGAC............................................................................................................................................................................. 25

.....CATGCAACTTGTGTGGACAGACT............................................................................................................................................................................ 2

.....CATGCAACTTGTGTGGACAGA.............................................................................................................................................................................. 5

......ATGCAACTTGTGTGGACAGA.............................................................................................................................................................................. 3582

......ATGCAACTTGTGTGGACAG............................................................................................................................................................................... 11

......ATGCAACTTGTGTGGACAGACT............................................................................................................................................................................ 3875

......ATGCAACTTGTGTGGACAGAC............................................................................................................................................................................. 6301

......ATGCAACTTGTGTGGACAGACTG........................................................................................................................................................................... 1

.......TGCAACTTGTGTGGACAGACT............................................................................................................................................................................ 178

.......TGCAACTTGTGTGGACAGAC............................................................................................................................................................................. 82

.......TGCAACTTGTGTGGACAGACTG........................................................................................................................................................................... 3

.......TGCAACTTGTGTGGACAGA.............................................................................................................................................................................. 17

........GCAACTTGTGTGGACAGACT............................................................................................................................................................................ 2

........GCAACTTGTGTGGACAGAC............................................................................................................................................................................. 1

...........................TGACTAGTCTAGTGGTGTAG......................................................................................................................................................... 1

...........................TGACTAGTCTAGTGGTGTAGT........................................................................................................................................................ 3

............................GACTAGTCTAGTGGTGTAGTGGAA.................................................................................................................................................... 1

..............................................................................................................................................................................TATGTCCATTGCAGTTGCATA..... 58

..............................................................................................................................................................................TATGTCCATTGCAGTTGCATACA... 4

..............................................................................................................................................................................TATGTCCATTGCAGTTGCATAC.... 863

..............................................................................................................................................................................TATGTCCATTGCAGTTGCAT...... 1

..............................................................................................................................................................................TATGTCCATTGCAGTTGCA....... 3

...............................................................................................................................................................................ATGTCCATTGCAGTTGCAT...... 1

...............................................................................................................................................................................ATGTCCATTGCAGTTGCATAC.... 283

...............................................................................................................................................................................ATGTCCATTGCAGTTGCATACA... 1

...............................................................................................................................................................................ATGTCCATTGCAGTTGCATA..... 9

>ppt-MIR538a_MI0003510_Physcomitrella_patens_miR538a_stem-loop GSM313215

UUACAAUGAGUUUGUGAGUCUCCAUGCUGCUCUAACAUUGCAUGGAGUCUAUGUCUGGAGUAUUGUCCUUCAUUCCCGUGCAAAUGCUGAAGUAAUGCAAGAAUGUGAACAUUGGGACUAUUCCAGACGUAGCCUUCAUGCAUGUUAGAGUGAUGUGGAAACCCGUGAACUACUUGUAA

.(((((..((((..((.((.((((((..((((((((((.((((((((.((((((((((((((..(((((.(((((...((((..(((....))).)))).)))))........))))))))))))))))))).))))))))))))))))))..)))))).)).))..))))..))))). (-83.50)

................AGTCTCCATGCTGCTCTAACA.............................................................................................................................................. 1

.................GTCTCCATGCTGCTCTAACATT............................................................................................................................................ 1

.................GTCTCCATGCTGCTCTAACA.............................................................................................................................................. 2

.................GTCTCCATGCTGCTCTAACAT............................................................................................................................................. 16

..................TCTCCATGCTGCTCTAACATT............................................................................................................................................ 2

..............................TCTAACATTGCATGGAGTCTA................................................................................................................................ 1

................................TAACATTGCATGGAGTCTATG.............................................................................................................................. 6

................................TAACATTGCATGGAGTCTATGT............................................................................................................................. 5

................................TAACATTGCATGGAGTCTATGTC............................................................................................................................ 9

....................................ATTGCATGGAGTCTATGTCTGGA........................................................................................................................ 1

.....................................TTGCATGGAGTCTATGTCTGG......................................................................................................................... 16

.....................................TTGCATGGAGTCTATGTCTG.......................................................................................................................... 2

.....................................TTGCATGGAGTCTATGTCT........................................................................................................................... 1

.....................................TTGCATGGAGTCTATGTCTGGA........................................................................................................................ 1749

.....................................TTGCATGGAGTCTATGTCTGGAG....................................................................................................................... 1

......................................TGCATGGAGTCTATGTCTGGAG....................................................................................................................... 12

......................................TGCATGGAGTCTATGTCTGG......................................................................................................................... 29

......................................TGCATGGAGTCTATGTCTGGA........................................................................................................................ 413

.......................................GCATGGAGTCTATGTCTGGAG....................................................................................................................... 2

.......................................GCATGGAGTCTATGTCTGG......................................................................................................................... 1

.......................................GCATGGAGTCTATGTCTGGA........................................................................................................................ 12

........................................CATGGAGTCTATGTCTGGA........................................................................................................................ 1

.........................................ATGGAGTCTATGTCTGGAGTA..................................................................................................................... 1

...............................................................................................................TTGGGACTATTCCAGACGTAGCCTTC.......................................... 1

........................................................................................................................TTCCAGACGTAGCCTTCATGC...................................... 7

..........................................................................................................................CCAGACGTAGCCTTCATGCAT.................................... 5

...........................................................................................................................CAGACGTAGCCTTCATGCAT.................................... 8

...........................................................................................................................CAGACGTAGCCTTCATGCATG................................... 10

............................................................................................................................AGACGTAGCCTTCATGCATG................................... 14

............................................................................................................................AGACGTAGCCTTCATGCATGT.................................. 10

...............................................................................................................................CGTAGCCTTCATGCATGTTAGA.............................. 1

................................................................................................................................GTAGCCTTCATGCATGTTAGA.............................. 10

.................................................................................................................................TAGCCTTCATGCATGTTAGA.............................. 2

..................................................................................................................................AGCCTTCATGCATGTTAGAGT............................ 1

....................................................................................................................................CCTTCATGCATGTTAGAGTG........................... 3

.......................................................................................................................................TCATGCATGTTAGAGTGATGTGGAAA.................. 1

.......................................................................................................................................TCATGCATGTTAGAGTGATG........................ 1

..........................................................................................................................................TGCATGTTAGAGTGATGTGGA.................... 1

...........................................................................................................................................GCATGTTAGAGTGATGTGGAAA.................. 1

............................................................................................................................................CATGTTAGAGTGATGTGGAAA.................. 5

............................................................................................................................................CATGTTAGAGTGATGTGGAAAC................. 5

.............................................................................................................................................ATGTTAGAGTGATGTGGAAAC................. 1

.............................................................................................................................................ATGTTAGAGTGATGTGGAAACC................ 1

...............................................................................................................................................GTTAGAGTGATGTGGAAACCC............... 191

...............................................................................................................................................GTTAGAGTGATGTGGAAAC................. 2

...............................................................................................................................................GTTAGAGTGATGTGGAAACC................ 21

................................................................................................................................................TTAGAGTGATGTGGAAACCCG.............. 245

................................................................................................................................................TTAGAGTGATGTGGAAACCC............... 3

.................................................................................................................................................TAGAGTGATGTGGAAACCCG.............. 2

..................................................................................................................................................AGAGTGATGTGGAAACCCG.............. 1

.....................................................................................................................................................GTGATGTGGAAACCCGTGAAC......... 6

>ppt-MIR1219a_MI0004718_Physcomitrella_patens_miR1219a_stem-loop GSM313215

UGAAGUGUGGACGAUGGAGAGUCAGCCUCUUCCUGCCUCUCACUAGCUUCAUCCCUUCCUCCCUAAAUUUUAGUCUGGGAGGGAAGGAGCUAUUGGUGGUCAGGAAUAGCGCACCCUUCAUUUAUCCACACUUCA

.((((((((((.(((((((.((..((((.((((((((.(.((.(((((((.((((((((...(((.....)))...)))))))).))))))).))).)).)))))).)).)))).)))))))..)))))))))). (-67.20)

.....TGTGGACGATGGAGAGTCAGC............................................................................................................. 9

.....TGTGGACGATGGAGAGTCA............................................................................................................... 11

.....TGTGGACGATGGAGAGTCAG.............................................................................................................. 38

.....TGTGGACGATGGAGAGTCAGCCT........................................................................................................... 78

.....TGTGGACGATGGAGAGTCAGCC............................................................................................................ 162

......GTGGACGATGGAGAGTCAGCCT........................................................................................................... 3

......GTGGACGATGGAGAGTCAGCC............................................................................................................ 9

.......TGGACGATGGAGAGTCAGCC............................................................................................................ 5

............................CTTCCTGCCTCTCACTAGCTTC..................................................................................... 1

............................CTTCCTGCCTCTCACTAGCTT...................................................................................... 124

.............................TTCCTGCCTCTCACTAGCTT...................................................................................... 2

........................................................................................GCTATTGGTGGTCAGGAATAG.......................... 45

........................................................................................GCTATTGGTGGTCAGGAATA........................... 5

.........................................................................................CTATTGGTGGTCAGGAATAG.......................... 1

.............................................................................................................CGCACCCTTCATTTATCCA....... 7

.............................................................................................................CGCACCCTTCATTTATCCACAC.... 1

.............................................................................................................CGCACCCTTCATTTATCCACACT... 4

>ppt-MIR1030f_MI0005980_Physcomitrella_patens_miR1030f_stem-loop GSM313215

CCUCUACAUAACUACUCCAAUGCCACGUAUCUGCAUCUGCACCUGCACCAAAGUUUUGUAAUUCUACGGCUCGUGGUAGUCUGUUGUUGUGAAGGUGGGAUGUAGCUGAGUGUAUUACUCGGUGUUCAUUUGAUCAUUAUUACCCGAUGGUGCGGGUUCUUAUGCAGAGCCGUGGCAUUGGAGUAAUCCUCUACUU

............(((((((((((((((..(((((((..(.((((((((((.....(((((....))))).(((.((((..........((((.(((.(((((..((((((((...))))))))...))))).))).))))))))))))))))))))).)..)))))))..)))))))))))))))........... (-82.00)

.............ACTCCAATGCCACGTATCTGC.................................................................................................................................................................. 3

.............................TCTGCATCTGCACCTGCACCA.................................................................................................................................................. 68

.............................TCTGCATCTGCACCTGCAC.................................................................................................................................................... 3

.............................TCTGCATCTGCACCTGCACC................................................................................................................................................... 1

..............................CTGCATCTGCACCTGCACCA.................................................................................................................................................. 2

.....................................................................................................................................................GTGCGGGTTCTTATGCAGAG........................... 2

.....................................................................................................................................................GTGCGGGTTCTTATGCAGAGC.......................... 3

......................................................................................................................................................TGCGGGTTCTTATGCAGAGC.......................... 1

.....................................................................................................................................................................AGAGCCGTGGCATTGGAGTAA.......... 3

>ppt-MIR1063d_MI0006025_Physcomitrella_patens_miR1063d_stem-loop GSM313215

ACUACACUUAUUGCCUUUUUGCCCACCCUCAUCUUGGAGUACUGCAUCUUCAUGUCGGCUGCUUUUCAUGUCUGGCUACGAGGUUGCUCAUGUGAUUAGGUUUGAUCCAUGUUAGGCUUUAGGUUGUAUCAGUGAAUUAGUGUCGACAGUUAGGAGUGAAACUUUUUUUAGCUGUUGAUACGCGGGAGUUAUGGGAUUCUCAUUUCAACACAAUGUAAUCUUGUGUCAAUCCAGCCUAUGCUUGUAGAGUUACUGAGUCACAUGAGCACAUCGAAUGCCUUUGAGGCUUUGAAAUUCUACCGACGAGUAGAUGCAGAACUACAAGAAGCGAGUGGGUAACAGGCUAUUUUUCAAGA

............((((..((((((((.(.(.(((((.(((.((((((((.(.((((((..(..(((((.(((((((..(((...(((((((((((((.(((....((.....(((((..(((((((......((((...(((((((((((((((((........)))))))))))))))))..(((((((....)))))))..))))((((((.......)))))).....))))))).)))))..))...))).)))))))))))))..)))...)))....))))..)))))..)..)))))).).)))))))).))).))))).).).)))))))).))))............ (-134.90)

.............................CATCTTGGAGTACTGCATCTT.................................................................................................................................................................................................................................................................................................................. 36

.............................CATCTTGGAGTACTGCATCTTC................................................................................................................................................................................................................................................................................................................. 3

.............................CATCTTGGAGTACTGCATCT................................................................................................................................................................................................................................................................................................................... 2

................................................................................................................................................................................................................................................................................................TTGAAATTCTACCGACGAGTA............................................... 1

.....................................................................................................................................................................................................................................................................................................................GATGCAGAACTACAAGAAGC........................... 11

......................................................................................................................................................................................................................................................................................................................ATGCAGAACTACAAGAAGCG.......................... 1

>ppt-MIR1063g_MI0006028_Physcomitrella_patens_miR1063g_stem-loop GSM313215

GACUGCGAGUUCGCUUUGUUGCUCACCCUCAUCUUGGAGUACUGCAUCUUCACAUCUCUGGGUUUCACAAUCUGCCUCAGGUUGUUAAAGUGCCGGAGUAAAAUUGUGUUAUUGGAUAUUCAGUAAAGAUGGGUGCGAAAAUGGAGUCAAUCCAUCAUUUGCAUAGUACUAGAGAUAAAGUGUUUACACACUUAUCAUAGUACAAAACUCGGGGCACUUGAGCACACCAUAUGGAAAGAAGGUUUGAAAUUCUUGUGAUGGGCAGAUGCAGAACUGCAAGAAGUGAGUGGGCAACAGGCUAUUUUCGAAGA

..((.((((...((((.(((((((((.(.(.(((((.(((.((((((((((.((((.(.((((((((.(((((.((...(((((((.(((((((.((((...(((...(((((((....))))))).)))..(((((((.(((((.....)))))..))))))).((((((..(((((.((((....))))))))).))))))...))))..))))))).)))).)))....)).....)))))))))))))..).)))))).)))))))).))).))))).).).)))))))))))))....)))).)). (-116.30)

......................TCACCCTCATCTTGGAGTACT............................................................................................................................................................................................................................................................................ 1

.............................CATCTTGGAGTACTGCATCTT..................................................................................................................................................................................................................................................................... 36

.............................CATCTTGGAGTACTGCATCTTC.................................................................................................................................................................................................................................................................... 3

.............................CATCTTGGAGTACTGCATCT...................................................................................................................................................................................................................................................................... 2

.................................TTGGAGTACTGCATCTTCACA................................................................................................................................................................................................................................................................. 2

..................................TGGAGTACTGCATCTTCACAT................................................................................................................................................................................................................................................................ 1

..................................................CACATCTCTGGGTTTCACAATC............................................................................................................................................................................................................................................... 1

..................................................................................................................................................................................................................................................TTTGAAATTCTTGTGATGGGCA............................................... 1

......................................................................................................................................................................................................................................................................CAGATGCAGAACTGCAAGAAG............................ 1

........................................................................................................................................................................................................................................................................GATGCAGAACTGCAAGAAGTG.......................... 47

........................................................................................................................................................................................................................................................................GATGCAGAACTGCAAGAAGT........................... 634

........................................................................................................................................................................................................................................................................GATGCAGAACTGCAAGAAG............................ 3

.........................................................................................................................................................................................................................................................................ATGCAGAACTGCAAGAAGT........................... 1

>ppt-MIR390b_MI0003495_Physcomitrella_patens_miR390b_stem-loop GSM313216 3

AUCAAGCUGCUGGGAGAUACAAUUACGAAGCUCAGGAGGGAUAGCGCCCAGCCCUGCUUUUACUGUUCAAUCAGUUAGAGUGCUUCUGAUAGUAAGAUGCAGAUGCAUGACGCUAUCCAUUCUGAGCUUUGCAACUGUGUCCCCCUAAUUUGGGCAACCGGAUCGGUUAGCUGGU

((((.(((((((((.((((((.((.((((((((((((.((((((((..((((.(((((((((((((.((...(((......)))..))))))))))).))))..)).)).)))))))).)))))))))))).)).)))))).)))..((((((....)))))).)).)))))))) (-75.80)

........................ACGAAGCTCAGGAGGGATAGC.................................................................................................................................. 2

........................ACGAAGCTCAGGAGGGATAGCG................................................................................................................................. 1

.........................CGAAGCTCAGGAGGGATAGCG................................................................................................................................. 3

...........................AAGCTCAGGAGGGATAGCGC................................................................................................................................ 11

...........................AAGCTCAGGAGGGATAGCG................................................................................................................................. 5

...........................AAGCTCAGGAGGGATAGCGCCC.............................................................................................................................. 3

...........................AAGCTCAGGAGGGATAGCGCC............................................................................................................................... 754

............................AGCTCAGGAGGGATAGCGCC............................................................................................................................... 39

.................................AGGAGGGATAGCGCCCAGCC.......................................................................................................................... 1

..............................................................................AGTGCTTCTGATAGTAAGATGC........................................................................... 1

............................................................................................................GACGCTATCCATTCTGAGCTT.............................................. 2

.............................................................................................................ACGCTATCCATTCTGAGCTTT............................................. 2

..............................................................................................................CGCTATCCATTCTGAGCTTT............................................. 10

..............................................................................................................CGCTATCCATTCTGAGCTT.............................................. 1

..............................................................................................................CGCTATCCATTCTGAGCTTTG............................................ 105

...............................................................................................................GCTATCCATTCTGAGCTTT............................................. 1

...............................................................................................................GCTATCCATTCTGAGCTTTG............................................ 4

.................................................................................................................TATCCATTCTGAGCTTTGCA.......................................... 1

>ppt-MIR319a_MI0003496_Physcomitrella_patens_miR319a_stem-loop GSM313216 5

GUGGAGCUCCGUUUCGGUCCAAUAGUGGCUGCGACGGAAGGUGGUCCCGCUGCCGAAUCACACGUCCGGGUUGCUUAUCGGGGCAGGGCCCCGAUACGGUAUCCGAACGUUUGUCCCGGGAACUGGUCGACCUUCCGCCCGGCGUCUCUUGGACUGAAGGGAGCUCCAC

((((((((((.(((((((((((.((..((((.(.((((((((.(.((.(.(.(((...((.((((.(((((.((((((((((((...))))))))).)))))))).)))).))...))).).).)).).)))))))).)))))..)).))))))))))))))))))))) (-98.80)

..GGAGCTCCGTTTCGGTCCAAT.................................................................................................................................................. 3

..GGAGCTCCGTTTCGGTCCAATA................................................................................................................................................. 4

...GAGCTCCGTTTCGGTCCAATAG................................................................................................................................................ 248

...GAGCTCCGTTTCGGTCCAATAGT............................................................................................................................................... 7

...GAGCTCCGTTTCGGTCCAAT.................................................................................................................................................. 17

...GAGCTCCGTTTCGGTCCAA................................................................................................................................................... 2

...GAGCTCCGTTTCGGTCCAATA................................................................................................................................................. 90

....AGCTCCGTTTCGGTCCAATAG................................................................................................................................................ 429

....AGCTCCGTTTCGGTCCAAT.................................................................................................................................................. 63

....AGCTCCGTTTCGGTCCAATA................................................................................................................................................. 210

....AGCTCCGTTTCGGTCCAATAGT............................................................................................................................................... 70

.....GCTCCGTTTCGGTCCAATAG................................................................................................................................................ 1

.............TCGGTCCAATAGTGGCTGCGA....................................................................................................................................... 1

..................CCAATAGTGGCTGCGACGGAAG................................................................................................................................. 2

........................GTGGCTGCGACGGAAGGTGGTC........................................................................................................................... 1

.........................TGGCTGCGACGGAAGGTGG............................................................................................................................. 2

.........................TGGCTGCGACGGAAGGTGGTC........................................................................................................................... 436

.........................TGGCTGCGACGGAAGGTGGTCC.......................................................................................................................... 1267

.........................TGGCTGCGACGGAAGGTGGT............................................................................................................................ 7

.........................TGGCTGCGACGGAAGGTGGTCCC......................................................................................................................... 42

..........................GGCTGCGACGGAAGGTGGTCC.......................................................................................................................... 11

..........................GGCTGCGACGGAAGGTGGTCCC......................................................................................................................... 1

..........................GGCTGCGACGGAAGGTGGTC........................................................................................................................... 5

...........................GCTGCGACGGAAGGTGGTCC.......................................................................................................................... 1

...........................GCTGCGACGGAAGGTGGTC........................................................................................................................... 2

...............................................CGCTGCCGAATCACACGTCCG..................................................................................................... 1

..............................................................................................................................TCGACCTTCCGCCCGGCGT........................ 2

..............................................................................................................................TCGACCTTCCGCCCGGCGTC....................... 3

..............................................................................................................................TCGACCTTCCGCCCGGCGTCT...................... 16

................................................................................................................................GACCTTCCGCCCGGCGTCT...................... 1

.....................................................................................................................................TCCGCCCGGCGTCTCTTGGA................ 1

................................................................................................................................................TCTCTTGGACTGAAGGGAGC..... 10

................................................................................................................................................TCTCTTGGACTGAAGGGAGCT.... 21

................................................................................................................................................TCTCTTGGACTGAAGGGAGCTC... 1

.................................................................................................................................................CTCTTGGACTGAAGGGAGCTC... 1

..................................................................................................................................................TCTTGGACTGAAGGGAGCTCC.. 15

...................................................................................................................................................CTTGGACTGAAGGGAGCTCCAC 7

...................................................................................................................................................CTTGGACTGAAGGGAGCTCCA. 244

...................................................................................................................................................CTTGGACTGAAGGGAGCTC... 7

...................................................................................................................................................CTTGGACTGAAGGGAGCTCC.. 516

....................................................................................................................................................TTGGACTGAAGGGAGCTCC.. 17

....................................................................................................................................................TTGGACTGAAGGGAGCTCCA. 24

....................................................................................................................................................TTGGACTGAAGGGAGCTCCAC 14

.....................................................................................................................................................TGGACTGAAGGGAGCTCCA. 2

.....................................................................................................................................................TGGACTGAAGGGAGCTCCAC 1

>ppt-MIR319b_MI0003497_Physcomitrella_patens_miR319b_stem-loop GSM313216 6

GAGCUCUUUUCAGUCCAGUAGCAGCUAAUGUCGAAGGUUGUACCGCUGCCGACUCAAACUUCCGGCUUCCAUAUCACGACGCGUGAUAUGGAAUCCGAAGGUCUGAUCCGGGAGCUGAUCGAUCUCCAGGUUAGCAUCUCUUGGACUGAAGGGAGCUCCU

((((((((((((((((((.((..((((((.(.((.(((((....(((.(((..(((.((((.(((.((((((((((((...)))))))))))).))).)))).)))..))).)))....))))))).).))))))..)).)))))))))))))))))).. (-83.70)

GAGCTCTTTTCAGTCCAGT............................................................................................................................................. 1

GAGCTCTTTTCAGTCCAGTA............................................................................................................................................ 10

.AGCTCTTTTCAGTCCAGTA............................................................................................................................................ 8

.AGCTCTTTTCAGTCCAGTAG........................................................................................................................................... 24

.....................CAGCTAATGTCGAAGGTTGTAC..................................................................................................................... 1

...........................................CGCTGCCGACTCAAACTTCCG................................................................................................ 1

........................................................................ATCACGACGCGTGATATGGAATC................................................................. 1

.................................................................................................AAGGTCTGATCCGGGAGCTGA.......................................... 25

.................................................................................................AAGGTCTGATCCGGGAGCT............................................ 2

..................................................................................................AGGTCTGATCCGGGAGCTGA.......................................... 1

..................................................................................................................................TTAGCATCTCTTGGACTGA........... 1

..................................................................................................................................TTAGCATCTCTTGGACTGAA.......... 5

..................................................................................................................................TTAGCATCTCTTGGACTGAAG......... 111

...................................................................................................................................TAGCATCTCTTGGACTGAAG......... 1

.....................................................................................................................................GCATCTCTTGGACTGAAGGGAGCTCC. 1

.......................................................................................................................................ATCTCTTGGACTGAAGGGAGC.... 7

.......................................................................................................................................ATCTCTTGGACTGAAGGGA...... 6

........................................................................................................................................TCTCTTGGACTGAAGGGAGC.... 10

........................................................................................................................................TCTCTTGGACTGAAGGGAGCT... 21

........................................................................................................................................TCTCTTGGACTGAAGGGAGCTC.. 1

.........................................................................................................................................CTCTTGGACTGAAGGGAGCTC.. 1

..........................................................................................................................................TCTTGGACTGAAGGGAGCTCC. 15

...........................................................................................................................................CTTGGACTGAAGGGAGCTCCT 15

...........................................................................................................................................CTTGGACTGAAGGGAGCTC.. 7

...........................................................................................................................................CTTGGACTGAAGGGAGCTCC. 516

............................................................................................................................................TTGGACTGAAGGGAGCTCC. 17

............................................................................................................................................TTGGACTGAAGGGAGCTCCT 2

.............................................................................................................................................TGGACTGAAGGGAGCTCCT 1

>ppt-MIR319c_MI0003498_Physcomitrella_patens_miR319c_stem-loop GSM313216 7

UAACCUCACUGGCUGUGGGAGCUUCCUUCGGUUCAAUAGUGGCUGAUAUGAGGUUGCACUGCUGCCGACUCAAACUUCCGGCUUCCCUCUCUUAGAAUGGCAGGGAAUCCGAAUGUCUGAUGCGGGAGCUGAGCGGUCUUCAACUCAGCUUCUCUUGGACUGAAGGGAGCUCCCAUGUCUUUGUGGUUA

(((((.((..(((.((((((((((((((((((((((.((.((((((..(((((((((.(.(((.(((..(((.((...(((.((((((.((.......)).)))))).)))...)).)))..))).))).).)))).)))))..)))))).)).)))))))))))))))))))))))))..)).))))) (-99.10)

...............TGGGAGCTTCCTTCGGTTCAA......................................................................................................................................................... 1

..................GAGCTTCCTTCGGTTCAATAG...................................................................................................................................................... 789

..................GAGCTTCCTTCGGTTCAATA....................................................................................................................................................... 153

..................GAGCTTCCTTCGGTTCAATAGT..................................................................................................................................................... 98

..................GAGCTTCCTTCGGTTCAAT........................................................................................................................................................ 61

...................AGCTTCCTTCGGTTCAATA....................................................................................................................................................... 10

...................AGCTTCCTTCGGTTCAATAG...................................................................................................................................................... 265

...................AGCTTCCTTCGGTTCAATAGT..................................................................................................................................................... 26

.....................CTTCCTTCGGTTCAATAGT..................................................................................................................................................... 1

.........................CTTCGGTTCAATAGTGGCTGA............................................................................................................................................... 3

..........................TTCGGTTCAATAGTGGCTGA............................................................................................................................................... 9

......................................GTGGCTGATATGAGGTTGCAC.................................................................................................................................. 2

.......................................TGGCTGATATGAGGTTGCA................................................................................................................................... 16

.......................................TGGCTGATATGAGGTTGCAC.................................................................................................................................. 702

.......................................TGGCTGATATGAGGTTGCACT................................................................................................................................. 2

........................................GGCTGATATGAGGTTGCAC.................................................................................................................................. 4

...........................................................TGCTGCCGACTCAAACTTCCG............................................................................................................. 3

...........................................................TGCTGCCGACTCAAACTTCC.............................................................................................................. 2

................................................................................GCTTCCCTCTCTTAGAATGGCA....................................................................................... 1

................................................................................GCTTCCCTCTCTTAGAATGGCAGGGA................................................................................... 1

................................................................................GCTTCCCTCTCTTAGAATGGCAGGG.................................................................................... 1

..................................................................................TTCCCTCTCTTAGAATGGCAGGGA................................................................................... 1

...................................................................................TCCCTCTCTTAGAATGGCAGGGAAT................................................................................. 1

...............................................................................................................AATGTCTGATGCGGGAGCTGA......................................................... 48

.................................................................................................................TGTCTGATGCGGGAGCTGA......................................................... 1

.................................................................................................................................TGAGCGGTCTTCAACTCAGCT....................................... 1

....................................................................................................................................GCGGTCTTCAACTCAGCTTCT.................................... 229

....................................................................................................................................GCGGTCTTCAACTCAGCTT...................................... 6

....................................................................................................................................GCGGTCTTCAACTCAGCTTC..................................... 42

....................................................................................................................................GCGGTCTTCAACTCAGCTTCTC................................... 3

......................................................................................................................................GGTCTTCAACTCAGCTTCT.................................... 1

..................................................................................................................................................AGCTTCTCTTGGACTGAAGGGAGC................... 3

..................................................................................................................................................AGCTTCTCTTGGACTGAAGGG...................... 2

...................................................................................................................................................GCTTCTCTTGGACTGAAGGGAGCTC................. 1

...................................................................................................................................................GCTTCTCTTGGACTGAAGGGAGCT.................. 1

......................................................................................................................................................TCTCTTGGACTGAAGGGAGC................... 10

......................................................................................................................................................TCTCTTGGACTGAAGGGAGCT.................. 21

......................................................................................................................................................TCTCTTGGACTGAAGGGAGCTC................. 1

.......................................................................................................................................................CTCTTGGACTGAAGGGAGCTC................. 1

........................................................................................................................................................TCTTGGACTGAAGGGAGCTCCC............... 3

........................................................................................................................................................TCTTGGACTGAAGGGAGCTCC................ 15

.........................................................................................................................................................CTTGGACTGAAGGGAGCTCCC............... 1811

.........................................................................................................................................................CTTGGACTGAAGGGAGCTCCCA.............. 42

.........................................................................................................................................................CTTGGACTGAAGGGAGCTC................. 7

.........................................................................................................................................................CTTGGACTGAAGGGAGCTCC................ 516

.........................................................................................................................................................CTTGGACTGAAGGGAGCTCCCAT............. 1

..........................................................................................................................................................TTGGACTGAAGGGAGCTCC................ 17

..........................................................................................................................................................TTGGACTGAAGGGAGCTCCCA.............. 3

..........................................................................................................................................................TTGGACTGAAGGGAGCTCCC............... 47

...........................................................................................................................................................TGGACTGAAGGGAGCTCCC............... 12

...........................................................................................................................................................TGGACTGAAGGGAGCTCCCA.............. 4

...........................................................................................................................................................TGGACTGAAGGGAGCTCCCAT............. 1

............................................................................................................................................................GGACTGAAGGGAGCTCCCA.............. 1

>ppt-MIR533a_MI0003500_Physcomitrella_patens_miR533a_stem-loop GSM313216 3

AUGGGGAGCUGGCCAGGCUGUGAGGGACGGAGCAGAGUUGGCUUGUGGCUCCUUGUGGCCCCCUCCAGCAGCUUCCUCCUCACAGUCUGCACAGCUCUCCGU

(((((((((((..(((((((((((((..(((((...(((((...(.((((......)))))...))))).))))).)))))))))))))..))))))))))) (-59.10)

.....GAGCTGGCCAGGCTGTGAGGGAC.......................................................................... 4

.....GAGCTGGCCAGGCTGTGAGG............................................................................. 3

.....GAGCTGGCCAGGCTGTGAGGGA........................................................................... 79

.....GAGCTGGCCAGGCTGTGAGGG............................................................................ 43

......AGCTGGCCAGGCTGTGAGGGA........................................................................... 23

......AGCTGGCCAGGCTGTGAGGGAC.......................................................................... 3

......AGCTGGCCAGGCTGTGAGGG............................................................................ 7

......AGCTGGCCAGGCTGTGAGGGACG......................................................................... 2

.......GCTGGCCAGGCTGTGAGGGAC.......................................................................... 1

.....................................................TGTGGCCCCCTCCAGCAGCTTCCT......................... 1

...........................................................................CTCCTCACAGTCTGCACAGCTCTC... 1

.............................................................................CCTCACAGTCTGCACAGCTCTC... 5

..............................................................................CTCACAGTCTGCACAGCTC..... 2

..............................................................................CTCACAGTCTGCACAGCTCTCC.. 18

..............................................................................CTCACAGTCTGCACAGCTCT.... 7

..............................................................................CTCACAGTCTGCACAGCTCTC... 406

...............................................................................TCACAGTCTGCACAGCTCTCC.. 1

...............................................................................TCACAGTCTGCACAGCTCTC... 50

>ppt-MIR534a_MI0003501_Physcomitrella_patens_miR534a_stem-loop GSM313216 4

AUAUGCAUGCAACUUGUGUGGACAGACUGACUAGUCUAGUGGUGUAGUGGAAAGUAUUAGUUGGUUGAUGCACGAUGUUGUUUUGCAACAAGUGACUCGUCGCUGCAAUACUGAACCCACAAGUUUUGGAAGUUGUUUUGAUUGGGGCCACCCACAUUGCUCGACUAGAUUCAGUAUGUCCAUUGCAGUUGCAUACAUAU

(((((.((((((((...(((((((.(((((((((((.((..((((.((((..((((((.((.(((.(((((((..(((((.....))))).)))...)))))))))))))))...((((((((.............))))..)))).))))..))))..)).))))))..))))).)))))))...)))))))).))))) (-75.32)

.....CATGCAACTTGTGTGGACAGAC............................................................................................................................................................................. 2

.....CATGCAACTTGTGTGGACAGACT............................................................................................................................................................................ 1

......ATGCAACTTGTGTGGACAGAC............................................................................................................................................................................. 2986

......ATGCAACTTGTGTGGACAGA.............................................................................................................................................................................. 810

......ATGCAACTTGTGTGGACAG............................................................................................................................................................................... 18

......ATGCAACTTGTGTGGACAGACTGAC......................................................................................................................................................................... 3

......ATGCAACTTGTGTGGACAGACT............................................................................................................................................................................ 1446

......ATGCAACTTGTGTGGACAGACTGA.......................................................................................................................................................................... 17

.......TGCAACTTGTGTGGACAGACTGA.......................................................................................................................................................................... 116

.......TGCAACTTGTGTGGACAGACT............................................................................................................................................................................ 25

.......TGCAACTTGTGTGGACAGACTG........................................................................................................................................................................... 2

.......TGCAACTTGTGTGGACAGAC............................................................................................................................................................................. 44

.......TGCAACTTGTGTGGACAGA.............................................................................................................................................................................. 6

.......TGCAACTTGTGTGGACAGACTGAC......................................................................................................................................................................... 16

........GCAACTTGTGTGGACAGACTGA.......................................................................................................................................................................... 3

...................................................AAGTATTAGTTGGTTGATGCACG.............................................................................................................................. 1

........................................................................................................................................................CACATTGCTCGACTAGATTCAGT......................... 1

............................................................................................................................................................................AGTATGTCCATTGCAGTTGCATAC.... 10

............................................................................................................................................................................AGTATGTCCATTGCAGTTGC........ 1

............................................................................................................................................................................AGTATGTCCATTGCAGTTGCATA..... 6

..............................................................................................................................................................................TATGTCCATTGCAGTTGCATA..... 45

..............................................................................................................................................................................TATGTCCATTGCAGTTGCA....... 7

..............................................................................................................................................................................TATGTCCATTGCAGTTGCAT...... 5

..............................................................................................................................................................................TATGTCCATTGCAGTTGCATACA... 2

..............................................................................................................................................................................TATGTCCATTGCAGTTGCATAC.... 1056

...............................................................................................................................................................................ATGTCCATTGCAGTTGCATAC.... 180

...............................................................................................................................................................................ATGTCCATTGCAGTTGCATA..... 3

...............................................................................................................................................................................ATGTCCATTGCAGTTGCATACA... 1

................................................................................................................................................................................TGTCCATTGCAGTTGCATAC.... 1

>ppt-MIR538c_MI0003512_Physcomitrella_patens_miR538c_stem-loop GSM313216 4

UUACAAAGAGUUCUUGAGUCUCCAUGCUUCUCUGACGUUGCAUGGAGUCUAUGUCUGGACCUUCUCCAUUUCCUCUUUCAAGAGAAUGUGAGCAUUGAAGGAUUCGGACAUAGCCUUCAUGCAUGCUAGAGUAUCAUGGAGGCCCGUGAACUACUUGUAA

.(((((..(((((.((.(((((((((...(((((.(((.((((((((.((((((((((((((((.((((...((((....))))...))).)....))))).))))))))))).))))))))))).)))))...))))))))).)).)))))..))))). (-73.90)

................AGTCTCCATGCTTCTCTGACG........................................................................................................................... 11

................AGTCTCCATGCTTCTCTGAC............................................................................................................................ 2

............................TCTCTGACGTTGCATGGAGTC............................................................................................................... 1

...................................CGTTGCATGGAGTCTATGTCT........................................................................................................ 1

.....................................TTGCATGGAGTCTATGTCTGG...................................................................................................... 28

.....................................TTGCATGGAGTCTATGTCTG....................................................................................................... 2

.....................................TTGCATGGAGTCTATGTCTGGACC................................................................................................... 3

.....................................TTGCATGGAGTCTATGTCT........................................................................................................ 1

.....................................TTGCATGGAGTCTATGTCTGGAC.................................................................................................... 13

.....................................TTGCATGGAGTCTATGTCTGGA..................................................................................................... 2980

......................................TGCATGGAGTCTATGTCTGGA..................................................................................................... 326

......................................TGCATGGAGTCTATGTCTGGAC.................................................................................................... 12

......................................TGCATGGAGTCTATGTCTGG...................................................................................................... 10

......................................TGCATGGAGTCTATGTCTG....................................................................................................... 1

.......................................GCATGGAGTCTATGTCTGGA..................................................................................................... 7

........................................CATGGAGTCTATGTCTGGA..................................................................................................... 2

.......................................................................................................TCGGACATAGCCTTCATGCATG................................... 3

........................................................................................................CGGACATAGCCTTCATGCATGC.................................. 3

........................................................................................................CGGACATAGCCTTCATGCATG................................... 363

........................................................................................................CGGACATAGCCTTCATGCA..................................... 1

........................................................................................................CGGACATAGCCTTCATGCAT.................................... 8

.........................................................................................................GGACATAGCCTTCATGCATG................................... 4

.............................................................................................................................CTAGAGTATCATGGAGGCC................ 1

.............................................................................................................................CTAGAGTATCATGGAGGCCC............... 2

.............................................................................................................................CTAGAGTATCATGGAGGCCCGT............. 6

.............................................................................................................................CTAGAGTATCATGGAGGCCCG.............. 164

..............................................................................................................................TAGAGTATCATGGAGGCCCG.............. 2

>ppt-MIR1219a_MI0004718_Physcomitrella_patens_miR1219a_stem-loop GSM313216 5

UGAAGUGUGGACGAUGGAGAGUCAGCCUCUUCCUGCCUCUCACUAGCUUCAUCCCUUCCUCCCUAAAUUUUAGUCUGGGAGGGAAGGAGCUAUUGGUGGUCAGGAAUAGCGCACCCUUCAUUUAUCCACACUUCA

.((((((((((.(((((((.((..((((.((((((((.(.((.(((((((.((((((((...(((.....)))...)))))))).))))))).))).)).)))))).)).)))).)))))))..)))))))))). (-67.20)

....GTGTGGACGATGGAGAGTCAG.............................................................................................................. 1

....GTGTGGACGATGGAGAGTCAGCC............................................................................................................ 1

.....TGTGGACGATGGAGAGTCAGC............................................................................................................. 11

.....TGTGGACGATGGAGAGTCA............................................................................................................... 16

.....TGTGGACGATGGAGAGTCAGCCT........................................................................................................... 64

.....TGTGGACGATGGAGAGTCAG.............................................................................................................. 66

.....TGTGGACGATGGAGAGTCAGCC............................................................................................................ 190

......GTGGACGATGGAGAGTCAG.............................................................................................................. 1

......GTGGACGATGGAGAGTCAGCC............................................................................................................ 5

......GTGGACGATGGAGAGTCAGCCT........................................................................................................... 2

.........................CCTCTTCCTGCCTCTCACTAGCTT...................................................................................... 1

............................CTTCCTGCCTCTCACTAGCT....................................................................................... 16

............................CTTCCTGCCTCTCACTAGC........................................................................................ 10

............................CTTCCTGCCTCTCACTAGCTTC..................................................................................... 5

............................CTTCCTGCCTCTCACTAGCTTCA.................................................................................... 1

............................CTTCCTGCCTCTCACTAGCTT...................................................................................... 68

.............................TTCCTGCCTCTCACTAGCTTC..................................................................................... 1

.............................TTCCTGCCTCTCACTAGCTT...................................................................................... 1

................................CTGCCTCTCACTAGCTTCATCC................................................................................. 1

................................................................AAATTTTAGTCTGGGAGGGAA.................................................. 1

...................................................................................AAGGAGCTATTGGTGGTCAGGA.............................. 1

........................................................................................GCTATTGGTGGTCAGGAATAGC......................... 4

........................................................................................GCTATTGGTGGTCAGGAAT............................ 4

........................................................................................GCTATTGGTGGTCAGGAATAG.......................... 95

........................................................................................GCTATTGGTGGTCAGGAATA........................... 8

.............................................................................................................CGCACCCTTCATTTATCCACAC.... 3

.............................................................................................................CGCACCCTTCATTTATCCACACT... 5

..............................................................................................................GCACCCTTCATTTATCCACACT... 1

>ppt-MIR535d_MI0004725_Physcomitrella_patens_miR535d_stem-loop GSM313216 4

GGUGACAACGAGAGAGAGCACGCCGGAAUGCGUUCAUGCAGCGAGUGCCUGGAGGUGUUCGAGCGUGCCCUCUCCCGUCGUCACC

((((((.(((.(((((.((((((..((((((.((((.(((.....))).)))).))))))..)))))).))))).))).)))))) (-48.50)

..TGACAACGAGAGAGAGCACGCC............................................................. 304

..TGACAACGAGAGAGAGCACG............................................................... 20

..TGACAACGAGAGAGAGCACGC.............................................................. 3341

..TGACAACGAGAGAGAGCAC................................................................ 66

...GACAACGAGAGAGAGCACGCC............................................................. 3

...GACAACGAGAGAGAGCACGC.............................................................. 36

....ACAACGAGAGAGAGCACGCC............................................................. 1

....ACAACGAGAGAGAGCACGC.............................................................. 20

.......ACGAGAGAGAGCACGCCGGAA......................................................... 2

.......ACGAGAGAGAGCACGCCGGA.......................................................... 11

.......................CGGAATGCGTTCATGCAGC........................................... 3

........................................GCGAGTGCCTGGAGGTGTTCGAGC..................... 1

...........................................AGTGCCTGGAGGTGTTCGAGC..................... 2

.............................................TGCCTGGAGGTGTTCGAGC..................... 1

....................................................AGGTGTTCGAGCGTGCCCTCT............ 1

...........................................................CGAGCGTGCCCTCTCCCGTCGT.... 1

...........................................................CGAGCGTGCCCTCTCCCGTCG..... 4

................................................................GTGCCCTCTCCCGTCGTCACC 618

................................................................GTGCCCTCTCCCGTCGTCA.. 6

................................................................GTGCCCTCTCCCGTCGTCAC. 39

.................................................................TGCCCTCTCCCGTCGTCACC 18

.................................................................TGCCCTCTCCCGTCGTCAC. 2

>ppt-MIR319e_MI0005665_Physcomitrella_patens_miR319e_stem-loop GSM313216 5

CUAGCCGUGGGAGCUCCUUCCGGUUCAAUAGUGGCUGAUGUGAGGUUGCACUGCUGCCGAUUCAAACUUCCGGCUUCCCUUUCUUAACACGACAGGGAAUCCGAAUGUCUGAUGCGGGAGCUGUGCGGUCUUCAACUUUAGCGCCUCUUGGACUGAAGGGAGCUCCCAAGUCUU

.......((((((((((((.((((((((.((..(((((..(((((((((((.(((.(((..(((.((...(((.((((((.((.......)).)))))).)))...)).)))..))).))).)))))).)))))...)))))..)).)))))))).))))))))))))...... (-86.10)

.........GGAGCTCCTTCCGGTTCAAT................................................................................................................................................. 1

..........GAGCTCCTTCCGGTTCAAT................................................................................................................................................. 13

..........GAGCTCCTTCCGGTTCAATA................................................................................................................................................ 53

..........GAGCTCCTTCCGGTTCAATAGT.............................................................................................................................................. 7

..........GAGCTCCTTCCGGTTCAATAG............................................................................................................................................... 55

...........AGCTCCTTCCGGTTCAATA................................................................................................................................................ 1

...........AGCTCCTTCCGGTTCAATAG............................................................................................................................................... 2

.................................GCTGATGTGAGGTTGCACTG......................................................................................................................... 1

.......................................................................................................AATGTCTGATGCGGGAGCTGT.................................................. 2

............................................................................................................................GCGGTCTTCAACTTTAGCGCCT............................ 4

................................................................................................................................................CTCTTGGACTGAAGGGAGCTC......... 1

.................................................................................................................................................TCTTGGACTGAAGGGAGCTCCC....... 3

.................................................................................................................................................TCTTGGACTGAAGGGAGCTCC........ 15

..................................................................................................................................................CTTGGACTGAAGGGAGCTCCC....... 1811

..................................................................................................................................................CTTGGACTGAAGGGAGCTCCCA...... 42

..................................................................................................................................................CTTGGACTGAAGGGAGCTC......... 7

..................................................................................................................................................CTTGGACTGAAGGGAGCTCC........ 516

...................................................................................................................................................TTGGACTGAAGGGAGCTCC........ 17

...................................................................................................................................................TTGGACTGAAGGGAGCTCCCA...... 3

...................................................................................................................................................TTGGACTGAAGGGAGCTCCC....... 47

....................................................................................................................................................TGGACTGAAGGGAGCTCCC....... 12

....................................................................................................................................................TGGACTGAAGGGAGCTCCCA...... 4

.....................................................................................................................................................GGACTGAAGGGAGCTCCCA...... 1

>ppt-MIR896_MI0005680_Physcomitrella_patens_miR896_stem-loop GSM313216 8

CCCACCCUGAGCGGGGGGGUCUGAUUGUGGUCGGUCGAGGGGCGCUGACCGUGUGGCCCAAUGGAUAAGGCGCUUGCCUACGGAGCAAGAGAUUCUGGGUUCGAUCCCCAGCAUGGUCGUGGGUCGCCGGAAUUGUAGAUUGUGGCGCGUGUCAAUUUGGCCGAGUGGUUAAGGCGGCAGACUCGAAAUCUGCUGGGGUUUCCCCGCGCAGGUUCAAAUCCUGCA

.....((((.((((((((..((.(..(..(....(((((.(.(((((((((..(((((.(((.((((..(((((((((....).)))...((((((((...((((((((.....))....)))))))))))))).........))))).)))).))).)))))..)))))..)))).)...)))))...)..)).))..)))))))).))))............. (-94.10)

....................................GAGGGGCGCTGACCGTGTGGCCCA..................................................................................................................................................................... 1

..............................................GACCGTGTGGCCCAATGGA................................................................................................................................................................ 16

..............................................GACCGTGTGGCCCAATGGATAA............................................................................................................................................................. 2

..............................................GACCGTGTGGCCCAATGGAT............................................................................................................................................................... 7

..............................................GACCGTGTGGCCCAATGGATA.............................................................................................................................................................. 3

..............................................GACCGTGTGGCCCAATGGATAAGGC.......................................................................................................................................................... 7

..............................................GACCGTGTGGCCCAATGGATAAGG........................................................................................................................................................... 4

..............................................GACCGTGTGGCCCAATGGATAAG............................................................................................................................................................ 7

....................................................GTGGCCCAATGGATAAGGC.......................................................................................................................................................... 1

.................................................................................GGAGCAAGAGATTCTGGGTTCGA......................................................................................................................... 1

...................................................................................AGCAAGAGATTCTGGGTTC........................................................................................................................... 3

......................................................................................AAGAGATTCTGGGTTCGATCCCC.................................................................................................................... 1

...................................................................................................TTCGATCCCCAGCATGGTCG.......................................................................................................... 1

........................................................................................................TCCCCAGCATGGTCGTGGGTC.................................................................................................... 1

...........................................................................................................................TCGCCGGAATTGTAGATTGTGGCGCG............................................................................ 1

............................................................................................................................CGCCGGAATTGTAGATTGTGGCGC............................................................................. 1

.............................................................................................................................GCCGGAATTGTAGATTGTGG................................................................................ 1

.............................................................................................................................GCCGGAATTGTAGATTGTGGC............................................................................... 2

.............................................................................................................................GCCGGAATTGTAGATTGTGGCGC............................................................................. 26

.............................................................................................................................GCCGGAATTGTAGATTGTGGCG.............................................................................. 13

.............................................................................................................................GCCGGAATTGTAGATTGTGGCGCGT........................................................................... 4

...............................................................................................................................CGGAATTGTAGATTGTGGCGCG............................................................................ 1

...................................................................................................................................ATTGTAGATTGTGGCGCGT........................................................................... 1

......................................................................................................................................................GTCAATTTGGCCGAGTGGTTAAGGC.................................................. 33

......................................................................................................................................................GTCAATTTGGCCGAGTGGTT....................................................... 5

......................................................................................................................................................GTCAATTTGGCCGAGTGGTTA...................................................... 11

......................................................................................................................................................GTCAATTTGGCCGAGTGGTTAAG.................................................... 36

......................................................................................................................................................GTCAATTTGGCCGAGTGGT........................................................ 3

......................................................................................................................................................GTCAATTTGGCCGAGTGGTTAAGG................................................... 42

......................................................................................................................................................GTCAATTTGGCCGAGTGGTTAA..................................................... 24

.......................................................................................................................................................TCAATTTGGCCGAGTGGTTAAGG................................................... 1

.......................................................................................................................................................TCAATTTGGCCGAGTGGTTAAG.................................................... 1

........................................................................................................................................................CAATTTGGCCGAGTGGTTAAGGC.................................................. 2

.........................................................................................................................................................AATTTGGCCGAGTGGTTAAG.................................................... 1

.........................................................................................................................................................AATTTGGCCGAGTGGTTAAGG................................................... 1

..........................................................................................................................................................ATTTGGCCGAGTGGTTAAGG................................................... 1

..........................................................................................................................................................ATTTGGCCGAGTGGTTAAGGC.................................................. 1

...........................................................................................................................................................TTTGGCCGAGTGGTTAAGGC.................................................. 2

............................................................................................................................................................TTGGCCGAGTGGTTAAGGC.................................................. 1

.............................................................................................................................................................................GCGGCAGACTCGAAATCTGCTGG............................. 1

..............................................................................................................................................................................CGGCAGACTCGAAATCTGCT............................... 1

...................................................................................................................................................................................GACTCGAAATCTGCTGGGGTT......................... 1

......................................................................................................................................................................................TCGAAATCTGCTGGGGTTT........................ 1

......................................................................................................................................................................................TCGAAATCTGCTGGGGTTTC....................... 2

......................................................................................................................................................................................TCGAAATCTGCTGGGGTTTCCC..................... 1

.......................................................................................................................................................................................CGAAATCTGCTGGGGTTTC....................... 4

.......................................................................................................................................................................................CGAAATCTGCTGGGGTTTCC...................... 1

........................................................................................................................................................................................GAAATCTGCTGGGGTTTCCCCGCGC................ 1

........................................................................................................................................................................................GAAATCTGCTGGGGTTTCCC..................... 1

........................................................................................................................................................................................GAAATCTGCTGGGGTTTCC...................... 1

........................................................................................................................................................................................GAAATCTGCTGGGGTTTCCCC.................... 1

...........................................................................................................................................................................................ATCTGCTGGGGTTTCCCCGCGC................ 1

...........................................................................................................................................................................................ATCTGCTGGGGTTTCCCCGCGCAGGT............ 1

.............................................................................................................................................................................................CTGCTGGGGTTTCCCCGCGCA............... 2

.............................................................................................................................................................................................CTGCTGGGGTTTCCCCGCGCAGG............. 3

>ppt-MIR899_MI0005682_Physcomitrella_patens_miR899_stem-loop GSM313216 4

ACGGCGUUUUUCUCGGAGAAAAAUACCAUCCUUUGGUUCAACAACGGUUGCUGCGUUUUCCACGUUCGGAUACAACCCUGCCCAGUUGCUGUGAGAGAUUGACGAGUGCAAGAAGACCCUCUUUCCAUGUUCCAGACGCAGGCCUUCGCCGAACUGAGAUACAUCGCAAUCGACACACCGGCCGCUUCAGAAUCAGUGACUAGCAUACCCAGCUGCUAUUGG

..((..((((((.....))))))..)).....(((((((.....((((((.((.(((......((((((.......(((((...(..((((.((((((.....................)))))))).))..)....)))))......)))))).((......))......))).)).)))))).....)))))))....(((((........))))).... (-51.42)

..........................................................TCCACGTTCGGATACAACCCT............................................................................................................................................... 1

...........................................................CCACGTTCGGATACAACCCT............................................................................................................................................... 1

.........................................................................................TGTGAGAGATTGACGAGTGCA................................................................................................................ 26

.........................................................................................TGTGAGAGATTGACGAGTGC................................................................................................................. 2

..........................................................................................GTGAGAGATTGACGAGTGCA................................................................................................................ 2

...........................................................................................TGAGAGATTGACGAGTGCAAG.............................................................................................................. 1

.............................................................................................AGAGATTGACGAGTGCAAGAA............................................................................................................ 1

..............................................................................................GAGATTGACGAGTGCAAGAAG........................................................................................................... 6

...............................................................................................AGATTGACGAGTGCAAGAAG........................................................................................................... 15

...............................................................................................AGATTGACGAGTGCAAGAAGA.......................................................................................................... 386

...............................................................................................AGATTGACGAGTGCAAGAAGAC......................................................................................................... 5

...............................................................................................AGATTGACGAGTGCAAGAA............................................................................................................ 8

................................................................................................GATTGACGAGTGCAAGAAGA.......................................................................................................... 1

.........................................................................................................................TTTCCATGTTCCAGACGCAGG................................................................................ 1

..........................................................................................................................TTCCATGTTCCAGACGCAGGC............................................................................... 1

....................................................................................................................................................................................GCCGCTTCAGAATCAGTGACTA.................... 1

......................................................................................................................................................................................CGCTTCAGAATCAGTGACTAG................... 1

>ppt-MIR898b_MI0005694_Physcomitrella_patens_miR898b_stem-loop GSM313216 5

UACUGCAGAACUUAGUUACAGAUUCAUCUGCUUUGUUCAUUUCCGGCAUCUUGCUGUGCACUACUUAGUACGCGCGUAGUUGCAGGCAUUCCGGGUGCAGCAACUGACGCGGAAGACUUUUCCCAGAGUGUGCGCUUCAACUGGCGUGCUGAGUAGUGCACAGCAAUAUGCUGGGCAUGGACAGCUACCACCGCUGAGUUCGCUGUAUAGAACGUUUGCU

(((.((.(((((((((..((((....))))..((((((((.((((((((.(((((((((((((((((((((((...((((((.((((.(((((.((.(((...))))).))))).((((......))))....))))))))))))))))))))))))))))))))).)))))))).))))))))........))))))))))).)))............. (-104.00)

............................TGCTTTGTTCATTTCCGGCATC.......................................................................................................................................................................... 1

...........................................CGGCATCTTGCTGTGCACTAC............................................................................................................................................................ 2

..............................................CATCTTGCTGTGCACTACTTA......................................................................................................................................................... 1

...............................................ATCTTGCTGTGCACTACTTA......................................................................................................................................................... 5

...............................................ATCTTGCTGTGCACTACTTAG........................................................................................................................................................ 47

...............................................ATCTTGCTGTGCACTACTTAGT....................................................................................................................................................... 3

................................................TCTTGCTGTGCACTACTTAGT....................................................................................................................................................... 46

................................................TCTTGCTGTGCACTACTTA......................................................................................................................................................... 1

................................................TCTTGCTGTGCACTACTTAG........................................................................................................................................................ 29

................................................TCTTGCTGTGCACTACTTAG........................................................................................................................................................ 2

................................................TCTTGCTGTGCACTACTTAGTA...................................................................................................................................................... 1

................................................TCTTGCTGTGCACTACTTAGTAC..................................................................................................................................................... 1

.................................................CTTGCTGTGCACTACTTAGTA...................................................................................................................................................... 3

.................................................CTTGCTGTGCACTACTTAGTACG.................................................................................................................................................... 1

.................................................CTTGCTGTGCACTACTTAGT....................................................................................................................................................... 3

.................................................CTTGCTGTGCACTACTTAGTAC..................................................................................................................................................... 2

..................................................TTGCTGTGCACTACTTAGTA...................................................................................................................................................... 175

..................................................TTGCTGTGCACTACTTAGTAC..................................................................................................................................................... 2031

..................................................TTGCTGTGCACTACTTAGTACGC................................................................................................................................................... 4

..................................................TTGCTGTGCACTACTTAGTACG.................................................................................................................................................... 390

..................................................TTGCTGTGCACTACTTAGT....................................................................................................................................................... 12

...................................................TGCTGTGCACTACTTAGTA...................................................................................................................................................... 6

...................................................TGCTGTGCACTACTTAGTACG.................................................................................................................................................... 1

...................................................TGCTGTGCACTACTTAGTACGCG.................................................................................................................................................. 8

...................................................TGCTGTGCACTACTTAGTACG.................................................................................................................................................... 662

...................................................TGCTGTGCACTACTTAGTACGC................................................................................................................................................... 11

...................................................TGCTGTGCACTACTTAGTAC..................................................................................................................................................... 181

....................................................GCTGTGCACTACTTAGTAC..................................................................................................................................................... 10

....................................................GCTGTGCACTACTTAGTACG.................................................................................................................................................... 11

...........................................................ACTACTTAGTACGCGCGTAGTTGC......................................................................................................................................... 2

....................................................................TACGCGCGTAGTTGCAGGCATT.................................................................................................................................. 1

.....................................................................ACGCGCGTAGTTGCAGGCATT.................................................................................................................................. 1

................................................................................................................................TGTGCGCTTCAACTGGCGTGC....................................................................... 1

................................................................................................................................TGTGCGCTTCAACTGGCGTGCT...................................................................... 1

................................................................................................................................TGTGCGCTTCAACTGGCGTGCTGA.................................................................... 1

.................................................................................................................................................GTGCTGAGTAGTGCACAGCAATA.................................................... 2

..................................................................................................................................................TGCTGAGTAGTGCACAGCAAT..................................................... 3

..................................................................................................................................................TGCTGAGTAGTGCACAGCA....................................................... 2

..................................................................................................................................................TGCTGAGTAGTGCACAGCAATA.................................................... 13

...................................................................................................................................................GCTGAGTAGTGCACAGCAAT..................................................... 2

...................................................................................................................................................GCTGAGTAGTGCACAGCAATA.................................................... 3

.....................................................................................................................................................TGAGTAGTGCACAGCAATA.................................................... 1

.....................................................................................................................................................TGAGTAGTGCACAGCAATAT................................................... 1

......................................................................................................................................................GAGTAGTGCACAGCAATATG.................................................. 1

......................................................................................................................................................GAGTAGTGCACAGCAATATGC................................................. 31

.......................................................................................................................................................AGTAGTGCACAGCAATATGC................................................. 1

..........................................................................................................................................................AGTGCACAGCAATATGCTGGG............................................. 2

>ppt-MIR536d_MI0005932_Physcomitrella_patens_miR536d_stem-loop GSM313216 3

AGGGUGCAUUCCAAGUGCCGUCCCUUGGAAGCCGCAGUUUGGCACGAAGCCCUUUCUCCUGCCGCUACUGUUUUGUUAGUCAGGUUUGCAGCGUGUCUCGAUAUAGUGGUGUGCUUCCGUUGCUAUCCUUUCCCUCCAGGUAGGAAUUCUGUGGCAAACUAGUGGUUGGUUCGCUUAUCAAGUUGCAGUCCUUCAUACCUUGAUUUUGAAUUACUGCAGUCACACCCUUCUUGUCGGCAGUUUUUUUUUUUUUUGUGUGUCCUACCGACUGCACUGACUGCAGAAAGUCGAAGCAAUGGAGGUGUCGUUGCAUUUUCCUCAUAGUUUUGCUAGCUUCUACUUCUGGGUUGAUGUUGAACCCACUCCACCCACUUGUAGCGGAAAGAUGUUAGAAUUUCGUGCCAAGCUGUGUGCAACCAAGGGGUUUGUUACUUCAAAUCCAC

.((((((((.....))))..((((((((..(((((((((((((((((((..((..(((..(((((((((((.(((..((.((.((.....)).)).))))).))))))))).)).((((((((..((((..((.....)).)))).....((((.......((((..((((((..(((((.((((((((..((((...........))))..))))))).)..((((.........((((((............(((((((.....))).)))).))))))...((((.(((((...((((((((....))))))))....(((.....)))))))).))))..))))))))).))))))....))))))))..))))))))..)).)..))..))))))))))))))))).))..)))))))).............)))).. (-151.63)

...........................GAAGCCGCAGTTTGGCACGAA........................................................................................................................................................................................................................................................................................................................................................................................................... 1

............................AAGCCGCAGTTTGGCACGA............................................................................................................................................................................................................................................................................................................................................................................................................ 6

............................AAGCCGCAGTTTGGCACGAAG.......................................................................................................................................................................................................................................................................................................................................................................................................... 887

............................AAGCCGCAGTTTGGCACGAA........................................................................................................................................................................................................................................................................................................................................................................................................... 129

............................AAGCCGCAGTTTGGCACGAAGC......................................................................................................................................................................................................................................................................................................................................................................................................... 58

.............................AGCCGCAGTTTGGCACGAAG.......................................................................................................................................................................................................................................................................................................................................................................................................... 226

.............................AGCCGCAGTTTGGCACGAAGC......................................................................................................................................................................................................................................................................................................................................................................................................... 5225

.............................AGCCGCAGTTTGGCACGAAGCC........................................................................................................................................................................................................................................................................................................................................................................................................ 21

.............................AGCCGCAGTTTGGCACGAA........................................................................................................................................................................................................................................................................................................................................................................................................... 34

..............................GCCGCAGTTTGGCACGAAGC......................................................................................................................................................................................................................................................................................................................................................................................................... 45

..............................GCCGCAGTTTGGCACGAAG.......................................................................................................................................................................................................................................................................................................................................................................................................... 1

...............................CCGCAGTTTGGCACGAAGC......................................................................................................................................................................................................................................................................................................................................................................................................... 2

.......................................................................................................................................................................................................................................................................................................................................................................................TAGCGGAAAGATGTTAGAATT............................................... 2

........................................................................................................................................................................................................................................................................................................................................................................................................AATTTCGTGCCAAGCTGTGTGCAAC.......................... 1

.........................................................................................................................................................................................................................................................................................................................................................................................................ATTTCGTGCCAAGCTGTGTGCAAC.......................... 2

..........................................................................................................................................................................................................................................................................................................................................................................................................TTTCGTGCCAAGCTGTGTGCAAC.......................... 4

...........................................................................................................................................................................................................................................................................................................................................................................................................TTCGTGCCAAGCTGTGTGCAA........................... 1134

...........................................................................................................................................................................................................................................................................................................................................................................................................TTCGTGCCAAGCTGTGTGC............................. 50

...........................................................................................................................................................................................................................................................................................................................................................................................................TTCGTGCCAAGCTGTGTGCA............................ 72

...........................................................................................................................................................................................................................................................................................................................................................................................................TTCGTGCCAAGCTGTGTGCAACCA........................ 6

...........................................................................................................................................................................................................................................................................................................................................................................................................TTCGTGCCAAGCTGTGTGCAAC.......................... 18884

...........................................................................................................................................................................................................................................................................................................................................................................................................TTCGTGCCAAGCTGTGTGCAACC......................... 197

............................................................................................................................................................................................................................................................................................................................................................................................................TCGTGCCAAGCTGTGTGCAACC......................... 2944

............................................................................................................................................................................................................................................................................................................................................................................................................TCGTGCCAAGCTGTGTGCA............................ 22

............................................................................................................................................................................................................................................................................................................................................................................................................TCGTGCCAAGCTGTGTGCAAC.......................... 9497

............................................................................................................................................................................................................................................................................................................................................................................................................TCGTGCCAAGCTGTGTGCAACCA........................ 48

............................................................................................................................................................................................................................................................................................................................................................................................................TCGTGCCAAGCTGTGTGCAA........................... 797

.............................................................................................................................................................................................................................................................................................................................................................................................................CGTGCCAAGCTGTGTGCAAC.......................... 193

.............................................................................................................................................................................................................................................................................................................................................................................................................CGTGCCAAGCTGTGTGCAA........................... 9

.............................................................................................................................................................................................................................................................................................................................................................................................................CGTGCCAAGCTGTGTGCAACC......................... 72

.............................................................................................................................................................................................................................................................................................................................................................................................................CGTGCCAAGCTGTGTGCAACCA........................ 2

..............................................................................................................................................................................................................................................................................................................................................................................................................GTGCCAAGCTGTGTGCAACCA........................ 5

..............................................................................................................................................................................................................................................................................................................................................................................................................GTGCCAAGCTGTGTGCAAC.......................... 468

..............................................................................................................................................................................................................................................................................................................................................................................................................GTGCCAAGCTGTGTGCAACC......................... 181

...............................................................................................................................................................................................................................................................................................................................................................................................................TGCCAAGCTGTGTGCAACC......................... 2

...............................................................................................................................................................................................................................................................................................................................................................................................................TGCCAAGCTGTGTGCAACCAA....................... 1

...............................................................................................................................................................................................................................................................................................................................................................................................................TGCCAAGCTGTGTGCAACCAAG...................... 2

...................................................................................................................................................................................................................................................................................................................................................................................................................AAGCTGTGTGCAACCAAGGGGT.................. 2

...................................................................................................................................................................................................................................................................................................................................................................................................................AAGCTGTGTGCAACCAAGGGGTT................. 1

...................................................................................................................................................................................................................................................................................................................................................................................................................AAGCTGTGTGCAACCAAGGGG................... 1

>ppt-MIR1030a_MI0005975_Physcomitrella_patens_miR1030a_stem-loop GSM313216 3

AAGGUACGCAAUUAAAGCUGCACCGUCACAUUUCUGCAUCUGCACCUGCACCAAAACGCGACUUUGAGGUUGUGAUGGAAUUCUGCAGUCACACCCAGCCAUUUGCAAGAGUGUUCUGCCGGUGUUGGAUUGUGGCAAUGGAUUGCAUUCACAGGCUUGUGUACUGGUGCAGUGCUUAUGCAGAAAUGUUGCGUUGUAGCUCAAACUGGAUUGAG

........(((((..(((((((.(((.((((((((((((..((((.(((((((..((((......(((.((((((((.(((((....((((((.(((((.((..(((.(......))))..)))))))..))))))...))))).)).)))))).)))))))..)))))))))))..)))))))))))).))).))))))).......))))).. (-88.90)

................................TCTGCATCTGCACCTGCACCA.................................................................................................................................................................. 89

................................TCTGCATCTGCACCTGCACCAAA................................................................................................................................................................ 2

................................TCTGCATCTGCACCTGCAC.................................................................................................................................................................... 1

................................TCTGCATCTGCACCTGCACCAA................................................................................................................................................................. 1

..................................................................................................................................................................ACTGGTGCAGTGCTTATGCAGAAA............................. 2

...................................................................................................................................................................CTGGTGCAGTGCTTATGCAGAA.............................. 1

....................................................................................................................................................................TGGTGCAGTGCTTATGCAGAA.............................. 1

....................................................................................................................................................................TGGTGCAGTGCTTATGCAGA............................... 1

......................................................................................................................................................................GTGCAGTGCTTATGCAGAAA............................. 213

......................................................................................................................................................................GTGCAGTGCTTATGCAGAA.............................. 22

.......................................................................................................................................................................TGCAGTGCTTATGCAGAAA............................. 1

.....................................................................................................................................................................................AGAAATGTTGCGTTGTAGCTC............. 1

>ppt-MIR1030f_MI0005980_Physcomitrella_patens_miR1030f_stem-loop GSM313216 3

CCUCUACAUAACUACUCCAAUGCCACGUAUCUGCAUCUGCACCUGCACCAAAGUUUUGUAAUUCUACGGCUCGUGGUAGUCUGUUGUUGUGAAGGUGGGAUGUAGCUGAGUGUAUUACUCGGUGUUCAUUUGAUCAUUAUUACCCGAUGGUGCGGGUUCUUAUGCAGAGCCGUGGCAUUGGAGUAAUCCUCUACUU

............(((((((((((((((..(((((((..(.((((((((((.....(((((....))))).(((.((((..........((((.(((.(((((..((((((((...))))))))...))))).))).))))))))))))))))))))).)..)))))))..)))))))))))))))........... (-82.00)

......................CCACGTATCTGCATCTGCAC.......................................................................................................................................................... 1

.............................TCTGCATCTGCACCTGCACCA.................................................................................................................................................. 89

.............................TCTGCATCTGCACCTGCACCAAA................................................................................................................................................ 2

.............................TCTGCATCTGCACCTGCAC.................................................................................................................................................... 1

.............................TCTGCATCTGCACCTGCACCAA................................................................................................................................................. 1

.....................................................................................................................................................GTGCGGGTTCTTATGCAGAGC.......................... 137

......................................................................................................................................................TGCGGGTTCTTATGCAGAGC.......................... 8

........................................................................................................................................................CGGGTTCTTATGCAGAGCCG........................ 1

.....................................................................................................................................................................AGAGCCGTGGCATTGGAGTAA.......... 1

>ppt-MIR1047_MI0006006_Physcomitrella_patens_miR1047_stem-loop GSM313216 3

UUGCGUUAUGUGUGACCUAUGACCUCUUGGAUCACUAGGUAGUCCUUGAUUACAAAGGCCUUAAUUCUUUACAGCAAACGAGUUAAGGCCCCUGUGAUCAGCGAUGACCUAGUUGCUCAAUUGGGCACAGUGUCUCUUCGCGAGG

(((((......(.(((((.((.((..(((..(.(((((((.(((.(((((((((..(((((((((((.((......)).)))))))))))..))))))))).))).))))))).)..)))..)).)).)).))).)..))))).. (-56.70)

.................................ACTAGGTAGTCCTTGATTACA........................................................................................... 5

..................................CTAGGTAGTCCTTGATTACA........................................................................................... 1

......................................................AAGGCCTTAATTCTTTACAG....................................................................... 1

......................................................AAGGCCTTAATTCTTTACA........................................................................ 1

..............................................................................................TGATCAGCGATGACCTAGTTG.............................. 3

>ppt-MIR1063b_MI0006023_Physcomitrella_patens_miR1063b_stem-loop GSM313216 3

AGAGUUACUUGUGCUUCGUAGUUCACUUACAUCUUGGAGUACUGCAUCUUCAGAUCUGUGAGUCUUCGCCGCCUAUUGCCUUCGCCACCAUGCUUGACGAAUGGUUUGAAACUGAUCACAUCCAACUUGAACGGAUAUCCUGCCCCUUCAUAGUGCUGUGAUUCCGUUCAAGCAGUUCAGUUGUCCGACAACCAUGGUGUGGUAGGACAGUAGUACUGCUAAGGCUUUGACAUUUUGAAGAUGCAGCACUACAAGAAGUGUGUGAGCAACGGGCUGGAUUCAAGAU

.(((((......(((.(((.((((((.(((.(((((.(((.(((((((((((((..((((((((((.((...(((((((((((((.((((((......((((.(((((((...(((((((....(((((((.((.........)).)))).)))..)))))))...))))))).)))).((((.....)))))))))))))).))).))))))....)).)))))))..))).))))))))))))).))).))))).))).)))))).))))))..)))))..... (-107.90)

...........................TACATCTTGGAGTACTGCATC.............................................................................................................................................................................................................................................. 1

.............................CATCTTGGAGTACTGCATCT............................................................................................................................................................................................................................................. 4

.............................CATCTTGGAGTACTGCATCTT............................................................................................................................................................................................................................................ 33

.............................CATCTTGGAGTACTGCATCTTC........................................................................................................................................................................................................................................... 9

.............................CATCTTGGAGTACTGCATC.............................................................................................................................................................................................................................................. 9

..............................ATCTTGGAGTACTGCATCTT............................................................................................................................................................................................................................................ 1

...............................TCTTGGAGTACTGCATCTTCA.......................................................................................................................................................................................................................................... 3

...............................TCTTGGAGTACTGCATCTTCA.......................................................................................................................................................................................................................................... 1

................................CTTGGAGTACTGCATCTTC........................................................................................................................................................................................................................................... 1

....................................................................................................................................................................................................GTGTGGTAGGACAGTAGTACTGC................................................................... 1

..............................................................................................................................................................................................................................................AGATGCAGCACTACAAGAAGT........................... 4

...............................................................................................................................................................................................................................................GATGCAGCACTACAAGAAGT........................... 8

>ppt-MIR1068_MI0006034_Physcomitrella_patens_miR1068_stem-loop GSM313216 3

CUUUUCAACACCUUGGCGCUGGCCUUCGUAGCCAUUUGCUUGAAGGUCAACUCAGACGGGUGGAAGAGGCAAUGUAUUGACGUCUUCCUCCAUUUUCCGACCAUUCUCUAGGCACCACAGAGGCAUCAAUGCUCUGCCUGUUUCUGCCGUGUGGGUUGGUCCUCAAGCAACGGGCUACGAGGACCAAUGUCAAUAAGCAUCAAC

............((((((.(((.((((((((((..((((((((.(..(((((((.((((..((((.(((((..(((((((.(((((..........((............)).......))))).)))))))..))))).))))..)))).)))))))..).))))))))..)))))))))).))).))))))........... (-88.73)

............................TAGCCATTTGCTTGAAGGTCA........................................................................................................................................................... 3

.............................AGCCATTTGCTTGAAGGTCAA.......................................................................................................................................................... 2

..............................GCCATTTGCTTGAAGGTCAAC......................................................................................................................................................... 1

.................................................ACTCAGACGGGTGGAAGAGGCA..................................................................................................................................... 1

............................................................................................................................................................TGGTCCTCAAGCAACGGGCTA........................... 1

.............................................................................................................................................................GGTCCTCAAGCAACGGGCTAC.......................... 11

...............................................................................................................................................................TCCTCAAGCAACGGGCTACG......................... 1

>ppt-MIR1069_MI0006035_Physcomitrella_patens_miR1069_stem-loop GSM313216 4

UGUUCUUCAAGCACAUGUCCACUUCAUGUUCUCAAGUCUUAUCAUUGGAUUGAGCACCUUGGUCUUUCAAAUAUUCUAUUAUGAUAUGUUGAUAUAUAAUUGGUCUUUCAAGUAUUUUCUCAUUAAUAUGAAAACAUAUUAUUACAAAAUAUUUUUGCAUAUUAUUGUAGUAUACUUGAUAAAUCAAAGUGCUCACUGUAAUGAUAAAGCUUGAGAACAUGAAGUCUACUUGACUUCUACUUUG

.............((.((..((((((((((((((((..(((((((((.(.(((((((.(((((...((((.(((.((((.(((((((((.(((((....((((....))))............(((((((....))))))).......)))))...))))))))).)))).))).))))...))))).))))))).).)))))))))..))))))))))))))))..)).))............ (-79.20)

............................TTCTCAAGTCTTATCATTGGA................................................................................................................................................................................................... 5

............................TTCTCAAGTCTTATCATTGGAT.................................................................................................................................................................................................. 1

.............................TCTCAAGTCTTATCATTGGAT.................................................................................................................................................................................................. 10

.............................TCTCAAGTCTTATCATTGGA................................................................................................................................................................................................... 1

.....................................CTTATCATTGGATTGAGCACC.......................................................................................................................................................................................... 1

.................................................TTGAGCACCTTGGTCTTTCAA.............................................................................................................................................................................. 1

................................................................................................................................................................................TGATAAATCAAAGTGCTCACT............................................... 2

.............................................................................................................................................................................................TGCTCACTGTAATGATAAAGC.................................. 1

....................................................................................................................................................................................................TGTAATGATAAAGCTTGAGAA........................... 1

.....................................................................................................................................................................................................GTAATGATAAAGCTTGAGAAC.......................... 4

......................................................................................................................................................................................................TAATGATAAAGCTTGAGAACA......................... 1

>ppt-MIR2083_MI0010537_Physcomitrella_patens_miR2083_stem-loop GSM313216 5

AAAAGUGUGUGUAUGCGGAAGUUGGAGAAAACCCAGACUUCAUUGCUCGAUGAAGCAUGUAGCUUCGCAGUCGUACUUCUUGCACUCCUCCAUCUCUCGCAUCUCUUGCGCAUUGUGGUUCUCCAACACCUGCACAGCGGCGGUCUUGUCCGGAUUAGGGAUCGUCUACACCAGAUAAGCCACGGCGUUUUUCUCGGAGAAAAAUACCAUCCUUUGGUUCAACAACGGUUGCUGCGUUUUCCACGUUCGGAUACAACCCUGCCCAGUUGCUGUGAGAGAUUGACGAGUGCAAGAAGACCCUCUUUCCAUGUUCCAGACGCA

....((((.((((((.(((((..((.(((....((..(((((((....)))))))..))....))).........((((((((((((.((.((((((((((.(..(((.(((.(((((....)).)))..)))...((((.(((..(((((((((...(((.((.......(((...((((..((..((((((.....))))))..)).....(((......))).)))).)))))...)))..)))))))))..))))))).)))..).)))))))))).)).)))))))))))).))..)))))))))...)).)))). (-109.51)

...........................................................TAGCTTCGCAGTCGTACTTCT................................................................................................................................................................................................................................................. 1

............................................................................TTCTTGCACTCCTCCATCT.................................................................................................................................................................................................................................. 1

............................................................................TTCTTGCACTCCTCCATCTCT................................................................................................................................................................................................................................ 114

............................................................................TTCTTGCACTCCTCCATCTC................................................................................................................................................................................................................................. 22

............................................................................TTCTTGCACTCCTCCATCTCTC............................................................................................................................................................................................................................... 12

............................................................................TTCTTGCACTCCTCCATCTCTCGC............................................................................................................................................................................................................................. 1

.............................................................................TCTTGCACTCCTCCATCTCT................................................................................................................................................................................................................................ 4

.............................................................................TCTTGCACTCCTCCATCTCTC............................................................................................................................................................................................................................... 1

..............................................................................CTTGCACTCCTCCATCTCT................................................................................................................................................................................................................................ 1

.......................................................................................................................................................................ACACCAGATAAGCCACGGCGT..................................................................................................................................... 1

..........................................................................................................................................................................CCAGATAAGCCACGGCGTTTT.................................................................................................................................. 1

............................................................................................................................................................................AGATAAGCCACGGCGTTTTTC................................................................................................................................ 1

...............................................................................................................................................................................................................................................TCCACGTTCGGATACAACCCT............................................................. 1

................................................................................................................................................................................................................................................CCACGTTCGGATACAACCCT............................................................. 1

..............................................................................................................................................................................................................................................................................TGTGAGAGATTGACGAGTGCA.............................. 26

..............................................................................................................................................................................................................................................................................TGTGAGAGATTGACGAGTGC............................... 2

...............................................................................................................................................................................................................................................................................GTGAGAGATTGACGAGTGCA.............................. 2

................................................................................................................................................................................................................................................................................TGAGAGATTGACGAGTGCAAG............................ 1

..................................................................................................................................................................................................................................................................................AGAGATTGACGAGTGCAAGAA.......................... 1

...................................................................................................................................................................................................................................................................................GAGATTGACGAGTGCAAGAAG......................... 6

....................................................................................................................................................................................................................................................................................AGATTGACGAGTGCAAGAAG......................... 15

....................................................................................................................................................................................................................................................................................AGATTGACGAGTGCAAGAAGA........................ 386

....................................................................................................................................................................................................................................................................................AGATTGACGAGTGCAAGAAGAC....................... 5

....................................................................................................................................................................................................................................................................................AGATTGACGAGTGCAAGAA.......................... 8

.....................................................................................................................................................................................................................................................................................GATTGACGAGTGCAAGAAGA........................ 1

>ppt-MIR319a_MI0003496_Physcomitrella_patens_miR319a_stem-loop GSM313217 5

GUGGAGCUCCGUUUCGGUCCAAUAGUGGCUGCGACGGAAGGUGGUCCCGCUGCCGAAUCACACGUCCGGGUUGCUUAUCGGGGCAGGGCCCCGAUACGGUAUCCGAACGUUUGUCCCGGGAACUGGUCGACCUUCCGCCCGGCGUCUCUUGGACUGAAGGGAGCUCCAC

((((((((((.(((((((((((.((..((((.(.((((((((.(.((.(.(.(((...((.((((.(((((.((((((((((((...))))))))).)))))))).)))).))...))).).).)).).)))))))).)))))..)).))))))))))))))))))))) (-98.80)

.TGGAGCTCCGTTTCGGTCCAAT.................................................................................................................................................. 2

..GGAGCTCCGTTTCGGTCCAAT.................................................................................................................................................. 1

..GGAGCTCCGTTTCGGTCCAATA................................................................................................................................................. 3

...GAGCTCCGTTTCGGTCCAATAG................................................................................................................................................ 226

...GAGCTCCGTTTCGGTCCAATAGT............................................................................................................................................... 24

...GAGCTCCGTTTCGGTCCAAT.................................................................................................................................................. 17

...GAGCTCCGTTTCGGTCCAA................................................................................................................................................... 4

...GAGCTCCGTTTCGGTCCAATA................................................................................................................................................. 92

....AGCTCCGTTTCGGTCCAATAG................................................................................................................................................ 431

....AGCTCCGTTTCGGTCCAAT.................................................................................................................................................. 54

....AGCTCCGTTTCGGTCCAATA................................................................................................................................................. 196

....AGCTCCGTTTCGGTCCAATAGT............................................................................................................................................... 62

.....GCTCCGTTTCGGTCCAATAG................................................................................................................................................ 6

.............TCGGTCCAATAGTGGCTGCG........................................................................................................................................ 1

.............TCGGTCCAATAGTGGCTGCGA....................................................................................................................................... 1

.........................TGGCTGCGACGGAAGGTGG............................................................................................................................. 2

.........................TGGCTGCGACGGAAGGTGGTCCCG........................................................................................................................ 1

.........................TGGCTGCGACGGAAGGTGGTCC.......................................................................................................................... 1274

.........................TGGCTGCGACGGAAGGTGGTC........................................................................................................................... 425

.........................TGGCTGCGACGGAAGGTGGT............................................................................................................................ 12

.........................TGGCTGCGACGGAAGGTGGTCCC......................................................................................................................... 56

..........................GGCTGCGACGGAAGGTGGTCC.......................................................................................................................... 6

..........................GGCTGCGACGGAAGGTGGTC........................................................................................................................... 2

...........................GCTGCGACGGAAGGTGGTCC.......................................................................................................................... 2

...........................GCTGCGACGGAAGGTGGTC........................................................................................................................... 1

..................................CGGAAGGTGGTCCCGCTGC.................................................................................................................... 1

........................................GTGGTCCCGCTGCCGAATCACA........................................................................................................... 1

........................................GTGGTCCCGCTGCCGAATCACA........................................................................................................... 1

...............................................CGCTGCCGAATCACACGTCCG..................................................................................................... 1

..............................................................................................................................TCGACCTTCCGCCCGGCGT........................ 1

..............................................................................................................................TCGACCTTCCGCCCGGCGTC....................... 10

..............................................................................................................................TCGACCTTCCGCCCGGCGTCT...................... 11

....................................................................................................................................TTCCGCCCGGCGTCTCTTGGA................ 1

.....................................................................................................................................TCCGCCCGGCGTCTCTTGGAC............... 1

................................................................................................................................................TCTCTTGGACTGAAGGGAGCTCC.. 1

................................................................................................................................................TCTCTTGGACTGAAGGGAGC..... 7

................................................................................................................................................TCTCTTGGACTGAAGGGAGCT.... 16

................................................................................................................................................TCTCTTGGACTGAAGGGAG...... 2

.................................................................................................................................................CTCTTGGACTGAAGGGAGCT.... 2

.................................................................................................................................................CTCTTGGACTGAAGGGAGCTC... 1

..................................................................................................................................................TCTTGGACTGAAGGGAGCTCC.. 9

..................................................................................................................................................TCTTGGACTGAAGGGAGCTC... 1

...................................................................................................................................................CTTGGACTGAAGGGAGCTCCAC 5

...................................................................................................................................................CTTGGACTGAAGGGAGCTCCA. 214

...................................................................................................................................................CTTGGACTGAAGGGAGCTC... 14

...................................................................................................................................................CTTGGACTGAAGGGAGCTCC.. 453

....................................................................................................................................................TTGGACTGAAGGGAGCTCC.. 14

....................................................................................................................................................TTGGACTGAAGGGAGCTCCAC 4

....................................................................................................................................................TTGGACTGAAGGGAGCTCCA. 17

.....................................................................................................................................................TGGACTGAAGGGAGCTCCA. 4

.....................................................................................................................................................TGGACTGAAGGGAGCTCCAC 3

>ppt-MIR319b_MI0003497_Physcomitrella_patens_miR319b_stem-loop GSM313217 4

GAGCUCUUUUCAGUCCAGUAGCAGCUAAUGUCGAAGGUUGUACCGCUGCCGACUCAAACUUCCGGCUUCCAUAUCACGACGCGUGAUAUGGAAUCCGAAGGUCUGAUCCGGGAGCUGAUCGAUCUCCAGGUUAGCAUCUCUUGGACUGAAGGGAGCUCCU

((((((((((((((((((.((..((((((.(.((.(((((....(((.(((..(((.((((.(((.((((((((((((...)))))))))))).))).)))).)))..))).)))....))))))).).))))))..)).)))))))))))))))))).. (-83.70)

GAGCTCTTTTCAGTCCAGT............................................................................................................................................. 1

GAGCTCTTTTCAGTCCAGTAG........................................................................................................................................... 3

GAGCTCTTTTCAGTCCAGTA............................................................................................................................................ 8

.AGCTCTTTTCAGTCCAGTA............................................................................................................................................ 7

.AGCTCTTTTCAGTCCAGTAGCA......................................................................................................................................... 1

.AGCTCTTTTCAGTCCAGTAG........................................................................................................................................... 18

...........................................CGCTGCCGACTCAAACTTCCG................................................................................................ 2

..............................................TGCCGACTCAAACTTCCGGCT............................................................................................. 1

.................................................................................................AAGGTCTGATCCGGGAGCTG........................................... 2

.................................................................................................AAGGTCTGATCCGGGAGCTGA.......................................... 20

..................................................................................................AGGTCTGATCCGGGAGCTGA.......................................... 1

..................................................................................................................................TTAGCATCTCTTGGACTGAAGGG....... 1

..................................................................................................................................TTAGCATCTCTTGGACTGA........... 1

..................................................................................................................................TTAGCATCTCTTGGACTGAA.......... 5

..................................................................................................................................TTAGCATCTCTTGGACTGAAG......... 83

...................................................................................................................................TAGCATCTCTTGGACTGAAG......... 3

....................................................................................................................................AGCATCTCTTGGACTGAAGGGA...... 1

......................................................................................................................................CATCTCTTGGACTGAAGGGA...... 1

......................................................................................................................................CATCTCTTGGACTGAAGGGAGCT... 1

.......................................................................................................................................ATCTCTTGGACTGAAGGGAGC.... 4

.......................................................................................................................................ATCTCTTGGACTGAAGGGA...... 7

........................................................................................................................................TCTCTTGGACTGAAGGGAGCTCC. 1

........................................................................................................................................TCTCTTGGACTGAAGGGAGC.... 7

........................................................................................................................................TCTCTTGGACTGAAGGGAGCT... 16

........................................................................................................................................TCTCTTGGACTGAAGGGAG..... 2

.........................................................................................................................................CTCTTGGACTGAAGGGAGCT... 2

.........................................................................................................................................CTCTTGGACTGAAGGGAGCTC.. 1

..........................................................................................................................................TCTTGGACTGAAGGGAGCTCC. 9

..........................................................................................................................................TCTTGGACTGAAGGGAGCTC.. 1

...........................................................................................................................................CTTGGACTGAAGGGAGCTCCT 11

...........................................................................................................................................CTTGGACTGAAGGGAGCTC.. 14

...........................................................................................................................................CTTGGACTGAAGGGAGCTCC. 453

............................................................................................................................................TTGGACTGAAGGGAGCTCC. 14

............................................................................................................................................TTGGACTGAAGGGAGCTCCT 1

>ppt-MIR319c_MI0003498_Physcomitrella_patens_miR319c_stem-loop GSM313217 7

UAACCUCACUGGCUGUGGGAGCUUCCUUCGGUUCAAUAGUGGCUGAUAUGAGGUUGCACUGCUGCCGACUCAAACUUCCGGCUUCCCUCUCUUAGAAUGGCAGGGAAUCCGAAUGUCUGAUGCGGGAGCUGAGCGGUCUUCAACUCAGCUUCUCUUGGACUGAAGGGAGCUCCCAUGUCUUUGUGGUUA

(((((.((..(((.((((((((((((((((((((((.((.((((((..(((((((((.(.(((.(((..(((.((...(((.((((((.((.......)).)))))).)))...)).)))..))).))).).)))).)))))..)))))).)).)))))))))))))))))))))))))..)).))))) (-99.10)

..................GAGCTTCCTTCGGTTCAATAG...................................................................................................................................................... 751

..................GAGCTTCCTTCGGTTCAATA....................................................................................................................................................... 162

..................GAGCTTCCTTCGGTTCAAT........................................................................................................................................................ 62

..................GAGCTTCCTTCGGTTCAATAGT..................................................................................................................................................... 88

...................AGCTTCCTTCGGTTCAATA....................................................................................................................................................... 11

...................AGCTTCCTTCGGTTCAATAG...................................................................................................................................................... 279

...................AGCTTCCTTCGGTTCAATAGT..................................................................................................................................................... 21

....................GCTTCCTTCGGTTCAATAG...................................................................................................................................................... 1

.....................CTTCCTTCGGTTCAATAGT..................................................................................................................................................... 1

.....................CTTCCTTCGGTTCAATAGTGG................................................................................................................................................... 1

.........................CTTCGGTTCAATAGTGGCTGA............................................................................................................................................... 6

..........................TTCGGTTCAATAGTGGCTGA............................................................................................................................................... 4

......................................GTGGCTGATATGAGGTTGCAC.................................................................................................................................. 2

......................................GTGGCTGATATGAGGTTGCA................................................................................................................................... 1

.......................................TGGCTGATATGAGGTTGCA................................................................................................................................... 17

.......................................TGGCTGATATGAGGTTGCACT................................................................................................................................. 3

.......................................TGGCTGATATGAGGTTGCAC.................................................................................................................................. 643

........................................GGCTGATATGAGGTTGCAC.................................................................................................................................. 2

.....................................................TTGCACTGCTGCCGACTCAAAC.................................................................................................................. 1

.....................................................TTGCACTGCTGCCGACTCAAA................................................................................................................... 1

...........................................................TGCTGCCGACTCAAACTTCCG............................................................................................................. 4

..............................................................TGCCGACTCAAACTTCCGGCT.......................................................................................................... 1

................................................................................GCTTCCCTCTCTTAGAATGGCAGGGA................................................................................... 5

.................................................................................CTTCCCTCTCTTAGAATGGCAGGGA................................................................................... 1

..................................................................................TTCCCTCTCTTAGAATGGCAGG..................................................................................... 1

...............................................................................................................AATGTCTGATGCGGGAGCTGA......................................................... 53

...............................................................................................................AATGTCTGATGCGGGAGCTG.......................................................... 1

................................................................................................................ATGTCTGATGCGGGAGCTGA......................................................... 2

.................................................................................................................................TGAGCGGTCTTCAACTCAGCTTCT.................................... 1

..................................................................................................................................GAGCGGTCTTCAACTCAGCTT...................................... 1

....................................................................................................................................GCGGTCTTCAACTCAGCTTCT.................................... 219

....................................................................................................................................GCGGTCTTCAACTCAGCTT...................................... 5

....................................................................................................................................GCGGTCTTCAACTCAGCTTC..................................... 48

....................................................................................................................................GCGGTCTTCAACTCAGCTTCTC................................... 1

......................................................................................................................................GGTCTTCAACTCAGCTTCT.................................... 1

..................................................................................................................................................AGCTTCTCTTGGACTGAAGGG...................... 2

..................................................................................................................................................AGCTTCTCTTGGACTGAAGGGAGC................... 1

..................................................................................................................................................AGCTTCTCTTGGACTGAAGG....................... 1

...................................................................................................................................................GCTTCTCTTGGACTGAAGGGAGCTC................. 1

...................................................................................................................................................GCTTCTCTTGGACTGAAGGGAGC................... 1

.....................................................................................................................................................TTCTCTTGGACTGAAGGGAGC................... 1

......................................................................................................................................................TCTCTTGGACTGAAGGGAGCTCC................ 1

......................................................................................................................................................TCTCTTGGACTGAAGGGAGC................... 7

......................................................................................................................................................TCTCTTGGACTGAAGGGAGCT.................. 16

......................................................................................................................................................TCTCTTGGACTGAAGGGAG.................... 2

.......................................................................................................................................................CTCTTGGACTGAAGGGAGCT.................. 2

.......................................................................................................................................................CTCTTGGACTGAAGGGAGCTCCC............... 1

.......................................................................................................................................................CTCTTGGACTGAAGGGAGCTC................. 1

........................................................................................................................................................TCTTGGACTGAAGGGAGCTCCC............... 2

........................................................................................................................................................TCTTGGACTGAAGGGAGCTCC................ 9

........................................................................................................................................................TCTTGGACTGAAGGGAGCTC................. 1

.........................................................................................................................................................CTTGGACTGAAGGGAGCTCCC............... 1812

.........................................................................................................................................................CTTGGACTGAAGGGAGCTCCCA.............. 40

.........................................................................................................................................................CTTGGACTGAAGGGAGCTC................. 14

.........................................................................................................................................................CTTGGACTGAAGGGAGCTCC................ 453

..........................................................................................................................................................TTGGACTGAAGGGAGCTCC................ 14

..........................................................................................................................................................TTGGACTGAAGGGAGCTCCCA.............. 1

..........................................................................................................................................................TTGGACTGAAGGGAGCTCCC............... 53

...........................................................................................................................................................TGGACTGAAGGGAGCTCCC............... 6

...........................................................................................................................................................TGGACTGAAGGGAGCTCCCA.............. 1

>ppt-MIR538c_MI0003512_Physcomitrella_patens_miR538c_stem-loop GSM313217 4

UUACAAAGAGUUCUUGAGUCUCCAUGCUUCUCUGACGUUGCAUGGAGUCUAUGUCUGGACCUUCUCCAUUUCCUCUUUCAAGAGAAUGUGAGCAUUGAAGGAUUCGGACAUAGCCUUCAUGCAUGCUAGAGUAUCAUGGAGGCCCGUGAACUACUUGUAA

.(((((..(((((.((.(((((((((...(((((.(((.((((((((.((((((((((((((((.((((...((((....))))...))).)....))))).))))))))))).))))))))))).)))))...))))))))).)).)))))..))))). (-73.90)

................AGTCTCCATGCTTCTCTGACG........................................................................................................................... 9

................AGTCTCCATGCTTCTCTGAC............................................................................................................................ 3

................AGTCTCCATGCTTCTCTGA............................................................................................................................. 1

.....................................TTGCATGGAGTCTATGTCTGG...................................................................................................... 41

.....................................TTGCATGGAGTCTATGTCTG....................................................................................................... 1

.....................................TTGCATGGAGTCTATGTCTGGACC................................................................................................... 2

.....................................TTGCATGGAGTCTATGTCTGGAC.................................................................................................... 20

.....................................TTGCATGGAGTCTATGTCTGGA..................................................................................................... 2853

......................................TGCATGGAGTCTATGTCTGGA..................................................................................................... 283

......................................TGCATGGAGTCTATGTCTGGAC.................................................................................................... 14

......................................TGCATGGAGTCTATGTCTGG...................................................................................................... 10

......................................TGCATGGAGTCTATGTCTG....................................................................................................... 1

.......................................GCATGGAGTCTATGTCTGGA..................................................................................................... 5

........................................CATGGAGTCTATGTCTGGA..................................................................................................... 2

......................................................................................................TTCGGACATAGCCTTCATGCATG................................... 1

.......................................................................................................TCGGACATAGCCTTCATGCATG................................... 3

........................................................................................................CGGACATAGCCTTCATGCATGC.................................. 2

........................................................................................................CGGACATAGCCTTCATGCAT.................................... 10

........................................................................................................CGGACATAGCCTTCATGCATG................................... 383

........................................................................................................CGGACATAGCCTTCATGCA..................................... 1

.........................................................................................................GGACATAGCCTTCATGCATG................................... 4

.............................................................................................................................CTAGAGTATCATGGAGGCCC............... 1

.............................................................................................................................CTAGAGTATCATGGAGGCCCGT............. 5

.............................................................................................................................CTAGAGTATCATGGAGGCCCG.............. 162

..............................................................................................................................TAGAGTATCATGGAGGCCCG.............. 3

......................................................................................................................................CATGGAGGCCCGTGAACTAC...... 1

>ppt-MIR1219a_MI0004718_Physcomitrella_patens_miR1219a_stem-loop GSM313217 5

UGAAGUGUGGACGAUGGAGAGUCAGCCUCUUCCUGCCUCUCACUAGCUUCAUCCCUUCCUCCCUAAAUUUUAGUCUGGGAGGGAAGGAGCUAUUGGUGGUCAGGAAUAGCGCACCCUUCAUUUAUCCACACUUCA

.((((((((((.(((((((.((..((((.((((((((.(.((.(((((((.((((((((...(((.....)))...)))))))).))))))).))).)).)))))).)).)))).)))))))..)))))))))). (-67.20)

...AGTGTGGACGATGGAGAGTC................................................................................................................ 1

.....TGTGGACGATGGAGAGTCA............................................................................................................... 19

.....TGTGGACGATGGAGAGTCAGC............................................................................................................. 26

.....TGTGGACGATGGAGAGTCAGCCTC.......................................................................................................... 1

.....TGTGGACGATGGAGAGTCAGCCT........................................................................................................... 66

.....TGTGGACGATGGAGAGTCAG.............................................................................................................. 57

.....TGTGGACGATGGAGAGTCAGCC............................................................................................................ 187

......GTGGACGATGGAGAGTCAG.............................................................................................................. 2

......GTGGACGATGGAGAGTCAGC............................................................................................................. 1

......GTGGACGATGGAGAGTCAGCC............................................................................................................ 5

......GTGGACGATGGAGAGTCAGCCT........................................................................................................... 2

.......TGGACGATGGAGAGTCAGCCTC.......................................................................................................... 1

.......TGGACGATGGAGAGTCAGCC............................................................................................................ 2

.......TGGACGATGGAGAGTCAGCCT........................................................................................................... 1

............................CTTCCTGCCTCTCACTAGCTTC..................................................................................... 6

............................CTTCCTGCCTCTCACTAGC........................................................................................ 5

............................CTTCCTGCCTCTCACTAGCT....................................................................................... 10

............................CTTCCTGCCTCTCACTAGCTT...................................................................................... 85

.............................TTCCTGCCTCTCACTAGCTT...................................................................................... 2

.....................................................................TTAGTCTGGGAGGGAAGGA............................................... 1

...................................................................................AAGGAGCTATTGGTGGTCAGG............................... 1

........................................................................................GCTATTGGTGGTCAGGAATAGC......................... 5

........................................................................................GCTATTGGTGGTCAGGAAT............................ 5

........................................................................................GCTATTGGTGGTCAGGAATAG.......................... 104

........................................................................................GCTATTGGTGGTCAGGAATA........................... 12

..........................................................................................TATTGGTGGTCAGGAATAG.......................... 1

.............................................................................................................CGCACCCTTCATTTATCCACAC.... 1

.............................................................................................................CGCACCCTTCATTTATCCACACT... 6

.............................................................................................................CGCACCCTTCATTTATCCA....... 2

>ppt-MIR1219d_MI0004721_Physcomitrella_patens_miR1219d_stem-loop GSM313217 3

AGAGGCGUGUGCGGAGCACCGUGAGUCUUUUCCUGCCUCUCACUAGCUUCUUCCCUCCCUGCCUGAGUCGCAGUCUGGGAGGUAAGGAGCUAUUGGUAGACAGGAAUAGCGCGCACGUAGUCCCACGCAUGUUUCU

(((((((((((.((..(..((((.((((.((((((.((..((.(((((((((.((((((.(.(((.....))).).)))))).))))))))).))..)).)))))).)).)).))))..)..)).))))))))))) (-68.90)

...........................TTTTCCTGCCTCTCACTAGCT........................................................................................ 1

............................TTTCCTGCCTCTCACTAGCTTC...................................................................................... 2

............................TTTCCTGCCTCTCACTAGC......................................................................................... 1

............................TTTCCTGCCTCTCACTAGCT........................................................................................ 4

............................TTTCCTGCCTCTCACTAGCTT....................................................................................... 25

.............................TTCCTGCCTCTCACTAGCTT....................................................................................... 2

...................................................................TCGCAGTCTGGGAGGTAAGGA................................................ 1

................................................................................GGTAAGGAGCTATTGGTAGAC................................... 4

.....................................................................................GGAGCTATTGGTAGACAGGAAT............................. 1

.....................................................................................GGAGCTATTGGTAGACAGGAA.............................. 3

........................................................................................GCTATTGGTAGACAGGAATA............................ 10

........................................................................................GCTATTGGTAGACAGGAAT............................. 3

........................................................................................GCTATTGGTAGACAGGAATAGC.......................... 18

........................................................................................GCTATTGGTAGACAGGAATAG........................... 103

.........................................................................................CTATTGGTAGACAGGAATAG........................... 2

.........................................................................................CTATTGGTAGACAGGAATAGCG......................... 2

..........................................................................................TATTGGTAGACAGGAATAGCG......................... 8

..........................................................................................TATTGGTAGACAGGAATAG........................... 1

>ppt-MIR319e_MI0005665_Physcomitrella_patens_miR319e_stem-loop GSM313217 5

CUAGCCGUGGGAGCUCCUUCCGGUUCAAUAGUGGCUGAUGUGAGGUUGCACUGCUGCCGAUUCAAACUUCCGGCUUCCCUUUCUUAACACGACAGGGAAUCCGAAUGUCUGAUGCGGGAGCUGUGCGGUCUUCAACUUUAGCGCCUCUUGGACUGAAGGGAGCUCCCAAGUCUU

.......((((((((((((.((((((((.((..(((((..(((((((((((.(((.(((..(((.((...(((.((((((.((.......)).)))))).)))...)).)))..))).))).)))))).)))))...)))))..)).)))))))).))))))))))))...... (-86.10)

.........GGAGCTCCTTCCGGTTCAATA................................................................................................................................................ 1

..........GAGCTCCTTCCGGTTCAAT................................................................................................................................................. 15

..........GAGCTCCTTCCGGTTCAATA................................................................................................................................................ 50

..........GAGCTCCTTCCGGTTCAATAGT.............................................................................................................................................. 6

..........GAGCTCCTTCCGGTTCAATAG............................................................................................................................................... 46

...........AGCTCCTTCCGGTTCAATAGT.............................................................................................................................................. 1

...........AGCTCCTTCCGGTTCAATAG............................................................................................................................................... 1

...............................TGGCTGATGTGAGGTTGCAC........................................................................................................................... 2

.......................................................................................................AATGTCTGATGCGGGAGCTGT.................................................. 2

.......................................................................................................AATGTCTGATGCGGGAGCTG................................................... 1

............................................................................................................................GCGGTCTTCAACTTTAGCGCC............................. 1

............................................................................................................................GCGGTCTTCAACTTTAGCGCCT............................ 2
[truncated: 82,319 more chars]
