# Supplementary material for: Multiple distinct small RNAs originate from the same microRNA precursors
Source: Genome Biol. 2010 Aug 9;11(8):R81. doi: 10.1186/gb-2010-11-8-r81 (PMC2945783; doi:10.1186/gb-2010-11-8-r81)
Supplement: Additional file 5 — Supplemental File S4. This is a file for sequencing reads mapped and aligned to miRNA precursors that can produce miRNA-sibling small RNAs (msRNAs) in Medicago (mtr). The sequencing data were obtained from GEO; see Materials and methods for details. [file gb-2010-11-8-r81-S5.DOCX]

Zhang, et al., Multiple distinct small RNAs originate from the same microRNA precursors

Supplemental File 4 - Sequencing reads mapped and aligned to miRNA precursors that can produce

miRNA-like RNAs in *Medicago truncatula.*

>mtr-MIR319_MI0001751_Medicago_truncatula_miR319_stem-loop

ATTATTAGAAATAGATGAAGAGAGCTTCCTTCAGTCCACTCATGGAAGGGTAAGGGGTTTGAATTACCTGCTGACTCATTGATTCAAACACAATAGACAATTATGGGGTTATGCTATTGTGAATGTGTGAATGATGCAGGAGGTGAATTTCTTCCTTTTCTTCTTTGCTTGGACTGAAGGGAGCTCCCTTTTTCTATTTATAAATTA

************************************************************************************************************************************************************************TTGGACTGAAGGGAGCTCCC******************* mtr-MIR319 20

....(((.(((((((.((((.(((((((((((((((((..((.(((((((.((((((...((.((((((.(((..(((((.((.((..((((((((..((((....))))...))))))))..)).)).)))))..))).)))))).))..))))))))))))).))..))))))))))))))))).)))).))))))).))).... (-87.10)

....................AGAGCTTCCTTCAGTCCACTC...................................................................................................................................................................... 2 21

.............................................................................................................................TGTGAATGATGCAGGAGGTGA............................................................. 2 21

..................................................................................................................................................................CTTTGCTTGGACTGAAGGGA......................... 3 20

..................................................................................................................................................................CTTTGCTTGGACTGAAGGGAG........................ 2 21

...................................................................................................................................................................TTTGCTTGGACTGAAGGGA......................... 1 19

...................................................................................................................................................................TTTGCTTGGACTGAAGGGAG........................ 1 20

...................................................................................................................................................................TTTGCTTGGACTGAAGGGAGC....................... 15 21

....................................................................................................................................................................TTGCTTGGACTGAAGGGAGC....................... 3 20

.....................................................................................................................................................................TGCTTGGACTGAAGGGAGCTC..................... 1 21

.......................................................................................................................................................................CTTGGACTGAAGGGAGCT...................... 122 18

.......................................................................................................................................................................CTTGGACTGAAGGGAGCTC..................... 85 19

.......................................................................................................................................................................CTTGGACTGAAGGGAGCTCC.................... 55 20

.......................................................................................................................................................................CTTGGACTGAAGGGAGCTCCC................... 870 21

.......................................................................................................................................................................CTTGGACTGAAGGGAGCTCCCT.................. 1020 22

.......................................................................................................................................................................CTTGGACTGAAGGGAGCTCCCTT................. 34 23

........................................................................................................................................................................TTGGACTGAAGGGAGCTC..................... 172 18

........................................................................................................................................................................TTGGACTGAAGGGAGCTCC.................... 230 19

........................................................................................................................................................................TTGGACTGAAGGGAGCTCCC................... 2679 20

........................................................................................................................................................................TTGGACTGAAGGGAGCTCCCT.................. 23865 21

........................................................................................................................................................................TTGGACTGAAGGGAGCTCCCTT................. 201 22

........................................................................................................................................................................TTGGACTGAAGGGAGCTCCCTTT................ 3 23

.........................................................................................................................................................................TGGACTGAAGGGAGCTCC.................... 3 18

.........................................................................................................................................................................TGGACTGAAGGGAGCTCCC................... 59 19

.........................................................................................................................................................................TGGACTGAAGGGAGCTCCCT.................. 383 20

.........................................................................................................................................................................TGGACTGAAGGGAGCTCCCTT................. 47 21

..........................................................................................................................................................................GGACTGAAGGGAGCTCCC................... 6 18

..........................................................................................................................................................................GGACTGAAGGGAGCTCCCT.................. 35 19

...........................................................................................................................................................................GACTGAAGGGAGCTCCCT.................. 8 18

>mtr-MIR169d_MI0005578_Medicago_truncatula_miR169d_stem-loop

AGATGAAGCCAAGGATGACTTGCCGGTATAATAGTAATTTGCCACAAATCTAGATAGCTATTAGCTATGTTTGGATGGGCGGTGAGATTAACAAAATTACAGCAGCATTGTGATTTTGTTGATGCTTTAAAGTGTAGTTTTTATCAAAATTACAGTGGTTCACTGTAATTATGAGAATCTCACCGTCAATCTAAATATGCATTTAGTTTCATTTCCGGCAGGTCATCCTTCGGCTATATT

*****AAGCCAAGGATGACTTGCCGG********************************************************************************************************************************************************************************************************************** mtr-MIR169d 21

.((((.((((((((((((((((((((..(((((((.(((((.........))))).)))))))((.((((((((((.((((((((((((..((.((((((((.(.(((((((((((((.(((.(((.........)))..))).))))))))))))).)...)))))))).))..)))))))))))).))))))))))))..............)))))))))))))))).)))).)))) (-102.30)

....GAAGCCAAGGATGACTTGCCGG...................................................................................................................................................................................................................... 1 22

.....AAGCCAAGGATGACTTGC......................................................................................................................................................................................................................... 1 18

.....AAGCCAAGGATGACTTGCC........................................................................................................................................................................................................................ 1 19

.....AAGCCAAGGATGACTTGCCG....................................................................................................................................................................................................................... 2 20

.....AAGCCAAGGATGACTTGCCGG...................................................................................................................................................................................................................... 169 21

......AGCCAAGGATGACTTGCCGG...................................................................................................................................................................................................................... 5 20

.......................................................................TGGATGGGCGGTGAGATTAACA................................................................................................................................................... 1 22

........................................................................................................................................................................................................................GGCAGGTCATCCTTCGGC...... 2 18

........................................................................................................................................................................................................................GGCAGGTCATCCTTCGGCT..... 1 19

........................................................................................................................................................................................................................GGCAGGTCATCCTTCGGCTAT... 31 21

........................................................................................................................................................................................................................GGCAGGTCATCCTTCGGCTATA.. 1 22

.........................................................................................................................................................................................................................GCAGGTCATCCTTCGGCTATAT. 1 22

>mtr-MIR319b_MI0005596_Medicago_truncatula_miR319b_stem-loop

TAAGAGAGCTTTCTTTAGTCCACTCATGGGTGACAATAAGATTTCAATTAGCTGCTGACTCATTCATCCAAATGTTGAGTAAAATATATAGAAACATATACTCATCAAATGAGTGAATGATGCGGGAGACAAATTGAATCTTAAGTTTCCTATACTTGGACTGAAGGGAGCTCCCTTTTC

***********************************************************************************************************************************************************TTGGACTGAAGGGAGCTCCC***** mtr-MIR319b 20

.(((.(((((..((((((((((...(((((.(((..(((((((.(((((.(((.(((..(((((((..((..((.((((((.................)))))).))..))..)))))))..))).)).).)))))))))))).))).)))))...))))))))))..))))).)))... (-68.03)

....AGAGCTTTCTTTAGTCCACT............................................................................................................................................................ 2 20

....AGAGCTTTCTTTAGTCCACTC........................................................................................................................................................... 2 21

...............................................................................................................AGTGAATGATGCGGGAGACAA................................................ 2 21

.......................................................................................................................................................ATACTTGGACTGAAGGGAGCTCCC..... 2 24

.......................................................................................................................................................ATACTTGGACTGAAGGGAGCTCCCT.... 1 25

..........................................................................................................................................................CTTGGACTGAAGGGAGCT........ 122 18

..........................................................................................................................................................CTTGGACTGAAGGGAGCTC....... 85 19

..........................................................................................................................................................CTTGGACTGAAGGGAGCTCC...... 55 20

..........................................................................................................................................................CTTGGACTGAAGGGAGCTCCC..... 870 21

..........................................................................................................................................................CTTGGACTGAAGGGAGCTCCCT.... 1020 22

..........................................................................................................................................................CTTGGACTGAAGGGAGCTCCCTT... 34 23

...........................................................................................................................................................TTGGACTGAAGGGAGCTC....... 172 18

...........................................................................................................................................................TTGGACTGAAGGGAGCTCC...... 230 19

...........................................................................................................................................................TTGGACTGAAGGGAGCTCCC..... 2679 20

...........................................................................................................................................................TTGGACTGAAGGGAGCTCCCT.... 23865 21

...........................................................................................................................................................TTGGACTGAAGGGAGCTCCCTT... 201 22

...........................................................................................................................................................TTGGACTGAAGGGAGCTCCCTTT.. 3 23

............................................................................................................................................................TGGACTGAAGGGAGCTCC...... 3 18

............................................................................................................................................................TGGACTGAAGGGAGCTCCC..... 59 19

............................................................................................................................................................TGGACTGAAGGGAGCTCCCT.... 383 20

............................................................................................................................................................TGGACTGAAGGGAGCTCCCTT... 47 21

.............................................................................................................................................................GGACTGAAGGGAGCTCCC..... 6 18

.............................................................................................................................................................GGACTGAAGGGAGCTCCCT.... 35 19

..............................................................................................................................................................GACTGAAGGGAGCTCCCT.... 8 18

>mtr-MIR169l_MI0005631_Medicago_truncatula_miR169l_stem-loop

AGATGAAGCCAAGGATGACTTGCCGGTATAATAGTAATTTGCCACAAATCTAGATAGCTATTAGCTATGTTTGGATGGGCGGTGAGATTAACAAAATTACAGCAGCATTGTGATTTTGTTGATGCTTTAAAGTGTAGTTTTTATCAAAATTACAGTGGTTCACTGTAATTATGAGAATCTCACCGTCAATCTAAATATGCATTTAGTTTCATTTCCGGCAGGTCATCCTTCGGCTATATT

*****AAGCCAAGGATGACTTGCCGG********************************************************************************************************************************************************************************************************************** mtr-MIR169l 21

.((((.((((((((((((((((((((..(((((((.(((((.........))))).)))))))((.((((((((((.((((((((((((..((.((((((((.(.(((((((((((((.(((.(((.........)))..))).))))))))))))).)...)))))))).))..)))))))))))).))))))))))))..............)))))))))))))))).)))).)))) (-102.30)

....GAAGCCAAGGATGACTTGCCGG...................................................................................................................................................................................................................... 1 22

.....AAGCCAAGGATGACTTGC......................................................................................................................................................................................................................... 1 18

.....AAGCCAAGGATGACTTGCC........................................................................................................................................................................................................................ 1 19

.....AAGCCAAGGATGACTTGCCG....................................................................................................................................................................................................................... 2 20

.....AAGCCAAGGATGACTTGCCGG...................................................................................................................................................................................................................... 169 21

......AGCCAAGGATGACTTGCCGG...................................................................................................................................................................................................................... 5 20

.......................................................................TGGATGGGCGGTGAGATTAACA................................................................................................................................................... 1 22

........................................................................................................................................................................................................................GGCAGGTCATCCTTCGGC...... 2 18

........................................................................................................................................................................................................................GGCAGGTCATCCTTCGGCT..... 1 19

........................................................................................................................................................................................................................GGCAGGTCATCCTTCGGCTAT... 31 21

........................................................................................................................................................................................................................GGCAGGTCATCCTTCGGCTATA.. 1 22

.........................................................................................................................................................................................................................GCAGGTCATCCTTCGGCTATAT. 1 22

>mtr-MIR1510b_MI0010540_Medicago_truncatula_miR1510b_stem-loop

ACATCTAGTTAGTCATACCGATAGGCGAGAGAGATTTGCTACTGCAATTGTTGTTTTACCCGCTCCTCCCATCCCATGGATCCCTACCATGTGGACTCCATCATCATCAGATCCAATCTTGTGATAATAGAGTCACATGGTCGGTATCCCTGGAATGGAGGATCAGGTAAAACAACTTTTGCTTTAGAAATGATTCATTTCTCATCACCTTCATCAGGTTTATGATTTCTGTCTGT

**************************************************************************************************************************************ACATGGTCGGTATCCCTGGAA********************************************************************************* mtr-MIR1510b 21

(((..(((..(((((((((((((((.(((((((((...(((..((((..(((((((((((.(.((((((.((.(((.((((.((.((((((((.((((.((.(((((.((((...)))).))))).)).)))))))))))).)).)))).))).)))))))).).)))))))))))..))))..))).........))))))).)).)))..))).)))..)))))).)))..))) (-93.00)

...............................................TTGTTGTTTTACCCGCTCCTCC....................................................................................................................................................................... 1 22

...................................................TGTTTTACCCGCTCCTCC....................................................................................................................................................................... 4 18

...................................................TGTTTTACCCGCTCCTCCC...................................................................................................................................................................... 18 19

...................................................TGTTTTACCCGCTCCTCCCA..................................................................................................................................................................... 19 20

...................................................TGTTTTACCCGCTCCTCCCAT.................................................................................................................................................................... 106 21

...................................................TGTTTTACCCGCTCCTCCCATC................................................................................................................................................................... 44 22

....................................................GTTTTACCCGCTCCTCCCAT.................................................................................................................................................................... 3 20

....................................................GTTTTACCCGCTCCTCCCATC................................................................................................................................................................... 3 21

.....................................................TTTTACCCGCTCCTCCCATC................................................................................................................................................................... 1 20

.....................................................TTTTACCCGCTCCTCCCATCC.................................................................................................................................................................. 1 21

......................................................TTTACCCGCTCCTCCCATC................................................................................................................................................................... 1 19

.......................................................TTACCCGCTCCTCCCATCCCA................................................................................................................................................................ 3 21

.......................................................TTACCCGCTCCTCCCATCCCAT............................................................................................................................................................... 9 22

........................................................TACCCGCTCCTCCCATCCC................................................................................................................................................................. 1 19

........................................................TACCCGCTCCTCCCATCCCA................................................................................................................................................................ 3 20

........................................................TACCCGCTCCTCCCATCCCAT............................................................................................................................................................... 6 21

........................................................TACCCGCTCCTCCCATCCCATG.............................................................................................................................................................. 17 22

.........................................................ACCCGCTCCTCCCATCCCAT............................................................................................................................................................... 1 20

............................................................CGCTCCTCCCATCCCATGGA............................................................................................................................................................ 20 20

............................................................CGCTCCTCCCATCCCATGGAT........................................................................................................................................................... 2 21

............................................................CGCTCCTCCCATCCCATGGATC.......................................................................................................................................................... 13 22

............................................................CGCTCCTCCCATCCCATGGATCC......................................................................................................................................................... 1 23

..................................................................TCCCATCCCATGGATCCCTAC..................................................................................................................................................... 3 21

..................................................................TCCCATCCCATGGATCCCTACC.................................................................................................................................................... 1 22

.......................................................................TCCCATGGATCCCTACCATG................................................................................................................................................. 2 20

........................................................................CCCATGGATCCCTACCATGTG............................................................................................................................................... 3 21

........................................................................CCCATGGATCCCTACCATGTGG.............................................................................................................................................. 54 22

.........................................................................CCATGGATCCCTACCATGTG............................................................................................................................................... 1 20

.........................................................................CCATGGATCCCTACCATGTGG.............................................................................................................................................. 67 21

.........................................................................CCATGGATCCCTACCATGTGGA............................................................................................................................................. 1 22

...........................................................................ATGGATCCCTACCATGTGG.............................................................................................................................................. 1 19

............................................................................TGGATCCCTACCATGTGG.............................................................................................................................................. 1 18

............................................................................TGGATCCCTACCATGTGGA............................................................................................................................................. 5 19

............................................................................TGGATCCCTACCATGTGGAC............................................................................................................................................ 6 20

............................................................................TGGATCCCTACCATGTGGACT........................................................................................................................................... 3 21

............................................................................TGGATCCCTACCATGTGGACTC.......................................................................................................................................... 78 22

............................................................................TGGATCCCTACCATGTGGACTCC......................................................................................................................................... 14 23

............................................................................TGGATCCCTACCATGTGGACTCCAT....................................................................................................................................... 1 25

.............................................................................GGATCCCTACCATGTGGACTCC......................................................................................................................................... 3 22

..............................................................................GATCCCTACCATGTGGACT........................................................................................................................................... 1 19

..............................................................................GATCCCTACCATGTGGACTC.......................................................................................................................................... 1 20

..............................................................................GATCCCTACCATGTGGACTCC......................................................................................................................................... 1 21

..............................................................................GATCCCTACCATGTGGACTCCA........................................................................................................................................ 28 22

..............................................................................GATCCCTACCATGTGGACTCCATCA..................................................................................................................................... 1 25

...............................................................................ATCCCTACCATGTGGACTCC......................................................................................................................................... 2 20

...............................................................................ATCCCTACCATGTGGACTCCAT....................................................................................................................................... 2 22

................................................................................TCCCTACCATGTGGACTCC......................................................................................................................................... 11 19

................................................................................TCCCTACCATGTGGACTCCA........................................................................................................................................ 11 20

................................................................................TCCCTACCATGTGGACTCCAT....................................................................................................................................... 5 21

................................................................................TCCCTACCATGTGGACTCCATC...................................................................................................................................... 309 22

................................................................................TCCCTACCATGTGGACTCCATCA..................................................................................................................................... 10 23

.................................................................................CCCTACCATGTGGACTCCATCA..................................................................................................................................... 1 22

..................................................................................CCTACCATGTGGACTCCATC...................................................................................................................................... 2 20

.............................................................................................................................AATAGAGTCACATGGTCGG............................................................................................ 1 19

.............................................................................................................................AATAGAGTCACATGGTCGGTA.......................................................................................... 2 21

..............................................................................................................................ATAGAGTCACATGGTCGG............................................................................................ 1 18

..............................................................................................................................ATAGAGTCACATGGTCGGT........................................................................................... 1 19

..............................................................................................................................ATAGAGTCACATGGTCGGTAT......................................................................................... 7 21

...............................................................................................................................TAGAGTCACATGGTCGGTA.......................................................................................... 4 19

...............................................................................................................................TAGAGTCACATGGTCGGTAT......................................................................................... 9 20

...............................................................................................................................TAGAGTCACATGGTCGGTATC........................................................................................ 21 21

...............................................................................................................................TAGAGTCACATGGTCGGTATCCC...................................................................................... 3 23

...............................................................................................................................TAGAGTCACATGGTCGGTATCCCT..................................................................................... 2 24

................................................................................................................................AGAGTCACATGGTCGGTA.......................................................................................... 1 18

................................................................................................................................AGAGTCACATGGTCGGTAT......................................................................................... 2 19

................................................................................................................................AGAGTCACATGGTCGGTATC........................................................................................ 9 20

................................................................................................................................AGAGTCACATGGTCGGTATCC....................................................................................... 4 21

................................................................................................................................AGAGTCACATGGTCGGTATCCC...................................................................................... 10 22

................................................................................................................................AGAGTCACATGGTCGGTATCCCT..................................................................................... 6 23

................................................................................................................................AGAGTCACATGGTCGGTATCCCTG.................................................................................... 7 24

.................................................................................................................................GAGTCACATGGTCGGTAT......................................................................................... 4 18

.................................................................................................................................GAGTCACATGGTCGGTATC........................................................................................ 6 19

.................................................................................................................................GAGTCACATGGTCGGTATCC....................................................................................... 5 20

.................................................................................................................................GAGTCACATGGTCGGTATCCC...................................................................................... 67 21

.................................................................................................................................GAGTCACATGGTCGGTATCCCT..................................................................................... 2 22

..................................................................................................................................AGTCACATGGTCGGTATC........................................................................................ 1 18

..................................................................................................................................AGTCACATGGTCGGTATCC....................................................................................... 2 19

..................................................................................................................................AGTCACATGGTCGGTATCCC...................................................................................... 7 20

..................................................................................................................................AGTCACATGGTCGGTATCCCT..................................................................................... 12 21

..................................................................................................................................AGTCACATGGTCGGTATCCCTG.................................................................................... 1 22

...................................................................................................................................GTCACATGGTCGGTATCCC...................................................................................... 2 19

....................................................................................................................................TCACATGGTCGGTATCCCT..................................................................................... 1 19

....................................................................................................................................TCACATGGTCGGTATCCCTGG................................................................................... 7 21

....................................................................................................................................TCACATGGTCGGTATCCCTGGAA................................................................................. 2 23

....................................................................................................................................TCACATGGTCGGTATCCCTGGAAT................................................................................ 2 24

.....................................................................................................................................CACATGGTCGGTATCCCTGG................................................................................... 1 20

.....................................................................................................................................CACATGGTCGGTATCCCTGGA.................................................................................. 7 21

......................................................................................................................................ACATGGTCGGTATCCCTGG................................................................................... 26 19

......................................................................................................................................ACATGGTCGGTATCCCTGGA.................................................................................. 146 20

......................................................................................................................................ACATGGTCGGTATCCCTGGAA................................................................................. 724 21

......................................................................................................................................ACATGGTCGGTATCCCTGGAAT................................................................................ 5 22

.......................................................................................................................................CATGGTCGGTATCCCTGGAA................................................................................. 2 20

.......................................................................................................................................CATGGTCGGTATCCCTGGAAT................................................................................ 3 21

.........................................................................................................................................TGGTCGGTATCCCTGGAA................................................................................. 3 18

.........................................................................................................................................TGGTCGGTATCCCTGGAAT................................................................................ 5 19

.........................................................................................................................................TGGTCGGTATCCCTGGAATG............................................................................... 2 20

.........................................................................................................................................TGGTCGGTATCCCTGGAATGGAGG........................................................................... 1 24

.................................................................................................................................................ATCCCTGGAATGGAGGATCAG...................................................................... 1 21

..................................................................................................................................................TCCCTGGAATGGAGGATCAGGT.................................................................... 3 22

....................................................................................................................................................CCTGGAATGGAGGATCAG...................................................................... 2 18

....................................................................................................................................................CCTGGAATGGAGGATCAGG..................................................................... 2 19

....................................................................................................................................................CCTGGAATGGAGGATCAGGT.................................................................... 1 20

.....................................................................................................................................................CTGGAATGGAGGATCAGGTAA.................................................................. 1 21

......................................................................................................................................................TGGAATGGAGGATCAGGT.................................................................... 6 18

......................................................................................................................................................TGGAATGGAGGATCAGGTA................................................................... 15 19

......................................................................................................................................................TGGAATGGAGGATCAGGTAA.................................................................. 36 20

......................................................................................................................................................TGGAATGGAGGATCAGGTAAA................................................................. 410 21

......................................................................................................................................................TGGAATGGAGGATCAGGTAAAA................................................................ 42 22

.......................................................................................................................................................GGAATGGAGGATCAGGTAAA................................................................. 3 20

.......................................................................................................................................................GGAATGGAGGATCAGGTAAAA................................................................ 4 21

........................................................................................................................................................GAATGGAGGATCAGGTAAAA................................................................ 3 20

.........................................................................................................................................................AATGGAGGATCAGGTAAA................................................................. 1 18

.........................................................................................................................................................AATGGAGGATCAGGTAAAA................................................................ 1 19

..........................................................................................................................................................ATGGAGGATCAGGTAAAACA.............................................................. 1 20

..........................................................................................................................................................ATGGAGGATCAGGTAAAACAA............................................................. 1 21

...........................................................................................................................................................TGGAGGATCAGGTAAAAC............................................................... 6 18

...........................................................................................................................................................TGGAGGATCAGGTAAAACA.............................................................. 11 19

...........................................................................................................................................................TGGAGGATCAGGTAAAACAA............................................................. 4 20

...........................................................................................................................................................TGGAGGATCAGGTAAAACAAC............................................................ 99 21

.............................................................................................................................................................GAGGATCAGGTAAAACAACT........................................................... 2 20

>mtr-MIR2087_MI0010544_Medicago_truncatula_miR2087_stem-loop

GCTTGATGAAAACTGCTCTTGACACATATGATAGAGCTAAAAAACATATGTAAAAGACGAAAATGCCCATGTAGCAGTTTACTACCGAAGTAAAGAACCGGCTGCAGTTAACTGCTGAAGTGTGAAGTTTTTTTTTTTTTTTCTTTTTCGTCGGCAGTTAACTGCAGTCGGTTTCTTACTTCGGTAGTTAACCGTTGCAGGGGCATTTTCGTCATTTACATGTGTTTTTCAGCCCTATCATATGTGTCAACAGCAATTTTCTAGC

**************************************************************************************GAAGTAAAGAACCGGCTGCAG************************************************************************************************************************************************************** mtr-MIR2087 21

(((....(((((.((((.((((((((((((((((.(((.(((((((((((((((.(((((((((((((.((((((.(((.(((((((((((((..(((((((((((((((((((((((.....((((...............))))....)))))))))))))))))))))))..))))))))))))).))).)))))).))))))))))))).))))))))))))))).))).)))))))))))))))).)))).))))).))) (-159.16)

.............................................ATATGTAAAAGACGAAAAT......................................................................................................................................................................................................... 1 19

..............................................TATGTAAAAGACGAAAAT......................................................................................................................................................................................................... 1 18

...............................................ATGTAAAAGACGAAAATGC....................................................................................................................................................................................................... 1 19

...................................................................CATGTAGCAGTTTACTACCGAA................................................................................................................................................................................ 1 22

..............................................................................TTACTACCGAAGTAAAGAACC...................................................................................................................................................................... 1 21

..................................................................................TACCGAAGTAAAGAACCGGC................................................................................................................................................................... 1 20

....................................................................................CCGAAGTAAAGAACCGGCTGC................................................................................................................................................................ 14 21

....................................................................................CCGAAGTAAAGAACCGGCTGCA............................................................................................................................................................... 1 22

.....................................................................................CGAAGTAAAGAACCGGCT.................................................................................................................................................................. 2 18

.....................................................................................CGAAGTAAAGAACCGGCTGC................................................................................................................................................................ 1 20

.....................................................................................CGAAGTAAAGAACCGGCTGCA............................................................................................................................................................... 2 21

......................................................................................GAAGTAAAGAACCGGCTGCA............................................................................................................................................................... 5 20

......................................................................................GAAGTAAAGAACCGGCTGCAG.............................................................................................................................................................. 35 21

......................................................................................GAAGTAAAGAACCGGCTGCAGT............................................................................................................................................................. 1 22

.......................................................................................AAGTAAAGAACCGGCTGCA............................................................................................................................................................... 4 19

.......................................................................................AAGTAAAGAACCGGCTGCAGT............................................................................................................................................................. 12 21

.......................................................................................AAGTAAAGAACCGGCTGCAGTTAA.......................................................................................................................................................... 2 24

.........................................................................................GTAAAGAACCGGCTGCAG.............................................................................................................................................................. 1 18

.........................................................................................GTAAAGAACCGGCTGCAGTTAAC......................................................................................................................................................... 1 23

.........................................................................................GTAAAGAACCGGCTGCAGTTAACT........................................................................................................................................................ 1 24

..........................................................................................TAAAGAACCGGCTGCAGTTAA.......................................................................................................................................................... 4 21

..........................................................................................TAAAGAACCGGCTGCAGTTAAC......................................................................................................................................................... 1 22

..........................................................................................TAAAGAACCGGCTGCAGTTAACT........................................................................................................................................................ 1 23

..........................................................................................TAAAGAACCGGCTGCAGTTAACTG....................................................................................................................................................... 1 24

..........................................................................................TAAAGAACCGGCTGCAGTTAACTGC...................................................................................................................................................... 1 25

...........................................................................................AAAGAACCGGCTGCAGTTAA.......................................................................................................................................................... 4 20

...........................................................................................AAAGAACCGGCTGCAGTTAAC......................................................................................................................................................... 5 21

...........................................................................................AAAGAACCGGCTGCAGTTAACT........................................................................................................................................................ 5 22

...........................................................................................AAAGAACCGGCTGCAGTTAACTG....................................................................................................................................................... 1 23

...........................................................................................AAAGAACCGGCTGCAGTTAACTGC...................................................................................................................................................... 11 24

............................................................................................AAGAACCGGCTGCAGTTAA.......................................................................................................................................................... 1 19

............................................................................................AAGAACCGGCTGCAGTTAAC......................................................................................................................................................... 6 20

............................................................................................AAGAACCGGCTGCAGTTAACT........................................................................................................................................................ 1 21

............................................................................................AAGAACCGGCTGCAGTTAACTGC...................................................................................................................................................... 2 23

.............................................................................................AGAACCGGCTGCAGTTAA.......................................................................................................................................................... 3 18

.............................................................................................AGAACCGGCTGCAGTTAAC......................................................................................................................................................... 4 19

.............................................................................................AGAACCGGCTGCAGTTAACT........................................................................................................................................................ 3 20

.............................................................................................AGAACCGGCTGCAGTTAACTG....................................................................................................................................................... 7 21

.............................................................................................AGAACCGGCTGCAGTTAACTGC...................................................................................................................................................... 1 22

.............................................................................................AGAACCGGCTGCAGTTAACTGCT..................................................................................................................................................... 2 23

.............................................................................................AGAACCGGCTGCAGTTAACTGCTG.................................................................................................................................................... 4 24

..............................................................................................GAACCGGCTGCAGTTAACT........................................................................................................................................................ 5 19

..............................................................................................GAACCGGCTGCAGTTAACTGC...................................................................................................................................................... 2 21

...............................................................................................AACCGGCTGCAGTTAACT........................................................................................................................................................ 2 18

................................................................................................................................................TTTTCGTCGGCAGTTAAC....................................................................................................... 1 18

................................................................................................................................................................ACTGCAGTCGGTTTCTTACT..................................................................................... 1 20

................................................................................................................................................................ACTGCAGTCGGTTTCTTACTTC................................................................................... 1 22

.................................................................................................................................................................CTGCAGTCGGTTTCTTACTTC................................................................................... 2 21

..................................................................................................................................................................TGCAGTCGGTTTCTTACTTC................................................................................... 3 20

..................................................................................................................................................................TGCAGTCGGTTTCTTACTTCG.................................................................................. 1 21

..................................................................................................................................................................TGCAGTCGGTTTCTTACTTCGG................................................................................. 1 22

....................................................................................................................................................................CAGTCGGTTTCTTACTTCGGT................................................................................ 4 21

....................................................................................................................................................................CAGTCGGTTTCTTACTTCGGTA............................................................................... 3 22

>mtr-MIR2088_MI0010545_Medicago_truncatula_miR2088_stem-loop

TCTCTGTATCTCGAAGTAGACGGCGGCTTAACCGATGTAGGCCTAGATTACATTGGACAAAACAGTCCAAACTTGAGATGGTTACTTCTTGGTCCAATGTAATCTAGGTCTACATCTGTTAAGTCTCTAAAGTATTCTGAAAGAGA

**************************************AGGCCTAGATTACATTGGAC**************************************************************************************** mtr-MIR2088 20

(((((...((..(((......((.((((((((.(((((((((((((((((((((((((.....(((...((((......))))))).....))))))))))))))))))))))))).)))))))).))......))).)).))))) (-64.20)

.CTCTGTATCTCGAAGTAGACGGCG......................................................................................................................... 1 24

..............AGTAGACGGCGGCTTAACCGATGT............................................................................................................ 1 24

.................AGACGGCGGCTTAACCGATGT............................................................................................................ 1 21

.....................................TAGGCCTAGATTACATTGGAC........................................................................................ 4 21

......................................AGGCCTAGATTACATTGG.......................................................................................... 1 18

......................................AGGCCTAGATTACATTGGA......................................................................................... 2 19

......................................AGGCCTAGATTACATTGGAC........................................................................................ 11 20

......................................AGGCCTAGATTACATTGGACA....................................................................................... 8 21

............................................................................................TCCAATGTAATCTAGGTC.................................... 1 18

............................................................................................TCCAATGTAATCTAGGTCTA.................................. 11 20

............................................................................................TCCAATGTAATCTAGGTCTAC................................. 569 21

............................................................................................TCCAATGTAATCTAGGTCTACA................................ 22 22

............................................................................................TCCAATGTAATCTAGGTCTACAT............................... 2 23

.............................................................................................CCAATGTAATCTAGGTCTA.................................. 1 19

.............................................................................................CCAATGTAATCTAGGTCTACA................................ 1 21

..............................................................................................CAATGTAATCTAGGTCTACA................................ 1 20

.................................................................................................TGTAATCTAGGTCTACATC.............................. 1 19

.................................................................................................TGTAATCTAGGTCTACATCTG............................ 8 21

>mtr-MIR2089_MI0010546_Medicago_truncatula_miR2089_stem-loop

GCAGGCAACCAAGCATTCAAGATCTAGAGCAAGATTTATTTTACCTATTCCACCAATTCCATTGATCCTGGTTTCAAGATCTGAAAGTGAATCTGGGATGTATAGGATTGGTGTAATAGGTAAAACAACACTTGCACTAGAAGCAAAATATATATTTGTTTGC

****************************************TTACCTATTCCACCAATTCCAT***************************************************************************************************** mtr-MIR2089 22

((((((((..............(((((.(((((..((.(((((((((((.(((((((((.((..((((..(.((((...........)))).)..))))..)).))))))))).))))))))))).))..))))).)))))..............)))))))) (-54.99)

........................................TTACCTATTCCACCAATT......................................................................................................... 2 18

........................................TTACCTATTCCACCAATTC........................................................................................................ 5 19

........................................TTACCTATTCCACCAATTCC....................................................................................................... 15 20

........................................TTACCTATTCCACCAATTCCA...................................................................................................... 36 21

........................................TTACCTATTCCACCAATTCCAT..................................................................................................... 154 22

........................................TTACCTATTCCACCAATTCCATT.................................................................................................... 4 23

.........................................TACCTATTCCACCAATTCCAT..................................................................................................... 1 21

..............................................................TGATCCTGGTTTCAAGATCT................................................................................. 1 20

.......................................................................................................AGGATTGGTGTAATAGGTAAA....................................... 1 21

.......................................................................................................AGGATTGGTGTAATAGGTAAAA...................................... 4 22

...........................................................................................................TTGGTGTAATAGGTAAAA...................................... 1 18

>mtr-MIR159a_MI0010696_Medicago_truncatula_miR159a_stem-loop

GGGGTGGAGCTTCCTTTAGTCCAAATATGGATCTTGCTATGTTGATAGAGCTGCTTAGCTATGGGTCCCTCAACTCTACCCATCTTGTTCTTTGTGGTAGTTTTGTGGCTTCCATATCTAGGGAGCCTTATCACCTTTAGTTTAATCTTTCTTTGGATTGAAGGGAGCTCTACATCTTGCTCT

*******************************************************************************************************************************************************TTTGGATTGAAGGGAGCTCTA*********** mtr-MIR159a 21

(..((((((((((((((((((((((...((((...((((.(.((((((.(((.(((((.((((((..((.(((..(((((((..........)).)))))..))).))..)))))).))))).))).)))))).)..))))...))))...))))))))))))))))))))))..)....... (-80.40)

......GAGCTTCCTTTAGTCCAAA.............................................................................................................................................................. 1 19

......GAGCTTCCTTTAGTCCAAATA............................................................................................................................................................ 1 21

...........................TGGATCTTGCTATGTTGATAG....................................................................................................................................... 2 21

................................................AGCTGCTTAGCTATGGGTC.................................................................................................................... 1 19

................................................AGCTGCTTAGCTATGGGTCCC.................................................................................................................. 6 21

............................................................................................................CTTCCATATCTAGGGAGC......................................................... 5 18

............................................................................................................CTTCCATATCTAGGGAGCC........................................................ 1 19

............................................................................................................CTTCCATATCTAGGGAGCCT....................................................... 3 20

............................................................................................................CTTCCATATCTAGGGAGCCTT...................................................... 46 21

............................................................................................................CTTCCATATCTAGGGAGCCTTA..................................................... 2 22

.....................................................................................................................................................TCTTTGGATTGAAGGGAGC............... 1 19

......................................................................................................................................................CTTTGGATTGAAGGGAGC............... 4 18

......................................................................................................................................................CTTTGGATTGAAGGGAGCT.............. 3 19

......................................................................................................................................................CTTTGGATTGAAGGGAGCTC............. 3 20

......................................................................................................................................................CTTTGGATTGAAGGGAGCTCT............ 10 21

......................................................................................................................................................CTTTGGATTGAAGGGAGCTCTA........... 11 22

.......................................................................................................................................................TTTGGATTGAAGGGAGCT.............. 19586 18

.......................................................................................................................................................TTTGGATTGAAGGGAGCTC............. 8144 19

.......................................................................................................................................................TTTGGATTGAAGGGAGCTCT............ 2943 20

.......................................................................................................................................................TTTGGATTGAAGGGAGCTCTA........... 37241 21

.......................................................................................................................................................TTTGGATTGAAGGGAGCTCTAC.......... 167 22

.......................................................................................................................................................TTTGGATTGAAGGGAGCTCTACA......... 5 23

.......................................................................................................................................................TTTGGATTGAAGGGAGCTCTACAT........ 1 24

........................................................................................................................................................TTGGATTGAAGGGAGCTC............. 111 18

........................................................................................................................................................TTGGATTGAAGGGAGCTCT............ 45 19

........................................................................................................................................................TTGGATTGAAGGGAGCTCTA........... 669 20

........................................................................................................................................................TTGGATTGAAGGGAGCTCTAC.......... 1 21

.........................................................................................................................................................TGGATTGAAGGGAGCTCT............ 5 18

.........................................................................................................................................................TGGATTGAAGGGAGCTCTA........... 125 19

.........................................................................................................................................................TGGATTGAAGGGAGCTCTACA......... 1 21

.........................................................................................................................................................TGGATTGAAGGGAGCTCTACATCT...... 3 24

..........................................................................................................................................................GGATTGAAGGGAGCTCTA........... 51 18
