# Supplementary material for: Multiple distinct small RNAs originate from the same microRNA precursors
Source: Genome Biol. 2010 Aug 9;11(8):R81. doi: 10.1186/gb-2010-11-8-r81 (PMC2945783; doi:10.1186/gb-2010-11-8-r81)
Supplement: Additional file 6 — Supplemental File S5. This is a file for sequencing reads mapped and aligned to miRNA precursors that can produce miRNA-sibling small RNAs (msRNAs) in Populus (ptc). The sequencing data were obtained from GEO; see Materials and methods for details. [file gb-2010-11-8-r81-S6.DOCX]

Zhang, et al., Multiple distinct small RNAs originate from the same microRNA precursors

Supplemental File 5 - Sequencing reads mapped and aligned to miRNA precursors that can produce

miRNA-like RNAs in *Populus trichocarpa.*

>ptc-MIR159a_MI0002195_Populus_trichocarpa_miR159a_stem-loop

GAUUAGGGAGUGGAGCUCCUUGAAGUCCAAUAGAGGUUCUUGCUGGGUAGAUUAAGCUGCUAAGCUAUGGAUCCACAGUCCUAUCUAUCAACUGAAGGAUAGGUUUGCGGCUUGCAUAUCUCAGGAGCUUUAUUGCCUAAUGUUAGAUCCCUUUUUGGAUUGAAGGGAGCUCUAAACCCAUAA

.....(((..(((((((((((..(((((((.(((((.(((.(((((((((...(((((.((.((.((((.(.((.(((.(((((((.((....)).))))))).))).))..).)))).)).)).)))))..)))))))..)).)))..))))))))))))..)))))))))))..))).... (-79.30)

............GAGCTCCTTGAAGTCCAATAG...................................................................................................................................................... 11

............GAGCTCCTTGAAGTCCAATAGA..................................................................................................................................................... 1

.............AGCTCCTTGAAGTCCAATAGA..................................................................................................................................................... 1

.............AGCTCCTTGAAGTCCAATAG...................................................................................................................................................... 14

.............AGCTCCTTGAAGTCCAAT........................................................................................................................................................ 8

.............AGCTCCTTGAAGTCCAATA....................................................................................................................................................... 17

...............CTCCTTGAAGTCCAATAGAGGT.................................................................................................................................................. 3

....................................................TAAGCTGCTAAGCTATGGATC.............................................................................................................. 2

......................................................AGCTGCTAAGCTATGGATC.............................................................................................................. 2

......................................................AGCTGCTAAGCTATGGATCC............................................................................................................. 1

..............................................................................................................CTTGCATATCTCAGGAGCTTT.................................................... 4

..............................................................................................................CTTGCATATCTCAGGAGCTTTAT.................................................. 1

...............................................................................................................TTGCATATCTCAGGAGCTTT.................................................... 3

...............................................................................................................TTGCATATCTCAGGAGCTTT.................................................... 2

.......................................................................................................................................................TTTTTGGATTGAAGGGAGCTCT.......... 4

.......................................................................................................................................................TTTTTGGATTGAAGGGAGCTC........... 2

........................................................................................................................................................TTTTGGATTGAAGGGAGCTCT.......... 156

........................................................................................................................................................TTTTGGATTGAAGGGAGCTC........... 1

........................................................................................................................................................TTTTGGATTGAAGGGAGCTCTA......... 208

........................................................................................................................................................TTTTGGATTGAAGGGAGCTCT.......... 1

.........................................................................................................................................................TTTGGATTGAAGGGAGCTCT.......... 17694

.........................................................................................................................................................TTTGGATTGAAGGGAGCTCTA......... 22755

.........................................................................................................................................................TTTGGATTGAAGGGAGCT............ 111

.........................................................................................................................................................TTTGGATTGAAGGGAGCTCTAA........ 50

.........................................................................................................................................................TTTGGATTGAAGGGAGCTCTAAA....... 4

.........................................................................................................................................................TTTGGATTGAAGGGAGCTC........... 40

.........................................................................................................................................................TTTGGATTGAAGGGAGCTCTA......... 272

.........................................................................................................................................................TTTGGATTGAAGGGAGCTCT.......... 224

.........................................................................................................................................................TTTGGATTGAAGGGAGCTC........... 1

.........................................................................................................................................................TTTGGATTGAAGGGAGCTCT.......... 111

.........................................................................................................................................................TTTGGATTGAAGGGAGCTCTA......... 107

.........................................................................................................................................................TTTGGATTGAAGGGAGCTC........... 2

.........................................................................................................................................................TTTGGATTGAAGGGAGCTCTAA........ 1

..........................................................................................................................................................TTGGATTGAAGGGAGCTCTAAA....... 115

..........................................................................................................................................................TTGGATTGAAGGGAGCTCTAA........ 106

..........................................................................................................................................................TTGGATTGAAGGGAGCTC........... 4

..........................................................................................................................................................TTGGATTGAAGGGAGCTCTA......... 634

..........................................................................................................................................................TTGGATTGAAGGGAGCTCT.......... 398

..........................................................................................................................................................TTGGATTGAAGGGAGCTCT.......... 136

..........................................................................................................................................................TTGGATTGAAGGGAGCTCTA......... 48

..........................................................................................................................................................TTGGATTGAAGGGAGCTCT.......... 51

..........................................................................................................................................................TTGGATTGAAGGGAGCTCTA......... 17

...........................................................................................................................................................TGGATTGAAGGGAGCTCTA......... 8

...........................................................................................................................................................TGGATTGAAGGGAGCTCT.......... 5

...........................................................................................................................................................TGGATTGAAGGGAGCTCTAAA....... 22

...........................................................................................................................................................TGGATTGAAGGGAGCTCT.......... 33

...........................................................................................................................................................TGGATTGAAGGGAGCTCTA......... 5

...........................................................................................................................................................TGGATTGAAGGGAGCTCT.......... 18

...........................................................................................................................................................TGGATTGAAGGGAGCTCTA......... 4

............................................................................................................................................................GGATTGAAGGGAGCTCTA......... 8

............................................................................................................................................................GGATTGAAGGGAGCTCTA......... 14

>ptc-MIR159b_MI0002196_Populus_trichocarpa_miR159b_stem-loop

GAUUAUGGAGUGGAGCUCCUUGAAGUCCAAUAGAAGCUCCUGCUGGGUAGAUCGAGCUGCUGAGCUAUGAAUCCCACAGCCCUAUCACCAUCAGUCAUUUUGAUGGGCCUGCGGCUUGCAUAUCUCAGGAGCUUUAUUACCUAAUGUUAGAUCUUUUUUUGGAUUGAAGGGAGCUCUAAACCUUUGA

......((..(((((((((((..(((((((.(((((.((..(((((((((...(((((.(((((.((((....((.(((.(((((((.............))))))).))).))....)))).))))).)))))..)))))))..))..)).))))).)))))))..)))))))))))..))..... (-78.42)

............GAGCTCCTTGAAGTCCAATAG.......................................................................................................................................................... 11

............GAGCTCCTTGAAGTCCAATAGA......................................................................................................................................................... 1

.............AGCTCCTTGAAGTCCAATAGA......................................................................................................................................................... 1

.............AGCTCCTTGAAGTCCAATAG.......................................................................................................................................................... 14

.............AGCTCCTTGAAGTCCAAT............................................................................................................................................................ 8

.............AGCTCCTTGAAGTCCAATA........................................................................................................................................................... 17

.................................AAGCTCCTGCTGGGTAGATCGA.................................................................................................................................... 1

.................................AAGCTCCTGCTGGGTAGATCG..................................................................................................................................... 1

......................................................AGCTGCTGAGCTATGAATCCC................................................................................................................ 7

......................................................AGCTGCTGAGCTATGAATCC................................................................................................................. 11

......................................................AGCTGCTGAGCTATGAATC.................................................................................................................. 4

..................................................................................................................CTTGCATATCTCAGGAGCTTT.................................................... 4

..................................................................................................................CTTGCATATCTCAGGAGCTTTAT.................................................. 1

...................................................................................................................TTGCATATCTCAGGAGCTTT.................................................... 3

...................................................................................................................TTGCATATCTCAGGAGCTTT.................................................... 2

...........................................................................................................................................................TTTTTGGATTGAAGGGAGCTCT.......... 4

...........................................................................................................................................................TTTTTGGATTGAAGGGAGCTC........... 2

............................................................................................................................................................TTTTGGATTGAAGGGAGCTCT.......... 156

............................................................................................................................................................TTTTGGATTGAAGGGAGCTC........... 1

............................................................................................................................................................TTTTGGATTGAAGGGAGCTCTA......... 208

............................................................................................................................................................TTTTGGATTGAAGGGAGCTCT.......... 1

.............................................................................................................................................................TTTGGATTGAAGGGAGCTCT.......... 17694

.............................................................................................................................................................TTTGGATTGAAGGGAGCTCTA......... 22755

.............................................................................................................................................................TTTGGATTGAAGGGAGCT............ 111

.............................................................................................................................................................TTTGGATTGAAGGGAGCTCTAA........ 50

.............................................................................................................................................................TTTGGATTGAAGGGAGCTCTAAA....... 4

.............................................................................................................................................................TTTGGATTGAAGGGAGCTC........... 40

.............................................................................................................................................................TTTGGATTGAAGGGAGCTCTA......... 272

.............................................................................................................................................................TTTGGATTGAAGGGAGCTCT.......... 224

.............................................................................................................................................................TTTGGATTGAAGGGAGCTC........... 1

.............................................................................................................................................................TTTGGATTGAAGGGAGCTCT.......... 111

.............................................................................................................................................................TTTGGATTGAAGGGAGCTCTA......... 107

.............................................................................................................................................................TTTGGATTGAAGGGAGCTC........... 2

.............................................................................................................................................................TTTGGATTGAAGGGAGCTCTAA........ 1

..............................................................................................................................................................TTGGATTGAAGGGAGCTCTAAA....... 115

..............................................................................................................................................................TTGGATTGAAGGGAGCTCTAA........ 106

..............................................................................................................................................................TTGGATTGAAGGGAGCTC........... 4

..............................................................................................................................................................TTGGATTGAAGGGAGCTCTA......... 634

..............................................................................................................................................................TTGGATTGAAGGGAGCTCT.......... 398

..............................................................................................................................................................TTGGATTGAAGGGAGCTCT.......... 136

..............................................................................................................................................................TTGGATTGAAGGGAGCTCTA......... 48

..............................................................................................................................................................TTGGATTGAAGGGAGCTCT.......... 51

..............................................................................................................................................................TTGGATTGAAGGGAGCTCTA......... 17

...............................................................................................................................................................TGGATTGAAGGGAGCTCTA......... 8

...............................................................................................................................................................TGGATTGAAGGGAGCTCT.......... 5

...............................................................................................................................................................TGGATTGAAGGGAGCTCTAAA....... 22

...............................................................................................................................................................TGGATTGAAGGGAGCTCT.......... 33

...............................................................................................................................................................TGGATTGAAGGGAGCTCTA......... 5

...............................................................................................................................................................TGGATTGAAGGGAGCTCT.......... 18

...............................................................................................................................................................TGGATTGAAGGGAGCTCTA......... 4

................................................................................................................................................................GGATTGAAGGGAGCTCTA......... 8

................................................................................................................................................................GGATTGAAGGGAGCTCTA......... 14

>ptc-MIR159c_MI0002197_Populus_trichocarpa_miR159c_stem-loop

GAUUAGGGAGUGGAGCUCCUUGAAGUCCAAUAGAGGUUCUUGCUGGGUAGAUUAAGCUGCUAAGCUAUGGAUCCACAGUCCUAUCUAUCAACCGAAGGAUAGGUUUGCGGCUUGCAUAUCUCAGGAGCUUUAUUGCCUAAUGUUAGAUCCCUUUUUGGAUUGAAGGGAGCUCUAAACCCAUAA

.....(((..(((((((((((..(((((((.(((((.(((.(((((((((...(((((.((.((.((((.(.((.(((.(((((((.((....)).))))))).))).))..).)))).)).)).)))))..)))))))..)).)))..))))))))))))..)))))))))))..))).... (-79.00)

............GAGCTCCTTGAAGTCCAATAG...................................................................................................................................................... 11

............GAGCTCCTTGAAGTCCAATAGA..................................................................................................................................................... 1

.............AGCTCCTTGAAGTCCAATAGA..................................................................................................................................................... 1

.............AGCTCCTTGAAGTCCAATAG...................................................................................................................................................... 14

.............AGCTCCTTGAAGTCCAAT........................................................................................................................................................ 8

.............AGCTCCTTGAAGTCCAATA....................................................................................................................................................... 17

...............CTCCTTGAAGTCCAATAGAGGT.................................................................................................................................................. 3

....................................................TAAGCTGCTAAGCTATGGATC.............................................................................................................. 2

......................................................AGCTGCTAAGCTATGGATC.............................................................................................................. 2

......................................................AGCTGCTAAGCTATGGATCC............................................................................................................. 1

..............................................................................................................CTTGCATATCTCAGGAGCTTT.................................................... 4

..............................................................................................................CTTGCATATCTCAGGAGCTTTAT.................................................. 1

...............................................................................................................TTGCATATCTCAGGAGCTTT.................................................... 3

...............................................................................................................TTGCATATCTCAGGAGCTTT.................................................... 2

.......................................................................................................................................................TTTTTGGATTGAAGGGAGCTCT.......... 4

.......................................................................................................................................................TTTTTGGATTGAAGGGAGCTC........... 2

........................................................................................................................................................TTTTGGATTGAAGGGAGCTCT.......... 156

........................................................................................................................................................TTTTGGATTGAAGGGAGCTC........... 1

........................................................................................................................................................TTTTGGATTGAAGGGAGCTCTA......... 208

........................................................................................................................................................TTTTGGATTGAAGGGAGCTCT.......... 1

.........................................................................................................................................................TTTGGATTGAAGGGAGCTCT.......... 17694

.........................................................................................................................................................TTTGGATTGAAGGGAGCTCTA......... 22755

.........................................................................................................................................................TTTGGATTGAAGGGAGCT............ 111

.........................................................................................................................................................TTTGGATTGAAGGGAGCTCTAA........ 50

.........................................................................................................................................................TTTGGATTGAAGGGAGCTCTAAA....... 4

.........................................................................................................................................................TTTGGATTGAAGGGAGCTC........... 40

.........................................................................................................................................................TTTGGATTGAAGGGAGCTCTA......... 272

.........................................................................................................................................................TTTGGATTGAAGGGAGCTCT.......... 224

.........................................................................................................................................................TTTGGATTGAAGGGAGCTC........... 1

.........................................................................................................................................................TTTGGATTGAAGGGAGCTCT.......... 111

.........................................................................................................................................................TTTGGATTGAAGGGAGCTCTA......... 107

.........................................................................................................................................................TTTGGATTGAAGGGAGCTC........... 2

.........................................................................................................................................................TTTGGATTGAAGGGAGCTCTAA........ 1

..........................................................................................................................................................TTGGATTGAAGGGAGCTCTAAA....... 115

..........................................................................................................................................................TTGGATTGAAGGGAGCTCTAA........ 106

..........................................................................................................................................................TTGGATTGAAGGGAGCTC........... 4

..........................................................................................................................................................TTGGATTGAAGGGAGCTCTA......... 634

..........................................................................................................................................................TTGGATTGAAGGGAGCTCT.......... 398

..........................................................................................................................................................TTGGATTGAAGGGAGCTCT.......... 136

..........................................................................................................................................................TTGGATTGAAGGGAGCTCTA......... 48

..........................................................................................................................................................TTGGATTGAAGGGAGCTCT.......... 51

..........................................................................................................................................................TTGGATTGAAGGGAGCTCTA......... 17

...........................................................................................................................................................TGGATTGAAGGGAGCTCTA......... 8

...........................................................................................................................................................TGGATTGAAGGGAGCTCT.......... 5

...........................................................................................................................................................TGGATTGAAGGGAGCTCTAAA....... 22

...........................................................................................................................................................TGGATTGAAGGGAGCTCT.......... 33

...........................................................................................................................................................TGGATTGAAGGGAGCTCTA......... 5

...........................................................................................................................................................TGGATTGAAGGGAGCTCT.......... 18

...........................................................................................................................................................TGGATTGAAGGGAGCTCTA......... 4

............................................................................................................................................................GGATTGAAGGGAGCTCTA......... 8

............................................................................................................................................................GGATTGAAGGGAGCTCTA......... 14

>ptc-MIR319c_MI0002298_Populus_trichocarpa_miR319c_stem-loop

AAUGGUUUAAGAGAGCUUUCUUCAGUCCACUCAUGGACGGGCGAAGGGUUUGGAUUAGCUGCCGACUCAUUCAUUCAAACACAGUAGAAAUUAAGGGAGCAGUAUGGCUGCUAUUGUGAAUGUGUGAAUGAUGCGGGAGAUAAAUUUCAUCCUUUUCUUCUCUGUGCUUGGACUGAAGGGAGCUCCCUUUAAUCGU

.((((((.(((.(((((..((((((((((..((((((..((.(((((((..(((((..((.(((..((((((((.((..((((((............(((((.....)))))))))))..)).))))))))..))).))...))))).))))))).))..))))))..))))))))))..))))).))).)))))) (-82.80)

........................................................AGCTGCCGACTCATTCATTCA....................................................................................................................... 2

..................................................................................................................................................................TGTGCTTGGACTGAAGGGAGC............. 1

......................................................................................................................................................................CTTGGACTGAAGGGAGCT............ 1

.......................................................................................................................................................................TTGGACTGAAGGGAGCTCC.......... 1

.......................................................................................................................................................................TTGGACTGAAGGGAGCTCCC......... 6

.......................................................................................................................................................................TTGGACTGAAGGGAGCTCCCT........ 2

.......................................................................................................................................................................TTGGACTGAAGGGAGCTCCCT........ 1

........................................................................................................................................................................TGGACTGAAGGGAGCTCCC......... 1

........................................................................................................................................................................TGGACTGAAGGGAGCTCCC......... 2

>ptc-MIR319d_MI0002299_Populus_trichocarpa_miR319d_stem-loop

AAUGGUUUAACAGAGCUUCCUUCAGUCCACUCAUGGACGGGCGAAGGGUUUGGAUUAGCUGCCGACUCAUUCAUUCAAACACAGUAGACAAGGAGUGGCAGCGGCUGCUAUUGUGAAUGUGUGAAUGACGCGGGAGAUUAAUUUCAUCCUUUUCUUCUCUGUGCUUGGACUGAAGGGAGCUCCCUUUAAUUGU

.(..(((.((..(((((((((((((((((..((((((..((.(((((((..(((((((((.(((..((((((((.((..((((((((...((..((....))..)).))))))))..)).))))))))..))).)).))))))).))))))).))..))))))..)))))))))))))))))..)).)))..) (-84.50)

........................................................AGCTGCCGACTCATTCATTCA.................................................................................................................... 2

...............................................................................................................................................................TGTGCTTGGACTGAAGGGAGC............. 1

...................................................................................................................................................................CTTGGACTGAAGGGAGCT............ 1

....................................................................................................................................................................TTGGACTGAAGGGAGCTCC.......... 1

....................................................................................................................................................................TTGGACTGAAGGGAGCTCCC......... 6

....................................................................................................................................................................TTGGACTGAAGGGAGCTCCCT........ 2

....................................................................................................................................................................TTGGACTGAAGGGAGCTCCCT........ 1

.....................................................................................................................................................................TGGACTGAAGGGAGCTCCC......... 1

.....................................................................................................................................................................TGGACTGAAGGGAGCTCCC......... 2

>ptc-MIR319f_MI0002301_Populus_trichocarpa_miR319f_stem-loop

UAAUGGUGGGAGAGAGCUUCCUUCAGCCCACUCAUGGAUAGGAGAAAGGGGUUGAAUUAGCUGCCGACUCAUUCAUUCAAGCACCAGUAGAAAAAGGGGAAUGGAUAUUCUUUUGCUACUGUGAUUGUGUGAAUGAUGCGGGAGAUAAUUUUACAUCCCCUCUUUUUCUGUGCUUGGACUGAAGGGAGCUCCUUCCUUCUAU

..((((.(((((.(((((((((((((.(((..((((((.((((((..((((..((((((.((.(((..((((((((.(((.(((.(((((....((((((((....)))))))).)))))))).))).))))))))..))).)).)))))).....))))))))))))))))..))).))))))))))))))))))..)))) (-91.10)

..........................................................AGCTGCCGACTCATTCATTCA........................................................................................................................... 2

........................................................................................................................................................................TGTGCTTGGACTGAAGGGAGC............. 1

............................................................................................................................................................................CTTGGACTGAAGGGAGCTCCTT........ 15

............................................................................................................................................................................CTTGGACTGAAGGGAGCT............ 1

............................................................................................................................................................................CTTGGACTGAAGGGAGCTCCTT........ 6

.............................................................................................................................................................................TTGGACTGAAGGGAGCTCC.......... 1

.............................................................................................................................................................................TTGGACTGAAGGGAGCTCCT......... 1

.............................................................................................................................................................................TTGGACTGAAGGGAGCTCCTT........ 16

>ptc-MIR319g_MI0002302_Populus_trichocarpa_miR319g_stem-loop

UAAUCGUGGGAGAGAGCUUCUUUCAGCCCACUCGUGGAUAGGACAAAGGGGUUGAACUAGCUGCCGACUCAUUCAUUCAAGCACUAGUAGAAAAAAAGGCGAACGGUGUUUCUUUUGCUACUGUGAUUGUGUGAAUGAUGCGGGAGAUAAUUUUCCAUCCUCUCCUUUUCUGUGCUUGGACUGAAGGGAGCUCCUUCCUUCUCU

.......(((((.(((((((((((((.(((..((..((.((((....(((((.(((....((.(((..((((((((.(((.(((.(((((.((((.(((((.....))))).)))).)))))))).))).))))))))..))).))......))).))))).))))..))..))..))).))))))))))))))))))...... (-80.50)

..........................................................AGCTGCCGACTCATTCATTCA............................................................................................................................. 2

..........................................................................................................................................................................TGTGCTTGGACTGAAGGGAGC............. 1

..............................................................................................................................................................................CTTGGACTGAAGGGAGCTCCTT........ 15

..............................................................................................................................................................................CTTGGACTGAAGGGAGCT............ 1

..............................................................................................................................................................................CTTGGACTGAAGGGAGCTCCTT........ 6

...............................................................................................................................................................................TTGGACTGAAGGGAGCTCC.......... 1

...............................................................................................................................................................................TTGGACTGAAGGGAGCTCCT......... 1

...............................................................................................................................................................................TTGGACTGAAGGGAGCTCCTT........ 16

>ptc-MIR394a_MI0002313_Populus_trichocarpa_miR394a_stem-loop

UCAUGUGGGUUUUGCAAAGGGUUUCUUACAGAGUUUUUUGGCAUUCUGUCCACCUCCAUCUGUAGAAACUACAAGUUGUUCUACUUUCUGGAGGUGGGCAUACUGCCAACUGAGCUCUGUUGGUCUCUCUUUGUAAAACCCUCGUGA

(((((.(((((((((((((((...((.(((((((((.((((((.(.((((((((((((...((((((.(........)))))))....)))))))))))).).))))))..))))))))).))...))))))))))))))).))))) (-80.30)

.....................................TTGGCATTCTGTCCACCTCC.......................................................................................... 128

.....................................TTGGCATTCTGTCCACCTC........................................................................................... 1

.....................................TTGGCATTCTGTCCACCTCCA......................................................................................... 2

.....................................TTGGCATTCTGTCCACCTCC.......................................................................................... 7

.....................................TTGGCATTCTGTCCACCTCCA......................................................................................... 1

................................................................................................................AGCTCTGTTGGTCTCTCTTTG.............. 1

....................................................................................................................CTGTTGGTCTCTCTTTGTAAA.......... 1

>ptc-MIR394b_MI0002314_Populus_trichocarpa_miR394b_stem-loop

UCAUGUGGAUUUAGCAAAGGGUUUCUUACAGAGUUUAUUGGCAUUCUGUCCACCUCCUAUCUUUAGAAAUUAGAAUAUCUCUUUCAUAUGGAGGUGGGCAUACUGCCAACCGAGCUCUGUUGGUCUCUCUUUGUAAAACCCUCGUGA

(((((.((.(((.((((((((...((.(((((((((.((((((.(.(((((((((((.......(((.((......)).))).......))))))))))).).))))))..))))))))).))...)))))))).))).)).))))) (-63.74)

.....................................TTGGCATTCTGTCCACCTCC.......................................................................................... 128

.....................................TTGGCATTCTGTCCACCTC........................................................................................... 1

.....................................TTGGCATTCTGTCCACCTCC.......................................................................................... 7

................................................................................................................AGCTCTGTTGGTCTCTCTTTG.............. 1

....................................................................................................................CTGTTGGTCTCTCTTTGTAAA.......... 1

>ptc-MIR482_MI0002397_Populus_trichocarpa_miR482_stem-loop

GAGUCCUAGCAAGUCUUUGGAGAUGGGAGAGUAUGCAAGAAGGAAAAAUUCAUGAUUUAAUAUUCUUUCUUGCCUACUCCUCCCAUUCCAUCUGCUUUCUGCGACUC

(((((.(((.((((...(((((.((((((((((.((((((((((((((((...)))))....))))))))))).)))).)))))))))))...)))).))).))))) (-46.30)

GAGTCCTAGCAAGTCTTTGGA...................................................................................... 2

GAGTCCTAGCAAGTCTTTGG....................................................................................... 20

...............TTTGGAGATGGGAGAGTATGCA...................................................................... 1

....................AGATGGGAGAGTATGCAAGA................................................................... 2

....................AGATGGGAGAGTATGCAAGAAG................................................................. 1

....................AGATGGGAGAGTATGCAAG.................................................................... 2

....................AGATGGGAGAGTATGCAAGAAG................................................................. 2

....................AGATGGGAGAGTATGCAAGAA.................................................................. 1

....................AGATGGGAGAGTATGCAAGAAG................................................................. 3

....................AGATGGGAGAGTATGCAAGAA.................................................................. 1

.....................GATGGGAGAGTATGCAAG.................................................................... 1

.................................................................TTTCTTGCCTACTCCTCCCATTCC.................. 3

..................................................................TTCTTGCCTACTCCTCCCATTC................... 5

..................................................................TTCTTGCCTACTCCTCCCATTC................... 1

...................................................................TCTTGCCTACTCCTCCCATTCC.................. 57

...................................................................TCTTGCCTACTCCTCCCAT..................... 3

...................................................................TCTTGCCTACTCCTCCCATTC................... 2

...................................................................TCTTGCCTACTCCTCCCATT.................... 1

...................................................................TCTTGCCTACTCCTCCCATTCC.................. 10

...................................................................TCTTGCCTACTCCTCCCATTCC.................. 7

....................................................................CTTGCCTACTCCTCCCATTCCA................. 1

.....................................................................TTGCCTACTCCTCCCATTCCA................. 3

.....................................................................TTGCCTACTCCTCCCATTCC.................. 1

.........................................................................CTACTCCTCCCATTCCATCTGC............ 4

..........................................................................TACTCCTCCCATTCCATCTGCT........... 1

........................................................................................CATCTGCTTTCTGCGACT. 1

>ptc-MIR1450_MI0007050_Populus_trichocarpa_miR1450_stem-loop

GAGUCAAUUAGGUCAGUUGUGUAGUCUGACCCGAGCCAUUGAAGACAAUCAUUUGUUGGUGUCUCAUUGGAUUUGUUACCUGACAGUGUUUAUCCAACAAAUGAUUCUCUUCAAUGGCUCGGUCAGGUUACACAAGCAAUCUAGUUGGCUC

(((((((((((((...(((((((..((((((.(((((((((((((.(((((((((((((.....(((((..............))))).....))))))))))))).)))))))))))))))))))..)))))))...))))))))))))) (-84.14)

.AGTCAATTAGGTCAGTTGTGTA................................................................................................................................ 2

.AGTCAATTAGGTCAGTTGTGT................................................................................................................................. 1

..GTCAATTAGGTCAGTTGTGT................................................................................................................................. 8

..GTCAATTAGGTCAGTTGTGTA................................................................................................................................ 26

...TCAATTAGGTCAGTTGTGTAG............................................................................................................................... 2

........TAGGTCAGTTGTGTAGTCTGA.......................................................................................................................... 1

............TCAGTTGTGTAGTCTGACCCG...................................................................................................................... 1

............TCAGTTGTGTAGTCTGACCCGA..................................................................................................................... 4

..............AGTTGTGTAGTCTGACCCGA..................................................................................................................... 1

................TTGTGTAGTCTGACCCGAGCCA................................................................................................................. 1

................TTGTGTAGTCTGACCCGAGCC.................................................................................................................. 2

.................TGTGTAGTCTGACCCGAGCCA................................................................................................................. 2

...................TGTAGTCTGACCCGAGCCATTGA............................................................................................................. 1

....................GTAGTCTGACCCGAGCCATTGA............................................................................................................. 1

......................AGTCTGACCCGAGCCATTGAAGA.......................................................................................................... 7

.......................GTCTGACCCGAGCCATTGAAGA.......................................................................................................... 17

.........................CTGACCCGAGCCATTGAAGA.......................................................................................................... 3

..........................TGACCCGAGCCATTGAAGA.......................................................................................................... 1

................................................................................................ACAAATGATTCTCTTCAATGG.................................. 1

...................................................................................................AATGATTCTCTTCAATGGCTC............................... 255

...................................................................................................AATGATTCTCTTCAATGGCTCGGT............................ 2

...................................................................................................AATGATTCTCTTCAATGGCT................................ 1

....................................................................................................ATGATTCTCTTCAATGGCTC............................... 2

.....................................................................................................TGATTCTCTTCAATGGCTCGGTC........................... 1

.....................................................................................................TGATTCTCTTCAATGGCTCGG............................. 1

.....................................................................................................TGATTCTCTTCAATGGCTC............................... 1

..........................................................................................................CTCTTCAATGGCTCGGTCAGG........................ 3

............................................................................................................CTTCAATGGCTCGGTCAGGTTA..................... 1

.............................................................................................................TTCAATGGCTCGGTCAGG........................ 1

.............................................................................................................TTCAATGGCTCGGTCAGGTT...................... 169

.............................................................................................................TTCAATGGCTCGGTCAGGTTACAC.................. 2

.............................................................................................................TTCAATGGCTCGGTCAGGTTAC.................... 42

.............................................................................................................TTCAATGGCTCGGTCAGGTTA..................... 1819

.............................................................................................................TTCAATGGCTCGGTCAGGT....................... 5

.............................................................................................................TTCAATGGCTCGGTCAGGTTA..................... 154

.............................................................................................................TTCAATGGCTCGGTCAGGTT...................... 3

.............................................................................................................TTCAATGGCTCGGTCAGG........................ 2

.............................................................................................................TTCAATGGCTCGGTCAGGTTA..................... 77

.............................................................................................................TTCAATGGCTCGGTCAGGTT...................... 3

.............................................................................................................TTCAATGGCTCGGTCAGGT....................... 1

..............................................................................................................TCAATGGCTCGGTCAGGTTA..................... 6

..............................................................................................................TCAATGGCTCGGTCAGGTTAC.................... 32

..............................................................................................................TCAATGGCTCGGTCAGGT....................... 1

..............................................................................................................TCAATGGCTCGGTCAGGTTA..................... 7

..............................................................................................................TCAATGGCTCGGTCAGGTTA..................... 3

...............................................................................................................CAATGGCTCGGTCAGGTTA..................... 3

...............................................................................................................CAATGGCTCGGTCAGGTTA..................... 7

................................................................................................................AATGGCTCGGTCAGGTTA..................... 8

................................................................................................................AATGGCTCGGTCAGGTTAC.................... 1

................................................................................................................AATGGCTCGGTCAGGTTA..................... 1

..................................................................................................................TGGCTCGGTCAGGTTACACA................. 1

....................................................................................................................GCTCGGTCAGGTTACACAAGC.............. 1

..................................................................................................................................CACAAGCAATCTAGTTGGCTC 5

..................................................................................................................................CACAAGCAATCTAGTTGGCTC 2

.....................................................................................................................................AAGCAATCTAGTTGGCTC 2

.....................................................................................................................................AAGCAATCTAGTTGGCTC 2
