# Supplementary material for: Multiple distinct small RNAs originate from the same microRNA precursors
Source: Genome Biol. 2010 Aug 9;11(8):R81. doi: 10.1186/gb-2010-11-8-r81 (PMC2945783; doi:10.1186/gb-2010-11-8-r81)
Supplement: Additional file 7 — Supplemental File S6. This is a file for sequencing reads mapped and aligned to miRNA precursors that can produce miRNA-sibling small RNAs (msRNAs) in H. sapiens (has). The sequencing data were obtained from GEO; see Materials and methods for details. [file gb-2010-11-8-r81-S7.DOCX]

Zhang, et al., Multiple distinct small RNAs originate from the same microRNA precursors

Supplemental File 6 - Sequencing reads mapped and aligned to miRNA precursors that can produce

miRNA-like RNAs in *Homo sapiens.*

>hsa-mir-15b_MI0000438_Homo_sapiens_miR-15b_stem-loop lekumia_bm

UUGAGGCCUUAAAGUACUGUAGCAGCACAUCAUGGUUUACAUGCUACAGUCAAGAUGCGAAUCAUUAUUUGCUGCUCUAGAAAUUUAAGGAAAUUCAU

.((((.(((((((...(((.(((((((.((.((((((..(((.((.......)))))..)))))).)).))))))).)))...)))))))...)))). (-32.70)

TTGAGGCCTTAAAGTACTG............................................................................... 4

.TGAGGCCTTAAAGTACTG............................................................................... 2

...................TAGCAGCACATCATGGTTTA........................................................... 1042

...................TAGCAGCACATCATGGTTTACA......................................................... 692

...................TAGCAGCACATCATGGTTT............................................................ 368

...................TAGCAGCACATCATGGTTTAC.......................................................... 200

...................TAGCAGCACATCATGGTT............................................................. 100

...................TAGCAGCACATCATGGTTTACAT........................................................ 6

....................AGCAGCACATCATGGTTTACA......................................................... 1

....................AGCAGCACATCATGGTTTA........................................................... 1

.....................GCAGCACATCATGGTTTACA......................................................... 1

.....................GCAGCACATCATGGTTTA........................................................... 1

........................................................GCGAATCATTATTTGCTGCTCT.................... 2

.........................................................CGAATCATTATTTGCTGCTCTA................... 57

.........................................................CGAATCATTATTTGCTGCTCT.................... 56

.........................................................CGAATCATTATTTGCTGCT...................... 37

.........................................................CGAATCATTATTTGCTGCTC..................... 8

.........................................................CGAATCATTATTTGCTGC....................... 2

..........................................................GAATCATTATTTGCTGCTCTA................... 1

>hsa-mir-331_MI0000812_Homo_sapiens_miR-331_stem-loop lekumia_bm

GAGUUUGGUUUUGUUUGGGUUUGUUCUAGGUAUGGUCCCAGGGAUCCCAGAUCAAACCAGGCCCCUGGGCCUAUCCUAGAACCAACCUAAGCUC

............(((((((((.((((((((.((((.(((((((..(.............)..))))))).)))))))))))).))))))))).. (-43.52)

...TTTGGTTTTGTTTGGGTTTGTT..................................................................... 1

.....TGGTTTTGTTTGGGTTTGTT..................................................................... 16

......GGTTTTGTTTGGGTTTGTT..................................................................... 23

.......GTTTTGTTTGGGTTTGTT..................................................................... 18

.......................TTCTAGGTATGGTCCCAGGGAT................................................. 1

.........................CTAGGTATGGTCCCAGGGAT................................................. 4

............................................................GCCCCTGGGCCTATCCTAGAA............. 22

............................................................GCCCCTGGGCCTATCCTAGA.............. 8

>hsa-mir-1977_MI0009987_Homo_sapiens_miR-1977_stem-loop lekumia_bm

UUGAUUAGGGUGCUUAGCUGUUAACUAAGUGUUUGUGGGUUUAAGUCCCAUUGGUCUAGUAAGGGCUUAGCUUAAUUAA

.(((((((...(((.((((.(((.(((.(.....(((((.......)))))....))))))).)))).)))))))))). (-20.50)

TTGATTAGGGTGCTTAGCTGTTAA....................................................... 1

.....TAGGGTGCTTAGCTGTTAA....................................................... 2

......................AACTAAGTGTTTGTGGGTTTA.................................... 1

............................................................AAGGGCTTAGCTTAATTA. 3

>hsa-mir-7-1_MI0000263_Homo_sapiens_miR-7-1_stem-loop lekumia_Acute lymphoblastic leukemia patient

UUGGAUGUUGGCCUAGUUCUGUGUGGAAGACUAGUGAUUUUGUUGUUUUUAGAUAACUAAAUCGACAACAAAUCACAGUCUGCCAUAUGGCACAGGCCAUGCCUCUACAG

.((((.((((((((.((.((((((((.(((((.(((((((.(((((((((((....)))))..)))))))))))))))))).)))))))).)))))))).)).))))... (-48.00)

TTGGATGTTGGCCTAGTTCT.......................................................................................... 1

.......................TGGAAGACTAGTGATTTTGTTGT................................................................ 611

.......................TGGAAGACTAGTGATTTTGTTGTTT.............................................................. 35

........................GGAAGACTAGTGATTTTGTTGTT............................................................... 3

........................GGAAGACTAGTGATTTTGTTGT................................................................ 1

.................................................................CAACAAATCACAGTCTGCCATA....................... 57

.................................................................CAACAAATCACAGTCTGCCAT........................ 14

.................................................................CAACAAATCACAGTCTGCCA......................... 10

.................................................................CAACAAATCACAGTCTGCC.......................... 3

.................................................................CAACAAATCACAGTCTGCCATAT...................... 1

..................................................................AACAAATCACAGTCTGCCATA....................... 14

..................................................................AACAAATCACAGTCTGCCAT........................ 2

..................................................................AACAAATCACAGTCTGCCATAT...................... 2

..................................................................AACAAATCACAGTCTGCCA......................... 1

...................................................................ACAAATCACAGTCTGCCATAT...................... 1

...................................................................ACAAATCACAGTCTGCCATA....................... 1

...................................................................ACAAATCACAGTCTGCCAT........................ 1

>hsa-mir-181a-2_MI0000269_Homo_sapiens_mir-181a-2_stem-loop lekumia_Acute lymphoblastic leukemia patient

AGAAGGGCUAUCAGGCCAGCCUUCAGAGGACUCCAAGGAACAUUCAACGCUGUCGGUGAGUUUGGGAUUUGAAAAAACCACUGACCGUUGACUGUACCUUGGGGUCCUUA

.((((((((....)))...))))).(((((((((((((.(((.((((((..(((((((.((((...........))))))))))))))))).))).))))))))))))). (-54.00)

..................GCCTTCAGAGGACTCCAAGG........................................................................ 1

...................CCTTCAGAGGACTCCAAGG........................................................................ 1

.....................................GAACATTCAACGCTGTCGGTGAGT................................................. 1

......................................AACATTCAACGCTGTCGGTGAG.................................................. 4337

......................................AACATTCAACGCTGTCGGTGAGTT................................................ 3499

......................................AACATTCAACGCTGTCGGTGAGT................................................. 2856

......................................AACATTCAACGCTGTCGGTGAGTTT............................................... 922

......................................AACATTCAACGCTGTCGGTGA................................................... 454

......................................AACATTCAACGCTGTCGGTG.................................................... 197

......................................AACATTCAACGCTGTCGGT..................................................... 41

......................................AACATTCAACGCTGTCGG...................................................... 2

.......................................ACATTCAACGCTGTCGGTGAGTT................................................ 7

.......................................ACATTCAACGCTGTCGGTGAG.................................................. 3

.......................................ACATTCAACGCTGTCGGTGA................................................... 1

.......................................ACATTCAACGCTGTCGGTGAGTTT............................................... 1

.......................................ACATTCAACGCTGTCGGTGAGT................................................. 1

........................................CATTCAACGCTGTCGGTGAG.................................................. 1

.............................................AACGCTGTCGGTGAGTTT............................................... 1

...........................................................................AACCACTGACCGTTGACTG................ 1

...........................................................................AACCACTGACCGTTGACTGTAC............. 1

............................................................................ACCACTGACCGTTGACTGT............... 95

............................................................................ACCACTGACCGTTGACTGTA.............. 37

............................................................................ACCACTGACCGTTGACTGTAC............. 26

............................................................................ACCACTGACCGTTGACTG................ 24

............................................................................ACCACTGACCGTTGACTGTACC............ 11

............................................................................ACCACTGACCGTTGACTGTACCT........... 1

>hsa-mir-193a_MI0000487_Homo_sapiens_miR-193a_stem-loop lekumia_Acute lymphoblastic leukemia patient

CGAGGAUGGGAGCUGAGGGCUGGGUCUUUGCGGGCGAGAUGAGGGUGUCGGAUCAACUGGCCUACAAAGUCCCAGUUCUCGGCCCCCG

.......(((.(((((((((((((.(((((..(((.((.(((..........))).)).)))..))))).))))))))))))).))). (-50.80)

CGAGGATGGGAGCTGAGGGC.................................................................... 1

....................TGGGTCTTTGCGGGCGAGATGA.............................................. 50

....................TGGGTCTTTGCGGGCGAGATG............................................... 9

....................TGGGTCTTTGCGGGCGAG.................................................. 3

....................TGGGTCTTTGCGGGCGAGAT................................................ 1

......................................................AACTGGCCTACAAAGTCCCA.............. 107

......................................................AACTGGCCTACAAAGTCCCAG............. 42

......................................................AACTGGCCTACAAAGTCCCAGT............ 34

......................................................AACTGGCCTACAAAGTCCC............... 2

>hsa-mir-331_MI0000812_Homo_sapiens_miR-331_stem-loop lekumia_Acute lymphoblastic leukemia patient

GAGUUUGGUUUUGUUUGGGUUUGUUCUAGGUAUGGUCCCAGGGAUCCCAGAUCAAACCAGGCCCCUGGGCCUAUCCUAGAACCAACCUAAGCUC

............(((((((((.((((((((.((((.(((((((..(.............)..))))))).)))))))))))).))))))))).. (-43.52)

..GTTTGGTTTTGTTTGGGTTTGTT..................................................................... 1

....TTGGTTTTGTTTGGGTTTGTT..................................................................... 5

.....TGGTTTTGTTTGGGTTTGTT..................................................................... 29

.....TGGTTTTGTTTGGGTTTGT...................................................................... 3

......GGTTTTGTTTGGGTTTGTT..................................................................... 38

......GGTTTTGTTTGGGTTTGT...................................................................... 3

.......GTTTTGTTTGGGTTTGTT..................................................................... 19

.......................TTCTAGGTATGGTCCCAGGGAT................................................. 4

.........................CTAGGTATGGTCCCAGGGAT................................................. 1

............................................................GCCCCTGGGCCTATCCTAGAA............. 9

............................................................GCCCCTGGGCCTATCCTAGA.............. 2

.............................................................CCCCTGGGCCTATCCTAGAA............. 1

>hsa-mir-34a_MI0000268_Homo_sapiens_miR-34a_stem-loop undifferentiated hESC

GGCCAGCUGUGAGUGUUUCUUUGGCAGUGUCUUAGCUGGUUGUUGUGAGCAAUAGUAAGGAAGCAAUCAGCAAGUAUACUGCCCUAGAAGUGCUGCACGUUGUGGGGCCC

((((.((((((((..(((((..(((((((((((.((((((((((....((....)).....))))))))))))).))))))))..)))))..)).))))..))..)))). (-50.70)

.GCCAGCTGTGAGTGTTTCTTT........................................................................................ 1

....................TTGGCAGTGTCTTAGCTGGT...................................................................... 1

....................TTGGCAGTGTCTTAGCTGGTTG.................................................................... 1

.....................TGGCAGTGTCTTAGCTGGTT..................................................................... 3

.....................TGGCAGTGTCTTAGCTGGTTGTTG................................................................. 1

.....................TGGCAGTGTCTTAGCTGGTTGTT.................................................................. 44

.....................TGGCAGTGTCTTAGCTGGTTG.................................................................... 3

.....................TGGCAGTGTCTTAGCTGG....................................................................... 1

.....................TGGCAGTGTCTTAGCTGGTTGT................................................................... 15

......................GGCAGTGTCTTAGCTGGTTGT................................................................... 1

......................GGCAGTGTCTTAGCTGGTTGTT.................................................................. 1

..........................GTGTCTTAGCTGGTTGTTG................................................................. 2

...........................................TGTGAGCAATAGTAAGGA................................................. 1

...............................................................CAATCAGCAAGTATACTGCCCT......................... 1

...............................................................CAATCAGCAAGTATACTGCCCTA........................ 3

................................................................AATCAGCAAGTATACTGCCCTA........................ 9

................................................................AATCAGCAAGTATACTGCCCT......................... 1

................................................................AATCAGCAAGTATACTGCC........................... 1

................................................................AATCAGCAAGTATACTGCCC.......................... 1

>hsa-mir-181a-2_MI0000269_Homo_sapiens_mir-181a-2_stem-loop undifferentiated hESC

AGAAGGGCUAUCAGGCCAGCCUUCAGAGGACUCCAAGGAACAUUCAACGCUGUCGGUGAGUUUGGGAUUUGAAAAAACCACUGACCGUUGACUGUACCUUGGGGUCCUUA

.((((((((....)))...))))).(((((((((((((.(((.((((((..(((((((.((((...........))))))))))))))))).))).))))))))))))). (-54.00)

..................GCCTTCAGAGGACTCCAAGG........................................................................ 2

...................CCTTCAGAGGACTCCAAGG........................................................................ 3

.....................................GAACATTCAACGCTGTCGGTGA................................................... 1

.....................................GAACATTCAACGCTGTCGGTGAG.................................................. 1

.....................................GAACATTCAACGCTGTCGGTG.................................................... 2

......................................AACATTCAACGCTGTCGGT..................................................... 141

......................................AACATTCAACGCTGTCGG...................................................... 250

......................................AACATTCAACGCTGTCGGTGAGTT................................................ 110

......................................AACATTCAACGCTGTCG....................................................... 79

......................................AACATTCAACGCTGTCGGTGA................................................... 20

......................................AACATTCAACGCTGTCGGTGAG.................................................. 81

......................................AACATTCAACGCTGTCGGTG.................................................... 311

......................................AACATTCAACGCTGTCGGTGAGT................................................. 58

......................................AACATTCAACGCTGTCGGTGAGTTTG.............................................. 5

......................................AACATTCAACGCTG.......................................................... 3

......................................AACATTCAACGCTGTCGGTGAGTTT............................................... 571

.......................................ACATTCAACGCTGTCGGTGAGTT................................................ 1

.......................................ACATTCAACGCTGTCGGTGAG.................................................. 1

.......................................ACATTCAACGCTGTCGGT..................................................... 1

.......................................ACATTCAACGCTGTCGGTGAGTTT............................................... 3

.........................................ATTCAACGCTGTCGGTGAGTTT............................................... 2

.........................................ATTCAACGCTGTCGGTGAGT................................................. 1

............................................CAACGCTGTCGGTGAGTTT............................................... 1

..........................................................................AAACCACTGACCGTTGACTGTA.............. 1

...........................................................................AACCACTGACCGTTGACTGTAC............. 5

...........................................................................AACCACTGACCGTTGACTGTA.............. 1

............................................................................ACCACTGACCGTTGACTGTA.............. 5

............................................................................ACCACTGACCGTTGACTG................ 2

............................................................................ACCACTGACCGTTGACTGTACC............ 34

............................................................................ACCACTGACCGTTGACTGT............... 4

............................................................................ACCACTGACCGTTGACTGTACCT........... 10

............................................................................ACCACTGACCGTTGACTGTAC............. 22

.............................................................................CCACTGACCGTTGACTGTACC............ 1

>hsa-mir-187_MI0000274_Homo_sapiens_miR-187_stem-loop undifferentiated hESC

GGUCGGGCUCACCAUGACACAGUGUGAGACCUCGGGCUACAACACAGGACCCGGGCGCUGCUCUGACCCCUCGUGUCUUGUGUUGCAGCCGGAGGGACGCAGGUCCGCA

.(.((((((.............((((...((((.((((.(((((((((((.((((.((......).).)).)).))))))))))).)))).))))..))))))))))). (-47.91)

..............TGACACAGTGTGAGACCTCG........................................................................... 1

..............TGACACAGTGTGAGACC.............................................................................. 1

..................................GGCTACAACACAGGACCC......................................................... 1

..................................GGCTACAACACAGGACCCGGGC..................................................... 3

...................................GCTACAACACAGGACCCGGGC..................................................... 2

...................................GCTACAACACAGGACCCGGG...................................................... 5

...................................GCTACAACACAGGACCCGGGCG.................................................... 18

...................................GCTACAACACAGGACCCG........................................................ 1

.....................................................................CTCGTGTCTTGTGTTGCAGC.................... 1

.....................................................................CTCGTGTCTTGTGTTGCAGCCG.................. 5

.....................................................................CTCGTGTCTTGTGTTGCAG..................... 1

.....................................................................CTCGTGTCTTGTGTTGCAGCCGG................. 5

.....................................................................CTCGTGTCTTGTGTTGCA...................... 1

......................................................................TCGTGTCTTGTGTTGCAGCCGGA................ 21

......................................................................TCGTGTCTTGTGTTGCAGCC................... 20

......................................................................TCGTGTCTTGTGTTGCAG..................... 25

......................................................................TCGTGTCTTGTGTTGCAGCCGG................. 281

......................................................................TCGTGTCTTGTGTTGCA...................... 11

......................................................................TCGTGTCTTGTGTTGCAGC.................... 12

......................................................................TCGTGTCTTGTGTTGCAGCCGGAG............... 1

......................................................................TCGTGTCTTGTGTTGCAGCCG.................. 44

.......................................................................CGTGTCTTGTGTTGCAGCCGG................. 24

.......................................................................CGTGTCTTGTGTTGCAG..................... 1

........................................................................GTGTCTTGTGTTGCAGCCGG................. 3

>hsa-mir-204_MI0000284_Homo_sapiens_miR-204_stem-loop undifferentiated hESC

GGCUACAGUCUUUCUUCAUGUGACUCGUGGACUUCCCUUUGUCAUCCUAUGCCUGAGAAUAUAUGAAGGAGGCUGGGAAGGCAAAGGGACGUUCAAUUGUCAUCACUGGC

(((....))).....(((((((((...(((((.((((((((((.(((((.((((...............))))))))).)))))))))).)))))...)))).)).))). (-42.26)

............TCTTCATGTGACTCGTGGAC.............................................................................. 8

............TCTTCATGTGACTCGTGGACT............................................................................. 2

.............CTTCATGTGACTCGTGGAC.............................................................................. 3

.............CTTCATGTGACTCGTGG................................................................................ 2

.............CTTCATGTGACTCGTGGACT............................................................................. 1

..............TTCATGTGACTCGTGGA............................................................................... 1

..............TTCATGTGACTCGTGGAC.............................................................................. 1

...............TCATGTGACTCGTGGAC.............................................................................. 2

..............................ACTTCCCTTTGTCATCCTATGCCTG....................................................... 1

................................TTCCCTTTGTCATCCTATGCC......................................................... 25

................................TTCCCTTTGTCATCCTATGCCTG....................................................... 35

................................TTCCCTTTGTCATCCTATGCCT........................................................ 690

................................TTCCCTTTGTCATCCTATGC.......................................................... 7

................................TTCCCTTTGTCATCCTATGCCTGA...................................................... 1

................................TTCCCTTTGTCATCCTAT............................................................ 32

................................TTCCCTTTGTCATCCTA............................................................. 2

................................TTCCCTTTGTCATCCTATG........................................................... 16

.................................TCCCTTTGTCATCCTATG........................................................... 1

.................................TCCCTTTGTCATCCTATGCC......................................................... 1

.................................TCCCTTTGTCATCCTATGCCT........................................................ 7

......................................................................GGCTGGGAAGGCAAAGGG...................... 1

.......................................................................GCTGGGAAGGCAAAGGGACG................... 2

.......................................................................GCTGGGAAGGCAAAGGGACGTTC................ 1

.......................................................................GCTGGGAAGGCAAAGGG...................... 1

.......................................................................GCTGGGAAGGCAAAGGGACGT.................. 6

>hsa-mir-221_MI0000298_Homo_sapiens_miR-221_stem-loop undifferentiated hESC

UGAACAUCCAGGUCUGGGGCAUGAACCUGGCAUACAAUGUAGAUUUCUGUGUUCGUUAGGCAACAGCUACAUUGUCUGCUGGGUUUCAGGCUACCUGGAAACAUGUUCUC

.((((((((((((....(((.((((((..(((.(((((((((....(((((((.....))).))))))))))))).)))..)).)))).))))))))))....))))).. (-47.20)

TGAACATCCAGGTCTGGGGCATGA...................................................................................... 3

.....ATCCAGGTCTGGGGCATGA...................................................................................... 1

.......................AACCTGGCATACAATGTAGA................................................................... 2

.......................AACCTGGCATACAATGTAGATTTC............................................................... 34

.......................AACCTGGCATACAATGTAGATT................................................................. 3

.......................AACCTGGCATACAATGTAGATTTCT.............................................................. 28

.......................AACCTGGCATACAATGTAG.................................................................... 10

.......................AACCTGGCATACAATGT...................................................................... 3

.......................AACCTGGCATACAATGTAGAT.................................................................. 2

.......................AACCTGGCATACAATGTAGATTT................................................................ 56

........................ACCTGGCATACAATGTAGATTTCTG............................................................. 29

........................ACCTGGCATACAATGTAG.................................................................... 22

........................ACCTGGCATACAATGTAGATTTC............................................................... 357

........................ACCTGGCATACAATGTAGATTTCT.............................................................. 637

........................ACCTGGCATACAATGTAGATTTCTGT............................................................ 65

........................ACCTGGCATACAATGTAGA................................................................... 4

........................ACCTGGCATACAATGTA..................................................................... 5

........................ACCTGGCATACAATGTAGAT.................................................................. 5

........................ACCTGGCATACAATGTAGATT................................................................. 9

........................ACCTGGCATACAATGTAGATTT................................................................ 294

.........................CCTGGCATACAATGTAGATTTCTGT............................................................ 1

.........................CCTGGCATACAATGTAGATTT................................................................ 2

.........................CCTGGCATACAATGTAGATTTCT.............................................................. 6

.........................CCTGGCATACAATGTAGATTTC............................................................... 2

..........................CTGGCATACAATGTAGATTTCTGT............................................................ 1

..........................CTGGCATACAATGTAGATTTCT.............................................................. 2

...........................TGGCATACAATGTAGATTTCTGT............................................................ 1

...............................................................CAGCTACATTGTCTGCTGGGTTTC....................... 5

...............................................................CAGCTACATTGTCTGCTGGG........................... 1

................................................................AGCTACATTGTCTGCT.............................. 9

................................................................AGCTACATTGTCTGC............................... 22

................................................................AGCTACATTGTCTG................................ 2

................................................................AGCTACATTGTCTGCTGGGTTT........................ 11226

................................................................AGCTACATTGTCTGCTGGGTT......................... 634

................................................................AGCTACATTGTCTGCTGGGTTTCA...................... 790

................................................................AGCTACATTGTCTGCTGGGTTTCAG..................... 3

................................................................AGCTACATTGTCTGCTGGGT.......................... 214

................................................................AGCTACATTGTCTGCTGGG........................... 920

................................................................AGCTACATTGTCTGCTG............................. 277

................................................................AGCTACATTGTCTGCTGGGTTTC....................... 16275

................................................................AGCTACATTGTCTGCTGG............................ 475

.................................................................GCTACATTGTCTGCT.............................. 2

.................................................................GCTACATTGTCTGCTGGGTT......................... 21

.................................................................GCTACATTGTCTGCTGGGTTT........................ 410

.................................................................GCTACATTGTCTGCTGGGTTTCA...................... 18

.................................................................GCTACATTGTCTGCTGGGTTTC....................... 521

.................................................................GCTACATTGTCTGCTGG............................ 15

.................................................................GCTACATTGTCTGCTGGG........................... 50

.................................................................GCTACATTGTCTGCTGGGT.......................... 7

..................................................................CTACATTGTCTGCTGGGTTT........................ 1

..................................................................CTACATTGTCTGCTGGGTTTCA...................... 1

..................................................................CTACATTGTCTGCTGGGTTTC....................... 2

...................................................................TACATTGTCTGCTGGGTTT........................ 3

....................................................................ACATTGTCTGCTGGGTTT........................ 1

....................................................................ACATTGTCTGCTGGGTTTCA...................... 1

.....................................................................CATTGTCTGCTGGGTTTC....................... 1

.......................................................................................AGGCTACCTGGAAACAT...... 1

>hsa-mir-331_MI0000812_Homo_sapiens_miR-331_stem-loop undifferentiated hESC

GAGUUUGGUUUUGUUUGGGUUUGUUCUAGGUAUGGUCCCAGGGAUCCCAGAUCAAACCAGGCCCCUGGGCCUAUCCUAGAACCAACCUAAGCUC

............(((((((((.((((((((.((((.(((((((..(.............)..))))))).)))))))))))).))))))))).. (-43.52)

.....TGGTTTTGTTTGGGTTTGTT..................................................................... 14

......GGTTTTGTTTGGGTTTGTT..................................................................... 16

.......GTTTTGTTTGGGTTTGTT..................................................................... 5

.......................TTCTAGGTATGGTCCCAGGGAT................................................. 2

........................TCTAGGTATGGTCCCAGGGAT................................................. 1

.........................CTAGGTATGGTCCCAGGGATCC............................................... 3

.........................CTAGGTATGGTCCCAGGGATC................................................ 2

.........................CTAGGTATGGTCCCAGGGATCCC.............................................. 1

............................................................GCCCCTGGGCCTATCCT................. 129

............................................................GCCCCTGGGCCTATCCTAGAA............. 1129

............................................................GCCCCTGGGCCTATCCTAGAACC........... 2

............................................................GCCCCTGGGCCTATCCTAGAAC............ 15

............................................................GCCCCTGGGCCTATCC.................. 11

............................................................GCCCCTGGGCCTATCCTAG............... 179

............................................................GCCCCTGGGCCTATCCTA................ 76

............................................................GCCCCTGGGCCTATCCTAGA.............. 369

.............................................................CCCCTGGGCCTATCCTAGAAC............ 1

.............................................................CCCCTGGGCCTATCCTAG............... 2

.............................................................CCCCTGGGCCTATCCTAGAA............. 5

.............................................................CCCCTGGGCCTATCCTAGA.............. 3

................................................................CTGGGCCTATCCTAGAA............. 1

>hsa-mir-498_MI0003142_Homo_sapiens_miR-498_stem-loop undifferentiated hESC

AACCCUCCUUGGGAAGUGAAGCUCAGGCUGUGAUUUCAAGCCAGGGGGCGUUUUUCUAUAACUGGAUGAAAAGCACCUCCAGAGCUUGAAGCUCACAGUUUGAGAGCAAUCGUCUAAGGAAGUU

.....((((((((...((...(((((((((((((((((((((.(((((.(((((((...........))))))).))))).).)))))))).))))))))))))..))....)))))))).... (-57.50)

............GAAGTGAAGCTCAGGCTGTGA........................................................................................... 1

.............AAGTGAAGCTCAGGCTGTGA........................................................................................... 1

.................................TTTCAAGCCAGGGGGCGTTTTTCT................................................................... 27

.................................TTTCAAGCCAGGGGGCGTTT....................................................................... 4

.................................TTTCAAGCCAGGGGGCGTTTT...................................................................... 10

.................................TTTCAAGCCAGGGGGCGTTTTT..................................................................... 3

.................................TTTCAAGCCAGGGGGCGTTTTTCTA.................................................................. 1

.................................TTTCAAGCCAGGGGGCGT......................................................................... 1

.................................TTTCAAGCCAGGGGGCGTTTTTC.................................................................... 65

..................................TTCAAGCCAGGGGGCGTTTTTCT................................................................... 1

...................................TCAAGCCAGGGGGCGTTTT...................................................................... 1

.....................................................................AAAGCACCTCCAGAGCTTGAAGC................................ 1

.....................................................................AAAGCACCTCCAGAGCTTGAAGCT............................... 3

.....................................................................AAAGCACCTCCAGAGCTTGAAGCTC.............................. 4

.....................................................................AAAGCACCTCCAGAGCTTGA................................... 1

.....................................................................AAAGCACCTCCAGAGCTTGAAGCTCA............................. 1

.......................................................................................................GAGCAATCGTCTAAGGA.... 1

>hsa-mir-518c_MI0003159_Homo_sapiens_miR-518c_stem-loop undifferentiated hESC

GCGAGAAGAUCUCAUGCUGUGACUCUCUGGAGGGAAGCACUUUCUGUUGUCUGAAAGAAAACAAAGCGCUUCUCUUUAGAGUGUUACGGUUUGAGAAAAGC

.........(((((.((((((((.((((((((((((((.(((..((((.((.....)).))))))).)))))))))))))).)))))))).)))))..... (-46.40)

...AGAAGATCTCATGCTGTGAC.............................................................................. 1

...AGAAGATCTCATGCTGT................................................................................. 2

...AGAAGATCTCATGCTGTG................................................................................ 3

...AGAAGATCTCATGCTGTGACT............................................................................. 16

....GAAGATCTCATGCTGTGACT............................................................................. 9

....GAAGATCTCATGCTGTGA............................................................................... 2

....GAAGATCTCATGCTGTG................................................................................ 2

.......................TCTCTGGAGGGAAGCACTTTCTGT...................................................... 1

.......................TCTCTGGAGGGAAGCACTTTCT........................................................ 2

.......................TCTCTGGAGGGAAGCACTTTCTG....................................................... 1

.......................TCTCTGGAGGGAAGCACTTTCTGTT..................................................... 7

........................CTCTGGAGGGAAGCACT............................................................ 3

........................CTCTGGAGGGAAGCACTTTC......................................................... 1

........................CTCTGGAGGGAAGCACTTTCTGT...................................................... 14

........................CTCTGGAGGGAAGCACTTTCTGTT..................................................... 43

........................CTCTGGAGGGAAGCACTTTCTG....................................................... 2

........................CTCTGGAGGGAAGCACTTTCT........................................................ 1

.............................................................CAAAGCGCTTCTCTTTAGA..................... 1

.............................................................CAAAGCGCTTCTCTTTA....................... 4

.............................................................CAAAGCGCTTCTCTTTAGAGTGT................. 8

.............................................................CAAAGCGCTTCTCTTTAG...................... 2

.............................................................CAAAGCGCTTCTCTTTAGAGT................... 17

.............................................................CAAAGCGCTTCTCTTTAGAGTG.................. 20

..............................................................AAAGCGCTTCTCTTTAGAGT................... 3

..............................................................AAAGCGCTTCTCTTTAGA..................... 4

>hsa-mir-187_MI0000274_Homo_sapiens_miR-187_stem-loop differentiating hESC EB library

GGUCGGGCUCACCAUGACACAGUGUGAGACCUCGGGCUACAACACAGGACCCGGGCGCUGCUCUGACCCCUCGUGUCUUGUGUUGCAGCCGGAGGGACGCAGGUCCGCA

.(.((((((.............((((...((((.((((.(((((((((((.((((.((......).).)).)).))))))))))).)))).))))..))))))))))). (-47.91)

.............ATGACACAGTGTGAGACCTCG........................................................................... 1

.................................GGGCTACAACACAGGACCCGG....................................................... 1

...................................GCTACAACACAGGACCCGGG...................................................... 1

...................................GCTACAACACAGGACCCGGGC..................................................... 1

.....................................................................CTCGTGTCTTGTGTTGCAGCCG.................. 2

.....................................................................CTCGTGTCTTGTGTTGC....................... 1

......................................................................TCGTGTCTTGTGTTGCAGCCGGA................ 13

......................................................................TCGTGTCTTGTGTTGCAGCC................... 8

......................................................................TCGTGTCTTGTGTTGCAG..................... 14

......................................................................TCGTGTCTTGTGTTGCAGCCGG................. 130

......................................................................TCGTGTCTTGTGTTGCA...................... 12

......................................................................TCGTGTCTTGTGTTGCAGC.................... 4

......................................................................TCGTGTCTTGTGTTGCAGCCGGAG............... 3

......................................................................TCGTGTCTTGTGTTGCAGCCG.................. 27

.......................................................................CGTGTCTTGTGTTGCAGCCGGA................ 1

.......................................................................CGTGTCTTGTGTTGCAGCC................... 1

.......................................................................CGTGTCTTGTGTTGCAGCCGG................. 18

........................................................................GTGTCTTGTGTTGCAGCCGG................. 2

>hsa-mir-204_MI0000284_Homo_sapiens_miR-204_stem-loop differentiating hESC EB library

GGCUACAGUCUUUCUUCAUGUGACUCGUGGACUUCCCUUUGUCAUCCUAUGCCUGAGAAUAUAUGAAGGAGGCUGGGAAGGCAAAGGGACGUUCAAUUGUCAUCACUGGC

(((....))).....(((((((((...(((((.((((((((((.(((((.((((...............))))))))).)))))))))).)))))...)))).)).))). (-42.26)

...........TTCTTCATGTGACTCGTGGACT............................................................................. 1

............TCTTCATGTGACTCGTGGACTT............................................................................ 1

............TCTTCATGTGACTCGTG................................................................................. 3

............TCTTCATGTGACTCGTGG................................................................................ 4

............TCTTCATGTGACTCGTGGAC.............................................................................. 25

............TCTTCATGTGACTCGTGGACT............................................................................. 6

.............CTTCATGTGACTCGTGG................................................................................ 4

.............CTTCATGTGACTCGTGGAC.............................................................................. 12

..............TTCATGTGACTCGTGGAC.............................................................................. 6

...............TCATGTGACTCGTGGAC.............................................................................. 1

................................TTCCCTTTGTCATCCTATGCCT........................................................ 1505

................................TTCCCTTTGTCATCCTATGCCTG....................................................... 82

................................TTCCCTTTGTCATCCTATGCC......................................................... 43

................................TTCCCTTTGTCATCCTATGC.......................................................... 32

................................TTCCCTTTGTCATCCTATGCCTGA...................................................... 2

................................TTCCCTTTGTCATCCTAT............................................................ 146

................................TTCCCTTTGTCATCCTA............................................................. 8

................................TTCCCTTTGTCATCCTATG........................................................... 41

.................................TCCCTTTGTCATCCTATGC.......................................................... 1

.................................TCCCTTTGTCATCCTATG........................................................... 1

.................................TCCCTTTGTCATCCTAT............................................................ 2

.................................TCCCTTTGTCATCCTATGCCT........................................................ 19

...................................CCTTTGTCATCCTATGCCT........................................................ 2

....................................................................GAGGCTGGGAAGGCAAAGGG...................... 1

....................................................................GAGGCTGGGAAGGCAAAGG....................... 1

......................................................................GGCTGGGAAGGCAAAGGGACGT.................. 1

.......................................................................GCTGGGAAGGCAAAGGGACGTT................. 2

.......................................................................GCTGGGAAGGCAAAGGGACG................... 4

.......................................................................GCTGGGAAGGCAAAGGGACGTTC................ 4

.......................................................................GCTGGGAAGGCAAAGGG...................... 3

.......................................................................GCTGGGAAGGCAAAGGGACGT.................. 19

>hsa-mir-124-2_MI0000444_Homo_sapiens_miR-124-2_stem-loop differentiating hESC EB library

AUCAAGAUUAGAGGCUCUGCUCUCCGUGUUCACAGCGGACCUUGAUUUAAUGUCAUACAAUUAAGGCACGCGGUGAAUGCCAAGAGCGGAGCCUACGGCUGCACUUGAA

.(((((..(((((((((((((((..((((((((.(((..((((((((...........))))))))..))).))))))))..))))))))))))....)))..))))). (-51.20)

...AAGATTAGAGGCTCTGCTCTC..................................................................................... 1

........................CGTGTTCACAGCGGACCTTGATTT............................................................. 3

........................CGTGTTCACAGCGGACCTTG................................................................. 1

........................CGTGTTCACAGCGGACCTTGA................................................................ 3

..........................TGTTCACAGCGGACCTTGATT.............................................................. 1

.............................................................TAAGGCACGCGGTGAATGCCAA.......................... 592

.............................................................TAAGGCACGCGGTGAAT............................... 21

..............................................................AAGGCACGCGGTGAATGCCA........................... 3

..............................................................AAGGCACGCGGTGAATGC............................. 1

>hsa-mir-331_MI0000812_Homo_sapiens_miR-331_stem-loop differentiating hESC EB library

GAGUUUGGUUUUGUUUGGGUUUGUUCUAGGUAUGGUCCCAGGGAUCCCAGAUCAAACCAGGCCCCUGGGCCUAUCCUAGAACCAACCUAAGCUC

............(((((((((.((((((((.((((.(((((((..(.............)..))))))).)))))))))))).))))))))).. (-43.52)

....TTGGTTTTGTTTGGGTTTGTT..................................................................... 1

.....TGGTTTTGTTTGGGTTTGT...................................................................... 2

.....TGGTTTTGTTTGGGTTTGTT..................................................................... 17

......GGTTTTGTTTGGGTTTG....................................................................... 1

......GGTTTTGTTTGGGTTTGTT..................................................................... 15

.......GTTTTGTTTGGGTTTGTT..................................................................... 6

....................TTGTTCTAGGTATGGTCC........................................................ 1

....................TTGTTCTAGGTATGGTCCCAGGGA.................................................. 1

....................TTGTTCTAGGTATGGTCCCAGG.................................................... 1

....................TTGTTCTAGGTATGGTCCCAGGG................................................... 1

.......................TTCTAGGTATGGTCCCAGGGAT................................................. 3

........................TCTAGGTATGGTCCCAGGG................................................... 1

........................TCTAGGTATGGTCCCAGGGAT................................................. 1

.........................CTAGGTATGGTCCCAGGGATCC............................................... 1

.........................CTAGGTATGGTCCCAGGGAT................................................. 2

.........................CTAGGTATGGTCCCAGGG................................................... 1

............................................................GCCCCTGGGCCTATCCT................. 59

............................................................GCCCCTGGGCCTATCCTAGAA............. 434

............................................................GCCCCTGGGCCTATCCTAGAAC............ 9

............................................................GCCCCTGGGCCTATCC.................. 5

............................................................GCCCCTGGGCCTATCCTAG............... 74

............................................................GCCCCTGGGCCTATCCTA................ 36

............................................................GCCCCTGGGCCTATCCTAGA.............. 156

.............................................................CCCCTGGGCCTATCCTA................ 1

.............................................................CCCCTGGGCCTATCCTAGAA............. 1

>hsa-mir-518c_MI0003159_Homo_sapiens_miR-518c_stem-loop differentiating hESC EB library

GCGAGAAGAUCUCAUGCUGUGACUCUCUGGAGGGAAGCACUUUCUGUUGUCUGAAAGAAAACAAAGCGCUUCUCUUUAGAGUGUUACGGUUUGAGAAAAGC

.........(((((.((((((((.((((((((((((((.(((..((((.((.....)).))))))).)))))))))))))).)))))))).)))))..... (-46.40)

...AGAAGATCTCATGCTGTGACT............................................................................. 5

...AGAAGATCTCATGCTGTGA............................................................................... 1

....GAAGATCTCATGCTGTGACT............................................................................. 4

....GAAGATCTCATGCTGTG................................................................................ 1

......................CTCTCTGGAGGGAAGCACTTTCT........................................................ 1

.......................TCTCTGGAGGGAAGCACTTTCT........................................................ 1

.......................TCTCTGGAGGGAAGCACTTTCTGT...................................................... 1

.......................TCTCTGGAGGGAAGCACTTTCTGTT..................................................... 1

........................CTCTGGAGGGAAGCACT............................................................ 1

........................CTCTGGAGGGAAGCACTTTCTGT...................................................... 6

........................CTCTGGAGGGAAGCACTTTCTGTT..................................................... 8

........................CTCTGGAGGGAAGCACTTTCTG....................................................... 1

.............................................................CAAAGCGCTTCTCTTTAGA..................... 4

.............................................................CAAAGCGCTTCTCTTTAGAG.................... 3

.............................................................CAAAGCGCTTCTCTTTAGAGTGTT................ 3

.............................................................CAAAGCGCTTCTCTTTA....................... 11

.............................................................CAAAGCGCTTCTCTTTAGAGTGT................. 6

.............................................................CAAAGCGCTTCTCTTTAG...................... 1

.............................................................CAAAGCGCTTCTCTTTAGAGT................... 23

.............................................................CAAAGCGCTTCTCTTTAGAGTG.................. 29

..............................................................AAAGCGCTTCTCTTTAGAGT................... 4

..............................................................AAAGCGCTTCTCTTTAGAGTG.................. 1

..............................................................AAAGCGCTTCTCTTTAG...................... 7

..............................................................AAAGCGCTTCTCTTTAGA..................... 7

>hsa-mir-769_MI0003834_Homo_sapiens_miR-769_stem-loop differentiating hESC EB library

GCCUUGGUGCUGAUUCCUGGGCUCUGACCUGAGACCUCUGGGUUCUGAGCUGUGAUGUUGCUCUCGAGCUGGGAUCUCCGGGGUCUUGGUUCAGGGCCGGGGCCUCUGGGUUCCAAGC

..(((((.(((((..(((.((((((((.(..(((((((.(((.(((.((((.(((........))))))).))).))).)))))))..).)))))))).)))..))..))).))))). (-60.10)

.........CTGATTCCTGGGCTCTGACCT........................................................................................ 1

.............................TGAGACCTCTGGGTTCTGAGCT................................................................... 298

.............................TGAGACCTCTGGGTTCTGAGC.................................................................... 54

.............................TGAGACCTCTGGGTTCT........................................................................ 9

.............................TGAGACCTCTGGGTTCTGAGCTGT................................................................. 4

.............................TGAGACCTCTGGGTTCTGAGCTG.................................................................. 16

.............................TGAGACCTCTGGGTTCTGA...................................................................... 17

.............................TGAGACCTCTGGGTTCTGAG..................................................................... 10

.............................TGAGACCTCTGGGTTCTG....................................................................... 32

..............................GAGACCTCTGGGTTCTGAGCT................................................................... 6

..............................GAGACCTCTGGGTTCTGAGC.................................................................... 1

..............................GAGACCTCTGGGTTCTGAG..................................................................... 1

..................................CCTCTGGGTTCTGAGCT................................................................... 1

....................................................................CTGGGATCTCCGGGGTCTT............................... 1

....................................................................CTGGGATCTCCGGGGTCTTGGTT........................... 5

....................................................................CTGGGATCTCCGGGGTCTTGGT............................ 2

....................................................................CTGGGATCTCCGGGGTCTTGG............................. 1

.....................................................................TGGGATCTCCGGGGTCTTGGTT........................... 21

.....................................................................TGGGATCTCCGGGGTCTTGGTTC.......................... 1

.....................................................................TGGGATCTCCGGGGTCTTG.............................. 1

.....................................................................TGGGATCTCCGGGGTCTTGGT............................ 1
