# Supplementary material for: Multiple distinct small RNAs originate from the same microRNA precursors
Source: Genome Biol. 2010 Aug 9;11(8):R81. doi: 10.1186/gb-2010-11-8-r81 (PMC2945783; doi:10.1186/gb-2010-11-8-r81)
Supplement: Additional file 8 — Supplemental File S7. This is a file for sequencing reads mapped and aligned to miRNA precursors that can produce miRNA-sibling small RNAs (msRNAs) in M. musculus (mms). The sequencing data were obtained from GEO; see Materials and methods for details. [file gb-2010-11-8-r81-S8.DOCX]

Zhang, et al., Multiple distinct small RNAs originate from the same microRNA precursors

Supplemental File 7 - Sequencing reads mapped and aligned to miRNA precursors that can produce

miRNA-like RNAs in *Mus musculus.*

>mmu-mir-20a_MI0000568_Mus_musculus_miR-20a_stem-loop ESC wild type

GUGUGAUGUGACAGCUUCUGUAGCACUAAAGUGCUUAUAGUGCAGGUAGUGUGUAGCCAUCUACUGCAUUACGAGCACUUAAAGUACUGCCAGCUGUAGAACUCCAG

.((.((.((.((((((...((((.(((.(((((((..((((((((.(((.(((....))))))))))))))..)))))))..))).)))).))))))...)))))). (-41.00)

....GATGTGACAGCTTCTGTAGCAC................................................................................. 2

.....ATGTGACAGCTTCTGT...................................................................................... 2

.....ATGTGACAGCTTCTGTAGCAC................................................................................. 3

........................ACTAAAGTGCTTATAGTGCAGGTAG.......................................................... 1

.........................CTAAAGTGCTTATAGTGCAG.............................................................. 1

.........................CTAAAGTGCTTATAGTGCAGGTAGT......................................................... 3

.........................CTAAAGTGCTTATAGTGCAGGTA........................................................... 5

.........................CTAAAGTGCTTATAGTGCAGG............................................................. 1

.........................CTAAAGTGCTTATAGTGCAGGT............................................................ 4

.........................CTAAAGTGCTTATAGTGCA............................................................... 11

.........................CTAAAGTGCTTATAGT.................................................................. 2

.........................CTAAAGTGCTTATAGTG................................................................. 3

.........................CTAAAGTGCTTATAGTGCAGGTAG.......................................................... 53

.........................CTAAAGTGCTTATAGTGC................................................................ 1

..........................TAAAGTGCTTATAGTGCAGGTA........................................................... 2087

..........................TAAAGTGCTTATAGTGCAG.............................................................. 139

..........................TAAAGTGCTTATAGTGCAGGTAGTGT....................................................... 6

..........................TAAAGTGCTTATAGTGCAGGTAG.......................................................... 32642

..........................TAAAGTGCTTATAGTGCAGGTAGTGTG...................................................... 1

..........................TAAAGTGCTTATAGTG................................................................. 285

..........................TAAAGTGCTTATAGTGCAGG............................................................. 1269

..........................TAAAGTGCTTATAGTGCAGGT............................................................ 3568

..........................TAAAGTGCTTATAGTGCAGGTAGTG........................................................ 2

..........................TAAAGTGCTTATAGTGC................................................................ 1341

..........................TAAAGTGCTTATAGTGCAGGTAGT......................................................... 1110

..........................TAAAGTGCTTATAGTGCA............................................................... 2986

...........................AAAGTGCTTATAGTGCAGGT............................................................ 5

...........................AAAGTGCTTATAGTGCAGGTAGT......................................................... 1

...........................AAAGTGCTTATAGTGCAGGTA........................................................... 4

...........................AAAGTGCTTATAGTGCAGGTAG.......................................................... 63

...........................AAAGTGCTTATAGTGCAGG............................................................. 1

...........................AAAGTGCTTATAGTGCA............................................................... 5

...........................AAAGTGCTTATAGTGC................................................................ 7

............................AAGTGCTTATAGTGCAGGTA........................................................... 3

............................AAGTGCTTATAGTGCA............................................................... 3

............................AAGTGCTTATAGTGCAGGT............................................................ 4

............................AAGTGCTTATAGTGCAGGTAGT......................................................... 1

............................AAGTGCTTATAGTGCAGG............................................................. 1

............................AAGTGCTTATAGTGCAGGTAG.......................................................... 24

.............................AGTGCTTATAGTGCAGGT............................................................ 3

.............................AGTGCTTATAGTGCAGGTAGT......................................................... 4

.............................AGTGCTTATAGTGCAGG............................................................. 3

.............................AGTGCTTATAGTGCAGGTAG.......................................................... 70

.............................AGTGCTTATAGTGCAGGTA........................................................... 5

..............................GTGCTTATAGTGCAGGTA........................................................... 20

..............................GTGCTTATAGTGCAGGT............................................................ 28

..............................GTGCTTATAGTGCAGGTAGT......................................................... 6

..............................GTGCTTATAGTGCAGG............................................................. 7

..............................GTGCTTATAGTGCAGGTAG.......................................................... 144

...............................TGCTTATAGTGCAGGTAG.......................................................... 42

...............................TGCTTATAGTGCAGGT............................................................ 10

...............................TGCTTATAGTGCAGGTA........................................................... 17

................................GCTTATAGTGCAGGTA........................................................... 10

................................GCTTATAGTGCAGGTAG.......................................................... 190

................................GCTTATAGTGCAGGTAGT......................................................... 1

.................................CTTATAGTGCAGGTAGT......................................................... 8

.................................CTTATAGTGCAGGTAG.......................................................... 110

..................................TTATAGTGCAGGTAGT......................................................... 133

................................................GTGTGTAGCCATCTACT.......................................... 1

................................................GTGTGTAGCCATCTACTGCATTACG.................................. 2

..........................................................ATCTACTGCATTACGAGCACTT........................... 1

..............................................................ACTGCATTACGAGCACTTAAAG....................... 11

..............................................................ACTGCATTACGAGCACTTAAAGT...................... 53

..............................................................ACTGCATTACGAGCACTTAA......................... 4

..............................................................ACTGCATTACGAGCACTTAAA........................ 4

..............................................................ACTGCATTACGAGCACTTAAAGTA..................... 2

..............................................................ACTGCATTACGAGCACTTA.......................... 1

...............................................................CTGCATTACGAGCACTTAAAGT...................... 12

................................................................TGCATTACGAGCACTTAAAGT...................... 16

..................................................................CATTACGAGCACTTAAA........................ 1

..................................................................CATTACGAGCACTTAAAGT...................... 2

..................................................................CATTACGAGCACTTAA......................... 1

...................................................................ATTACGAGCACTTAAA........................ 1

...................................................................ATTACGAGCACTTAAAGT...................... 2

...................................................................ATTACGAGCACTTAAAGTA..................... 1

....................................................................TTACGAGCACTTAAAGT...................... 5

....................................................................TTACGAGCACTTAAAG....................... 6

>mmu-mir-96_MI0000583_Mus_musculus_miR-96_stem-loop ESC wild type

CCAGUACCAUCUGCUUGGCCGAUUUUGGCACUAGCACAUUUUUGCUUGUGUCUCUCCGCUGUGAGCAAUCAUGUGUAGUGCCAAUAUGGGAAAAGCGGGCUGCUGC

.(((((...(((((((..((.((.(((((((((.(((((..(((((..(((......)).)..)))))..)))))))))))))).)).))..))))))).))))). (-47.30)

...GTACCATCTGCTTGGCC...................................................................................... 1

...GTACCATCTGCTTGGCCGAT................................................................................... 3

....TACCATCTGCTTGGCCGAT................................................................................... 4

....TACCATCTGCTTGGCCG..................................................................................... 2

....TACCATCTGCTTGGCCGA.................................................................................... 2

.....ACCATCTGCTTGGCCGAT................................................................................... 7

......CCATCTGCTTGGCCGA.................................................................................... 1

......CCATCTGCTTGGCCGAT................................................................................... 5

......................TTTTGGCACTAGCACATTTTTGC............................................................. 1

.......................TTTGGCACTAGCACATTTTT............................................................... 27

.......................TTTGGCACTAGCACATTTTTG.............................................................. 32

.......................TTTGGCACTAGCACATTTTTGCT............................................................ 22244

.......................TTTGGCACTAGCACATTT................................................................. 26

.......................TTTGGCACTAGCACATTTTTGC............................................................. 1029

.......................TTTGGCACTAGCACATTTT................................................................ 22

.......................TTTGGCACTAGCACAT................................................................... 235

.......................TTTGGCACTAGCACATTTTTGCTT........................................................... 183

.......................TTTGGCACTAGCACATT.................................................................. 97

........................TTGGCACTAGCACATTTTTGCT............................................................ 64

........................TTGGCACTAGCACATTTTTGC............................................................. 3

........................TTGGCACTAGCACATTTTTG.............................................................. 3

.........................TGGCACTAGCACATTTTTGCT............................................................ 23

.........................TGGCACTAGCACATTTTTGC............................................................. 3

..........................GGCACTAGCACATTTTTGCT............................................................ 1

...........................GCACTAGCACATTTTTGCT............................................................ 13

...........................GCACTAGCACATTTTTG.............................................................. 1

...........................GCACTAGCACATTTTTGC............................................................. 1

............................CACTAGCACATTTTTGCTT........................................................... 69

............................CACTAGCACATTTTTG.............................................................. 18

............................CACTAGCACATTTTTGCT............................................................ 6488

............................CACTAGCACATTTTTGC............................................................. 762

.............................ACTAGCACATTTTTGCT............................................................ 399

.............................ACTAGCACATTTTTGC............................................................. 32

..............................CTAGCACATTTTTGCTT........................................................... 7

..............................CTAGCACATTTTTGCT............................................................ 1606

...............................TAGCACATTTTTGCTT........................................................... 1

..............................................TGTGTCTCTCCGCTGTGAG......................................... 4

.................................................................CAATCATGTGTAGTGCCAATAT................... 98

.................................................................CAATCATGTGTAGTGCC........................ 1

.................................................................CAATCATGTGTAGTGCCAA...................... 3

.................................................................CAATCATGTGTAGTGCCA....................... 9

.................................................................CAATCATGTGTAGTGC......................... 12

.................................................................CAATCATGTGTAGTGCCAATA.................... 8

.................................................................CAATCATGTGTAGTGCCAAT..................... 30

..................................................................AATCATGTGTAGTGCCAAT..................... 1

..................................................................AATCATGTGTAGTGCCAATAT................... 9

..................................................................AATCATGTGTAGTGCCA....................... 1

......................................................................ATGTGTAGTGCCAATAT................... 1

.......................................................................TGTGTAGTGCCAATAT................... 8

>mmu-mir-328_MI0000603_Mus_musculus_miR-328_stem-loop ESC wild type

CUGUCUCGGAGCCUGGGGCAGGGGGGCAGGAGGGGCUCAGGGAGAAAGUAUCUACAGCCCCUGGCCCUCUCUGCCCUUCCGUCCCCUGUCCCCAAGU

.......((..(..(((((.(((((((((..(((((.(((((.(...((....))..)))))))))))..))))))))).)))))..)..))..... (-52.40)

.TGTCTCGGAGCCTGGGGCA............................................................................. 3

..GTCTCGGAGCCTGGGGCA............................................................................. 4

....CTCGGAGCCTGGGGCA............................................................................. 2

.....................GGGGGCAGGAGGGGCTCAGGG....................................................... 1

..........................................AGAAAGTATCTACAGCCC..................................... 4

............................................................CTGGCCCTCTCTGCCCTTCCGT............... 36

............................................................CTGGCCCTCTCTGCCC..................... 1

............................................................CTGGCCCTCTCTGCCCTTCCG................ 5

>mmu-mir-329_MI0000605_Mus_musculus_miR-329_stem-loop ESC wild type

UGUUCGCUUCUGGUACCGGAAGAGAGGUUUUCUGGGUCUCUGUUUCUUUGAUGAGAAUGAAACACACCCAGCUAACCUUUUUUUCAGUAUCAAAUCC

..........((((((.((((((((((((..((((((...(((((((((.....))).)))))).))))))..)))))))))))).))))))..... (-36.50)

.GTTCGCTTCTGGTACCGGAAG........................................................................... 1

......................AGAGGTTTTCTGGGTCTCTG....................................................... 2

......................AGAGGTTTTCTGGGTCTC......................................................... 17

......................AGAGGTTTTCTGGGTCTCTGTT..................................................... 45

......................AGAGGTTTTCTGGGTCTCTGT...................................................... 43

......................AGAGGTTTTCTGGGTCTCTGTTTC................................................... 297

......................AGAGGTTTTCTGGGTCTCT........................................................ 26

......................AGAGGTTTTCTGGGTCTCTGTTTCT.................................................. 31

......................AGAGGTTTTCTGGGTCTCTGTTT.................................................... 130

......................AGAGGTTTTCTGGGTCT.......................................................... 3

......................AGAGGTTTTCTGGGTC........................................................... 12

.......................GAGGTTTTCTGGGTCTCTGTTTCT.................................................. 1

.......................GAGGTTTTCTGGGTCTCTGTTTC................................................... 7

........................AGGTTTTCTGGGTCTCTGTTTC................................................... 2

.........................GGTTTTCTGGGTCTCT........................................................ 1

.........................GGTTTTCTGGGTCTCTGTTT.................................................... 3

.........................GGTTTTCTGGGTCTCTGTTTC................................................... 3

..........................GTTTTCTGGGTCTCTGTTTC................................................... 1

..........................GTTTTCTGGGTCTCTGTTT.................................................... 2

...........................TTTTCTGGGTCTCTGTTT.................................................... 2

...........................TTTTCTGGGTCTCTGTTTC................................................... 6

............................TTTCTGGGTCTCTGTTT.................................................... 1

............................TTTCTGGGTCTCTGTTTC................................................... 1

............................................................AACACACCCAGCTAACC.................... 42

............................................................AACACACCCAGCTAACCTTTTTT.............. 44

............................................................AACACACCCAGCTAACCT................... 55

............................................................AACACACCCAGCTAACCTTT................. 6

............................................................AACACACCCAGCTAACCTT.................. 12

............................................................AACACACCCAGCTAAC..................... 37

............................................................AACACACCCAGCTAACCTTTTTTT............. 1

............................................................AACACACCCAGCTAACCTTTT................ 20

............................................................AACACACCCAGCTAACCTTTTT............... 207

.............................................................ACACACCCAGCTAACCTT.................. 3

.............................................................ACACACCCAGCTAACCTTTTT............... 2

.............................................................ACACACCCAGCTAACCTTTTTT.............. 5

.............................................................ACACACCCAGCTAACCTTT................. 1

.............................................................ACACACCCAGCTAACCT................... 1

.............................................................ACACACCCAGCTAACC.................... 3

..............................................................CACACCCAGCTAACCT................... 1

..............................................................CACACCCAGCTAACCTTTTT............... 1

...............................................................ACACCCAGCTAACCTTTT................ 1

................................................................CACCCAGCTAACCTTTTT............... 1

.................................................................ACCCAGCTAACCTTTTT............... 2

.................................................................ACCCAGCTAACCTTTT................ 1

..................................................................CCCAGCTAACCTTTTT............... 4

...................................................................CCAGCTAACCTTTTTT.............. 1

>mmu-mir-331_MI0000609_Mus_musculus_miR-331_stem-loop ESC wild type

GAGUCUGGUUUUGUUUGGGUUUGUUCUAGGUAUGGUCCCAGGGAUCCCAGAUCAAACCAGGCCCCUGGGCCUAUCCUAGAACCAACCUAAACCCGU

......((....(((((((((.((((((((.((((.(((((((..(.............)..))))))).)))))))))))).))))))))))).. (-43.72)

.....TGGTTTTGTTTGGGTTTGTT....................................................................... 4

.....TGGTTTTGTTTGGGTT........................................................................... 1

......GGTTTTGTTTGGGTTTGTT....................................................................... 3

.......GTTTTGTTTGGGTTTGTT....................................................................... 2

........................TCTAGGTATGGTCCCAGGGAT................................................... 1

........................TCTAGGTATGGTCCCAGGGATC.................................................. 2

.........................CTAGGTATGGTCCCAGGGAT................................................... 1

.........................CTAGGTATGGTCCCAGGGATC.................................................. 5

.........................CTAGGTATGGTCCCAGGGATCC................................................. 5

..............................TATGGTCCCAGGGATC.................................................. 2

............................................................GCCCCTGGGCCTATCCTAGAAC.............. 2

............................................................GCCCCTGGGCCTATCCTA.................. 2

............................................................GCCCCTGGGCCTATCCTAGAA............... 47

............................................................GCCCCTGGGCCTATCCTAGA................ 25

.............................................................CCCCTGGGCCTATCCTAGAA............... 1

.............................................................CCCCTGGGCCTATCCTAGA................ 1

.............................................................CCCCTGGGCCTATCCTAGAAC.............. 1

>mmu-mir-337_MI0000615_Mus_musculus_miR-337_stem-loop ESC wild type

CAGUGUAGUGAGAAGUUGGGGGGUGGGAACGGCGUCAUGCAGGAGUUGAUUGCACAGCCAUUCAGCUCCUAUAUGAUGCCUUUCUUCACCCCCUUCA

..............(..((((((((((((.(((((((((.(((((((((.((......)).))))))))).))))))))).))).))))))))).). (-49.60)

......AGTGAGAAGTTGGGGGGTGGGA..................................................................... 1

........................GGGAACGGCGTCATGCAGGAGT................................................... 1

.........................GGAACGGCGTCATGCAGGAGTT.................................................. 1

..........................GAACGGCGTCATGCAGGAGTTG................................................. 1

..........................GAACGGCGTCATGCAGGAGTT.................................................. 1

..........................GAACGGCGTCATGCAGGAGTTGA................................................ 9

...........................AACGGCGTCATGCAGGAGTTG................................................. 6

...........................AACGGCGTCATGCAGGAGTTGA................................................ 19

............................ACGGCGTCATGCAGGAGTTGA................................................ 1

.............................CGGCGTCATGCAGGAG.................................................... 2

.............................CGGCGTCATGCAGGAGTTGA................................................ 19

.............................CGGCGTCATGCAGGAGTTGATT.............................................. 788

.............................CGGCGTCATGCAGGAGTTGAT............................................... 118

.............................CGGCGTCATGCAGGAGT................................................... 1

.............................CGGCGTCATGCAGGAGTTGATTG............................................. 5

..............................GGCGTCATGCAGGAGTTGATTG............................................. 21

..............................GGCGTCATGCAGGAGTTGA................................................ 2

..............................GGCGTCATGCAGGAGTTG................................................. 1

..............................GGCGTCATGCAGGAGTTGATT.............................................. 15

...............................GCGTCATGCAGGAGTTGA................................................ 2

...............................GCGTCATGCAGGAGTTGATT.............................................. 5

................................CGTCATGCAGGAGTTGA................................................ 19

................................CGTCATGCAGGAGTTGATT.............................................. 70

................................CGTCATGCAGGAGTTGAT............................................... 17

................................CGTCATGCAGGAGTTG................................................. 3

.................................GTCATGCAGGAGTTGATT.............................................. 3

..................................TCATGCAGGAGTTGATT.............................................. 4

...........................................................ATTCAGCTCCTATATGATGCCTT............... 1

...........................................................ATTCAGCTCCTATATGATGCCT................ 1

............................................................TTCAGCTCCTATATGATGCCT................ 23

............................................................TTCAGCTCCTATATGATGCCTTT.............. 168

............................................................TTCAGCTCCTATATGATG................... 3

............................................................TTCAGCTCCTATATGA..................... 12

............................................................TTCAGCTCCTATATGAT.................... 12

............................................................TTCAGCTCCTATATGATGC.................. 23

............................................................TTCAGCTCCTATATGATGCC................. 12

............................................................TTCAGCTCCTATATGATGCCTT............... 124

.............................................................TCAGCTCCTATATGATGCC................. 15

.............................................................TCAGCTCCTATATGATGCCTTTC............. 1

.............................................................TCAGCTCCTATATGAT.................... 20

.............................................................TCAGCTCCTATATGATGCCTTT.............. 1071

.............................................................TCAGCTCCTATATGATGCCTT............... 64

.............................................................TCAGCTCCTATATGATGC.................. 298

.............................................................TCAGCTCCTATATGATG................... 7

.............................................................TCAGCTCCTATATGATGCCT................ 5

..............................................................CAGCTCCTATATGATGC.................. 2

...............................................................AGCTCCTATATGATGCCTT............... 1

...............................................................AGCTCCTATATGATGCCTTTCT............ 1

...............................................................AGCTCCTATATGATGCCTTTCTT........... 2

...............................................................AGCTCCTATATGATGCCTTTCTTC.......... 1

...............................................................AGCTCCTATATGATGCCTTT.............. 25

...............................................................AGCTCCTATATGATGC.................. 4

...............................................................AGCTCCTATATGATGCCT................ 1

...............................................................AGCTCCTATATGATGCCTTTC............. 1

...............................................................AGCTCCTATATGATGCC................. 2

.................................................................CTCCTATATGATGCCTTTCTTC.......... 1

.................................................................CTCCTATATGATGCCTTT.............. 3

..................................................................TCCTATATGATGCCTTT.............. 3

..................................................................TCCTATATGATGCCTTTCTTC.......... 1

..................................................................TCCTATATGATGCCTT............... 1

...................................................................CCTATATGATGCCTTT.............. 9

>mmu-mir-341_MI0000625_Mus_musculus_miR-341_stem-loop ESC wild type

AAAAUGAUGAUGUCAGUUGGCCGGUCGGCCGAUCGCUCGGUCUGUCAGUCAGUCGGUCGGUCGAUCGGUCGGUCGGUCAGUCGGCUUCCUGUCUUC

.....(((((.(((..(((((((..((((((((((..(((.(((.(.....).))))))..))))))))))..)))))))..))).))..)))... (-47.10)

AAAATGATGATGTCAGTTGGC........................................................................... 1

.AAATGATGATGTCAGTTGGC........................................................................... 2

..AATGATGATGTCAGTTGGC........................................................................... 1

....TGATGATGTCAGTTGGCCG......................................................................... 1

......ATGATGTCAGTTGGCCGGT....................................................................... 1

.....................CGGTCGGCCGATCGCTCGGTCT..................................................... 1

.....................CGGTCGGCCGATCGCTCGGTC...................................................... 4

........................TCGGCCGATCGCTCGGTC...................................................... 1

.........................CGGCCGATCGCTCGGTC...................................................... 2

.......................................................GTCGGTCGATCGGTCGGTCGGT................... 4

.......................................................GTCGGTCGATCGGTCGGTCGG.................... 1

........................................................TCGGTCGATCGGTCGGT....................... 9

........................................................TCGGTCGATCGGTCGG........................ 1

........................................................TCGGTCGATCGGTCGGTCGG.................... 6

........................................................TCGGTCGATCGGTCGGTC...................... 7

........................................................TCGGTCGATCGGTCGGTCGGTC.................. 4

........................................................TCGGTCGATCGGTCGGTCG..................... 6

........................................................TCGGTCGATCGGTCGGTCGGT................... 100

..........................................................GGTCGATCGGTCGGTCGGT................... 2

...........................................................GTCGATCGGTCGGTCGG.................... 6

...........................................................GTCGATCGGTCGGTCGGTC.................. 1

...........................................................GTCGATCGGTCGGTCGGT................... 8

...........................................................GTCGATCGGTCGGTCG..................... 1

...........................................................GTCGATCGGTCGGTCGGTCAGT............... 4

............................................................TCGATCGGTCGGTCGGTCAG................ 6

............................................................TCGATCGGTCGGTCGGTCAGT............... 83

............................................................TCGATCGGTCGGTCGGTC.................. 5

............................................................TCGATCGGTCGGTCGGT................... 21

...............................................................ATCGGTCGGTCGGTCAGT............... 2

................................................................TCGGTCGGTCGGTCAGT............... 1

>mmu-mir-101b_MI0000649_Mus_musculus_miR-101b_stem-loop ESC wild type

AUCUGAGACUGAACUGCCCUUUUUCGGUUAUCAUGGUACCGAUGCUGUAGCUCUGAAAGGUACAGUACUGUGAUAGCUGAAGAAUGGCGGUGCCAUC

.........((.((((((.((((((((((((((((((......((((((.((.....)).)))))))))))))))))))))))).))))))..)).. (-39.70)

..CTGAGACTGAACTGCCCTTTT.......................................................................... 1

..CTGAGACTGAACTGCCCT............................................................................. 1

......GACTGAACTGCCCTTTTT......................................................................... 3

.......ACTGAACTGCCCTTTTT......................................................................... 1

.......................TCGGTTATCATGGTACCGATGCT................................................... 2

.......................TCGGTTATCATGGTACCGATGC.................................................... 4

........................CGGTTATCATGGTACCGATGCT................................................... 5

........................CGGTTATCATGGTACCGATGCTG.................................................. 3

........................CGGTTATCATGGTACC......................................................... 2

...........................................................GTACAGTACTGTGATAG..................... 56

...........................................................GTACAGTACTGTGATAGCTGA................. 425

...........................................................GTACAGTACTGTGATAGCTG.................. 22

...........................................................GTACAGTACTGTGATAGCT................... 88

...........................................................GTACAGTACTGTGATAGCTGAA................ 630

...........................................................GTACAGTACTGTGATAGC.................... 30

...........................................................GTACAGTACTGTGATA...................... 99

...........................................................GTACAGTACTGTGATAGCTGAAG............... 1

............................................................TACAGTACTGTGATAGCTGAAG............... 1235

............................................................TACAGTACTGTGATAGCTG.................. 38

............................................................TACAGTACTGTGATAG..................... 75

............................................................TACAGTACTGTGATAGCTGAA................ 890

............................................................TACAGTACTGTGATAGCTGA................. 1173

............................................................TACAGTACTGTGATAGCTGAAGAA............. 4

............................................................TACAGTACTGTGATAGCTGAAGA.............. 50

............................................................TACAGTACTGTGATAGC.................... 189

............................................................TACAGTACTGTGATAGCT................... 380

.............................................................ACAGTACTGTGATAGCTGAA................ 4

.............................................................ACAGTACTGTGATAGCTGAAG............... 9

.............................................................ACAGTACTGTGATAGCTGA................. 25

.............................................................ACAGTACTGTGATAGCT................... 4

.............................................................ACAGTACTGTGATAGCTG.................. 1

.............................................................ACAGTACTGTGATAGC.................... 2

.............................................................ACAGTACTGTGATAGCTGAAGA.............. 11

..............................................................CAGTACTGTGATAGCTGAAG............... 3

..............................................................CAGTACTGTGATAGCT................... 2

..............................................................CAGTACTGTGATAGCTGAA................ 2

..............................................................CAGTACTGTGATAGCTGA................. 6

..............................................................CAGTACTGTGATAGCTGAAGA.............. 1

...............................................................AGTACTGTGATAGCTG.................. 4

...............................................................AGTACTGTGATAGCTGA................. 5

................................................................GTACTGTGATAGCTGA................. 5

................................................................GTACTGTGATAGCTGAA................ 8

.................................................................TACTGTGATAGCTGAA................ 1

>mmu-mir-138-1_MI0000722_Mus_musculus_miR-138-1_stem-loop ESC wild type

CUCUAGCAUGGUGUUGUGGGACAGCUGGUGUUGUGAAUCAGGCCGUUGCCAAUCAGAGAACGGCUACUUCACAACACCAGGGCCACACUGCACUGCAAG

.....(((..((((.(((((.(..(((((((((((((...(((((((..(.....)..)))))))..))))))))))))).))).))).)))))))... (-45.60)

..CTAGCATGGTGTTGTGGGAC............................................................................. 1

......................AGCTGGTGTTGTGAAT............................................................. 2

......................AGCTGGTGTTGTGAATCAGGCCG...................................................... 35

......................AGCTGGTGTTGTGAATCAGGCC....................................................... 18

......................AGCTGGTGTTGTGAATCAGGCCGTT.................................................... 10

......................AGCTGGTGTTGTGAATCAGGC........................................................ 37

......................AGCTGGTGTTGTGAATC............................................................ 7

......................AGCTGGTGTTGTGAATCA........................................................... 1

......................AGCTGGTGTTGTGAATCAGG......................................................... 5

......................AGCTGGTGTTGTGAATCAGGCCGT..................................................... 35

......................AGCTGGTGTTGTGAATCAG.......................................................... 1

..........................GGTGTTGTGAATCAGGCCG...................................................... 1

..........................GGTGTTGTGAATCAGGCC....................................................... 1

............................TGTTGTGAATCAGGCCG...................................................... 1

.............................GTTGTGAATCAGGCCG...................................................... 1

.............................................TTGCCAATCAGAGAACG..................................... 1

>mmu-mir-7a-1_MI0000728_Mus_musculus_miR-7a-1_stem-loop ESC wild type

UUGGAUGUUGGCCUAGUUCUGUGUGGAAGACUAGUGAUUUUGUUGUUUUUAGAUAACUAAAACGACAACAAAUCACAGUCUGCCAUAUGGCACAGGCCACCUCUACAG

.((((.((.(((((.((.((((((((.(((((.(((((((.(((((((((((....)))))..)))))))))))))))))).)))))))).))))))))).))))... (-47.10)

..GGATGTTGGCCTAGTTCTGTG..................................................................................... 1

...GATGTTGGCCTAGTTCTGTG..................................................................................... 1

....ATGTTGGCCTAGTTCTGTG..................................................................................... 2

.....TGTTGGCCTAGTTCTGTG..................................................................................... 1

.......................TGGAAGACTAGTGATTT.................................................................... 3

.......................TGGAAGACTAGTGATTTTGTTG............................................................... 2232

.......................TGGAAGACTAGTGATTTTGTT................................................................ 557

.......................TGGAAGACTAGTGATTTTG.................................................................. 9

.......................TGGAAGACTAGTGATTTTGTTGTTT............................................................ 270

.......................TGGAAGACTAGTGATTTTGTTGTT............................................................. 10482

.......................TGGAAGACTAGTGATTTTGTTGT.............................................................. 7044

.......................TGGAAGACTAGTGATTTTGT................................................................. 87

.......................TGGAAGACTAGTGATTTTGTTGTTTT........................................................... 19

.......................TGGAAGACTAGTGATT..................................................................... 6

.......................TGGAAGACTAGTGATTTTGTTGTTTTT.......................................................... 1

........................GGAAGACTAGTGATTTT................................................................... 2

........................GGAAGACTAGTGATTTTGTTGT.............................................................. 33

........................GGAAGACTAGTGATTTTGTTGTT............................................................. 43

........................GGAAGACTAGTGATTTTGTTG............................................................... 8

.........................GAAGACTAGTGATTTTGTTGTT............................................................. 1

.........................GAAGACTAGTGATTTTGTTG............................................................... 1

.........................GAAGACTAGTGATTTTGTTGT.............................................................. 12

.........................GAAGACTAGTGATTTTGTTGTTT............................................................ 1

.........................GAAGACTAGTGATTTTGT................................................................. 1

..........................AAGACTAGTGATTTTGTTGT.............................................................. 1

..........................AAGACTAGTGATTTTGTTGTT............................................................. 3

..........................AAGACTAGTGATTTTGTT................................................................ 1

...........................AGACTAGTGATTTTGTTGTTT............................................................ 23

...........................AGACTAGTGATTTTGTTGT.............................................................. 697

...........................AGACTAGTGATTTTGTTG............................................................... 209

...........................AGACTAGTGATTTTGTT................................................................ 115

...........................AGACTAGTGATTTTGTTGTT............................................................. 1080

...........................AGACTAGTGATTTTGTTGTTTT........................................................... 1

...........................AGACTAGTGATTTTGT................................................................. 187

............................GACTAGTGATTTTGTTGTTT............................................................ 1

............................GACTAGTGATTTTGTT................................................................ 17

............................GACTAGTGATTTTGTTG............................................................... 59

............................GACTAGTGATTTTGTTGT.............................................................. 181

............................GACTAGTGATTTTGTTGTT............................................................. 175

.............................ACTAGTGATTTTGTTGTTT............................................................ 15

.............................ACTAGTGATTTTGTTGT.............................................................. 711

.............................ACTAGTGATTTTGTTGTTTT........................................................... 1

.............................ACTAGTGATTTTGTTGTT............................................................. 488

.............................ACTAGTGATTTTGTTG............................................................... 349

..............................CTAGTGATTTTGTTGTTT............................................................ 13

..............................CTAGTGATTTTGTTGTT............................................................. 515

..............................CTAGTGATTTTGTTGT.............................................................. 900

..............................CTAGTGATTTTGTTGTTTT........................................................... 1

...............................TAGTGATTTTGTTGTT............................................................. 43

...............................TAGTGATTTTGTTGTTT............................................................ 2

................................AGTGATTTTGTTGTTT............................................................ 1

...............................................................GACAACAAATCACAGTCTGCCAT...................... 2

.................................................................CAACAAATCACAGTCTGCCAT...................... 26

.................................................................CAACAAATCACAGTCT........................... 1

.................................................................CAACAAATCACAGTCTG.......................... 3

.................................................................CAACAAATCACAGTCTGCCA....................... 2

.................................................................CAACAAATCACAGTCTGCCATA..................... 86

.................................................................CAACAAATCACAGTCTGCC........................ 3

..................................................................AACAAATCACAGTCTGCCAT...................... 4

..................................................................AACAAATCACAGTCTGCCATA..................... 17

..................................................................AACAAATCACAGTCTGCCATAT.................... 1

...................................................................ACAAATCACAGTCTGCCATAT.................... 2

.....................................................................AAATCACAGTCTGCCAT...................... 2

......................................................................AATCACAGTCTGCCAT...................... 5

......................................................................AATCACAGTCTGCCATA..................... 2

>mmu-mir-434_MI0001526_Mus_musculus_miR-434_stem-loop ESC wild type

UCGACUCUGGGUUUGAACCAAAGCUCGACUCAUGGUUUGAACCAUUACUUAAUUCGUGGUUUGAACCAUCACUCGACUCCUGGUUCGAACCAUC

.........(((((((((((.((.((((.(.((((((..((((((..........))))))..)))))).).))))))..)))))))))))... (-38.80)

.CGACTCTGGGTTTGAACCAA......................................................................... 1

...ACTCTGGGTTTGAACCAAA........................................................................ 1

...................AAAGCTCGACTCATGGTTT........................................................ 1

.....................AGCTCGACTCATGGTTTGAACC................................................... 280

.....................AGCTCGACTCATGGTTT........................................................ 1

.....................AGCTCGACTCATGGTT......................................................... 4

.....................AGCTCGACTCATGGTTTGAA..................................................... 18

.....................AGCTCGACTCATGGTTTGAACCAT................................................. 1

.....................AGCTCGACTCATGGTTTGA...................................................... 4

.....................AGCTCGACTCATGGTTTGAACCA.................................................. 145

.....................AGCTCGACTCATGGTTTGAAC.................................................... 70

......................GCTCGACTCATGGTTTGA...................................................... 8

......................GCTCGACTCATGGTTT........................................................ 6

......................GCTCGACTCATGGTTTGAACCAT................................................. 4

......................GCTCGACTCATGGTTTGAACCA.................................................. 321

......................GCTCGACTCATGGTTTGAAC.................................................... 164

......................GCTCGACTCATGGTTTGAACC................................................... 513

......................GCTCGACTCATGGTTTG....................................................... 2

......................GCTCGACTCATGGTTTGAA..................................................... 20

.......................CTCGACTCATGGTTTGAAC.................................................... 1

.......................CTCGACTCATGGTTTGAACCA.................................................. 2

........................TCGACTCATGGTTTGAACCA.................................................. 10

........................TCGACTCATGGTTTGAAC.................................................... 3

........................TCGACTCATGGTTTGAACC................................................... 8

.........................CGACTCATGGTTTGAACC................................................... 2

.........................CGACTCATGGTTTGAACCA.................................................. 4

..........................GACTCATGGTTTGAACCA.................................................. 4

..........................GACTCATGGTTTGAACC................................................... 6

...........................ACTCATGGTTTGAACCA.................................................. 25

...........................ACTCATGGTTTGAACC................................................... 11

............................CTCATGGTTTGAACCAT................................................. 2

............................CTCATGGTTTGAACCA.................................................. 120

..........................................CATTACTTAATTCGTGG................................... 1

...........................................ATTACTTAATTCGTGG................................... 194

..........................................................GTTTGAACCATCACTCGACTCCT............. 8

..........................................................GTTTGAACCATCACTCGACTCC.............. 84

..........................................................GTTTGAACCATCACTCGAC................. 1

..........................................................GTTTGAACCATCACTCGACTC............... 11

..........................................................GTTTGAACCATCACTC.................... 3

..........................................................GTTTGAACCATCACTCGA.................. 1

..........................................................GTTTGAACCATCACTCGACT................ 3

...........................................................TTTGAACCATCACTCG................... 33

...........................................................TTTGAACCATCACTCGACTCCT............. 1990

...........................................................TTTGAACCATCACTCGAC................. 36

...........................................................TTTGAACCATCACTCGACTCCTGG........... 1

...........................................................TTTGAACCATCACTCGA.................. 394

...........................................................TTTGAACCATCACTCGACTCC.............. 1203

...........................................................TTTGAACCATCACTCGACT................ 43

...........................................................TTTGAACCATCACTCGACTCCTG............ 1

...........................................................TTTGAACCATCACTCGACTC............... 84

............................................................TTGAACCATCACTCGACTCC.............. 3

............................................................TTGAACCATCACTCGACTCCT............. 4

..............................................................GAACCATCACTCGACTCC.............. 2

..............................................................GAACCATCACTCGACTC............... 1

...............................................................AACCATCACTCGACTCC.............. 3

...............................................................AACCATCACTCGACTCCT............. 4

................................................................ACCATCACTCGACTCCT............. 52

................................................................ACCATCACTCGACTCC.............. 19

.................................................................CCATCACTCGACTCCT............. 5

>mmu-mir-669a-1_MI0004523_Mus_musculus_mir-669a-1_stem-loop ESC wild type

UGUAUGUGCAUGUGUGUAUAGUUGUGUGUGCAUGUUCAUGUCUAUAUUUGAAUAUACAUAACAUACACACACACGUAUAAACGCAAGCACACAUACA

.((((((((.(((((.((((..((((((((.(((((.((((.(((......))).))))))))).))))))))..)))).))))).)))))..))). (-36.80)

TGTATGTGCATGTGTGT................................................................................ 1

TGTATGTGCATGTGTGTAT.............................................................................. 6

TGTATGTGCATGTGTGTA............................................................................... 2

.GTATGTGCATGTGTGTAT.............................................................................. 2

..TATGTGCATGTGTGTAT.............................................................................. 7

...ATGTGCATGTGTGTATA............................................................................. 4

...ATGTGCATGTGTGTATAGTTGT........................................................................ 2

...ATGTGCATGTGTGTATAGTTGTGTG..................................................................... 1

...ATGTGCATGTGTGTATAGTTGTGT...................................................................... 7

...ATGTGCATGTGTGTAT.............................................................................. 2

....TGTGCATGTGTGTATAGTTGTGT...................................................................... 37

....TGTGCATGTGTGTATAGTTGTG....................................................................... 6

....TGTGCATGTGTGTATAG............................................................................ 1

....TGTGCATGTGTGTATA............................................................................. 11

....TGTGCATGTGTGTATAGTTGTGTG..................................................................... 13

....TGTGCATGTGTGTATAGT........................................................................... 7

....TGTGCATGTGTGTATA............................................................................. 3

....TGTGCATGTGTGTATAGTTGTGTGT.................................................................... 1

....TGTGCATGTGTGTATAGTTGT........................................................................ 1

.....GTGCATGTGTGTATAGTTGTGT...................................................................... 3

.....GTGCATGTGTGTATAGTTGTGTG..................................................................... 2

......TGCATGTGTGTATAGTT.......................................................................... 1

......TGCATGTGTGTATAGT........................................................................... 1

........CATGTGTGTATAGTTGTG....................................................................... 1

.........ATGTGTGTATAGTTGTGTGT.................................................................... 1

.........ATGTGTGTATAGTTGTGTG..................................................................... 4

.........ATGTGTGTATAGTTGTG....................................................................... 1

.........ATGTGTGTATAGTTGTGT...................................................................... 6

.........ATGTGTGTATAGTTGT........................................................................ 1

..........TGTGTGTATAGTTGTGT...................................................................... 25

..........TGTGTGTATAGTTGTG....................................................................... 2

..........TGTGTGTATAGTTGTGTG..................................................................... 4

...........GTGTGTATAGTTGTGTG..................................................................... 3

...........GTGTGTATAGTTGTGT...................................................................... 1

.................ATAGTTGTGTGTGCATGT.............................................................. 1

.................ATAGTTGTGTGTGCATGTTCAT.......................................................... 1

.................ATAGTTGTGTGTGCATGTTCATGT........................................................ 6

..................TAGTTGTGTGTGCATGTTCAT.......................................................... 3

..................TAGTTGTGTGTGCATGTTCATG......................................................... 23

..................TAGTTGTGTGTGCATG............................................................... 60

..................TAGTTGTGTGTGCATGTT............................................................. 12

..................TAGTTGTGTGTGCATGTTC............................................................ 1

..................TAGTTGTGTGTGCATGTTCATGTCT...................................................... 5

..................TAGTTGTGTGTGCATGTTCATGTC....................................................... 72

..................TAGTTGTGTGTGCATGTTCA........................................................... 1

..................TAGTTGTGTGTGCATGTTCATGT........................................................ 271

..................TAGTTGTGTGTGCATGT.............................................................. 65

...................AGTTGTGTGTGCATGTTCATG......................................................... 405

...................AGTTGTGTGTGCATGTTCATGTCTA..................................................... 18

...................AGTTGTGTGTGCATGTTCATGTCTATA................................................... 2

...................AGTTGTGTGTGCATGTT............................................................. 66

...................AGTTGTGTGTGCATGT.............................................................. 260

...................AGTTGTGTGTGCATGTTC............................................................ 55

...................AGTTGTGTGTGCATGTTCAT.......................................................... 216

...................AGTTGTGTGTGCATGTTCA........................................................... 17

...................AGTTGTGTGTGCATGTTCATGTCT...................................................... 3251

...................AGTTGTGTGTGCATGTTCATGTC....................................................... 3663

...................AGTTGTGTGTGCATGTTCATGT........................................................ 3587

....................GTTGTGTGTGCATGTTCATGTCT...................................................... 3

....................GTTGTGTGTGCATGTTCATGTC....................................................... 9

....................GTTGTGTGTGCATGTTCATGT........................................................ 5

....................GTTGTGTGTGCATGTTCAT.......................................................... 1

.....................TTGTGTGTGCATGTTCAT.......................................................... 1

.....................TTGTGTGTGCATGTTCATGT........................................................ 4

.....................TTGTGTGTGCATGTTCATGTCT...................................................... 3

.....................TTGTGTGTGCATGTTCATGTC....................................................... 6

......................TGTGTGTGCATGTTCATGTCT...................................................... 7

......................TGTGTGTGCATGTTCATG......................................................... 1

......................TGTGTGTGCATGTTCATGT........................................................ 8

......................TGTGTGTGCATGTTCATGTC....................................................... 14

......................TGTGTGTGCATGTTCAT.......................................................... 2

.......................GTGTGTGCATGTTCATGTCT...................................................... 9

.......................GTGTGTGCATGTTCATGT........................................................ 5

.......................GTGTGTGCATGTTCAT.......................................................... 4

.......................GTGTGTGCATGTTCATG......................................................... 3

.......................GTGTGTGCATGTTCATGTC....................................................... 15

........................TGTGTGCATGTTCATGT........................................................ 2

........................TGTGTGCATGTTCATGTC....................................................... 8

.........................GTGTGCATGTTCATGTCT...................................................... 7

.........................GTGTGCATGTTCATGT........................................................ 22

.........................GTGTGCATGTTCATGTC....................................................... 23

.........................GTGTGCATGTTCATGTCTAT.................................................... 1

..........................TGTGCATGTTCATGTC....................................................... 10

..........................TGTGCATGTTCATGTCT...................................................... 3

...........................GTGCATGTTCATGTCT...................................................... 14

...........................GTGCATGTTCATGTCTA..................................................... 1

......................................................TACATAACATACACACACACG...................... 1

......................................................TACATAACATACACACACAC....................... 1

......................................................TACATAACATACACACACA........................ 1

......................................................TACATAACATACACACACACGTAT................... 7

......................................................TACATAACATACACACACACGT..................... 79

......................................................TACATAACATACACACAC......................... 1

......................................................TACATAACATACACAC........................... 1

......................................................TACATAACATACACACACACGTA.................... 11

......................................................TACATAACATACACACA.......................... 1

.......................................................ACATAACATACACACACACGT..................... 1637

.......................................................ACATAACATACACACA.......................... 89

.......................................................ACATAACATACACACACACGTA.................... 2944

.......................................................ACATAACATACACACAC......................... 123

.......................................................ACATAACATACACACACA........................ 164

.......................................................ACATAACATACACACACACG...................... 68

.......................................................ACATAACATACACACACACGTATA.................. 20

.......................................................ACATAACATACACACACAC....................... 96

.......................................................ACATAACATACACACACACGTAT................... 5828

........................................................CATAACATACACACACACGTAT................... 610

........................................................CATAACATACACACACACGT..................... 34

........................................................CATAACATACACACAC......................... 13

........................................................CATAACATACACACACACGTA.................... 64

........................................................CATAACATACACACACACGTATA.................. 2

........................................................CATAACATACACACACACG...................... 3

........................................................CATAACATACACACACA........................ 15

........................................................CATAACATACACACACAC....................... 14

.........................................................ATAACATACACACACACGTA.................... 28

.........................................................ATAACATACACACACACGT..................... 22

.........................................................ATAACATACACACACA........................ 5

.........................................................ATAACATACACACACAC....................... 9

.........................................................ATAACATACACACACACG...................... 1

.........................................................ATAACATACACACACACGTAT................... 127

.........................................................ATAACATACACACACACGTATA.................. 29

..........................................................TAACATACACACACACGTATAA................. 1

..........................................................TAACATACACACACACGT..................... 20

..........................................................TAACATACACACACACGTATA.................. 6

..........................................................TAACATACACACACACGTA.................... 125

..........................................................TAACATACACACACAC....................... 3

..........................................................TAACATACACACACACGTAT................... 467

..........................................................TAACATACACACACACG...................... 3

...........................................................AACATACACACACACGTA.................... 128

...........................................................AACATACACACACACGT..................... 11

...........................................................AACATACACACACACG...................... 2

...........................................................AACATACACACACACGTAT................... 293

............................................................ACATACACACACACGT..................... 3

............................................................ACATACACACACACGTAT................... 76

............................................................ACATACACACACACGTA.................... 12

............................................................ACATACACACACACGTATA.................. 2

.............................................................CATACACACACACGTA.................... 19

.............................................................CATACACACACACGTAT................... 163

.............................................................CATACACACACACGTATA.................. 2

..............................................................ATACACACACACGTATA.................. 5

..............................................................ATACACACACACGTAT................... 191

...............................................................TACACACACACGTATA.................. 4

..............................................................................AAACGCAAGCACACATA.. 1

>mmu-mir-674_MI0004611_Mus_musculus_mir-674_stem-loop ESC wild type

GGCCUAGUCAUCACCCUGAGCCUUGCACUGAGAUGGGAGUGGUGUAAGGCUCAGGUAUGCACAGCUCCCAUCUCAGAACAAGGCUCGGGUGUGCUCAGCU

(((..((.((.(((((.((((((((..(((((((((((((.(((((..((....)).))))).)))))))))))))..)))))))))))))))))..))) (-60.40)

.GCCTAGTCATCACCCTGAGCCTT............................................................................ 1

...CTAGTCATCACCCTGAGCCTT............................................................................ 4

....TAGTCATCACCCTGAGCCTT............................................................................ 12

....TAGTCATCACCCTGAG................................................................................ 1

.....AGTCATCACCCTGAGC............................................................................... 2

.....AGTCATCACCCTGAGCCTT............................................................................ 2

......GTCATCACCCTGAGCCTT............................................................................ 5

.......TCATCACCCTGAGCCTT............................................................................ 1

........CATCACCCTGAGCCTT............................................................................ 1

........CATCACCCTGAGCCTTG........................................................................... 2

.......................TGCACTGAGATGGGAGTGGTG........................................................ 7

.......................TGCACTGAGATGGGAGTGGTGTA...................................................... 7

.......................TGCACTGAGATGGGAGTGGT......................................................... 2

.......................TGCACTGAGATGGGAGT............................................................ 2

.......................TGCACTGAGATGGGAGTGGTGT....................................................... 89

.......................TGCACTGAGATGGGAGTGGTGTAA..................................................... 3

.......................TGCACTGAGATGGGAGTGG.......................................................... 4

.......................TGCACTGAGATGGGAG............................................................. 1

........................GCACTGAGATGGGAGTGGT......................................................... 7

........................GCACTGAGATGGGAGTG........................................................... 4

........................GCACTGAGATGGGAGTGG.......................................................... 41

........................GCACTGAGATGGGAGTGGTGTAA..................................................... 168

........................GCACTGAGATGGGAGT............................................................ 5

........................GCACTGAGATGGGAGTGGTGT....................................................... 246

........................GCACTGAGATGGGAGTGGTGTA...................................................... 158

........................GCACTGAGATGGGAGTGGTGTAAG.................................................... 2

........................GCACTGAGATGGGAGTGGTG........................................................ 8

.........................CACTGAGATGGGAGTGGTGTAA..................................................... 10

.........................CACTGAGATGGGAGTGG.......................................................... 3

.........................CACTGAGATGGGAGTGGTGTA...................................................... 8

.........................CACTGAGATGGGAGTGGTGT....................................................... 6

..........................ACTGAGATGGGAGTGGTGTAA..................................................... 19

..........................ACTGAGATGGGAGTGGTGTA...................................................... 10

..........................ACTGAGATGGGAGTGGTGT....................................................... 13

..........................ACTGAGATGGGAGTGG.......................................................... 1

..........................ACTGAGATGGGAGTGGTG........................................................ 2

...........................CTGAGATGGGAGTGGTGTA...................................................... 10

...........................CTGAGATGGGAGTGGTG........................................................ 2

...........................CTGAGATGGGAGTGGTGTAA..................................................... 4

...........................CTGAGATGGGAGTGGT......................................................... 1

...........................CTGAGATGGGAGTGGTGT....................................................... 6

............................TGAGATGGGAGTGGTGT....................................................... 1

............................TGAGATGGGAGTGGTGTA...................................................... 1

.............................GAGATGGGAGTGGTGT....................................................... 3

.............................GAGATGGGAGTGGTGTA...................................................... 3

..............................AGATGGGAGTGGTGTAA..................................................... 6

..............................AGATGGGAGTGGTGTA...................................................... 7

...............................GATGGGAGTGGTGTAA..................................................... 4

...........................................................CACAGCTCCCATCTCAGA....................... 115

...........................................................CACAGCTCCCATCTCAGAACAAG.................. 3

...........................................................CACAGCTCCCATCTCAGAAC..................... 51

...........................................................CACAGCTCCCATCTCA......................... 4

...........................................................CACAGCTCCCATCTCAGAACA.................... 122

...........................................................CACAGCTCCCATCTCAGAACAA................... 209

...........................................................CACAGCTCCCATCTCAGAA...................... 19

...........................................................CACAGCTCCCATCTCAG........................ 25

............................................................ACAGCTCCCATCTCAGAACA.................... 1

............................................................ACAGCTCCCATCTCAGAACAA................... 8

............................................................ACAGCTCCCATCTCAGAACAAG.................. 1

............................................................ACAGCTCCCATCTCAGA....................... 1

.............................................................CAGCTCCCATCTCAGAACAAGG................. 2

.............................................................CAGCTCCCATCTCAGAAC..................... 2

..............................................................AGCTCCCATCTCAGAACA.................... 1

..............................................................AGCTCCCATCTCAGAACAA................... 1

..............................................................AGCTCCCATCTCAGAAC..................... 1

.................................................................TCCCATCTCAGAACAA................... 1

>mmu-mir-685_MI0004649_Mus_musculus_miR-685_stem-loop ESC wild type

GUCGGGGCGGAUGCCUCCCUCGCCGGAGCGGAUGCCUCCCUCGCCGGAGCUUGGAACAGACUCACGGCCAGCAGUGCGAGUUCAAUGGCUGAGGUGAGGCACCUCCCGG

..(((((.((.(((((((((((((.((((...(((((..((.(((((((((......)).))).)))).)).)).))).))))...))).)))).))))))))))))). (-53.00)

GTCGGGGCGGATGCCTCCCTCGC...................................................................................... 1

.........GATGCCTCCCTCGCCGGAGCGGA............................................................................. 1

.........GATGCCTCCCTCGCCGGAG................................................................................. 2

.........GATGCCTCCCTCGCCGGAGC................................................................................ 1

..............................GATGCCTCCCTCGCCGGAG............................................................ 2

..............................GATGCCTCCCTCGCCGGAGC........................................................... 1

..............................GATGCCTCCCTCGCCGGAGCTT......................................................... 1

................................................GCTTGGAACAGACTCACGGCCAGC..................................... 1

..................................................TTGGAACAGACTCACGG.......................................... 1

..........................................................GACTCACGGCCAGCAGTGCGAG............................. 1

............................................................CTCACGGCCAGCAGTGCGAG............................. 1

.............................................................................GAGTTCAATGGCTGAGGTGAG........... 1

..............................................................................AGTTCAATGGCTGAGGTGAGGC......... 1

...............................................................................GTTCAATGGCTGAGGTGAGG.......... 2

...............................................................................GTTCAATGGCTGAGGTGAGGCA........ 1

...............................................................................GTTCAATGGCTGAGGTGAGGC......... 1

...............................................................................GTTCAATGGCTGAGGT.............. 2

...............................................................................GTTCAATGGCTGAGGTGA............ 3

................................................................................TTCAATGGCTGAGGTGAGGC......... 2

................................................................................TTCAATGGCTGAGGTGA............ 1

................................................................................TTCAATGGCTGAGGTGAGGCA........ 3

................................................................................TTCAATGGCTGAGGTGAGGCACC...... 1

.................................................................................TCAATGGCTGAGGTGAGGCA........ 1

.................................................................................TCAATGGCTGAGGTGAG........... 1

..................................................................................CAATGGCTGAGGTGAGGCA........ 1

..................................................................................CAATGGCTGAGGTGAGGC......... 1

..................................................................................CAATGGCTGAGGTGAGG.......... 1

...................................................................................AATGGCTGAGGTGAGGCA........ 3

....................................................................................ATGGCTGAGGTGAGGCAC....... 1

....................................................................................ATGGCTGAGGTGAGGCA........ 1

.....................................................................................TGGCTGAGGTGAGGCA........ 2

>mmu-mir-669b_MI0004666_Mus_musculus_miR-669b_stem-loop ESC wild type

AUGAAUGUAUGUGCAUGUGUAUAUAGUUUUGUGUGCAUGUGCAUGUGUGUCUAUUAAUGUACAUAUACAUACACACAAACAUAUACACGCAUGCGCA

.........(((((((((((.((((..((((((((.(((((.(((((..(......)..))))).))))).))))))))..)))).))))))))))) (-39.80)

....ATGTATGTGCATGTGTATAT......................................................................... 1

....ATGTATGTGCATGTGT............................................................................. 2

.....TGTATGTGCATGTGTATAT......................................................................... 1

.......................TAGTTTTGTGTGCATGTGCATGT................................................... 1

........................AGTTTTGTGTGCATGTGCATGTGTG................................................ 1

........................AGTTTTGTGTGCATGT......................................................... 31

........................AGTTTTGTGTGCATGTGCATGT................................................... 1303

........................AGTTTTGTGTGCATGTGCAT..................................................... 171

........................AGTTTTGTGTGCATGTGC....................................................... 142

........................AGTTTTGTGTGCATGTGCATGTGTGT............................................... 3

........................AGTTTTGTGTGCATGTGCA...................................................... 280

........................AGTTTTGTGTGCATGTGCATGTG.................................................. 674

........................AGTTTTGTGTGCATGTGCATG.................................................... 593

........................AGTTTTGTGTGCATGTG........................................................ 17

........................AGTTTTGTGTGCATGTGCATGTGT................................................. 481

.........................GTTTTGTGTGCATGTGCATGT................................................... 5

.........................GTTTTGTGTGCATGTGCATGTG.................................................. 3

.........................GTTTTGTGTGCATGTGCAT..................................................... 1

.........................GTTTTGTGTGCATGTGCATGTGT................................................. 3

.........................GTTTTGTGTGCATGTGCATG.................................................... 1

..........................TTTTGTGTGCATGTGCATGTG.................................................. 1

..........................TTTTGTGTGCATGTGCATGTGT................................................. 1

..........................TTTTGTGTGCATGTGCA...................................................... 1

...........................TTTGTGTGCATGTGCATGTG.................................................. 10

...........................TTTGTGTGCATGTGCATGT................................................... 5

...........................TTTGTGTGCATGTGCAT..................................................... 1

...........................TTTGTGTGCATGTGCATGTGT................................................. 4

...........................TTTGTGTGCATGTGCA...................................................... 7

...........................TTTGTGTGCATGTGCATG.................................................... 4

............................TTGTGTGCATGTGCATG.................................................... 2

............................TTGTGTGCATGTGCATGTG.................................................. 3

............................TTGTGTGCATGTGCATGT................................................... 2

............................TTGTGTGCATGTGCATGTGT................................................. 1

.............................TGTGTGCATGTGCATGT................................................... 68

.............................TGTGTGCATGTGCATG.................................................... 57

.............................TGTGTGCATGTGCATGTGTG................................................ 3

.............................TGTGTGCATGTGCATGTGTGT............................................... 59

.............................TGTGTGCATGTGCATGTG.................................................. 18

.............................TGTGTGCATGTGCATGTGT................................................. 10

.............................TGTGTGCATGTGCATGTGTGTCT............................................. 5

..............................GTGTGCATGTGCATGTGT................................................. 7

..............................GTGTGCATGTGCATGTGTG................................................ 5

..............................GTGTGCATGTGCATGTGTGT............................................... 168

..............................GTGTGCATGTGCATGTGTGTCTAT........................................... 3

..............................GTGTGCATGTGCATGTGTGTCTA............................................ 1

..............................GTGTGCATGTGCATGT................................................... 66

..............................GTGTGCATGTGCATGTG.................................................. 2

...............................TGTGCATGTGCATGTG.................................................. 1

................................GTGCATGTGCATGTGT................................................. 4

.............................................TGTGTCTATTAATGTACA.................................. 1

...........................................................TACATATACATACACAC..................... 1

.............................................................CATATACATACACACAAACAT............... 13

.............................................................CATATACATACACACAAACATAT............. 35

.............................................................CATATACATACACACAAACATA.............. 4

.............................................................CATATACATACACACA.................... 5

.............................................................CATATACATACACACAAAC................. 3

.............................................................CATATACATACACACAA................... 2

..............................................................ATATACATACACACAAACATAT............. 53

..............................................................ATATACATACACACAAAC................. 8

..............................................................ATATACATACACACAAACAT............... 2

..............................................................ATATACATACACACAA................... 1

..............................................................ATATACATACACACAAACATA.............. 2

..............................................................ATATACATACACACAAA.................. 1

.................................................................TACATACACACAAACATAT............. 1

..................................................................ACATACACACAAACATAT............. 9

..................................................................ACATACACACAAACATA.............. 1

..................................................................ACATACACACAAACAT............... 1

...................................................................CATACACACAAACATA.............. 1

...................................................................CATACACACAAACATAT............. 16

....................................................................ATACACACAAACATAT............. 3

>mmu-mir-708_MI0004692_Mus_musculus_miR-708_stem-loop ESC wild type

CUGUGUUUGAAAUGGGGACUGCCCUCAAGGAGCUUACAAUCUAGCUGGGGGUAGAUGACUUGCACUUGAACACAACUAGACUGUGAGCUUCUAGAGGGCAGGGGCCUUA

............(((((.((((((((.(((((((((((.(((((.((.(..(((.((.....)).)))..).)).))))).))))))))))).))))))))...))))) (-48.50)

......TTGAAATGGGGACTGCCCTC................................................................................... 1

........GAAATGGGGACTGCCCTC................................................................................... 2

.........................CAAGGAGCTTACAATCTAGCTGG............................................................. 1

.........................CAAGGAGCTTACAATCTAGCTGGG............................................................ 1

..........................AAGGAGCTTACAATCTAG................................................................. 1

..........................AAGGAGCTTACAATCTAGCTGGG............................................................ 1334

..........................AAGGAGCTTACAATCT................................................................... 10

..........................AAGGAGCTTACAATCTAGCTG.............................................................. 114

..........................AAGGAGCTTACAATCTAGCTGGGG........................................................... 2

..........................AAGGAGCTTACAATCTA.................................................................. 8

..........................AAGGAGCTTACAATCTAGC................................................................ 5

..........................AAGGAGCTTACAATCTAGCTGG............................................................. 662

..........................AAGGAGCTTACAATCTAGCT............................................................... 29

...........................AGGAGCTTACAATCTAGCTGG............................................................. 9

...........................AGGAGCTTACAATCTAGCT............................................................... 1

...........................AGGAGCTTACAATCTAGCTGGGG........................................................... 1

...........................AGGAGCTTACAATCTAGCTGGG............................................................ 32

...........................AGGAGCTTACAATCTAGCTG.............................................................. 3

............................GGAGCTTACAATCTAGCTGG............................................................. 112

............................GGAGCTTACAATCTAGCTGGG............................................................ 139

............................GGAGCTTACAATCTAGCTGGGG........................................................... 1

............................GGAGCTTACAATCTAGCTG.............................................................. 6

.............................GAGCTTACAATCTAGCTG.............................................................. 2

.............................GAGCTTACAATCTAGCTGGG............................................................ 138

.............................GAGCTTACAATCTAGCTGG............................................................. 111

.............................GAGCTTACAATCTAGCT............................................................... 1

..............................AGCTTACAATCTAGCTGGG............................................................ 8

..............................AGCTTACAATCTAGCTGG............................................................. 3

..............................AGCTTACAATCTAGCT............................................................... 1

...............................GCTTACAATCTAGCTGGG............................................................ 14

...............................GCTTACAATCTAGCTGG............................................................. 11

................................CTTACAATCTAGCTGG............................................................. 3

................................CTTACAATCTAGCTGGG............................................................ 4

.................................TTACAATCTAGCTGGG............................................................ 32

...............................................GGGGTAGATGACTTGC.............................................. 1

.................................................GGTAGATGACTTGCACTTGAACA..................................... 8

.................................................GGTAGATGACTTGCACTTGAAC...................................... 1

.....................................................GATGACTTGCACTTGAACA..................................... 2

.....................................................GATGACTTGCACTTGA........................................ 1

.......................................................TGACTTGCACTTGAACA..................................... 3

........................................................................CAACTAGACTGTGAGCTTCT................. 12

........................................................................CAACTAGACTGTGAGCTTCTAGA.............. 75

........................................................................CAACTAGACTGTGAGCTTCTAG............... 100

........................................................................CAACTAGACTGTGAGCTTC.................. 3

........................................................................CAACTAGACTGTGAGCTTCTA................ 4

........................................................................CAACTAGACTGTGAGCTT................... 1

.........................................................................AACTAGACTGTGAGCTTCTAGA.............. 40

.........................................................................AACTAGACTGTGAGCTTCTAG............... 16

.........................................................................AACTAGACTGTGAGCTTC.................. 1

.........................................................................AACTAGACTGTGAGCT.................... 1

.........................................................................AACTAGACTGTGAGCT.................... 1

..........................................................................ACTAGACTGTGAGCTTCTAGA.............. 13

..........................................................................ACTAGACTGTGAGCTTCT................. 1

..........................................................................ACTAGACTGTGAGCTTCTAG............... 12

...........................................................................CTAGACTGTGAGCTTCTAG............... 1

...............................................................................ACTGTGAGCTTCTAGA.............. 3

>mmu-mir-466l_MI0006278_Mus_musculus_miR-466l_stem-loop ESC wild type

CUAUGUAUGUGCAUGUGUGUUUGUGUGUACAUGUACAUGUAUAUAUAUAUUGAUAUACAUAUAAAUACAUGCACACAUAUUCAUGCAUGCACACGCACACAUUAAUGGCACUCUUAGAUCC

...((..((((((((((((..(((((((.((((((..(((((.(((((....))))).)))))..)))))).)))))))..))))))))))))..))........................ (-43.60)

CTATGTATGTGCATGTGTGTT.................................................................................................... 1

.TATGTATGTGCATGTGTGTT.................................................................................................... 2

..ATGTATGTGCATGTGTG...................................................................................................... 3

..ATGTATGTGCATGTGTGT..................................................................................................... 5

..ATGTATGTGCATGTGTGTT.................................................................................................... 14

..ATGTATGTGCATGTGT....................................................................................................... 2

...TGTATGTGCATGTGTGT..................................................................................................... 1

...TGTATGTGCATGTGTGTT.................................................................................................... 7

...TGTATGTGCATGTGTGTTTG.................................................................................................. 1

...TGTATGTGCATGTGTGTTT................................................................................................... 3

....................TTGTGTGTACATGTACAT................................................................................... 1

....................TTGTGTGTACATGTACATGTATATA............................................................................ 2

....................TTGTGTGTACATGTACATGTATAT............................................................................. 6

....................TTGTGTGTACATGTACATGT................................................................................. 3

....................TTGTGTGTACATGTACATGTAT............................................................................... 20

....................TTGTGTGTACATGTACATG.................................................................................. 2

....................TTGTGTGTACATGTACATGTATA.............................................................................. 15

....................TTGTGTGTACATGTAC..................................................................................... 1

.....................TGTGTGTACATGTACAT................................................................................... 6

.....................TGTGTGTACATGTACA.................................................................................... 2

.....................TGTGTGTACATGTACATGTATA.............................................................................. 11

.....................TGTGTGTACATGTACATGT................................................................................. 1

.....................TGTGTGTACATGTACATGTAT............................................................................... 2

......................GTGTGTACATGTACAT................................................................................... 15

......................GTGTGTACATGTACATGT................................................................................. 10

......................GTGTGTACATGTACATG.................................................................................. 8

.......................TGTGTACATGTACATG.................................................................................. 6

.......................TGTGTACATGTACATGT................................................................................. 2

........................GTGTACATGTACATGT................................................................................. 1

..........................GTACATGTACATGTAT............................................................................... 1

.........................................................CATATAAATACATGCACACATA.......................................... 2

.........................................................CATATAAATACATGCACACAT........................................... 1

...........................................................TATAAATACATGCACACA............................................ 2

...........................................................TATAAATACATGCACACATATTC....................................... 16

...........................................................TATAAATACATGCACACATA.......................................... 2

...........................................................TATAAATACATGCACACATAT......................................... 5

...........................................................TATAAATACATGCACACATATT........................................ 36

...........................................................TATAAATACATGCACAC............................................. 3

...........................................................TATAAATACATGCACACAT........................................... 10

............................................................ATAAATACATGCACAC............................................. 1

............................................................ATAAATACATGCACACAT........................................... 2

............................................................ATAAATACATGCACACATATT........................................ 1

............................................................ATAAATACATGCACACATATTC....................................... 10

.............................................................TAAATACATGCACACATATTC....................................... 8

.............................................................TAAATACATGCACACATATTCA...................................... 7

.............................................................TAAATACATGCACACATA.......................................... 1

.............................................................TAAATACATGCACACAT........................................... 2

................................................................ATACATGCACACATATT........................................ 3

>mmu-mir-669e_MI0006300_Mus_musculus_miR-669e_stem-loop ESC wild type

GCCUGGGAUUCAUGGGCUGUACCCUAUAUAUGGGCAGGUGUGUGUCUUGUGUGUGCAUGUUCAUUUGUGUAUAUGAAUAUGAAUAUACACACACUUACACACUCAUGCACACACACACA

(((((........(((.....)))......)))))..((((((((...(((((((..(((......((((((((........)))))))))))..)))))))....))))))))..... (-40.04)

......................CCTATATATGGGCAGGTGTG............................................................................. 1

.......................CTATATATGGGCAGGTGTG............................................................................. 2

..........................................TGTCTTGTGTGTGCATGTTCATTT..................................................... 3

..........................................TGTCTTGTGTGTGCATGTTCAT....................................................... 240

..........................................TGTCTTGTGTGTGCAT............................................................. 66

..........................................TGTCTTGTGTGTGCATGTT.......................................................... 4

..........................................TGTCTTGTGTGTGCATGTTCA........................................................ 5

..........................................TGTCTTGTGTGTGCATGT........................................................... 20

..........................................TGTCTTGTGTGTGCATGTTCATT...................................................... 391

..........................................TGTCTTGTGTGTGCATG............................................................ 15

..........................................TGTCTTGTGTGTGCATGTTC......................................................... 1

...........................................GTCTTGTGTGTGCATGTTCATT...................................................... 1

...........................................GTCTTGTGTGTGCATGTTCAT....................................................... 1

...........................................GTCTTGTGTGTGCATGT........................................................... 1

............................................TCTTGTGTGTGCATGTTCATT...................................................... 1

............................................TCTTGTGTGTGCATGTTCAT....................................................... 1

.............................................CTTGTGTGTGCATGTTCATT...................................................... 1

..............................................TTGTGTGTGCATGTTCAT....................................................... 1

..............................................TTGTGTGTGCATGTTCATT...................................................... 1

...............................................TGTGTGTGCATGTTCATT...................................................... 1

...............................................TGTGTGTGCATGTTCAT....................................................... 2

................................................GTGTGTGCATGTTCAT....................................................... 4

.............................................................................TATGAATATACACACACTTAC..................... 1

.............................................................................TATGAATATACACACAC......................... 6

.............................................................................TATGAATATACACACACTTACAC................... 1

.............................................................................TATGAATATACACACACTTACA.................... 2

..............................................................................ATGAATATACACACAC......................... 3

...............................................................................TGAATATACACACACTTACACA.................. 40

...............................................................................TGAATATACACACACTT....................... 3

...............................................................................TGAATATACACACACTTACAC................... 48

...............................................................................TGAATATACACACACTTAC..................... 4

...............................................................................TGAATATACACACACT........................ 4

...............................................................................TGAATATACACACACTTACA.................... 10

................................................................................GAATATACACACACTTACAC................... 1

................................................................................GAATATACACACACTT....................... 1

..................................................................................ATATACACACACTTACACA.................. 1

..................................................................................ATATACACACACTTACA.................... 1

...................................................................................TATACACACACTTACAC................... 1

....................................................................................ATACACACACTTACAC................... 1

>mmu-mir-1199_MI0006307_Mus_musculus_miR-1199_stem-loop ESC wild type

AGCCUGCGCCGGAGCCGGGGUCUGAGUCCCGGUCGCGCGGGCGAGGAACUCAUUGAGUUGCGCGUGCGGCCGGUGCUCAGUCGGCCCGGCUCCGGUACUCCGCUGCCGCGCGCCCUGGA

..((.(..((((((((((((.(((((..(((((((((((.(((....((((...))))))).)))))))))))..))))).)..)))))))))))..)..(((......)))....)). (-72.50)

...................GTCTGAGTCCCGGTCGCGCGGG.............................................................................. 2

....................TCTGAGTCCCGGTCGCGCGGGCGA........................................................................... 1

....................TCTGAGTCCCGGTCGCGCGGGC............................................................................. 2

....................TCTGAGTCCCGGTCGCGCGG............................................................................... 4

....................TCTGAGTCCCGGTCGCGCGGG.............................................................................. 2

..............................................................CGTGCGGCCGGTGCTCAGTCG.................................... 1

................................................................TGCGGCCGGTGCTCAGTCGG................................... 1

................................................................TGCGGCCGGTGCTCAGTCGGC.................................. 1

.....................................................................................CCGGCTCCGGTACTCCGCTGC............. 1

>mmu-mir-669m-1_MI0009943_Mus_musculus_miR-669m_stem-loop ESC wild type

UGUAUGUGCAUGUGUGUAUAGUUUUGUGUGCAUGUGCAUGUGUGUAUAUGAAUAGACAUAUACAUCCACACAAACAUAUACAAGCAAGCACAGAUACA

.((((((((.(((.((((((..((((((((.(((((.((((.(((......))).)))).))))).))))))))..)))))).))).)))))..))). (-38.20)

TGTATGTGCATGTGTGT................................................................................. 1

TGTATGTGCATGTGTGTAT............................................................................... 6

TGTATGTGCATGTGTGTA................................................................................ 2

.GTATGTGCATGTGTGTAT............................................................................... 2

..TATGTGCATGTGTGTAT............................................................................... 7

...ATGTGCATGTGTGTATA.............................................................................. 4

...ATGTGCATGTGTGTAT............................................................................... 2

....TGTGCATGTGTGTATAG............................................................................. 1

....TGTGCATGTGTGTATA.............................................................................. 11

....TGTGCATGTGTGTATAGT............................................................................ 7

....TGTGCATGTGTGTATA.............................................................................. 3

....TGTGCATGTGTGTATAGTTTTGT....................................................................... 1

......TGCATGTGTGTATAGTT........................................................................... 1

......TGCATGTGTGTATAGT............................................................................ 1

..................TAGTTTTGTGTGCATGTGCATGT......................................................... 1

...................AGTTTTGTGTGCATGTGCATGTGTG...................................................... 1

...................AGTTTTGTGTGCATGT............................................................... 31

...................AGTTTTGTGTGCATGTGCATGT......................................................... 1303

...................AGTTTTGTGTGCATGTGCAT........................................................... 171

...................AGTTTTGTGTGCATGTGC............................................................. 142

...................AGTTTTGTGTGCATGTGCATGTGTGT..................................................... 3

...................AGTTTTGTGTGCATGTGCA............................................................ 280

...................AGTTTTGTGTGCATGTGCATGTG........................................................ 674

...................AGTTTTGTGTGCATGTGCATG.......................................................... 593

...................AGTTTTGTGTGCATGTG.............................................................. 17

...................AGTTTTGTGTGCATGTGCATGTGT....................................................... 481

....................GTTTTGTGTGCATGTGCATGT......................................................... 5

....................GTTTTGTGTGCATGTGCATGTG........................................................ 3

....................GTTTTGTGTGCATGTGCAT........................................................... 1

....................GTTTTGTGTGCATGTGCATGTGT....................................................... 3

....................GTTTTGTGTGCATGTGCATG.......................................................... 1

.....................TTTTGTGTGCATGTGCATGTG........................................................ 1

.....................TTTTGTGTGCATGTGCATGTGT....................................................... 1

.....................TTTTGTGTGCATGTGCA............................................................ 1

......................TTTGTGTGCATGTGCATGTG........................................................ 10

......................TTTGTGTGCATGTGCATGT......................................................... 5

......................TTTGTGTGCATGTGCAT........................................................... 1

......................TTTGTGTGCATGTGCATGTGT....................................................... 4

......................TTTGTGTGCATGTGCA............................................................ 7

......................TTTGTGTGCATGTGCATG.......................................................... 4

.......................TTGTGTGCATGTGCATG.......................................................... 2

.......................TTGTGTGCATGTGCATGTG........................................................ 3

.......................TTGTGTGCATGTGCATGT......................................................... 2

.......................TTGTGTGCATGTGCATGTGT....................................................... 1

........................TGTGTGCATGTGCATGTGTGTATAT................................................. 20

........................TGTGTGCATGTGCATGTGTGTA.................................................... 20

........................TGTGTGCATGTGCATGT......................................................... 68

........................TGTGTGCATGTGCATG.......................................................... 57

........................TGTGTGCATGTGCATGTGTG...................................................... 3

........................TGTGTGCATGTGCATGTGTGT..................................................... 59

........................TGTGTGCATGTGCATGTG........................................................ 18

........................TGTGTGCATGTGCATGTGTGTATA.................................................. 87

........................TGTGTGCATGTGCATGTGT....................................................... 10

........................TGTGTGCATGTGCATGTGTGTAT................................................... 409

.........................GTGTGCATGTGCATGTGT....................................................... 7

.........................GTGTGCATGTGCATGTGTGTAT................................................... 71

.........................GTGTGCATGTGCATGTGTGTATATGA............................................... 1

.........................GTGTGCATGTGCATGTGTGTATAT................................................. 102

.........................GTGTGCATGTGCATGTGTG...................................................... 5

.........................GTGTGCATGTGCATGTGTGT..................................................... 168

.........................GTGTGCATGTGCATGTGTGTA.................................................... 12

.........................GTGTGCATGTGCATGT......................................................... 66

.........................GTGTGCATGTGCATGTGTGTATA.................................................. 30

.........................GTGTGCATGTGCATGTG........................................................ 2

..........................TGTGCATGTGCATGTGTGTAT................................................... 2

..........................TGTGCATGTGCATGTG........................................................ 1

...........................GTGCATGTGCATGTGTGTATAT................................................. 1

...........................GTGCATGTGCATGTGTGTAT................................................... 1

...........................GTGCATGTGCATGTGT....................................................... 4

............................TGCATGTGCATGTGTGTATATGA............................................... 2

.............................GCATGTGCATGTGTGTATATGA............................................... 1

..............................CATGTGCATGTGTGTATAT................................................. 1

...............................ATGTGCATGTGTGTATA.................................................. 4

...............................ATGTGCATGTGTGTATAT................................................. 1

...............................ATGTGCATGTGTGTAT................................................... 2

................................TGTGCATGTGTGTATAT................................................. 6

................................TGTGCATGTGTGTATA.................................................. 11

................................TGTGCATGTGTGTATA.................................................. 3

.................................GTGCATGTGTGTATAT................................................. 1

.........................................GTGTATATGAATAGAC......................................... 2

.......................................................ACATATACATCCACACAAACATA.................... 1

........................................................CATATACATCCACACAAACATA.................... 3

........................................................CATATACATCCACACAAACA...................... 2

........................................................CATATACATCCACACAAAC....................... 5

........................................................CATATACATCCACACAA......................... 4

........................................................CATATACATCCACACAAACATAT................... 29

........................................................CATATACATCCACACA.......................... 1

........................................................CATATACATCCACACAAACAT..................... 12

........................................................CATATACATCCACACAAACATATA.................. 1

........................................................CATATACATCCACACAAA........................ 1

.........................................................ATATACATCCACACAAACATAT................... 8

.........................................................ATATACATCCACACAAACAT..................... 4

.........................................................ATATACATCCACACAA......................... 1

.........................................................ATATACATCCACACAAAC....................... 7

.........................................................ATATACATCCACACAAA........................ 2

..........................................................TATACATCCACACAAAC....................... 1

.............................................................ACATCCACACAAACATAT................... 2

.............................................................ACATCCACACAAACAT..................... 1

..............................................................CATCCACACAAACATAT................... 6

>mmu-mir-669m-2_MI0009944_Mus_musculus_miR-669n_stem-loop ESC wild type

AUAUUUGCAUGUGUGUAUAGUUUUGUGUGCAUGUGCAUGUGUGUAUAUGAAUAUACAUAUACAUCCACACAAACAUAUACAAGCAUGCACAGAUAUAC

((((((((((((.((((((..((((((((.(((((.((((((((......)))))))).))))).))))))))..)))))).)))))))..))))).. (-40.00)

ATATTTGCATGTGTGTAT................................................................................ 1

...TTTGCATGTGTGTATAGT............................................................................. 8

...TTTGCATGTGTGTATAGTTTTGT........................................................................ 1

...TTTGCATGTGTGTATAGTT............................................................................ 3

...TTTGCATGTGTGTATAG.............................................................................. 2

....TTGCATGTGTGTATAGTT............................................................................ 5

....TTGCATGTGTGTATAG.............................................................................. 2

....TTGCATGTGTGTATAGT............................................................................. 14

.....TGCATGTGTGTATAGTT............................................................................ 1

.....TGCATGTGTGTATAGT............................................................................. 1

.................TAGTTTTGTGTGCATGTGCATGT.......................................................... 1

..................AGTTTTGTGTGCATGTGCATGTGTG....................................................... 1

..................AGTTTTGTGTGCATGT................................................................ 31

..................AGTTTTGTGTGCATGTGCATGT.......................................................... 1303

..................AGTTTTGTGTGCATGTGCAT............................................................ 171

..................AGTTTTGTGTGCATGTGC.............................................................. 142

..................AGTTTTGTGTGCATGTGCATGTGTGT...................................................... 3

..................AGTTTTGTGTGCATGTGCA............................................................. 280

..................AGTTTTGTGTGCATGTGCATGTG......................................................... 674

..................AGTTTTGTGTGCATGTGCATG........................................................... 593

..................AGTTTTGTGTGCATGTG............................................................... 17

..................AGTTTTGTGTGCATGTGCATGTGT........................................................ 481

...................GTTTTGTGTGCATGTGCATGT.......................................................... 5

...................GTTTTGTGTGCATGTGCATGTG......................................................... 3

...................GTTTTGTGTGCATGTGCAT............................................................ 1

...................GTTTTGTGTGCATGTGCATGTGT........................................................ 3

...................GTTTTGTGTGCATGTGCATG........................................................... 1

....................TTTTGTGTGCATGTGCATGTG......................................................... 1

....................TTTTGTGTGCATGTGCATGTGT........................................................ 1

....................TTTTGTGTGCATGTGCA............................................................. 1

.....................TTTGTGTGCATGTGCATGTG......................................................... 10

.....................TTTGTGTGCATGTGCATGT.......................................................... 5

.....................TTTGTGTGCATGTGCAT............................................................ 1

.....................TTTGTGTGCATGTGCATGTGT........................................................ 4

.....................TTTGTGTGCATGTGCA............................................................. 7

.....................TTTGTGTGCATGTGCATG........................................................... 4

......................TTGTGTGCATGTGCATG........................................................... 2

......................TTGTGTGCATGTGCATGTG......................................................... 3

......................TTGTGTGCATGTGCATGT.......................................................... 2

......................TTGTGTGCATGTGCATGTGT........................................................ 1

.......................TGTGTGCATGTGCATGTGTGTATAT.................................................. 20

.......................TGTGTGCATGTGCATGTGTGTA..................................................... 20

.......................TGTGTGCATGTGCATGT.......................................................... 68

.......................TGTGTGCATGTGCATG........................................................... 57

.......................TGTGTGCATGTGCATGTGTG....................................................... 3

.......................TGTGTGCATGTGCATGTGTGT...................................................... 59

.......................TGTGTGCATGTGCATGTG......................................................... 18

.......................TGTGTGCATGTGCATGTGTGTATA................................................... 87

.......................TGTGTGCATGTGCATGTGT........................................................ 10

.......................TGTGTGCATGTGCATGTGTGTAT.................................................... 409

........................GTGTGCATGTGCATGTGT........................................................ 7

........................GTGTGCATGTGCATGTGTGTAT.................................................... 71

........................GTGTGCATGTGCATGTGTGTATATGA................................................ 1

........................GTGTGCATGTGCATGTGTGTATAT.................................................. 102

........................GTGTGCATGTGCATGTGTG....................................................... 5

........................GTGTGCATGTGCATGTGTGT...................................................... 168

........................GTGTGCATGTGCATGTGTGTA..................................................... 12

........................GTGTGCATGTGCATGT.......................................................... 66

........................GTGTGCATGTGCATGTGTGTATA................................................... 30

........................GTGTGCATGTGCATGTG......................................................... 2

.........................TGTGCATGTGCATGTGTGTAT.................................................... 2

.........................TGTGCATGTGCATGTG......................................................... 1

..........................GTGCATGTGCATGTGTGTATAT.................................................. 1

..........................GTGCATGTGCATGTGTGTAT.................................................... 1

..........................GTGCATGTGCATGTGT........................................................ 4

...........................TGCATGTGCATGTGTGTATATGA................................................ 2

............................GCATGTGCATGTGTGTATATGA................................................ 1

.............................CATGTGCATGTGTGTATAT.................................................. 1

..............................ATGTGCATGTGTGTATA................................................... 4

..............................ATGTGCATGTGTGTATAT.................................................. 1

..............................ATGTGCATGTGTGTAT.................................................... 2

...............................TGTGCATGTGTGTATAT.................................................. 6

...............................TGTGCATGTGTGTATA................................................... 11

...............................TGTGCATGTGTGTATA................................................... 3

................................GTGCATGTGTGTATAT.................................................. 1

.......................................TGTGTATATGAATATACA......................................... 6

........................................GTGTATATGAATATAC.......................................... 9

.....................................................TACATATACATCCACACAAAC........................ 1

......................................................ACATATACATCCACACAAACATA..................... 1

.......................................................CATATACATCCACACAAACATA..................... 3

.......................................................CATATACATCCACACAAACA....................... 2

.......................................................CATATACATCCACACAAAC........................ 5

.......................................................CATATACATCCACACAA.......................... 4

.......................................................CATATACATCCACACAAACATAT.................... 29

.......................................................CATATACATCCACACA........................... 1

.......................................................CATATACATCCACACAAACAT...................... 12

.......................................................CATATACATCCACACAAACATATA................... 1

.......................................................CATATACATCCACACAAA......................... 1

........................................................ATATACATCCACACAAACATAT.................... 8

........................................................ATATACATCCACACAAACAT...................... 4

........................................................ATATACATCCACACAA.......................... 1

........................................................ATATACATCCACACAAAC........................ 7

........................................................ATATACATCCACACAAA......................... 2

.........................................................TATACATCCACACAAAC........................ 1

............................................................ACATCCACACAAACATAT.................... 2

............................................................ACATCCACACAAACAT...................... 1

.............................................................CATCCACACAAACATAT.................... 6

>mmu-mir-30b_MI0000145_Mus_musculus_miR-30b_stem-loop ESC dcr

CUAAGCCAAGUUUCAGUUCAUGUAAACAUCCUACACUCAGCUGUCAUACAUGCGUUGGCUGGGAUGUGGAUGUUUACGUCAGCUGUCUUGGAGUAU

.((..(((((...(((((.((((((((((((.(((((((((((.(........).)))))))).))))))))))))))).))))).)))))..)). (-42.70)

CTAAGCCAAGTTTCAGTTCA............................................................................ 1

....................TGTAAACATCCTACACTCAGC....................................................... 1

....................TGTAAACATCCTACACT........................................................... 3

....................TGTAAACATCCTACACTCAGCTG..................................................... 1

....................TGTAAACATCCTACACTCAGCT...................................................... 1

....................TGTAAACATCCTACACTCAGCTGTCAT................................................. 4

....................TGTAAACATCCTACACTCAGCTGTCA.................................................. 1

....................TGTAAACATCCTACACTCAGCTGT.................................................... 5

....................TGTAAACATCCTACACTCAGCTGTC................................................... 5

........................AACATCCTACACTCAGCTGTC................................................... 1

.....................................................GTTGGCTGGGATGTGGATGTTT..................... 1

..........................................................CTGGGATGTGGATGTTTACGT................. 2

>mmu-mir-20a_MI0000568_Mus_musculus_miR-20a_stem-loop ESC dcr

GUGUGAUGUGACAGCUUCUGUAGCACUAAAGUGCUUAUAGUGCAGGUAGUGUGUAGCCAUCUACUGCAUUACGAGCACUUAAAGUACUGCCAGCUGUAGAACUCCAG

.((.((.((.((((((...((((.(((.(((((((..((((((((.(((.(((....))))))))))))))..)))))))..))).)))).))))))...)))))). (-41.00)

....GATGTGACAGCTTCTGTAGCAC................................................................................. 2

.....ATGTGACAGCTTCTGTAGCAC................................................................................. 2

.....ATGTGACAGCTTCTGTAGCA.................................................................................. 2

.....ATGTGACAGCTTCTGTAGC................................................................................... 1

..........................TAAAGTGCTTATAGTGCAG.............................................................. 1

..........................TAAAGTGCTTATAGTGCAGG............................................................. 3

..........................TAAAGTGCTTATAGTGCAGGTAGTGT....................................................... 3

..........................TAAAGTGCTTATAGTGCAGGTAGTG........................................................ 4

..........................TAAAGTGCTTATAGTGCAGGTA........................................................... 4

..........................TAAAGTGCTTATAGTGCAGGTAG.......................................................... 10

..........................TAAAGTGCTTATAGTGCAGGTAGTGTG...................................................... 1

..........................TAAAGTGCTTATAGTGC................................................................ 2

..........................TAAAGTGCTTATAGTGCAGGT............................................................ 8

..........................TAAAGTGCTTATAGTGCAGGTAGT......................................................... 14

..........................TAAAGTGCTTATAGTGCA............................................................... 16

...........................AAAGTGCTTATAGTGCAGG............................................................. 1

...........................AAAGTGCTTATAGTGCAGGTAGT......................................................... 1

..................................TTATAGTGCAGGTAGTGTGT..................................................... 1

..................................TTATAGTGCAGGTAGTGT....................................................... 3

................................................GTGTGTAGCCATCTACTGCATT..................................... 1

..................................................GTGTAGCCATCTACTGCATTACGAGC............................... 1

...................................................TGTAGCCATCTACTGCATTACGAGC............................... 1

.......................................................GCCATCTACTGCATTACGAGCACTT........................... 1

................................................................TGCATTACGAGCACTTAA......................... 1

>mmu-mir-24-2_MI0000572_Mus_musculus_miR-24-2_stem-loop ESC dcr

GCCUCUCUCCGGGCUCCGCCUCCCGUGCCUACUGAGCUGAAACAGUUGAUUCCAGUGCACUGGCUCAGUUCAGCAGGAACAGGAGUCCAGCCCCCUAGGAGCUGGCA

(((...(((((((((....((((.((.(((.(((((((((..((((..((....))..))))..))))))))).))).)).))))...)))))....))))..))). (-48.90)

....CTCTCCGGGCTCCGCCTCCC................................................................................... 5

.....TCTCCGGGCTCCGCCTCCC................................................................................... 1

......CTCCGGGCTCCGCCTCCC................................................................................... 1

........................GTGCCTACTGAGCTGAA.................................................................. 1

........................GTGCCTACTGAGCTGAAACAGT............................................................. 3

........................GTGCCTACTGAGCTGAAACAG.............................................................. 2

........................GTGCCTACTGAGCTGAAACA............................................................... 1

........................GTGCCTACTGAGCTGAAAC................................................................ 1

........................GTGCCTACTGAGCTGAAACAGTTGA.......................................................... 1

.........................TGCCTACTGAGCTGAAA................................................................. 1

..........................GCCTACTGAGCTGAAA................................................................. 1

............................CTACTGAGCTGAAACAGT............................................................. 2

.................................................TTCCAGTGCACTGGCTCAGTTC.................................... 1

....................................................CAGTGCACTGGCTCAGTTCAGCAGGA............................. 1

....................................................CAGTGCACTGGCTCAGTTCAGCA................................ 1

.......................................................TGCACTGGCTCAGTTC.................................... 1

..........................................................ACTGGCTCAGTTCAGCAGGAACAG......................... 2

...........................................................CTGGCTCAGTTCAGCAGGA............................. 1

............................................................TGGCTCAGTTCAGCAGGAAC........................... 13

............................................................TGGCTCAGTTCAGCAGGAACA.......................... 12

............................................................TGGCTCAGTTCAGCAGGA............................. 1

............................................................TGGCTCAGTTCAGCAG............................... 12

............................................................TGGCTCAGTTCAGCAGGAA............................ 16

............................................................TGGCTCAGTTCAGCAGGAACAG......................... 60

............................................................TGGCTCAGTTCAGCAGG.............................. 6

.............................................................GGCTCAGTTCAGCAGGAACAG......................... 2

...............................................................CTCAGTTCAGCAGGAAC........................... 1

>mmu-mir-96_MI0000583_Mus_musculus_miR-96_stem-loop ESC dcr

CCAGUACCAUCUGCUUGGCCGAUUUUGGCACUAGCACAUUUUUGCUUGUGUCUCUCCGCUGUGAGCAAUCAUGUGUAGUGCCAAUAUGGGAAAAGCGGGCUGCUGC

.(((((...(((((((..((.((.(((((((((.(((((..(((((..(((......)).)..)))))..)))))))))))))).)).))..))))))).))))). (-47.30)

...GTACCATCTGCTTGGCCGA.................................................................................... 1

...GTACCATCTGCTTGGCCGAT................................................................................... 8

....TACCATCTGCTTGGCCGA.................................................................................... 1

....TACCATCTGCTTGGCCGAT................................................................................... 11

.....ACCATCTGCTTGGCCGAT................................................................................... 19

......CCATCTGCTTGGCCGA.................................................................................... 2

......CCATCTGCTTGGCCGAT................................................................................... 13

.......................TTTGGCACTAGCACATTTTTGCTTGT......................................................... 12

.......................TTTGGCACTAGCACATTTTTGCTTG.......................................................... 17

.......................TTTGGCACTAGCACATTTTT............................................................... 6

.......................TTTGGCACTAGCACATTTT................................................................ 5

.......................TTTGGCACTAGCACATTTTTG.............................................................. 2

.......................TTTGGCACTAGCACATTTTTGCT............................................................ 29

.......................TTTGGCACTAGCACATTTTTGCTT........................................................... 45

.......................TTTGGCACTAGCACAT................................................................... 57

.......................TTTGGCACTAGCACATTT................................................................. 3

.......................TTTGGCACTAGCACATTTTTGC............................................................. 7

.......................TTTGGCACTAGCACATT.................................................................. 8

........................TTGGCACTAGCACATTTTTGCTT........................................................... 1

............................CACTAGCACATTTTTGCTTGT......................................................... 1

............................CACTAGCACATTTTTGCTTG.......................................................... 7

............................CACTAGCACATTTTTGC............................................................. 2

............................CACTAGCACATTTTTGCT............................................................ 9

............................CACTAGCACATTTTTGCTT........................................................... 6

.............................ACTAGCACATTTTTGCTTG.......................................................... 1

.............................ACTAGCACATTTTTGCT............................................................ 2

..............................CTAGCACATTTTTGCTTGTGT....................................................... 1

..............................CTAGCACATTTTTGCTT........................................................... 2

...................................ACATTTTTGCTTGTGTC...................................................... 1

.....................................ATTTTTGCTTGTGTCTCTCC................................................. 1

...................................................CTCTCCGCTGTGAGCAATCATGTGT.............................. 1

.........................................................GCTGTGAGCAATCATGTGTAGTGCC........................ 1

............................................................GTGAGCAATCATGTGTAGTGCCAATA.................... 1

............................................................GTGAGCAATCATGTGTAGTGCCAAT..................... 1

............................................................GTGAGCAATCATGTGTAG............................ 1

...............................................................AGCAATCATGTGTAGTGCCAAT..................... 1

................................................................GCAATCATGTGTAGTGC......................... 3

................................................................GCAATCATGTGTAGTGCC........................ 1

..................................................................AATCATGTGTAGTGCCAATAT................... 2

.....................................................................CATGTGTAGTGCCAAT..................... 2

.......................................................................................GGGAAAAGCGGGCTGCT.. 1

.......................................................................................GGGAAAAGCGGGCTGC... 1

>mmu-mir-331_MI0000609_Mus_musculus_miR-331_stem-loop ESC dcr

GAGUCUGGUUUUGUUUGGGUUUGUUCUAGGUAUGGUCCCAGGGAUCCCAGAUCAAACCAGGCCCCUGGGCCUAUCCUAGAACCAACCUAAACCCGU

......((....(((((((((.((((((((.((((.(((((((..(.............)..))))))).)))))))))))).))))))))))).. (-43.72)

....CTGGTTTTGTTTGGGTTTGTT....................................................................... 1

.....TGGTTTTGTTTGGGTTTGTT....................................................................... 3

......GGTTTTGTTTGGGTTTGTT....................................................................... 15

.......GTTTTGTTTGGGTTTGTT....................................................................... 13

.........................CTAGGTATGGTCCCAGGGATC.................................................. 1

............................................................GCCCCTGGGCCTATCCTAGA................ 1

>mmu-mir-669a-1_MI0004523_Mus_musculus_mir-669a-1_stem-loop ESC dcr

UGUAUGUGCAUGUGUGUAUAGUUGUGUGUGCAUGUUCAUGUCUAUAUUUGAAUAUACAUAACAUACACACACACGUAUAAACGCAAGCACACAUACA

.((((((((.(((((.((((..((((((((.(((((.((((.(((......))).))))))))).))))))))..)))).))))).)))))..))). (-36.80)

TGTATGTGCATGTGTGTAT.............................................................................. 3

TGTATGTGCATGTGTGT................................................................................ 1

..TATGTGCATGTGTGTAT.............................................................................. 4

....TGTGCATGTGTGTATAGTTGTGT...................................................................... 2

....TGTGCATGTGTGTATA............................................................................. 1

....TGTGCATGTGTGTATAGTTGTGTGT.................................................................... 1

....TGTGCATGTGTGTATAGTTGTGTG..................................................................... 4

..........TGTGTGTATAGTTGTG....................................................................... 1

..................TAGTTGTGTGTGCATGTT............................................................. 1

...................AGTTGTGTGTGCATGTTCATGTCTATA................................................... 3

...................AGTTGTGTGTGCATGTTCATGTCTAT.................................................... 4

...................AGTTGTGTGTGCATGTTCAT.......................................................... 3

...................AGTTGTGTGTGCATGTTCATGTCT...................................................... 26

...................AGTTGTGTGTGCATGTTC............................................................ 3

...................AGTTGTGTGTGCATGTTCATGT........................................................ 6

...................AGTTGTGTGTGCATGTTCATG......................................................... 1

...................AGTTGTGTGTGCATGTTCATGTCTA..................................................... 1

...................AGTTGTGTGTGCATGTTCATGTC....................................................... 14

....................GTTGTGTGTGCATGTTCATGTCT...................................................... 1

......................TGTGTGTGCATGTTCATGTCTAT.................................................... 1

................................TGTTCATGTCTATATTTGA.............................................. 1

........................................TCTATATTTGAATATACATAACATAC............................... 1

.........................................CTATATTTGAATATACATA..................................... 1

............................................TATTTGAATATACATAACAT................................. 1

.......................................................ACATAACATACACACACACGT..................... 1

..............................................................................AAACGCAAGCACACATA.. 1

..............................................................................AAACGCAAGCACACAT... 1

>mmu-mir-685_MI0004649_Mus_musculus_miR-685_stem-loop ESC dcr

GUCGGGGCGGAUGCCUCCCUCGCCGGAGCGGAUGCCUCCCUCGCCGGAGCUUGGAACAGACUCACGGCCAGCAGUGCGAGUUCAAUGGCUGAGGUGAGGCACCUCCCGG

..(((((.((.(((((((((((((.((((...(((((..((.(((((((((......)).))).)))).)).)).))).))))...))).)))).))))))))))))). (-53.00)

.......CGGATGCCTCCCTCGCC..................................................................................... 1

........GGATGCCTCCCTCGCC..................................................................................... 1

.........GATGCCTCCCTCGCCGGAGCGGA............................................................................. 1

.........GATGCCTCCCTCGCCGGAGC................................................................................ 2

..........ATGCCTCCCTCGCCGGAG................................................................................. 1

...........TGCCTCCCTCGCCGGAGCGGA............................................................................. 1

...........TGCCTCCCTCGCCGGAGC................................................................................ 1

.................CCTCGCCGGAGCGGATGCCTC....................................................................... 1

..........................AGCGGATGCCTCCCTCGCC................................................................ 1

............................CGGATGCCTCCCTCGCC................................................................ 1

............................CGGATGCCTCCCTCGCCGGAGCTT......................................................... 1

............................CGGATGCCTCCCTCGCCGGAGCTTGGA...................................................... 1

.............................GGATGCCTCCCTCGCC................................................................ 1

..............................GATGCCTCCCTCGCCGGAGC........................................................... 2

...............................ATGCCTCCCTCGCCGGAG............................................................ 1

................................TGCCTCCCTCGCCGGAGC........................................................... 1

..............................................GAGCTTGGAACAGACT............................................... 1

................................................GCTTGGAACAGACTCACGGCCAGC..................................... 1

..........................................................GACTCACGGCCAGCAGT.................................. 1

.......................................................................CAGTGCGAGTTCAATGG..................... 1

.............................................................................GAGTTCAATGGCTGAGGTGAGG.......... 2

..............................................................................AGTTCAATGGCTGAGGT.............. 1

..............................................................................AGTTCAATGGCTGAGGTGAGG.......... 8

..............................................................................AGTTCAATGGCTGAGGTGAGGCA........ 4

..............................................................................AGTTCAATGGCTGAGGTGAGGC......... 1

...............................................................................GTTCAATGGCTGAGGTGAGGC......... 3

...............................................................................GTTCAATGGCTGAGGTGAGG.......... 12

...............................................................................GTTCAATGGCTGAGGTGAG........... 1

...............................................................................GTTCAATGGCTGAGGTGAGGCACCTC.... 1

...............................................................................GTTCAATGGCTGAGGTGAGGCACCT..... 1

...............................................................................GTTCAATGGCTGAGGTGAGGCA........ 4

...............................................................................GTTCAATGGCTGAGGTGA............ 2

................................................................................TTCAATGGCTGAGGTGAG........... 1

................................................................................TTCAATGGCTGAGGTGAGGCA........ 10

................................................................................TTCAATGGCTGAGGTGAGGCACCTC.... 2

................................................................................TTCAATGGCTGAGGTGAGG.......... 5

.................................................................................TCAATGGCTGAGGTGAGGCACCT..... 2

.................................................................................TCAATGGCTGAGGTGAGGCACC...... 1

.................................................................................TCAATGGCTGAGGTGAGG.......... 4

.................................................................................TCAATGGCTGAGGTGAGGCA........ 2

.................................................................................TCAATGGCTGAGGTGAGGC......... 2

.................................................................................TCAATGGCTGAGGTGAG........... 1

..................................................................................CAATGGCTGAGGTGAGGCA........ 6

..................................................................................CAATGGCTGAGGTGAGGC......... 2

..................................................................................CAATGGCTGAGGTGAG........... 1

..................................................................................CAATGGCTGAGGTGAGG.......... 1

...................................................................................AATGGCTGAGGTGAGGC......... 9

...................................................................................AATGGCTGAGGTGAGGCA........ 7

...................................................................................AATGGCTGAGGTGAGG.......... 7

....................................................................................ATGGCTGAGGTGAGGCA........ 1

....................................................................................ATGGCTGAGGTGAGGC......... 1

.....................................................................................TGGCTGAGGTGAGGCA........ 2

........................................................................................CTGAGGTGAGGCACCTC.... 3

>mmu-mir-669b_MI0004666_Mus_musculus_miR-669b_stem-loop ESC dcr

AUGAAUGUAUGUGCAUGUGUAUAUAGUUUUGUGUGCAUGUGCAUGUGUGUCUAUUAAUGUACAUAUACAUACACACAAACAUAUACACGCAUGCGCA

.........(((((((((((.((((..((((((((.(((((.(((((..(......)..))))).))))).))))))))..)))).))))))))))) (-39.80)

....ATGTATGTGCATGTGTATAT......................................................................... 2

........................AGTTTTGTGTGCATGTGC....................................................... 1

........................AGTTTTGTGTGCATGTGCATGT................................................... 1

.............................TGTGTGCATGTGCATGTGTGT............................................... 2

...............................TGTGCATGTGCATGTGT................................................. 1

...........................................TGTGTGTCTATTAATGTAC................................... 1

>mmu-mir-669a-2_MI0004667_Mus_musculus_miR-669a-2_stem-loop ESC dcr

CAUGUAUGUGCAUGUGUGUAUAGUUGUGUGUGCAUGUUCAUGUCUAUAUUUGAAUAUACAUAACAUACACACACACGUAUAAACGCAAGCACACACA

..(((.(((((.(((((.((((..((((((((.(((((.((((.(((......))).))))))))).))))))))..)))).))))).))))).))) (-37.80)

CATGTATGTGCATGTGTGT.............................................................................. 2

CATGTATGTGCATGTGTGTAT............................................................................ 3

.ATGTATGTGCATGTGTGT.............................................................................. 3

.ATGTATGTGCATGTGTGTAT............................................................................ 13

..TGTATGTGCATGTGTGTAT............................................................................ 3

..TGTATGTGCATGTGTGT.............................................................................. 1

....TATGTGCATGTGTGTAT............................................................................ 4

......TGTGCATGTGTGTATAGTTGTGT.................................................................... 2

......TGTGCATGTGTGTATA........................................................................... 1

......TGTGCATGTGTGTATAGTTGTGTGT.................................................................. 1

......TGTGCATGTGTGTATAGTTGTGTG................................................................... 4

............TGTGTGTATAGTTGTG..................................................................... 1

....................TAGTTGTGTGTGCATGTT........................................................... 1

.....................AGTTGTGTGTGCATGTTCATGTCTATA................................................. 3

.....................AGTTGTGTGTGCATGTTCATGTCTAT.................................................. 4

.....................AGTTGTGTGTGCATGTTCAT........................................................ 3

.....................AGTTGTGTGTGCATGTTCATGTCT.................................................... 26

.....................AGTTGTGTGTGCATGTTC.......................................................... 3

.....................AGTTGTGTGTGCATGTTCATGT...................................................... 6

.....................AGTTGTGTGTGCATGTTCATG....................................................... 1

.....................AGTTGTGTGTGCATGTTCATGTCTA................................................... 1

.....................AGTTGTGTGTGCATGTTCATGTC..................................................... 14

......................GTTGTGTGTGCATGTTCATGTCT.................................................... 1

........................TGTGTGTGCATGTTCATGTCTAT.................................................. 1

..................................TGTTCATGTCTATATTTGA............................................ 1

..........................................TCTATATTTGAATATACATAACATAC............................. 1

...........................................CTATATTTGAATATACATA................................... 1

..............................................TATTTGAATATACATAACAT............................... 1

.........................................................ACATAACATACACACACACGT................... 1

>mmu-mir-669a-3_MI0004668_Mus_musculus_miR-669a-3_stem-loop ESC dcr

UUCCUCCAUGUAUGUGCAUGUGUGUAUAGUUGUGUGUGCAUGUUCAUGUCUAUAUUUGAAUAUACAUAACAUACACACACAUGUAUAAACGCAAGCACACAUACACAGA

........(((((((((.(((((.((((..((((((((.(((((.((((.(((......))).))))))))).))))))))..)))).))))).)))))..)))).... (-37.90)

...CTCCATGTATGTGCATGTGTGTAT.................................................................................. 1

.....CCATGTATGTGCATGTGTGT.................................................................................... 3

......CATGTATGTGCATGTGTGT.................................................................................... 2

......CATGTATGTGCATGTGTGTAT.................................................................................. 3

.......ATGTATGTGCATGTGTGT.................................................................................... 3

.......ATGTATGTGCATGTGTGTAT.................................................................................. 13

........TGTATGTGCATGTGTGTAT.................................................................................. 3

........TGTATGTGCATGTGTGT.................................................................................... 1

..........TATGTGCATGTGTGTAT.................................................................................. 4

............TGTGCATGTGTGTATAGTTGTGT.......................................................................... 2

............TGTGCATGTGTGTATA................................................................................. 1

............TGTGCATGTGTGTATAGTTGTGTGT........................................................................ 1

............TGTGCATGTGTGTATAGTTGTGTG......................................................................... 4

..................TGTGTGTATAGTTGTG........................................................................... 1

..........................TAGTTGTGTGTGCATGTT................................................................. 1

...........................AGTTGTGTGTGCATGTTCATGTCTATA....................................................... 3

...........................AGTTGTGTGTGCATGTTCATGTCTAT........................................................ 4

...........................AGTTGTGTGTGCATGTTCAT.............................................................. 3

...........................AGTTGTGTGTGCATGTTCATGTCT.......................................................... 26

...........................AGTTGTGTGTGCATGTTC................................................................ 3

...........................AGTTGTGTGTGCATGTTCATGT............................................................ 6

...........................AGTTGTGTGTGCATGTTCATG............................................................. 1

...........................AGTTGTGTGTGCATGTTCATGTCTA......................................................... 1

...........................AGTTGTGTGTGCATGTTCATGTC........................................................... 14

............................GTTGTGTGTGCATGTTCATGTCT.......................................................... 1

..............................TGTGTGTGCATGTTCATGTCTAT........................................................ 1

........................................TGTTCATGTCTATATTTGA.................................................. 1

................................................TCTATATTTGAATATACATAACATAC................................... 1

.................................................CTATATTTGAATATACATA......................................... 1

....................................................TATTTGAATATACATAACAT..................................... 1

......................................................................................AAACGCAAGCACACATA...... 1

......................................................................................AAACGCAAGCACACAT....... 1

>mmu-mir-708_MI0004692_Mus_musculus_miR-708_stem-loop ESC dcr

CUGUGUUUGAAAUGGGGACUGCCCUCAAGGAGCUUACAAUCUAGCUGGGGGUAGAUGACUUGCACUUGAACACAACUAGACUGUGAGCUUCUAGAGGGCAGGGGCCUUA

............(((((.((((((((.(((((((((((.(((((.((.(..(((.((.....)).)))..).)).))))).))))))))))).))))))))...))))) (-48.50)

......TTGAAATGGGGACTGCCCTC................................................................................... 2

.......TGAAATGGGGACTGCCCTC................................................................................... 2

..........................AAGGAGCTTACAATCTAGCTGGGGG.......................................................... 1

..........................AAGGAGCTTACAATCTAGCT............................................................... 1

.............................GAGCTTACAATCTAGCTGG............................................................. 1

...................................ACAATCTAGCTGGGGGTA........................................................ 1

..........................................................................ACTAGACTGTGAGCTTCTAG............... 1

>mmu-mir-2134-3_MI0010742_Mus_musculus_miR-2134-3_stem-loop ESC dgcr8

GAUCCGGUGCGGAGAGCCGUUGGUCUUGGGAAACGGGGUGCGGCCGGAAAGGGGGCCGCCGUCUCGCCCGUGGAACCUGGCGCUAAACCA

.....(((....((.((((..(((((((((...((((..((((((........))))).)..)))))))).)).))))))).))..))). (-36.40)

..TCCGGTGCGGAGAGCCGTT..................................................................... 2

..TCCGGTGCGGAGAGCC........................................................................ 8

..TCCGGTGCGGAGAGCCGT...................................................................... 6

...CCGGTGCGGAGAGCCG....................................................................... 15

...CCGGTGCGGAGAGCCGTT..................................................................... 9

...CCGGTGCGGAGAGCCGT...................................................................... 83

....CGGTGCGGAGAGCCGT...................................................................... 8

....CGGTGCGGAGAGCCGTT..................................................................... 6

.....GGTGCGGAGAGCCGTT..................................................................... 6

......................GTCTTGGGAAACGGGGT................................................... 2

......................GTCTTGGGAAACGGGGTGCGGCC............................................. 10

......................GTCTTGGGAAACGGGGTGC................................................. 1

......................GTCTTGGGAAACGGGGTGCGGCCG............................................ 1

......................GTCTTGGGAAACGGGGTGCGGCCGGA.......................................... 1

......................GTCTTGGGAAACGGGGTGCGGC.............................................. 1

.......................TCTTGGGAAACGGGGTGCGGCC............................................. 1

.......................TCTTGGGAAACGGGGT................................................... 1

.......................TCTTGGGAAACGGGGTG.................................................. 1

........................CTTGGGAAACGGGGTGCGG............................................... 2

........................CTTGGGAAACGGGGTGCGGCC............................................. 4

........................CTTGGGAAACGGGGTGC................................................. 1

........................CTTGGGAAACGGGGTGCGGC.............................................. 2

........................CTTGGGAAACGGGGTGCGGCCG............................................ 1

.........................TTGGGAAACGGGGTGCGGC.............................................. 10

.........................TTGGGAAACGGGGTGCGGCCG............................................ 2

.........................TTGGGAAACGGGGTGC................................................. 3

.........................TTGGGAAACGGGGTGCGGCCGG........................................... 2

.........................TTGGGAAACGGGGTGCGGCCGGA.......................................... 2

.........................TTGGGAAACGGGGTGCGGCC............................................. 7

.........................TTGGGAAACGGGGTGCG................................................ 1

.........................TTGGGAAACGGGGTGCGGCCGGAAAG....................................... 2

..........................TGGGAAACGGGGTGCGGC.............................................. 2

..........................TGGGAAACGGGGTGCGG............................................... 3

...........................GGGAAACGGGGTGCGGC.............................................. 1

............................GGAAACGGGGTGCGGC.............................................. 2

.............................GAAACGGGGTGCGGCCGGA.......................................... 3

.............................GAAACGGGGTGCGGCCGGAA......................................... 1

.............................GAAACGGGGTGCGGCC............................................. 6

..............................AAACGGGGTGCGGCCGGAAA........................................ 1

..............................AAACGGGGTGCGGCCG............................................ 1

..............................AAACGGGGTGCGGCCGGA.......................................... 3

..............................AAACGGGGTGCGGCCGG........................................... 1

..............................AAACGGGGTGCGGCCGGAA......................................... 1

...............................AACGGGGTGCGGCCGGAA......................................... 19

...............................AACGGGGTGCGGCCGG........................................... 45

...............................AACGGGGTGCGGCCGGAAAGGGGGCCG................................ 1

...............................AACGGGGTGCGGCCGGAAAGGGGGCC................................. 3

...............................AACGGGGTGCGGCCGGAAA........................................ 49

...............................AACGGGGTGCGGCCGGA.......................................... 59

................................ACGGGGTGCGGCCGGAAAGGGG.................................... 1

................................ACGGGGTGCGGCCGGA.......................................... 10

................................ACGGGGTGCGGCCGGAAAG....................................... 1

................................ACGGGGTGCGGCCGGAAA........................................ 12

................................ACGGGGTGCGGCCGGAA......................................... 7

................................ACGGGGTGCGGCCGGAAAGGGGGCCGC............................... 1

................................ACGGGGTGCGGCCGGAAAGGGGGCC................................. 1

.................................CGGGGTGCGGCCGGAAAGGGGGCC................................. 2

.................................CGGGGTGCGGCCGGAAAGGGGGC.................................. 2

.................................CGGGGTGCGGCCGGAAAGGGGG................................... 8

.................................CGGGGTGCGGCCGGAA......................................... 3

.................................CGGGGTGCGGCCGGAAAGGGGGCCGCC.............................. 1

.................................CGGGGTGCGGCCGGAAA........................................ 2

..................................GGGGTGCGGCCGGAAAG....................................... 12

..................................GGGGTGCGGCCGGAAA........................................ 90

..................................GGGGTGCGGCCGGAAAGGGGGCCGCC.............................. 2

..................................GGGGTGCGGCCGGAAAGGGGGC.................................. 4

..................................GGGGTGCGGCCGGAAAGGGGGCC................................. 8

..................................GGGGTGCGGCCGGAAAGGGGGCCGC............................... 1

...................................GGGTGCGGCCGGAAAGGG..................................... 1

...................................GGGTGCGGCCGGAAAGGGGGCCGCC.............................. 2

...................................GGGTGCGGCCGGAAAG....................................... 29

...................................GGGTGCGGCCGGAAAGGGGGC.................................. 1

...................................GGGTGCGGCCGGAAAGGGGGCCGC............................... 1

...................................GGGTGCGGCCGGAAAGGGGGCC................................. 16

....................................GGTGCGGCCGGAAAGG...................................... 4

....................................GGTGCGGCCGGAAAGGGG.................................... 1

....................................GGTGCGGCCGGAAAGGGGGCCGCC.............................. 1

....................................GGTGCGGCCGGAAAGGG..................................... 2

....................................GGTGCGGCCGGAAAGGGGGCC................................. 35

....................................GGTGCGGCCGGAAAGGGGGC.................................. 2

....................................GGTGCGGCCGGAAAGGGGGCCGC............................... 1

....................................GGTGCGGCCGGAAAGGGGG................................... 11

.....................................GTGCGGCCGGAAAGGGG.................................... 1

.....................................GTGCGGCCGGAAAGGGGGCCGC............................... 1

.....................................GTGCGGCCGGAAAGGGGG................................... 1

.....................................GTGCGGCCGGAAAGGGGGCCGCC.............................. 1

.....................................GTGCGGCCGGAAAGGGGGCC................................. 3

......................................TGCGGCCGGAAAGGGGGC.................................. 2

......................................TGCGGCCGGAAAGGGGGCC................................. 5

......................................TGCGGCCGGAAAGGGGGCCGCC.............................. 1

.......................................GCGGCCGGAAAGGGGG................................... 3

.......................................GCGGCCGGAAAGGGGGCC................................. 5

.......................................GCGGCCGGAAAGGGGGCCGC............................... 1

........................................CGGCCGGAAAGGGGGCCGC............................... 1

........................................CGGCCGGAAAGGGGGCCGCC.............................. 1

........................................CGGCCGGAAAGGGGGCC................................. 10

........................................CGGCCGGAAAGGGGGC.................................. 1

.........................................GGCCGGAAAGGGGGCCGCC.............................. 1

.........................................GGCCGGAAAGGGGGCCGC............................... 4

.........................................GGCCGGAAAGGGGGCC................................. 3

..........................................GCCGGAAAGGGGGCCGCC.............................. 27

..........................................GCCGGAAAGGGGGCCGC............................... 18

..........................................GCCGGAAAGGGGGCCGCCGTC........................... 1

..........................................GCCGGAAAGGGGGCCG................................ 10

...........................................CCGGAAAGGGGGCCGC............................... 139

...........................................CCGGAAAGGGGGCCGCC.............................. 48

............................................CGGAAAGGGGGCCGCC.............................. 82

.............................................GGAAAGGGGGCCGCCGT............................ 1

.....................................................................GTGGAACCTGGCGCTAAAC.. 3

.....................................................................GTGGAACCTGGCGCTAAACC. 9

......................................................................TGGAACCTGGCGCTAAACC. 2

......................................................................TGGAACCTGGCGCTAAACCA 1

........................................................................GAACCTGGCGCTAAACC. 1

.........................................................................AACCTGGCGCTAAACC. 6

.........................................................................AACCTGGCGCTAAACCA 5

..........................................................................ACCTGGCGCTAAACCA 44
