# Supplementary material for: Multiple distinct small RNAs originate from the same microRNA precursors
Source: Genome Biol. 2010 Aug 9;11(8):R81. doi: 10.1186/gb-2010-11-8-r81 (PMC2945783; doi:10.1186/gb-2010-11-8-r81)
Supplement: Additional file 9 — Supplemental File S8. This is a file for sequencing reads mapped and aligned to miRNA precursors that can produce miRNA-sibling small RNAs (msRNAs) in C. elegans (cel). The sequencing data were obtained from GEO; see Materials and methods for details. [file gb-2010-11-8-r81-S9.DOCX]

Zhang, et al., Multiple distinct small RNAs originate from the same microRNA precursors

Supplemental File 8 - Sequencing reads mapped and aligned to miRNA precursors that can produce

miRNA-like RNAs in *C.elegans.*

>cel-mir-61_MI0000032_Caenorhabditis_elegans_miR-61_stem-loop GSM139137

UUCCAUUAUCGCUGAACCUCGAGAUGGGUUACGGGGCUUAGUCCUUCCUCCGUAUGGCAAUGACUAGAACCGUUACUCAUCUCGAGGUUUCGGUGAU

.......((((((((((((((((((((((.((((..((.((((.((((.......)).)).))))))..)))).))))))))))))).))))))))) (-50.50)

.TCCATTATCGCTGAACCTCGA........................................................................... 1

........................TGGGTTACGGGGCTTAGTCCTT................................................... 3

..........................................................AATGACTAGAACCGTTACTCATC................ 1

...........................................................ATGACTAGAACCGTTACTCATC................ 2

............................................................TGACTAGAACCGTTACTCATCT............... 75

............................................................TGACTAGAACCGTTACTCA.................. 3

............................................................TGACTAGAACCGTTACTCATCTCG............. 4

............................................................TGACTAGAACCGTTACTC................... 1

............................................................TGACTAGAACCGTTACTCATC................ 1677

............................................................TGACTAGAACCGTTACTCAT................. 123

.............................................................GACTAGAACCGTTACTCATC................ 3

...............................................................CTAGAACCGTTACTCATCTCG............. 2

>cel-mir-51_MI0000022_Caenorhabditis_elegans_miR-51_stem-loop GSM444000

GUCCGAAAAGUCCGUCUACCCGUAGCUCCUAUCCAUGUUACUGGUCAAAAAGUGAACAUGGAAGCAGGUACAGGUGCACGGCGAGUAGGGUCAUGAAGCU

((((.....(.((((.((((.(((.((.((.(((((((((((........)))).))))))))).)).))).)))).))))).....))).)........ (-33.30)

GTCCGAAAAGTCCGTCTACCC............................................................................... 1

................TACCCGTAGCTCCTATCC.................................................................. 26

................TACCCGTAGCTCCTATCCAT................................................................ 46

................TACCCGTAGCTCCTATCCATG............................................................... 324

................TACCCGTAGCTCCTATCCATGT.............................................................. 1834

................TACCCGTAGCTCCTATCCATGTT............................................................. 27486

................TACCCGTAGCTCCTATCCATGTTAC........................................................... 12

.................ACCCGTAGCTCCTATCCATGTT............................................................. 5

.................ACCCGTAGCTCCTATCCATGTTAC........................................................... 1

..................CCCGTAGCTCCTATCCATG............................................................... 1

..................CCCGTAGCTCCTATCCATGTT............................................................. 6

...................CCGTAGCTCCTATCCATGTT............................................................. 2

........................GCTCCTATCCATGTTACTGGTC...................................................... 1

........................................................CATGGAAGCAGGTACAGGT......................... 1

........................................................CATGGAAGCAGGTACAGGTGC....................... 11

>cel-mir-60_MI0000031_Caenorhabditis_elegans_miR-60_stem-loop GSM444000

CUCGAAAACCGCUUGUUCUUGAACUGGAAGAGUGCCAUAAAAUCAUGACAAAGUACGUGAUAUUAUGCACAUUUUCUAGUUCAAGACUUGAGAAAUCG

................(((((((((((((((((((.((((.((((((........)))))).)))))))).)))))))))))))))...((....)). (-35.00)

..CGAAAACCGCTTGTTCTTGAAC.......................................................................... 2

.....................AACTGGAAGAGTGCCATAAAAT....................................................... 27

.....................AACTGGAAGAGTGCCATAAAATC...................................................... 59

.....................AACTGGAAGAGTGCCATAAAATCAT.................................................... 1

......................ACTGGAAGAGTGCCATAAAATC...................................................... 1

...........................................................ATATTATGCACATTTTCTAGTTC................ 6

............................................................TATTATGCACATTTTCTAG................... 1

............................................................TATTATGCACATTTTCTAGTT................. 4

............................................................TATTATGCACATTTTCTAGTTC................ 2595

.............................................................ATTATGCACATTTTCTAGTTC................ 1

>cel-mir-61_MI0000032_Caenorhabditis_elegans_miR-61_stem-loop GSM444000

UUCCAUUAUCGCUGAACCUCGAGAUGGGUUACGGGGCUUAGUCCUUCCUCCGUAUGGCAAUGACUAGAACCGUUACUCAUCUCGAGGUUUCGGUGAU

.......((((((((((((((((((((((.((((..((.((((.((((.......)).)).))))))..)))).))))))))))))).))))))))) (-50.50)

.TCCATTATCGCTGAACCTCG............................................................................ 8

.......................ATGGGTTACGGGGCTTAGTCCTT................................................... 4

........................TGGGTTACGGGGCTTAGTCCT.................................................... 5

........................TGGGTTACGGGGCTTAGTCCTT................................................... 16

........................TGGGTTACGGGGCTTAGTCCTTC.................................................. 1

..............................ACGGGGCTTAGTCCTTCCTC............................................... 1

.......................................AGTCCTTCCTCCGTATGGCAA..................................... 1

...........................................................ATGACTAGAACCGTTACTCATC................ 8

............................................................TGACTAGAACCGTTACTC................... 34

............................................................TGACTAGAACCGTTACTCAT................. 451

............................................................TGACTAGAACCGTTACTCATC................ 3276

............................................................TGACTAGAACCGTTACTCATCT............... 297

............................................................TGACTAGAACCGTTACTCATCTC.............. 5

............................................................TGACTAGAACCGTTACTCATCTCG............. 4

.............................................................GACTAGAACCGTTACTCAT................. 1

.............................................................GACTAGAACCGTTACTCATC................ 3

..............................................................ACTAGAACCGTTACTCAT................. 1

..............................................................ACTAGAACCGTTACTCATCTCG............. 1

...............................................................CTAGAACCGTTACTCATCTCG............. 4

>cel-mir-63_MI0000034_Caenorhabditis_elegans_miR-63_stem-loop GSM444000

UCAACAAGCAGACACAAUUUCUAACUCGUCGGUAGUCAUCGUUCUAGCUGAAAAGGACACUAUGACACUGAAGCGAGUUGGAAAUAGUGGUUCUACUUGAGCAA

....((((.((((((.(((((((((((((((((.(((((.(((((........)))))...)))))))))..))))))))))))).)))..))).))))..... (-38.00)

...................TCTAACTCGTCGGTAGTCAT................................................................. 1

...................TCTAACTCGTCGGTAGTCATC................................................................ 2

...................TCTAACTCGTCGGTAGTCATCG............................................................... 32

...................TCTAACTCGTCGGTAGTCATCGT.............................................................. 255

...................TCTAACTCGTCGGTAGTCATCGTT............................................................. 1

....................CTAACTCGTCGGTAGTCATCGT.............................................................. 1

....................................CATCGTTCTAGCTGAAAAGGAC.............................................. 1

............................................................TATGACACTGAAGCGAGT.......................... 41

............................................................TATGACACTGAAGCGAGTT......................... 13

............................................................TATGACACTGAAGCGAGTTG........................ 102

............................................................TATGACACTGAAGCGAGTTGG....................... 3345

.............................................................ATGACACTGAAGCGAGTTGG....................... 3

.............................................................................TTGGAAATAGTGGTTCTACTTG..... 1

>cel-mir-244_MI0000320_Caenorhabditis_elegans_miR-244_stem-loop GSM444000

CUCCAUAUCUCAAUCUCUUUGGUUGUACAAAGUGGUAUGGCUCAUCGAAUAAGCACAUACUGCUUUUCAGCUAAAGGAAUUGAGAUUUUGUAGGCUUUU

((.((.((((((((..(((((((((...((((..((((((((.........))).)))))..)))).)))))))))..))))))))..)).))...... (-38.20)

...............TCTTTGGTTGTACAAAGTGG................................................................ 3

...............TCTTTGGTTGTACAAAGTGGT............................................................... 135

...............TCTTTGGTTGTACAAAGTGGTAT............................................................. 109

...............TCTTTGGTTGTACAAAGTGGTATG............................................................ 2395

...............TCTTTGGTTGTACAAAGTGGTATGG........................................................... 2

................CTTTGGTTGTACAAAGTGGTATG............................................................ 1

..................TTGGTTGTACAAAGTGGTATG............................................................ 1

.........................................................TACTGCTTTTCAGCTAAAGG...................... 5

.........................................................TACTGCTTTTCAGCTAAAGGAAT................... 1

............................................................................GAATTGAGATTTTGTAGGCTTT. 1

.................................................................................GAGATTTTGTAGGCTTTT 1

>cel-mir-60_MI0000031_Caenorhabditis_elegans_miR-60_stem-loop GSM444001

CUCGAAAACCGCUUGUUCUUGAACUGGAAGAGUGCCAUAAAAUCAUGACAAAGUACGUGAUAUUAUGCACAUUUUCUAGUUCAAGACUUGAGAAAUCG

................(((((((((((((((((((.((((.((((((........)))))).)))))))).)))))))))))))))...((....)). (-35.00)

..CGAAAACCGCTTGTTCTTGAAC.......................................................................... 2

.....................AACTGGAAGAGTGCCATAAAAT....................................................... 27

.....................AACTGGAAGAGTGCCATAAAATC...................................................... 59

.....................AACTGGAAGAGTGCCATAAAATCAT.................................................... 1

......................ACTGGAAGAGTGCCATAAAATC...................................................... 1

...........................................................ATATTATGCACATTTTCTAGTTC................ 6

............................................................TATTATGCACATTTTCTAG................... 1

............................................................TATTATGCACATTTTCTAGTT................. 4

............................................................TATTATGCACATTTTCTAGTTC................ 2595

.............................................................ATTATGCACATTTTCTAGTTC................ 1

>cel-mir-61_MI0000032_Caenorhabditis_elegans_miR-61_stem-loop GSM444001

UUCCAUUAUCGCUGAACCUCGAGAUGGGUUACGGGGCUUAGUCCUUCCUCCGUAUGGCAAUGACUAGAACCGUUACUCAUCUCGAGGUUUCGGUGAU

.......((((((((((((((((((((((.((((..((.((((.((((.......)).)).))))))..)))).))))))))))))).))))))))) (-50.50)

.TCCATTATCGCTGAACCTCG............................................................................ 8

.......................ATGGGTTACGGGGCTTAGTCCTT................................................... 4

........................TGGGTTACGGGGCTTAGTCCT.................................................... 5

........................TGGGTTACGGGGCTTAGTCCTT................................................... 16

........................TGGGTTACGGGGCTTAGTCCTTC.................................................. 1

..............................ACGGGGCTTAGTCCTTCCTC............................................... 1

.......................................AGTCCTTCCTCCGTATGGCAA..................................... 1

...........................................................ATGACTAGAACCGTTACTCATC................ 8

............................................................TGACTAGAACCGTTACTC................... 34

............................................................TGACTAGAACCGTTACTCAT................. 451

............................................................TGACTAGAACCGTTACTCATC................ 3276

............................................................TGACTAGAACCGTTACTCATCT............... 297

............................................................TGACTAGAACCGTTACTCATCTC.............. 5

............................................................TGACTAGAACCGTTACTCATCTCG............. 4

.............................................................GACTAGAACCGTTACTCAT................. 1

.............................................................GACTAGAACCGTTACTCATC................ 3

..............................................................ACTAGAACCGTTACTCAT................. 1

..............................................................ACTAGAACCGTTACTCATCTCG............. 1

...............................................................CTAGAACCGTTACTCATCTCG............. 4

>cel-mir-244_MI0000320_Caenorhabditis_elegans_miR-244_stem-loop GSM444001

CUCCAUAUCUCAAUCUCUUUGGUUGUACAAAGUGGUAUGGCUCAUCGAAUAAGCACAUACUGCUUUUCAGCUAAAGGAAUUGAGAUUUUGUAGGCUUUU

((.((.((((((((..(((((((((...((((..((((((((.........))).)))))..)))).)))))))))..))))))))..)).))...... (-38.20)

...............TCTTTGGTTGTACAAAGTGG................................................................ 3

...............TCTTTGGTTGTACAAAGTGGT............................................................... 135

...............TCTTTGGTTGTACAAAGTGGTAT............................................................. 109

...............TCTTTGGTTGTACAAAGTGGTATG............................................................ 2395

...............TCTTTGGTTGTACAAAGTGGTATGG........................................................... 2

................CTTTGGTTGTACAAAGTGGTATG............................................................ 1

..................TTGGTTGTACAAAGTGGTATG............................................................ 1

.........................................................TACTGCTTTTCAGCTAAAGG...................... 5

.........................................................TACTGCTTTTCAGCTAAAGGAAT................... 1

............................................................................GAATTGAGATTTTGTAGGCTTT. 1

.................................................................................GAGATTTTGTAGGCTTTT 1

>cel-mir-61_MI0000032_Caenorhabditis_elegans_miR-61_stem-loop GSM444002

UUCCAUUAUCGCUGAACCUCGAGAUGGGUUACGGGGCUUAGUCCUUCCUCCGUAUGGCAAUGACUAGAACCGUUACUCAUCUCGAGGUUUCGGUGAU

.......((((((((((((((((((((((.((((..((.((((.((((.......)).)).))))))..)))).))))))))))))).))))))))) (-50.50)

.TCCATTATCGCTGAACCT.............................................................................. 1

.TCCATTATCGCTGAACCTCGA........................................................................... 3

........................TGGGTTACGGGGCTTAGT....................................................... 4

........................TGGGTTACGGGGCTTAGTCCTT................................................... 2

............................................................TGACTAGAACCGTTACTC................... 185

............................................................TGACTAGAACCGTTACTCA.................. 194

............................................................TGACTAGAACCGTTACTCAT................. 459

............................................................TGACTAGAACCGTTACTCATC................ 260

............................................................TGACTAGAACCGTTACTCATCT............... 28

.............................................................GACTAGAACCGTTACTCA.................. 3

.............................................................GACTAGAACCGTTACTCAT................. 6

.............................................................GACTAGAACCGTTACTCATC................ 3

..............................................................ACTAGAACCGTTACTCAT................. 1

...............................................................CTAGAACCGTTACTCATC................ 2

...............................................................CTAGAACCGTTACTCATCT............... 1

...............................................................CTAGAACCGTTACTCATCTC.............. 2

...............................................................CTAGAACCGTTACTCATCTCG............. 1

>cel-mir-70_MI0000041_Caenorhabditis_elegans_miR-70_stem-loop GSM444002

UCAAAAUAAAACGAUGAAAACUAUCGAAAUACUAUCGACGAAUAACACUUAUGAAGAAAUGUAAUACGUCGUUGGUGUUUCCAUAGUUUGAAUUGUUUAU

........(((((((..(((((((.(((((((((.(((((.((.(((.((......)).))).)).))))).))))))))).)))))))..))))))).. (-32.30)

..AAAATAAAACGATGAAAACT.............................................................................. 1

...AAATAAAACGATGAAAACTAT............................................................................ 1

.....................TATCGAAATACTATCGACGAAT......................................................... 1

........................CGAAATACTATCGACGAAT......................................................... 10

........................CGAAATACTATCGACGAATA........................................................ 12

........................CGAAATACTATCGACGAATAA....................................................... 31

........................CGAAATACTATCGACGAATAAC...................................................... 41

........................CGAAATACTATCGACGAATAACA..................................................... 16

..............................................ACTTATGAAGAAATGTAATACGTC.............................. 1

.............................................................TAATACGTCGTTGGTGTT..................... 663

.............................................................TAATACGTCGTTGGTGTTT.................... 1332

.............................................................TAATACGTCGTTGGTGTTTC................... 3559

.............................................................TAATACGTCGTTGGTGTTTCC.................. 2461

.............................................................TAATACGTCGTTGGTGTTTCCA................. 5106

.............................................................TAATACGTCGTTGGTGTTTCCAT................ 7198

.............................................................TAATACGTCGTTGGTGTTTCCATA............... 11

..............................................................AATACGTCGTTGGTGTTT.................... 117

..............................................................AATACGTCGTTGGTGTTTC................... 127

..............................................................AATACGTCGTTGGTGTTTCC.................. 201

..............................................................AATACGTCGTTGGTGTTTCCA................. 407

..............................................................AATACGTCGTTGGTGTTTCCAT................ 393

...............................................................ATACGTCGTTGGTGTTTC................... 1

...............................................................ATACGTCGTTGGTGTTTCC.................. 2

...............................................................ATACGTCGTTGGTGTTTCCA................. 11

...............................................................ATACGTCGTTGGTGTTTCCAT................ 17

................................................................TACGTCGTTGGTGTTTCC.................. 4

................................................................TACGTCGTTGGTGTTTCCA................. 16

................................................................TACGTCGTTGGTGTTTCCAT................ 20

................................................................TACGTCGTTGGTGTTTCCATA............... 3

................................................................TACGTCGTTGGTGTTTCCATAG.............. 1

................................................................TACGTCGTTGGTGTTTCCATAGT............. 1

.................................................................ACGTCGTTGGTGTTTCCA................. 133

.................................................................ACGTCGTTGGTGTTTCCAT................ 129

.................................................................ACGTCGTTGGTGTTTCCATA............... 1

..................................................................CGTCGTTGGTGTTTCCAT................ 27

..................................................................CGTCGTTGGTGTTTCCATA............... 2

>cel-mir-75_MI0000046_Caenorhabditis_elegans_miR-75_stem-loop GSM444002

UUCUUGUUGCUUUGAAGAAUUGCAGUCGGUUGCAAGCUUAAAUACAAAUCCGAAUUGUUAUUAAAGCUACCAACCGGCUUCAAGUCUGAAAGAGCAG

.......((((((..(((.(((.(((((((((..(((((.(((((((.......))).)))).)))))..))))))))).))).)))...)))))). (-33.60)

TTCTTGTTGCTTTGAAGAATT............................................................................ 1

......................CAGTCGGTTGCAAGCTTA......................................................... 4

......................CAGTCGGTTGCAAGCTTAA........................................................ 15

......................CAGTCGGTTGCAAGCTTAAA....................................................... 20

......................CAGTCGGTTGCAAGCTTAAAT...................................................... 17

......................CAGTCGGTTGCAAGCTTAAATA..................................................... 5

.......................AGTCGGTTGCAAGCTTAA........................................................ 1

............................................................TTAAAGCTACCAACCGGC................... 6

............................................................TTAAAGCTACCAACCGGCT.................. 76

............................................................TTAAAGCTACCAACCGGCTT................. 805

............................................................TTAAAGCTACCAACCGGCTTC................ 1609

............................................................TTAAAGCTACCAACCGGCTTCA............... 1789

............................................................TTAAAGCTACCAACCGGCTTCAA.............. 2

.............................................................TAAAGCTACCAACCGGCT.................. 1

.............................................................TAAAGCTACCAACCGGCTT................. 9

.............................................................TAAAGCTACCAACCGGCTTC................ 22

.............................................................TAAAGCTACCAACCGGCTTCA............... 26

.............................................................TAAAGCTACCAACCGGCTTCAA.............. 1

..............................................................AAAGCTACCAACCGGCTT................. 28

..............................................................AAAGCTACCAACCGGCTTC................ 67

..............................................................AAAGCTACCAACCGGCTTCA............... 87

..............................................................AAAGCTACCAACCGGCTTCAA.............. 1

...............................................................AAGCTACCAACCGGCTTCA............... 1

...............................................................AAGCTACCAACCGGCTTCAA.............. 1

................................................................AGCTACCAACCGGCTTCA............... 1

>cel-mir-232_MI0000307_Caenorhabditis_elegans_miR-232_stem-loop GSM444002

AAGUUCAAUUUUUGGAUCCCUGCAGUUUCGAUGAUUUUAUCCUUAAUUCUGAAGAUGUGAUAAAUGCAUCUUAACUGCGGUGAUCUAGAUCAUGAACA

..(((((...((((((((.((((((((..((((..(((((((.....((....)).).))))))..))))..)))))))).))))))))...))))). (-35.60)

..GTTCAATTTTTGGATCCCTGCA.......................................................................... 1

..................CCTGCAGTTTCGATGATTTT............................................................ 3

....................TGCAGTTTCGATGATTTTA........................................................... 1

............................................................TAAATGCATCTTAACTGCG................... 5

............................................................TAAATGCATCTTAACTGCGG.................. 95

............................................................TAAATGCATCTTAACTGCGGT................. 300

............................................................TAAATGCATCTTAACTGCGGTG................ 10

............................................................TAAATGCATCTTAACTGCGGTGA............... 164

............................................................TAAATGCATCTTAACTGCGGTGAT.............. 7

.............................................................AAATGCATCTTAACTGCGG.................. 5

.............................................................AAATGCATCTTAACTGCGGT................. 19

.............................................................AAATGCATCTTAACTGCGGTGA............... 7

..............................................................AATGCATCTTAACTGCGGT................. 3

...............................................................ATGCATCTTAACTGCGGTGA............... 2

>cel-mir-258-1_MI0000335_Caenorhabditis_elegans_miR-258-1_stem-loop GSM444003

GCAAUGGUUUUGAGAGGAAUCCUUUUACAUAUUUGUUGAAGUUUUCGCUCGAAUUUGUGGUCGAAUACUGUAGAAGGAAGCCUGCACGAAGUUUUG

.......(((((.((((..((((((((((((((((.....(..(((....)))..).....)))))).))))))))))..))).).)))))..... (-24.60)

.....GGTTTTGAGAGGAATCCTTTT...................................................................... 1

............................ATATTTGTTGAAGTTTTCGC................................................ 1

............................ATATTTGTTGAAGTTTTCGCTC.............................................. 1

..............................ATTTGTTGAAGTTTTCGCTC.............................................. 1

..................................GTTGAAGTTTTCGCTCGAAT.......................................... 1

..................................GTTGAAGTTTTCGCTCGAATT......................................... 1

.....................................GAAGTTTTCGCTCGAATT......................................... 1

.....................................GAAGTTTTCGCTCGAATTT........................................ 1

........................................GTTTTCGCTCGAATTTGT...................................... 2

..............................................GCTCGAATTTGTGGTCGAATA............................. 1

..............................................GCTCGAATTTGTGGTCGAATAC............................ 1

..................................................GAATTTGTGGTCGAATACTG.......................... 1

..................................................GAATTTGTGGTCGAATACTGT......................... 1

..................................................GAATTTGTGGTCGAATACTGTA........................ 2

..............................................................GAATACTGTAGAAGGAAGCC.............. 1

.....................................................................GTAGAAGGAAGCCTGCACGAA...... 1

>cel-mir-353_MI0000752_Caenorhabditis_elegans_miR-353_stem-loop GSM444003

UGUUGAAGUAUUGCAAAAGCAAAGGACGGGCACAAUUGCCAUGUGUUGGUAUUAUUGCUUCAAGUUAUUUGAAGCUGUAAUAUCAAUAAGCAUGUCUCGUGUGAAGUCCG

..........((((....)))).((((...((((...(.((((((((((((((((.((((((((...)))))))).)))))))))))..))))).)...))))..)))). (-39.90)

.........ATTGCAAAAGCAAAGGACGGG................................................................................ 1

..........TTGCAAAAGCAAAGGACGGG................................................................................ 1

...........TGCAAAAGCAAAGGACGGG................................................................................ 1

..............AAAAGCAAAGGACGGGCA.............................................................................. 1

................AAGCAAAGGACGGGCACAAT.......................................................................... 1

................................CAATTGCCATGTGTTGGTAT.......................................................... 1

................................CAATTGCCATGTGTTGGTATTATTG..................................................... 1

.................................AATTGCCATGTGTTGGTATTA........................................................ 1

........................................ATGTGTTGGTATTATTGCTTCAAGT............................................. 1

........................................ATGTGTTGGTATTATTGCTTC................................................. 1

.................................................................TATTTGAAGCTGTAATATCAATAAGCA.................. 1

..................................................................ATTTGAAGCTGTAATATC.......................... 1

..................................................................ATTTGAAGCTGTAATATCAA........................ 1

..................................................................ATTTGAAGCTGTAATATCAATAAGC................... 2

..................................................................ATTTGAAGCTGTAATATCAATAAGCA.................. 1

...................................................................TTTGAAGCTGTAATATCAATAA..................... 1

.....................................................................TGAAGCTGTAATATCAAT....................... 1

........................................................................................AGCATGTCTCGTGTGAAG.... 1

>cel-mir-258-2_MI0010691_Caenorhabditis_elegans_miR-258-2_stem-loop GSM444003

GCAAUGGUUUUGAGAGGAAUCCUUUUACAUAUUUGUUGAAGUUUUCGCUCGAAUUUGUGGUCGAAUACUGUAGAAGGAAGCCUGCACGAAGUUUUG

.......(((((.((((..((((((((((((((((.....(..(((....)))..).....)))))).))))))))))..))).).)))))..... (-24.60)

.....GGTTTTGAGAGGAATCCTTTT...................................................................... 1

............................ATATTTGTTGAAGTTTTCGC................................................ 1

............................ATATTTGTTGAAGTTTTCGCTC.............................................. 1

..............................ATTTGTTGAAGTTTTCGCTC.............................................. 1

..................................GTTGAAGTTTTCGCTCGAAT.......................................... 1

..................................GTTGAAGTTTTCGCTCGAATT......................................... 1

.....................................GAAGTTTTCGCTCGAATT......................................... 1

.....................................GAAGTTTTCGCTCGAATTT........................................ 1

........................................GTTTTCGCTCGAATTTGT...................................... 2

..............................................GCTCGAATTTGTGGTCGAATA............................. 1

..............................................GCTCGAATTTGTGGTCGAATAC............................ 1

..................................................GAATTTGTGGTCGAATACTG.......................... 1

..................................................GAATTTGTGGTCGAATACTGT......................... 1

..................................................GAATTTGTGGTCGAATACTGTA........................ 2

..............................................................GAATACTGTAGAAGGAAGCC.............. 1

.....................................................................GTAGAAGGAAGCCTGCACGAA...... 1

>cel-mir-61_MI0000032_Caenorhabditis_elegans_miR-61_stem-loop GSM444004

UUCCAUUAUCGCUGAACCUCGAGAUGGGUUACGGGGCUUAGUCCUUCCUCCGUAUGGCAAUGACUAGAACCGUUACUCAUCUCGAGGUUUCGGUGAU

.......((((((((((((((((((((((.((((..((.((((.((((.......)).)).))))))..)))).))))))))))))).))))))))) (-50.50)

.TCCATTATCGCTGAACCTCG............................................................................ 12

.......................ATGGGTTACGGGGCTTAGTCCT.................................................... 4

.......................ATGGGTTACGGGGCTTAGTCCTT................................................... 8

........................TGGGTTACGGGGCTTAGTCC..................................................... 1

........................TGGGTTACGGGGCTTAGTCCT.................................................... 18

........................TGGGTTACGGGGCTTAGTCCTT................................................... 162

..............................ACGGGGCTTAGTCCTTCCTC............................................... 2

...........................................................ATGACTAGAACCGTTACTCAT................. 3

...........................................................ATGACTAGAACCGTTACTCATC................ 9

............................................................TGACTAGAACCGTTACTC................... 21

............................................................TGACTAGAACCGTTACTCAT................. 380

............................................................TGACTAGAACCGTTACTCATC................ 3130

............................................................TGACTAGAACCGTTACTCATCT............... 241

............................................................TGACTAGAACCGTTACTCATCTC.............. 1

............................................................TGACTAGAACCGTTACTCATCTCG............. 2

.............................................................GACTAGAACCGTTACTCATCT............... 2

..............................................................ACTAGAACCGTTACTCAT................. 2

..............................................................ACTAGAACCGTTACTCATC................ 7

...............................................................CTAGAACCGTTACTCATCTCG............. 21

>cel-mir-63_MI0000034_Caenorhabditis_elegans_miR-63_stem-loop GSM444004

UCAACAAGCAGACACAAUUUCUAACUCGUCGGUAGUCAUCGUUCUAGCUGAAAAGGACACUAUGACACUGAAGCGAGUUGGAAAUAGUGGUUCUACUUGAGCAA

....((((.((((((.(((((((((((((((((.(((((.(((((........)))))...)))))))))..))))))))))))).)))..))).))))..... (-38.00)

.CAACAAGCAGACACAATTTC................................................................................... 1

...............AATTTCTAACTCGTCGGTAGTC................................................................... 1

...................TCTAACTCGTCGGTAGTCATC................................................................ 1

...................TCTAACTCGTCGGTAGTCATCG............................................................... 25

...................TCTAACTCGTCGGTAGTCATCGT.............................................................. 106

...................TCTAACTCGTCGGTAGTCATCGTT............................................................. 7

....................CTAACTCGTCGGTAGTCATC................................................................ 1

.....................TAACTCGTCGGTAGTCAT................................................................. 1

..................................GTCATCGTTCTAGCTGAAAAGGACACT........................................... 1

............................................................TATGACACTGAAGCGAGT.......................... 42

............................................................TATGACACTGAAGCGAGTT......................... 62

............................................................TATGACACTGAAGCGAGTTG........................ 166

............................................................TATGACACTGAAGCGAGTTGG....................... 3789

............................................................TATGACACTGAAGCGAGTTGGAAAT................... 13

.............................................................ATGACACTGAAGCGAGTTGG....................... 3

.............................................................ATGACACTGAAGCGAGTTGGAAAT................... 1

................................................................ACACTGAAGCGAGTTGGAAAT................... 1

>cel-mir-354_MI0000753_Caenorhabditis_elegans_miR-354_stem-loop GSM444004

CAGAGCCGACUAAGCACCUUGGUGCGGCUGCAGACGGGUAUCCGGCUCGACGUUCAUACAUCACGACUUCUUUCCUUUUACCUUGUUUGUUGCUGCUCCUAUUGGUUUUG

(((((((((...........((.(((((.(((((((((((...((...((.(((..........))).))...))...)))).))))))).))))).))..))))))))) (-33.02)

..........TAAGCACCTTGGTGCGGCTGC............................................................................... 1

.............................GCAGACGGGTATCCGGCTCGAC........................................................... 1

................................GACGGGTATCCGGCTCGACGTTC....................................................... 1

.................................ACGGGTATCCGGCTCGACGTTC....................................................... 1

.......................................ATCCGGCTCGACGTTCATACATC................................................ 1

......................................................CATACATCACGACTTCTTTC.................................... 3

......................................................CATACATCACGACTTCTTTCC................................... 1

.......................................................ATACATCACGACTTCTTTCCTTTT............................... 1

.......................................................ATACATCACGACTTCTTTCC................................... 2

.......................................................ATACATCACGACTTCTTTCCTTTTACCT........................... 1

........................................................TACATCACGACTTCTTTCC................................... 1

.........................................................ACATCACGACTTCTTTCCTTTT............................... 1

.........................................................ACATCACGACTTCTTTCCTT................................. 1

.........................................................ACATCACGACTTCTTTCCTTTT............................... 1

..........................................................CATCACGACTTCTTTCCT.................................. 1

..........................................................CATCACGACTTCTTTCCTTTTACC............................ 1

...........................................................ATCACGACTTCTTTCCTT................................. 1

...........................................................ATCACGACTTCTTTCCTTTTACC............................ 1

...........................................................ATCACGACTTCTTTCCTTTTACCT........................... 1

...........................................................ATCACGACTTCTTTCCTTTTAC............................. 1

............................................................TCACGACTTCTTTCCTTTT............................... 1

............................................................TCACGACTTCTTTCCTTTTACCTT.......................... 1

.............................................................CACGACTTCTTTCCTTTT............................... 1

.............................................................CACGACTTCTTTCCTTTTAC............................. 1

..................................................................CTTCTTTCCTTTTACCTTGTTTGT.................... 1

..............................................................................TACCTTGTTTGTTGCTGCTC............ 1

...............................................................................ACCTTGTTTGTTGCTGCT............. 1

...............................................................................ACCTTGTTTGTTGCTGCTCCT.......... 2

..................................................................................TTGTTTGTTGCTGCTCCT.......... 1

...................................................................................TGTTTGTTGCTGCTCCTATTGGTTTT. 1

....................................................................................GTTTGTTGCTGCTCCTATT....... 1

>cel-mir-60_MI0000031_Caenorhabditis_elegans_miR-60_stem-loop GSM444005

CUCGAAAACCGCUUGUUCUUGAACUGGAAGAGUGCCAUAAAAUCAUGACAAAGUACGUGAUAUUAUGCACAUUUUCUAGUUCAAGACUUGAGAAAUCG

................(((((((((((((((((((.((((.((((((........)))))).)))))))).)))))))))))))))...((....)). (-35.00)

..CGAAAACCGCTTGTTCTTGAAC.......................................................................... 2

..................TTGAACTGGAAGAGTGCCAT............................................................ 1

.....................AACTGGAAGAGTGCCATAAAAT....................................................... 84

.....................AACTGGAAGAGTGCCATAAAATC...................................................... 342

.....................AACTGGAAGAGTGCCATAAAATCAT.................................................... 5

.....................AACTGGAAGAGTGCCATAAAATCATG................................................... 1

.....................AACTGGAAGAGTGCCATAAAATC...................................................... 1

......................ACTGGAAGAGTGCCATAAAAT....................................................... 1

......................ACTGGAAGAGTGCCATAAAATC...................................................... 5

......................ACTGGAAGAGTGCCATAAAATCAT.................................................... 1

......................ACTGGAAGAGTGCCATAAAATC...................................................... 1

.........................GGAAGAGTGCCATAAAATC...................................................... 1

..................................CCATAAAATCATGACAAAGTAC.......................................... 1

...........................................................ATATTATGCACATTTTCTAGTTC................ 32

............................................................TATTATGCACATTTTCTAG................... 1

............................................................TATTATGCACATTTTCTAGT.................. 1

............................................................TATTATGCACATTTTCTAGTT................. 85

............................................................TATTATGCACATTTTCTAGTTC................ 15509

............................................................TATTATGCACATTTTCTAGTTCAAG............. 5

.............................................................ATTATGCACATTTTCTAGTTC................ 10

..............................................................TTATGCACATTTTCTAGTTC................ 1

................................................................ATGCACATTTTCTAGTTC................ 1

>cel-mir-61_MI0000032_Caenorhabditis_elegans_miR-61_stem-loop GSM444005

UUCCAUUAUCGCUGAACCUCGAGAUGGGUUACGGGGCUUAGUCCUUCCUCCGUAUGGCAAUGACUAGAACCGUUACUCAUCUCGAGGUUUCGGUGAU

.......((((((((((((((((((((((.((((..((.((((.((((.......)).)).))))))..)))).))))))))))))).))))))))) (-50.50)

.TCCATTATCGCTGAACCTCG............................................................................ 1

.......................ATGGGTTACGGGGCTTAGTCCT.................................................... 1

.......................ATGGGTTACGGGGCTTAGTCCTT................................................... 1

........................TGGGTTACGGGGCTTAGTCCT.................................................... 11

........................TGGGTTACGGGGCTTAGTCCTT................................................... 69

........................TGGGTTACGGGGCTTAGTCCTTC.................................................. 1

...........................................................ATGACTAGAACCGTTACTCAT................. 4

...........................................................ATGACTAGAACCGTTACTCATC................ 22

...........................................................ATGACTAGAACCGTTACTCATCT............... 2

...........................................................ATGACTAGAACCGTTACTCATC................ 1

............................................................TGACTAGAACCGTTACTC................... 151

............................................................TGACTAGAACCGTTACTCAT................. 3226

............................................................TGACTAGAACCGTTACTCATC................ 22765

............................................................TGACTAGAACCGTTACTCATCT............... 2276

............................................................TGACTAGAACCGTTACTCATCTC.............. 30

............................................................TGACTAGAACCGTTACTCATCTCG............. 6

..............................................................ACTAGAACCGTTACTCATCTCG............. 5

...............................................................CTAGAACCGTTACTCATCTCG............. 25

>cel-mir-1817_MI0007979_Caenorhabditis_elegans_miR-1817_stem-loop GSM444005

UGUACAUUUCAAUUUUCGAGUAGCCAAUGUCUUCUCUAUCAUGCAUUUUACAAAUAAUGAGUACAUGAUAGUGAAAUAUUUGCUUCCUGAAUUUCAGAGAUGUUU

...(((((((....((((.(.(((.(((((.(((.((((((((.((((..........)))).)))))))).)))))))).))).).)))).....))))))).. (-27.30)

...ACATTTCAATTTTCGAGTAGCC................................................................................ 1

....................TAGCCAATGTCTTCTCTATC................................................................. 36

....................TAGCCAATGTCTTCTCTATCAT............................................................... 14

....................TAGCCAATGTCTTCTCTATCATG.............................................................. 393

....................TAGCCAATGTCTTCTCTATCATGC............................................................. 9

>cel-mir-60_MI0000031_Caenorhabditis_elegans_miR-60_stem-loop GSM443998

CUCGAAAACCGCUUGUUCUUGAACUGGAAGAGUGCCAUAAAAUCAUGACAAAGUACGUGAUAUUAUGCACAUUUUCUAGUUCAAGACUUGAGAAAUCG

................(((((((((((((((((((.((((.((((((........)))))).)))))))).)))))))))))))))...((....)). (-35.00)

..CGAAAACCGCTTGTTCTTGAAC.......................................................................... 2

.....................AACTGGAAGAGTGCCATAAAAT....................................................... 27

.....................AACTGGAAGAGTGCCATAAAATC...................................................... 59

.....................AACTGGAAGAGTGCCATAAAATCAT.................................................... 1

......................ACTGGAAGAGTGCCATAAAATC...................................................... 1

...........................................................ATATTATGCACATTTTCTAGTTC................ 6

............................................................TATTATGCACATTTTCTAG................... 1

............................................................TATTATGCACATTTTCTAGTT................. 4

............................................................TATTATGCACATTTTCTAGTTC................ 2595

.............................................................ATTATGCACATTTTCTAGTTC................ 1

>cel-mir-61_MI0000032_Caenorhabditis_elegans_miR-61_stem-loop GSM443998

UUCCAUUAUCGCUGAACCUCGAGAUGGGUUACGGGGCUUAGUCCUUCCUCCGUAUGGCAAUGACUAGAACCGUUACUCAUCUCGAGGUUUCGGUGAU

.......((((((((((((((((((((((.((((..((.((((.((((.......)).)).))))))..)))).))))))))))))).))))))))) (-50.50)

.TCCATTATCGCTGAACCTC............................................................................. 1

.TCCATTATCGCTGAACCTCGA........................................................................... 2

........................TGGGTTACGGGGCTTAGT....................................................... 1

........................TGGGTTACGGGGCTTAGTCC..................................................... 1

........................TGGGTTACGGGGCTTAGTCCTT................................................... 13

.....................................TTAGTCCTTCCTCCGTATGGCAA..................................... 1

..........................................CCTTCCTCCGTATGGCAATGAC................................. 1

.........................................................CAATGACTAGAACCGTTACTC................... 1

...........................................................ATGACTAGAACCGTTACTCAT................. 2

............................................................TGACTAGAACCGTTACTC................... 26

............................................................TGACTAGAACCGTTACTCA.................. 62

............................................................TGACTAGAACCGTTACTCAT................. 329

............................................................TGACTAGAACCGTTACTCATC................ 808

............................................................TGACTAGAACCGTTACTCATCT............... 42

...............................................................CTAGAACCGTTACTCATC................ 23

...............................................................CTAGAACCGTTACTCATCT............... 1

...............................................................CTAGAACCGTTACTCATCTCG............. 6

................................................................TAGAACCGTTACTCATCT............... 1

.......................................................................GTTACTCATCTCGAGGTTTC...... 2

>cel-mir-63_MI0000034_Caenorhabditis_elegans_miR-63_stem-loop GSM443998

UCAACAAGCAGACACAAUUUCUAACUCGUCGGUAGUCAUCGUUCUAGCUGAAAAGGACACUAUGACACUGAAGCGAGUUGGAAAUAGUGGUUCUACUUGAGCAA

....((((.((((((.(((((((((((((((((.(((((.(((((........)))))...)))))))))..))))))))))))).)))..))).))))..... (-38.00)

...................TCTAACTCGTCGGTAGTCAT................................................................. 1

...................TCTAACTCGTCGGTAGTCATC................................................................ 2

...................TCTAACTCGTCGGTAGTCATCG............................................................... 32

...................TCTAACTCGTCGGTAGTCATCGT.............................................................. 255

...................TCTAACTCGTCGGTAGTCATCGTT............................................................. 1

....................CTAACTCGTCGGTAGTCATCGT.............................................................. 1

....................................CATCGTTCTAGCTGAAAAGGAC.............................................. 1

............................................................TATGACACTGAAGCGAGT.......................... 41

............................................................TATGACACTGAAGCGAGTT......................... 13

............................................................TATGACACTGAAGCGAGTTG........................ 102

............................................................TATGACACTGAAGCGAGTTGG....................... 3345

.............................................................ATGACACTGAAGCGAGTTGG....................... 3

.............................................................................TTGGAAATAGTGGTTCTACTTG..... 1

>cel-mir-64_MI0000035_Caenorhabditis_elegans_miR-64_stem-loop GSM443998

CUCCCCGCUGACCUCGCCGAAUAUGACACUGAAGCGUUACCGAACCGUUUUCCCACACCUGGAUUCGGUGCAACGAUCAGUGGCAUGCUCGGCUAGCGCCAGUUAAGUAU

......((((.(((.(((((.((((.((((((..(((((((((((((............))).)))))))..))).)))))).)))).))))).)).).))))....... (-38.50)

.....CGCTGACCTCGCCGAATATGAC................................................................................... 1

.....CGCTGACCTCGCCGAATA....................................................................................... 1

....................ATATGACACTGAAGCGTTACCGA................................................................... 1

.....................TATGACACTGAAGCGTTA....................................................................... 46

.....................TATGACACTGAAGCGTTAC...................................................................... 337

.....................TATGACACTGAAGCGTTACC..................................................................... 1125

.....................TATGACACTGAAGCGTTACCG.................................................................... 139

.....................TATGACACTGAAGCGTTACCGA................................................................... 4587

.....................TATGACACTGAAGCGTTACCGAA.................................................................. 22847

.....................TATGACACTGAAGCGTTACCGAAC................................................................. 75

.....................TATGACACTGAAGCGTTACCGAACC................................................................ 38

.....................TATGACACTGAAGCGTTACCGAACCG............................................................... 14

......................ATGACACTGAAGCGTTAC...................................................................... 4

......................ATGACACTGAAGCGTTACC..................................................................... 8

......................ATGACACTGAAGCGTTACCG.................................................................... 6

......................ATGACACTGAAGCGTTACCGA................................................................... 86

......................ATGACACTGAAGCGTTACCGAA.................................................................. 349

......................ATGACACTGAAGCGTTACCGAAC................................................................. 2

.......................TGACACTGAAGCGTTACCGA................................................................... 7

.......................TGACACTGAAGCGTTACCGAA.................................................................. 34

........................GACACTGAAGCGTTACCG.................................................................... 3

........................GACACTGAAGCGTTACCGA................................................................... 98

........................GACACTGAAGCGTTACCGAA.................................................................. 476

........................GACACTGAAGCGTTACCGAAC................................................................. 1

.........................ACACTGAAGCGTTACCGA................................................................... 19

.........................ACACTGAAGCGTTACCGAA.................................................................. 118

.........................ACACTGAAGCGTTACCGAAC................................................................. 11

..........................CACTGAAGCGTTACCGAA.................................................................. 110

..........................CACTGAAGCGTTACCGAACC................................................................ 3

...........................ACTGAAGCGTTACCGAAC................................................................. 3

...........................ACTGAAGCGTTACCGAACC................................................................ 2

...........................ACTGAAGCGTTACCGAACCG............................................................... 1

............................CTGAAGCGTTACCGAACC................................................................ 1

............................................CCGTTTTCCCACACCTGGAT.............................................. 1

............................................CCGTTTTCCCACACCTGGATT............................................. 3

.................................................................CGGTGCAACGATCAGTGGCA......................... 1

.................................................................CGGTGCAACGATCAGTGGCATG....................... 1

.................................................................CGGTGCAACGATCAGTGGCATGC...................... 13

.................................................................CGGTGCAACGATCAGTGGCATGCT..................... 32

...................................................................GTGCAACGATCAGTGGCATG....................... 1

...................................................................GTGCAACGATCAGTGGCATGC...................... 1

...................................................................GTGCAACGATCAGTGGCATGCT..................... 3

....................................................................TGCAACGATCAGTGGCATGCT..................... 1

.......................................................................AACGATCAGTGGCATGCT..................... 4

.........................................................................................CGGCTAGCGCCAGTTAAGTA. 1

>cel-mir-75_MI0000046_Caenorhabditis_elegans_miR-75_stem-loop GSM443998

UUCUUGUUGCUUUGAAGAAUUGCAGUCGGUUGCAAGCUUAAAUACAAAUCCGAAUUGUUAUUAAAGCUACCAACCGGCUUCAAGUCUGAAAGAGCAG

.......((((((..(((.(((.(((((((((..(((((.(((((((.......))).)))).)))))..))))))))).))).)))...)))))). (-33.60)

.........CTTTGAAGAATTGCAGTC...................................................................... 1

......................CAGTCGGTTGCAAGCTTA......................................................... 3

......................CAGTCGGTTGCAAGCTTAA........................................................ 2

......................CAGTCGGTTGCAAGCTTAAA....................................................... 21

......................CAGTCGGTTGCAAGCTTAAAT...................................................... 30

......................CAGTCGGTTGCAAGCTTAAATA..................................................... 15

.......................AGTCGGTTGCAAGCTTAAA....................................................... 1

..........................CGGTTGCAAGCTTAAATA..................................................... 1

........................................AATACAAATCCGAATTGTTAT.................................... 1

..............................................AATCCGAATTGTTATTAAA................................ 1

..................................................CGAATTGTTATTAAAGCTAC........................... 1

............................................................TTAAAGCTACCAACCGGC................... 19

............................................................TTAAAGCTACCAACCGGCT.................. 24

............................................................TTAAAGCTACCAACCGGCTT................. 630

............................................................TTAAAGCTACCAACCGGCTTC................ 778

............................................................TTAAAGCTACCAACCGGCTTCA............... 4017

............................................................TTAAAGCTACCAACCGGCTTCAA.............. 28

............................................................TTAAAGCTACCAACCGGCTTCAAGT............ 1

.............................................................TAAAGCTACCAACCGGCTT................. 2

.............................................................TAAAGCTACCAACCGGCTTC................ 11

.............................................................TAAAGCTACCAACCGGCTTCA............... 45

..............................................................AAAGCTACCAACCGGCTT................. 3

..............................................................AAAGCTACCAACCGGCTTC................ 12

..............................................................AAAGCTACCAACCGGCTTCA............... 65

..............................................................AAAGCTACCAACCGGCTTCAAG............. 1

...............................................................AAGCTACCAACCGGCTTC................ 1

...............................................................AAGCTACCAACCGGCTTCA............... 48

................................................................AGCTACCAACCGGCTTCA............... 3

................................................................AGCTACCAACCGGCTTCAA.............. 1

>cel-mir-78_MI0000049_Caenorhabditis_elegans_miR-78_stem-loop GSM443998

AAUAAAAUAUAUUGUUUCAUAGUGUCCGUAAAAUAACUAGAUUUAUUUUGUAAAAACUAUUGGAGGCCUGGUUGUUUGUGCUGGAAUGUUUCGAGA

..............((((..((..((((((((((((((((.(((.....((....)).....)))..)))))))))).))).)).)..))..)))) (-15.00)

............................................ATTTTGTAAAAACTATTGGAGG.............................. 1

............................................ATTTTGTAAAAACTATTGGAG............................... 1

.........................................................TATTGGAGGCCTGGTTGT..................... 1

.........................................................TATTGGAGGCCTGGTTGTT.................... 1

.........................................................TATTGGAGGCCTGGTTGTTT................... 2

.........................................................TATTGGAGGCCTGGTTGTTTG.................. 2

...........................................................TTGGAGGCCTGGTTGTTTGT................. 1

............................................................TGGAGGCCTGGTTGTTTG.................. 1

............................................................TGGAGGCCTGGTTGTTTGT................. 7

............................................................TGGAGGCCTGGTTGTTTGTG................ 11

............................................................TGGAGGCCTGGTTGTTTGTGC............... 7

............................................................TGGAGGCCTGGTTGTTTGTGCT.............. 1

.......................................................................TTGTTTGTGCTGGAATGTTTC.... 1

.........................................................................GTTTGTGCTGGAATGTTTC.... 1

>cel-mir-80_MI0000051_Caenorhabditis_elegans_miR-80/miR-227_stem-loop GSM443998

AUGGACACUCGUUCGCUCAGCUUUCGACAUGAUUCUGAACAAUCCGCAAGCCCAUGUUGUUGAGAUCAUUAGUUGAAAGCCGAAUGAUCAGAGAUAUC

.......(((..(((.((.((((((((((((((.((.((((((.(....).....)))))).)))))))..))))))))).)).)))...)))..... (-30.50)

.....CACTCGTTCGCTCAGCTTTC......................................................................... 1

.......CTCGTTCGCTCAGCTTTC......................................................................... 1

..................AGCTTTCGACATGATTCT.............................................................. 2

..................AGCTTTCGACATGATTCTG............................................................. 2

..................AGCTTTCGACATGATTCTGA............................................................ 8

..................AGCTTTCGACATGATTCTGAA........................................................... 58

..................AGCTTTCGACATGATTCTGAAC.......................................................... 148

..................AGCTTTCGACATGATTCTGAACA......................................................... 1

..................AGCTTTCGACATGATTCTG............................................................. 1

...................GCTTTCGACATGATTCTGAAC.......................................................... 1

....................CTTTCGACATGATTCTGAAC.......................................................... 2

.....................TTTCGACATGATTCTGAAC.......................................................... 7

......................TTCGACATGATTCTGAAC.......................................................... 1

.........................GACATGATTCTGAACAATCCGC................................................... 1

.............................TGATTCTGAACAATCCGCAAGC............................................... 1

........................................AATCCGCAAGCCCATGTTGT...................................... 2

........................................AATCCGCAAGCCCATGTTGTTGAGA................................. 1

.......................................................GTTGTTGAGATCATTAGTTGAA..................... 1

...........................................................TTGAGATCATTAGTTGAAA.................... 2

...........................................................TTGAGATCATTAGTTGAAAG................... 4

...........................................................TTGAGATCATTAGTTGAAAGC.................. 1

...........................................................TTGAGATCATTAGTTGAAAGCC................. 5

...........................................................TTGAGATCATTAGTTGAAAGCCG................ 14

...........................................................TTGAGATCATTAGTTGAAAGCCGA............... 41

............................................................TGAGATCATTAGTTGAAA.................... 214

............................................................TGAGATCATTAGTTGAAAG................... 299

............................................................TGAGATCATTAGTTGAAAGC.................. 1461

............................................................TGAGATCATTAGTTGAAAGCC................. 1706

............................................................TGAGATCATTAGTTGAAAGCCG................ 3986

............................................................TGAGATCATTAGTTGAAAGCCGA............... 51716

............................................................TGAGATCATTAGTTGAAAGCCGAA.............. 139

............................................................TGAGATCATTAGTTGAAAGCCGAAT............. 16

.............................................................GAGATCATTAGTTGAAAGC.................. 3

.............................................................GAGATCATTAGTTGAAAGCC................. 9

.............................................................GAGATCATTAGTTGAAAGCCG................ 11

.............................................................GAGATCATTAGTTGAAAGCCGA............... 108

..............................................................AGATCATTAGTTGAAAGCC................. 3

..............................................................AGATCATTAGTTGAAAGCCG................ 1

..............................................................AGATCATTAGTTGAAAGCCGA............... 31

...............................................................GATCATTAGTTGAAAGCC................. 13

...............................................................GATCATTAGTTGAAAGCCG................ 55

...............................................................GATCATTAGTTGAAAGCCGA............... 944

...............................................................GATCATTAGTTGAAAGCCGAA.............. 6

................................................................ATCATTAGTTGAAAGCCG................ 105

................................................................ATCATTAGTTGAAAGCCGA............... 1725

................................................................ATCATTAGTTGAAAGCCGAA.............. 6

................................................................ATCATTAGTTGAAAGCCGAAT............. 1

.................................................................TCATTAGTTGAAAGCCGA............... 1300

.................................................................TCATTAGTTGAAAGCCGAA.............. 5

.................................................................TCATTAGTTGAAAGCCGAAT............. 4

..................................................................CATTAGTTGAAAGCCGAA.............. 10

..................................................................CATTAGTTGAAAGCCGAAT............. 1

>cel-mir-244_MI0000320_Caenorhabditis_elegans_miR-244_stem-loop GSM443998

CUCCAUAUCUCAAUCUCUUUGGUUGUACAAAGUGGUAUGGCUCAUCGAAUAAGCACAUACUGCUUUUCAGCUAAAGGAAUUGAGAUUUUGUAGGCUUUU

((.((.((((((((..(((((((((...((((..((((((((.........))).)))))..)))).)))))))))..))))))))..)).))...... (-38.20)

...............TCTTTGGTTGTACAAAGTGG................................................................ 3

...............TCTTTGGTTGTACAAAGTGGT............................................................... 135

...............TCTTTGGTTGTACAAAGTGGTAT............................................................. 109

...............TCTTTGGTTGTACAAAGTGGTATG............................................................ 2395

...............TCTTTGGTTGTACAAAGTGGTATGG........................................................... 2

................CTTTGGTTGTACAAAGTGGTATG............................................................ 1

..................TTGGTTGTACAAAGTGGTATG............................................................ 1

.........................................................TACTGCTTTTCAGCTAAAGG...................... 5

.........................................................TACTGCTTTTCAGCTAAAGGAAT................... 1

............................................................................GAATTGAGATTTTGTAGGCTTT. 1

.................................................................................GAGATTTTGTAGGCTTTT 1

>cel-mir-257_MI0000334_Caenorhabditis_elegans_miR-257_stem-loop GSM443998

AUAAUAUUUCCCGCUGAGUAUCAGGAGUACCCAGUGAUCGCCUUCACAUAUUGGGAAGAAGUAUGUGAUUGCGUCCUGCAGUUCUUCCAUGAUGUACUCAGG

.............((((((((((((((.((.(((.((.(((..(((((((((.......)))))))))..))))))))..))..)))).)))..))))))). (-33.80)

............GCTGAGTATCAGGAGTACC....................................................................... 1

............GCTGAGTATCAGGAGTACCC...................................................................... 1

............GCTGAGTATCAGGAGTACCCA..................................................................... 1

..........................GTACCCAGTGATCGCCTTCAC....................................................... 1

................................AGTGATCGCCTTCACATATT.................................................. 1

.................................GTGATCGCCTTCACATATTG................................................. 1

...............................................ATATTGGGAAGAAGTATGTGATT................................ 1

.................................................ATTGGGAAGAAGTATGTGATTG............................... 1

....................................................GGGAAGAAGTATGTGATTGCG............................. 1

.....................................................GGAAGAAGTATGTGATTGCGT............................ 1

......................................................GAAGAAGTATGTGATTGCGT............................ 1

.............................................................................GCAGTTCTTCCATGATGTACTC... 1

................................................................................GTTCTTCCATGATGTACTCAGG 4

>cel-mir-353_MI0000752_Caenorhabditis_elegans_miR-353_stem-loop GSM443998

UGUUGAAGUAUUGCAAAAGCAAAGGACGGGCACAAUUGCCAUGUGUUGGUAUUAUUGCUUCAAGUUAUUUGAAGCUGUAAUAUCAAUAAGCAUGUCUCGUGUGAAGUCCG

..........((((....)))).((((...((((...(.((((((((((((((((.((((((((...)))))))).)))))))))))..))))).)...))))..)))). (-39.90)

..............AAAAGCAAAGGACGGGCACA............................................................................ 1

....................................TGCCATGTGTTGGTATTATTGCTTC................................................. 1

........................................ATGTGTTGGTATTATTGCTTCAA............................................... 1

...........................................TGTTGGTATTATTGCTTCAA............................................... 1

............................................GTTGGTATTATTGCTTCAA............................................... 2

.............................................TTGGTATTATTGCTTCAAG.............................................. 1

....................................................................TTGAAGCTGTAATATCAATA...................... 1

.....................................................................TGAAGCTGTAATATCAATAA..................... 4

.............................................................................TAATATCAATAAGCATGTCTCG........... 1

>cel-mir-61_MI0000032_Caenorhabditis_elegans_miR-61_stem-loop GSM443999

UUCCAUUAUCGCUGAACCUCGAGAUGGGUUACGGGGCUUAGUCCUUCCUCCGUAUGGCAAUGACUAGAACCGUUACUCAUCUCGAGGUUUCGGUGAU

.......((((((((((((((((((((((.((((..((.((((.((((.......)).)).))))))..)))).))))))))))))).))))))))) (-50.50)

.TCCATTATCGCTGAACCTCGA........................................................................... 1

........................TGGGTTACGGGGCTTAGTCC..................................................... 1

........................TGGGTTACGGGGCTTAGTCCTT................................................... 2

............................................................TGACTAGAACCGTTACTC................... 12

............................................................TGACTAGAACCGTTACTCA.................. 13

............................................................TGACTAGAACCGTTACTCAT................. 57

............................................................TGACTAGAACCGTTACTCATC................ 42

............................................................TGACTAGAACCGTTACTCATCT............... 3

.............................................................GACTAGAACCGTTACTCAT................. 1

...............................................................CTAGAACCGTTACTCATC................ 2

.......................................................................GTTACTCATCTCGAGGTTTC...... 1

>cel-mir-63_MI0000034_Caenorhabditis_elegans_miR-63_stem-loop GSM443999

UCAACAAGCAGACACAAUUUCUAACUCGUCGGUAGUCAUCGUUCUAGCUGAAAAGGACACUAUGACACUGAAGCGAGUUGGAAAUAGUGGUUCUACUUGAGCAA

....((((.((((((.(((((((((((((((((.(((((.(((((........)))))...)))))))))..))))))))))))).)))..))).))))..... (-38.00)

...ACAAGCAGACACAATTTCTA................................................................................. 1

.....AAGCAGACACAATTTCTAAC............................................................................... 1

........CAGACACAATTTCTAACTCG............................................................................ 1

...................TCTAACTCGTCGGTAGTCAT................................................................. 1

...................TCTAACTCGTCGGTAGTCATCGT.............................................................. 1

......................AACTCGTCGGTAGTCATCGT.............................................................. 1

...........................GTCGGTAGTCATCGTTCTAGC........................................................ 1

....................................CATCGTTCTAGCTGAAAA.................................................. 1

.....................................ATCGTTCTAGCTGAAAAGGAC.............................................. 1

......................................TCGTTCTAGCTGAAAAGGAC.............................................. 1

...............................................CTGAAAAGGACACTATGAC...................................... 1

..........................................................ACTATGACACTGAAGCGAGTT......................... 1

...........................................................CTATGACACTGAAGCGAGTT......................... 1

............................................................TATGACACTGAAGCGAGT.......................... 1

............................................................TATGACACTGAAGCGAGTT......................... 1

............................................................TATGACACTGAAGCGAGTTG........................ 1

............................................................TATGACACTGAAGCGAGTTGG....................... 6

............................................................TATGACACTGAAGCGAGTTGGA...................... 3

............................................................TATGACACTGAAGCGAGTTGGAA..................... 30

............................................................TATGACACTGAAGCGAGTTGGAAA.................... 96

.............................................................ATGACACTGAAGCGAGTTGGAAA.................... 1

...............................................................GACACTGAAGCGAGTTGGAA..................... 1

...............................................................GACACTGAAGCGAGTTGGAAA.................... 2

................................................................ACACTGAAGCGAGTTGGAAA.................... 1

................................................................ACACTGAAGCGAGTTGGAAAT................... 1

.................................................................CACTGAAGCGAGTTGGAAA.................... 1

..................................................................ACTGAAGCGAGTTGGAAA.................... 5

....................................................................TGAAGCGAGTTGGAAATAGT................ 1

........................................................................GCGAGTTGGAAATAGTGGTTC........... 1

..................................................................................AATAGTGGTTCTACTTGA.... 1
