# Supplementary material for: Multiple distinct small RNAs originate from the same microRNA precursors
Source: Genome Biol. 2010 Aug 9;11(8):R81. doi: 10.1186/gb-2010-11-8-r81 (PMC2945783; doi:10.1186/gb-2010-11-8-r81)
Supplement: Additional file 10 — Supplemental File S9. This is a file for sequencing reads mapped and aligned to miRNA precursors that can produce miRNA-sibling small RNAs (msRNAs) in D. melanogaster (dme). The sequencing data were obtained from GEO; see Materials and methods for details. [file gb-2010-11-8-r81-S10.DOCX]

Zhang, et al., Multiple distinct small RNAs originate from the same microRNA precursors

Supplemental File 9 - Sequencing reads mapped and aligned to miRNA precursors that can produce

miRNA-like RNAs in *Drosophila melanogaster.*

>dme-mir-279_MI0000363_Drosophila_melanogaster_miR-279_stem-loop GSM272651

GGAAUUCAUACUACUGUUUUUAGUGGGUGGGGGUCCAGUGUUUCACAUUGAUUUUCUUAGUAUUUGUGACUAGAUCCACACUCAUUAAUAACGGUAGUUC

.........(((((((((.((((((((((.(((((.((....(((((...(((.....)))...))))))).))))).)))))))))).))))))))).. (-36.40)

.GAATTCATACTACTGTTTTT............................................................................... 1

.....................AGTGGGTGGGGGTCCAGTGTTTC........................................................ 3

.....................AGTGGGTGGGGGTCCAGTGTTTCACA..................................................... 4

.....................AGTGGGTGGGGGTCCAGTGTTT......................................................... 2

.....................AGTGGGTGGGGGTCCAGTGT........................................................... 1

.....................AGTGGGTGGGGGTCCAGTGTTTCAC...................................................... 1

.....................AGTGGGTGGGGGTCCAGTGTTTCA....................................................... 5

......................GTGGGTGGGGGTCCAGTGTTTCACA..................................................... 2

......................GTGGGTGGGGGTCCAGTGTTTCAC...................................................... 2

......................GTGGGTGGGGGTCCAGTGTTTC........................................................ 1

......................GTGGGTGGGGGTCCAGTGTTT......................................................... 4

......................GTGGGTGGGGGTCCAGTGTTTCA....................................................... 3

...........................TGGGGGTCCAGTGTTTCACA..................................................... 2

..................................................................TGACTAGATCCACACTCATT.............. 1049

..................................................................TGACTAGATCCACACTCATTAAT........... 2

..................................................................TGACTAGATCCACACTCATTA............. 2141

..................................................................TGACTAGATCCACACTCAT............... 121

..................................................................TGACTAGATCCACACTCA................ 8

..................................................................TGACTAGATCCACACTCATTAA............ 1514

...................................................................GACTAGATCCACACTCATTAA............ 3

...................................................................GACTAGATCCACACTCATT.............. 3

......................................................................TAGATCCACACTCATTAA............ 1

>dme-mir-33_MI0000364_Drosophila_melanogaster_miR-33_stem-loop GSM272651

CUCUUCCUCUGGAGAUGACACGAAGGUGCAUUGUAGUCGCAUUGUCUGUCCCAAUUGCUUCAGGCAAUACAACUUCAGUGCAAGCUCUGUGCAUUUCAC

...........((((((.((((.((.(((((((.(((.(.((((((((...(....)...)))))))).).))).)))))))..)).)))))))))).. (-33.20)

.....CCTCTGGAGATGACACGAAG.......................................................................... 3

.........................GTGCATTGTAGTCGCATTGTCTGT.................................................. 2

.........................GTGCATTGTAGTCGCATTGTCTG................................................... 13

.........................GTGCATTGTAGTCGCATTG....................................................... 17

.........................GTGCATTGTAGTCGCATTGTC..................................................... 436

.........................GTGCATTGTAGTCGCATTGT...................................................... 199

.........................GTGCATTGTAGTCGCATT........................................................ 7

.........................GTGCATTGTAGTCGCATTGTCT.................................................... 12

..........................TGCATTGTAGTCGCATTGTCT.................................................... 1

..........................TGCATTGTAGTCGCATTGTC..................................................... 1

...........................GCATTGTAGTCGCATTGT...................................................... 1

..............................................TGTCCCAATTGCTTCAGG................................... 17

..............................................................GGCAATACAACTTCAGTGCA................. 1

..............................................................GGCAATACAACTTCAGTGCAA................ 1

..............................................................GGCAATACAACTTCAGTGCAAG............... 3

..............................................................GGCAATACAACTTCAGTGCAAGC.............. 10

..............................................................GGCAATACAACTTCAGTGC.................. 1

...............................................................GCAATACAACTTCAGTGCAAG............... 1

................................................................CAATACAACTTCAGTGCAAGCT............. 32

................................................................CAATACAACTTCAGTGCA................. 1

................................................................CAATACAACTTCAGTGCAAGC.............. 4

................................................................CAATACAACTTCAGTGCAA................ 1

................................................................CAATACAACTTCAGTGCAAG............... 1

>dme-mir-999_MI0005861_Drosophila_melanogaster_miR-999_stem-loop GSM272651

AAGGAUGCCGCUCAAUUACCCCGACAUAGUCAUACGGUGAAUGUUGUGUAUUGGAGACCAAUGUUAACUGUAAGACUGUGUCUCGGUGGUUGCCAGCCCAGCCAC

..((.((..(((((((((((..(((((((((.((((((.(((((((.((.......))))))))).)))))).)))))))))..))))))))..))).)).)).. (-40.80)

...GATGCCGCTCAATTACCCCG.................................................................................. 1

.....TGCCGCTCAATTACCCCG.................................................................................. 1

.......................ACATAGTCATACGGTGAATGTT............................................................ 4

.......................ACATAGTCATACGGTGAATGTTG........................................................... 1

............................................................ATGTTAACTGTAAGACTGTGTCT...................... 1

.............................................................TGTTAACTGTAAGACTGTGTCT...................... 74

..............................................................GTTAACTGTAAGACTGTGTCT...................... 1

>dme-mir-277_MI0000360_Drosophila_melanogaster_miR-277_stem-loop GSM272652

UUGAAGGUUUUGGGCUGCGUGUCAGGAGUGCAUUUGCACUGAAACUAUCUGAAGCAUGUAAAUGCACUAUCUGGUACGACAUUCCAGAACGUACAAUCUU

(((.(.(((((((..(((((..((((((((((((((((.((...(.....)...)))))))))))))).))))..))).))..))))))).).))).... (-40.30)

.TGAAGGTTTTGGGCTGCGTGT.............................................................................. 1

................GCGTGTCAGGAGTGCATTTGCA.............................................................. 3

.................CGTGTCAGGAGTGCATTTG................................................................ 17

.................CGTGTCAGGAGTGCATTTGCA.............................................................. 316

.................CGTGTCAGGAGTGCATTTGC............................................................... 60

.................CGTGTCAGGAGTGCATTTGCAC............................................................. 1

......................................CTGAAACTATCTGAAGCATG.......................................... 4

..........................................................TAAATGCACTATCTGGTACGA..................... 659

..........................................................TAAATGCACTATCTGGTACGACA................... 1628

..........................................................TAAATGCACTATCTGGTACGACAT.................. 4

..........................................................TAAATGCACTATCTGGTAC....................... 5

..........................................................TAAATGCACTATCTGGTACG...................... 29

..........................................................TAAATGCACTATCTGGTACGAC.................... 951

..........................................................TAAATGCACTATCTGGTA........................ 2

...........................................................AAATGCACTATCTGGTACGAC.................... 9

...........................................................AAATGCACTATCTGGTACG...................... 2

...........................................................AAATGCACTATCTGGTACGA..................... 4

...........................................................AAATGCACTATCTGGTACGACA................... 2

............................................................AATGCACTATCTGGTACGACA................... 2

............................................................AATGCACTATCTGGTACGAC.................... 2

............................................................AATGCACTATCTGGTACGA..................... 4

.............................................................ATGCACTATCTGGTACGAC.................... 1

..............................................................TGCACTATCTGGTACGAC.................... 1

>dme-mir-33_MI0000364_Drosophila_melanogaster_miR-33_stem-loop GSM272652

CUCUUCCUCUGGAGAUGACACGAAGGUGCAUUGUAGUCGCAUUGUCUGUCCCAAUUGCUUCAGGCAAUACAACUUCAGUGCAAGCUCUGUGCAUUUCAC

...........((((((.((((.((.(((((((.(((.(.((((((((...(....)...)))))))).).))).)))))))..)).)))))))))).. (-33.20)

....TCCTCTGGAGATGACACGAAG.......................................................................... 3

.....CCTCTGGAGATGACACGAAG.......................................................................... 9

.........................GTGCATTGTAGTCGCATTG....................................................... 18

.........................GTGCATTGTAGTCGCATTGTCTG................................................... 25

.........................GTGCATTGTAGTCGCATTGTC..................................................... 1164

.........................GTGCATTGTAGTCGCATTGT...................................................... 392

.........................GTGCATTGTAGTCGCATTGTCTGT.................................................. 2

.........................GTGCATTGTAGTCGCATTGTCT.................................................... 31

.........................GTGCATTGTAGTCGCATT........................................................ 25

..........................TGCATTGTAGTCGCATTGTCT.................................................... 1

..........................TGCATTGTAGTCGCATTGTC..................................................... 2

..........................TGCATTGTAGTCGCATTGTCTG................................................... 1

............................CATTGTAGTCGCATTGTC..................................................... 1

..............................TTGTAGTCGCATTGTCTG................................................... 1

..............................................TGTCCCAATTGCTTCAGG................................... 55

..............................................................GGCAATACAACTTCAGTGCAA................ 3

..............................................................GGCAATACAACTTCAGTGCAAG............... 8

..............................................................GGCAATACAACTTCAGTGCAAGC.............. 38

...............................................................GCAATACAACTTCAGTGCAAG............... 3

...............................................................GCAATACAACTTCAGTGCAAGC.............. 13

................................................................CAATACAACTTCAGTGCAAGCT............. 119

................................................................CAATACAACTTCAGTGCA................. 1

................................................................CAATACAACTTCAGTGCAAGC.............. 13

................................................................CAATACAACTTCAGTGCAAG............... 1

>dme-mir-988_MI0005847_Drosophila_melanogaster_miR-988_stem-loop GSM272652

GACGGCGGUACCGGGCAUUUUGGGUGUGUGAUUUGUAGCAAAGUGAUAUGUAUUUGAUCAUCCCCUUGUUGCAAACCUCACGCCAAAGAUGAUCUGCGA

....((((.......((((((.((((((.(.((((((((((.(((((.........)))))....)))))))))).).)))))).))))))..)))).. (-31.10)

.....CGGTACCGGGCATTTTGGGT.......................................................................... 2

......GGTACCGGGCATTTTGGGT.......................................................................... 1

.......GTACCGGGCATTTTGGGT.......................................................................... 2

........................TGTGTGATTTGTAGCAAAGTGAT.................................................... 1

.........................GTGTGATTTGTAGCAAAGTGAT.................................................... 121

.........................GTGTGATTTGTAGCAAAGTGATA................................................... 5

.........................GTGTGATTTGTAGCAAAGTG...................................................... 14

.........................GTGTGATTTGTAGCAAAGTGA..................................................... 201

.........................GTGTGATTTGTAGCAAAGT....................................................... 1

.........................GTGTGATTTGTAGCAAAG........................................................ 3

..........................TGTGATTTGTAGCAAAGTGAT.................................................... 2

..........................TGTGATTTGTAGCAAAGTGA..................................................... 5

............................................................TCCCCTTGTTGCAAACCTCAC.................. 1

............................................................TCCCCTTGTTGCAAACCTCACGC................ 7

............................................................TCCCCTTGTTGCAAACCTCA................... 1

............................................................TCCCCTTGTTGCAAACCTCACG................. 22

.............................................................CCCCTTGTTGCAAACCTCAC.................. 1035

.............................................................CCCCTTGTTGCAAACCTCA................... 537

.............................................................CCCCTTGTTGCAAACCTC.................... 49

.............................................................CCCCTTGTTGCAAACCTCACG................. 8335

.............................................................CCCCTTGTTGCAAACCTCACGC................ 3322

.............................................................CCCCTTGTTGCAAACCTCACGCC............... 4

..............................................................CCCTTGTTGCAAACCTCACGC................ 59

..............................................................CCCTTGTTGCAAACCTCA................... 2

..............................................................CCCTTGTTGCAAACCTCACGCC............... 1

..............................................................CCCTTGTTGCAAACCTCACG................. 40

..............................................................CCCTTGTTGCAAACCTCAC.................. 4

...............................................................CCTTGTTGCAAACCTCAC.................. 1

...............................................................CCTTGTTGCAAACCTCACGCC............... 2

...............................................................CCTTGTTGCAAACCTCACGC................ 10

...............................................................CCTTGTTGCAAACCTCACG................. 5

................................................................CTTGTTGCAAACCTCACG................. 6

................................................................CTTGTTGCAAACCTCACGC................ 1

>dme-mir-33_MI0000364_Drosophila_melanogaster_miR-33_stem-loop GSM272653

CUCUUCCUCUGGAGAUGACACGAAGGUGCAUUGUAGUCGCAUUGUCUGUCCCAAUUGCUUCAGGCAAUACAACUUCAGUGCAAGCUCUGUGCAUUUCAC

...........((((((.((((.((.(((((((.(((.(.((((((((...(....)...)))))))).).))).)))))))..)).)))))))))).. (-33.20)

....TCCTCTGGAGATGACACGAAG.......................................................................... 2

.....CCTCTGGAGATGACACGAAG.......................................................................... 4

......CTCTGGAGATGACACGAAG.......................................................................... 1

.........................GTGCATTGTAGTCGCATTG....................................................... 34

.........................GTGCATTGTAGTCGCATTGTCT.................................................... 15

.........................GTGCATTGTAGTCGCATTGTCTG................................................... 18

.........................GTGCATTGTAGTCGCATTGTC..................................................... 1727

.........................GTGCATTGTAGTCGCATTGT...................................................... 580

.........................GTGCATTGTAGTCGCATT........................................................ 32

..........................TGCATTGTAGTCGCATTGTCT.................................................... 1

..........................TGCATTGTAGTCGCATTGT...................................................... 1

............................CATTGTAGTCGCATTGTC..................................................... 1

.............................ATTGTAGTCGCATTGTCTG................................................... 1

..............................................TGTCCCAATTGCTTCAGG................................... 24

..............................................................GGCAATACAACTTCAGTGCAA................ 3

..............................................................GGCAATACAACTTCAGTGCAAGC.............. 9

..............................................................GGCAATACAACTTCAGTGC.................. 1

...............................................................GCAATACAACTTCAGTGCAAG............... 5

...............................................................GCAATACAACTTCAGTGCAA................ 1

...............................................................GCAATACAACTTCAGTGCAAGC.............. 14

................................................................CAATACAACTTCAGTGCAAGCT............. 193

................................................................CAATACAACTTCAGTGCA................. 4

................................................................CAATACAACTTCAGTGCAAGC.............. 16

................................................................CAATACAACTTCAGTGCAAG............... 1

.................................................................AATACAACTTCAGTGCAAGC.............. 1

...................................................................TACAACTTCAGTGCAAGC.............. 1

>dme-mir-283_MI0000368_Drosophila_melanogaster_miR-283_stem-loop GSM272653

CUCACACGAUUCUCAAAGGUAAAUAUCAGCUGGUAAUUCUGGGAGCUAAGCCUAAAUAUGAAACACUCGGAAUUUCAGUUGGUAUCGACUUUUUUGAAUU

.......(((((..((((((..(((((((((((.(((((((((......................))))))))))))))))))))..))))))..))))) (-29.55)

CTCACACGATTCTCAAAGGT................................................................................ 6

.TCACACGATTCTCAAAGGT................................................................................ 49

..CACACGATTCTCAAAGGT................................................................................ 5

...................TAAATATCAGCTGGTAATTCTGGG......................................................... 1

...................TAAATATCAGCTGGTAATTCT............................................................ 1

...................TAAATATCAGCTGGTAATTC............................................................. 1

....................AAATATCAGCTGGTAATTCTG........................................................... 22

....................AAATATCAGCTGGTAATTCTGGGA........................................................ 5

....................AAATATCAGCTGGTAATTCT............................................................ 155

....................AAATATCAGCTGGTAATT.............................................................. 5

....................AAATATCAGCTGGTAATTCTGGG......................................................... 435

....................AAATATCAGCTGGTAATTC............................................................. 7

....................AAATATCAGCTGGTAATTCTGG.......................................................... 70

.....................AATATCAGCTGGTAATTCT............................................................ 2

.....................AATATCAGCTGGTAATTCTGGG......................................................... 12

......................ATATCAGCTGGTAATTCTGGG......................................................... 7

.......................TATCAGCTGGTAATTCTGGG......................................................... 5

.......................TATCAGCTGGTAATTCTGGGAGC...................................................... 1

...........................................AGCTAAGCCTAAATATGAAACACT................................. 9

..............................................TAAGCCTAAATATGAAAC.................................... 1

..................................................................TCGGAATTTCAGTTGGTATCG............. 1

..................................................................TCGGAATTTCAGTTGGTATCGAC........... 1

..................................................................TCGGAATTTCAGTTGGTATCGA............ 6

...................................................................CGGAATTTCAGTTGGTATCGA............ 55

...................................................................CGGAATTTCAGTTGGTATCGACT.......... 1

...................................................................CGGAATTTCAGTTGGTATCG............. 31

...................................................................CGGAATTTCAGTTGGTATC.............. 1

...................................................................CGGAATTTCAGTTGGTATCGAC........... 32

>dme-mir-33_MI0000364_Drosophila_melanogaster_miR-33_stem-loop GSM272691

CUCUUCCUCUGGAGAUGACACGAAGGUGCAUUGUAGUCGCAUUGUCUGUCCCAAUUGCUUCAGGCAAUACAACUUCAGUGCAAGCUCUGUGCAUUUCAC

...........((((((.((((.((.(((((((.(((.(.((((((((...(....)...)))))))).).))).)))))))..)).)))))))))).. (-33.20)

....TCCTCTGGAGATGACACGAAG.......................................................................... 1

.........................GTGCATTGTAGTCGCATTG....................................................... 5

.........................GTGCATTGTAGTCGCATTGTCT.................................................... 1

.........................GTGCATTGTAGTCGCATTGTC..................................................... 83

.........................GTGCATTGTAGTCGCATTGT...................................................... 9

.........................GTGCATTGTAGTCGCATT........................................................ 3

..............................................TGTCCCAATTGCTTCAGG................................... 5

..............................................................GGCAATACAACTTCAGTGCAAGC.............. 1

................................................................CAATACAACTTCAGTGCAAGCT............. 12

................................................................CAATACAACTTCAGTGCA................. 1

>dme-mir-283_MI0000368_Drosophila_melanogaster_miR-283_stem-loop GSM272691

CUCACACGAUUCUCAAAGGUAAAUAUCAGCUGGUAAUUCUGGGAGCUAAGCCUAAAUAUGAAACACUCGGAAUUUCAGUUGGUAUCGACUUUUUUGAAUU

.......(((((..((((((..(((((((((((.(((((((((......................))))))))))))))))))))..))))))..))))) (-29.55)

CTCACACGATTCTCAAAGGT................................................................................ 1

.TCACACGATTCTCAAAGGT................................................................................ 1

..CACACGATTCTCAAAGGT................................................................................ 1

....................AAATATCAGCTGGTAATTCTGGG......................................................... 13

....................AAATATCAGCTGGTAATTCTGG.......................................................... 1

.......................TATCAGCTGGTAATTCTGGG......................................................... 1

...................................................................CGGAATTTCAGTTGGTATCGA............ 6

...................................................................CGGAATTTCAGTTGGTATCG............. 2

...................................................................CGGAATTTCAGTTGGTATCGAC........... 3

>dme-mir-964_MI0005819_Drosophila_melanogaster_miR-964_stem-loop GSM272691

CAAUAACAUAUUGGUCCAACUUGCCUUAGAAUAGGGGAGCUUAACUUAUGUUUUUGAUGUUUAAGUUAAAAGCCUCUGUUCUAAGACAAUUUGAUGAUCA

...........((((((((.(((.(((((((((((((...((((((((.............))))))))...))))))))))))).))).)))..))))) (-31.62)

.....ACATATTGGTCCAACTTGCC........................................................................... 1

......CATATTGGTCCAACTTGCC........................................................................... 2

......CATATTGGTCCAACTTGCCT.......................................................................... 1

.......ATATTGGTCCAACTTGCC........................................................................... 7

.........................TTAGAATAGGGGAGCTTAACT...................................................... 29

.........................TTAGAATAGGGGAGCTTAACTT..................................................... 190

.........................TTAGAATAGGGGAGCTTAA........................................................ 2

.........................TTAGAATAGGGGAGCTTA......................................................... 1

.........................TTAGAATAGGGGAGCTTAAC....................................................... 2

..........................TAGAATAGGGGAGCTTAACTTA.................................................... 40

..........................TAGAATAGGGGAGCTTAACT...................................................... 18

..........................TAGAATAGGGGAGCTTAA........................................................ 1

..........................TAGAATAGGGGAGCTTAACTT..................................................... 64

..........................TAGAATAGGGGAGCTTAAC....................................................... 2

...........................AGAATAGGGGAGCTTAACTT..................................................... 1

............................GAATAGGGGAGCTTAACTT..................................................... 2

...............................................................AGTTAAAAGCCTCTGTTCTAAG............... 2

...............................................................AGTTAAAAGCCTCTGTTCTAA................ 1

................................................................GTTAAAAGCCTCTGTTCTAAG............... 7

................................................................GTTAAAAGCCTCTGTTCTAAGA.............. 5

................................................................GTTAAAAGCCTCTGTTCTAA................ 1

.................................................................TTAAAAGCCTCTGTTCTAAGA.............. 2

..................................................................TAAAAGCCTCTGTTCTAAGACA............ 1

>dme-mir-976_MI0005833_Drosophila_melanogaster_miR-976_stem-loop GSM272691

CAUCGCCAUGCAGUGCCGCGGCAUUGGUGAGGCCAUCUCCAAUGGAUUAGUUCUCAACAUUGGAUUAGUUAUCAUCAAUGCCGGUGCACUGCACCUA

........(((((((((.((((((((((((.......(((((((.............))))))).......))))))))))))).)))))))).... (-43.76)

..TCGCCATGCAGTGCCGCGGC........................................................................... 3

...CGCCATGCAGTGCCGCGGC........................................................................... 10

.....................CATTGGTGAGGCCATCTCCAAT...................................................... 1

......................ATTGGTGAGGCCATCTCCAATG..................................................... 1

..........................................................ATTGGATTAGTTATCATCAATGCC............... 1

..........................................................ATTGGATTAGTTATCATCAATGC................ 1

...........................................................TTGGATTAGTTATCATCAATGCC............... 15

...........................................................TTGGATTAGTTATCATCAATGC................ 35

...........................................................TTGGATTAGTTATCATCAATG................. 2

............................................................TGGATTAGTTATCATCAATGCC............... 13

>dme-mir-997_MI0005859_Drosophila_melanogaster_miR-997_stem-loop GSM272691

UUAUGGAUCCUCUUUCAAUGAAUUUAGUAUGCCCAAACUCGAAGGAGUUUCACCUCCAUAAGAGCGACAGUCCUGGAGAAGUUAUCAGAGCCAAAAAAAUUCAUAUGAUGAUGCAUUUUCCGUCUCUGAAAACGUCUUCAGCAGAAGUUGUUUUUAGCGAAGUGAAACUCAUUCGAUUUUGAUCAUACUAACGACAUUGGAUGCUUGGAUCGGCA

.....(((((...(((((((...((((((((..((((.(((((.(((((((((.((..((((((((((..(((((((((.(((.((((((.(...(((((.(((......))).)))))..).)))))).))).)))))))..)).))))))))))..)).))))))))).))))).))))..))))))))...))))))).....))))).... (-78.20)

...........................TATGCCCAAACTCGAAGGAGTTT..................................................................................................................................................................... 1

............................ATGCCCAAACTCGAAGGAGTTTCA................................................................................................................................................................... 1

.............................TGCCCAAACTCGAAGGAGTTTC.................................................................................................................................................................... 1

...............................CCCAAACTCGAAGGAGTTTCA................................................................................................................................................................... 116

................................CCAAACTCGAAGGAGTTTCA................................................................................................................................................................... 1

....................................................CCTCCATAAGAGCGACAGTCCTGGA.......................................................................................................................................... 1

....................................................CCTCCATAAGAGCGACAGTCCTGGAG......................................................................................................................................... 1

....................................................................AGTCCTGGAGAAGTTATCA................................................................................................................................ 1

....................................................................................................................................................................AAACTCATTCGATTTTGATCA.............................. 2
